
JOURNAL INFORMATION
==============================
NLM Title Abbreviation: JBMR Plus
Journal ID: JBMR Plus
NLM Journal ID: 101707013
Journal ID: jbmrplus

JBMR Plus

EISSN: 2473-4039
Publisher: Oxford University Press

ARTICLE INFORMATION
==============================
PMCID: PMC13100505
DOI: 10.1093/jbmrpl/ziag066
Article ID: ziag066
Article version: 1
Subjects: Abstracts, AcademicSubjects/MED00010, AcademicSubjects/MED00160, AcademicSubjects/MED00250

Abstracts of the ECTS 2026 Congress

Publication date: 2026 Apr
Electronic publication date: 2026 Apr 22
Volume: 10
Issue: Suppl 1
First page: i1
Last page: i319
Copyright: © The Author(s) 2026. Published by Oxford University Press on behalf of the American Society for Bone and Mineral Research.
Copyright year: 2026
License: This is an Open Access article distributed under the terms of the Creative Commons Attribution License (https://creativecommons.org/licenses/by/4.0/), which permits unrestricted reuse, distribution, and reproduction in any medium, provided the original work is properly cited.
License URL: https://creativecommons.org/licenses/by/4.0/

Erratum in: Correction to: Abstracts of the ECTS 2026 Congress 10 12 10 11 2026 ziag181 JBMR Plus 10.1093/jbmrpl/ziag181 PMC13750166 43011859
Page count: 1

==============================

### Abstracts of the ECTS 2026 Congress

### ECTS 2026 Congress

53rd European Calcified Tissue Society Congress

24 – 27 April 2026

**Scientific Programme Committee**

**Chair:**
Peter Pietschmann (Vienna, Austria)

**Co-Chairs:**
Martine Cohen-Solal (Paris, France)
**Clinical Co-Chair:**
Barbara Hauser (Edinburgh, United Kingdom)
**Basic Co-Chair:**
Kent Søe (Odense, Denmark)
**Local Organizing Committee (LOC)-Chair:**
Núria Guañabens (Barcelona, Spain)

**Members:**
Roland Chapurlat (Lyon, France)
Judit Donath (Budapest, Hungary)
Nadia Rucci (L'Aquila, Italy)
Gonzalo Sanchez-Duffhues (Oviedo, Spain)
Jan Tuckermann (Ulm, Germany)

**International Associate Members:**
Aliya Khan (Hamilton, Canada)
Andre van Wijnen (Burlington, Canada)

**Local Organising Committee:**
Núria Guañabens (Barcelona, Spain) LOC Co-Chair
Pilar Peris (Barcelona, Spain)
Cristina Carbonell (Barcelona, Spain)
Dacia Cerda (Barcelona, Spain)
Laia Gifre (Barcelona, Spain)
Xavier Nogues (Barcelona, Spain)
Delia Reina (Barcelona, Spain)
Patricia Reyner (Girona, Spain)
Daniel Roig (Sant Joan Despí, Spain)
Gonzalo Sanchez-Duffhues (Oviedo, Spain)

**Abstract Review Panel**

Each abstract was scored blind.

Bo Abrahamsen (Denmark)
Giovanni Adami (Italy)
Nerea Alonso (Ausrtria)
Athanasios Anastasilakis (Greece)
Christina Andreasen (Denmark)
Natasha Appelman-Dijkstra (Netherlands)
Korhan Baklacı (Turkey)
Ulrike Baschant (Germany)
Duncan Bassett (United Kingdom)
Giulia Battafarano (Italy)
Genevieve Baujat (France)
Teresita Bellido (United States)
Francis Berenbaum (France)
Clemens Bergwitz (United States)
Camille Blandin (France)
Claudine Blin (France)
Jean-Jacques Body (Belgium)
Nicolas Bonnet (Switzerland)
Annemieke Boot (Netherlands)
Roger Bouillon (Belgium)
Béatrice Bouvard (France)
Aline Bozec (Germany)
Nathalie Bravenboer (Netherlands)
Franziska Brinkmann (Germany)
Karine Briot (France)
Ludmila Brunerova (Czech Republic)
Natalie C Butterfield (United Kingdom)
John Carey (Ireland)
Geert Carmeliet (Belgium)
Mara Carsote (Romania)
Antonino Catalano (Italy)
Dacia Cerdà Gabaroi (Spain)
Andrei Chagin (Sweden)
Roland Chapurlat (France)
Chantal Chenu (United Kingdom)
Gavin Clunie (United Kingdom)
Martine Cohen-Solal (France)
Graziana Colaianni (Italy)
Luciano Colangelo (Italy)
Céline Colnot (France)
Juliet Compston (United Kingdom)
Edward Czerwiński (Poland)
Emilie Dambroise (France)
Bess Dawson-Hughes (United States)
Teun De Vries (Netherlands)
Judit Donáth (Hungary)
Stella D'oronzo (Italy)
Eleni Douni (Greece)
Patricia Ducy (United States)
Gustavo Duque (Canada)
Ivan Duran (United States)

Richard Eastell (United Kingdom)
Grahame Elder (Australia)
Florent Elefteriou (United States)
Ari Elson (Israel)
Karol Estrada (United States)
Colin Farquharson (United Kingdom)
Verena Fischer (Germany)
Lars Folkestad (Denmark)
Antonella Forlino (Italy)
Melissa M Formosa (Malta)
Nadja Fratzl-Zelman (Austria)
Morten Frost (Denmark)
Seiji Fukumoto (Japan)
Thomas Funck-Brentano (France)
Cayetano Galera (Spain)
Sonja Gamsjaeger (Austria)
Rudolf Gasser (Austria)
Kaare Gautvik (Norway)
Luigi Gennari (Italy)
Valerie Geoffroy (France)
Jeroen Geurts (Switzerland)
Fernando Gianfrancesco (Italy)
Laia Gifre (Spain)
Claus-Christian Glüer (Germany)
Froilán Granero-Moltó (Spain)
Alanna Green (Australia)
Jenny Gregory (United Kingdom)
Celia Gregson (United Kingdom)
Agi Grigoriadis (Canada)
Daniel Grinberg (Spain)
Bettina Groetsch (Germany)
Núria Guañabens (Spain)
Vito Guarnieri (Italy)
Peyman Hadji (Germany)
Melanie Haffner-Luntzer (Germany)
Morten Svarer Hansen (Denmark)
Barbara Hauser (United Kingdom)
Eric Hay (France)
Ursula Heilmeier (Germany)
Gretl Hendrickx (Belgium)
Eric Hesse (Germany)
Lorenz Hofbauer (Germany)
Wolfgang Högler (Austria)
Ingunn Holen (Norway)
Anita Ignatius (Germany)
Yuuki Imai (Japan)
Alex Ireland (United Kingdom)
Jelena Jadzic (Serbia)
Abbas Jafari (Denmark)
Katharina Jähn-Rickert (Germany)
Franz Jakob (Germany)

Hervé Kempf (France)
Franziska Knopf (Germany)
Uwe Kornak (Germany)
Martin Kuzma (Slovakia)
Laurence Legeai-Mallet (France)
Willem Lems (Netherlands)
Ulf Lerner (Sweden)
Thomas Levin Andersen (Denmark)
Mattias Lorentzon (Sweden)
Guillaume Mabilleau (France)
Christa Maes (Belgium)
Outi Mäkitie (Finland)
Polyzois Makras (Greece)
Radmila Matijevic (Serbia)
Antonio Maurizi (Italy)
Sandro Mazzaferro (Italy)
Carolina Medina-Gomez (Netherlands)
Christian Meier (Switzerland)
Ciro Menale (Italy)
Petar Milovanovic (Serbia)
Salvatore Minisola (Italy)
Barbara Misof (Austria)
Geert Mortier (Belgium)
Ralph Müller (Switzerland)
Anda Mihaela Naciu (Romania)
Amy Naylor (United Kingdom)
Barbara Obermayer-Pietsch (Austria)
Ling Oei (Netherlands)
Claes Ohlsson (Sweden)
Eric Orwoll (United States)
Julien Paccou (France)
Biagio Palmisano (Italy)
Eleftherios Paschalis (Austria)
Pilar Peris (Spain)
Olivier Peyruchaud (France)
Peter Pietschmann (Austria)
Richard Pikner (Czech Republic)
Adalbert Raimann (Austria)
Alexander Rauch (Germany)
Martina Rauner (Germany)
David Reid (United Kingdom)
Particia Reiner (Spain)
Lars Rejnmark (Denmark)
Mara Riminucci (Italy)
Ilaria Roato (Italy)
Pamela Gehron Robey (United States)
François Robin (France)
Daniel Roig (Spain)
Tim Rolvien (Germany)
Michela Rossi (Italy)
Katrine Hass Rubin (Denmark)

Nadia Rucci (Italy)
Kenneth Saag (United States)
Syazrah Salam (United Kingdom)
Arne Schaefer (Germany)
Camilla Schalin-Jantti (Finland)
Martin Schepelmann (Austria)
Thorsten Schinke (Germany)
Federica Scotto di Carlo (Italy)
Karan Shah (India)
Natalie Sims (Australia)
Iulia Simona Soare (Romania)
Cristina Sobacchi (Italy)
Kent Søe (Denmark)
Anne Sophie Sølling (Denmark)
Nicole Sommer (Austria)
Antonia Sophocleous (Cyprus)
Jakob Starup-Linde (Denmark)
Steve Stegen (Belgium)
Gudrun Stenbeck (Germany)
Nikola Štoković (Greece)
Pawel Szulc (France)
Gaia Tabacco (Italy)
Hanna Taipaleenmaki (Germany)
Josephine T. Tauer (Germany)
Michaela Tencerova (Slovakia)
Anna Maria Teti (Italy)
Jonathan Tobias (United Kingdom)
Drenka Trivanovic (Serbia)
Elena Tsourdi (Greece)
Jan Tuckermann (Germany)
Mustafa Unal (Turkey)
Maria Teresa Valenti (Italy)
Bram van der Eerden (Netherlands)
Marjolein Van Driel (Netherlands)
Wim Van Hul (Belgium)
Arnaud Vanjak (France)
Laura Ventura (Italy)
Christel Verollet (France)
Charlotte Verroken (Belgium)
Laurence Vico (France)
Anna Villa (Italy)
Felix Nikolai von Brackel (Germany)
Ling Wang (Switzerland)
Michael Whyte (United States)
Elizabeth Winter (Netherlands)
Eva Maria Wölfel (Germany)
Maria Yavropoulou (Greece)
Timur Yorgan (Germany)
Vit Zikan (Czech Republic)
Carola Zillikens (Netherlands)
Philippe Zysset (Switzerland)

**Poster number abbreviations:**

PLOxx – Plenary Oral Presentation
COPxx – Concurrent Oral Presentation
Pxx – Poster
NDxx – New Data Poster

*The information regarding author affiliations and countries included in this abstract book has been provided directly by the abstract submitters. The European Calcified Tissue Society (ECTS) does not verify or take responsibility for the accuracy, completeness, or correctness of this information.*

**Plenary Oral Presentations 1: Bone Biology and Rare Diseases**

**PLO01**

**Dynamics in Osteoclasts: A Single-Cell Perspective on Fusion and Fission**

*Valeriia Rezapova[1], Maria Materozzi[1], Camille Girolet[1], Maxim Artyomov[2], Abdelilah Wakkach[1], Claudine Blin-Wakkach[1]*
*[1]Université Côte d'Azur, Lp2m UMR7370, Nice, France*
*[2]Washington University School of Medicine, Department of Pathology and Immunology, St-Louis, United States*

**Abstract Text**

Osteoclasts (OCLs) are multinucleated bone-resorbing cells formed through precursor fusion and can also undergo fission into osteomorphs, recycling into new OCLs. We previously identified two major OCL subsets: highly resorbing CD200+ OCLs and inflammatory CD16/32+ (*Fcgr2b/3*+) OCLs. However, how fusion dynamics shape OCL phenotypes remains unclear.

To address this, we generated OCLs from bone marrow cells using M-CSF and RANKL, and performed scRNA-seq on sorted mononucleated cells (1N), 3–4-nuclei OCLs (3–4N), and 5–6-nuclei OCLs (5–6N). Unsupervised clustering identified seven clusters (C0-C6) whose proportion and transcriptional profiles changed with multinucleation.

All clusters, including 1N cells, expressed core OCL markers, indicating that key osteoclastic features emerge before fusion. Trajectory inference revealed two lineages originating from C2 (1N-enriched) progressing through C1, C3, and C4 (all *Fcgr2b/3+,* 3-4N-enriched) with immune-related signature ($p$-value<0.05), and converging on C0 (*CD200*+, 5-6N-enriched) strongly associated with bone resorption ($p$-value<0.05) but having lower immune capacities. The trajectory split into 2 multinucleated terminal states: C6, with higher resorption capacity and low mitochondrial content, and C5, with intermediate resorptive/immune features ($p$-value<0.05) and strong enrichment for osteomorph markers (mean z-score 4.67).

Flow cytometry confirmed dynamic marker expression: CD16/32 peaked in 3–4N OCLs, while CD200 peaked in 5–6N OCLs. In vitro, sorted CD16/32 OCLs differentiated into CD200+ OCLs, but the opposite was not observed, validating the inferred trajectory. Cells sorted on markers of C5 (Log2FC 14.183) showed reduced bone resorption capacity on resorption plate and multinucleation in microscopy.

These findings uncover previously unrecognized transition during OCL fusion. Early-fused OCLs enhance immune-related functions and switch toward resorption specialization as fusion progresses. Only part of OCLs appear to undergo osteomorph recycling, reactivating immune capacities at the expense of resorption. Deeper characterization of these subsets may support new therapeutic strategies for OCL-related diseases.

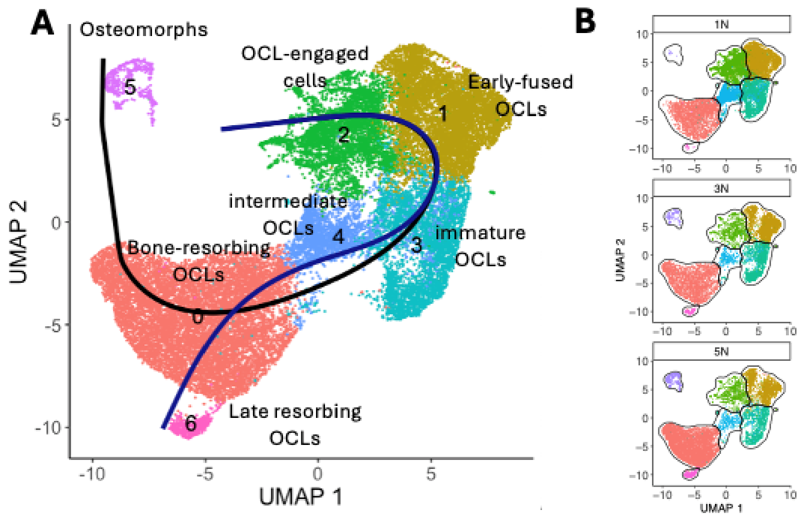

**Dynamics of OCL fusion revealed by scRNAseq analysis**
**(A)** Trajectory inference analysis, showing 2 lineages: lineage 1 (black line) with endpoint as C5, lineage 2 (red line) with endpoint C6. B) UMAP representing distribution of cells in clusters based on the number of nuclei

### PLO02

### Insulin resistance and changes in bone mineral density and cortical geometry in otherwise healthy young men: results of a longitudinal study.

*Steven Van Offel*[1], *Bruno Lapauw*[1], *Hans-Georg Zmierczak*[1], *Stefan Goemaere*[1], *Charlotte Verroken*[1]
[1]*Ghent University Hospital, Endocrinology, Ghent, Belgium*

### Abstract Text

**Background**: Type 2 diabetes (T2DM) increases fracture risk, but mechanisms are poorly understood. Because skeletal fragility results from many longstanding processes and insulin resistance (IR) is the precursor state of T2DM, many clinical studies have investigated associations between IR and parameters of bone health, although almost exclusively cross-sectional.

**Objective**: To assess whether IR associates with changes in bone mineral density (BMD) or cortical bone geometry over time in young healthy men.

**Methods:** In 999 young healthy men, areal BMD (aBMD) was measured at the femoral neck, total hip and lumbar spine using dual energy x-ray absorptiometry and volumetric BMD (vBMD) and cortical bone geometry at the radius and tibia diaphysis using peripheral quantitative computed tomography. After 11.5±1.9 years, these measurements were repeated in 709 participants. Longitudinal associations were investigated using multiple linear regression models, with annualized changes in bone parameters as outcome parameters.

**Results**: Baseline IR (baseline log-HOMA-IR) associated negatively with changes in periosteal and endosteal circumference (β=-0.178[-0.263- -0.094], $p<0.001$ and β=-0.148[-0.229- -0.066], $p<0.001$, respectively) at the radius after adjustment for baseline age, height, lean mass, fat mass and the baseline values of the respective outcome parameter. However, significance was lost if not baseline but exposure to IR (log transformation of the geometric mean of HOMA-IR at both visits) was used.

Exposure to IR was positively associated with change in aBMD at the femoral neck (β=0.121[0.040–0.202], p=0.004), total hip (β=0.155[0.071–0.239], $p<0.001$) and lumbar spine (β=0.135[0.043–0.227], p=0.004), after adjusting for baseline age, height, weight, the baseline value of the respective outcome parameter, and the annualized change in body weight. However, after adjusting for baseline and annualized change in lean and fat mass (instead of body weight), these relationships lost significance.

**Conclusion:** In young healthy men, IR does not associate with changes in bone parameters after adjustment for confounders.

**PLO03**

**Muscle density, but not appendicular lean mass, predicts fractures and falls and reveals distinct gut microbiome signatures in older women**

*<u>Marina Vilar Geraldi</u>[1], Chinmay Dwibedi[2], Victor Wåhlstrand[3], Jennifer Alvén[3], Ida Häggström[4], Kristian Axelsson[5], Lisa Johansson[5], Valentina Tremaroli[6], Fredrik Bäckhed[6], Mattias Lorentzon[7]*
*[1]University of Gothenburg, Department of Internal Medicine and Clinical Nutrition, Mölndal, Sweden*
*[2]The Wallenberg Laboratory, Department of Molecular and Clinical Medicine, Gothenburg, Sweden*
*[3]Chalmers University of Technology, Electrical Engineering, Gothenburg, Sweden*
*[4]Institute of Clinical Sciences, Electrical Engineering, Gothenburg, Sweden*
*[5]Sahlgrenska Osteoporosis Centre, Department of Internal Medicine and Clinical Nutrition, Gothenburg, Sweden*
*[6]The Wallenberg Laboratory, Molecular and Clinical Medicine, Gothenburg, Sweden*
*[7]Sahlgrenska Osteoporosis Centre, Internal Medicine and Clinical Nutrition, Gothenburg, Sweden*

**Abstract Text**

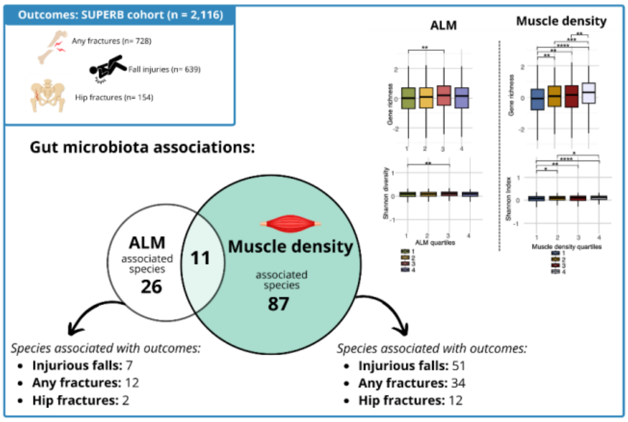

**Background:** Musculoskeletal mass is a recognized predictor of falls and fractures in older adults, and although the gut microbiota (GM) has been linked to lean mass, its relationships with specific components of muscle composition remain unclear.
**Purpose:** To (1) compare the ability of muscle density (MD) and appendicular lean mass (ALM) to predict incident fractures (any fracture, hip fracture) and fall injuries in community-dwelling older women; and (2) examine associations between GM profiles and measures of muscle composition.
**Methods:** We analyzed data from 2,116 community-dwelling women aged 75–80 years in the SUPERB cohort (median follow-up: 7.9 years). GM composition was profiled using shotgun metagenomic sequencing. Muscle composition assessments included proximal tibia MD measured by HR-pQCT and ALM assessed by DXA. Physical function was evaluated via walking speed, timed up-and-go, one-leg stance, and chair-stand tests. Associations were adjusted for age, fat mass, and height and reported as hazard ratios (HRs) per standard deviation (SD).
**Results:** Higher MD was associated with lower risk of injurious falls (HR 0.83, 95% CI 0.81–0.96; P<0.001), hip fractures (HR 0.75, 95% CI 0.64–0.88; P<0.001), and any fracture (HR 0.79, 95% CI 0.74–0.86; P<0.001) and was significantly associated with all physical function tests, whereas ALM was associated only with chair stand test. We identified 98 GM species significantly (Bonferroni-corrected) associated with MD and 37 associated with ALM, with only 11 species overlapping. Among these, 51 MD-associated species were related to injurious falls compared with 7 ALM-associated species. Shannon diversity and gene richness show robust and graded differences across MD quartiles, but inconsistent differences across ALM quartiles.
**Conclusion:** MD demonstrated stronger predictive value for fractures, injurious falls, and impaired physical function than ALM and showed greater ability to capture microbial signatures related to these outcomes in older women.

**PLO04**

**KOL17091, a First-in-Class Dual GIP/GLP-2 Analogue, Strongly Protects Against Skeletal Fragility in Type III Osteogenesis Imperfecta**

*Malory Couchot[1], Antoine Chretien[2], Catherine Behets[2], Guillaume Mabilleau[1]*
*[1]University of Angers, Inserm UMR_S 1229 RMeS, Angers, France*
*[2]UCLouvain, Pole of Morphology- Institute of Experimental and Clinical Research, Brussels, Belgium*

**Abstract Text**

**Background/Introduction:** Osteogenesis imperfecta (OI) is a rare genetic bone disorder marked by pronounced skeletal fragility and a substantial reduction in quality of life. To date, no pharmacological therapy has been approved to effectively address bone fragility in OI. Gut hormones, particularly glucose-dependent insulinotropic polypeptide (GIP) and glucagon-like peptide-2 (GLP-2), have recently emerged as promising candidates for improving bone material properties, suggesting a novel therapeutic avenue for managing skeletal fragility in OI.

**Purpose:** With the development of dual GIP/GLP-2 analogues, we hypothesized that KOL17091, the first-in-class molecule of this category, could offer an innovative strategy to counteract skeletal fragility in OI and tested it in the osteogenesis imperfecta mouse (oim), a model of type III OI.

**Methods:** Five-week-old female homozygous oim (n=30) were randomized to receive either saline or KOL17091 (25 nmol/kg/day). Only animals presenting a baseline fracture were included (n=13 OI+saline, n=9 OI+KOL17091). Wild-type littermates (n=10) served as controls. Mice were scanned in vivo at baseline, week 4, and endpoint (week 14) using NanoSPECT/CT$^{PLUS}$. Fractures were counted from reconstructed images. Bone microarchitecture, material properties, and biomechanics were assessed ex vivo using high-resolution micro-CT, Fourier-transform infrared imaging, and three-point bending, respectively, in animals with intact femora. Statistical analyses were performed using one-way ANOVA with Dunnett's post-hoc test ($p<0.05$).

**Results:** Compared with saline, KOL17091 administration reduced fracture incidence by 92% in OI mice. Bone microarchitectural parameters, including BV/TV (p=0.141), Tb.Th (p=0.564), Tb.N (p=0.144), and Ct.Th (p=0.623), remained unchanged. In contrast, collagen maturity, reflecting collagen matrix cross-linking, increased significantly in KOL17091-treated animals (+10%, $p<0.001$), which translated into enhanced biomechanical performance, as evidenced by higher bending stress (+20%, p=0.046).

**Conclusions:** Dual GIP/GLP-2 analogues targeting bone material properties represent a promising therapeutic alternative for reducing skeletal fragility in OI. Further investigation is warranted to determine whether these benefits translate to human patients.

**PLO05**

**Ex vivo expansion and lentiviral gene correction of circulating hematopoietic stem/progenitor cells to treat TCIRG1 autosomal recessive osteopetrosis: analysis at single cell resolution**

*Valentina Capo[1,2], Erika Zonari[1], Fabio Bedin[1], Valeria Iannello[1,3], Matteo Barcella[1], Alessandra Zecchillo[1,4], Elena Draghici[1], Beatrice Martini[1], Robert Chiesa[5], Alper Gezdirici[6], Fabrizio Benedicenti[1], Eugenio Montini[1], Ivan Merelli[1,7], Matteo Maria Naldini[1], Bernhard Gentner[1,8], Anna Villa[1,2]*
*[1]IRCCS San Raffaele Scientific Institute, San Raffaele Telethon Institute for Gene Therapy SR-Tiget, Milan, Italy*
*[2]Consiglio Nazionale delle Ricerche CNR,*
*Milan Unit- Institute of Genetic and Biomedical Research IRGB, Milan, Italy*
*[3]Vita-Salute San Raffaele University, PhD in Molecular Medicine, Milan, Italy*
*[4]University of Milano Bicocca, Translational and Molecular Medicine DIMET, Milan, Italy*
*[5]Great Ormond Street Hospital, Department of Bone Marrow Transplant, London, United Kingdom*
*[6]University of Health Sciences Basaksehir Cam and Sakura State Hospital,*
*Department of Medical Genetics, Istanbul, Turkey*
*[7]Consiglio Nazionale delle Ricerche CNR, Institute for Biomedical Technologies, Segrate, Italy*
*[8]University of Lausanne UNIL and Lausanne University Hospital CHUV,*
*Ludwig Institute for Cancer Research and Department of Oncology, Lausanne, Switzerland*

**Abstract Text**

**Introduction.** *TCIRG1*-autosomal recessive osteopetrosis (ARO) is a rare genetic disease characterized by defective osteoclasts, that leads to high bone density and increased circulation of CD34+ hematopoietic stem/progenitor cells (HSPCs).
**Purpose.** To design a safer and more effective treatment than standard hematopoietic stem cell (HSC) transplantation, we dissected the biology of ARO circulating HSPCs and their fitness to undergo *ex vivo* expansion and lentiviral correction in a large-scale, GMP-compliant protocol.
**Methods.** Unmanipulated or cultured CD34+ cells from 3 *TCIRG1* patients were analyzed by single-cell RNA sequencing, CITE-seq and xenotransplantation in NSG mice.
**Results.** We identified 17 clusters, including both primitive HSCs and committed progenitors. Unmanipulated ARO circulating CD34+ cells showed reduced content of HSCs/multipotent progenitors (30.5% vs 42.8%) and megakaryocyte/erythroid-biased progenitors (9.5% vs 19.6%) in favour of lympho/myeloid-primed (14.1% vs 5.6%) and B cell progenitors (7.6% vs 0.71%), when compared to cord blood healthy donors. Along the myeloid pseudotime trajectory, we observed lower expression of genes regulating HSC quiescence, coupled to higher adhesion and migration signals in ARO CD34+ cells than cord blood, indicative of CD34+ cell displacement from bone marrow niches into circulation. Following UM171 expansion and lentiviral correction with $10^8$ transducing units/ml of PGK.TCIRG1 vector, we observed the sustained presence of cells with an HSC and early progenitor signature, in line with the long-term engraftment and repopulation capacity in NSG mice (1.5%-33.4% human engraftment in BM). After expansion, we also observed the presence of CD34-CD33+ myeloid progenitors (20.8%-61.1%), that will contribute to the prompt differentiation of gene-corrected osteoclasts, as demonstrated by functional assays *in vitro*.
**Conclusion.** We optimized and tested a clinically-compliant gene therapy protocol based on lentiviral transduction and UM171 expansion, starting from circulating CD34+ cells of ARO patients, leading to the approval of a Phase I/II clinical trial for TCIRG1-osteopetrosis, open for recruitment from Q1 2026.

**PLO06**

**Palopegteriparatide Treatment Improves Skeletal Dynamics in Adults with Chronic Hypoparathyroidism: 214-Week Results from the Phase 2 PaTH Forward Trial**

*Elena Tsourdi[1], Mishaela Rubin[2], Aliya Khan[3], Peter Schwarz[4], Andrea Palermo[5], Filomena Cetani[6], Lynn Kohlmeier[7], Rajesh Jain[8], Bart Clarke[9], Carol Zhao[10], Michael Ominsky[11], Bryant Lai[11], Jenny Ukena[11], Christopher Sibley[11], Aimee Shu[11], Lars Rejnmark[12]*
*[1]Technische Universität Dresden, Department of Medicine III- Center for Healthy Aging, Dresden, Germany*
*[2]Columbia University, Endocrinology, New York, United States*
*[3]McMaster University, Division of Endocrinology and Geriatrics, Ontario, Canada*
*[4]Rigshospitalet, Department of Endocrinology, Copenhagen, Denmark*
*[5]Fondazione Policlinico Campus Bio-medico, Unit of Metabolic Bone and Thyroid Disorders, Rome, Italy*
*[6]University of Pisa, Endocrine Unit, Pisa, Italy*
*[7]Endocrinology and Spokane Osteoporosis, Endocrinology, Spokane, United States*
*[8]University of Chicago, Endocrinology- Diabetes and Metabolism, Chicago, United States*

*[9]Mayo Clinic, Endocrinology, Rochester, United States*
*[10]Ascendis Pharma Inc., Global Biometrics, Palo Alto, United States*
*[11]Ascendis Pharma Inc., Endocrine and Rare Disease Medical Sciences, Palo Alto, United States*
*[12]Aarhus University Hospital,*
*Department of Endocrinology and Internal Medicine- Department of Clinical Medicine, Aarhus, Denmark*

**Abstract Text**

**Background:** Hypoparathyroidism is associated with low rates of bone remodeling and increased bone mineral density (BMD) due to insufficient levels of parathyroid hormone (PTH). Palopegteriparatide (TransCon PTH), a prodrug of PTH (1-34) administered once daily, is designed to provide active PTH within the physiological range for 24 hours/day in adults with chronic hypoparathyroidism. Palopegteriparatide has received regulatory approval in the US, EU, and several other countries. This analysis investigated skeletal endpoints through week 214 of the PaTH Forward trial.

**Methods:** PaTH Forward was a phase 2, randomized, double-blind placebo-controlled 4-week trial followed by an open-label extension though week 266. Serum bone turnover markers procollagen type 1 N-terminal propeptide (P1NP) and C-terminal telopeptide of type 1 collagen (CTx) and BMD measured by DXA at the lumbar spine, total hip, femoral neck, and 1/3 distal radius were assessed.

**Results:** Of the 95% (N=56) of participants who continued to receive palopegteriparatide treatment through week 214, 93% were independent from conventional therapy (≤600 mg/day elemental calcium and no active vitamin D) and 98% had albumin-adjusted serum calcium levels in the normal range (8.3-10.6 mg/dL).

Mean CTx and P1NP were in the low end of normal at baseline, and increased with palopegteriparatide treatment, peaking at weeks 12 and 26, respectively. Thereafter, mean CTx and P1NP declined and remained stable and above baseline levels through week 214. The elevated baseline mean BMD Z-scores trended towards age- and sex-matched norms at the lumbar spine, femoral neck, and total hip and largely stabilized after 26 weeks of treatment, remaining above zero through week 214. No new safety signals were identified.

**Conclusions**: In adults with chronic hypoparathyroidism, continued treatment with palopegteriparatide through week 214 of PaTH Forward showed sustained skeletal remodeling with trends toward a new skeletal steady state closer to age-appropriate norms.

### Plenary Oral Presentations 2: Treatment and Fracture Healing

### PLO07

### BB-SEAL PROJECT: Fracture incidence, bone mass evolution, and bone turnover markers after denosumab discontinuation with sequential zoledronate treatment: A multicenter prospective study. 12-month analysis

*<u>Xavier Nogues</u>[1], Laia Gifre[2], Diana Ovejero[3], Helena Florez[4], Monegal Ana[4], Agueda Prior-Español[5],*

*Jade Soldado-Folgado[6], Nuria Guañabens[7], Natalia Garcia-Giralt[8], Pilar Peris[9]*
*[1]Hospital del Mar Research Institute.CIBERFES.Universitat Pompeu Fabra, Internal Medicine. Grup de recerca múscul-esqueletic, Barcelona, Spain*
*[2]Hospital Germans Trias i Pujol Universitat Autonoma de Barcelona, Rheumatology, Badalona, Spain*
*[3]Hospital del Mar Research Institute. CIBERFES, Grup de recerca múscul-esqueletic, Barcelona, Spain*
*[4]Hospital Clinic, Rheumatology, Barcelona, Spain*
*[5]Hospital Germans Trias i Pujol, Rheumatology, Badalona, Spain*
*[6]Hospital del Mar Research Institute. CIBERFES, Internal Medicine.Grup de recerca múscul-esqueletic., Barcelona, Spain*
*[7]Hospital Clinic. Universitat de Barcelona, Rheumatology, Barcelona, Spain*
*[8]Hospital del Mar Research Institute.CIBERFES.Universitat de Barcelona, Grup de recerca múscul-esqueletic., Barcelona, Spain*
*[9]Hospital Clinic.Universitat de Barcelona, Rheumatology, Barcelona, Spain*

**Abstract Text**

**Introduction:** Denosumab (Dmab) discontinuation leads to an accelerated loss of bone mass, which may be associated with vertebral fractures, even multiple. The ECTS has proposed the administration of bisphosphonates after Dmab withdrawal.

**Materials and Methods:** A prospective multicenter study (BB-SEAL Project) was designed to analyze, at 24 months, the incidence of new clinical and morphometric fractures, changes in BMD, and bone turnover markers (BTMs) in postmenopausal women and men discontinuing Dmab. Zoledronate (ZOL) 5 mg IV is administered with a baseline dose between the 5th and 6th month after the last Dmab injection, followed by additional doses at 6, 12, and 24 months after the baseline dose. Data collected include age, age at menopause, calcium intake, vitamin D levels, baseline BMD and every 6 months thereafter, BTMs at 3, 6, 12, and 24 months, and thoracic and lumbar spine radiographs at baseline and at 6, 12, and 24 months.

**Results:** Ninety-two patients were included: 85 women (mean age 67.8 ± 8.5 years) and 7 men (mean age 61.5 ± 14.7 years). Preliminary results are presented for 66 patients who reached 12 months of follow-up with ZOL (baseline dose and 6-month dose). No clinical or morphometric incidental fractures were detected. At 12 months, a mild loss of BMD was observed at all sites, significantly correlated with BTMs (CTX, P1NP, and bone alkaline phosphatase) at 6 and 12 months. A treatment duration with Dmab longer than 5 years is associated with a higher risk of experiencing a LS BMD loss greater than 3% at 12 months. BTMs increased significantly and remained elevated at 12 months (Table).

**Conclusions:** Sequential treatment with ZOL every 6 months prevents fractures and attenuates bone loss, although BTMs remain elevated, supporting the indication for a third dose of ZOL in all cases.

**PLO08**

**Rapamycin alleviates bone loss in ovariectomized mice – Insights from a study in mice with heterogeneous background**

Melina Guillon[1], Marc Petersen Harford[1], Ming Ding[2], Moustapha Kassem[1,3,4], Florence Figeac[1]
[1]Odense University Hospital & University of Southern Denmark,
Department of Molecular Endocrinology- KMEB, Odense, Denmark
[2]Odense University Hospital & University of Southern Denmark,
Department of Orthopaedic Surgery and Traumatology, Odense, Denmark
[3]Khalifa University, Faculty of Medicine and Health Sciences, Abu Dhabi, United Arab Emirates
[4]University of Copenhagen, Department of Cellular and Molecular Medicine, Copenhagen, Denmark

**Abstract Text**

**Background:** Aging is the main driver of skeletal fragility, yet preventive strategies are limited. Although rapamycin extends lifespan in mice and counters age-related decline, its specific effects on musculoskeletal health remain largely unknown.

**Purpose:** To investigate the effects of a rapamycin-enriched diet on bone phenotype after ovariectomy in genetically heterogeneous UM-HET3 mice.

**Methods**: Sixteen weeks old female UM-HET3 mice ovariectomized (OVX) or sham operated (SHAM) were fed either a control diet (ND) or a diet supplemented with 14 ppm rapamycin (RAPA) for 8 weeks. All experimental procedures were approved by the Danish Animal Ethical committee. Long bone microarchitecture and strength were assessed by micro–computed tomography and three-point bending mechanical testing, respectively. Histological analysis and RNA sequencing (RNA-seq) were performed on bone tissue. *In vitro* experiments were performed using human bone marrow mesenchymal stem cells (hTERT-MSCs) to examine osteogenic differentiation and primary human osteoclasts to assess bone-resorptive behavior following low-dose (1 nM) rapamycin treatment.

**Results**: Rapamycin diet markedly protected against OVX-induced bone loss (–19.1% in OVX-RAPA vs –36.4% in OVX-ND), primarily by preserving trabecular number (–2.0% vs –21.1%). It impaired glucose tolerance without altering β-cell mass, whereas OVX maintained normal glucose tolerance despite increased β-cell mass. RNA-seq further showed that rapamycin reversed the OVX signature, upregulating bone-formation pathways and suppressing OVX-driven inflammation. *In vitro* experiments showed that low-dose rapamycin (1 nM) boosted bone formation in hTERT-MSCs and decreased the bone-resorptive activity of primary human osteoclasts, at concentrations comparable to those achieved in treated mice.

**Conclusion:** Even though chronic rapamycin diet impaired glucose metabolism in our model, it preserved trabecular bone and reversed OVX-driven molecular signatures in genetically diverse UM-HET3 mice, a model with greater human relevance. Supporting *in vitro* data from human MSCs and osteoclasts further underscore its translational potential. Optimized dosing strategies may help retain skeletal benefits while limiting adverse effects.

**PLO09**

**The Number Needed to Treat (NNT) Analysis: A Clinical Context for the Treatment-Related Change in Total Hip Bone Mineral Density: the ASBMR-SABRE Project**

*Tatiane Vilaca*[1], *Li-Yung Lui*[2], *Marian Schini*[1], *Susan Ewing*[3], *Austin Thompson*[3], *Eric Vittinghoff*[3], *Douglas Bauer*[3,4], *Bente Langdahl*[5], *Mary Bouxsein*[6,7], *Dennis Black*[3], *Richard Eastell*[1]
[1]*University of Sheffield, Division of Clinical Medicine, Sheffield, United Kingdom*
[2]*California Pacific Medical Center, Research Institute, San Francisco, United States*
[3]*University of California, Department of Epidemiology & Biostatistics, San Francisco, United States*
[4]*University of California, Department of Medicine, San Francisco, United States*
[5]*Aarhus University Hospital, Department of Endocrinology, Aarhus, Denmark*
[6]*Beth Israel Deaconess Medical Center, Center for Advanced Orthopedic Studies, Boston, United States*
[7]*Harvard Medical School, Department of Orthopedic Surgery, Boston, United States*

**Abstract Text**

**Background:** The Study to Advance Bone Mineral Density as a Regulatory Endpoint (SABRE) has demonstrated that the treatment-related difference (active–placebo) in total hip bone mineral density percent change at 24 months (Δ%THBMD) is an excellent surrogate for fracture risk reduction.

**Purpose:** In this analysis, we estimated the clinical significance of Δ%THBMD by calculating the number needed to treat (NNT) to prevent one fracture over a three-year period, providing a direct measure of treatment benefit.

**Methods:** NNTs were derived from absolute risk reduction, defined as the difference in fracture incidence between treated and placebo groups. We calculated NNTs for vertebral and hip fractures for Δ%THBMD changes of 3% and 5%. Because NNT depends on background fracture incidence, we estimated 3-year fracture rates for each fracture type among placebo participants. We evaluated NNTs across the lowest, first quartile, median, third quartile, and highest incidence categories. Meta-regression curves were used to estimate relative risk as a function of Δ%THBMD change across these incidence levels.

**Results:** We analysed data from 25 randomised, placebo-controlled trials, comprising 137,717 participants receiving bisphosphonates (n=13 trials), odanacatib (n=1), hormone therapy (n=2), parathyroid hormone receptor agonists (n=3), denosumab (n=1), selective oestrogen receptor modulators (n=4), and romosozumab (n=1). NNTs decreased with higher fracture incidence and greater Δ%THBMD change. For a 3% increase in Δ%THBMD, NNTs for vertebral fractures ranged from 79 (2.8% incidence) to 7 (30.2% incidence) (panel A) and for hip fractures, from 1,408 (0.3% incidence) to 88 (4.8% incidence). At 5% Δ%THBMD, NNTs for vertebral fractures ranged from 56 to 5 and for hip, 757 to 47 respectively (panel B).

**Conclusion:** Integrating fracture risk reduction with NNT estimates based on treatment-related total hip BMD changes provides a clinically meaningful framework for quantifying treatment benefit in osteoporosis. This approach supports patient-centred interpretation of trial outcomes and may inform future trial design and regulatory evaluation.

**PLO10**

**Dlx5/6 inactivation in adult skeletal stem progenitor cells promotes bone hypertrophy and alters osteoblast cell fate**

*Morgane Bourmaud[1], Nicolas Narboux-Neme[2], Mylène Zarka[1], Camille Blandin[1], Sebastien Mella[3], Valentina Libri[3], Milena Hasan[3], Eric Hay[1], Giovanni Levi[2], Martine Cohen-Solal[1]*
*[1]Inserm U1132 and Universite Paris Cite, Hopital Lariboisiere, Paris, France*
*[2]Museum National d'Histoire Naturelle, Physiologie Moléculaire et Adaptation, Paris, France*
*[3]Institut Pasteur, Single Cell Biomarkers UTechS, Paris, France*

**Abstract Text**

**Purpose**: Bone homeostasis in adults relies on the coordinated activities of skeletal stem/progenitor cells (SSPCs). While the homeobox transcription factors *Dlx5* and *Dlx6* are established regulators of osteogenesis during development, their roles in adult bone maintenance remain unclear.

**Objectives:** We aimed to elucidate the postnatal function of *Dlx5/6* in bone homeostasis.

**Methods:** we analyze the bone phenotype in conditionally inactivated *Dlx5/6* in *Osx*-expressing SSPCs starting at 4 weeks of age (*Dlx5/6* $^{\Delta/\Delta\ Osx\text{-}cre}$) compared to Non-floxed *Osx-Cre* mice as controls (Ctl).

**Results:** Postnatal deletion of *Dlx5/6* resulted in marked femoral bone hypertrophy compared to Ctl, characterized by increased cross-sectional area (2.063±0.101 $mm^3$ vs 1.66±0.059 $mm^3$ p<0.05) and a decreased cortical thickness (140.4±3.186 µm vs 194.5±3.5 µm, p<0.001). Periosteal surfaces displayed increased bone formation (BFR: 0.5594±0.211 µm/day vs 0.1152±0.056 µm/day, p<0.05) with higher number of *Osx+* cells. Conversely, trabecular bone showed similar BV/TV (20.20±1.978% vs 16.74±1.221%, p: NS), but decrease bone formation (OS/BS: 10.33±1.257 % vs 28.60±1.986 %, p<0.01) and BFR (0.2427±0.02 µm/day vs 0.4253±0.04 µm/day, p<0.05). SSPCs cultures showed a lower expression of late osteoblast markers and impaired *ex vivo* osteoblast maturation (47.76±7.663 % vs 83.05±3.764 % mineralized nodules, p<0.01). Exploring the mechanisms of low bone formation, single-cell RNA sequencing revealed downregulation of *Sfrp1* in SSPCs. RNAscope confirmed a lower number of *Sfrp1* positive cells/$mm^2$ in bone marrow (1254±459.2 positive cells/$mm^2$ vs 10528±4638 positive cells/$mm^2$, p<0.01).

Conclusion: Lack of *Dlx5/6* in SSPCs promoted an expansion of the osteoprogenitor pool at the periosteum and trabecular bone, whilst reduced the terminal differentiation. These data revealed the essential role of *Dlx5/6* in balancing SSPCs commitment and osteoblast maturation in postnatal mature bones.

**PLO11**

**Effects of Bisphosphonates on Periprosthetic Bone Loss After Total Hip Arthroplasty**

*Changchuan Li[1], Wing Cheuk Ko[1], Yue Ding[1]*
*[1]Sun Yat-sen Memorial Hospital- Sun Yat-sen University, Orthopaedic Surgery, Guangzhou, China*

**Abstract Text**

**Introduction:** Periprosthetic bone loss is a major cause of implant loosening after total hip arthroplasty (THA). Bisphosphonates may mitigate postoperative loss, but comparative effects remain unclear.

**Purpose:** To assess the effect of alendronate in patients with normal preoperative bone mass undergoing THA and to compare zoledronic acid with alendronate in osteoporotic patients.

**Methods:** In this prospective randomized study, adults undergoing primary THA were allocated according to baseline bone status. Normal BMD (bone mineral density) patients received either no specific postoperative treatment (control) or alendronate (calcium-vitamin $D_3$ 600 mg/day plus alendronate-vitamin $D_3$ 70 mg/week). Osteoporotic patients received zoledronic acid (calcium-vitamin $D_3$ 600 mg/day, calcitriol 0.25 µg/day, and zoledronic acid 5 mg annually) or alendronate (calcium-vitamin $D_3$ 600 mg/day, calcitriol 0.25 µg/day, and alendronate-vitamin $D_3$ 70 mg/week). BMD of seven Gruen zones, spine, and hip was measured at day 7, month 3, month 12, and annually. Ethical approval and informed consent were obtained.

**Results:** A total of 202 patients were included (normal BMD control 42; normal BMD + alendronate 42; osteoporosis + zoledronic acid 62; osteoporosis + alendronate 56). In normal BMD patients, controls showed decreased total Gruen-zone BMD at 3 months (–4.82%, $p = 0.013$) and remained below baseline at years 2–3 (–5.23%, –5.10%). The alendronate group showed a smaller early decrease, and significant increases above baseline at 2 years (+4.47%, $p = 0.048$) and 3 years (+4.52%, $p = 0.047$). Among osteoporotic patients, alendronate caused approximately 5% BMD loss at 3 months, most prominent in zone 1 (–9.25%). Zoledronic acid produced consistent zone-1 BMD gains at years 1–3 (+11.40%, $p = 0.018$; +12.23%, $p = 0.013$; +12.83%, $p = 0.038$), significantly exceeding alendronate, except at 3 months (+4.74%, $p = 0.089$).

**Conclusion:** Alendronate preserves periprosthetic BMD in normal BMD patients after THA, whereas zoledronic acid provides superior protection in osteoporotic patients.

**PLO12**

**Interleukin-6 released from mast cells drives inflammatory responses and disrupts bone repair after trauma**

*<u>Verena Fischer</u>[1], Dorothea Gebauer[1], Jana Bleher[1], Sandra Dieterich[1], Melanie Haffner-Luntzer[1], Anne Dudeck[2], Anita Ignatius[1]*
*[1]Institute of Orthopedic Research and Biomechanics, Ulm University Medical Center, Ulm, Germany*
*[2]Institute of Molecular and Clinical Immunology, Otto-von-Guericke University Magdeburg, Magdeburg, Germany*

**Abstract Text**

**Introduction:** Multiply injured patients often experience impaired bone healing. Mast cells, tissue-resident innate immune cells, drive post-traumatic inflammation and delayed bone repair. Interleukin-6 (IL-6) is a major mediator of systemic inflammation after trauma linked to complications.

**Purpose:** Here, we investigated the role of mast cell-derived IL-6 in trauma-induced impaired bone repair.

**Methods:** Male mice (12-weeks) with mast cell-specific *IL-6* deletion and Cre-negative littermates underwent femur osteotomy, with half also receiving thoracic trauma (TxT) (No. 1518). Inflammatory responses were analyzed at 3 and 24h (circulating mediators, immune cells, lung inflammation, hepatic acute-phase response). At day 21 post-fracture, bone repair was assessed via biomechanical, µCT, and histomorphometric analysis (n=5-8/group, ANOVA/Fisher's LSD post-hoc-test, $p<0.05$).

**Results:** TxT controls showed elevated blood pro-inflammatory mediators (IL-6, CCL11, MCP-3, IL-12p70; all $p<0.05$), more severe lung injury (60% vs. 33%), and increased pulmonary neutrophils (p=0.01) at 3h, as well as increased hepatic *Cxcl-1* expression (p=0.03) at 24h compared to isolated fracture. Fracture hematoma immune populations were unchanged at 24h. At day 21, TxT controls displayed impaired bone repair with reduced bending stiffness (p=0.01), fewer bridged cortices, decreased bone (p=0.03) and increased cartilage (p=0.025) content, decreased osteoblast, and increased osteoclast activity. Notably, mast cell-specific *IL-6* deletion reduced trauma-induced systemic inflammation (IL-6, p=0.01; CCL-11, p=0.04) and lung injury/neutrophil infiltration (p=0.01) at 3h, reduced cytotoxic T cell numbers (p=0.01) in the fracture hematoma and the hepatic acute phase response (*Cxcl-1*, p=0.01) at 24h. These mice were protected against compromised bone repair at day 21, showing increased bone volume (1.0 *vs* 1.6 $mm^3$), enhanced cortical bridging (p=0.004), increased osteoblast, and reduced osteoclast parameters.

**Conclusion:** Mast cells are a critical source of post-traumatically increased IL-6, driving posttraumatic systemic inflammation and compromised bone repair after additional thoracic trauma. Targeting mast cells or their mediators may offer a therapeutic strategy to reduce fracture complications in multiply injured patients.

### Plenary Oral Presentations 3: Risk Factors and Pathophysiology

### PLO13

### Risk of Major Osteoporotic and Hip Fractures as a function of height: A Nationwide Case-Control Study

*Line Voss Karlsen*[1], *Bente Lomholt Langdahl*[1], *Torben Harsløf*[1], *Jakob Starup Linde*[1]
[1]*Aarhus University Hospital, Department of Endocrinology and Internal Medicine, Aarhus N, Denmark*

**Abstract Text**

**Background:** Osteoporosis is common, and fracture risk assessment is essential. Body height is associated with increased fracture risk but also increases areal bone mineral density (aBMD) as the increased depth of the bone seen with increased height increases bone mineral content.

**Purpose:** In this population-based case-control study, we examined the risk of major osteoporotic fractures (MOF) and hip fractures across 10-cm height categories.

**Methods:** We obtained dual-energy x-ray absorptiometry (DXA) data (2000-2019), diagnoses (1977-2019) and prescriptions (1997-2019) from the National Danish Registries. Data were linked through identifiers. Cases had their first MOF within two years before or one year after DXA. Controls had no MOF and were matched by age, gender, and DXA date. Height was analysed in 10-cm intervals with 150-160 cm as reference. Analyses were stratified by anti-osteoporotic treatment. Associations were analysed using multivariate logistic regression.

**Results:** We identified 8,130 cases and 8,130 controls in the non-bisphosphonate group and 2,990 cases and 2,990 controls among bisphosphonate (BP) users.

In individuals not using BP, height 170-180 cm and >180 cm were associated with increased MOF risk adjusted for total hip T-score (OR: 1.2, 95% CI: 1.1-1.3, p=0.001 and OR: 1.2, 95% CI: 1.0-1.5, p=0.016). No significant associations were observed among BP users.

For hip fractures, height >160 cm was associated with increased risk in non-BP users, most pronounced for height >180 cm (OR: 2.6, 95% CI: 1.9-3.5, p<0.001) adjusted for total hip T-score. Among BP-users, height >160 cm was also associated with increased hip-fracture risk, most pronounced for height >180 cm (OR: 2.2, 95% CI: 1.1:4.4, p=0.032) adjusted for total hip T-score. Findings were similar when adjusting for femoral neck and lumbar spine T-score.

**Conclusion:** Independently of BMD, MOF risk increased with height in non-BP users and hip-fracture risk increased with height independent of anti-osteoporotic treatment.

**PLO14**

**Proton pump inhibitors exposure, fracture and mortality risk in a nationwide matched cohort: results from the French OSIRIS study.**

*<u>Cécile Philippoteaux</u>[1], Chloé Saint-Dizier[2], Michaël Génin[3], Emmanuel Chazard[4], Antoine Lamer[2], Julien Paccou[1]*
*[1]Lille University Hospital, Rheumatology, Lille, France*
*[2]Lille University, Metrics ULR 2694, Lille, France*
*[3]Lille University Hospital, Statistics- Economic evaluation- Data management Seed, Lille, France*
*[4]Lille University Hospital, Public Health, Lille, France*

**Abstract Text**

**Background:** Proton pump inhibitors (PPI) are widely prescribed and often used long term. Although evidence suggests an association between PPI and fracture risk, findings remain inconsistent due to residual confounding and selected populations.

**Purpose**: This study aimed to investigate the relationship between PPI exposure, fracture and mortality risk at a population level.

**Methods:** OSIRIS is a retrospective cohort study using the French Nationwide Health Claims Database (SNDS). Adults aged ≥40 years newly exposed to PPIs for ≥6 months were propensity score-matched (1:1) with non-exposed individuals on age, sex, Charlson comorbidity index, major comorbidities, secondary osteoporosis etiologies, indications for PPI use, and commonly co-prescribed medications. Associations with major osteoporotic fractures were assessed using conditional logistic regression, while all-cause mortality was evaluated using Cox proportional hazards models.

**Results:** A total of 1,372,763 matched individuals were analyzed (mean age 65.6 ± 14.3 years; 50.0% women), with a mean follow-up of 8.75 ± 2.08 years. PPI exposure was associated with an increased fracture risk, including hip (OR 1.23 [CI 1.18-1.29]), vertebrae (OR 1.46; [1.36-1.56]), humerus (OR 1.19 [1.10-1.29]), and wrist (OR 1.20 [1.14-1.26]). Age-stratified analyses identified a non-linear pattern, with the greatest excess fracture risk in individuals aged 60–69 years (OR 1.58 [1.45-1.72]) (**Figure 1**). Overall fracture risk increased with longer treatment duration, particularly for hip (12 months OR 1.29 [1.22-1.36]; 24 months: OR 1.37 [1.29-1.47]) and vertebral fractures (VF) (12 months OR 1.49 [1.37–1.62]; 24 months: OR 1.56 [1.41-1.74]). PPI exposure was associated with an increased all-cause mortality (adjusted HR 1.24; 95% CI 1.21-1.28).

**Conclusion:** PPI exposure was associated with a consistent increase fracture risk from 6 months onward, with a greater risk for VF along with a significant higher mortality. Individuals aged 60-69 years presented the highest risk of fractures, identifying a critical window for fracture prevention and reassessment of long-term PPI use.

**Figure 1** - Age-stratified odds ratios for major osteoporotic fractures associated with proton pump inhibitor exposure (6 months).

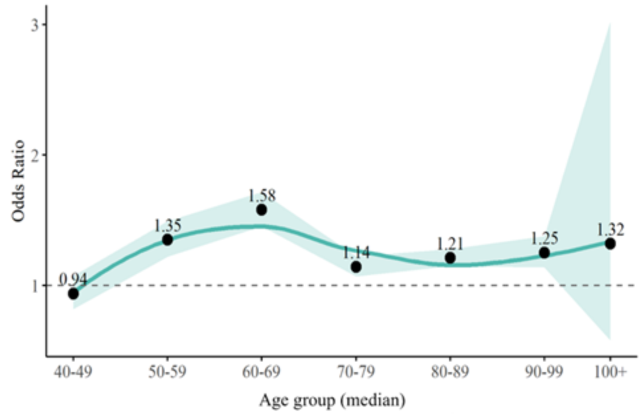

Odds ratios are shown by age group (median age). The shaded area represents the 95% confidence interval, and the dashed horizontal line indicates no association (OR = 1).

**PLO15**

**Sarcopenic-like myotubes derived extracellular vesicles mediates age-related dysfunctions in osteoblasts**

*<u>Laura Gerosa</u>[1], Chiara Verdelli[1], Amir Mohammad Malvandi[1], Maria Emanuela Orlando[1], Valerio Leoni[2], Giuseppe Banfi[1,3], Giovanni Lombardi[1,4]*
*[1]IRCCS Ospedale Galeazzi Sant'Ambrogio,*

*Laboratory of Experimental Biochemistry & Advanced Diagnostics, Milan, Italy*
[2]*University of Milano-Bicocca, Department of Laboratory Medicine, Desio, Italy*
[3]*Vita-Salute San Raffaele University, Faculty of Medicine and Surgery, Milan, Italy*
[4]*Poznan University of Physical Education, Department of Athletics- Strength and Conditioning, Poznan, Poland*

**Abstract Text**

Bone and skeletal muscle form an integrated unit connected not only mechanically but also through endocrine-like signals, meaning dysfunction in one tissue can affect the other. Aging impacts the musculoskeletal system, but the underlying mechanisms remain unclear.

This study investigated the molecular pathways through which aged skeletal muscle may impair bone cells' function, focusing on extracellular vesicles (EVs)-mediated communication. EVs play a key role in maintaining musculoskeletal homeostasis by transferring bioactive molecules, including lipids and miRNAs.

We used our recently established *in vitro* model of murine sarcopenic-like myotubes. Nanoparticle tracking analysis revealed that sarcopenic-EVs were less abundant ($6.25\times10^8 \pm 2{,}13x10^8$ vs. $4.66\times10^8 \pm 1{,}74\ x10^8$ particles/mL; $p= 0.0343$, unpaired t-test) and were larger in size (159.3±6.7 vs. 167.8±7.1 nm; $p= 0.0046$, unpaired t-test) compared to EVs derived from control cells. LC–MS lipidomics showed alterations in several lipid species (e.g., ceramide C16, lysophosphatidylcholine), while miRNA profiling indicated a significant downregulation of miR-27a-3p (1.000 vs. 0.315±0.203; $p< 0.0001$, unpaired t-test) and miR-206-3p (1.000 vs. 0.524±0.202; $p= 0.0034$, unpaired t-test).

We then explored the impact of these altered EVs on bone cells. Both pre-osteoblasts and mature osteoblasts internalized EVs derived from control and sarcopenic-like myotubes. However, sarcopenic-EVs more strongly impaired osteoblast alkaline phosphatase (ALP) activity and, to a lesser extent, matrix deposition. Long-term exposure, during pre-osteoblast differentiation, reduced ALP activity in derived osteoblasts by 48.7% (day 7) and 55.86% (day 14), while matrix deposition in mature osteoblasts decreased by 12%. Short-term exposure (24 h) of mature osteoblasts reduced ALP activity by 38%.

Ongoing experiments aim to clarify the molecular mechanisms driving these effects, assessing whether deregulated EV miRNAs, lipid alterations, or their combined action are responsible. Overall, our findings indicate that EVs from sarcopenic-like myotubes impair osteoblast function, underscoring EV-mediated muscle–bone communication as a contributor to age-related bone loss and a potential therapeutic target.

**PLO16**

**Association between glycated haemoglobin A1c and automated abdominal aortic calcification: Insights from the UK Biobank cohort**

*Haftom Abraha*[1,2], *Abadi Gebre*[1], *Cassandra Smith*[1,3], *Lakshini Herat*[4], *Afsah Saleem*[1,5], *Zulqarnain Gilani*[1,5,6], *Christian Girgis*[7,8], *Nicklas Rasmussen*[9], *William Leslie*[10], *John Schousboe*[11,12],

*Nicholas Harvey[13], Marc Sim[1,3], Joshua Lewis[1,3]*
*[1]Edith Cowan University, Nutrition and Health Innovation Research Institute, Perth, Australia*
*[2]Mekelle University, College of Health Sciences, Mekelle, Ethiopia*
*[3]University of Western Australia, Medical School, Perth, Australia*
*[4]University of Western Australia, School of Biomedical Sciences, Perth, Australia*
*[5]Edith Cowan University, School of Science, Perth, Australia*
*[6]University of Western Australia, Department of Computer Science and Software Engineering, Perth, Australia*
*[7]Westmead Hospital, Endocrinology Department, Sydney, Australia*
*[8]University of Sydney, Faculty of Medicine & Health, Sydney, Australia*
*[9]Aalborg University Hospital, Steno Diabetes Center North Denmark, Aalborg, Denmark*
*[10]University of Manitoba, Departments of Internal Medicine and Radiology, Winnipeg, Canada*
*[11]University of Minnesota, Departments of Internal Medicine and Radiology, Minneapolis, United States*
*[12]Park Nicollet Clinic and HealthPartners Institute, HealthPartners, Minneapolis, United States*
*[13]University of Southampton, The MRC Epidemiology Resource Centre, Southampton, United Kingdom*

**Abstract Text**

**Introduction:** Higher glycaemic levels, including presence of prediabetes and diabetes are associated with increased risk of clinical cardiovascular disease (CVD). However, little is known whether glycaemic levels are related to abdominal aortic calcification (AAC), a marker of subclinical CVD.

**Purpose:** To investigate the association between glycated haemoglobin (HbA1c) levels and moderate-to-high machine learned AAC24 scores (ML-AAC24 ≥2) among UK Biobank study participants.

**Methods:** This prospective study included 48,912 middle-aged to older adults without prior atherosclerotic CVD. HbA1c was measured at baseline (2006-2010) and ML-AAC24 scores were predicted using a validated automated algorithm applied to dual-energy X-ray lateral spine images (2014-2022). Participants were stratified according to HbA1c level as normal (<39.0 mmol/mol [<5.7%]), prediabetes (39.0–47.9 mmol/mol [5.7-6.4%]) and diabetes (≥48 mmol/mol [≥6.5%]) or according to self-reported/data-linked diabetes status. The association between HbA1c level and moderate-to-high ML-AAC24 was analysed using logistic regression adjusted for cardiovascular risk factors.

**Results:** Among 48,912 participants (mean ± SD age 55 ± 7.6 years; body mass index 26.6 ± 4.2 kg/m$^2$; 52% female), 9.7% had prediabetes and 3.1% had diabetes. Moderate-to-high ML-AAC24 prevalence increased across HbA1c categories: normal (19%), prediabetes (33%) and diabetes (39%). Each unit increase in HbA1c (%) was associated with 26% higher odds of moderate-to-high ML-AAC24 (aOR 1.26, 95%CI: 1.18-1.35) [**Figure 1**]. Compared to individuals with normal HbA1c level, those with prediabetes and diabetes had 19% (aOR 1.19, 95%CI: 1.08-1.30) and 64% (aOR 1.64, 95%CI: 1.39-1.94) higher odds of having moderate-to-high ML-AAC24, respectively. After excluding individuals with prediabetes and diabetes, similar results were observed per unit increase in HbA1c (%) (aOR 1.26, 95%CI: 1.11-1.44).

**Conclusion**: Graded associations between HbA1c levels and AAC burden were observed in UK adults. These results indicate that vascular calcification may develop early in the dysglycaemic continuum, supporting timely cardiometabolic risk assessment even amongst people with HbA1c below 5.7%.

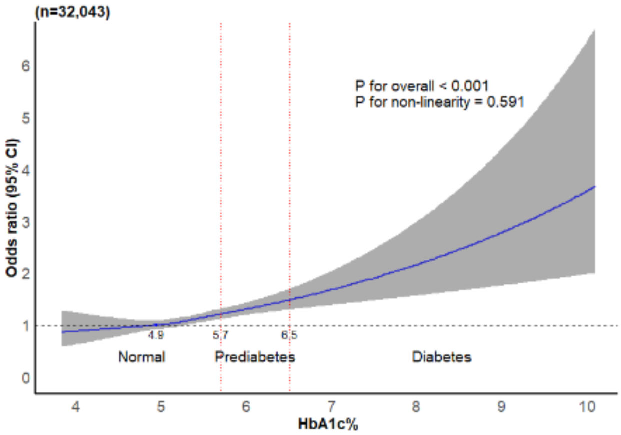

**Figure 1:** Cubic spline curves depicting the association between HbA1c% and moderate-to-high ML-AAC24 in participants of the UK biobank excluding people on antidiabetic medications with the median HbA1c for people in the lowest quartile (4.9%) serving as the reference.

**PLO17**

**Increased adipokine resistin in the bone niche in vivo: a novel link between aging, obesity and myeloma progression**

*Danielle J. Whipp[1], Claire M. Edwards[1], Magdalena Hutchins[1], Beatriz Gamez[1]*
*[1]University of Oxford, Nuffield Department of Surgical Sciences, Oxford, United Kingdom*

**Abstract Text**

Ageing and obesity are associated with negative changes in bone homeostasis, including increased adiposity. Multiple myeloma (MM) is a B cell malignancy where plasma cells invade the bone marrow (BM) leading to osteolytic bone lesions. Despite improvements in overall survival MM remains incurable.

We previously showed how C57Bl/KaLwRij on high-cholesterol diet had increased LDL (low-density lipoprotein) (2-fold, $p<0.05$) which led to higher BM tumour burden (2-fold, $p<0.01$) and increased osteolytic bone lesions ($p<0.01$).

We have identified the adipokine resistin as elevated specifically in the bone marrow plasma (BMP) in MM (>2 fold, $p<0.001$), with levels correlating with serum paraprotein ($R^2=0.5209$, $p<0.01$). Resistin was also increased in mice on a high cholesterol diet and aged mice ($p<0.05$). Resistin was not increased in serum, suggesting a specificity for the bone niche. In silico analysis of GSE47552 dataset showed increased BM resistin ($p<0.01$) in patients with the myeloma precursor condition MGUS (monoclonal gammopathy of undetermined significance), providing clinical evidence for a role in MM progression. In vitro, resistin was predominantly secreted from BM adipocytes (BMAds), secreting 50-fold more resistin than stromal and myeloma cells. LDL treated BMAds increased resistin expression (2-fold, $p<0.05$) and secretion (1.7-fold, $p<0.05$). Although myeloma cells expressed low resistin, co-culture with ST2 stromal cells increased their expression ($p<0.05$). Preliminary data showed resistin treatment promoted MM cell migration (2-fold).

Mechanistically, resistin induced gene expression of survival factors TNFa ($p<0.05$) and IL6 ($p<0.01$) and downregulated adiponectin receptors in MM cells ($p<0.01$). Resistin-treated BMAds downregulated adiponectin protien expression (western blot). As adiponectin has anti-myeloma activity, these results suggest that resistin may induce resistance to adiponectin pro-apoptotic signal in the BM niche.

We have identified resistin as dysregulated in myeloma and in myeloma-risk conditions as obesity and ageing, and as a potential mediator of disease progression due to its chemoattractant characteristics and through a novel resistin-adiponectin mechanism.

**PLO18**

**Immune Microenvironment of Non-Small Cell Lung Cancer Bone Metastases is "Cold" and Remodeled by Anti-PD-L1 Therapy in a Syngeneic Murine Model.**

*Emmanuel Massy*[1]*, Mathieu Point*[2]*, Lamia Bouazza*[3]*, Marie Brevet*[4]*, Nicolas Girard*[5]*, Jean Baptiste Pialat*[6]*, Philippe Clézardin*[3]*, Edith Bonnelye*[7]*, Cyrille Confavreux*[8]

[1]*Hospices Civils de Lyon, Rheumatology, PIERRE-BENITE, France*
[2]*CRCL, centre de recherche en cancérologie de lyon, Lyon, France*
[3]*U1033, lyos, lyon, France*
[4]*Cypath, Pathology, lyon, France*
[5]*institut curie, pneumology, paris, France*
[6]*Hospices Civils de Lyon, Radiology, lyon, France*
[7]*UMR9020-UMR1277-CANTHER, Université de Lille, Lille, France*
[8]*Hospices Civils de Lyon, Rheumatology, lyon, France*

**Abstract Text**

**Introduction**: Non-small cell lung cancer (NSCLC) is highly incident, and 40% of patients develop bone metastases (BM), which severely impact prognosis. Immune checkpoint inhibitors (ICIs) show reduced efficacy in patients with bone metastases, suggesting a unique immune microenvironment in the bone.

**Purpose:** We aimed to characterize the immunological differences between NSCLC primary tumors (PT) and BM, and to investigate the effects of anti-PD-L1 therapy on the immune cell populations within the bone microenvironment *in vivo*.

**Methods:** In a prospective human cohort (POUMOS, ethical protocol n°11-480), NSCLC PT and BM tissues were analyzed using multiplex immunohistochemistry to characterize CD8+ T cells and CD4+ subsets. In parallel, a syngeneic murine model was established using CMT167 cells to induce orthotopic BM. Mice were treated by anti-PD-L1 or control antibody. Immune responses were assessed by flow cytometry and bone metastasis by microCT.

**Results:** IHC analysis showed that NSCLC BM were "cold", characterized by a lower infiltration of CD8+ effector T cells (35.1±28.2 in PT vs 1.3±1.6 cells/mm² in BM, $p=0.0006$) and CD4+ Th1 lymphocytes (24.57±32.63 in PT vs. 0.37±0.50 cells/mm² in BM, $p=0.03$). Macrophages exhibited a predominant

M2 phenotype in BM with M1/M2 ratio 4.42±5.75 for PT vs. 0.21±0.27 in BM, p=0.03. Moreover, *in vivo* anti-PD-L1 treatment increased the proportion of CD4+ (1.7±0.3 fold change, p<0.05) and CD8+ effector T cells (2.1±0.4 fold change, p>0.05) in the bone microenvironment. MicroCT analysis revealed no difference in bone microarchitecture between anti-PD-L1 and control groups.

**Conclusion:** NSCLC bone metastases display a distinct, immunosuppressive microenvironment, notably lacking CD8 effectors and Th1 cells. Anti-PD-L1 therapy successfully induces an increase in effector T lymphocytes in the bone microenvironment *in vivo*, though this was not associated with changes in bone destruction. This suggests that ICIs effectively modulate local immunity, but their impact on bone structure may require combined therapeutic approaches.

### Concurrent Oral Presentations

### Concurrent Oral Presentations 1: Cell Biology in Bone Disease

**COP01**

### The Osteoprotective Role of Eosinophils in Bone Homeostasis and Inflammatory Bone Loss

*Pauline Porschitz*[1]*, Jule Lindörfer*[1]*, Mohammed Safhi*[2]*, Andreas Petzold*[3]*, Aline Bozec*[2]*,*
*Martina Rauner*[4]*, Darja Andreev*[1]
[1]*Technical University TU Dresden, Center for Regenerative Therapies Dresden CRTD, Dresden, Germany*
[2]*Friedrich-Alexander-University FAU Erlangen-Nürnberg and Universitätsklinikum Erlangen, Department of Internal Medicine 3 - Rheumatology and Immunology, Erlangen, Germany*
[3]*Center for Regenerative Therapies Dresen CRTD, Dresden-Concept Genome Center, Dresden, Germany*
[4]*University Medical Center- Technical University Dresden,*
*Division of Endocrinology- Diabetes and Bone Diseases- Department of Medicine III- and Center for He althy Aging, Dresden, Germany*

**Abstract Text**

**Background/Introduction:** Eosinophils are prominent bone marrow immune cells and key mediators of type 2 immunity, producing cytokines with potential anti-osteoclastogenic and pro-osteoblastogenic properties. Emerging evidence suggests eosinophils contribute to tissue homeostasis beyond classical immune functions, yet their role in bone homeostasis remains poorly understood.

**Purpose:** This study investigates the osteoprotective role of eosinophils in maintaining bone homeostasis under steady-state conditions and during inflammatory bone loss *in vitro* and *in vivo*.

**Methods:** Eosinophils were co-cultured with bone marrow-derived osteoclasts or MC3T3-E1 osteoblasts under steady-state and inflammatory conditions (IL-1β, IL-6/IL-6R, TNFα, or combined).

Bone marrow and long bone tissue from eosinophil-deficient ΔdblGATA mice were examined by immunofluorescence microscopy and single-cell RNA sequencing. Serum-induced arthritis (SIA) was induced in C57BL/6 male mice; bone marrow eosinophils were FACS-isolated and analyzed by single-cell RNA sequencing, metabolomics, and proteomics.

**Results:** Eosinophils significantly suppressed osteoclast formation and resorptive activity under steady-state conditions ($p < 0.0001$). Consistent with this, eosinophil-deficient ΔdblGATA mice showed elevated osteoclast precursor gene expression, while also exhibiting increased bone marrow adipocytes (2.1-fold Adipoq, $p < 0.05$), indicating that eosinophil loss promotes osteoclast development, while shifting mesenchymal progenitors toward adipocytes rather than osteoblasts.

*In vitro*, pro-inflammatory cytokines overcame eosinophil-mediated osteoclast suppression ($p<0.001$), while in osteoblasts, eosinophils counteracted the cytokine-induced reduction of the osteoblastogenic transcription factor Runx2 and the associated reduction of mineralization, restoring both to baseline levels ($p < 0.001$).

In SIA mice, bone marrow eosinophils showed a 35% increase in immune-activating subsets with elevated alarmins (S100a8/S100a9; 4.8-fold, $p<0.001$), increased secretion of anti-osteoclastogenic IL-33 and pro-osteoblastogenic Pentraxin-3, and altered TCA cycle metabolism, demonstrating metabolic and transcriptional reprogramming during inflammatory bone loss.

**Conclusion:** Eosinophils protect bone by suppressing osteoclastogenesis and promoting osteoblast-adipocyte lineage commitment toward bone formation. While inflammation can overwhelm eosinophil-mediated osteoclast suppression, eosinophils rescue osteoblast functionality and undergo metabolic and transcriptional reprogramming, suggesting adaptive osteoprotective mechanisms in inflammatory bone loss.

**COP02**

### Cystic Fibrosis-related Bone Disease: a preferential adipogenic differentiation impairs osteoblastogenesis

*Laurine Hamon[1], Claire Dumortier[1,2], Johan Sergheraert[3], Léa Thoraval[1], Sophie Gangloff[1], Antony Hoarau[2], Denise Al Alam[2], Julien Braux[3], Marie-Laure Jourdain[3], Frédéric Velard[1]*
*[1]Université de Reims Champagne-Ardenne, UR BIOS "Biomatériaux et Inflammation en site Osseux », Reims, France*
*[2]The Lundquist Institute, Harbor-UCLA Medical Center, Torrance, United States*
*[3]Université de Reims Champagne-Ardenne, CHU Reims- UR BIOS- Pôle de Médecine Bucco-Dentaire- UFR Odontologie, Reims, France*

### Abstract Text

Cystic Fibrosis-related Bone Disease (CFBD) affects up to 52% of adult CF patients. Osteoblasts obtained from F508delCFTR induced pluripotent stem cells evidenced an altered phenotype with a higher *PPARG* level (adipocyte transcription factor) as compared to non-CF [Dumortier, 2025]. A relationship exists between low bone density and increased medullar adiposity in several boneaffecting pathologies [Rozman, 1989; Verma, 2002; Zhou, 2008; Piccinin, 2014]. We hypothesized that adipocyte differentiation would be preferentially triggered in skeletal stem cells (SSCs), common precursor for both adipocytes and osteoblasts, when carrying *CFTR* mutations, which cause CF.

SSCs from non-CF donors have been differentiated into adipocytes and osteoblasts during 21 days, with or without CFTR inhibitors ($Inh_{172}$ or BPO-27) (n=7). Oil Red'O and Bodipy$^{TM}$ stainings, as well as FABP4 and PPARG immunostainings, were performed. MicroCT analysis was performed in sham operated or ovariectomized (OVX) CF female mice, and in both sexes within a one-year aging model. Osmium staining of decalcified bone was performed to evaluate bone marrow adiposity.

CFTR blockade induced lipid droplets formation in osteoblasts and SSCs cultures. In adipocyte cultures, it led to an increased number of adipocytes and in size of lipid vesicles (*p<0.05*) (**Figure**). CF OVX mice exhibited a markedly aggravated bone phenotype, characterized by decreased trabecular number and thickness, reduced bone mineral density and volume, and an increased trabecular separation, compared with non-CF OVX mice and wild-type littermates. In aged mice, male and female CF mice showed accelerated bone decline. Bone marrow adiposity is still under study, but preliminary data suggest elevated bone tissue adiposity in the CF genotype.

These results highlight a preferential differentiation of SSCs towards adipocytes when CFTR is blocked. Data indicate that aging in both sexes and postmenopause-induced osteoporosis accelerate bone disease in CF. Finally, the role of lipid metabolism may be pivotal to understand CFBD.

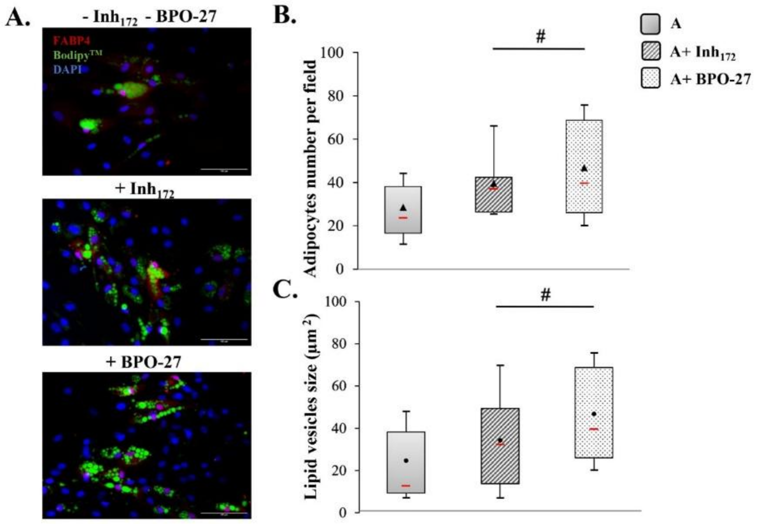

*Figure : CFTR inhibitor impact on adipocytes. Representative pictures of adipocytes at day 21 cultured with or without $Inh_{172}$ or BPO-27: FABP4 immunostaining (red), counterstained with DAPI (nucleus, blue) and bodipy$^{TM}$ (lipid vesicles, green)* (**A**). *Number of adipocytes at day 21 with $Inh_{172}$ (striped bar) or with BPO-27 (point bar) or without inhibitors (gray) per field* (**B**). *Lipid vesicle size in adipocytes with $Inh_{172}$ (striped bar) or with BPO-27 (point bar) or without inhibitors (gray)* (**C**). *# $p < 0.05$ compared to adipocytes without inhibitor.*

**COP03**

**Spatial transcriptional changes in bone cell populations and bone microstructure across age in ADO2 and WT mice**

*Xenia Goldberg Dahl[1,2,3], Anika Shimonty[2], Malene Hykkelbjerg Nielsen[1,3], Elissa Zboinsky[2], Julia*

*F. Charles[2], Thomas L. Andersen[1,3,4,5]*
*[1]University of Southern Denmark, Molecular Bone Histology Team- Dept. of Clinical Research, Odense, Denmark*
*[2]Brigham & Women's Hospital, Harvard Medical School, Boston, United States*
*[3]Odense University Hospital, Dept. of Pathology, Odense, Denmark*
*[4]Aarhus University, Dept. of Forensic Medicine, Odense, Denmark*
*[5]University of Southern Denmark, Danish Spatial Imaging Consortium DanSIC, Odense, Denmark*

**Abstract Text**

Autosomal dominant osteopetrosis type II (ADO2) is a rare genetic disorder caused by mutations in the *CLCN7* gene encoding a $Cl^-/H^+$ exchanger in osteoclasts. ADO2-patients have high bone mass with many (poorly resorbing) osteoclasts. We investigated the bone structure and cells of transgenic male mice with a *Clcn7*$^{F318L/+}$ heterozygous mutation mimicking a human mutation versus wildtype (WT) across aging.

We collected vertebrae at 12-weeks (*Clcn7*$^{F318L/+}$ n=13, WT n=12), 24-weeks (*Clcn7*$^{F318L/+}$ n=15, WT n=15), and 52-weeks (*Clcn7*$^{F318L/+}$ n=17, WT n=15). Bone samples were fixed and µCT-scanned before being decalcified and paraffin-embedded. Embedded samples from 12-weeks (*Clcn7*$^{F318L/+}$ n=3, WT n=4) and 52-weeks (*Clcn7*$^{F318L/+}$ n=4, WT n=3) were analyzed by GeoMx™ digital spatial profiling (DSP) using the Mouse Whole Transcriptome Atlas. ROI-selection in the GeoMx software was guided by a TRAcP/Osteopontin immunofluorescent staining to select ROIs of eroded surfaces with osteoclasts and osteoprogenitors, marrow cells, osteoblasts, and osteocytes.

As expected, *Clcn7*$^{F318L/+}$-mice had a significantly higher BV/TV than WT across ages. Furthermore, BV/TV of WT was significantly decreased at 52-weeks compared to both 12- and 24-weeks (p=0.02 and p=0.0007). Surprisingly, vertebral BV/TV of 52-weeks *Clcn7*$^{F318L/+}$ was maintained compared to both 12- and 24-weeks (p=1.0). GeoMx DSP analysis showed that aging not genotype was the main driver of transcriptional differences with components of senescence-associated secretory phenotype (SASP) changing with age in both genotypes. When comparing cell populations from 12- and 52-week mice a total of 126 differentially expressed transcripts were identified. Differentially expressed transcripts included *Cyfip2* and *Spp1* in osteoblasts, *Epha3*, *Mmp13*, and *Ddx51* on eroded surfaces, and *Vim* and *Tmsb4x* in osteocytes. However, each genotype displayed distinct transcriptional changes with aging. To validate this, more samples are being added to the GeoMx-experiments, but preliminary findings indicate that aging affects F318L mice differently than WT mice.

**COP04**

**Estrogen prevents anti-tumor effects of Zoledronic acid in breast cancer through the NK and CD8+T cell PD-1/PD-L1 axes**

*Jiabao Zhou[1], Christopher George[1], Victor Canuas-Landero[1], Diane Lefley[1], Russell Hughes[1], Fiona Thistletwaite[2], Caroline Wilson[3], Ingunn Holen[1], Mark Dunning[1], Sarah Danson[1], Matthew Winter[3], Lewis Quayle[4], Munitta Muthana[1], Penelope Ottewell[1]*
*[1]University of Sheffield, Division of Clinical Medicine, Sheffield, United Kingdom*

[2]The Christie NHS Foundation Trust, Experimental Cancer Medicine Team, Manchester, United Kingdom
[3]Sheffield Teaching Hospitals NHS Foundation Trust, Weston Park Hospital, Sheffield, United Kingdom
[4]Sheffield Hallam University, School of computing and Digital Technologies, Sheffield, United Kingdom

**Abstract Text**

**Background/Introduction:** Large clinical trials in breast cancer (including AZURE; involving 3360 patients) have demonstrated reduced recurrence in bone and soft tissue in postmenopausal women following adjuvant treatment with Zoledronic acid (Zol). In contrast, the same treatment resulted in increased risk of extra-skeletal recurrence in premenopausal women. The reason for these stark differences has remained unclear.

**Purpose:** To determine the mechanism by which oestrogen impedes the ability of Zol to elicit anti-tumour effects.

**Methods:** Mice were rendered pre- or post-menopausal by ovariectomy and subsequent administration of 0 or 12.5 mg/L 17β-estradiol before initiation of metastasis by intra-cardiac injection of E0771/4T1 cells. Effects on immune cells, immune checkpoints and tumour growth were determined by flow cytometry and IVIS imaging. Specific contributions of NK and CD8+T cells were assessed following antibody depletion. Effects of menopause on PD-L1 expression were determined on tumour samples from the AZURE dataset.

**Results:** Zol induced anti-tumor responses by increasing infiltration of CD8+T, dendritic and NK cells into soft tissue metastases, but only when estrogen concentrations were low. Anti-tumor response to Zol was CD8+T and NK cell-dependent in soft tissue, but only NK cell-dependent in bone. Conversely, estrogen significantly reduced systemic and tumor infiltrating CD8+ and CD4+T cells, increased PD-1 expression on T and NK cells and increased PD-L1 on tumor cells. Furthermore, PD-L1 expression was increased on tumours isolated from pre-menopausal patients and PD-L1 correlated with poor anti-tumour response to Zol ($p=0.0019$ distant recurrence). Combining Zol with PD-L1/PD-1 inhibition increases the anti-tumour effects of Zol in soft tissue and bone in high estrogen condition**.**

**Conclusion(s):** Estradiol reduces anti-cancer lymphoid cell infiltration into tumors and increases their exhaustion status, preventing Zol-induced activation of T and NK cells. Under premenopausal concentrations of estradiol, anti-tumor response to Zol can be restored by blocking PD-1/PD-L1 interactions, thereby overcoming estrogen driven immune suppression.

**COP05**

### A high fat diet exacerbates a VDR-deficiency induced bone phenotype

*<u>Kayleigh Rillaerts</u>[1], Lieve Verlinden[1], Stefanie Doms[1], Mieke Verstuyf[1], Iris Janssens[1]*
*[1]KULeuven, Chronic Disease and Metabolism, Leuven, Belgium*

**Abstract Text**

**Background:** Transcriptional regulation by the vitamin D receptor (VDR) is critical for calcium homeostasis and bone health, mainly via modulating intestinal and renal calcium (re)absorption. Dietary composition can additionally influence mineral balance and skeletal outcomes. This study examined how dietary calcium and high fat intake interact with VDR deficiency to affect mineral and bone homeostasis.

**Methods:** Male and female wildtype (*Vdr*$^{+/+}$) and systemic *Vdr* knockout (*Vdr*$^{-/-}$) mice were weaned onto a rescue diet (RD) or a high fat rescue diet (HFRD), both containing 2% calcium, 1.25% phosphate, and 20% lactose. 8-week-old mice were sacrificed to examine their calcium and bone phenotype.

**Results:** On the RD, both *Vdr*$^{+/+}$ and *Vdr*$^{-/-}$ mice remained normocalcemic, although male *Vdr*$^{-/-}$ mice displayed significantly lower serum calcium levels than their wildtype littermates (A). On the HFRD, serum calcium remained normal in wildtypes but were markedly reduced in *Vdr*$^{-/-}$ mice, with male *Vdr*$^{-/-}$ mice becoming hypocalcemic. The HFRD significantly decreased renal calcium excretion in both sexes, particularly in *Vdr*$^{-/-}$ mice (B). Duodenal expression of the calcium transporters *Trpv6* and *S100g* was significantly upregulated by the HFRD in wildtype mice with a similar trend observed in *Vdr*$^{-/-}$ mice. However, expression levels in *Vdr*$^{-/-}$ mice remained lower than in wildtypes on both diets (C, D). On the RD, female and male *Vdr*$^{-/-}$ mice showed a tendency toward reduced trabecular bone volume (E, G) and increased cortical porosity relative to wildtypes (F), and these differences became more pronounced on the HFRD. Conversely, the HFRD had minimal effects on the bone phenotype of wildtype mice.

**Conclusion:** VDR deficiency and increased dietary fat levels resulted in sex-specific effects on calcium absorption and bone integrity. These findings reveal distinct compensatory mechanisms between sexes and highlight the detrimental impact of high fat intake on skeletal health, especially when VDR signaling is absent.

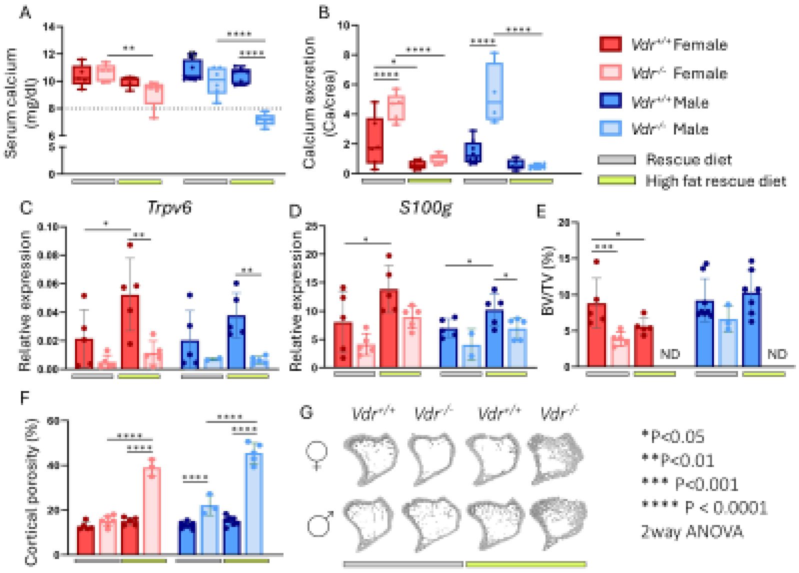

**COP06**

### Normal bone matrix mineralization, but reduced osteocyte canalicular density in patients with type 1 diabetes mellitus

*Varsha Margrette*[1], *Shannon Emerzian*[2,3], *Stéphane Blouin*[1], *Mary Bouxsein*[2,3], *Markus Hartmann*[1]
[1]*Ludwig Boltzmann Society, Ludwig Boltzmann Institute of Osteology, Vienna, Austria*
[2]*Beth Israel Deaconess Medical Center, Center for Advanced Orthopaedic Studies, Boston, United States*
[3]*Harvard Medical School, Harvard Medical School, Boston, United States*

**Abstract Text**

**Introduction:** Type 1 diabetes mellitus (T1D) patients have an increased fracture risk that cannot be fully explained by a reduction in bone mineral density (BMD). It is unclear which other factors contribute to the decrease in bone quality.

**Purpose:** To characterize bone matrix mineralization and osteocyte lacuno canalicular network properties in T1D patients.

**Methods:** Human cadaveric femoral neck samples were investigated from donors with T1D (9 males/11 females) and controls (8 males/10 females). Undecalcified samples were stained with rhodamine 6G and subsequently embedded in PMMA. Bone mineralization density distribution (BMDD), osteocyte lacunae section (OLS) and micropetrosis density were investigated with quantitative backscattered electron microscopy. The lacuno-canalicular network (LCN) density was assessed with laser scanning confocal microscopy. 2-Way-ANOVA with sex and +/-T1D as factors was used to analyze the data.

**Results:** No mineralization difference was observed between T1D and controls (mean and most frequent calcium content 25.12 wt.% Ca and 25.75 wt.% Ca) nor between sexes. A significant difference in mineralization was observed between the femoral neck and the iliac crest, the standard biopsy site (25.12 wt.% Ca and 22.96 wt.% Ca, $p<0.0001$). The OLS- and micropetrosis density was increased in females compared to males (OLS: 297 $1/mm^2$ and 264 $1/mm^2$, $p=0.009$; micropetrosis: 21 $1/mm^2$ and 13 $1/mm^2$, $p=0.009$), but similar between T1D and controls ($p=0.57$). The LCN-density was reduced in T1D compared to control samples (0.044 $\mu m/\mu m^3$ and 0.052 $\mu m/\mu m^3$, $p=0.048$), but similar between sexes ($p=0.24$).

**Conclusion:** These results are consistent with previous observations showing no mineralization difference in type 2 diabetes mellitus. Interestingly, females showed increased OLS-density and micropetrosis-density compared to males, independent of disease status, while a decrease in OLS-density was reported for women with type 2 diabetes mellitus. The LCN density is lower in T1D, indicating altered osteocyte function in this disease.

**Concurrent Oral Presentations 2: Determinants of Bone Homeostasis**

**COP07**

**PAR2 Modulates Energy Metabolism in Bone and Influences Bone Homeostasis.**

*Alexandros Lamprou*[1], *Patricia Riedlova*[1], *Carl Goodyear*[1], *Carmen Huesa*[1]
[1]*University of Glasgow, School of Infection and Immunity, Glasgow, United Kingdom*

**Abstract Text**

**Introduction:** Protease-activated receptor 2 (PAR2) is a G protein-coupled receptor activated by serine proteases and expressed in osteoblasts, osteoclasts, and bone marrow stromal cells. PAR2 has been implicated in bone formation and resorption via effects on proliferation, differentiation, and inflammatory signalling, but its broader regulatory mechanisms in bone remain unclear.

**Purpose:** This study aimed to define how PAR2 regulates bone homeostasis by integrating transcriptomic and functional analyses in murine and human osteogenic models.

**Results and Materials**: RNA sequencing was performed on long bones from 1-year-old wild-type and PAR2 knockout mice. Differential expression analysis (P.adj < 0.05, fold change) revealed two major gene clusters downregulated in the absence of PAR2: one enriched for metabolic processes, particularly lipid metabolism (Plin1, P.adj = 0.03; Scd1, P.adj = 0.003; Adipoq, P.adj = 0.0001), and another comprising extracellular matrix (ECM)-related genes, including multiple collagens and Cemip (P.adj = 0.03).

In vitro functional studies in murine MSCs and primary osteoblasts (pOBs), as well as human adipose-derived stem cells under mechanical stimulation, showed PAR2 gene expression peaked during early osteogenic differentiation. At this stage, Plin1 expression was significantly reduced ($p < 0.05$), consistent with in vivo findings, whereas Cemip was unexpectedly upregulated ($p < 0.05$). To validate metabolic findings, Seahorse analysis demonstrated that $PAR2^{-}/^{-}$ pOBs exhibited a significantly higher extracellular acidification rate (ECAR, P.adj = 0.049), indicating increased glycolysis. Pharmacological PAR2 inhibition in wild-type pOBs similarly increased ECAR, while PAR2 activation with 2-Furoyl-LIGRLO-amide reduced both oxygen consumption rate (OCR, P.adj = 0.037) and ECAR (P.adj = 0.035).

**Conclusion:** These data identify PAR2 as a metabolic brake in osteoblasts, tempering glycolytic flux and supporting lipid metabolic gene expression and ECM-related programmes. Loss or inhibition of PAR2 drives a shift toward glycolysis at the expense of lipid handling, with implications for osteoblast differentiation, matrix production, and bone homeostasis.

**COP09**

**SIRT5 regulates bone homeostasis via PINK1/Parkin mediated mitophagy and protects against senescence**

Ankita Paul[1], Devendra Pratap Singh[2], Saurabh Kumar Kaushal[2], Megha Dixit[2], Divya Singh[2]
[1]CSIR- Central Drug Research Institute, Endocrinology, Lucknow, India
[2]CSIR-Central Drug Research Institute, Endocrinology, Lucknow, India

**Abstract Text**

**Background:** SIRT5 is a mitochondrial sirtuin that regulates key enzymatic processes within the mitochondrial matrix and plays a crucial role in maintaining cellular redox homeostasis. While its protective functions have been well characterized in neurodegenerative diseases, cardiac stress, and cancer, the role of SIRT5 in the osteogenic niche remains largely unexplored.

**Purpose:** This study aimed to investigate the role of SIRT5 in osteoblast function and differentiation and to elucidate its contribution to mitochondrial integrity, oxidative stress regulation, and bone homeostasis.

**Methods:** SIRT5 expression was silenced in osteoblasts, with parallel pharmacological activation using resveratrol. Osteogenic differentiation, mitochondrial biogenesis, reactive oxygen species (ROS) production, mitophagy, and senescence markers were assessed using molecular, cellular, and biochemical approaches. In vivo validation was performed using SIRT5 knockdown in C57BL/6J mice, followed by analysis of bone microarchitecture and senescence-associated markers

**Results:** SIRT5 silencing significantly impaired osteogenesis and downregulated mitochondrial biogenesis markers, including TFAM, COX1, and PGC1α. Loss of SIRT5 resulted in elevated cellular and mitochondrial ROS levels and caused structural deterioration of mitochondria (TEM, ~60%, $P < 0.001$). Impaired mitophagy was evidenced by reduced expression of PINK1 (~80%; $P < 0.01$) and Parkin (~70%; $P < 0.05$, vs. Sc shRNA). Increased oxidative stress was associated with a marked upregulation of senescence markers p53, p21, and p16 in osteoblasts. In vivo, SIRT5 knockdown in C57BL/6J mice led to bone loss, compromised microarchitectural integrity (BV/TV, ~35%; Tb. Th, $P < 0.001$, Tb.N., ~50%; $P < 0.001$ reduction vs. AAV9-Control) and elevated senescence marker expression (p53, ~45%; $P < 0.001$, p21, ~24%; $P < 0.05$ vs. AAV9-Control).

**Conclusion(s):** These findings establish SIRT5 as a critical regulator of mitochondrial function, oxidative stress, and osteoblast homeostasis. Modulation of SIRT5 activity may represent a promising therapeutic strategy for preventing or treating osteoporosis by preserving mitochondrial integrity and limiting cellular senescence in bone.

**COP10**

**The Wnt1G177C/G177C mutation impairs fracture healing in mice**

Mubashir Ahmad[1], Humma Sarwar[1], Oliver Küppers[1], Jasmin Bülow[1], Christoph Kölbl[1], Sandra Dieterich[1], Melanie Haffner-Luntzer[1], Astrid Schoppa[1], Timur Alexander Yorgan[2], Michael Amling[2], Thorsten Schinke[2], Anita Ignatius[1]
[1]Institute of Orthopedic Research and Biomechanics, Ulm University, Ulm, Germany
[2]Department of Osteology and Biomechanics, University Medical Center Hamburg-Eppendorf, Hamburg, Germany

**Abstract Text**

**Introduction:** Wnt1 is essential for bone development and remodeling, with pathogenic variants linked to osteogenesis imperfecta type XV (OI-XV) and early-onset osteoporosis in humans and mice. However, the impact of Wnt1 variants on fracture healing remains unclear.
**Purpose:** This study investigates how the *Wnt1*$^{G177C/G177C}$ variant, associated with OI in humans, affects fracture repair, focusing on inflammation and bone formation.
**Methods:** A standardized femur osteotomy in 12-week-old female *Wnt1*$^{+/+}$ and *Wnt1*$^{G177C/G177C}$ mice, stabilized with an external fixator. Fracture healing was evaluated on days 2, 10, and 21 using three-point bending, microcomputed tomography (µCT), and histomorphometry. Early inflammation was analyzed using flow cytometry and a 36-plex cytokine/chemokine panel. Statistical analysis was performed using one-way ANOVA.
**Results:** *Wnt1*$^{G177C/G177C}$ mice exhibited significantly reduced mechanical strength (N.mm$^2$) in unfractured (2688.4 ± 486.7 vs 1660.3 ± 350.3, $P < 0.001$) and fractured femora (1358.3 ± 645.0 vs 439.3 ± 257.5, $P < 0.01$) compared with *Wnt1*$^{+/+}$ controls. µCT revealed decreased cortical thickness (µm) in unfractured femora (204.7 ± 13.1 vs 163.5 ± 9.5, $P < 0.001$) and reduced bone volume fraction (%) in the fracture callus (12.1 ± 3.9 vs 6.9 ± 2.8, $P < 0.05$) (Fig. 1A,B). Histomorphometry showed increased cartilage area (%) (14.1 ± 4.4 vs 22.0 ± 5.9, $P < 0.05$) and a trend toward reduced bone area (%) within the fracture callus (13.4 ± 3.4 vs 8.7 ± 4.5, $P = 0.06$) (Fig. 1C-E). Early inflammation was impaired in *Wnt1*$^{G177C/G177C}$ mice, evidenced by reduced macrophage numbers (Fig. 1F) and lower levels of chemokines CCL5 and CCL7.
**Conclusion:** The *Wnt1*$^{G177C/G177C}$ variant impairs fracture repair by disrupting bone formation and early inflammatory responses, both effective for healing. Targeting Wnt1-related signaling may offer a therapeutic strategy to improve fracture repair in Wnt1-associated bone disorders and other conditions with compromised healing.

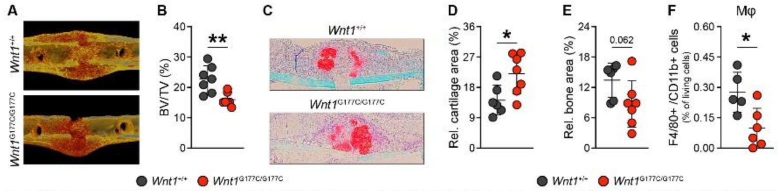

**Fig. 1: The *Wnt1*$^{G177C/G177C}$ mutation impairs fracture healing in mice. (A, B)** Representative µCT images of fracture calluses from *Wnt1*$^{+/+}$ and *Wnt1*$^{G177C/G177C}$ mice, with assessment of mineralized bone at day 21 post-femur fracture (n = 7 per group). **(C-E)** Safranin O staining of fracture calluses from *Wnt1*$^{+/+}$ and *Wnt1*$^{G177C/G177C}$ mice, with histomorphometric quantification of relative cartilage area and bone area at day 10 post-femur fracture (n = 6-7 per group). **(F)** Macrophage cell populations in the fracture hematoma, analyzed by flow cytometry from *Wnt1*$^{+/+}$ and *Wnt1*$^{G177C/G177C}$ mice at day 2 post-femur fracture (n = 7 per group). Data are presented as mean ± SD. $^*P < 0.05$, $^{**}P < 0.01$, one-way ANOVA test.

**COP11**

**Autotaxin is an autocrine inhibitor of parathyroid hormone-induced osteogenesis via inhibition of Wnt/β-Catenin pathway**

*Irma Machuca-Gayet[1], Mélanie Legrand[1], Gwénaëlle Jayat[2], Léa Demesmay[1], Candice Internicola[1], Lamia Bouazza[1], Candide Alioli[1], Agnès Linglart[3], Fabienne Coury[1], Justine Bacchetta[1], Sylvain Provot[2], Martine Cohen-Solal[2], Eric Hay[2], Olivier Peyruchaud[1]*
*[1]INSERM, Lyos/INSERM U.1033, Lyon, France*
*[2]INSERM, Bioscar/INSERM U.1132, Paris, France*

[3]*INSERM,*
*INSERM U.1185/Endocrinology and Diabetes for Children- Reference Center for Rare Diseases of the C alcium and Phosphate Metabolism, Paris, France*

**Abstract Text**

**Background:** Autotaxin (ATX, *Enpp2*) generates lysophosphatidic acid, a bioactive lipid regulating bone metabolism through cross-interaction between LPAR1 and LPAR4 receptors.

**Objective:** Characterize the role of ATX in osteoblast cell lineage, bone growth and PTH-mediating bone formation.

**Methods:** *Enpp2* expression (RT-qPCR) and ATX levels (Western-Blot/ELISA) were measured during osteoblast differentiation. We used bone of Col1-PPR mice expressing constitutively active PTHR1 under the Col1 promoter. We generated postnatal conditional *Enpp2* deletion in osteoblastic lineage (ObΔATX) using Osx-Cre mice. Bone phenotype was assessed in basal conditions and after intermittent PTH1-34 treatment (CT, histomorphometry and bulk RNAsequencing).

**Results:** *Enpp2* expression increased by 2-to-3-fold during differentiation of MC3T3-E1, MSCs and calvaria osteoblasts, being enhanced by 10-to-15-fold by forskolin, IBMX and PTH1-34. *Enpp2* expression was significantly higher in bone of Col1-PPR mice. Moreover, daily PTH1-34 treatment significantly increased circulating ATX levels in WT mice and hypoparathyroidism patients. Hence, ObΔATX mice showed no altered bone growth until 6 months of age. However, treatment with PTH1-34 for one week enhanced anabolic response in ObΔATX mice compared to control mice: BV/TV (40.3 vs 23.4 %), TbTh (68.6 vs 50.8 µm), MAR (4.6 vs 3.9 µm/day) and BFR (2.6 vs 1.6 cm3/µm2/day), all $p<0.001$.

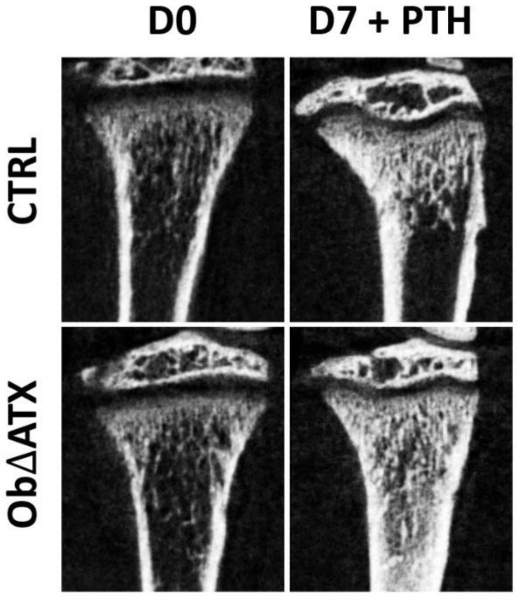

Bulk RNAseq of ObΔATX and control mice treated with PTH1-34 revealed upregulated bone osteoblast- (Bglap, Spp1, BMP4) and Wnt-related genes (TCF15, Wnt16, Fzrb) in ObΔATX mice. Moreover, genetic or pharmacological ATX inhibition in MSCs induced the nuclear translocation of b-catenin ($p<0.05$), the transcription of canonical Wnt-target genes (TCF7, LEF, C-Myc, Axin2) ($p<0.05$) and promoted osteogenesis as judged by higher alkaline phosphatase activity (2.3-fold) and mineralization (2-fold) after 7 days ($p<0.004$).

**Conclusion:** Our study revealed ATX as a new autocrine inhibitor of PTH1-34-induced osteogenesis by blocking the Wnt/β-catenin canonical pathway. These data provide insight to a better understanding of mechanism of bone response to PTH.

**COP12**

**The impact of gender-affirming therapy started before puberty on bone**

*Toke Liekens[1], Lieve Verlinden[1], Ingrid Stockmans[1], Karen Moermans[1], Dieter Schollaert[1], Vanessa Dubois[2], Steve Stegen[1]*
*[1]KU Leuven, chrometa, Leuven, Belgium*
*[2]UGent, Basic and Applied Medical Sciences, Gent, Belgium*

**Abstract Text**

**Introduction:** The number of young individuals seeking gender-affirming care is rapidly increasing. Hormone blockers are used during early puberty while awaiting future gender-affirming hormone treatment, leading to a relatively long period without sex hormone exposure. Long-term impact of these therapies on bone remains largely unknown.

**Hypothesis / methods:** We hypothesized that gender-affirming therapy, and especially early puberty suppression, causes skeletal cell dysfunction, which may not be fully restored by subsequent hormone supplementation, making bone more susceptible for future bone loss. To investigate this, a validated translational mouse model was used mimicking the clinical strategy applied in transboys, by treating 4-week-old female mice with the gonadotropin-releasing hormone antagonist Degarelix, followed by testosterone administration (Figure A).

**Results:** MicroCT analysis showed that, compared to native females and males, a 4-week Degarelix treatment caused a manifest decrease in trabecular bone volume (TBV) and cortical thickness (Ct.Th), which persisted and even further aggravated over time (Figure B, C). Subsequent testosterone administration restored the negative effects on TBV to female controls, but never reached the anticipated male levels. Moreover, compared to native females, Ct.Th remained decreased in testosterone-treated animals at all timepoints; only at the 16-week-timepoint native male levels were obtained (Figure B, C). Finally, we tested whether the effects of testosterone treatment resulted from androgen- or estrogen-receptor mediated signaling, by administering dihydrotestosterone (DHT) to Degarelix-treated mice (Figure A). At the 16-week timepoint, the Degarelix-induced bone phenotype was rescued by DHT to a similar extend as testosterone (Figure D, E), implying activation of androgen receptor signaling.

**Conclusion:** Our data indicates that early puberty suppression in female mice causes persisting adverse effects on bone that can only partially be reverted by testosterone treatment. These findings highlight the need to better understand the mechanisms by which gender-affirming therapies influence bone homeostasis and may lead to osteoporotic bone loss.

**Figure:**

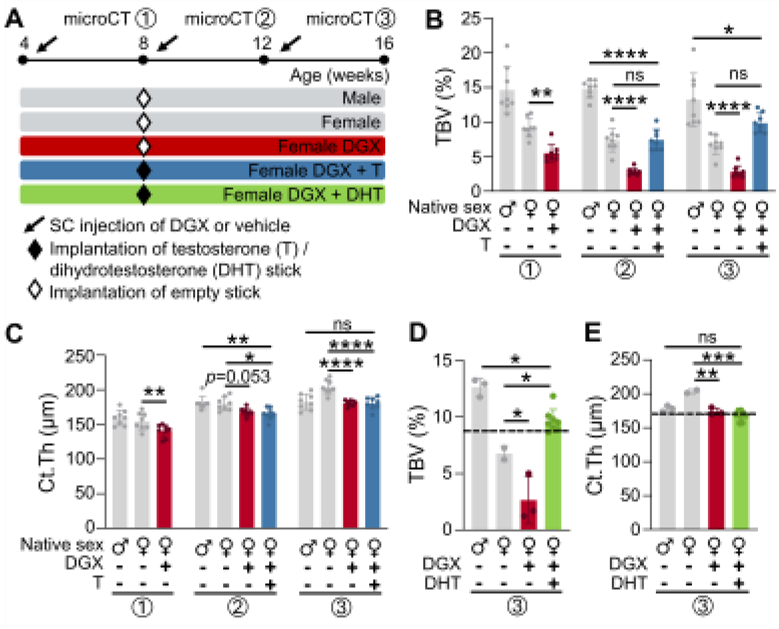

*p≤0.05, **p≤0.01, ***p≤0.001 ****p≤0.0001 (One-way ANOVA), ns: not significant. In figure D, E dotted line represents mean of DGX+T condition.

**Concurrent Oral Presentations 1: Rare Bone Diseases, Aortic Calcification**

**COP13**

**Fertility, pregnancy, labor, delivery, postpartum, and lactation in Italian women with X-linked hypophosphatemia: preliminary data from a survey-based multicenter study (the PPL-XLH study).**

*Silvia Carrara[1], Gaetano Paride Arcidiacono[2], Marco Barale[3], Giampiero Baroncelli[4], Maria Luisa Brandi[5,6], Valentina Camozzi[7], Filomena Cetani[8], Pasquale Comberiati[4,9], Simone Della Valentina[10], Lucia Digitale Selvaggio[11], Michela Ferrarese[7], Nadia Edvige Foligno[12], Sandro Giannini[2], Laura Gianotti[13], Anna Grandone[14], Gemma Marcucci[15], Laura Masi[16], Francesca Paglia[17], Daniela Pasquali[11], Francesca Pigliaru[18], Massimo Procopio[19], Vincenzo Rochira[17,20], Rosaria Maddalena Ruggeri[21], Elena Ruotolo[22], Noemi Sagone[19], Silvia Vai[1], Giuseppe Vezzoli[12], Sabrina Luigia Corbetta[1,23]*

*[1]IRCCS Istituto Auxologico Italiano,*
*Bone Metabolim Diseases and Diabetes Unit- Department of Endocrinology and Metabolic Diseases, Milan, Italy*
*[2]University of Padova, Clinica Medica 1- Department of Medicine, Padua, Italy*
*[3]University of Turin, Division of Oncological Endocrinology- Department of Medical Sciences, Turin, Italy*
*[4]University Hospital of Pisa,*
*Pediatric and Adolescent Endocrinology- Division of Pediatrics- Department of Obstetrics- Gynecology and Pediatrics, Pisa, Italy*
*[5]Villa Donatello Hospital, Donatello Bone Clinic, Florence, Italy*

*[6]F.I.R.M.O. Onlus Italian Foundation for the Research on Bone Diseases,*
*F.I.R.M.O. Onlus Italian Foundation for the Research on Bone Diseases, Florence, Italy*
*[7]University-Hospital of Padova, Endocrine Unit, Padua, Italy*
*[8]Department of Clinical and Experimental Medicine, University of Pisa- Endocrine Unit 2, Pisa, Italy*
*[9]University of Pisa, Department of Clinical and Experimental Medicine- Section of Paediatrics, Pisa, Italy*
*[10]University of Pisa, Department of Clinical and Experimental Medicine, Pisa, Italy*
*[11]University of Campania Luigi Vanvitelli, Department of Advanced Medical and Surgical Sciences, Naples, Italy*
*[12]IRCCS San Raffaele Scientific Institute, Nephrology and Dialysis Unit, Milan, Italy*
*[13]ASL Cuneo 1, Struttura Complessa di Endocrinologia e Diabetologia Territoriale, Cuneo, Italy*
*[14]Centre of Paediatric Endocrinology,*
*Department of Woman- Child and General and Specialised Surgery, Naples, Italy*
*[15]University Hospital of Florence,*
*Bone Metabolic Diseases Unit- Department of Experimental and Clinical Biomedical Sciences, Florence, Italy*
*[16]University Hospital of Florence, Metabolic Bone Diseases Unit, Florence, Italy*
*[17]Azienda Ospedaliero-Universitaria Policlinico di Modena- Ospedale Civile di Baggiovara,*
*Endocrinology Unit- Department of Medical Specialties, Modena, Italy*
*[18]AOU Cagliari, Endocrinology Unit, Cagliari, Italy*
*[19]University of Turin,*
*Division of Endocrinology- Diabetes and Metabolism- Department of Medical Sciences, Turin, Italy*
*[20]University of Modena and Reggio Emilia,*
*Unit of Endocrinology- Department of Biomedical- Metabolic and Neural Sciences, Modena, Italy*
*[21]University of Messina,*
*Dipartimento di Patologia Umana dell'Adulto e dell'Età Evolutiva "Gaetano Barresi"- Unità di Endocrin ologia, Messina, Italy*
*[22]University of Milan, Department of Medical Biotechnology and Translational Medicine, Milan, Italy*
*[23]University of Milan, Department of Biomedical- Surgical and Dental Sciences, Milan, Italy*

**Abstract Text**

**Introduction:** Recent recommendations from international panels of experts have proposed management of women with X-linked hypophosphatemia (XLH) during pregnancy and lactation.

**Purpose:** Investigating fertility, outcomes of pregnancy and post-partum in XLH women.

**Methods:** Survey-based study administered by bone specialists on September-November 2025.

**Results:** 63 XLH women aging 43.1±14.6 years answered the survey. Fertility All women experienced spontaneous menarche at 12.6±1.4 (10-16) years, 23.8% reporting oligo/amenorrhea. At the time of survey, 20 women were postmenopausal, with age at menopause ranging 44-57 (50.3±3.3) years. Pregnancy 36 women had 58 pregnancies, delivering 46 newborns, while 12 (20.7%) ended in miscarriages predominantly in the first trimester. Three women underwent 7 in vitro fertilization with embryo transfer with 3 newborns. Conception age ranged 19-41 (31.3±5.0) years. Only 36% of pregnant women received preconception genetic counseling. Though none suffered from pseudofractures/fractures while pregnant, 22% of women reported worsening of musculoskeletal symptoms. Fourteen pregnancies were complicated by hypothyroidism (n=5), kidney stones/infections (n=3), hypertension (n=3), gestational diabetes (n=1), anemia (n=1), carpal tunnel syndrome (n=1). Delivery 72.7% of women delivered by cesarian section (n=34), due to fetal podalic presentation (n=4), contracted maternal pelvis (n=10), or medical indications. Fifty-three percent of newborns were affected with XLH. Post-partum A fragility distal femur fracture occurred after miscarriage, one woman experienced multiple fractures, two women suffered dental abscesses. Lactation 77.8% of women breastfed their babies from 1 to 42 (9.1±10.8) months, three women reporting worsening of bone pain.

Conventional treatment before the age of 20 years is associated with early-term birth (37 vs 39 gestational weeks; p=0.0009). A minority of pregnant and lactating women were treated with conventional therapy and monitored by XLH experienced specialists.

**Conclusions:** Fertility is preserved in XLH women, while pregnancy and delivery need careful medical management due to complications. XLH women should be routinely referred to appropriate specialists.

**COP14**

**Clinical characteristics of patients with Fibrous Dysplasia/McCune Albright syndrome: an international, multicenter cohort study using the European Registries for Rare Endocrine and Bone Conditions**

*Sophie Huisman[1], Oana O Bulaicon[1], Roland Chapurlat[2], Deborah Gensburger[2], Mariya Cherenko[1], Corinna Grasemann[3], Violeta Iotova[4], M Kassim Javaid[5], Eva Kassi[6], Polyzois Makras[7], Stijn van der Meeren[8], Ondrej Soucek[9], Evert FS van Velsen[10], Annenienke C van de Ven[11], Charlotte Verroken[12], Pieter Bas de Witte[13], Maria Yavropoulou[14], Ana L Priego Zurita[1], Natasha M Appelman-Dijkstra[1]*

*[1]Leiden University Medical Center, Department of Internal Medicine- Division of Endocrinology, Leiden, Netherlands*

*[2]INSERM UMR 1033- Hôpital Edouard Herriot- Université Claude Bernard-Lyon 1, Service de Rhumatologie, Lyon, France*

*[3]Ruhr-University Bochum, Department of Paediatrics-Katholisches Klinikum Bochum, Bochum, Germany*

*[4]Medical University of Varna- UMHAT "Sv. Marina", Department of Pediatrics, Varna, Bulgaria*

*[5]University of Oxford, Nuffield Department of Orthopaedics- Rheumatology and Musculoskeletal Sciences, Oxford, United Kingdom*

*[6]National University of Athens, Department of Biological Chemistry- Medical School, Athens, Greece*

*[7]251 Hellenic Air Force & VA General Hospital, Department of Endocrinology & Diabetes- Department of Medical Research, Athens, Greece*

*[8]Leiden University Medical Center, Department of Ophthalmology, Leiden, Netherlands*

*[9]Charles University in Prague and Motol University Hospital, Department of Paediatrics, Prague, Czech Republic*

*[10]Erasmus Medical Center, Department of Internal Medicine, Rotterdam, Netherlands*

*[11]Radboud University Medical Center, Department of Internal Medicine- Division of Endocrinology,*

*Nijmegen, Netherlands*
*[12]Ghent University Hospital,*
*Department of Endocrinology- Unit for Osteoporosis and Metabolic Bone Diseases, Ghent, Netherlands*
*[13]Leiden University Medical Center, Department of Orthopaedics, Leiden, Netherlands*
*[14]National and Kapodistrian University of Athens,*
*First Department of Propaedeutic and Internal Medicine- Endocrinology Unit, Athens, Greece*

**Abstract Text**

**Introduction:** Fibrous dysplasia/McCune Albright (FD/MAS) syndrome is a rare, non-hereditary genetic bone disorder with limited large-scale data on clinical characteristics.

**Purpose:** This international, multicenter, cohort study aimed to provide a comprehensive overview of patient characteristics in a large FD/MAS cohort using data from the European Registries for Rare Endocrine and Bone conditions (EuRREB).

**Methods:** Between January 2023 and September 2025, detailed information was collected in a condition-specific module within the Core Registry of EuRREB, including 694 patients (638 adults, 56 children) from 8 different countries.

**Results:** Monostotic FD (MFD) was most prevalent in adults (49.0%), whereas MAS predominated in children (36.8%). The median (IQR) age at diagnosis was the highest in monostotic FD (28.7 (17.8-44.9) years) followed by polyostotic FD (PFD) (22.8 (13.5-35.4) years) and MAS (6.7 (2.9-14.2) years). Median (IQR) diagnostic delay was 1.3 (0.4-4.6) years with most delay occurring before first presentation at an expertise center. In MFD, craniofacial lesions were the most common (47.6%) whereas the lower extremity was the most often affected in PFD (67.6%). Among patients with craniofacial FD, 5 registered impaired vision (6.3%) and 1 (1.3%) patient had optic nerve compression.

At first presentation, all bone turnover markers (alkaline phosphatase, CTx and P1NP) were elevated in 46.2% of MAS, 21.3% of PFD and 4.5% of MFD patients. Multivariable analysis revealed that early age at diagnosis (OR 1.05, 95%CI 1.03-1.08), PFD (OR 2.59, 95%CI 1.41-4.76) and MAS (OR 3.02, 95%CI 1.34-6.76) were significantly associated with fractures before first presentation, whereas female sex (OR 2.57, 95%CI 1.56-4.25) and PFD (OR 2.43, 95% CI 1.42-4.17) were significantly associated with pain. Moreover, pain was more common for spine (83.3%) and rib lesions (77.8%) compared to craniofacial lesions (42.6%).

**Conclusion:** These results offer valuable insights into FD/MAS and can improve screening, diagnosing and therapeutic decision-making in clinical practice.

**COP15**

**Increased prevalence of intraductal papillary mucinous neoplasms of the pancreas in polyostotic fibrous dysplasia : TIMDYS study**

*Melanie Legrand[1], Deborah Gensburger[2], Romain L'Huillier[3], Flora Bagouet[2], Nadege Trehet-Mandel[2], Blandine Merle[2], Emmanuelle Vignot[2], Aurelie Fontana[2], Laurent Milot[3], Thomas Funck-Brentano[4],*

*Arnaud Vanjak[4], Nadia Mehsen-Cetre[5], thierry Marhadour[6], Sandrine Malochet-Guinamand[7], Bernard Cortet[8], Julien Paccou[8], Veronique Breuil[9], Guillaume Larid[10], Pascal Guggenbuhl[11], Adamah Amouzougan[12], Thierry Thomas[12], Rose-Marie Javier[13], Guillaume Couture[14], Chapurlat Roland[2]*
*[1]Geneva University Hospitals, Division of Bone Diseases- Department of Medicine-, Geneva, Switzerland*
*[2]Hospices Civils de Lyon- University of Lyon- INSERM UMR 1033, Department of Rheumatology- Edouard Herriot Hospital, Lyon, France*
*[3]Hospices Civils de Lyon- University of Lyon, Department of Medical Imaging- Edouard Herriot Hospital, Lyon, France*
*[4]Université Paris Cité- BIOSCAR INSERM U1132, Department of Rheumatology- Lariboisière Hospital, Paris, France*
*[5]Bordeaux University Hospital, Department of Rheumatology, Bordeaux, France*
*[6]Cavale-Blanche University Hospital, Department of Rheumatology, Brest, France*
*[7]Gabriel-Montpied University Hospital, Department of Rheumatology, Clermont-Ferrand, France*
*[8]Lille University Hospital- University of Lille- ULR 4490 MABLab, Department of Rheumatology, Lille, France*
*[9]Nice University Hospital- UMR 4320 PHEN-X- CEA–Université Côte d'Azur, Department of Rheumatology, Nice, France*
*[10]Poitiers University Hospital- University of Poitiers- LITEC, Department of Rheumatology, Poitiers, France*
*[11]Rennes University Hospital- University of Rennes- NUMECAN Institute- UMR 1317, Department of Rheumatology, Rennes, France*
*[12]Saint-Etienne University Hospital- Jean Monnet University- Inserm U1059, Department of Rheumatology, Saint-Étienne, France*
*[13]Strasbourg University Hospital- University of Strasbourg, Department of Rheumatology, Strasbourg, France*
*[14]Toulouse University Hospital- University of Toulouse, Department of Rheumatology, Toulouse, France*

**Abstract Text**

**Introduction**: Fibrous dysplasia (FD) of bone/McCune–Albright syndrome (MAS) is a rare disorder caused by activating mutations in GNAS gene, with oncogenic potential. GNAS mutations, frequently observed in intraductal papillary mucinous neoplasms (IPMNs) of the pancreas, have also been reported in patients with MAS. In line with the latest national guidelines, a pancreatic MRI screening program for IPMNs was implemented across the French FD/MAS reference centers.

**Purpose**: The study aimed to determine the prevalence and characteristics of IPMNs in patients with polyostotic FD.

**Methods**: In this retrospective cohort, adults with polyostotic FD who underwent pancreatic MRI as part of their clinical care were recruited from 13 French expert centers. Their data were extracted from the National Easily Hybrid DF database.  Pancreatic MRI findings were evaluated in a real-world cohort based on radiologist reports and compared with a centrally and blindly reviewed MRI series. Descriptive statistical analyses were performed using R.

**Results**: Among 202 adults with polyostotic FD (mean age 44 years), IPMNs were identified in 31 patients (15%), with only one pancreatic adenocarcinoma (0.5%) initially reported. After expert radiological review, IPMN prevalence increased to 29%. Patients with IPMN were significantly older than those without (53 vs. 42 years, p = 0.002). Most IPMNs were diagnosed after age 30 (90%) and more than one-third before age 50 (35%). Worrisome morphological features were more frequent in polyostotic FD patients with MAS than in those without MAS (60% vs. 14%, p = 0.02).

**Conclusion**: Polyostotic FD is associated with a markedly increased risk of IPMNs (up to 29%), compared with an estimated prevalence of ≈7% in the general population. These findings support the implementation of pancreatic MRI screening in FD patients from age 30 onward, ideally with images reviewed by expert radiologists.

**COP16**

**Cortical and trabecular bone changes in men with haemophilia: findings from The Haemophilia Osteoporosis Registry (THOR)**

*Ayse Zengin*[1]*, Vanessa Gan*[1]*, Huyen Tran*[2]*, Peter R Ebeling*[1]
[1]*Monash University, Department of Medicine- School of Clinical Sciences at Monash Health, Melbourne, Australia*
[2]*Alfred Hospital, Haemophilia Treatment Centre, Melbourne, Australia*

**Abstract Text**

**Background:** Haemophilia A is a rare X-linked bleeding disorder caused by factor VIII deficiency. Most people with haemophilia (PWH) experience haemarthrosis, leading to progressive joint damage and poorer bone health. Osteoporosis affects 27% of PWH, with a further 43% having osteopenia, and fragility fracture risk is 4.4-fold higher than in age- and sex-matched controls.

**Purpose:** To investigate differences in trabecular and cortical bone variables at the radius and tibia in PWH compared with controls over one year.

**Methods:** Eighty-seven men (76 controls, 11 PWH; ≥18 years) underwent HR-pQCT scans at baseline and one-year follow-up at the 4% and 30% radius and tibia. Outcomes included total volumetric bone mineral density (vBMD), cortical vBMD, trabecular vBMD, trabecular number, and cortical thickness. Mixed-effects models assessed whether changes over time differed between groups using a time×group interaction term, reporting β-coefficients. Models were adjusted for age, weight, and height.

**Results:** After adjustment, PWH showed consistently, and substantially lower bone density and impaired microarchitecture compared with controls at both skeletal sites. At the radius, PWH had lower total vBMD ($\beta = -57.3$ mg/cm$^3$, $p < 0.001$), trabecular vBMD ($\beta = -47.8$ mg/cm$^3$, $p<0.001$), trabecular number ($\beta = -0.24$ mm$^{-1}$, $p<0.001$), and cortical thickness ($\beta = -0.30$ mm, $p = 0.026$). Similar or greater deficits were observed at the tibia, including reduced total vBMD ($\beta = -54.8$ mg/cm$^3$, $p<0.001$), trabecular vBMD ($\beta = -62.5$ mg/cm$^3$, $p<0.001$), trabecular number ($\beta = -0.40$ mm$^{-1}$, $p<0.001$), and cortical thickness ($\beta = -1.50$ mm, $p<0.001$). No significant changes over time were detected in either group. All time×group interaction terms were non-significant ($p>0.57$), indicating similar longitudinal trajectories.

**Conclusion:** PWH exhibit markedly lower volumetric bone density and microarchitectural integrity than controls, with large deficits across all HR-pQCT variables. Over one year, bone outcomes remained stable in both groups. These results underscore the need for routine bone health screening and targeted interventions for PWH.

**COP18**

**Automated abdominal aortic calcification is associated with physical frailty in the UK Biobank imaging study**

*Abadi Gebre*[1], *Marion Mundt*[1,2], *Elsa Dent*[3], *James Webster*[4], *Afsah Saleem*[1,5], *Syed Zulqarnain Gilani*[1,5,6], *Yuandan Zhang*[7], *Cassandra Smith*[1,8], *Parminder Raina*[9,10,11], *John P. Kemp*[7], *William D. Leslie*[12], *John T. Schousboe*[13,14], *Nicholas C. Harvey*[15,16], *Joshua R. Lewis*[1,8], *Marc Sim*[1,8]

[1]*Edith Cowan University, Nutrition & Health Innovation Research Institute, Joondalup, Australia*
[2]*The University of Western Australia, School of Human Sciences, Perth, Australia*
[3]*University of Waikato, Division of Health, Hamilton, New Zealand*
[4]*University of Oxford, Applied Health Research Unit- Nuffield Department of Population Health, Oxford, United Kingdom*
[5]*Edith Cowan University, Centre for Artificial Intelligence & Machine Learning- School of Science, Joondalup, Australia*
[6]*The University of Western Australia, Department of Computer Science and Software Engineering, Perth, Australia*
[7]*The University of Queensland- Translational Research Institute, Mater Research Institute, Brisbane, Australia*
[8]*The University of Western Australia, Medical School, Perth, Australia*
[9]*McMaster University, McMaster Institute for Research on Aging, Hamilton, Canada*
[10]*McMaster University, Labarge Centre for Mobility in Aging, Hamilton, Canada*
[11]*McMaster University, Department of Health Research Methods- Evidence- and Impact, Hamilton, Canada*
[12]*University of Manitoba, Department of Internal Medicine- Rady Faculty of Health Sciences, Winnipeg, Canada*
[13]*Health Partners, Nicollet Clinic and HealthPartners Institute, Minneapolis, United States*
[14]*University of Minnesota, Division of Health Policy and Management, Minneapolis, United States*
[15]*University of Southampton, MRC Lifecourse Epidemiology Centre, Southampton, United Kingdom*
[16]*University of Southampton and University Hospital Southampton NHS Foundation Trust, NIHR Southampton Biomedical Research Centre, Southampton, United Kingdom*

**Abstract Text**

**Background:** Clinical cardiovascular disease (CVD) contributes towards frailty. However, it remains unclear whether subclinical CVD e.g., abdominal aortic calcification (AAC), is associated with frailty.

**Purpose:** To investigate the cross-sectional association between AAC from DXA-derived lateral spine images and scored using a validated machine learning algorithm (ML-AAC24), and physical frailty in the UK Biobank Imaging Study.

**Methods:** 49,081 participants from the UK Biobank without a history of atherosclerotic CVD (ASCVD) were included. ML-AAC24 extent was categorised as low, moderate and high based on established categories. Physical frailty was based on a modified Fried's frailty phenotype, which comprises weak hand grip strength, slow walking speed, unintentional weight loss, exhaustion, and low physical activity. Individuals with three or more deficits were considered frail, while one or two deficits was considered pre-frail. Multivariable-adjusted logistic regression models were used to test the associations between ML-AAC24 extent and the odds of being pre-frail or frail.

**Results:** The participants' (52.5% women) mean age was 64.4 (SD 7.8) years. One in five individuals had moderate (15.7%) or high ML-AAC24 (4.7%). Compared to individuals with low ML-AAC24, those with moderate and high ML-AAC24 had greater odds of being pre-frail (ORs 1.06 95%CI 1.00-1.12 moderate; 1.14 95%CI 1.04-1.26 high) or frail (ORs 1.27 95%CI 1.12-1.44 moderate; 1.58 95%CI 1.31-1.91 high), adjusted for multiple covariates (Model 2). When stratified by sex, moderate and high ML-AAC24 were associated with increased odds of being frail both in women and men (**Table 1**).

**Conclusions:** When considering individuals without clinical ASCVD, those with moderate and high ML-AAC24 were more likely to present as pre-frail and frail. Given ML-AAC24 can be opportunistically identified during routine bone density testing, it could serve as a useful marker for the early detection of frailty.

**Table 1**: Multinominal logistic regression analysis for the cross-sectional association between extent of ML-AAC24 with pre-frailty and frailty in individuals with no history of atherosclerotic CVD.

| Frailty status | Model | Total (n= 49,081) | ML-AAC24 | | |
|---|---|---|---|---|---|
| | | | **Low** (n=39,071) | **Moderate** (n=7,697) | **High** (n=2,313) |
| Prevalence (%) | | 28,182 (57.4) | 22,344 (57.2) | 4,462 (58.0) | 1,376 (59.5) |
| Pre-frailty | Model 1 | All | Referent | **1.08 (1.02-1.14)** | **1.19 (1.08-1.31)** |
| | | Women only | Referent | **1.14 (1.05-1.24)** | **1.25 (1.08-1.45)** |
| | | Men only | Referent | 1.02 (0.95-1.10) | **1.15 (1.02-1.30)** |
| | Model 2 | All | Referent | **1.06 (1.00-1.12)** | **1.14 (1.04-1.26)** |
| | | Women only | Referent | **1.13 (1.04-1.22)** | **1.21 (1.05-1.41)** |
| | | Men only | Referent | 1.00 (0.92-1.07) | 1.09 (0.96-1.24) |
| Prevalence (%) | | 2,093 (4.3) | 1,510 (3.9) | 413 (5.4) | 170 (7.4) |
| Frailty | Model 1 | All | Referent | **1.36 (1.20-1.55)** | **1.81 (1.50-2.18)** |
| | | Women only | Referent | **1.39 (1.18-1.66)** | **1.97 (1.50-2.57)** |
| | | Men only | Referent | **1.34 (1.12-1.61)** | **1.68 (1.29-2.18)** |
| | Model 2 | All | Referent | **1.27 (1.12-1.44)** | **1.58 (1.31-1.91)** |
| | | Women only | Referent | **1.34 (1.13-1.59)** | **1.79 (1.36-2.35)** |
| | | Men only | Referent | **1.20 (1.00-1.45)** | **1.37 (1.05-1.79)** |

**Model 1** included age, sex and body mass index (BMI); **Model 2** included Model 1 plus prevalent diabetes, smoking, year of imaging, country of residence and deprivation. Bold numbers indicate significant difference compared to low ML-AAC24.

### Concurrent Oral Presentations 2: Fracture Risk and Treatment of Osteoporosis

### COP19

### Bone strain index improves 10-year fracture risk prediction beyond bone mineral density and FRAX® in community-dwelling older women: a study of the FRISBEE cohort

*Jeroen De Filette[1], Amélie Bellanger[2], Luca Rinaudo[3], Fabio Massimo Ulivieri[4], Ivan Calderone[3], Sofia Cuttone[3], Dolores Sanchez-Rodriguez[5], Felicia Baleanu[1], Anne-Sophie Hambye[6], Florence Benoit[5], Murielle Surquin[5], Laura Iconaru[1], Jean-Jacques Body[1], Pierre Bergmann[6]*

*[1]CHU Brugmann, Endocrinology, Brussels, Belgium*

*[2]CHU Brugmann, Translational Research Unit, Brussels, Belgium*
*[3]Tecnologie Avanzate T.A. SRL, Tecnologie Avanzate T.A. SRL, Turin, Italy*
*[4]Casa di Cura La Madonnina- Gruppo San Donato, Bone Metabolic Unit, Milan, Italy*
*[5]CHU Brugmann, Geriatrics, Brussels, Belgium*
*[6]CHU Brugmann, Nuclear Medicine, Brussels, Belgium*

**Abstract Text**

**Objectives:** The Bone Strain Index (BSI), derived from finite element analysis of DXA, quantifies mechanical strain within bone. Its predictive value for fragility fractures in older women remains to be established in large prospective studies.

**Methods:** This prospective cohort study assessed the association between BSI, incident and major osteoporotic fractures (MOF) in 3338 community-dwelling older women (age 64-77 years) during a 5-year and 10-year follow-up. BSI was calculated at the lumbar spine, total hip, and femoral neck using baseline DXA scans. Incident fragility and MOF were radiographically validated. Discriminative performance was assessed using ROC curves, optimal cut-offs (Youden index), and Cox proportional-hazards models adjusted for age, areal bone mineral density (aBMD), or FRAX® score.

**Results:** During the 10-year follow-up, 803 women (24.1%) sustained at least one fragility fracture, including 525 MOF. Higher BSI values were significantly associated with increased fracture risk at 5 and 10 years, independently of age, aBMD, or FRAX®. Total hip BSI showed the strongest performance among all sites (AUC 0.63 at 5 and 10 years), outperforming lumbar spine BSI (AUC 0.58). Each 1-SD increase in total hip BSI was associated with higher fracture risk (HR 1.42 [1.32–1.53] at 5 years; 1.41 [1.34–1.49] at 10 years), which remained significant after FRAX® adjustment (HR 1.24 [1.14–1.35]).

**Conclusions:** BSI independently predicted incident fragility fractures and MOF during a 5-year and 10-year follow-up in community-dwelling older women. Total hip BSI showed stronger predictive performance than lumbar spine BSI, and both provided discriminative value beyond aBMD and FRAX®.

**COP20**

**Fracture risk prediction using healthcare administrative data can discriminate cardiovascular risk in elderly individuals**

*Clément Vachey[1], Claudia Beaudoin[2], Sonia Jean[2], Claudia Blais[2], Suzanne Morin[3], Lynne Moore[4], Fabrice Mac-Way[1]*
*[1]CHU de Québec-Université Laval Research Center, Endocrinology-Nephrology Research Unit, Quebec City, Canada*
*[2]Institut National de la Santé Publique du Québec, Bureau d'information et d'études en santé des populations, Quebec City, Canada*
*[3]Research Institute of the McGill University Health Centre, Centre for Outcomes Research and Evaluation, Montreal, Canada*
*[4]CHU de Québec-Université Laval Research Center, Population Health and Optimal Health Practices Research Unit, Quebec City, Canada*

**Abstract Text**

**Background:** Tools have been validated to predict fracture risk using healthcare administrative data (HAD).

**Purpose:** The aim of this study was to assess the association between predicted fracture risk and the risk of major adverse cardiovascular events (MACE) and all-cause mortality using HAD.

**Methods:** We conducted a retrospective, population-based, cohort study including residents of the province of Quebec (Canada) aged 66 and older in 2011-2012, not treated with anti-osteoporotic medications in the previous year. We estimated fracture risk at baseline using a previously validated 5-year hip/femur fracture risk prediction tool. We defined two types of MACE: MACE-3 which included myocardial infarction, stroke and cardiovascular death, and MACE-6 which additionally included unstable angina, heart failure and aortic stenosis. Associations were estimated with survival analyses.

**Results:** 879,739 individuals were included: mean age±standard deviation 75.1±7.1 years, women 52.4%, median 5-year fracture risk 2.4% (interquartile range (IQR): 1.0-5.2%) in women vs 1.0% (IQR: 0.5-2.2%) in men. After a 5-year follow-up, 88,616 MACE-3, 221,929 MACE-6 and 160,829 deaths were observed. For all outcomes, crude incidence rates were significantly higher in men and increased linearly with fracture risk in both sexes (Figure 1 results on MACE-3). In Fine and Gray models, a 1% increase in predicted fracture risk was associated with a significant, age-adjusted, linear increase in cardiovascular risk. For MACE-3, subdistribution hazard ratios (sHR) were 1.076 (95% Confidence Interval (95%CI): 1.072-1.080) and 1.073 (95%CI: 1.067-1.079) in women and men, respectively. For MACE-6, sHR=1.107 (95%CI: 1.104-1.110) in women, and sHR=1.122 (95%CI: 1.118-1.126) in men.  For all-cause mortality, in age-adjusted Cox models, HR=1.164 (95%CI: 1.161-1.166) and HR=1.225 (95%CI: 1.221-1.229) in women and men, respectively.

**Conclusion:** Fracture risk prediction using information available in HAD is positively associated with MACE risk and all-cause mortality, pointing to its potential role in cardiovascular risk surveillance for public health purposes.

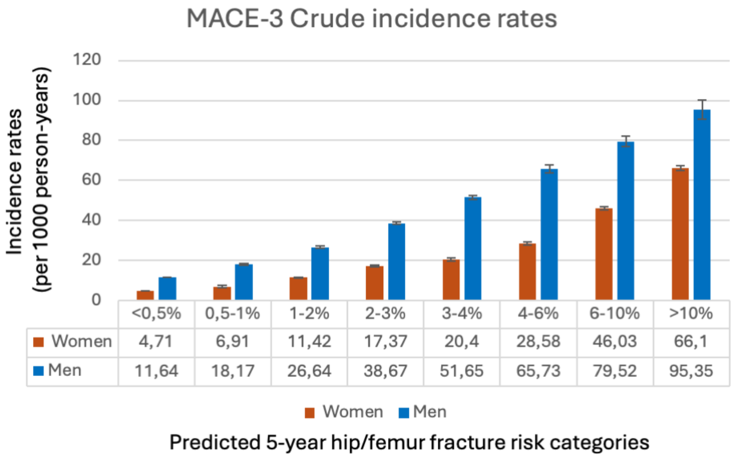

**COP21**

**Fracture-Derived Polygenic FRAX Reduces Misclassification and Improves Net Benefit Near Osteoporosis Treatment Thresholds**

*Qing Wu*[1], *Anqi Liu*[1]
[1]*The Ohio State University College of Medicine, Department of Biomedical Informatics, Columbus, United States*

**Abstract Text**

**Introduction:** FRAX guides osteoporosis treatment but lacks genome-wide genetic risk modeling, which contributes to gray-zone misclassification at treatment thresholds. We evaluated GPS-FRAX, a fracture GWAS–derived Bayesian polygenic score integrated with FRAX, to reduce misclassification and increase clinical net benefit at treatment thresholds.
**Purpose:** To test whether GPS-FRAX improves classification and clinical net benefit at guideline cut-points (NOF and NOGG) versus FRAX.
**Methods:** Genome-wide polygenic scores were derived from fracture-GWAS using PRS-CS, standardized, and integrated with FRAX. Internal validation included 6,932 postmenopausal women; external validation used an independent 2,330-participant hip-fracture cohort. The primary endpoint was net reclassification improvement (NRI) at the NOF 20% threshold and NOGG, focusing on a 15–25% gray-zone window. Secondary endpoints included AUROC, decision-curve analysis (DCA), and survival-aware 10-year performance.
**Results:** At the NOF 20% threshold, GPS-FRAX improved overall NRI by 4.8% (event NRI 6.2%). Among women reclassified at 20%, 23.3% moved from low to high risk and 13.0% from high to low risk, yielding ≈32 additional correct classifications per 1,000 women over 10 years. Crucially, within the 15–25% gray zone, 27% of women were reclassified, and 25% of fracture events moved from undertreatment to treatment. DCA demonstrated higher net benefit across the 15–20% intervention range (gain of 0.15/100 patients). Internally, AUROC improved from 0.66 to 0.70 ($p<0.01$); survival-aware 10-year AUROC rose from 0.73 to 0.75. External validation confirmed robustness with a positive overall NRI of ~3% and improved discrimination. Benefits were largest in women ≥70 years (NRI improved to 7.8%; AUROC improved from 0.52 to 0.61).
**Conclusions:** GPS-FRAX improves treatment-threshold decisions and net benefit where clinicians act, particularly for women in the gray zone, while maintaining survival-aware and external performance. Findings support genetics-informed, guideline-aligned fracture prevention, with the largest gains in women ≥70 years.

**COP22**

**Machine learning based estimation of the appendicular lean mass from hip and spine DXA scans in the UK Biobank Imaging Study**

*Marion Mundt*[1,2,3], *Afsah Saleem*[1,2], *Abadi K. Gebre*[1], *William D. Leslie*[4], *Namki Hong*[5], *Gustavo Duque*[6,7], *John T. Schousboe*[8,9], *Nicholas C. Harvey*[10,11], *Joshua R. Lewis*[1,12], *Marc Sim*[1,12]

[1]Edith Cowan University, Nutrition and Health Innovation Research Institute, Perth, Australia
[2]Edith Cowan University, Centre for Artificial Intelligence and Machine Learning, Perth, Australia
[3]The University of Western Australia, School of Human Sciences, Perth, Australia
[4]University of Manitoba, Department of Radiology, Winnipeg, Canada
[5]Yonsei University College of Medicine, Department of Internal Medicine, Seoul, Korea- South
[6]McGill University Health Centre, Bone- Muscle & Geroscience Group- Research Institute, Montreal, Canada
[7]McGill University, Department of Medicine, Montreal, Canada
[8]HealthPartners, Park Nicollet Clinic and HealthPartners Institute, Minneapolis, United States
[9]University of Minnesota, Division of Health Policy and Management, Minneapolis, United States
[10]University of Southampton, MRC Lifecourse Epidemiology Centre, Southampton, United Kingdom
[11]University of Southampton and University Hospital Southampton NHS Foundation Trust, NIHR Southampton Biomedical Research Centre, Southampton, United Kingdom
[12]The University of Western Australia, Medical School, Perth, Australia

**Abstract Text**

**Introduction:** Appendicular lean mass can be assessed on whole-body dual energy X-ray absorptiometry (DXA) scans and are used to define sarcopenia. Hip and anterior-posterior (AP) spine dual X-ray absorptiometry (DXA) scans are obtained to assess bone mineral density in more than 710,000 Australians annually. These scans also contain important "clues" or information about appendicular lean mass (ALM).

**Purpose:** We therefore applied a machine learning algorithm to hip and AP spine DXA scans to determine whether we could use these routinely captured images to estimate ALM.

**Methods:** Hip and AP spine DXA scans (GE Healthcare Lunar iDXA) and clinical data (sex, age, height, weight, BMI) of 33,938 individuals (51.7% female, age 65.2±7.8 years, height 170.1±9.4cm, weight 75.5±15.2kg, BMI 26.0±4.4kg/m$^2$) from the UK Biobank Imaging Study were included. We developed a custom three-branch machine learning model with hierarchical fusion gates to predict the ALM obtained from whole-body DXA scans. Models were trained using 5-fold cross validation with 60% scans used in training, 20% in validation and 20% in testing for a maximum of 50 epochs and optimised using mean-squared-error loss.

**Results:** The model achieved a mean absolute percentage error of 5.60% (MAE=1.160 kg, RMSE=1.512 kg). Applying established cut-off values for low ALM index (women<5.5kg/m$^2$, men<7.0kg/m$^2$), model accuracy was 89.1% and the Area Under Receiver Operating Characteristic curve (AUROC) was 89.9% (sensitivity 57.8%, specificity 93.0%, F1-score 53.8%, measured prevalence 11.0%, predicted prevalence 12.6%).

**Conclusion:** Our results highlight the feasibility and potential of applying a machine learning algorithm to hip and spine DXA to estimate low ALM with high specificity as part of routine osteoporosis screening. This overcomes the need for additional whole-body scans in those with low ALM and provides clinicians with new and important information on an individual’s risk of sarcopenia/osteosarcopenia.

**COP23**

**Characterisation of Bone Microstructure and Material Properties in Chronic Kidney Disease: A Case-Control Study**

*Praopilad Srisuwarn*[1], *Richard Eastell*[1], *Enrico Dall'Ara*[1,2], *Syazrah Salam*[1,3]
[1]*University of Sheffield, Clinical Medicine, Sheffield, United Kingdom*
[2]*University of Sheffield, INSIGNEO Institute, Sheffield, United Kingdom*
[3]*Sheffield Teaching Hospitals NHS Foundation Trust, Metabolic Bone Centre, Sheffield, United Kingdom*

**Abstract Text**

**Introduction:** Chronic kidney disease (CKD) is associated with an increased risk of fractures, which may be driven by alterations in bone quality beyond mineral density. Understanding the interplay between bone microarchitecture and mechanical properties in CKD is essential.

**Purpose:** To characterise bone microstructure and material properties in patients with advanced CKD compared to healthy controls.

**Methods:** In this case-control study, we recruited 10 kidney transplant candidates with CKD G5–5D and 10 healthy controls. Serum levels of parathyroid hormone (PTH) and bone turnover markers—bone-specific alkaline phosphatase (bone ALP), intact procollagen type I N-terminal propeptide (iPINP), and tartrate-resistant acid phosphatase 5b (TRAP5b)—were measured. Bone turnover phenotype was classified using serum bone ALP. Transiliac bone biopsies were analysed using micro-computed tomography, to assess bone microarchitecture, and nanoindentation, to evaluate local bone material properties. Results with p-values $< 0.05$ were considered statistically significant.

**Results:** CKD patients exhibited impaired bone microarchitecture, with a 15% lower trabecular thickness (mean difference, $p = 0.02$). Cortical bone was 72% thinner (median difference, $p < 0.01$) and showed a 40% reduction in cortical pore separation (median difference, $p = 0.02$). Age was negatively correlated with mean cortical thickness ($\rho = -0.72$, $p = 0.02$). CKD was independently associated with thinner cortices after adjusting for age ($\beta = -0.45$, adjusted $R^2 = 0.61$). Bone hardness and reduced modulus did not differ between CKD and control groups across inner and outer cortices or trabecular regions, and showed no correlation with age or bone turnover markers.

**Conclusion:** This is the first study to simultaneously assess bone microarchitecture and bone material properties in CKD. The findings confirm significant deterioration in both trabecular and cortical bone microstructure in CKD, which may contribute to increased fracture risk. In contrast, bone tissue material properties appeared minimally affected. Larger studies are needed to generalise these findings and explore their clinical implications.

**COP24**

**Bone turnover markers changes and BMD response to Romosozumab: insights from an Italian real-world cohort**

Alberto Ghielmetti[1], Antonio Stefano Salcuni[1], Francesca Marchese[1,2], Simona Lai[1], Alberto Falchetti[1], Fabio Vescini[1]
[1]University Hospital Santa Maria della Misericordia, Endocrinology Unit, Udine, Italy
[2]University of Trieste,
Department of Medical Surgical and Health Sciences- Cattinara Teaching Hospital, Trieste, Italy

**Abstract Text**

**Background:** Real-world evidence on Romosozumab effectiveness, especially in relation to bone turnover markers (BTMs) levels and prior osteoporosis treatment, remains limited.

**Purpose:** To evaluate changes in BTMs during Romosozumab therapy and their association with bone mineral density (BMD) gains after one year of treatment.

**Methods:** In all patients initiating Romosozumab, referred to the Endocrinology Unit, University Hospital Santa Maria Misericordia, Udine, Italy, we retrospectively evaluated BTMs at baseline and after 2, 6, and 12 months. BMD was assessed at baseline and after 12 months, in patients completing the treatment. Data on prior osteoporosis therapy were collected.

**Results:** 110 osteoporotic women (mean age 67 ± 6 years) were included. 46 (42%) were treatment-naïve, 27 (24%) had previously received teriparatide, 34 (31%) bisphosphonates and 3 (3%) denosumab. After 2 months mean ALP, bALP, P1NP levels increased, while CTX decreased compared to baseline ($p<0.001$ for all). At 12 months significant BMD gains were noticed (data available in 30 patients): lumbar spine (LS) 13.2 ± 8.3%, femoral neck (FN) 4.7 ± 5.3%, total hip (TH) 5.1 ± 4.8%, being LS gain higher in treatment-naïve patients vs. previously treated (18±10% vs. 11±6%, $p<0.05$) and 6 months mean P1NP levels higher in treatment-naïve vs. both antiresorptive and teriparatide groups ($p<0.05$ for all). Multivariate analysis revealed that LS BMD gains were directly associated with 6-month P1NP levels (standardized beta 0.43, $p<0.05$), while TH BMD gains correlated directly with baseline P1NP (standardized beta 0.61, $p<0.01$), regardless of baseline BMD at LS and TH, respectively.

**Conclusions:** Romosozumab significantly improves BMD and P1NP levels may help predict BMD gains. Irrespective of baseline BMD values, the more sustained elevation of P1NP at 6 months observed in treatment-naïve patients can justify their larger BMD response at LS, while higher baseline P1NP levels may drive a more pronounced BMD increase at TH.

### Poster Sessions

### Poster Sessions: Biomarkers and biochemical testing

**P001**

**Systematic review and meta-analysis of free (ionized) calcium estimation: albumin-adjustment versus total serum calcium as an optimal surrogate**

*<u>Ewout Verly</u>[1], Stephanie Desmedt[2]*
*[1]VITAZ, Endocrinology, Sint-Niklaas, Belgium*
*[2]AZ Delta, Endocrinology, Roeselare, Belgium*

**Abstract Text**

**Background:** Whilst approximately half of all circulating calcium is bound to serum albumin, only the ionized (free) fraction is biologically active. Measurement of free calcium is often not readily available due to technical, analytical, or financial limitations. Total calcium is frequently measured as a surrogate. Several authors propose that total serum calcium levels should be adjusted for albumin. The practice has led to disagreement in scientific literature as correction formulas were not validated in liver cirrhosis, end-stage kidney disease or intensive care. Over- or underdiagnosing calcium disorders might have severe health- and financial consequences with implications for general practitioners, specialists, and health services worldwide. Reported data is conflicting and a definitive conclusion is still lacking.

**Purpose:** evaluate whether albumin adjustment is superior to total calcium as a surrogate for biologically active (ionized) calcium based on accuracy measures.

**Methods:** Systematic review and meta-analysis. Protocol published on PROSPERO (CRD42023486132). 7.289 articles were screened, of which 36 were ultimately included. A detailed overview of the methodology can be found
here: https://www.crd.york.ac.uk/prospero/display_record.php?ID=CRD42023486132.

**Results:** the correlation (r) with free calcium is 0,60 for the BMJ formula, 0,46 for Berry, 0,27 for Clase, 0,51 for Orrell and 0,45 for Payne, compared to 0,60 for total calcium. The ICC for estimation of free calcium is 0,609 for BMJ, 0,754 for Orrell and 0,609 for Payne, compared to 0,720 for total calcium. Statistical tests and confidence intervals are lacking since these are unweighted results related to the limited number of studies for each parameter. 28 (77,8%) studies with 66.079 (84,1%) patients suggest that albumin adjustment has no benefit over total calcium as an estimate for the true (free) calcium status.

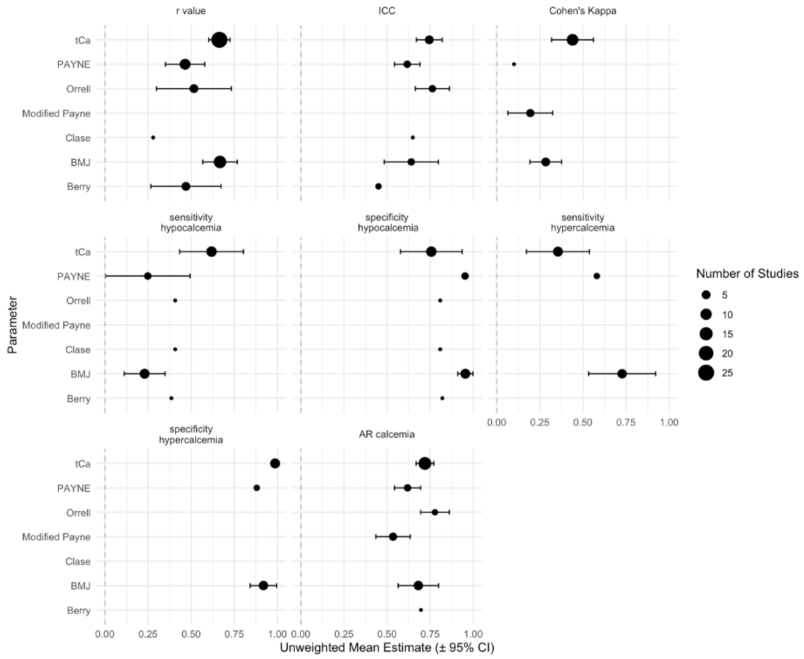

**Conclusion:** albumin adjustment does not seem to have a benefit over total calcium as an estimate for the true (free) calcium status based on our preliminary data-analysis.

**P002**

**Expected change: a new concept for monitoring patients on oral bisphosphonates**

*Marian Schini[1], Margaret Paggiosi[1], Fatma Gossiel[1], Steven Cummings[2], Eastell Richard[1]*
*[1]The University of Sheffield, Clinical Medicine, Sheffield, United Kingdom*
*[2]University of California, Epidemiology and Biostatistics, San Francisco, United States*

**Abstract Text**

**Introduction:** It is essential to closely monitor the response to oral bisphosphonate therapy for osteoporosis, as many patients are nonadherent. The conventional approach is to monitor whether changes in bone mineral density (BMD) or bone turnover markers (BTMs) exceed the least significant change. We propose an alternative approach, that of expected change. We define this expected change as the change in BMD (at 24 months) or BTMs (at three months) that is exceeded in 90% of patients who are compliant with oral bisphosphonate therapy.

**Methods:** We studied 108 postmenopausal women (age<85 years) who were randomised to the licensed dose of alendronate, ibandronate or risedronate treatment for two years, along with calcium and vitamin D supplementation. We identified the performance of BMD and BTMs in three ways.

**Results:** We calculated the signal-to-noise ratio, which was lower for BMD [4.1 and 2.1 for lumbar spine BMD (LSBMD) and total hip BMD (THBMD), respectively] compared to BTMs (9.4 and 10.2 for CTX and PINP, respectively). We estimated the response rate as the percentage of women exceeding the least significant change, which was lower for BMD (47% and 24% for LSBMD and THBMD, respectively) than for bone turnover markers (96% and 94% for CTX and PINP, respectively). We required the expected change to be greater than the least significant change, and this was not found for LS- and THBMD, but it was found for CTX and PINP.

**Conclusion:** Thus, the bone turnover markers CTX and PINP showed the best performance as response markers for monitoring oral bisphosphonate treatment, and the new approach is based on a biological rather than a statistical endpoint when using the expected change approach.

**P003**

**Role of CXCR4 in Teriparatide Response in Primary Human Osteoclasts and Reversal Cells**

*Mithra Sophie Gruber*[1], *Jacob Bastholm Olesen*[2,3], *Louise Hjorth Lind*[2], *Stuart H Ralston*[4], *Kent Søe*[2,3], *Nerea Alonso*[1]
[1]*Medical University of Graz, Clinical Institute for Medical and Chemical Laboratory Diagnostics, Graz, Austria*
[2]*University of Southern Denmark,*
*Clinical Cell Biology- Research Unit of Pathology- Department of Clinical Research, Odense, Denmark*
[3]*Odense University Hospital, Department of Pathology, Odense, Denmark*
[4]*University of Edinburgh, Rheumatic Disease Unit- Institute of Genetics and Cancer, Edinburgh, United Kingdom*

**Abstract Text**

Teriparatide, recombinant human parathyroid hormone 1-34 (PTH), is an anabolic drug for osteoporosis with variable patient response. Murine studies suggested *CXCR4* being associated with poor teriparatide response, by regulating osteoblasts and osteoclasts. We aim to validate the CXCR4 role in human teriparatide response. Osteoclasts were generated from CD14+ monocytes from blood donors (n=6) after MCSF and RANKL stimulation and seeded onto bovine bone slices. Reversal cells (RC) were obtained from hip arthroplasty donors (n=3) and cultured in differentiation medium (ethical approval number S-20110114). Resorption, alkaline phosphatase (ALP) and TRAcP activity in mono- and co-cultures without FBS and RANKL, and osteoclastogenesis in monocultures with FBS and RANKL were assessed, after treatment with 100µM CXCR4 inhibitor (AMD3100) and/or 50nM PTH. Paired t-test was performed in GraphPad Prism v8. PTH treatment showed large inter-individual variability in both cell types, increasing ALP activity in RC monocultures (n=2, Fold Change (FC)=1.12-1.26, p=0.01-0.06), whilst decreasing it by AMD3100-PTH combination (n=3, FC=0.58-0.86, p=0.0006–0.05). PTH-treated monocultures showed increased early osteoclastogenesis (n=2, FC=1.5-1.6, p=0.001-0.05) and decreased TRAcP activity during resorption (n=2, FC=0.78-0.79, p=0.002-0.004). PTH-induced resorption was donor-specific (n=6, FC=0.42-3.69, p=0.02-0.87). AMD3100-PTH combination decreased osteoclast size, number (n=5, FC=0.41-0.68, p=0.005-0.03), and resorption (n=4, FC=0.3-0.88, p=0.04-0.53), but increased TRAcP activity (n=5, FC=1.23-2.13, $p=6x10^{-7}$-0.03) compared to PTH. PTH-treated co-cultures showed no or opposite effects than monocultures in resorption and ALP activity, whilst AMD3100-PTH treatment increased ALP activity (n=2, FC=1.37-1.38, p=0.0006-0.005) and trended to promote the resorption phenotype of osteoclast monocultures (FC=0.98-0.82 in less resorptive osteoclasts and FC=1.14-1.38 in more resorptive ones, compared to

PTH). Our results suggest that PTH directly regulates osteoclastogenesis and function, steps prior to PTH-induced bone formation, via CXCR4. Direct and indirect PTH effects might activate compensatory mechanisms in co-cultures depending on underlying donor characteristics. Ultimately, PTH response is complex and donor-specific advocating for personalised approaches.

**P004**

**Proteomic and lipidomic profiling of femoral bone marrow plasma in comparison to peripheral blood**

*Niki Jalava*[1], *Alex Dickens*[2], *Otto Kauko*[2], *Matias Hemmilä*[3], *Keijo Mäkelä*[4], *Kaisa K. Ivaska*[1]
[1]*University of Turku, Institute of Biomedicine, Turku, Finland*
[2]*University of Turku and Åbo Akademi University, Turku Bioscience Centre, Turku, Finland*
[3]*Turku University Hospital, TYKS Orto Surgical Hospital, Turku, Finland*
[4]*Turku University Hospital and University of Turku, Department of Orthopaedics and Traumatology, Turku, Finland*

**Abstract Text**

**Background/Purpose:** Bone marrow (BM) is a unique microenvironment which hasn't been extensively studied due to inaccessibility and spatial heterogeneity. Human BM is often studied using iliac crest biopsies or dissected femoral heads. However, BM in these regions is rich in hematopoietic cells and may be different from adipocyte-rich BM environment which encompasses most of the adult human skeleton. Here we report a method for isolating femoral bone marrow plasma (BMPL) and compare the proteome and lipidome to respective peripheral blood plasma (PBPL).

**Methods:** We recruited eight patients scheduled for knee or hip arthroplasty surgery (Age:63.0(6.9)yr, Male/female:4/4, BMI:31.7(2.7)kg/m^2). BM sample was collected from femoral mid-diaphysis with large curette into heparin-containing tubes. First, tissue pieces were removed by centrifugation (200g, +4°C, 2min) after which clear BM plasma was collected by additional centrifugation (2000g, +4°C, 10min). PBPL was collected with standard procedures. We employed a multiomics approach to compare BMPL and PBPL. For proteomic analysis, we used enrichment method for extracellular vesicles (MagNet), followed by protein purification and LC-MS/MS analysis. For lipidomic analysis, plasma proteins were first precipitated and removed utilizing MeOH:$CHCl_3$ and then analysed with LC-MS/MS.

**Results:** BMPL had significant enrichment ($p<0.05$) in pathways concerning nucleic acid biosynthetic processes, DNA repair, ROS neutralization, and energy metabolism from carbohydrates and alternate sources. PBPL proteome was significantly enriched in pathways related to coagulation, complement activation, inflammation, and cell adhesion. Lipidomic analysis revealed that BMPL contained more ceramides ($p<0.001$) and di- and triglycerides ($p<0.007$, $p<0.001$) than PBPL, but lacked the presence of cholesteryl esters and had lower numbers of sphingomyelins ($p<0.01$) and phosphatidylcholines ($p<0.0001$) than PBPL.

**Conclusions:** Femoral BM microenvironment doesn't have similar levels of proteins related to coagulation and inflammation as peripheral blood, but instead is more enriched with proteins of biosynthetic and nucleic acid processes. This is also reflected in its unique lipid composition.

**P005**

**The complement system in Staphylococcus aureus-dependent osteomyelitis**

*Valentina Granata[1,2], Valentina Possetti[3], Dario Strina[1,2], Federica Violi[1], Alberto Mantovani[1,4], Cristina Sobacchi[1,2], Antonio Inforzato[1,3]*
*[1]IRCCS Humanitas Research Hospital, Department of Immunology and Inflammation, Rozzano, Italy*
*[2]National Research Council, Institute for Genetic and Biomedical Research, Milan, Italy*
*[3]Humanitas University, Department of Biomedical Sciences, Pieve Emanuele, Italy*
*[4]Queen Mary University of London, Harvey Research Institute, London, United Kingdom*

**Abstract Text**

**Background/Introduction:** Osteomyelitis (OM) is a bone infection arising from hematogenous spread or post-surgical/traumatic contamination. *Staphylococcus aureus* (SA) is the main causative pathogen, capable of evading immunity, developing antibiotic resistance, and establishing chronic colonization[1].

**Purpose:** This study investigated the pathogenetic mechanisms of SA-dependent OM, focusing on the complement system, a key regulator of innate immunity and bone homeostasis[2] (see panel A in the accompanying figure).

**Methods:** A murine model of OM was established via intra-bone injection of SA (panel B). The role of C3, a central complement component, was studied using $C3^{-/-}$ mice. Local and systemic infection and inflammation were assessed by microbiological analyses and proximity extension assays.

**Results:** $C3^{-/-}$ mice had sustained weight loss (-6.4 & - 7.2% at 3 and 6 dpi, respectively; panel C, upper) and elevated systemic inflammation assessed based on PTX3 serum levels (mean ±SEM, 171.4 ±37.6 *vs* 59.2 ±4.8 & 149.7 ±20.1 *vs* 59.7 ±6.9 ng/ml at 3 and 6 dpi, respectively; panel C, lower), compared to PBS controls. C3 deficiency promoted bacterial dissemination to contralateral bone, spleen, and kidneys, whereas in WT mice SA remained localized in the injected limb and liver (panel D). While bone colonization was similar (panel E, upper), liver bacterial load (1310 ±497 *vs* 46±13 CFU/organ weight; panel E, lower) and bone inflammatory mediators (including IL-6 and IL-1β, panel F) were higher in $C3^{-/-}$ mice.

**Conclusions:** C3 deficiency in SA-dependent OM led to systemic dissemination and heightened bone inflammation, highlighting the complement system's critical role in controlling infection.

**Keywords:** Bone infections, osteomyelitis, *Staphylococcus aureus*, innate immunity, complement.

**Ethics and Funding:** Approved by the Italian Ministry of Health (n. 406/2022-PR, July 4, 2022) and funded by Fondazione Beppe and Nuccy Angiolini Onlus.

**Bibliography:**

1. Song et al Clin Exp Med 2025;25(1):131

2. Mödinger Y et al Semin Immunol 2018;37:53

**Accompanying figure**:

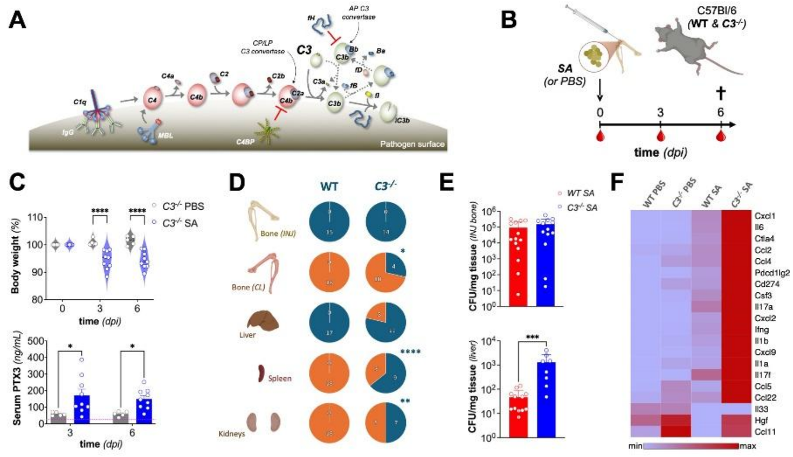

A) **Overview of the complement system** underscoring the central role of C3. B) **Outline of the mouse model of SA-OM** that recapitulates open-fracture and surgery-associated infections of the lower limbs in humans. A methicillin-sensitive, biofilm-forming SA isolate (or PBS, as control) was inoculated ($10^5$ CFU/mouse) into the femoral medullary canal of WT and *C3⁻/⁻* mice. These were sacrificed at 6 days post-infection (dpi), and bacterial colonization of the injected bone, dissemination to the contralateral limb, liver, spleen, and kidneys, as well as systemic (serum) and local (bone) inflammatory markers, were assessed. C) **Body weight and serum PTX3** were measured at 6 dpi (mean ±SEM; n=5-9 from 3 independent experiments; *p<0.05, ****p<0.0001, 2way ANOVA). The purple dotted line indicates the PTX3 concentration in untreated mice (26.88 ±8.77 ng/mL). **D & E) Infection.** Pie charts (**D**) showing number and frequency of infected and non-infected organs in SA-injected WT and *C3-/-* animals. n=14-18/mice per group from 4 independent experiments; *p<0.05, **p<0.01, ****p<0.0001 (WT SA vs *C3-/-* SA), Fisher's exact. Live SA in the injected bone (**E**, upper) and liver (**E**, lower) homogenates of WT and *C3-/-* mice at 6 dpi, expressed as CFU/organ weight (mean ±SEM; n=11-17 mice/group from 4 independent experiments; ***p<0.001, Mann-Whitney. **F) Bone inflammation**. Homogenates of the PBS- or SA-injected limbs from WT and *C3-/-* mice were run on a proximity extension assay (Olink Target 48 Mouse Cytokine). Concentration of the indicated inflammatory markers expressed as median in the heatmap (n=2-4/mice per group from 2 experiments).

**P006**

**Sclerostin Downregulation in Aseptic Loosening**

*__Assil-Ramin Alimy__[1], Eva Goedecke[1], Ana Ocokoljic[1], Christian Ries[1], Frank Timo Beil[1], Tim Rolvien[1]*
*[1]University Medical Center Hamburg-Eppendorf, Department of Trauma and Orthopedic Surgery, Hamburg, Germany*

**Abstract Text**

**Background:** Aseptic loosening (AL) is one of the leading indications for revision surgery after total hip and knee arthroplasty, yet its diagnosis and underlying mechanisms remain poorly defined.

**Purpose:** The aim of this study was to identify signatures associated with AL through targeted proteomic profiling of synovial fluid and analysis of periprosthetic bone tissue characteristics.

**Methods:** Patients were included across three groups: primary osteoarthritis (OA) (n=13), AL (n=15), and other aseptic revision indications (AR) (n=12). Bone-focused multiplex synovial fluid profiling was performed. Additionally, histomorphometry, quantitative backscattered electron imaging (qBEI), and immunofluorescence were performed on trabecular bone specimens.

**Results:** We detected a distinct reduction of the osteocyte-derived Wnt inhibitors sclerostin and DKK1 in AL compared with OA (p=0.026) and AR (p=0.029) (Fig. 1A). Histomorphometric analysis of periprosthetic trabecular bone samples demonstrated preserved microarchitecture and turnover across groups (Fig. 1B). However, qBEI revealed enlarged osteocyte lacunae specifically in AL (OA: 37 ± 4 $\mu m^2$ vs. AL: 46 ± 8 $\mu m^2$ vs. AR: 36 ± 3 $\mu m^2$, p=0.033) (Fig. 1C). Immunofluorescence confirmed reduced sclerostin expression within periprosthetic bone, mirroring synovial fluid findings.

**Conclusion:** Osteocyte dysregulation, evidenced by reduced sclerostin in both synovial fluid and bone, emerges as a defining feature of AL. Low synovial sclerostin also shows diagnostic promise and, together with suppressed DKK1, points to altered Wnt signaling at the implant-bone interface. Thus, local sclerostin measurements could complement current diagnostics and support the development of osteocyte- and Wnt-targeted preventive strategies.

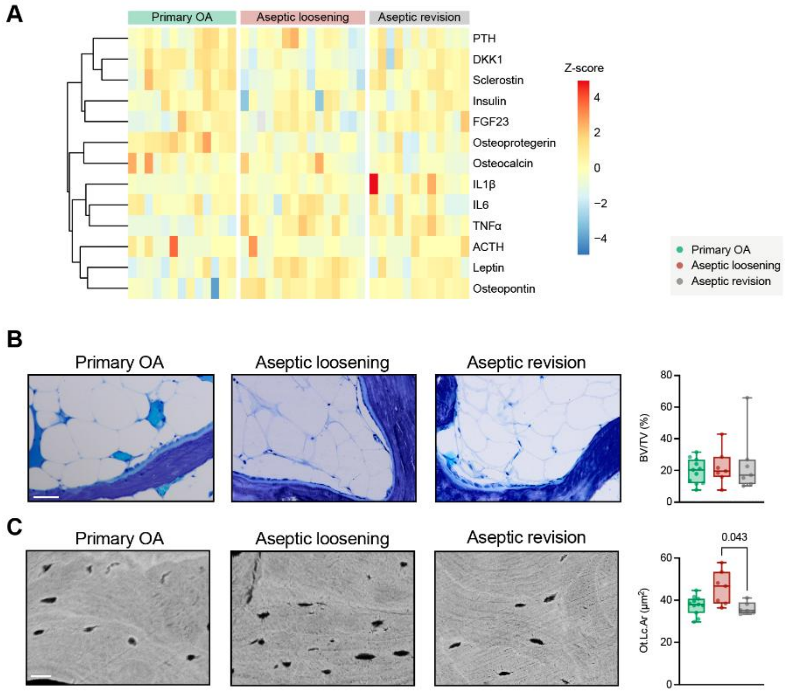

**Figure 1: Decreased synovial sclerostin and osteocyte dysregulation in aseptic loosening**
A) Heatmap of 13 synovial fluid proteins across primary OA, aseptic loosening, and other aseptic revision groups, shown as row z-scores. B) Representative toluidine blue-stained trabecular sections and the corresponding histomorphometric quantification of bone volume fraction (BV/TV). Scale bar: 50 μm. C) Representative images derived from quantitative backscattered electron imaging (qBEI) with quantification of osteocyte lacunar area (Ot.Lc.Ar) across groups. Scale bar: 20 μm.
Statistical analysis was performed using one-way ANOVA with Tukey's post hoc test or the Kruskal-Wallis test with Dunn's multiple comparisons, as appropriate. Exact p-values are reported for significant comparisons unless $p < 0.001$.

**P007**

**How Obesity Affects Vitamin D Metabolism: Novel Human Evidence from Plasma and Liver samples Analyzed by HPLC-MS/MS**

*Agnese Giamborino[1], Cristina Dettori[1], Valentina Vitelli[1], Leonardo Galfo[1], Rebelos Eleni[2], Miika Honka[3], Alessandro Saba[1], Federica Saponaro[1]*
*[1]University of Pisa,*
*Department of Surgical- Medical and Molecular Pathology and Critical Care Medicine, Pisa, Italy*
*[2]University of Turku - University of Pisa,*
*Turku PET Centre - Department of Clinical and Experimental Medicine, Pisa, Italy*
*[3]University of Turku, Turku PET Centre, Turku, Finland*

**Abstract Text**

**Introduction:** HypovitaminosisD relates to metabolic syndrome and obesity. This study investigates vitamin D metabolites in plasma and liver biopsies of obese vs healthy individual.

**Methods:** A novel HPLC-MS/MS method quantified simultaneously Vitamin D3, 25(OH)D3; 1,25(OH)$_2$D3, 24,25(OH)$_2$D3, in plasma and tissues. 70 obese and 40 lean patients underwent liver biopsies during bariatric or benign abdominal surgery. Preoperative plasma samples and clinical data were collected; all gave informed consent.

**Results**:

| **Parameter** | **Obese (mean ± SD)** | **Lean (mean ± SD)** | **P signif.** |
|---|---|---|---|
| Age (years) | 46,4 ± 10,3 | 42,1 ± 11,6 | ns = 0,2 |
| BMI (kg/m$^{3)}$ | 41,2 ± 4,4 | 24,1 ± 2,7 | < 0,0001 |
| Plasma glucose (mmol/L) | 5,7 ± 0,7 | 5,3 ± 0,4 | ns=0,05 |
| Plasma insulin (µU/mL) | 14,9 ± 8,4 | 6,8 ± 3,4 | < 0,0001 |
| Hb1Ac (%) | 5,7 ± 0,05 | 5,08 ± 0,3 | < 0,0001 |
| Vitamin D plasma (ng/mL) | 2,4 ± 1,9 | 3,5 ± 2,4 | ns=0,09 |
| 25(OH) plasma (ng/mL) | 26,47 ± 8,9 | 24,43 ± 8,27 | ns=0,33 |
| 24,25(OH)$_2$ plasma (ng/mL) | 1,88 ± 0,7 | 1,9 ± 0,8 | ns=0,91 |
| 1,25(OH)$_2$ plasma (ng/mL) | 0,10 ± 0,04 | 0,10 ± 0,04 | ns=0,27 |
| 25(OH) liver (ng/g) | 2,15 ± 1,2 | 3,34 ± 1,8 | 0,036 |
| 24,25(OH)$_2$ liver /ng/g) | 0,16 ± 0,1 | 0,57 ± 0,20 | < 0,0001 |

No significant plasma metabolites differences were found pre and post-surgery.

In both obese and lean patients, two hepatic metabolites were measured. 25(OH) was significantly higher in patients with BMI < 30 than in those with BMI > 30 (p=0.036). Cholecalciferol release and obesity-related metabolic changes enhancing hydroxylation and utilization. 24,25(OH)$_2$ showed a similar pattern (p < 0.0001).

Higher 25(OH) ratio tissue/plasma levels correlated negatively with glucose-6-phosphate, fructose-6-phosphate (p= 0.01 and p=0.03 respectively) and cysteine (p=0.04, suggesting enhanced glycolysis and antioxidant effects.

**Conclusions:** This study links hypovitaminosis D to obesity, and offers first human preliminary data on tissue Vitamin D metabolism.

**ND-P002**

**Screening of musculoskeletal status in spinal surgery patients: FNDC5/irisin involvement**

*Luisa Pellicani*[1], *Clelia Suriano*[1], *Angela Oranger*[2], *Sara Carpentieri*[3], *Maria Grano*[2], *Giovanni Barbanti Brodano*[4], *Francesco Grassi*[3], *Silvia Colucci*[1]
[1]*University of Bari, Department of Translational Biomedicine and Neuroscience DiBraiN, Bari, Italy*
[2]*University of Bari, Department of Precision and Regenerative Medicine and Ionian Area, Bari, Italy*
[3]*IRCCS Istituto Ortopedico Rizzoli, SSD LAb Ramses, Bologna, Italy*
[4]*IRCCS Istituto Ortopedico Rizzoli, SC Chirurgia Vertebrale, Bologna, Italy*

**Abstract Text**

Complications of spinal surgery are still unsolved problems. The identification of circulating markers of musculoskeletal health could be important in the prediction of spinal surgery complications. Recently, the myokine irisin has been recognized for its beneficial effects on bone and muscle. This molecule and its precursor FNDC5, expressed on muscle fibers, are involved in prevention and restoration of musculoskeletal damage of osteosarcopenic-induced murine models. In observational human studies irisin serum levels positively correlate with femoral and vertebral bone mineral density. Here, FNDC5/Irisin system involvement was studied as possible biomarker of musculoskeletal status in pre-operative screening of candidates for spinal surgery. We measured irisin circulating levels in 40 patients (27 female and 13 male) before the surgery, and we correlated them with bone biomarkers and DEXA parameters. In the serum of patients, we demonstrated a positive correlation between irisin and Bone Alkaline Phosphatase ($r=0.34$ $p=0.04$), Procollagen type I N-terminal propeptide ($r=0.37$, $p=0.02$) and C-terminal cross-linking telopeptide of type I collagen ($r=0.35$ $p=0.04$). We demonstrated that irisin levels positively correlate with Total Body (T.B.) Bone Mineral Content ($r=0.40$ $p=0.047$), T.B. t-score ($r=0.41$, $p=0.04$) and T.B. z-score ($r=0.40$, $p=0.045$) from the patients. These findings suggest irisin involvement in remodeling process and mineral content of bone.

We also demonstrated that irisin serum levels and gene expression of FNDC5 in biopsies of paravertebral muscles correlate positively with Myosin Heavy Chain-type I ($r=0.40$, $p=0.02$; $r=0.52$, $p<0.001$, respectively) and Myosin Heavy Chain-type IIa ($r=0.42$, $p=0.02$; $r=0.55$, $p<0.001$, respectively) expression genes, suggesting that irisin is related to markers of muscle structure and function.

Taken together, our results provide evidence that irisin could contribute to defining the preoperative musculoskeletal health status in spinal surgery candidates.

Funded by the European Union - Next Generation EU-NRRP M6C-Investment 2.1 Enhancement and strengthening of biomedical research in the NHS. PNRR-MCNT2-2023-12378098. CUP: H93C24000370006.

**ND-P003**

**Bone-brain crosstalk: cerebrospinal fluid sclerostin levels in the early Alzheimer's disease stages**

*Manuela Dicarlo[1], Luisa Pellicani[2], Patrizia Pignataro[2], Daniele Urso[3], Chiara Zecca[3], Maria Teresa Dell'Abate[3], Michela Taurino[1], Onofrio Daniele Terrevoli[1], Angela Oranger[1], Graziana Colaianni[1], Roberta Zerlotin[1], Clelia Suriano[2], Silvia Colucci[2], Giancarlo Logroscino[3], Maria Grano[1]*
*[1]University of Bari, Department of Precision and Regenerative Medicine and Ionian Area, Bari, Italy*
*[2]University of Bari, Department of Translational Biomedicine and Neuroscience, Bari, Italy*
*[3]University of Bari,*
*Center for Neurodegenerative Diseases and the Aging Brain- Pia Fondazione Card G. Panico, Tricase, Italy*

**Abstract Text**

**Background:** In the intriguing bone-brain crosstalk, the osteocyte-derived sclerostin has recently gained interest as bone-derived hormone involved in memory impairment in Alzheimer's disease (AD) mouse models. However, the existing studies on human cohorts are limited to few reports that did not focus on AD staging.

**Purpose:** We examined the role of sclerostin across the clinical stages of AD.

**Methods:** Sclerostin levels were measured in the cerebrospinal fluid (CSF) of patients with dementia due to AD, mild cognitive impairment (MCI), and subjective memory complaints (SMC), biologically characterized via the amyloid/tau/neurodegeneration classification of the National Institute on Ageing-Alzheimer's Association. Results were correlated with AD biomarkers, CSF amyloid β 42 (Aβ42), phosphorylated tau (p-tau), and total tau (t-tau), and clinical parameters of dementia severity, that is Clinical Dementia Rating Scale-Global score (CDR-GS) and CDR-Sum of Boxes (CDR-SOB).

**Results:** CSF sclerostin levels were significantly increased in patients with dementia due to AD compared to MCI ($P=0.0322$) and SMC ($P=0.0010$). CSF sclerostin correlated negatively with Aβ42 ($r = -0.230$, $P = 0.0052$) and positively with p-tau ($r = 0.328$, $P < 0.001$), t-tau ($r = 0.333$, $P < 0.0001$), and CDR-GS ($r=0.198$, $P=0.0223$). A positive trend was observed with CDR-SOB ($r=0.156$, $P=0.090$). We found significantly lower CSF Aβ42 levels ($P=0.0485$) and trends of increase in p-tau ($P=0.0806$) and t-tau levels ($P=0.0920$) only in MCI patients with high CSF sclerostin levels. By stratifying the study cohort based on their CDR-GS (from 0 to 3), a significant increase in CSF sclerostin levels was noticed in patients with mild dementia (CDR-GS=1) compared to non-demented (CDR-GS=0) ($P=0.0267$).

**Conclusions:** Our findings showed the relevance of bone-brain crosstalk by the association of CSF sclerostin with Aβ42, tau pathology, and dementia severity highlighting the great clinical importance of the osteokine as a promising biomarker of bone status as well as of early AD stages.

**Poster Sessions: Bone bioengineering, regeneration and implants**

**P008**

**Development of Small Molecule Inhibitors for Alveolar Bone Regeneration to Support Dental Implants**

*Andre Van Wijnen*[1], *Yasaman Daneshian*[2]
[1]*University of Vermont, Biochemistry, Burlington, United States*
[2]*University of Vermont, Biochemistry, Burlington- Vermont, United States*

**Abstract Text**

Application of restorative dental devices (e.g., titanium dental implants) is impeded by alveolar bone resorption due to periodontitis in elderly patients. Here, we addressed the compelling clinical need for improved alveolar bone regeneration by developing novel osteogenic small-molecule pharmacotherapies. Analysis of RNA-seq data for mesenchymal stem/stromal cells extracted from third molars (dental pulp stem cells, DPSCs), bone marrow (BMSCs) and adipose tissue (AMSCs) revealed the full complement of functional genes. The epigenetic enzyme EZH2 is robustly expressed in all three cell types and represents a mesenchymal lineage-selective suppressor of osteogenesis. Multiple EZH2 inhibitors (e.g., GSK126, Tazemetostat) stimulate osteoblast differentiation in vitro and in vivo. To expand the range of EZH2s inhibitors that can be considered for osteogenesis in dental applications, we tested a panel of novel compounds (designed to inhibit EZH2) for toxicity and osteogenic efficacy in the MC3T3 osteoblast cell culture system. Qualitatively, results from multiple biological tests revealed several promising new compounds that are soluble (in DMSO), effectively inhibit the enzymatic activity of EZH2 (western blotting), and have low toxicity (metabolic MTS assays and live-dead fluorescence microscopy). These inhibitors also effectively enhance calcium deposition in the extracellular matrix (Alizarin Red staining). Dosing experiments reveal that one treatment for the first three days suffices to provide osteogenic stimulation over 3+ weeks, although two consecutive treatments for six days total is more effective and permits reduced dosing. Thus, we have discovered novel potent and selective small-molecule inhibitors for EZH2 that may be suitable for alveolar bone regeneration or craniofacial bone tissue engineering using dental stem cells.

**P009**

**rhBMP6 and Ceramic Particle Size Modulate Osteochondroprogenitor Recruitment and Osteogenic Marker Immunolocalization in Osteogrow-C Implants**

*Nikola Štoković*[1], *Natalia Ivanjko*[1], *Viktorija Rumenović*[1], *Nataša Kovačić*[2], *Danka Grčević*[2], *Slobodan Vukičević*[1]
[1]*University of Zagreb School of Medicine, Laboratory for Mineralized Tissues, Zagreb, Croatia*
[2]*University of Zagreb School of Medicine, Laboratory for Molecular Immunology, Zagreb, Croatia*

**Abstract Text**

**Background:** Osteogrow-C is a novel bone regeneration therapy composed of recombinant human bone morphogenetic protein 6 (rhBMP6) delivered within an autologous blood coagulum (ABC) combined with synthetic calcium phosphate (CaP) ceramic particles.

**Purpose:** This study aimed to elucidate how rhBMP6 and ceramic particle size influence osteochondroprogenitor cell recruitment and early osteogenic differentiation.

**Methods:** Osteogrow-C implants with ceramic particles of three sizes (74–420, 500–1000, and 1000–4000 µm), along with control implants without rhBMP6, were subcutaneously implanted in rats. Samples were collected at early post-implantation time points of 3 and 5 days. Flow cytometry was used to quantify cell populations expressing mesenchymal and osteochondroprogenitor markers, including CD26, CD44, CD90, CD24, CD140a, and LepR. Immunohistochemical analyses were performed to evaluate the spatial expression of key osteogenic signaling molecules such as Smad1/5/8, PDGFR, and Syndecan in implants with different ceramic particle sizes. Animal study was approved by the National Ethics Committee.

**Results:** Flow cytometric analysis revealed that implants containing rhBMP6 induced significantly higher recruitment and differentiation of early and committed osteochondroprogenitor cells (CD90+, CD24+, CD140a+) compared to implants without rhBMP6. Notably, implants with small ceramic particles exhibited the greatest increase in these progenitor cell populations, suggesting that the larger surface area and specific microenvironment of smaller particles favor cell migration and attachment. Immunohistochemical staining showed increased expression of osteogenic signaling markers co-localizing with progenitor cells in Osteogrow-C implants containing small ceramic particles, corroborating the flow cytometry results. In contrast, control implants without rhBMP6 showed markedly lower progenitor cell recruitment and osteogenic signaling activation, underscoring the critical role of rhBMP6 in initiating and enhancing the early bone regenerative cellular response.

**Conclusions:** These findings demonstrate that Osteogrow-C's efficacy in bone regeneration is determined by both rhBMP6 presence and ceramic particle size, with rhBMP6 critically enhancing progenitor cell recruitment and osteogenic signaling.

**P010**

**Infuse and Osteogrow in Bone Regeneration: Comparative Insights into Activity, Release, and Cellular Response**

*<u>Natalia Ivanjko</u>[1], Nikola Štoković[1], Marina Milešević[1], Ena Vilagoš[1], Nataša Kovačić[2], Danka Grčević[2], Slobodan Vukičević[1]*
*[1]School of Medicine University of Zagreb, Laboratory for Mineralized Tissues, Zagreb, Croatia*
*[2]Croatian Institute for Brain Research- School of Medicine University of Zagreb, Laboratory for Molecular Immunology, Zagreb, Croatia*

**Abstract Text**

**Introduction:** Infuse, composed of rhBMP2 delivered on an absorbable collagen sponge (ACS), remains the only approved osteoinductive device for bone regeneration, yet it has notable limitations. A novel alternative, OSTEOGROW, combines rhBMP6 with autologous blood coagulum (ABC) to address these drawbacks and enhance biocompatibility. This study evaluated the biological activity of rhBMP2 and rhBMP6, characterized their release profiles from ACS and ABC carriers, and investigated the early cellular response during ectopic bone formation in a rat subcutaneous model at 24 and 72 hours, focusing on lymphoid, myeloid, and mesenchymal cell populations.

**Methods**: The bioactivity of rhBMP2 and rhBMP6 was assessed using a C2C12-BRE-Luc cell assay across concentrations from 1 to 400 ng/mL. The release kinetics of rhBMP2 from ABC and ACS were analyzed by ELISA. To characterize the early immune and mesenchymal responses induced by both devices, cell suspensions from implantation sites were labeled with rat-specific antibodies (CD3-PE, B220, GR-FTIC, CD11b-APC, CD44-AF647, CD90-APCCy7, and CD140a) and analyzed on a flow cytometer.

**Results:** Cumulative release data showed that rhBMP2 was released more rapidly and in higher amounts from ACS (85%) compared to ABC (56%) over 72 hours. Lymphoid cell populations (CD3+ and B220+) were significantly enriched in rhBMP6/ABC implants at both 24 and 72 hours, whereas myeloid cells (GR+CD11b+) were predominant in rhBMP2/ACS implants at the same time points. Mesenchymal cell numbers (CD44+) were comparable between groups; however, detailed profiling revealed a higher proportion of adipoprogenitors in rhBMP2/ACS and early multipotent progenitors (EMP) in rhBMP6/ABC implants after 24 hours.

**Conclusion:** The ABC carrier demonstrated a more sustained release profile compared to the ACS carrier. Myeloid populations were significantly higher in rhBMP2/ACS, indicating a stronger immune response, probably due to the xenogenic origin of ACS. Furthermore, adipoprogenitors were mostly present in rhBMP2/ACS implants, while early EMP were mostly present in rhBMP6/ABC implants.

**P012**

**Human Gingival Fibroblast-Mediated Remodeling of Three-Dimensional Fibrin Hydrogels**

*Ton Schoenmaker[1], Merve Ceylan[1], Marja Laine[1], Behwrouz Zandieh Doulabi[2], Hans Korfage[2], Teun Vries de[1]*
*[1]ACTA, department of periodontology, Amsterdam, Netherlands*
*[2]ACTA, department of oral cell biology, Amsterdam, Netherlands*

**Abstract Text**

**Background:** The extracellular matrix (ECM) critically regulates gingival fibroblast behavior during tissue repair and regeneration. However, how culture dimensionality influences fibroblast-mediated ECM remodeling remains unclear.

**Purpose:** To investigate the effects of three-dimensional (3D) fibrin hydrogels on the phenotype and remodeling activity of primary human gingival fibroblasts (GFs) compared to conventional two-dimensional (2D) monolayer cultures.

**Methods and Results:** Live/dead staining confirmed high GF viability in both conditions, with elongated and branched cell morphologies in 3D fibrin hydrogels, contrasting with spindle-shaped cells in 2D monolayers. Hematoxylin and Eosin, and Masson's Trichrome staining revealed progressive fibrin degradation and de novo collagen deposition over 21 days of culturing. Gene expression analysis showed that while FN1, COL1A1, and COL3A1 levels remained relatively stable, TGFB1 expression increased significantly from day 7 to day 14 in 3D hydrogels ($p<0.05$) and was higher than in 2D cultures at day 21 ($p<0.05$). This coincided with a marked upregulation of ACTA2 ($p<0.01$), indicating myofibroblast-like differentiation. MMP-2 activity increased significantly over time in both 2D and 3D cultures ($p<0.01$ and $p<0.001$, respectively). In contrast, PLAU and PLAT expression decreased significantly at days 14 and 21 ($p<0.001$ and $p<0.05$, respectively), reflecting a temporal shift from fibrinolytic to collagenolytic remodeling. Despite active remodeling, mechanical testing showed no significant changes in hydrogel stiffness or relaxation between day 1 and day 7, or between cell-seeded and acellular gels ($p>0.05$), likely due to the small contractile forces generated by the cells relative to the gel's bulk modulus.

**Conclusion:** These findings demonstrate that 3D fibrin hydrogels provide a biologically active and physiologically relevant microenvironment that supports fibroblast-mediated ECM remodeling, offering a biomimetic model for investigating the mechanobiology of periodontal and peri-implant soft tissue regeneration.

**P013**

**Magnesium-based implants induce bone formation independent of αCGRP**

*<u>Till Schoberwalter</u>[1], Ruben Augustin[1], Jan Sevecke[1], Chiara Gerriets[1], Shan Jiang[1], Weixin Xie[1], Jan-Marten Seitz[2], Alexander Kopp[2], Anke Baranowsky[1], Johannes Keller[1]*

*[1]University Medical Center Hamburg-Eppendorf, Department of Experimental Trauma Surgery, Hamburg, Germany*

*[2]Medical Magnesium GmbH, Research & Development, Aachen, Germany*

**Abstract Text**

**Background:** Magnesium (Mg) implants belong to a group of resorbable biomaterials that are gaining increasing relevance in orthopedic trauma surgery. A major advantage of Mg implants over conventional steel or titanium devices is that they not only eliminate the need for subsequent implant removal, but also induce the formation of new bone. In this regard, prior studies suggested that Mg-induced bone formation is associated with local release of osteoanabolic calcitonin gene-related peptide (CGRP). However, it remains unclear whether calcitonin gene-related peptide (CGRP), and specifically CGRP alpha (αCGRP), the isoform predominantly expressed in bone, is an integral component of magnesium-induced skeletal changes.

**Objectives:** To investigate whether αCGRP is necessary for Mg-induced bone formation and to delineate alternative molecular pathways contributing to the osteoanabolic effect of Mg.

**Methods:** Wildtype (WT) and $\alpha CGRP^{-/-}$ mice received intramedullary Mg or titanium (Ti) pins. Skeletal alterations were assessed after 2, 4 and 12 weeks using µCT, histology, and biomechanical testing. For gene expression analysis, qPCR and RNA-Seq were employed.

**Results:** For WT mice at all time points, Mg implants substantially increased the formation of cortical and trabecular bone around the implant compared to Ti. αCGRP$^{-/-}$ mice displayed osteogenic outcomes comparable to WT, demonstrating that αCGRP is not required for Mg-induced bone formation. Histological analyses indicated a greater extent of newly formed bone in the Mg groups of both genotypes, accompanied by increased expression of bone turnover markers and enhanced biomechanical stability. Transcriptomic profiling revealed several Mg-responsive signaling pathways, particularly WNT-associated networks, as potential molecular circuits that contribute to Mg-induced bone formation.

**Conclusions:** Magnesium implants elicit a strong osteoanabolic effect, which occurs independent of αCGRP. The effect results from enhanced osteoblast activity associated with increased WNT signaling. These findings refine our current biological understanding of Mg-based implants and reinforce their potential as resorbable, osteogenic devices for use in orthopedic applications.

**P014**

**Investigating the Bone-Regenerative Potential of a CaP Nanoparticle-Based Hydrogel**

*<u>Kai Böker</u>[1], Katarina Vasic[1], Benedikt Kruse[2], Jörg Wilting[3], Marina Komrakova[1], Matthias Epple[2], Arndt F. Schilling[1], Wolfgang Lehmann[1]*
*[1]University Medical Center Göttingen,*
*Department for Trauma Surgery- Orthopaedics and Plastic Surgery, Göttingen, Germany*
*[2]University of Duisburg-Essen, Inorganic Chemistry, Göttingen, Germany*
*[3]University Medical Center Göttingen, Institute of Anatomy and Cell Biology, Göttingen, Germany*

**Abstract Text**

**Background**: The development of a biodegradable and biocompatible bone adhesive could revolutionize fracture treatment by reducing patient risks and recovery time. However, a commercially available bone adhesive is currently not available.

**Purpose**: Functionalized calcium phosphate (CaP) nanoparticles represent a promising platform for the development of such a bone adhesive. In this study, we investigated the effectiveness of an experimental CaP nanoparticle-based hydrogel on bone healing processes in vitro and in ovo.

**Methods**: The CaP hydrogel was formulated by combining freeze-dried calcium phosphate carboxymethyl cellulose silicates (CaP/CMC/$SiO_2$) with a 2% aqueous alginate solution at a mass ratio of 3.6:1. The effect of CaP hydrogel on the differentiation of osteoclasts and osteoblasts was examined. TRAP and DAPI staining were used to confirm osteoclast differentiation, while Sirius Red staining showed collagen deposition of osteoblasts. Effect on angiogenesis was tested by CAM assays. To evaluate the effect of CaP hydrogel on bone healing, femora from 11-day-old chick embryos were osteotomized and treated with CaP samples. After 10 days of culture, the bones were scanned with high-resolution CT at an isotropic resolution of 6.8 µm.

**Results**: Sirius Red staining showed comparable collagen deposition on the CaP surface (55 ± 16%) and the control surface (38 ± 8%, unpaired t-test: $p > 0.05$). Using TRAP staining, multinucleated, TRAP-positive cells could be detected on the CaP surface. CAM assays revealed no suppression of angiogenesis. Micro-CT assessment of embryonic femora showed a bone volume, cortical porosity, average cortical thickness and bone mineral density comparable to the untreated bones.

**Conclusion**: The in-vitro results show that bone cells are functionally active in the presence of the newly developed CaP/CMC/$SiO_2$ hydrogel. In-ovo analyses suggest that the CaP/CMC/$SiO_2$ hydrogel does not adversely affect individual parameters of the bone healing process.

Future studies will focus on increasing biomechanical properties of different bone glue formulations.

**P015**

### Submicron Optical-Photothermal Infrared Micro-spectroscopy Enables High-Resolution Characterization of Bone Composition, Disease Phenotypes, and Biomaterial-Induced Biomineralization

*Miriam Unger*[1]
[1]*Photothermal Spectroscopy Corp GmbH, Applications, Muelheim an der Ruhr, Germany*

**Abstract Text**

**Background:** Submicron-resolution characterization of bone composition is essential for understanding bone disease, collagen integrity, and biomineralization at the tissue–material interface. Optical photothermal infrared (O-PTIR) spectroscopy, with simultaneous Raman acquisition, overcomes diffraction limits and enables biochemical and structural imaging at ≤500 nm resolution, offering new capabilities for probing bone quality in both regenerative and pathological contexts.

**Purpose:** This work demonstrates the utility of O-PTIR spectroscopy and imaging for (i) characterizing bone tissue composition in health and disease and (ii) elucidating biomineralization mechanisms driven by novel intrinsically disordered peptides (IDPs) incorporated into bone graft substitutes.

**Methods:** O-PTIR served as the primary analytical tool to quantify mineral–matrix composition, collagen structure, and peptide-mediated biochemical changes. Using a mid-IR pump and visible probe, O-PTIR enabled simultaneous IR and Raman acquisition from thick PMMA-embedded bone without microtomy. Hyperspectral maps captured submicron distributions of phosphate, carbonate, and amide I/II features, while polarization-sensitive O-PTIR with three-angle acquisition resolved ~1 µm collagen fiber orientation. Machine learning (support vector machines) classified wildtype vs. osteogenesis imperfecta bone based on collagen-range spectra. In parallel, two proline-rich IDPs (P2, P6) were integrated into SmartBone® scaffolds (SBN) and implanted into porcine cranial defects for 8 or 16 weeks; O-PTIR assessed mineralization and peptide conformational changes at the bone–material interface.

**Results:** SBN + P6 induced significantly greater early bone formation (8 weeks), whereas SBN + P2 promoted enhanced late-stage mineralization (16 weeks). O-PTIR detected strong amide I/II shifts (1660, 1546 $cm^{-1}$) and higher phosphate intensity (1037 $cm^{-1}$) in P2 groups, indicating peptide restructuring and mature mineral deposition. Submicron O-PTIR resolved mineral and collagen features around microporosity, while SVM classified bone phenotypes with >86% accuracy. Polarization-sensitive imaging revealed collagen fibers undetectable by FT-IR.

**Conclusion:** O-PTIR provides transformative, label-free, submicron biochemical and structural insights into bone disease, collagen architecture, and biomaterial-driven biomineralization.

**P016**

**Injectable Carrageenan-Alginate-CNF Hydrogels Functionalized with S53P4 Bioactive Glass Dissolution Extract for Bone Regeneration**

*<u>Negin Vaziri</u>[1], Mona Gibreel[2], Lippo Lassila[2], Timo Peltola[3], Jorma Määttä[1], Pekka Vallittu[2], Terhi Heino[1]*
*[1]Institute of Biomedicine, Faculty of Medicine, University of Turku, Turku, Finland*
*[2]Department of Biomaterials Science and Turku Clinical Biomaterials Centre (TCBC), Institute of Dentistry, University of Turku, Turku, Finland*
*[3]Research Development and Production Department, Stick Tech Ltd-Member of GC Group, Turku, Finland*

**Abstract Text**

**Background:** Despite bone's the inherent regenerative potential, current clinical approaches remain limited by invasiveness, poor graft integration, and insufficient vascularization. However, injectable hydrogels represent an emerging strategy as minimally invasive scaffolds for bone regeneration.

**Purpose:** Here, we developed an injectable and bioactive hydrogel based on carrageenan, alginate, and cellulose nanofibers (CNF)**,** enriched with the ion dissolution products of S53P4 bioactive glass at varying extract concentrations (0%, 20%, 40%, and 80%) followed by crosslinking with calcium chloride.

**Methods:** Hydrogel properties were systematically assessed through physicochemical, rheological and mechanical characterization, along with swelling and degradation kinetics, ion release profile, and biomineralization capacity prior to conducting cytocompatibility and osteogenic differentiation assays.

**Results:** Formulations with moderate bioactive glass dissolution extract (BGE) maintained stable viscosity with minor rheological changes, while demonstrating higher compressive strength than the 0% control ($P$ = 0.005 for 20%, $P$=0.028 for 40%, no further gain at 80%). BGE dose-dependently increased swelling and biomineralization, with significant effects at 40% ($P$=0.0036) and 80% ($P$=0.0039) versus control. MC3T3-E1 pre-osteoblasts showed improved viability and attachment with increasing BGE content, with metabolic activity significantly in 80% hydrogels compared to 0%, 20%, and 40% ($P$= 0.0063, 0.0100 and 0.0243, respectively). Importantly, BGE-containing formulations promoted spontaneous osteogenic differentiation in basal medium, as demonstrated by increased alkaline phosphatase expression and enzyme activity after 14 days (80%: $P$=0.0088; 40%: $P$=0.0047; 20%: $P$=0.0352 versus 0% control), whereas all groups exhibited similar differentiation capacity when cultured in osteogenic medium.

**Conclusion:** These results demonstrate that this naturally-derived injectable hydrogel has favorable properties to promote *in vitro* bioactivity, biocompatibility, and bioactive glass-mediated osteogenic differentiation and could offer a minimally invasive approach to improve bone regeneration.

**Keywords:** Injectable hydrogel, Bioactive glass extracts, Bone tissue regeneration, Carrageenan, Alginate, Cellulose nanofibers.

**P017**

**Bone Quality at the Magnesium Implant–Bone Interface in Rat Femur: A Multiscale Approach**

*Amel Batouche*[1], *Silja Paul*[1], *Sven Schimek*[2], *D.C.Florian Wieland*[2], *Imke Fiedler*[1], *Katharina Jähn-Rickert*[1], *Björn Busse*[1]
[1]*University Medical Center Hamburg-Eppendorf, Osteology and Biomechanics, Hamburg, Germany*
[2]*Metallic Biomaterials, Helmholtz-Zentrum hereon, Hamburg, Germany*

**Abstract Text**

**Introduction:** Most implants for fracture treatment are non-degradable and therefore remain in the body indefinitely. The stiffness mismatch between metallic alloys such as titanium (Ti) alloys (the gold standard) and bone can cause stress shielding, which leads to bone loss and compromises the bone's mechanical integrity. Moreover, in the case of temporary implants, a second removal surgery is required, adding further risk for patients. Magnesium alloys (Mg) offer an alternative to implants: their biodegradability and biocompatibility allow for resorption by the body, eliminating the need for secondary surgeries, while being closer to bone stiffness.
This study aims to investigate multiscale changes in bone quality and structure in Mg-implanted samples compared to Ti-alloys.

**Methods:** First, Quantitative Backscattered Electron Microscopy (Crossbeam 340, Zeiss, Germany) was used to obtain 2D images of bone cross-sections at the implant–bone interface to examine bone mineral density distribution and measure new bone formation. Additionally, to describe the osteocyte network, 2D lacunar density was assessed using XamFlow (LucidConcepts, Switzerland). Finally, nanoindentation (iNano, KLA Instruments, USA) was used to determine the local mechanical properties of the bone.
All data were statistically evaluated using suitable analytical methods.

**Results:** Overall, the Mg-implanted bones showed no significant differences compared to the Ti-implanted ones, although several trends emerged across groups. The calcium content at the bone–implant interface and the lacunar density tended to be lower and more heterogeneous in the Mg samples than in the titanium controls. New bone formation also tended to be higher in the Mg group compared with the Ti group, although not significantly. Finally, the nanoindentation results were consistent with these observations, as both the elastic modulus and hardness tended to be lower, also not significantly, in the Mg samples.

**Conclusion:** This study demonstrates that this Mg alloy does not impact bone quality compared to Ti-implants and may be suitable candidate for implant surgeries

**P018**

**Fibroblasts and mesenchymal stem cells coculture reveals distinct extracellular matrix and osteogenic profiles in bone healing models**

*Eva Šebová*[1], *Michala Klusáček Rampichová*[1], *Eva Filová*[1]
[1]*Institute of Experimental Medicine CAS, Tissue Engineering, Prague, Czech Republic*

**Abstract Text**

**Background and aim:** Fibroblasts contribute significantly to bone regeneration through extracellular matrix (ECM) production and paracrine support of osteogenesis. Clarifying their role in coculture with mesenchymal stem cells (MSCs) may improve cellular bone repair models.
**Methods:** This study investigated interactions between human dermal fibroblasts (HDFn) and bone marrow-derive MSCs in 1:1 coculture under osteogenic conditions. Assessments at days 2, 10, and 17 included ALP activity, osteopontin immunostaining, and qPCR analysis of osteogenic (osteopontin, Runx2, ALP, BMP-2), ECM (periostin, collagen15a1, fibulin-1, NANS), and signaling (cathepsin K, JAK2, STAT3) gene expression.
**Results:** ALP activity in fibroblasts was initially low—23.3-fold lower than MSCs at day 2 ($P < 0.01$)—but increased 35-fold by day 10, surpassing MSCs ($P < 0.05$), then decreased below coculture levels at day 17 ($P < 0.01$). Osteopontin expression remained stable in coculture, significantly higher than in fibroblasts at day 10 ($P < 0.05$), with MSCs peaking at day 17, when the protein signal was detected in all groups. Periostin rose in all groups at day 10, staying elevated only in MSCs at day 17 ($P < 0.05$). Collagen 15a1 expression was higher in fibroblasts at day 2 ($P < 0.01$ vs. MSC) and in coculture at day 10 ($P < 0.01$ vs. MSC) before declining. Cathepsin K was strongly expressed, with coculture exceeding MSCs at day 2 ($P < 0.05$) and maintaining this trend at day 17.
Figure 1 shows additional distinct expression patterns in the heat map.

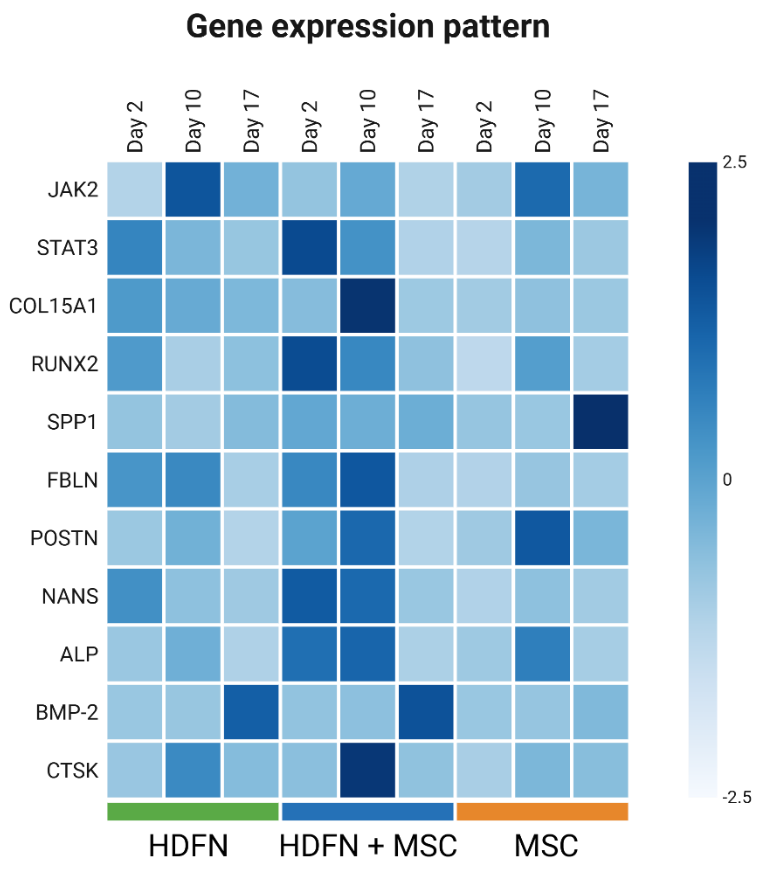

**Figure 1**
(Created in https://BioRender.com).

**Conclusion**: Fibroblasts exhibit osteogenic differentiation and distinct ECM gene expression patterns both alone and with MSCs, emphasizing their active contribution to bone healing. These findings suggest that the cooperative role of fibroblasts with other cell types—particularly MSCs—may be more significant in regulating tissue repair and matrix remodeling than previously assumed.

Supported by the Ministry of Education, Youth and Sports, CR, project: Excellent Research in Regenerative Medicine CZ.02.01.01/22_008/0004562.

**P019**

**Anti-miR-195-Engineered Mesenchymal Stem Cells Promote Migration, Vascularization, and ECM Protein Expression In Vivo**

*Sara Moura[1], Mafalda Montenegro Cortez[1], Isabel Brandão[1,2], Inês Alencastre[3], Susana Gomes Santos[1], Carla Cunha[1], Maria Inês Almeida[1,5]*
*[1]i3s - Instituto de Investigação e Inovação em Saúde, INEB - Instituto de Engenharia Biomédica, Universidade do Porto, Porto, Portugal*
*[2]ICBAS - Instituto de Ciências Biomédicas Abel Salazar, Universidade do Porto, Porto, Portugal*
*[3]RISE - Health, Departamento de Nefrologia R&D, Faculdade de Medicina, Universidade do Porto, Porto, Portugal*

[5]*FFUP - UCIBIO, Laboratório de Bioquímica, Faculdade de Farmácia da Universidade de Porto, Porto Portugal*

**Abstract Text**

**Background/Introduction:** Mesenchymal stromal/stem cells (MSCs) are promising advanced therapy medicinal products (ATMPs) for musculoskeletal disorders, but their in vivo efficacy remains limited by poor survival, engraftment and inconsistent outcomes. MicroRNAs (miRNAs) are post-transcriptional regulators that modulate virtually every major cellular process, including proliferation, differentiation, apoptosis, senescence and angiogenesis, by fine-tuning gene networks. In MSCs, miR-195 inhibition has been identified as a positive regulator of proliferation, osteogenic commitment and VEGF secretion. Therefore, antagonizing miR-195 through miRNA-based engineering represents a promising approach to promote a pro-regenerative MSC phenotype and enhance the potential of MSC-based ATMPs for bone repair.

**Purpose**: This study evaluated whether miR-195 inhibition in MSCs improves theirregenerative properties, focusing on migration, vascularization and osteogenic signature in vitro and in vivo, using a bone heterotopic model.

**Methods**: Human bone marrow-derived MSCs were transiently transfected with an anti-miR-195 inhibitor or scrambled control and analyzed by RNA sequencing and RT-qPCR for selected osteogenic and angiogenic genes. Anti-miR-195- or control-MSCs were seeded on collagen/hydroxyapatite scaffolds and implanted subcutaneously in nude mice for 14 or 28 days. Migration, scaffold colonization, collagen deposition, mineralization and vascularization were quantified by histology combined with image analysis.

**Results:** Anti-miR-195-MSC scaffolds showed ~40 % higher cell migration at day 14 (591.0 µm versus 377.3 µm) and significantly increased collagen deposition at day 28 ($p<0.05$). At day 28, RUNX2 and SPP1 expression levels were 1.56-fold and 3.49-fold higher, respectively, consistent with an enhanced osteogenic signature. The number of blood vessels per scaffold area was significantly higher in the anti-miR-195-MSC condition at day 14 ($p<0.05$), and by day 28 this group exhibited a greater vessel-covered area ($p<0.01$) and increased number of large vessels ($p<0.01$), supporting a pro-angiogenic effect.

**Conclusion**: Anti-miR-195 modulation enhances migration, matrix deposition, osteogenic gene expression and vascularization in vivo, supporting miRNA-based engineering of MSC-ATMPs as a promising strategy for bone repair.

This work was funded by COMPETE2030-FEDER-00727500 (Operation no. 16123; FCT).

**Keywords:** Mesenchymal stromal/stem cells; miRNAs; bone regeneration; vascularization; advanced therapy medicinal products

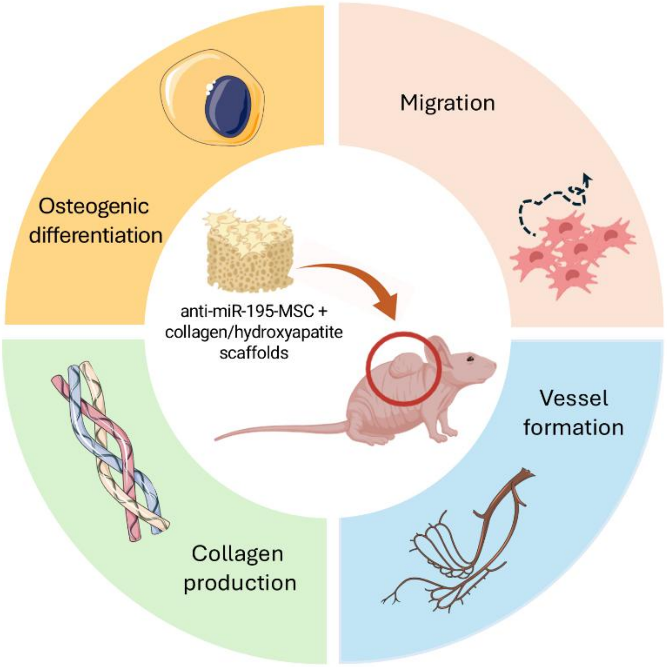

**Poster Sessions: Bone biomechanics and quality**

**P020**

**Skin, Tendon and Muscle Features in the hiOI Mouse Model**

*Laura Ventura*[1,2,3,4], *Lidiia Zhytnik*[1,2,3,4,5], *Yuanyuan Ma*[6], *Xuedan Zhao*[3,7], *Erica I. Salazar Delgado*[7], *Myrthe E. Hoogeland*[8], *Vivian de Waard*[8], *Huub Maas*[3,7], *Richard T. Jaspers*[3,7], *Marie L. Groot*[6], *Elisabeth M.W. Eekhoff*[2,3,4,9], *Dimitra Micha*[1,2,3,4]

[1]*Amsterdam UMC, Department of Human Genetics, Amsterdam, Netherlands*

[2]*Amsterdam UMC, Rare Bone Disease Center, Amsterdam, Netherlands*

[3]*Amsterdam UMC/Vrije Universiteit Amsterdam, Amsterdam Movement Sciences, Amsterdam, Netherlands*

[4]*Amsterdam UMC/Vrije Universiteit Amsterdam, Amsterdam Reproduction and Development, Amsterdam, Netherlands*

[5]*Institute of Biomedical Research in Malaga IBIMA-Plataforma BIONAND, Laboratory of Skeletal Biomedicine, Malaga, Spain*

[6]*Vrije University Amsterdam, LaserLaB- Department of Physics and Astronomy, Amsterdam, Netherlands*

[7]*Vrije Universiteit Amsterdam, Department of Human Movement Science- Faculty of Behavioral and Movement Sciences, Amsterdam, Netherlands*

[8]*Amsterdam UMC,*
*Department of Medical Biochemistry- Amsterdam Cardiovascular Sciences- Atherosclerosis & Ischaemic Syndromes, Amsterdam, Netherlands*
[9]*Amsterdam UMC, Department of Endocrinology and Metabolism, Amsterdam, Netherlands*

**Abstract Text**

**Introduction:** Osteogenesis Imperfecta (OI) is a connective tissue disorder caused by defects in collagen type I genes. Haploinsufficient (HI) OI type 1 results from *COL1A1* defects, causing collagen type I deficiency linked to bone fragility and extraskeletal features.

**Aim:** This study investigated abnormalities in skin, tendons and muscles in a HI OI type 1 (hiOI) mouse model.

**Methods:** hiOI mice were generated by CRISPR/Cas9 knockout of one Col1a1 allele in C57BL/6J mice. Collagen-rich tissues (skin, tendons, muscles) were analyzed in hiOI and wild-type (WT) mice (50% males) at 8 and/or 24 weeks. Collagen expression was measured by RT-qPCR. Dermal collagen fibrils were analyzed by second harmonic generation (SHG) microscopy (8 and 24 weeks). Achilles tendons (24 weeks) underwent mechanical testing under 8% strain and pull-to-failure. Muscle fiber types in the gastrocnemius (GM) were identified using myosin heavy chain (MHC) staining, and metabolic activity was assessed with Succinate Dehydrogenase (SDH) staining (8 weeks).

**Results:** hiOI mice had significantly lower *Col1a1*/*Col1a2* mRNA ratio in skin ($p<0.0001$), with a reduction trend in tendon and muscle. SHG showed reduced dermal collagen content ($p<0.0001$), fibril diameter ($p<0.005$), and more disordered fibril organization.

Tendons showed reduced force ($p<0.05$) and stiffness ($p=0.05$) at 8% strain, with pull-to-failure tests also showing decreased stiffness ($p<0.05$).

hiOI mice had lower GM weight than WT mice ($p<0.05$ for males). GM-to-body weight ratio was similar. hiOI mice exhibited a trend of fewer Type I and IIA and more Type IIB muscle fibers compared to WT. SDH staining showed slightly higher activity in males than females, and in WT mice compared to hiOI mice.

**Conclusion:** The hiOI model shows reduced *Col1a1*/*Col1a2* mRNA expression ratio and collagen type I in skin, altered tendon mechanics, and possible muscle fiber changes, elucidating HI OI type 1 pathophysiology beyond bone.

**P021**

**Diabetes and Femoral Fracture Patterns: A Nationwide Registry Study (1997-2021)**

*Asger Ahlmann Bech*[1], *Annika Vestergaard Kvist*[1], *Peter Vestergaard*[1], *Nicklas Højgaard-hessellund Rasmussen*[1]
[1]*Aalborg University Hospital, Steno Diabetes Center North Denmark, Gistrup, Denmark*

**Abstract Text**

**Background/Introduction:** Femoral fractures are a major cause of morbidity, mortality, and healthcare expenditure. Subtrochanteric and femoral shaft (ST/FS) fractures constitute a clinically important subgroup associated with poorer prognosis. Diabetes affects bone quality, increases fall and fracture risk, and worsens post-fracture outcomes. Diabetes may therefore affect femoral fracture patterns differently, compared to people without diabetes.

**Purpose:** To investigate the impact of diabetes on incidence rates (IR), incidence rate ratios (IRRs), and diabetes-site-interaction of ST/FS and hip fractures.

**Methods:** Using linked Danish health registries, we estimated IRs and IRRs of ST/FS and hip fractures from 1997–2021 in adults aged ≥65 years with type 1 diabetes, type 2 diabetes, or no diabetes, using quasi-Poisson regression. A multivariable logistic regression restricted to individuals with femoral fractures examined the association between diabetes and fracture site, adjusted for age, sex, and bone-related risk factors. Fractures and covariates were identified via ICD-10, ATC, and surgical procedure codes.

**Results:** IRs of both fracture types were higher in people with diabetes than those without. For type 1 diabetes, IRRs were 2.53 ($p<0.001$) for ST/FS and 2.17 ($p<0.001$) for hip fractures; for type 2 diabetes, IRRs were 1.08 ($p=0.002$) and 1.06 ($p=0.008$). No significant diabetes-by-site interaction was detected on a population level ($p=0.28$). Adjusted logistic regression restricted to people having sustained a femoral fracture found both type 1 (OR=1.14, $p=0.002$) and type 2 (OR=1.05, $p=0.008$) diabetes to be significantly associated with higher odds of ST/FS compared with hip fractures.

**Conclusion(s):** Both type 1 and type 2 diabetes were associated with increased incidence of femoral fractures and slightly higher odds of ST/FS than hip fractures after adjustment. People with diabetes may be particularly vulnerable to ST/FS fractures, especially when additional risk factors are present. Consequently, fracture prevention strategies considering ST/FS fractures may be beneficial to this population.

**P022**

**Dual GIP/GLP-2 analogues are capable of inducing bone strength in a mouse model of disuse osteoporosis**

*Aleksandra Mieczkowska[1], Guillaume Mabilleau[1]*
*[1]University of Angers, Inserm UMR_S 1229 RMeS, Angers, France*

**Abstract Text**

**Background:** Several evidence pointed out to the crucial role of gut hormones, and especially GIP and GLP-2, in maintaining bone strength. Although these molecules modulate bone remodeling markers, investigations in preclinical models highlighted that the main action was through enhancement of bone material properties. Recently, we developed a series of dual GIP/GLP-2 analogues capable of increasing bone strength in a validated animal model of post-menopausal osteoporosis.

**Purpose:** The aim of the present study was to investigate whether dual GIP/GLP-2 analogues were capable of enhancing bone strength in an animal model of disuse osteoporosis, the second cause of bone fragility.

**Methods:** Botulinum toxin (BTX) was administered in the right quadriceps femoris of 24 male swiss mice to induce paralysis and disuse osteoporosis. Animals were randomized (n=8/group) to further receive saline, KOL17091, a dual GIP/GLP-2 analogues, or zoledronic acid (100µg/kg). Left hindlimb in saline-treated animals was used as control. Three-point bending, microCT and Fourier-transform infrared microspectroscopy were performed to evaluate bone strength, mass, microarchitecture and bone material properties. Analyses of variance have been performed and considered significant at $p<0.05$.

**Results:** As expected, BTX administration led to a hindlimb paralysis, reductions in bone strength and bone mass, and alterations in bone microarchitecture and material properties. Administration of zoledronic acid, the gold standard in the treatment of osteoporosis, led to improvement in bone mass and bone microarchitecture, but failed to enhance either bone strength or bone material properties. On the other hand, administration of KOL17091 resulted in higher bone strength mostly by reversing alterations of bone material properties.

**Conclusions:** This study highlights the potential of dual GIP/GLP-2 analogues, and especially KOL17091, for the treatment of bone fragility. Further studies are required to assess whether all dual GIP/GLP-2 analogues exert such bone effects and whether these molecules are potent to increase bone strength in humans.

**P023**

**Persistence cortical and trabecular bone loss in adult patients with Osteogenesis Imperfecta.**

*<u>Blandin Camille</u>[1], Agnes Ostertag[2], Thomas Funck-Brentano[3], Corinne Collet[2], martine Cohen solal[2]*
*[1]Rheumatology, Lariboisière hospital, paris, France*
*[2]Inserm U1132, Lariboisière hospital, paris, France*
*[3]Rheumatology, Lariboisière hospital, paris, France*

**Abstract Text**

**Introduction:** Fractures incidence is high in adults with osteogenesis imperfecta (OI) that results in a high morbidity. The longitudinal changes in bone microarchitecture (BM) and the contribution to the fracture incidence remain unknown.

**Objectives:** We aimed to analyze prospectively the changes in BM and the prediction value of fracture incidence.

**Methods:** BM was measured at the tibia (HRpQCT) in patients with OI from 2014 to 2024. Longitudinal changes in Cortical (Ct.thickness ; Ct.vBMD) and trabecular (Tb.BV/TV ;Tb. vBMD) bone were analyzed by mixed effect models including interaction term Time*sex (men, women, menopausal status), adjusted for age, ethnicity and treatment at baseline. The probability of incident fracture was investigated by a logistic regression, including changes in structural parameters, sex, age, variant type and treatments.

**Results:** 55 patients were included at the initial visit (23 men, 32 including 6 menopausal women, mean age 43±14 years), median duration of follow-up 2.7 years (2.1-4). Five patients received a treatment at baseline. At the tibia, BV/TV decreased in men (-0.0030/yr, p<0.01) and in premenopausal women (-0.0027/yr, p<0.05), along with mild loss of Ct.Th (0.0735 mm/yr and 0.0813 mm/yr respectively, p<0.001). A similar reduction in Tb. and Ct.vBMD was observed (p<0.05 for both population). Conversely, in postmenopausal women, BV/TV increased (0.0019/yr, p<0.05) while cortical thickness decreased over time (-0.0819 mm/yr, p<0.0001) consistent with progressive cortical trabecularization. Incident fracture showed no association with the changes in the microarchitecture neither with changes in vBMD adjusted for associated factors. Same results were found at the radius.

**Conclusion:** Cortical and trabecular microarchitecture and vBMD changed significantly with time, according to gender/menopausal status. However, the probability of fractures is independent of the changes in any of the structural or BMD parameters. Longer follow-up is required to assess the predictive value of bone loss in fracture incidence.

**P024**

**Fracture risk in people with Type 2 diabetes and co-existing cardiovascular disease: a nationwide case-control study**

*Aksayan Arunanthy Mahalingasivam1*[1], *Annika Vestergaard Kvist*[2], *Peter Vestergaard*[1], *Nicklas Højgaard-Hessellund Rasmussen*[1]
[1]*Steno Diabetes Centre North Denmark- Aalborg University Hospital- Aalborg- Denmark, Department of Clinical Medicine- Aalborg University- Aalborg- Denmark, aalborg, Denmark*
[2]*Steno Diabetes Centre North Denmark, Aalborg University Hospital- Aalborg- Denmark, Aalborg, Denmark*

**Abstract Text**

**Background:** People with type 2 diabetes (T2D) have elevated risks of cardiovascular disease (CVD) and fractures, but how composite CVD, specific CVD subtypes, and cardiovascular medications influence fracture risk is unclear. We also examined whether associations differed by sex.

**Methods:** We conducted a nationwide registry-based case–control study including 12,390 adults with T2D who sustained a first fracture (2013–2021), matched 1:3 to 37,170 fracture-free controls by age, sex, T2D onset, and weighted Charlson Comorbidity Index. Composite CVD included atrial fibrillation/flutter, acute myocardial infarction, atherosclerosis, chronic kidney disease, heart failure, hypercholesterolemia, hypertension, and stroke; each condition was also analysed individually. Conditional logistic regression estimated odds ratios (ORs) with 95% confidence intervals (CIs), and sex interactions were tested.

**Results:** Composite CVD was associated with higher fracture risk (OR 1.20; 95% CI 1.15–1.26; p<0.01). Several CVD subtypes showed positive associations: atrial fibrillation/flutter (OR 1.18; 95% CI 1.08–

1.31), atherosclerosis (OR 1.11; 95% CI 1.01–1.23), chronic kidney disease (OR 1.37; 95% CI 1.23–1.52), heart failure (OR 1.16; 95% CI 1.03–1.30), hypercholesterolemia (OR 1.12; 95% CI 1.06–1.19), hypertension (OR 1.20; 95% CI 1.15–1.26), and stroke (OR 1.23; 95% CI 1.15–1.31). Acute myocardial infarction showed an inverse association (OR 0.90; 95% CI 0.83–0.97; p<0.01).

Among medications, fracture risk was higher with factor Xa inhibitors (OR 1.41; 95% CI 1.17–1.69), nitrates (OR 1.14; 95% CI 1.01–1.28), and antiplatelet agents (OR 1.16; 95% CI 1.09–1.23). Sex-related differences were limited to selected cardiovascular medications, whereas composite CVD and all CVD subtypes showed no interaction by sex.

**Conclusion:** Composite CVD and several CVD subtypes were independently associated with increased fracture risk in T2D, with additional contributions from selected cardiovascular medications. Sex differences were minimal. Fracture risk assessment in T2D should incorporate cardiovascular status and medication use.

**P025**

**Is the iliac crest, the standard biopsy site, a good predictor of bone quality in other fracture relevant skeletal sites?**

*Elisa Zamengo[1], Stéphane Blouin[1], Andrea Berzlanovich[2], Barbara M. Misof[1], Markus A. Hartmann[1]*
*[1]Ludwig Boltzmann Institute of Osteology at Hanusch Hospital of OEGK and AUVA Trauma Centre Mei dling, Material Science, Vienna, Austria*
*[2]Medical University of Vienna, Center of Forensic Medicine, Vienna, Austria*

**Abstract Text**

**Introduction:** Understanding trabecular bone microarchitecture at multiple anatomical sites is essential for assessing fracture risk. While the transiliac bone biopsy is frequently used as a reference site, its ability to reflect bone microarchitecture at other relevant skeletal sites remains unclear.

**Purpose:** This study compares trabecular bone microarchitecture at the transiliac biopsy site and other fracture-relevant skeletal sites in healthy individuals.

**Methods:** Bone autopsy samples from the iliac crest, distal radius, femoral neck and L1 vertebral body were collected from 16 bone-healthy females (middle-aged: n=8, mean age 62.4±5.5 years; elderly: n=8, mean age 92.3±4.0 years) (ethical approval: Medical University Vienna, EK Nr. 1757/2013). MicroCT scans (SCANCO 50, 24 µm resolution, 90kV) were performed and trabecular structural parameters were computed. The transiliac biopsy site was virtually segmented as a 7-mm diameter cylindrical volume from the iliac crest.

**Results:** Two-way ANOVA revealed significant differences in microarchitecture across anatomical sites, e.g. BV/TV at the biopsy site was significantly higher than at radius (p=0.002), femoral neck (p=0.005) and vertebra (p<0.0001). Age-related microarchitectural changes were observed, e.g. -34% BV/TV at biopsy site or -42% BV/TV at femoral neck between middle-age and elderly groups. Biopsy site microarchitecture correlated strongly with that at vertebrae, except for Tb.Th (BV/TV: r=0.79, p=0.0003; Tb.Sp: r=0.78, p=0.0003; Tb.N: r=0.63, p=0.009), moderately with the femoral neck for

BV/TV only (r=0.68, p=0.004), but not with the radius. In contrast, the radius correlated strongly with the femoral neck (BV/TV: r=0.82, p=0.0001; Tb.Th: r=0.70, p=0.003; Tb.Sp: r=0.86, p<0.0001; Tb.N: r=0.78, p=0.0004), but not with vertebrae.

**Conclusions:** Trabecular bone microarchitecture differs across skeletal sites and changes with age. The biopsy site predicts vertebral microarchitecture, but not that of femoral neck and radius which are mutually correlated. Our findings show the limitations of the transiliac bone biopsy for fracture risk prediction of cortical bone dominated regions.

**Figure 1:** Analyzed anatomical samples

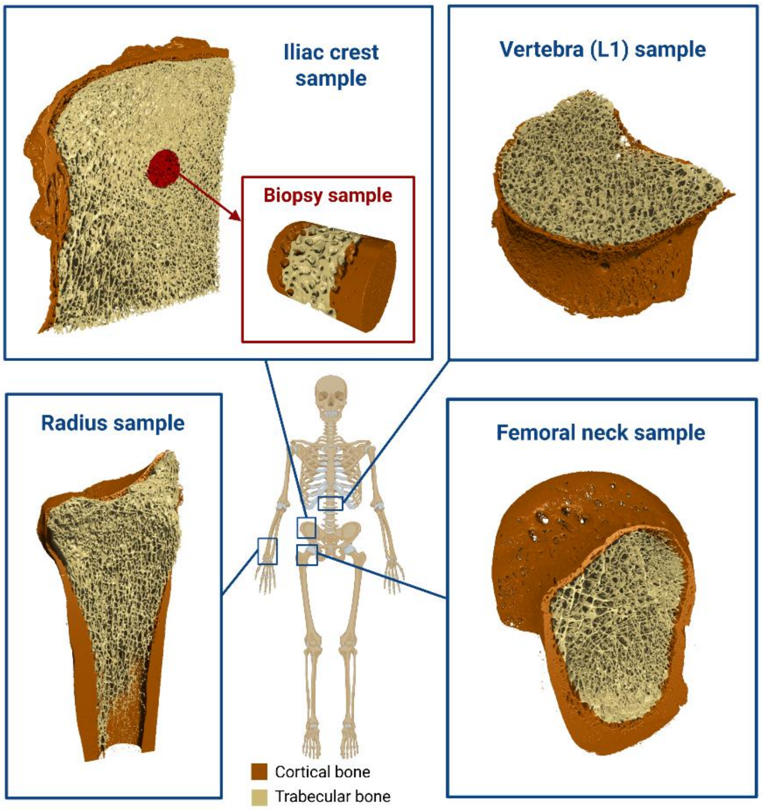

**P026**

**Drifting osteons in relation to occlusal loading in adult human mid-facial skeleton: preliminary study**

*<u>Jelena Jadzic</u>[1], Natasa Janovic[1], Igor Saveljic[2], Arso Vukicevic[3], Dalibor Nikolic[2], Gordana Jovicic[3], Nenad Filipovic[2], Petar Milovanovic[1], Bjorn Busse[4], Michael Hahn[4], Michael Amling[4], Marija Djuric[1], Aleksa Janovic[5]*

*[1]Faculty of Medicine- University of Belgrade, Center of Bone Biology, Belgrade, Serbia*

*[2]Faculty of Engineering- University of Kragujevac,*

*Bioengineering Research and Development Center BioIRC, Kragujevac, Serbia*

*[3]Univeristy of Kragujevac, Faculty of Engineering, Kragujevac, Serbia*

*[4]University Medical Center Hamburg-Eppendorf, Department of Osteology and Biomechanics,*

*Hamburg, Germany*
[5]*Faculty of Dental Medicine- University of Belgrade, Center for Diagnostic Radiology, Belgrade, Serbia*

**Abstract Text**

**Background:** Drifting osteons are a secondary osteonal morphotype characterized by a hemicyclical tail of lamellae trailing the Haversian canal that has been recently investigated in the human postcranial skeleton. However, the relationship between drifting osteons and the local mechanical environment has not been studied in the human mid-facial skeleton to date.

**Purpose:** Concerning the impact of occlusal forces on the regional variations in the cortical mechano-structural properties of facial bones, we aimed to conduct a histomorphometric assessment of drifting osteons in relation to the magnitude of occlusal loading and the type of stress in the adult human mid-facial skeleton.

**Methods:** Based on the results of our previous finite element analysis on Von Mises stress (VMS) during clenching in the mid-facial bones of a fully dentate human skull, a total of 25 facial bone samples were divided into a high-stress group (VMS ranging from 2.25 MPa to 2.75 MPa, $n = 7$) and a low-stress group (VMS below 1.49 MPa, $n = 18$). These bone samples were subjected to Von Kossa-modified staining, and histomorphometric analysis of the percentage and density of drifting osteons in cortical bone was conducted, following institutional ethics committee approval (no. 29/I-10).

**Results:** Despite a trend towards a lower percentage (60.89 ± 15.62% vs. 66.32 ± 14.85%) and the density of drifting osteons (3.43 ± 0.38/$mm^2$ vs. 3.17 ± 0.20/$mm^2$) in cortical bone of the high stress group samples compared to the low stress group, inter-group differences did not reach statistical significance ($p > 0.05$). Still, our data revealed a mild positive correlation between the percentage of drifting osteons and the degree of compressive stress ($r_s = 0.407$, $p = 0.044$).

**Conclusions:** Our study revealed that the density of drifting osteons may correlate with compressive loading in the adult human mid-facial skeleton, warranting further research.

**P027**

**Impact of Dietary Glutamine on Laying Performance and Bone Characteristics in Quail**

*Ewa Tomaszewska*[1], *Siemowit Muszyński*[2], *Iwona Puzio*[1], *Katarzyna Kras*[3], *Cezary Osiak-Wicha*[3], *Marcin Arciszewski*[3]
[1]*University of Life Sciences in Lublin, Department of Animal Physiology, Lublin, Poland*
[2]*University of Life Sciences in Lublin, Department of Biophysics, Lublin, Poland*
[3]*University of Life Sciences in Lublin, Department of Animal Anatomy and Histology, Lublin, Poland*

**Abstract Text**

Glutamine (Gln) is an endogenous, conditionally essential amino acid that plays a crucial role in animal nutrition. It is a pivotal nutrient in the diets of chickens and other avian species, supporting optimal growth, production efficiency, and overall health. This study investigated the effects of dietary Gln supplementation on tibia properties in laying quail over a 12-week period, with analyses conducted after 6 and 12 weeks of supplementation.

Japanese quails typically begin laying eggs at approximately 7 weeks of age, reach peak egg production by 12 weeks (i.e., 6 weeks after the onset of laying), and experience a decline in laying performance by 17 weeks of age (12 weeks after the onset of laying). Seven-week-old dual-purpose female quails at the onset of lay were assigned to diets containing 0% (control), 0.5%, 1%, or 1.5% Gln.

Dietary Gln supplementation in laying quail exerted differential effects on production performance and bone quality. Supplementation with 0.5% Gln significantly enhanced the laying rate after 12 weeks, while 1.5% inclusion increased breast muscle weight after 6 weeks. However, the mechanical and geometric properties of the tibia remained unchanged. Nevertheless, glutamine supplementation increased tibial calcium content in a dose-dependent manner.

These findings suggest that while Gln can improve certain production parameters, its influence on bone integrity may be limited. Further research is warranted to optimize dietary Gln levels that maximize production benefits without compromising skeletal health in laying quail.

**P028**

**Location and density of rib cortical microcracks from dynamic loading are related to aging and peak force**

*Gülislam Agacan[1], Randee Hunter[2], Mary Elizabeth Cole[2], Amanda Agnew[2], Yun-Seok Kang[2], Thomas Levin Andersen[3], Christina Andreasen[4]*
*[1]Aarhus University, Molecular Bone Histology lab- Dept of Forensics, Aarhus, Denmark*
*[2]The Ohio State University, Skeletal Biology Research lab- Injury Biomechanics Research Center, Ohio, United States*
*[3]Aarhus University, Molecular Bone Histology Laboratory- Dept. of Forensics, Aarhus, Denmark*
*[4]University of Southern Denmark, Research Unit of Pathology- Department of Clinical Research, Odense M, Denmark*

**Abstract Text**

Elderly individuals often have an increased risk of rib fractures during motor vehicle crashes. This may not only be explained by reduced bone mass but also changes in the capacity of the cortical bone matrix to dissipate energy.

This study examined how aging and rib mechanical properties are associated with the location and propagation of microcracks in rib cortical bone following dynamic loading. Osteonal cement lines normally act as protective barriers, deflecting microcracks along borders or into interstitial bone, and we investigated whether this persists with age. Specifically, differences between interstitial and osteonal bone, as well as osteonal remodeling type were examined.

Peak reaction force during dynamic loading to failure (A-P) was recorded for sixth ribs from 23 postmortem human subjects (22–89 years). A 2-cm transverse segment adjacent to the fracture was stained with 1% basic fuchsin, MMA-embedded, sectioned, and imaged at a 100x magnification. Cortical microcracks were analyzed for location, number, length, and density with particular focus to bone type and cement line penetration.

Rib cortical thickness was significantly less in elderly (>50 years) than younger donors (p=0.005). Elderly ribs had more microcracks (p=0.01) but with shorter length (p=0.013) and a higher prevalence in interstitial bone (p=0.043) than younger ribs. Older donors also had more microcracks crossing the cement line of type 2 remodeling events, though location and cement line penetration were similar. Microcrack number and length were unrelated to peak force, but crack density was lower in ribs experiencing a higher peak force (p=0.032).

Aging reduces rib cortical thickness and increases the number of microcracks, which are shorter and more prevalent in interstitial than osteonal bone. Peak force did not correlate with crack number or length but was negatively associated with microcrack density. This suggests a complex relationship between aging, resistant to fracture, age and microcrack accumulation in dynamic loading events.

**P029**

**Coupled Bone-Muscle Degeneration in Juvenile Pigs with Cerulein-Induced Chronic Pancreatitis: A Model of Secondary Osteosarcopenia**

*Iwona Puzio*[1], *Ewa Tomaszewska*[1], *Siemowit Muszyński*[2], *Marcin Arciszewski*[3]
[1]*University of Life Sciences in Lublin, Department of Animal Physiology, Lublin, Poland*
[2]*University of Life Sciences in Lublin, Department of Biophysics, Lublin, Poland*
[3]*University of Life Sciences in Lublin, Department of Animal Anatomy and Histology, Lublin, Poland*

**Abstract Text**

Osteosarcopenia, defined as the coexistence of bone loss and muscle wasting, is a debilitating condition that compromises mobility and overall health. While primary, age-related osteosarcopenia has been extensively studied, its secondary form associated with chronic diseases is poorly characterized, particularly in young individuals. This study investigated musculoskeletal alterations in a juvenile porcine model of cerulein-induced chronic pancreatitis over a 7-week period.

Ten growing pigs, 9–10 weeks old, were randomly assigned to control and experimental groups. Chronic pancreatitis was induced in the experimental group via repeated cerulein administration, after which femoral bones and quadriceps femoris muscles were subjected to densitometric, mechanical, and histological analyses, alongside serum bone turnover markers and muscle gene expression profiling.

Pigs with secondary osteosarcopenia exhibited significantly reduced femoral bone mineral density and content, altered cortical geometry, compromised elastic properties, and trabecular deterioration with impaired growth plate morphology. Circulating bone turnover markers indicated elevated resorption and disrupted mineralization. Muscle analysis revealed sarcopenic features characterized by selective atrophy of fast-twitch type II fibers and increased fiber density. At the molecular level, quadriceps muscles showed downregulation of catalase and CASP3 expression, suggesting that oxidative stress and caspase-independent pathways drive muscle atrophy rather than classical apoptosis.

These findings demonstrate that chronic pancreatitis in young pigs induces coupled bone–muscle degeneration consistent with secondary osteosarcopenia. The study highlights the early onset of bone–muscle crosstalk dysfunction in chronic inflammatory disease and provides a translational model for investigating integrated musculoskeletal decline in young individuals.

**P030**

**Effects of Dietary Vitamin D Supplementation on Bone Microarchitecture, Mineralization, and Mechanical Properties in Wistar Rat Animal Model**

*Cezary Osiak-Wicha*[1], *Siemowit Muszyński*[2], *Katarzyna Kras*[1], *Ewa Tomaszewska*[3], *Beata Szymczyk*[4], *Maria Oczkowicz*[5], *Małgorzata Świątkiewicz*[5], *Dariusz Wiącek*[6], *Daniel Kamiński*[7], *Marcin Arciszewski*[1]
[1]*University of Life Sciences in Lublin, Department of Animal Anatomy and Histology, Lublin, Poland*
[2]*University of Life Sciences in Lublin, Department of Biophysics, Lublin, Poland*
[3]*University of Life Sciences in Lublin, Department of Animal Physiology, Lublin, Poland*
[4]*National Research Institute of Animal Production, Department of Animal Nutrition and Feed Science, Balice, Poland*
[5]*National Research Institute of Animal Production, Department of Animal Molecular Biology, Balice, Poland*
[6]*Bohdan Dobrzański Institute of Agrophysics of the Polish Academy of Sciences, Department of Physical Properties of Plant Materials, Lublin, Poland*
[7]*Maria Curie-Sklodowska University, Department of Organic and Crystallochemistry, Lublin, Poland*

**Abstract Text**

Vitamin D is essential for skeletal development, but how different dietary doses during late growth affect bone quality remains unclear. We investigated how three dietary vitamin D3 levels influence bone properties in growing Wistar rats. Thirty-six rats (18 males, 18 females) were randomized ($n$ = 6/sex/dose) to receive 0, 1000, or 5000 IU/kg vitamin D3 in the diet for 12 weeks. Femoral bone mineral density (BMD; DXA), three-point bending mechanics, growth plate and metaphyseal histomorphometry, bone mineral composition (ICP-OES, Ca/P), and serum and tissue markers of bone turnover were assessed. BMD was lower at 0 IU/kg than at 1000 and 5000 IU/kg (dose effect $p < 0.001$), with no difference between supplemented groups. In males, bone mass was higher at 1000 IU/kg than at 0 IU/kg ($p = 0.004$) and 5000 IU/kg ($p = 0.025$). Breaking force (Fmax) was greater in supplemented versus deficient males ($p = 0.011$ and $p < 0.01$ for 1000 and 5000 IU/kg), but work to fracture (Wmax) increased only at 1000 IU/kg ($p = 0.003$). Breaking strain (εmax) was highest at 1000 IU/kg in both sexes (dose effect $p = 0.001$; pairwise $p < 0.001$ vs 0 and 5000 IU/kg). Vitamin D deficiency reduced Ca/P ratio and metaphyseal BV/TV and trabecular number versus 1000 IU/kg ($p \leq 0.016$), consistent with impaired endochondral ossification. The RANKL/OPG ratio was highest in 1000 IU/kg males ($p < 0.001$) and lowest in 5000 IU/kg females ($p \leq 0.017$), indicating sex- and dosedependent remodeling responses. Moderate dietary vitamin D3 (1000 IU/kg) produced the most consistent improvements in bone mass, mechanical competence, microarchitecture, and mineral quality during late growth, whereas deficiency compromised multiple skeletal endpoints and 5000 IU/kg conferred no consistent additional benefit and in some respects resembled deficiency.

**P031**

**Bone Impairment Associated with PTSD and the Therapeutic Potential of CE-123**

*Marcin Arciszewski*[1], *Katarzyna Kras*[1], *Ewa Tomaszewska*[2], *Siemowit Muszyński*[3], *Paweł Grochecki*[4], *Jolanta Kotlińska*[4], *Słowik Tymoteusz*[5], *Świetlicki Michał*[6], *Cezary Osiak-Wicha*[1]
[1]*University of Life Sciences in Lublin, Department of Animal Anatomy and Histology, Lublin, Poland*
[2]*University of Life Sciences in Lublin, Department of Animal Physiology, Lublin, Poland*
[3]*University of Life Sciences in Lublin, Department of Biophysics, Lublin, Poland*
[4]*Medical University of Lublin, Department of Pharmacology and Pharmacodynamics, Lublin, Poland*
[5]*Medical University, Experimental Medicine Center, Lublin, Poland*
[6]*Lublin University of Technology, Department of Applied Physics, Lublin, Poland*

**Abstract Text**

**Background**: Post-Traumatic Stress Disorder (PTSD) is a psychiatric condition characterized by intrusive memories, hyperarousal, and avoidance. Beyond psychological symptoms, PTSD has been linked to systemic alterations, including disturbances in bone metabolism, which may increase susceptibility to fractures.
**Purpose**: This study aimed to investigate the impact of PTSD on bone structural and mechanical properties in rats and to evaluate the potential protective role of CE-123, a novel dopamine transporter (DAT) inhibitor.
**Methods**: PTSD was induced using a fear-conditioning paradigm combining electric shocks with auditory cues. Forty-eight female Wistar rats were randomly assigned to four groups: control, PTSD, control+CE-123, and PTSD+CE-123. CE-123 (10 mg/kg b.w.) was administered intraperitoneally. Tibial bone characteristics were analyzed by dual-energy X-ray absorptiometry (DXA), mechanical testing, and histomorphometry. Statistical analyses included ANOVA and Kruskal–Wallis tests. **Results**: PTSD significantly decreased cortical index, relative wall thickness, and Young's modulus, indicating impaired bone integrity. Mechanical evaluation confirmed reduced yield stress in PTSD animals. CE-123 administration attenuated several PTSD-related skeletal changes, enhancing bone mineral content, cortical structure, and mechanical resistance ($W_{yield}$, $F_{max}$, $W_{max}$). Moreover, CE-123 modified growth plate morphology, expanding the proliferative zone in control animals while reducing it in PTSD rats.
**Conclusions**: PTSD negatively affects bone quality and strength, potentially elevating fracture risk. CE-123 demonstrated protective effects on mineralization, cortical architecture, and mechanical performance, highlighting dopaminergic modulation as a promising approach to counteract stress-related skeletal deterioration. These findings support further research into pharmacological strategies addressing bone impairment in trauma-exposed individuals and underscore the systemic nature of PTSD.

**P032**

**Demographic and clinical differences between individuals with and without DXA scans in Denmark**

*Annika Kvist*[1], *Sally Søgaard Andersen*[1,2], *Mia Daugaard Madsen*[1,2], *Rikke Viggers*[1,2,3], *Peter Vestergaard*[1,2,3]
[1]*Aalborg University Hospital, Steno Diabetes Center North Denmark, Gistrup, Denmark*
[2]*Aalborg University, Department of Clinical Medicine, Gistrup, Denmark*
[3]*Aalborg University Hospital, Department of Endocrinology, Aalborg, Denmark*

**Abstract Text**

**Introduction:** An increased focus on osteoporosis in Denmark has led to more research using DXA scan cohorts. Thus, identifying differences between DXA recipients and the general population is essential to detect potential selection bias, and this was the aim of this study.

**Methods:** We used two large population-based project databases from Statistics Denmark (Fracture[Fx] and Diabetes[DM] cohorts) to identify cases as individuals who received a DXA scan between primo 2000, and ultimo 2021. Individuals who received their first DXA scan before the age of 50 years were excluded. Cases were matched on sex and age to highest possible number of individuals who have never received a DXA scan.

Descriptive statistics for several factors were summarized prior to or at index. Furthermore, the analyses were performed stratified by time periods (2000-2010 and 2011-2021).

**Results:** We identified 379,686 cases and 1,571,452 controls from Fx, and 350,817 cases and 1,747,021 controls from DM. In both databases, we found differences between cases and controls in multiple factors, including (but not limited to) female sex(82.2% vs. 52.4% [SMD=0.67] and 73.7% vs. 40.8% [SMD=0.71]), glucocorticoid use(51.9% vs. 26.9% [SMD=0.53] and 39.2% vs. 17.5% [SMD=0.50]), hormone replacement therapy(51.0% vs. 21.1% [SMD=0.66] and 38.8% vs. 13.5% [SMD=0.60]), antiosteoporosis medication(47.6% vs. 3.5% [SMD=1.18] and 8.9% vs. 0.9% [SMD=0.38]), prior fractures(47.1% vs. 33.6% [SMD=0.28] and 36.4% vs. 18.9% [SMD=0.40]), smoking(14.9% vs. 7.8% [SMD=0.23] and 13.4% vs. 5.6% [SMD=0.27]), COPD(10.2% vs. 3.5% [SMD=0.27] and 10.8% vs. 3.5% [SMD=0.29]), cardiovascular diseases(39.7% vs. 27.8% [SMD=0.26] and 41.6% vs. 29.3% [SMD=0.26]), and antidepressive medication(44.6% vs. 29.6% [SMD=0.32] and 51.2% vs. 33.5% [SMD=0.37]). Moreover, the differences were smaller in the last period.

**Conclusion:** Systematic differences exist between individuals who undergo DXA scanning and those who do not. Over time, there appears to have been a progressive improvement in the referral of high-risk patients for DXA assessment.

**P033**

**Mandibular Growth and Biomechanics in Juvenile Wistar Rats Following Exogenous Irisin**

**Administration**

Katarzyna Kras[1], Siemowit Muszyński[2], Ewa Tomaszewska[3], Ligia Janicka[1], Aleksandra Dajnowska[1], Anna Arczewska-Włosek[4], Sylwester Świątkiewicz[4], Marcin Arciszewski[1], Cezary Osiak-Wicha[1]
[1]University of Life Sciences in Lublin, Department of Animal Anatomy and Histology, Lublin, Poland
[2]University of Life Sciences in Lublin, Department of Biophysics, Lublin, Poland
[3]University of Life Sciences in Lublin, Department of Animal Physiology, Lublin, Poland
[4]National Research Institute of Animal Production, Department of Animal Nutrition and Feed Science, Balice, Poland

**Abstract Text**

**Background:** Irisin, a myokine secreted predominantly in response to exercise, has been widely studied for its metabolic, thermogenic, and osteogenic effects in skeletal muscle and long bones. However, craniofacial bones such as the mandible are exposed to distinct functional loading patterns dominated by masticatory forces rather than gravitational strain, and little is known about irisin's role in their development.
**Purpose:** This study investigated the effects of exogenous irisin on mandibular structure, biomechanics, and growth dynamics in juvenile Wistar rats, with attention to potential sex-specific responses.
**Methods:** Three-week-old rats were divided into two groups: one received weekly intraperitoneal injections of irisin for four weeks, and controls received saline. Mandibles were analyzed by micro-computed tomography (micro-CT) for cortical and trabecular architecture, while mechanical properties were assessed by three-point bending tests (ZwickRoell 010). Mineral apposition and growth patterns were evaluated using calcein double labeling.
**Results:** Irisin administration altered mandibular phenotype in a sex-dependent manner. Both sexes exhibited reduced mandibular bone weight compared with controls. In females, irisin increased stiffness and yield force but decreased cortical thickness and markedly reduced work-to-fracture. In males, irisin suppressed buccal cortical wall growth and limited expansion of the canine alveolus, whereas in females it promoted alveolar growth in the same region. **Conclusions:** Exogenous irisin modifies mandibular growth and biomechanics in juvenile rats, with clear sex-specific effects. Unlike long bones, the mandible—characterized by non-weight-bearing but high functional loading—responds differently to systemic irisin, underscoring the need for further studies on its role in craniofacial skeletal development.

**P034**

**Classification of Bone Demineralization Patterns with HR-pQCT Texture Metrics and Derived Scores: Bone Microarchitectural Phenotypes as Indicators of Metabolic Disease**

Johannes Bereiter-Payr[1,2], Thomas Ausserer[1], Sonja Wagner[3,4], Martina Saretto[3,4], Heinz Zoller[3,4], Gerald Degenhart[1]
[1]Medical University of Innsbruck, University Clinic for Radiology- Core Facility Micro-CT, Innsbruck, Austria
[2]VASCage - Center on Clinical Stroke Research, Data Science, Innsbruck, Austria

*[3]Medical University of Innsbruck,*
*Department of Medicine I- Gastroenterology- Hepatology and Endocrinology, Innsbruck, Austria*
*[4]Medical University of Innsbruck, Christian Doppler Laboratory on Iron and Phosphate Biology, Innsbruck, Austria*

**Abstract Text**

**Introduction/Purpose:** We report ongoing work to quantify distinguishable patterns of bone demineralization in patients with metabolic and/or cardiovascular disease. This effort was motivated by (1) the consistent visual observation of distinct microarchitectural differences in HR-pQCT images of distal extremities within our study cohorts, and (2) previously published HR-pQCT phenotype classifications related to fracture risk by Whittier, Boyd and colleagues [1].

**Methods:** High-resolution peripheral quantitative CT (HR-pQCT) images of the distal radius were acquired in 192 patients from two clinical cohorts using an XCT-2 system (Scanco Medical AG), with 4–6 scans per patient over 12 months. Bone texture analysis (TA) was performed according to a standardized protocol provided by the manufacturer. For each patient, mean texture parameters across all examinations were normalized for age and sex using statistical models described by Warden et al. [2]. Normalized metrics were evaluated using principal component analysis (PCA) and fuzzy c-means clustering (FCM) to identify distinct (de-)mineralization patterns. Data were cross-referenced with areal BMD (aBMD) from DXA when available, or with projected mineral density derived from HR-pQCT.

**Results:** Among patients with aBMD values in the osteopenic range or showing early signs of demineralization, three potential phenotypes can be described: (1) generalized demineralization, (2) predominant trabecular deterioration, and (3) selective thinning of the distal cortical compartment with relatively preserved trabecular structure. Based on these findings, we propose a preliminary compartment-specific composite score to quantify individual demineralization patterns.

**Discussion:** HR-pQCT-derived structural markers can detect early alterations in bone mineralization prior to substantial changes in fracture risk. Some metabolic diseases appear to exhibit characteristic demineralization signatures. Accordingly, classification by the proposed scoring tool may serve as an indicator of bone involvement or may be useful to support the diagnosis suspected disease.

**References:**

[1] Whittier DE et al., J Bone Miner Res, 2020.
[2] Warden SJ et al., Osteoporos Int, 2022.

**P035**

### Characterization of the osteochondral phenotype in human and murine classical osteogenesis imperfecta

*<u>Sebastian Matzke</u>[1], Ana  Ocokoljic[1], Timur A. Yorgan[2], Ralf Oheim[2], Frank Timo Beil[1], Assil-Ramin Alimy[1], Tim Rolvien[1]*

[1]*University Medical Center Hamburg-Eppendorf, Department of Trauma and Orthopedic Surgery, Hamburg, Germany*
[2]*University Medical Center Hamburg-Eppendorf, Department of Osteology and Biomechanics, Hamburg, Germany*

**Abstract Text**

**Background:** Osteogenesis imperfecta (OI) is associated with an increased risk of early-onset osteoarthritis (OA), but it remains unclear whether underlying osteochondral changes are responsible for this.

**Purpose:** We aimed to characterize the osteochondral phenotype in OI to identify potential bone-driven mechanisms that may predispose to OA.

**Methods:** We analyzed bilateral tibial plateaus from an adult with classical OI undergoing total knee arthroplasty using histology and quantitative backscattered electron imaging (qBEI). Data were compared to samples from ten patients with primary OA. In addition, we examined hip and knee joints of six-week-old *Col1a2*$^{oim/oim}$ mice and wild-type (WT) controls by µCT, histology, and qBEI.

**Results:** In human specimens, overall osteochondral morphology was comparable between OI and primary OA, but OI subchondral bone showed a higher and more heterogeneous mineralization. *Col1a2*$^{oim/oim}$ mice exhibited a pronounced low bone mass phenotype in the hip (BV/TV, $p<0.001$) and knee joints (Fig.1A, 1B), while growth plate architecture and articular cartilage thickness were preserved. However, the subchondral and trabecular bone were characterized by a hyper- and heterogeneously mineralized matrix with increased osteocyte lacunar density. Moreover, the osteocyte lacunocanalicular network was markedly disrupted, with shorter canaliculi (WT: 3.9 ± 0.8 µm vs. *Col1a2*$^{oim/oim}$: 2.5 ± 0.3 µm, $p<0.001$) and fewer canaliculi per osteocyte.

**Conclusion:** Our findings indicate that type I collagen defects primarily impair subchondral bone matrix quality and osteocyte connectivity rather than causing early cartilage loss, supporting a bone-driven pathway of joint degeneration that may underlie the increased susceptibility to OA in OI.

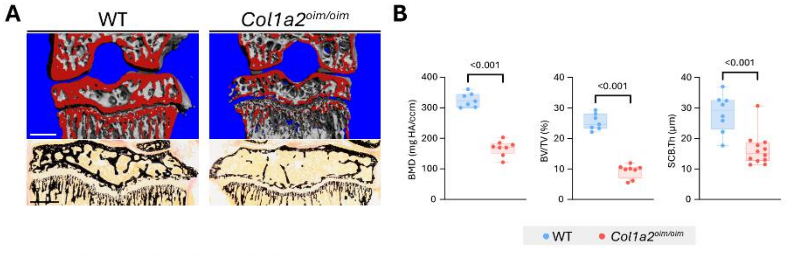

*Col1a2*$^{oim/oim}$ mice exhibit a subchondral low bone mass phenotype.
A) Representative three-dimensional µCT reconstructions (upper panels) and von Kossa-stained sections (lower panels) of the knee joint from wild-type (WT) and Col1a2oim/oim mice. Scale bars: 500 µm. B) Quantification of bone mineral density (BMD), bone volume fraction (BV/TV), and subchondral bone plate thickness (SCB.Th) in WT and Col1a2oim/oim mice (n≥7).
Statistical analysis was conducted using the Student's t-test or the Mann-Whitney U test. Exact p-values are shown for statistically significant comparisons, unless p < 0.001.

**P036**

**Vascular-Driven Aspects Contributing to Cortical Bone Structure**

Jannik Volker Theiding[1], Johannes Krug[1], Benjamin Ondruschka[2], Klaus Püschel[2], Michael Amling[1], Björn Busse[1], Felix Nikolai von Brackel[1]
[1]University Medical Center Hamburg-Eppendorf, Department of Osteology and Biomechanics, Hamburg, Germany
[2]University Medical Center Hamburg-Eppendorf, Institute of Legal Medicine, Hamburg, Germany

**Abstract Text**

**Introduction:** Arteriosclerosis is common and frequently coexists with low bone mass, a relationship often referred to as the calcification paradox. However, whether vascular calcification directly affects cortical bone quality remains uncertain. Because cortical bone depends on an intact vascular supply, it may be influenced by impaired arterial perfusion.

**Purpose:** To determine whether volumetric arteriosclerotic calcifications in the heart, abdominal aorta, and lower extremities are associated with cortical bone quality assessed by HR-pQCT and microCT.

**Methods:** Fifty-nine human tibiae obtained from autopsy (32 male, 27 female, mean age 59 ± 17 years; ethics approved) were analyzed. Arteriosclerotic burden was quantified in full-body CT using threshold-based segmentation of calcified plaque in cardiac, aortic, and lower-limb arteries. HR-pQCT (XtremeCT II, 60.7 µm voxel size) was performed in the tibial metaphysis and diaphysis to assess Ct.Po, cortical thickness (Ct.Th), volumetric BMD (vBMD), and trabecular BV/TV. Cortical porosity in the diaphysis was additionally quantified using microCT. Correlation analyses and group comparisons were conducted.

**Results:** Individuals with arteriosclerosis were significantly older than controls (68 ± 14 vs. 45 ± 12 years, $p<0.001$). vBMD in the diaphysis was lower in the arteriosclerosis group ($p=0.006$), though age strongly influenced bone parameters. MicroCT-derived Ct.Po showed a marked positive correlation with age ($r=0.686$, $p<0.001$). A borderline association was observed between HR-pQCT Ct.Po and calcified cardiac volume ($r=0.42$, $p=0.06$), while calcifications in the aorta showed no detectable relationship with microCT Ct.Po ($p=0.12$).

**Conclusion:** Lower vBMD in individuals with arteriosclerosis appears predominantly age-related rather than directly linked to vascular calcification. Only cardiac calcification showed a weak trend toward higher cortical porosity. These findings suggest limited bone-vascular coupling in this small cohort, while leaving open the possibility that the calcification paradox reflects more complex or cohort-specific mechanisms detectable in larger studies or other locations.

**P037**

**Structural porosity and low mineral density in enamel rods drive molar incisor hypomineralisation**

Johannes Krug[1,2], Katharina Jähn-Rickert[1,2], Gudrun Lotze[3,4], Imke A.K. Fiedler[1,2], Diana Freund[1,2], Ezgi Deniz Türkoğlu[5], Christine Plumeyer[1,2], Felix N. von Brackel[1,2], Anton Davydok[6], Manfred Burghammer[7], Thomas Beikler[5], Thomas F. Keller[8], Katrin Bekes[9], Elizabeth A. Zimmermann[10], Björn Busse[1,2]

[1]University Medical Center Hamburg-Eppendorf, Department of Osteology and Biomechanics, Hamburg, Germany
[2]University Medical Center Hamburg-Eppendorf,
Interdisciplinary Competence Center for Interface Research, Hamburg, Germany
[3]Uppsala University, Department of Chemistry - Ångström Laboratory, Uppsala, Sweden
[4]IGDORE, Institute for Globally Distributed Open Research and Education, Göteborg, Sweden
[5]University Medical Center Hamburg-Eppendorf,
Department of Periodontics- Preventive and Restorative Dentistry, Hamburg, Germany
[6]Helmholtz Zentrum Hereon, Institute of Materials Physics, Hamburg, Germany
[7]ESRF, The European Synchrotron, Grenoble, France
[8]Deutsches Elektronen-Synchrotron DESY, Centre for X-ray and Nano Science CXNS, Hamburg, Germany
[9]Medical University of Vienna, Department of Pediatric Dentistry at the University Clinic of Dentistry, Vienna, Austria
[10]McGill University, Faculty of Dental Medicine and Oral Health Sciences, Hamburg, Germany

**Abstract Text**

**Background:** Enamel protects against wear during mastication and resists chemical degradation. In the condition termed molar incisor hypomineralisation (MIH), impaired enamel quality leads to rapid tooth decay, hypersensitivity, and frequent need for early tooth extraction. MIH is highly prevalent in children, affecting approximately 14.2% of the global population, yet the mechanisms underlying the accelerated decay and structural breakdown of the enamel crown remain unclear.
**Purpose:** To enhance the understanding of MIH related enamel breakdown through material characterization on multiple length-scales.
**Methods:** Enamel of healthy and MIH affected teeth inside and sound enamel outside of MIH lesions was investigated on a microscopic level utilizing micro-computed tomography, quantitative backscattered electron microscopy, Raman spectroscopy, histology and polarized light microscopy. Subsequently, nano-scale investigations of morphology and composition through focused ion-beam milling scanning electron microscopy, wide angle x-ray diffraction, atomic force microscopy and energy-dispersive x-ray spectroscopy were conducted.
**Results:** MIH lesions thereby presented with lower calcium weight% (-4.3%, -4.2%), but higher protein content ($CH_2$-deformation/phosphate ratio, +3,563%, +1,639%) compared to healthy and MIH sound enamel, respectively.
High-resolution analyses revealed enlarged prims sheath regions around the enamel rods in MIH lesions compared to healthy (+683%) and sound enamel (+1,700%), consistent with a higher carbon content in these areas. Additionally, hydroxyapatite crystal arrangement and mechanical properties were altered in the interrod region of MIH affected, but not unaffected enamel.
**Conclusion:** The findings highlight that MIH involves coordinated compositional and microstructural alterations across multiple length scales, underscoring the need for scale-dependent assessment to guide the development of effective preventive and therapeutic strategies.

**P038**

**Structural and Material-Level Fragility Mechanisms in Glucocorticoid-Induced Bone Loss**

Praveer Sihota[1], Xenia Junimann[1], Johannes Krug[1], Jan Frischmuth[1], Lukas Harms[1], Timur A. Yorgan[1], Imke Fiedler[1], Katharina Jähn-Rickert[1], Annegreet Vlug[2], Björn Jobke[1], Michael Amling[1], Mathias Werner[3], Björn Busse[1]
[1]University Medical Center Hamburg-Eppendorf- Hamburg, Department of Osteology and Biomechanics, Hamburg, Germany
[2]Leiden University Medical Center, Centre for Bone Quality, Leiden, Netherlands
[3]Institute of Pathology- Friedrichshain Hospital- Osteopathology Reference Center, Vivantes Network for Health GmbH, Berlin, Germany

**Abstract Text**

**Background:** GCs are known to reduce bone mass and increase fracture risk; however, the impact of GC treatment on bone material quality at the tissue level remains poorly understood.
**Purpose:** The purpose of this study is to improve our understanding of GC-induced skeletal fragility.
**Method**: This retrospective case-control study evaluated *ex vivo* multi-scale trabecular bone quality parameters in iliac crest biopsies from patients with established glucocorticoid-induced osteoporosis. We analyzed n = 21 bone biopsies obtained from patients with chronic inflammatory or respiratory disorders under long-term GC therapy (≥ 4 mg/day, ≥ 6 months), and compared them to n = 13 age-matched control biopsies.
**Result**: In the GIOP group, micro-CT analysis revealed a significant reduction in bone volume fraction ($p < 0.001$), primarily due to trabecular thinning ($p = 0.002$). Histomorphometry indicated reduced bone formation, evidenced by lower osteoblast surface ($p = 0.002$) and osteoid thickness ($p < 0.001$). Higher erosion depth ($p = 0.011$) suggested increased osteoclast activity. Furthermore, lower viable osteocyte density ($p = 0.005$) and higher marrow adiposity ($p = 0.011$) were observed. Bone composition analysis showed elevated calcium content ($p < 0.001$), higher mineral homogeneity ($p = 0.007$), and lower proportion of low-mineralized bone ($p = 0.004$). Raman spectroscopy demonstrated decreased collagen cross-linking, reflected by lower relative pyridinoline ($p = 0.015$) and lower glycosaminoglycan levels ($p < 0.001$). Nanoindentation revealed lower elastic modulus ($p = 0.037$).
**Conclusion**: Glucocorticoid therapy induces multifaceted bone quality impairment, providing mechanistic insight into the increased skeletal fragility in GIOP.

**Table 1.** Baseline demographic parameters of the control and GIOP groups

| Parameters | Control (n = 13) | GIOP (n = 21) | p value |
|---|---|---|---|
| Female, n (%) | 6 (54%) | 14 (67%) | 0.238 |
| Age (years) | 52.5 ± 5.9 [41 61] | 60.4 ± 17.0 [27 85] | 0.061 |
| Bone fractures following GC administration | none | 7 (33%) | |

**P039**

**Piezo1 deficiency selectively reduces thickness and mineralization in loaded but not non loaded cranial bone in mice**

Bjarne Sahling[1], Johannes Krug[1], Michael Amling[1], Björn Busse[1], Felix N. von Brackel[1]
[1]University Medical Center Hamburg-Eppendorf, Osteology and Biomechanics, Hamburg, Germany

**Abstract Text**

**Background**: Bone remodeling is a mechanosensitive process orchestrated by osteocytes, but the mechanisms of load sensing remain incompletely understood. Piezo1, a mechanosensitive ion channel, has emerged as a key candidate. Previous whole-skull analyses of Piezo1 knockout mice reported no phenotype. We suggest that regional differences may have been masked and therefore investigated site-specific effects of Piezo1 deletion in cranial bone.
**Methods**: Skulls from 12-week-old conditional Piezo1$^{Dmp1Cre}$ knockout mice and floxed controls (Piezo1$^{fl}$) were analyzed. The unique anatomy of the calvarium enables direct comparison of non loaded parietal bone, lacking muscle attachment, with loaded temporal bone, subjected to masticatory forces. High-resolution µCT imaging (10 µm voxel size) was used to quantify calvarial thickness, calvarial porosity, bone mineral density (BMD), and tissue mineral density (TMD).
**Results**: Temporal bone showed pronounced alterations:  Piezo1 knockout mice exhibited ~25% reduced thickness (0.09 ± 0.01 vs. 0.12 ± 0.02 mm, p = 0.0046) and significantly lower TMD (786 ± 21 vs. 809 ± 24 mgHA/ccm, p = 0.036). In contrast, parietal bone did not differ between groups in thickness (0.14 ± 0.02 vs. 0.15 ± 0.01 mm) or mineral density (TMD: 822 ± 19 vs. 825 ± 25 mgHA/ccm). Calvarial porosity was consistently higher in parietal than temporal bone, but unaffected by genotype. Compared with femoral cortical bone, the effect of Piezo1 deletion was more pronounced in temporal bone, suggesting heightened mechanosensitivity under localized muscular loading.
**Conclusion**: Piezo1 deficiency selectively compromises thickness and mineralization of loaded cranial bone, while non loaded cranial bone remains unaffected. These findings demonstrate that Piezo1-mediated mechanotransduction is essential for site-specific cranial bone adaptation and extend mechanosensitivity concepts from the appendicular skeleton to the skull.

**P040**

**Bone resorption during bedrest occurs predominantly in anatomical regions previously optimized to mechanical loading and is associated with the degree of structural adaptation**

*Gabi Armbrecht[1], Roswitha Dietzel[1], Kay Raum[2]*
*[1]Charité-Universitätsmedizin Berlin, Department of Radiology, Berlin, Germany*
*[2]Charité-Universitätsmedizin Berlin, Center for Biomedicine, Berlin, Germany*

**Abstract Text**

**Introduction:** We investigated whether a cycling exercise coupled with artificial gravity via a short-arm human centrifuge helps to prevent/reduce the deleterious effects induced by 60 days of bedrest. The effects of two different training schemes in comparison to no training on the intracortical pore morphology was analyzed by means of HRpQCT.

**Materials & Methods:** The BRACE study was conducted at the MEDES-IMPS in Toulouse, France (IRB approval no. 2022-A02074-39), written informed consent was obtained from all participants. Details of the study have been described elsewhere. Briefly, 24 healthy young male volunteers underwent 60 days of bed rest and a 365-days recovery period. 3 Groups (G1: no training; G2: cycling exercise, G3: cycling and artificial gravity on a short-arm centrifuge) were stratified. HRpQCT readings were performed at tibia midshaft at Baseline (BDC), after the Bed Rest Phase (HDT60) and one year after bedrest (R+365). All measured parameters were normalized to the values measured at BDC. Two-way ANOVA and stewise regression were used to analyze the relative changes with respect to training and measurement time point and associations of bone loss at HDT60 with anthropometric and macrostructural information, respectively.

**Results:** Intracortical resorption was observed after bedrest. Increased porosity (HDT60: 1.6-fold, p=0.0003) was most pronounced in the anterior crest region (F=12.6), followed by the posterior (F=9.4) and medial regions (F=3.7). Cycling resulted in increases of the moment of inertia (HDT60: +2%, p=0.007), and reduced BMD peak values (-1.1%, p=0.01), while the trend was reversed in groups G1 and G3. Observed alterations were significant at HDT60 and were almost completely recovered at R+365, but independent of the exercise group. However, age (p=0.02), moment of inertia (p=0.02) and the Feret diameter ratio (p = 0.04) were associated with bone resorption at HDT 60 (adj. $R^2$ = 0.48).

**Discussion:** Disuse induced intracortical resorption occurs predominantly in previously reinforced tissue regions.

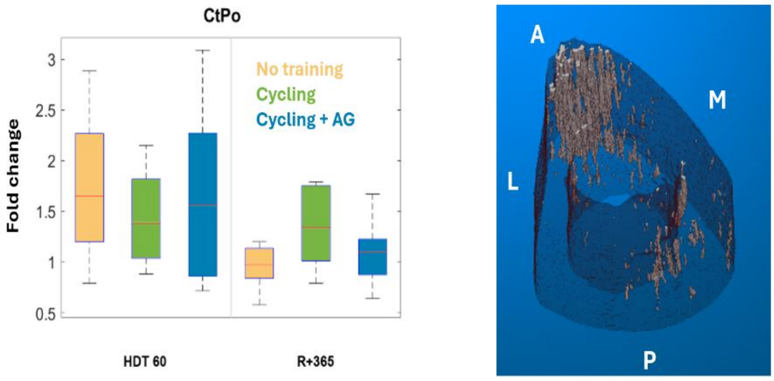

Fig. 1 Relative changes of the intracortical porosity of the full tibia midshaft cross-section after 60 days of bedrest (HDT60) and 365 days after bedrest (R+365). Intracortical resorption at HDT60 occurs predominantly at the anterior crest (right).

**P041**

### Simplified X-ray Based Parametric Foot Modeling for subject-specific Finite Element Analysis

*Olamide Hassan[1], Siwoo Jung[1], Taeyong Lee[1]*
*[1]Ewha Womans University, Division of Mechanical and Biomedical Engineering, Seoul, Korea- South*

**Abstract Text**

**Background:** Understanding foot biomechanics is essential for evaluating plantar load distribution, particularly in relation to structural variations such as flat foot and high arch. Finite element analysis (FEA) is widely applied; however, conventional modeling relies on computed tomography (CT), which is costly, exposes subjects to radiation, and requires high computational resources.

**Methods:** This study developed a simplified, parametric 3D foot bone modeling pipeline using only weight-bearing X-ray images obtained in four standard views: lateral, anteroposterior, dorsoplantar, and hindfoot alignment. Bone lengths, angles, and offsets extracted from images were used to generate geometric primitives and then assembled into a personalized bone structure. The reconstructed bones were embedded into the subject's 3D-scanned foot surface and evaluated using FEA under static standing conditions.

**Results:** Plantar pressure predictions from the simplified model were compared with experimental measurements and a CT-based model. The simplified model demonstrated less than 20% deviation in plantar pressure while reducing preprocessing time by 57.1% and computation time by 90%. Additional evaluations in four subjects with varying foot morphologies, including pes planus and pes cavus, showed consistent agreement with experimental data, with errors remaining less than 20%. Beyond accuracy, the pipeline demonstrated high repeatability and required minimal manual intervention once parameters were extracted. The use of standardized radiographic views enabled consistent reconstruction across subjects, suggesting that the approach may be automated and scaled for larger datasets. This robustness indicates suitability for longitudinal monitoring, population studies, and integration into clinical workflows where rapid turnaround and reproducibility are essential.

**Conclusion:** The findings support X-ray based parametric modeling as a lightweight, scalable, and low-cost alternative to CT-dependent FEA pipelines. This approach enables rapid subject-specific analysis, facilitates customized orthotic and insole design, and offers strong potential for implementation in large-scale biomechanical screening and low-resource clinical environments.

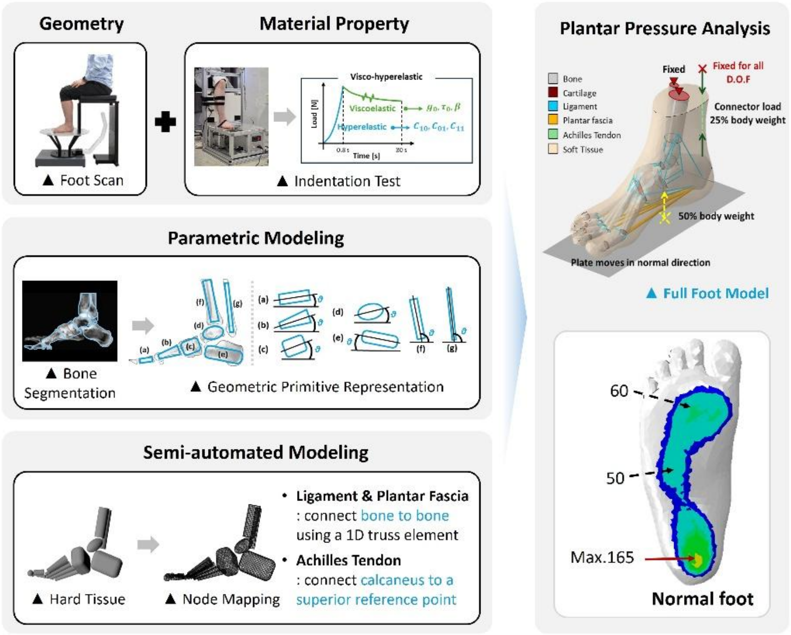

**P042**

**Investigating the influence of microcalli on trabecular bone mechanics through finite element analysis**

*<u>Xenia Junimann</u>[1,2], Johannes Krug[1,2], Sara Checa[3], Michael Hahn[1], Annika vom Scheidt[4], Björn Busse[1,2]*
*[1]University Medical Center Hamburg-Eppendorf, Department of Osteology and Biomechanics, Hamburg, Germany*
*[2]University Medical Center Hamburg-Eppendorf,*
*Interdisciplinary Competence Center for Interface Research, Hamburg, Germany*
*[3]Hamburg University of Technology, Institute of Biomechanics, Hamburg, Germany*
*[4]Medical University of Graz,*
*Division of Macroscopic and Clinical Anatomy- Gottfried Schatz Research Center, Graz, Austria*

**Abstract Text**

**Background:** Among osteoporotic fractures, non-traumatic microfractures caused by material fatigue a common occurence. Microcalli form as part of the fracture healing process in the trabecular bone where the fracture gap is bridged by a porous irregular structure of woven bone. However, the effect of microcalli on the mechanical integrity of the trabecular network remains unclear, especially with persisting microcalli.
**Purpose:** This study aims to investigate the mechanical influence of microcalli on trabecular networks with and without microcalli using finite element analysis.
**Methods:** Human lumbar vertebrae were assessed from autopsies of 82 ± 6.5 year old women diagnosed with osteoporosis, of which six were undergoing bisphosphonate treatment. Micro-computed tomography scans of the trabecular bone were acquired. To isolate the effects of microcalli on the mechanics of the trabecular network, subvolume surface meshes containing microcalli were manually modified to smoothed, microcallus-free networks and fractured model networks and simulated with a compression of 1%.
**Results:** The finite element analysis of this study revealed lower mean and maximum stresses in the original microcalli networks compared to their smoothed version in the local microcallus regions (-24.64%; p<0.0001), while slightly increasing stresses around the calli (+3.4%; p=0.0031). The global stiffness was significantly higher in the networks with original microcallus structures compared to their smoothed equivalent network although the effect size was low (+0.63%; p=0.0012). Additionally, microcalli affected trabecular bone mechanics of untreated and treated osteoporosis in a similar way (p=0.855), although more microcalli were found in the bisphosphonate treated samples.
**Conclusion:** The findings suggest that the effect of microcallus formations are mostly limited to a local stress-reduction in trabecular networks. This study underlines the mechanical role of microcalli, thereby adding crucial understanding of vertebral bone strength in osteoporosis and fracture risk assessment.

**P043**

**Deep Learning Integration of Raman Spectroscopy and Clinical Data for Predicting Tensile Mechanical Properties of Human Cortical Bone**

*Muhammet Varli[1], Mustafa Unal[2,3], Sasidhar Uppuganti[4], Ahmet Kayabasi[5], Jeffry S. Nyman[4]*
*[1]Karamanoglu Mehmetbey University, Department of Mechatronics Engineering, Karaman, Turkey*
*[2]Health Institutes of Turkiye-TUSEB, Aziz Sancar Research Center, Ankara, Turkey*
*[3]Harvard Medical School, Department of Orthopedic Surgery, Boston MA, United States*
*[4]Vanderbilt University Medical Center, Department of Orthopedic Surgery, Nashville TN, United States*
*[5]Karamanoglu Mehmetbey University, Department of Electrical and Electronics Engineering, Karaman, Turkey*

**Abstract Text**

**Introduction:** Bone strength and fracture resistance depend on tensile mechanical properties that are not fully captured by conventional clinical assessments, particularly in populations that include healthy individuals and those with metabolic disorders such as type 2 diabetes mellitus (T2DM). Raman spectroscopy (RS) provides detailed information on bone tissue composition, yet the high dimensionality of full spectrum RS data limits traditional analytical approaches. Recent advances in artificial intelligence, particularly deep learning (DL), enable automated extraction of nonlinear features from complex spectral and clinical datasets. Integrating RS with DL models offers a framework for predicting bone mechanical properties.

**Purpose:** This study evaluated whether Raman spectrum integrated with DL methods can predict tensile biomechanical properties of human cortical bone in individuals with and without T2DM.

**Methods:** Raman spectra were collected from cortical bone samples obtained from fresh frozen cadaver femurs of 120 donors, including 60 donors with T2DM and 60 non diabetic controls. The cohort included equal numbers of men and women aged 50 to 97 years. All samples underwent tensile mechanical testing, and yield stress, ultimate stress, toughness, post-yield toughness, and post-yield strain were calculated. A 1D CNN model was trained using the full Raman spectrum. A combined DL architecture integrated a CNN trained on Raman data with a multilayer perceptron trained on clinical variables, including age, BMI, sex, and chronic kidney disease (CKD) status.

**Results:** The combined DL model achieved the highest predictive performance, with $R^2$ values between 0.83 and 0.89 across tensile biomechanical properties. DL models using only Raman spectra also demonstrated strong predictive accuracy.

**Conclusion:** DL based analysis of full spectrum RS can accurately predict tensile biomechanical properties of human cortical bone. Incorporating clinical data improves performance. This study represents the first application of DL to predict tensile biomechanical properties and highlights the potential of DL enabled spectroscopic approaches for bone quality assessment.

**P045**

**Feasibility of estimation of femoral bone strength in fibrous dysplasia of bone with the MEKANOS tool.**

*Helene Follet[1], Cyrille Confavreux[2], Deborah Gensburger[3], Nadege Trehet-Mandel[3], Thibault Gandet[4], Marine Grandin[5], Aurélie Levillain[5], Marc Gardegaront[6], Emile Saillard[7], Thomas Grenier[7], David Mitton[5], Roland Chapurlat[8]*

*[1]INSERM / Université de Lyon, 1033 - LYOS, Lyon, France*
*[2]Hospices civils Lyon, Rhumathology department, Lyon, France*
*[3]Hospices civils de Lyon, Rhumathology department, Lyon, France*
*[4]Univ Lyon- Université Claude Bernard Lyon 1- INSERM, Lyos, Lyon, France*
*[5]Univ. gustave Eiffel, Lbmc, Bron, France*
*[6]Univ Lyon- Université Claude Bernard Lyon 1- INSERM- LYOS UMR 1033, Lbmc, Bron, France*
*[7]INSA, Creatis, Lyon, France*
*[8]Hospices Civils de Lyon, Rhumatology, Lyon, France*

**Abstract Text**

Fibrous dysplasia of bone (FD) is a rare, congenital bone disease due to a post-zygotic mutation of GNAS (an imprinted gene) causing bone pain, deformities and fractures. Mechanically speaking, FD lesions, which are often lytic and heterogeneous, can be compared to those of bone metastases. The assessment of mechanical strength and fracture risk in FD lesions, however, remains a significant unresolved issue for clinicians.

We have developed MEKANOS, a digital simulation tool for calculating the mechanical strength of tumor bone based on routine scans using a dedicated protocol. Briefly, we wanted to determine whether this tool can be used to quantify bone strength in FD, with routine CT scanning? To this end, we used scanned images of patients with FD (15 patients) obtained by the Fibrous Dysplasia National Reference Center. For each patient images, mid-length femur lengthening was done, calibration of Hounsfield Units (HU) to density was performed using (i) a patient-specific method and (ii) an asynchronous method. Femurs were automatically segmented, meshed using tetrahedral elements, and assigned linear elastic material properties using a model implemented in Lyon Femurs were loaded in axial compression, and their failure load was computed using a failure criterion based on a volume of contiguous failed elements. protocol.

We compared the fracture strength of femurs obtained numerically using the two calibration methods (n=28 from 14 patients). Both methods showed a strong correlation ($R^2$=0.92), but a relative bias of 12% (SD=5.56%, and IC95% [1.2 to 23%]). Between pathological bone and healthy bone, for 6 patients, both models were able to show a significant difference of bone strength between them (*resp* p=0.0278 for patient-specific method and p=0.0377 for asynchronous method).

These preliminary results are very encouraging for the continuation of the study and to elaborate a fracture risk criterion for this type of rare disease

**ND-P005**

### Does a novel compensatory strain mechanic at fibril-mineral nanoscale interface sustain macro-tissue performance at high strain in bone?

*Richie Stavri[1], Crispin Wiles[2], Ulrich Hansen[3], Richard Abel[1]*
*[1]Imperial College London, Musculoskeletal Laboratory- Department of Surgery & Cancer, London, United Kingdom*
*[2]University of Warwick, Warwick Medical School, Warwick, United Kingdom*
*[3]Imperial College London, Department of Mechanical Engineeering, London, United Kingdom*

**Abstract Text**

Despite extensive characterisation of various damage phenomena in human bone at tissue- and micro-scales, less is understood about the nanoscale, particularly regarding mechanisms of the collagen-mineral interface and their contribution to fracture risk. As such a novel synchrotron platform was developed combining small- and wide-angle X-ray scattering (SAXS/WAXS) with digital image correlation (DIC) to simultaneously capture nanoscale deformation and local tissue strain of 154 cadaveric femoral cortical sections loaded in tension to failure at I22 Beamline, Diamond Light Source. The multiscale-multimode design aimed to (1) elucidate better the load distribution during inelastic deformation (ID) of cortex on the nanoscale; (2) collocate this with tissue-level damage phenomena; and (3) evaluate age-related disruption to these pathways. In ageing bone, the research discovered a previously unrecognised compensatory strain mechanism, observed as a decoupling of the nanoscale strain responses in the high strain regime, with collagen fibrils maintaining or increasing strain contribution, $r$ = 0.23, 0.29 (Middle-Ageing, Ageing), $p < 0.005$, via least squares regression, to offset a declining mineral performance, $r$ = 0.34, 0.20, $p < 0.01$. The decoupling was found to occur simultaneously to the onset of tissue-level ID as resolved by DIC. Ageing as a variable was found to have no overall effect on peak strain values, though data-binning showed reductions in tissue-level strain in the ageing cohort were coincident to declining mineral strain whereas fibril strain performance was maintained, in corroboration with the novel mechanistic finding. Age was found to be associated generally with an elevated variability of measured mechanical properties, and that intra-donor sample variability was found in cases to be just as large as variability of samples from different donors. The work challenges conventional bone quality assessment paradigms by demonstrating that strength emerges from dynamic interactions between organic and inorganic

**Poster Sessions: Bone development/growth and fracture repair**

**P046**

**Impaired fracture healing upon neutrophil-specific adrenoreceptor beta 2 (Adrb2) knockout in non-osteoporotic and osteoporotic mice**

*Sandra Dieterich*[1]*, Nico Gläser*[1]*, Christoph Kölbl*[1]*, Dorothea Gebauer*[1]*, Jana Bleher*[1]*, Oliver Küppers*[1]*, Verena Fischer*[1]*, Anita Ignatius*[1]*, Melanie Haffner-Luntzer*[1]
[1]*University Medical Center Ulm, Institute of Orthopaedic Research and Biomechanics, Ulm, Germany*

**Abstract Text**

**Background/Introduction:** Osteoporotic mice display heightened systemic levels of proinflammatory cytokines and more neutrophils in the early fracture hematoma, which was associated with delayed bone healing. The underlying mechanism remains unclear. As it was shown in male mice that short-term injection of the beta-blocker Propranolol led to reduced neutrophil recruitment to the hematoma, we hypothesized that $\beta_2$-adrenoreceptor (Adrb2) signaling on neutrophils might be associated with delayed fracture healing in osteoporotic mice.
**Purpose:** The aim of this study was to analyze the role of Adrb2 signaling specifically in neutrophils on fracture healing.
**Methods:** A conditional KO of the *Adrb2* on $Ly6G^+$ neutrophils was generated using Ly6G-Cre Adrb2-flox mice. 12-week-old female mice underwent ovariectomy (OVX) to induce postmenopausal osteoporosis or sham surgery followed by a standardized femur osteotomy stabilized with an external fixator after 4 weeks (animal license: no.1612). Mice were sacrificed at 3- or 21-days post-fracture and analyzed using µCT analysis and histology. Neutrophils were further characterized via RNA-sequencing.
**Results:** Fracture healing was significantly impaired in OVX mice compared to sham mice regardless of genotype as indicated by reduced bone formation ($p<0.05$) 21 days post-injury. Furthermore, Cre+ sham and OVX mice healed significantly worse compared to the respective Cre- groups ($p<0.0001$)(Fig.1,a). The molecular mechanism behind this finding might be that Cre+ mice displayed drastically reduced neutrophil and mast cell numbers in the fracture callus (Fig.1,b,c). RNA-sequencing of isolated neutrophils further demonstrated a disturbed neutrophil activation and an impaired neutrophil/mast cell crosstalk in Cre+ neutrophils which could explain the worse fracture healing outcome.
**Conclusion:** Both non-osteoporotic and osteoporotic Ly6G-Adrb2-KO mice display impaired fracture healing compared to Cre- mice. This could be due to an excessive restriction of neutrophil recruitment and a disturbed neutrophil/mast cell interaction upon Ly6G-Adrb2-KO, indicating how delicate the balance of inflammation must be after fracture. Further molecular mechanisms need to be investigated in more detail.

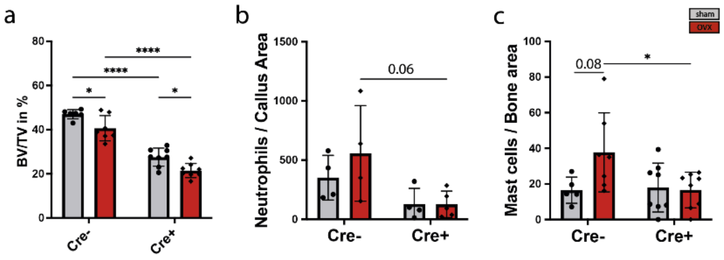

**Fig.1**: **a**: Bone volume/tissue volume (BV/TV) of the fracture callus 21 days post-fracture. **b**: Neutrophil (Ly6G) staining in the early fracture hematoma (3 days post-fracture). **c**: Mast cell (Mcpt5) staining in the fracture callus 21 days post-fracture. Statistical significance was determined by Two-way ANOVA. *P < 0.05, **P < 0.01, ***P < 0.001, ****P < 0.0001. Bars: light grey = sham; red = OVX. (*N*=4-6)

**P047**

**Early life stress alters the early cytokine response in a sex-specific manner, but has no long-term effect on fracture healing outcome in mice**

*Dorothea Gebauer*[1], *Tamara Schimmele*[2], *Dominik Langgartner*[2], *Jana Bleher*[1], *Anita Ignatius*[1], *Stefan O. Reber*[2], *Melanie Haffner-Luntzer*[1]

[1]*University Hospital Ulm, Institute of Orthopaedic Research and Biomechanics, Ulm, Germany*

[2]*University Hospital Ulm, Laboratory for Molecular Psychosomatics, Ulm, Germany*

**Abstract Text**

**Background:** Early life stress affects approximately 40% of the world population and is an acknowledged risk factor for the development of several somatic and affective disorders. This study aimed to investigate whether early life stress influences bone fracture healing, and whether potential effects are sex specific.

**Methods:** All animal experiments were approved by the local animal welfare committee and were performed in compliance with international regulations for laboratory animal welfare and handling. Early life stress was induced in C57BL/6N mice using a maternal separation (MS) paradigm (separation of litters from the dam for 3 hours per day during the first 14 postnatal days). At 12 weeks of age, a standardized right femur osteotomy was introduced and stabilized by an external fixator. Mice were euthanized 3 hours, 10 days, or 21 days post-fracture. Fracture healing was assessed via cytokine assays, histology, biomechanical testing, and µCT measurements.

Statistics: two-tailed Student's t tests with Welch's correction when appropriate, p<0.05, n=4-11/group.

**Results:** Male mice exposed to MS showed a reduced inflammatory response at the fracture site 3 hours post-fracture, whereas plasma cytokine concentrations were reduced in both sexes. These effects appeared to be transient, considering that as neither the composition of the fracture callus nor functional parameters differed between stressed and non-stressed mice at days 10 and 21 in either sex.

**Figure 1: Effects of MS on fracture healing at 10 and 21 days post-fracture in male and female mice.**
Histological analysis of fracture callus composition in (A) male and (C) female mice 10 days after femur osteotomy. Biomechanical testing and µCT analysis of fractured femura in (B) male and (D) female mice 21 days after femur osteotomy.
n=4-11, no significant differences.
MS: maternal separation; BMD: bone

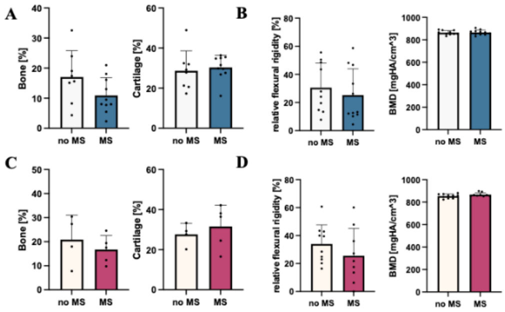

**Conclusion:** Our results indicate that MS modulates the early inflammatory phase of fracture healing in a sex-specific manner; however, these alterations did not translate into long-term effects on callus tissue formation and functional outcome. The data support the possibility of compensatory mechanisms or a threshold model, but further studies are required to elucidate the underlying processes.

**P048**

**The DAMPs-PRR axis promotes osteoclastogenesis and is upregulated in osteoclasts and in bone of mice with metabolic dysfunction-associated steatotic liver disease (MASLD).**

*Mafalda De Montenegro Cortez*[1], *Saimadhuvanthy Srinivas*[1], *Nadia Schlebe*[2,3,4], *Pauline Porschitz*[1], *Juliane Salbach-Hirsch*[2], *Hani Harb*[5], *Nikolaos Perakakis*[2,3,4], *Martina Rauner*[2], *Darja Andreev*[1]
[1]*Center for Regenerative Therapies Dresden, Technische Universität Dresden, Dresden, Germany*
[2]*Technische Universität Dresden,*
*Department of Internal Medicine III- University Hospital Carl Gustav Carus, Dresden, Germany*
[3]*University Hospital and Faculty of Medicine, Helmholtz Center Munich, Munich, Germany*
[4]*German Center for Diabetes Research DZD e.V., Paul Langerhans Institute Dresden PLID, Dresden, Germany*
[5]*Technische Universität Dresden, Institute for Medical Microbiology and Virology- Medical Faculty, Dresden, Germany*

**Abstract Text**

**Background/Introduction:** Metabolic dysfunction-associated steatotic liver disease (MASLD) is associated with bone loss, increased fracture risk, and reduced bone regenerative capacity. MASLD features elevated cell death and release of Damage-associated Molecular Patterns (DAMPs); however, whether these DAMPs activate Pattern Recognition Receptors (PRRs) on osteoclasts, thereby promoting osteoclast activity and bone loss, remains unknown.

**Purpose:** We aim to determine how cell death and DAMP release influence osteoclastogenesis and

PRR expression in vitro and during bone injury in vivo. We also examine how MASLD affects osteoclasts and the DAMPs–PRR axis, and whether dietary intervention can mitigate these effects.

**Methods:** We examined the effects of DAMPs on osteoclastogenesis by coculturing bone marrow–derived osteoclasts with necrotic osteoblasts and profiling the released DAMPs using a Proteome Profiler Array. A calvarial defect model performed on 14-week-old male mice (TVV 18/2022) was used to assess the DAMP–PRR pathway 14 days following bone injury. To investigate this pathway in MASLD, 6-week-old male mice were fed a Western diet for 10–36 weeks (TVV 5/2024) and subsequently underwent either an 8-week reversal diet or alternate-day fasting.

**Results:** Coculture of osteoclast precursors on day 2 of differentiation with necrotic osteoblasts enhanced the formation of TRAP⁺ osteoclasts and increased PRR expression (*Mincle* and *Tlr2*), indicating that the DAMP–PRR axis promotes osteoclastogenesis. In the calvarial defect model, the osteoclast genes *Acp5* and *Ctsk* were upregulated in the hole area, consistent with increased expression of the PRRs Dectin-1 and Tlr4. Notably, osteoclast differentiation was elevated in MASLD mice and was accompanied by higher PRR expression (*Dectin-1*, *Mincle* and *Tlr4*) in both osteoclasts and bone tissue. These increases in PRR expression were mitigated by dietary interventions.

**Conclusions:** During bone injury and metabolic disease, osteoclastogenesis was increased, accompanied by enhanced DAMP-PRR pathway, underlining the potential role of this axis in pathological bone loss.

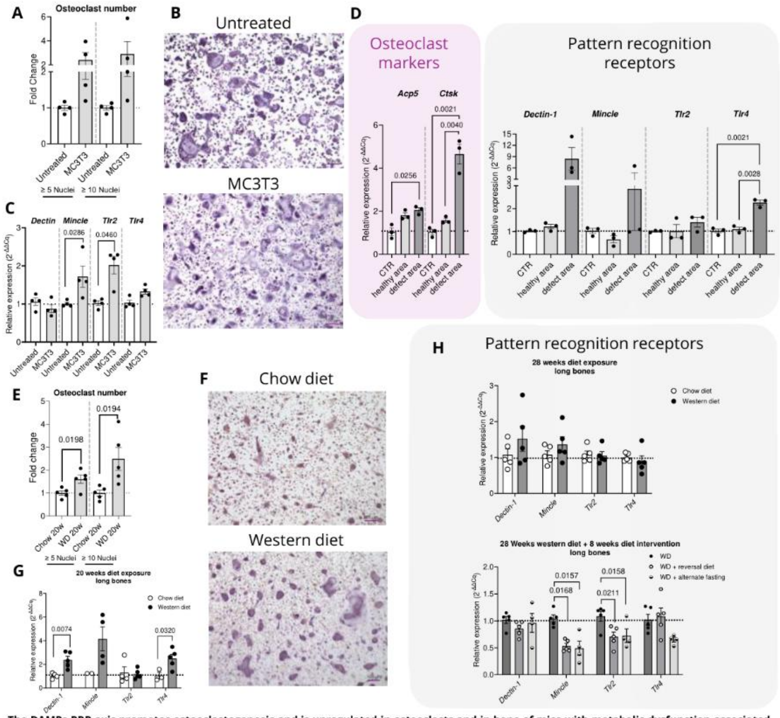

**The DAMPs-PRR axis promotes osteoclastogenesis and is upregulated in osteoclasts and in bone of mice with metabolic dysfunction-associated steatotic liver disease (MASLD). A)** Number of TRAP+ bone marrow (BM)-derived osteoclasts cocultured with necrotic MC3T3 cells on day 2 of differentiation, **B)** respective representative pictures (10x amplification) and **C)** expression of pattern recognition receptors (PRRs). **D)** Relative expression of osteoclast genes and PRRs in calvarial bone from *in vivo* calvaria bone injury model. **E)** Number of TRAP+ BM- derived osteoclasts from mice fed with western diet (WD) for 20 weeks versus control diet and **F)** respective representative pictures (10x amplification). **G)** Expression of PRRs in long bone of WD fed mice for 20, **H)** 28 weeks and 28 + 8 weeks diet intervention.

**P049**

**Short-term cold exposure promotes fracture repair**

_Jan Sevecke_[1], *Ruben Augustin*[1], *Jörg Heeren*[2], *Anke Baranowsky*[1], *Johannes Keller*[1]
[1]*University Medical Center Hamburg-Eppendorf,*
*Department of Trauma Surgery and Orthopedics- Experimental Trauma Surgery, Hamburg, Germany*
[2]*University Medical Center Hamburg-Eppendorf,*
*Department of Biochemistry and Molecular Cell biology, Hamburg, Germany*

**Abstract Text**

**Background:** Impaired fracture healing represents an ongoing clinical challenge, as therapeutic options are limited. Recently, the sympathetic nervous system has gained increased attention as a targetable stimulus due to its beneficial impact on fracture repair. Sympathetic nerves promote vascularization of newly formed callus tissue via beta-2-adrenergic (Adrb2) signaling (Jahn et al., 2024). This is also evident clinically in patients with fractures and concurrent traumatic brain injury, which causes an increase in sympathetic outflow and accelerates fracture healing. However, the validity of this principle in relation to other stimuli of the sympathetic nervous system remains unclear.

**Objectives:** To investigate the impact of cold exposure, a potent stimulus for sympathetic outflow, on fracture repair.

**Methods:** WT and Adrb2-deficient mice were subjected to a standardized femoral osteotomy stabilized by an external fixator. Subsequently, mice were maintained either at room temperature (control), at 6°C for seven days following osteotomy (short-term exposure), or continuously at 6°C (long-term exposure). On days 7, 14 and 21 after osteotomy, the mice were assessed for the distinct stages of bone regeneration using radiological and histological approaches. The biomechanical stability of the healing bones was tested 28 days after osteotomy.

**Results:** Compared to mice maintained at room temperature, WT mice with short-term cold exposure showed accelerated fracture repair, indicated by increased callus volume and mineralization ($p<0.05$). This was associated with increased formation of osteogenic type-H vessels in the callus and enhanced biomechanical stability ($p<0.05$). In contrast, no improvement in callus composition was observed in WT mice subjected to long-term cold exposure. Finally, Adrb2-deficient mice failed to display improved fracture repair after short-term exposure to cold temperature.

**Conclusions:** These findings demonstrate that short-term cold exposure - a strong and physiological stimulus for sympathetic outflow - enhances fracture repair in an Adrb2-dependent manner. Further studies are now warranted to explore means of clinical applicability.

**P050**

**Methotrexate-induced osteopathy: findings from affected patients versus a methotrexate-treated control group**

Klara Franziska Becker[1], Martina Blaschke[2,3], Gabriele Lehmann[2], Annette Lamersdorf[2],
Volker Thiem[4], Ludovic Humbert[5], Heide Siggelkow[2,6]
[1]1Research and Development- Department of Trauma- Orthopedics and Reconstructive Surgery, University Medical Center Göttingen, Göttingen, Germany
[2]MVZ endokrinologikum Göttingen,
Center for Endocrinology- Osteology- Rheumatology- Nuklear Medicine and Human Genetics, Goettingen, Germany
[3]Clinic of Gastroenterology- Gastrointestinal Oncology and Endocrinology,
University Medical Center Göttingen, Goettingen, Germany
[4]MVZ endokrinologikum Göttingen,
Center for Endocrinology- Osteology- Rheumatology- Nuklear Medicine and Human Genetics,
heide.siggelkow@med.uni-goettingen.de, Germany
[5]3D-Shaper Medical, 3D-Shaper Medical, Barcelona, Spain
[6]Research and Development- Department of Trauma- Orthopedics and Reconstructive Surgery, University Medical Center Göttingen, Goettingen, Germany

**Abstract Text**

**Background:** Methotrexate (MTX) is widely used for the treatment of rheumatic diseases. MTX can also have adverse effects on bone metabolism, including the development of methotrexate-induced osteopathy. We aimed to systematically identify risk factors for the development of methotrexate-induced osteopathy.

**Methods:** We retrospectively investigated data from 33 patients with MTX-osteopathy (MTX-O) in comparison to an age- and sex-matched control group of patients (ratio 3:1, n = 99) receiving MTX treatment without clinical signs of osteopathy (MTX-C). Assessments included bone mineral density measurements (DXA), laboratory parameters, fracture risk scores (FRAX, DVO risk calculator), as well as 3D-SHAPER® analyses of proximal femur geometry and bone quality.

**Results:** Of 132 participating patients, 94% were female. Patients with MTX-O received MTX more often subcutaneously compared to oral or sequential administration in the MTX-C ($p<0.001$). In the MTX-O 46% of patients had bilateral fractures, and 67% had more than one fracture. Analyses revealed elevated GGT and GOT levels ($p=0.004$; $p=0.002$) as well as higher 25-OH vitamin D and hemoglobin levels ($p<0.001$; $p=0.036$) in the MTX-O, whereas ESR and leukocyte counts were higher in the MTX-C group ($p=0.001$; $p=0.047$). Osteoporosis was more frequent in the MTX-O ($p<0.001$), accompanied by lower bone mineral density values in this group. After adjusting for osteoporosis, significant group differences in 3D-Shaper® analysis remained for the slice area of the right femoral neck and the surface bone mineral density of the intertrochanteric lateral region ($p=0.022$; $p=0.044$). FRAX score and Hip & Vertebral (HiVe) fracture risk score algorithm indicated a higher risk of osteoporotic fractures in the MTX-O.

**Conclusion:** Timely diagnosis of MTX-induced osteopathy is key to preserving mobility; in high-risk patients, MTX use should be carefully considered.

**Key Words:** methotrexate, osteopathy, osteoporosis, fracture, bone mineral density, 3D-Shaper®

**P051**

**Sex-dependent effects of nox4 inactivation in fracture healing model in obese mice**

*Mariana Santos*[1]*, Martina Dzubanova*[1]*, Kim Nguyen*[1]*, Michaela Ferencakova*[1]*, Adele K. Addington*[2]*, Sioban M. Craige*[2]*, Michaela Tencerova*[1]

[1]*Institute of Physiology of the Czech Academy of Sciences,*
*Laboratory of Molecular Physiology of Bone, Prague, Czech Republic*
[2]*Virginia Tech, Human Nutrition- Foods and Exercise, Blacksburg, United States*

**Abstract Text**

**Background**: Obesity increases bone fragility risk through oxidative stress. Our previous studies showed increased expression of NADPH oxidase 4 (NOX4), a major producer of reactive oxygen species, in obesity. However, its role in fracture healing in obesogenic conditions is unknown. Therefore, we investigated whether NOX4 deficiency improves fracture healing in obese mice in a sex-dependent manner.

**Methods**: Male and female WT, global NOX4 knockout (KO) and Prx1-Cre conditional NOX4 knockout (cKO) mice were fed either chow or a high-fat diet (HFD) for 8 weeks (n= 4-11/group). After 5 weeks, a monocortical tibial defect was performed. Body weight, glucose tolerance, callus microarchitecture (µCT) and gene expression (RT-qPCR) were assessed.

**Results**: KO and cKO males were protected from HFD-induced weight gain (-15.52% p<0.05; -21.09% p<0.01 vs WT-HFD), with no effect in females. Glucose metabolism was partially preserved in KO-HFD males and females (-14.60% p<0.05; -15.09% p<0.01 vs WT-HFD), with improvement in cKO-HFD restricted to males (-18.19% p<0.01 vs WT-HFD). µCT showed increased callus mineralization and trabecular thickness in KO-HFD males (112.76%; 37.44% p<0.001 vs WT-HFD), whereas cKO-HFD males showed increased trabecular number (22.41% p<0.001 vs WT-HFD). Females KO-HFD and cKO-HFD showed increased mineralization (47.60% p<0.05; 20.97% p<0.001 vs WT-HFD) despite trabecular disorganization. KO-HFD males upregulated *Alpl*, while cKO-HFD males downregulated it (23.36%; -18.41%). KO-HFD females downregulated *Bmp2* (-32.10%), *Col1a1* (-37.46%) and *Runx2* (-25.85%) and upregulated *Acp5* (55.48%); cKO-HFD females reduced *Bglap* (-32.13%) and *Ctsk* (-26.69%). KO-HFD reduced adipogenic markers in both sexes: *Adipoq* (-52.87%; -74.90%) and *Fsp27* (-11.95%; -30.48%), with females also reducing *Pparg2* (-57.04%). Only males cKO-HFD reduced *Fsp27* (-26.26%) and *Pparg2* (-35.96%) (vs WT-HFD *p<0.05*).

**Conclusion**: Global NOX4 deletion provides metabolic protection and improves bone repair in males but disrupts trabecular organization in females. Conditional deletion produces modest sex-dependent effects, highlighting the relevance of cellular context in obesity-related bone repair.

**P052**

**Control of cartilage resorption by hypoxia signaling**

*Miguel Echanove-González de Anleo[1], Tania López-Martínez[1], Henar Reina-García[1], Purificación Ripalda-Cemboráin[1], Amaia Cipitria[2], Leire Iturriaga[2], Emma Muiños-López[1], Froilán Granero-Moltó[1]*
[1]*Clínica Universidad de Navarra, Orthopedic Surgery and Traumatology, Pamplona, Spain*
[2]*Bioguipuzkoa, Bioengineering in Regeneration and Cancer, San Sebastian, Spain*

**Abstract Text**

**Background**: A critical step during fracture healing is the removal of the cartilage template, coupled with vascular invasion and new bone formation. We have demonstrated that hypoxia signaling controls endochondral ossification progression, and silencing Apex1 in limb bud mesenchyme results in delayed fracture healing and nonunion. It is unknown the role of hypoxia signaling in non-mesenchymal tissue and how impacts endochondral ossification progression.

**Purpose**: To determine the role of hypoxia signaling in hematopoietic cells during fracture healing.

**Methods**: All animal procedures were approved by our institutional committee for animal use in research. *Apex1* was silenced in hematopoietic progenitors with the inducible Mx1Cre deleter by intraperitoneal injection of polyI:C one-week before injury. A closed fracture was performed in the tibia of Mx1Cre;Apex1$^{C/C}$ (Apex1$^{KO}$) and WT mice littermates. The repair process was evaluated at the end the reparative phase (10- 14- and 21-dpf) by histology, immunohistochemistry and microcomputed tomography (µCT).

**Results**: Silencing Apex1 in hematopoietic progenitors resulted in significant increase in the content of mineralized tissue at 14-dpf (WT, 15.00±2.92 mm$^3$; Apex1$^{KO}$, 25.90±5.67 mm$^3$; p=0.0027) without significant differences in total callus volume (WT, 37.46±1.51 mm$^3$; Apex1$^{KO}$, 46.77±5.61 mm$^3$; p=0.1110) or soft tissue content (WT, 13.54±3.07 mm$^3$; Apex1$^{KO}$, 11.50±2.41 mm$^3$; p=0.1215) as determined by quantitative µCT. By 21-dpf, morphometric differences disappear between genotypes (Figure 1). Histological analysis showed no major differences in the content of bone or cartilage tissues at 14-dpf. TRAP staining showed a significant reduction in the number of TRAP$^+$ cells in the chondro-osseous junction of Apex1$^{KO}$ (WT, 0.516±0.453%; Apex1$^{KO}$, 0.08±0.08%; p=0.0045) resulting in the accumulation of hypertrophic cartilage. Interestingly, histological Von Kossa staining of non-decalcified calluses showed an increase on the mineralized cartilage in Apex1$^{KO}$ animals at 14-dpf.

In conclusion, silencing Apex1 in hematopoietic progenitors impairs cartilage resorption that results in the transient calcified cartilage accrual.

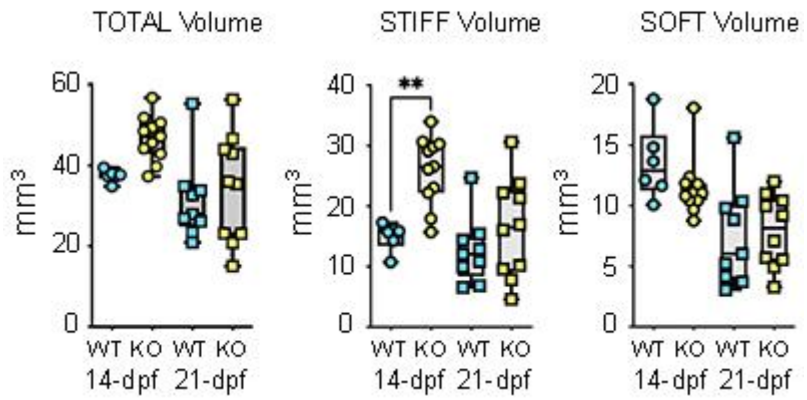

**P053**

**Characteristics of patients with Osteogenesis Imperfecta: first results of the Core registry of the European Registries for Rare Endocrine and Bone Conditions**

*Femke Van Haalen[1], Mariya Cherenko[1], Johan Blokzijl[2], Rachel Crowley[3], Corinna Grasemann[4], Nataliia Grygorieva[5], Nikola Ilic[6], Guus Janus[2], Daryna Kurylo[5], Marina Mordenti[7], Rodrigo Montero-Lopez[8], Adalbert Raimann[9], Ondrej Soucek[10], André Travessa[11], Evert van Velsen[12], Charlotte Verroken[13], Maria Yavropoulou[14], Wolfgang Högler[8], Natasha Appelman-Dijkstra[1]*

*[1]Leiden University Medical Centre, Department of Medicine- Division of Endocrinology, Leiden, Netherlands*

*[2]Isala, Orthopedics, Zwolle, Netherlands*

*[3]St. Vincent's University Hospital and University College Dublin, Endocrinology, Dublin, Ireland*

*[4]University of Mainz, Pediatrics, Mainz, Germany*

*[5]D.F. Chebotarev institute of gerontology of the NAMS of Ukraine, Gerontology, Kyiv, Ukraine*

*[6]Mother and Child Health Care Institute of Serbia "Dr. Vukan Cupic", Endocrinology, Belgrade, Serbia*

*[7]IRCCS Istituto Ortopedico Rizzoli, Department of Rare Skeletal Disorders, Bologna, Italy*

*[8]Johannes Kepler University Linz, Centre of Growth and Osteology- Department of Paediatrics and Adolescent Medicine, Linz, Austria*

*[9]Medical University Vienna, Department of Pediatrics & Adolescent Medicine, Vienna, Austria*

*[10]Charles University and Motol University Hospital, Second Faculty of Medicine, Prague, Czech Republic*

*[11]ULS Santa Maria and University of Lisbon, Department of Medical Genetics and Faculty of Medicine, Lisbon, Portugal*

*[12]Erasmus Medical Center, Department of Internal Medicine, Rotterdam, Netherlands*

*[13]Ghent University Hospital, Department of Endocrinology- Unit for Osteoporosis and Metabolic Bone Diseases, Ghent, Belgium*

*[14]General Hospital of Athens "LAIKO", First Department of Propaedeutic and Internal Medicine- Centre of Expertise for Rare Endocrine Diseases-C.E.R.E.D, Athens, Greece*

**Abstract Text**

**Background:** Osteogenesis Imperfecta (OI,ORPHA666) subsumes a group of rare, genetic bone disorders caused by variants in *COL1A1*,*COL1A2* or collagen-associated genes. Typical features involve impaired bone matrix composition and architecture leading to bone fragility, deformities, functional limitations, and pain. To date, information on treatment data is scarce.This study aimed to explore data from the European Registries for Rare Endocrine and Bone conditions (EuRREB) OI module.

**Results:** Between 2020 and 2025 650 individuals diagnosed with OI were recorded; 241 children(37%) and 409 adults(63%) originating from 16 countries and evaluated in 40 expert centers. Between January 2022-November 2025 more detailed information was collected in the Core Registry from 273 patients (201 adults and 72 children,147 females) from 11 countries(13 centres). Of these, 127 patients had OI type 1 (47%), 33 with type 3 (12%), 31 with type 4(11%), 5 with type 5(2%) and in 77 cases(28%) the type was not specified. The median age at diagnosis was 6.9(IQR 0.2-27.9) years. Median diagnostic delay was 0.6 (IQR 0-11.6) years. Molecular genetic testing was performed in 56 children(78%) and 137 of the adults(68%). The module was completed for 71(26%) patients (55 adults and 16 children). Out of these, fractures were reported in 9/16 children(56%) and 17/55 adults(31%).Nitrogen containing bisphosphonates were used in 9/16 children (56%) and 30/55 (55%) adults. 9/55(16%) adults reported other medication like etidronate, teriparatide, romosozumab at some timepoint. 5/16(30%) children and 23/55(42%) adults did not use any kind of bone active drugs. DEXA was performed in 50/55(91%) adults and in 8/16(50%) of the children. Surgeries were performed for fractures and deformities in 8/16(50%) children and 14/55(42%) adults.

**Conclusion:** These results contribute to a better understanding of OI via collection of real world data and can support significant improvements in diagnosing, screening and therapeutic decision-making in the outpatient clinic.

**P056**

**High fat diet / low-dose streptozotocin-induced type-2 diabetes impairs trabecular bone structure and delays fracture healing in C57BL/6J mice**

*Maria Schröder[1], Lena Gens[2], Daniel Arens[2], Nico Valerio Giger[3], Alisa Hangartner[4], Dominic Gehweiler[4], Stephan Zeiter[2], Martin Stoddart[3], Esther Wehrle[3]*
*[1]AO Research Institute Davos, Regenerative Orthopedics, Davos, Switzerland*
*[2]AO Research Institute Davos, Preclinical Services, Davos Platz, Switzerland*
*[3]AO Research Institute Davos, Regenerative Orthopedics, Davos Platz, Switzerland*
*[4]AO Research Institute Davos, Biomedical Development, Davos Platz, Switzerland*

**Abstract Text**

**Introduction:** Type-2 diabetes mellitus (T2-DM) is associated with increased bone fragility and higher risk for fracture healing complications. Mouse models enable the study of diabetic bone microarchitecture and fracture healing but often don't resemble human T2-DM.

**Purpose:** Characterization of fracture healing in a high fat diet (HFD)/streptozotocin (STZ)-T2-DM mouse model.

**Methods:** 8-week-old male C57BL/6J mice were fed a low-fat diet (LFD, 10%-kcal fat) (n=8/group) or HFD (60%-kcal fat) (n=16/group) (GR/01/2025, CH) throughout the study. At 12-weeks, T2-DM was induced in HFD-mice (n=8) by 5 daily STZ-injections (40mg/kg) (HFD+STZ). Body weight (BW) was monitored and oral glucose tolerance test (oGGT) performed in 6-hour fasted mice (2g/kg BW glucose/gavage). Animals were stratified (oGGT) and received a 0.66mm-femur osteotomy externally stabilized. Healing was monitored weekly by *in vivo* micro-CT (weeks 16-22). Cortical bridging (CP) was analyzed in 2 perpendicular planes and animals with ≥3 bridged cortices were classified as healed. Trabecular bone parameters (TBP) of contralateral femur (CF) were analyzed by micro-CT at week12+22. Statistics: GraphPad-Prism-v.10.1.2 (one/two-way ANOVA+Tukey post-hoc test; p≤0.05, significant).

**Results:** Prior to STZ, HFD-mice exhibited higher BW (BW-gain: 41.2±7.8%) compared with LFD-mice (16.8±6.9%, p<0.05), STZ-injections halted this increase in HFD+STZ-mice (p<0.001) **(Fig 1A)**. Fasting blood glucose of HFD+STZ-mice was 2-fold higher compared to LFD-mice (t0; p<0.001), and oGTT revealed impaired glucose (p<0.001) **(Fig 2B)**. TBP of CF decreased for all, with most pronounced effects on BV/TV (p<0.05), trabecular number (p<0.05) and increased trabecular separation (p<0.05) seen in HFD+STZ-mice (**Fig 1C**). All LFD-mice showed CP at post-operative week 3, whereas 50% of HFD+STZ-mice were categorized as non-bridged at this time (p<0.05) **(Fig 1D)**.

**Conclusion(s):** HFD/STZ-induced T2-DM impairs trabecular bone-structure and delays fracture healing in male C57BL/6J-mice. Combining this *in vivo*-model resembling hallmarks of human T2-DM, *in vivo*-micro-CT and omics techniques will allow us to characterize this and identify potential treatment targets.

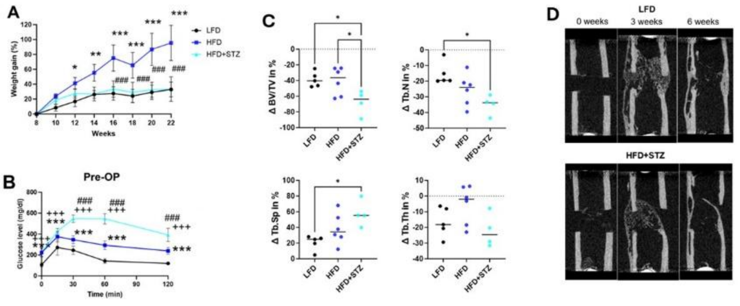

**Fig. 1: BW-gain, oral glucose tolerance test (oGTT), trabecular bone parameters of contralateral femur and in vivo micro-CT of fractured femur of male C57BL/6J mice with low fat diet (LFD), high fat diet (HFD) and HFD+streptozotocin (HFD+STZ). A:** Percentage change (%) in BW compared to initial weight during the study. **B:** Glucose level (mg/dl) after 0, 30, 60 and 120 mins during oGTT 1 week prior to surgery. **C:** Change (Δ) in BV/TV, trabecular number (Tb.N), trabecular thickness (Tb.Th) and trabecular separation (Tb.Sp) of the contralateral femur distal metaphysis assed by micro-CT at 12-weeks of age (prior to STZ-injections) and 22-weeks given in %. **D:** 2D in vivo micro-CT images of defect site of fractured femur. n=4-6/group. A-B: Two-way ANOVA followed by Tukey post-hoc test. *, **, *** (p<0.05, p<0.01, p<0.001; LFD vs. HFD), +++ (p<0.001; LFD vs. HFD+STZ) and ### (p<0.001; HFD vs. HFD+STZ). One-way ANOVA followed by Tukey post-hoc test.

### Poster Sessions: Cancer and bone

### P057

**Langerhans Cell Histiocytosis Mimicking Traumatic Bone Lesion in a Child with T-Cell Acute Lymphoblastic Leukemia**

*Aleksandra Osińska[1], Andrzej Materniak[2], Barbara Rusinowska[1], Balbina Tybulczuk[1], Magdalena Woźniak[2]*
*[1]Medical University of Lublin, Student Scientific Society at the Department of Pediatric Radiology, Lublin, Poland*
*[2]Medical University of Lublin, Department of Pediatric Radiology, Lublin, Poland*

**Abstract Text**

**Background:** Langerhans cell histiocytosis (LCH) is an uncommon clonal disorder of myeloid dendritic cells, often mimicking infectious or traumatic bone conditions. Its recognition can be particularly challenging in immunocompromised children, where symptoms are frequently attributed to treatment-related or post-traumatic causes.

**Case Presentation:** We report a 2-year-old boy receiving chemotherapy for T-cell acute lymphoblastic leukemia (T-ALL) who presented with right elbow pain and swelling after minor trauma. Initial radiographs suggested a post-traumatic lesion or osteomyelitis, and conservative management was initiated. Persistent symptoms and progressive osteolysis led to surgical biopsy, revealing LCH. Whole-body imaging demonstrated multiple osteolytic lesions and small pulmonary nodules, consistent with multisystem disease.

**Discussion:** The coexistence of LCH and T-ALL is exceptionally rare and raises questions regarding shared hematopoietic dysregulation or therapy-related pathogenesis. This case highlights the importance of reconsidering "traumatic" bone lesions in pediatric oncology patients when clinical evolution is atypical or unresponsive to standard management.

**Conclusion:** Early histopathological verification of atypical skeletal lesions is essential to avoid diagnostic delay. Clinicians should maintain a high index of suspicion for LCH in children with hematologic malignancies presenting with persistent or unexplained bone pain, even when a traumatic origin seems plausible.

**P058**

**Combined Chemotherapy and Proton Radiotherapy in Pediatric Cervical Ewing Sarcoma – A Successful Multimodal Approach**

*Aleksandra Osińska[1], Andrzej Materniak[2], Magdalena Woźniak[2], Monika Kamińska[1], Klaudia Marchewka[1]*
*[1]Medical University of Lublin, Student Scientific Society at the Department of Pediatric Radiology, Lublin, Poland*
*[2]Medical University of Lublin, Department of Pediatric Radiology, Lublin, Poland*

**Abstract Text**

**Background:** Ewing sarcoma is a rare, aggressive malignant tumor of bone or soft tissue, most commonly affecting adolescents and young adults. Primary localization in the cervical region is exceptionally uncommon and presents therapeutic challenges due to the proximity of vital neurovascular structures and the spinal cord.

**Case Presentation:** An 11-year-old girl presented with a progressive mass in the left suboccipital and posterior cervical region. Imaging revealed a large soft-tissue tumor extending from the occipital bone to the mid-cervical spine, infiltrating paraspinal muscles and partially encroaching on the spinal canal. Histopathological and molecular analysis confirmed Ewing sarcoma with EWSR1 gene rearrangement.

Following multidisciplinary evaluation, the patient received induction chemotherapy according to the EURO-EWING protocol, achieving significant clinical and radiological regression. The treatment was subsequently continued under the CWS VAIA III and CWS-SoTiSar protocols. For local control, proton beam radiotherapy was administered to the primary tumor region, with escalating doses up to 59.8 Gy RBE and careful spinal cord protection. The therapy was well tolerated, with only grade 2 acute skin reaction.

Chemotherapy was completed with substitution of ifosfamide by cyclophosphamide due to renal toxicity. Post-treatment imaging, including MRI and PET/CT, demonstrated marked structural and metabolic regression with no evidence of active disease. At follow-up, the patient remained in good general condition and without signs of recurrence.

**Conclusion:** This case shows the effectiveness and safety of a multimodal approach combining systemic chemotherapy and proton radiotherapy in pediatric cervical Ewing sarcoma. Proton therapy provided precise local control while minimizing radiation exposure to critical adjacent structures. Continued follow-up is required to monitor long-term outcomes and late toxicity.

**P059**

**Identification of molecular determinants in endosteal cancer cell dormancy**

*<u>Anna Maria Teti</u>[1], Antonio Maurizi[2], Maria Salbini[1], Michela Ciocca[2], Marzia Rea[2], Giuseppe D. Tocchini-Valentini[1], Matilde Merolle[1], Hanna Taipaleenmäki[3], Christina Møller Andreasen[4], Manuela Pellegrini[1]*
*[1]National Research Council, Biochemistry and Cell Biology, Monterotondo Rome, Italy*
*[2]University of L'Aquila, Biotechnological and Applied Clinical Sciences, L'Aquila, Italy*
*[3]LMU Munchen, Musculoskeletal University Center, Planegg-Martinsried, Germany*
*[4]University of Southern Denmark, Department of Clinical Research, Odense, Denmark*

**Abstract Text**

Notch signalling has been linked to breast cancer (BrCa) cell dormancy within the HSC endosteal niche enriched in Spindle-shaped/N-Cadherin$^{HIGH}$ Osteoblasts (SNO). In this study, we detected Notch1 and Notch2 expression in primary human BrCa samples and bone metastases, with Notch1$^{HIGH}$ cells appearing 5.08-fold as often as Notch2$^{HIGH}$ cells ($p<0.001$). Notch levels did not correlate with tumour grade or receptor status, although Notch2$^{HIGH}$ cells were reduced in poorly differentiated tumours (0.41-fold,p<0.04). In vitro, 54.3% of Notch2$^{HIGH}$ MDA-MB231 cells also expressed Notch1. Flow cytometry revealed rare but variable Notch$^{HIGH}$ subsets across various BrCa lines (Notch1$^{HIGH}$ 0.041-3.14%; Notch2$^{HIGH}$ 0.06-3.26%). Notch2$^{HIGH}$ MDA-MB231 cells showed reduced proliferation after SNO contact compared with Notch1$^{HIGH}$ cells (0.57-fold). Yet, experiments using Notch1$^{HIGH}$/Notch2$^{NEG}$ T47D cells confirmed that Notch1 alone does not influence SNO-induced dormancy. RNAseq of MDA-MB231 cells sorted by Notch1$^{HIGH}$/Notch1$^{LOW}$ or Notch2$^{HIGH}$/Nocth2$^{LOW}$ revealed reduced adhesion signatures in Notch1$^{HIGH}$ cells, whereas Notch2$^{HIGH}$ cells displayed enrichment for lysosomal and glycosaminoglycan degradation, as well as downregulation of nucleocytoplasmic transport, spliceosome, cell-cycle and DNA-replication pathways, consistent with dormancy. GSEA identified pluripotency and HSC-like programmes exclusively in Notch2$^{HIGH}$ cells, which expressed CXCR4. Consistently, CXCR4$^{HIGH}$ cells proliferated less (0.55-fold,p=0.0002), and formed fewer (0.44-fold,p=0.0001) mammospheres and less (p=0.02) bone metastasis in mice. Notch2$^{HIGH}$ cells upregulated many ER-stress response genes, with higher PERK phosphorylation (5.09-fold,p=0.0043) and increased CHOP nuclear translocation (2.55-fold,p=0.013). The surface marker CD177 was elevated in Notch2$^{HIGH}$ cells, and its ligands (PLAUR, ITGAM, CEACAM1) were enriched in SNO. Clinically, high CD177, ITGAM, and CEACAM1 expression associated with improved patient survival. Functionally, CD177$^{HIGH}$ cells were also Notch2$^{HIGH}$ and CXCR4$^{HIGH}$, showed reduced proliferation (p=0.01), and preferentially engaged with SNO, reinforcing their dormant phenotype. These findings underscore Notch2 as a key regulator of dormancy and stem-like traits in specific BrCa subsets, mediated through defined surface markers and specialised niche interactions, with potential implications for prognosis and therapy.

**P060**

**ADAR2-dependent Editing Activity on IGFBP7 Induces Osteogenic Terminal Differentiation in Osteosarcoma Cells**

*Michela Rossi[1], Federica Scotto di Carlo[2], Jacopo Di Gregorio[3], Laura Di Giuseppe[4], Sharon Russo[2], Sara Terreri[4], Giulia Battafarano[4], Olivia Pagliarosi[4], Jessica Pepe[5], Salvatore Minisola[6], Fernando Gianfrancesco[2], Angela Gallo[7], Andrea Del Fattore[4]*

*[1]Bambino Gesù Children's Hospital,*
*Bone Physiopathology Laboratory- Translational Pediatrics and Clinical Genetics Research Division, Rome, Italy*
*[2]National Research Council, Institute of Genetics and Biophysics "Adriano Buzzati-Traverso", Naples, Italy*
*[3]University of L'Aquila, Department of Biotechnology and Applied Clinical Sciences- L'Aquila, Rome, Italy*
*[4]Bambino Gesù Children's Hospital,*
*Bone Physiopathology Research Unit- Translational Pediatrics and Clinical Genetics Research Division-Rome- Italy, Rome, Italy*
*[5]"Sapienza" University of Rome, Department of Medical and Cardiovascular Sciences, Rome, Italy*
*[6]"Sapienza" University of Rome, Department of Medical and Cardiovascular Sciences, Rome, Italy*

[7]*Bambino Gesù Children's Hospital, RNA Editing Laboratory, Rome, Italy*

**Abstract Text**

Osteosarcoma is the most common primary bone malignancy in children, with high rate of recurrence and metastasis. Since RNA editing is a key regulator of cancer progression, we investigated the role of ADAR2 (Adenosine Deaminase Acting on RNA 2) editing enzyme in osteosarcoma. We revealed that ADAR2 expression is lower in Saos-2 and 143B osteosarcoma cell lines compared to osteoblasts and inversely correlates to the aggressiveness of tumor cells. Moreover, a microarray dataset demonstrated that osteosarcoma samples exhibited lower levels of ADAR2 compared to human mesenchymal stem cells. To study the role of ADAR2, Saos-2 cells were transfected with vectors overexpressing ADAR2 (pADAR2) or the inactive form of ADAR2 (pADAR2-EA) and with empty vector (pEmpty) as control. The overexpression of ADAR2 in Saos-2 cells reduced the proliferation rate and migration ability (Fig.1a-c) compared to the pADAR2-EA- and pEmpty-transfected cells. Enhanced ability to form mineralized nodules, as shown by Alizarin Red and Von Kossa stainings (Fig. 1d), and increased level of osteogenic markers were revealed in pADAR2-Saos-2. Intratibial injection of pADAR2-Saos-2 cells in NSG mice led to the formation of bone tumor characterized by reduced size (Fig.1e-f) and metastatic ability (Fig.1g). *In vitro* and *in vivo* experiments performed with 143B cells overexpressing ADAR2 confirmed the anti-tumoral effect of this enzyme. Moreover, RNA-seq analysis revealed a significant editing level of IGFBP7 in ADAR2-overexpressing cells compared to controls. Interestingly, the K95R-edited IGFBP7-form fails to stimulate the IGF1R-pathway, and increased the expression of Runx2 compared to WT-IGFBP7-treated cells.

Our results showed that ADAR2 acts as tumor suppressor in osteosarcoma cells and induces the osteogenic terminal differentiation of osteosarcoma cells, opening the way for a novel therapeutic target for this rare tumor.

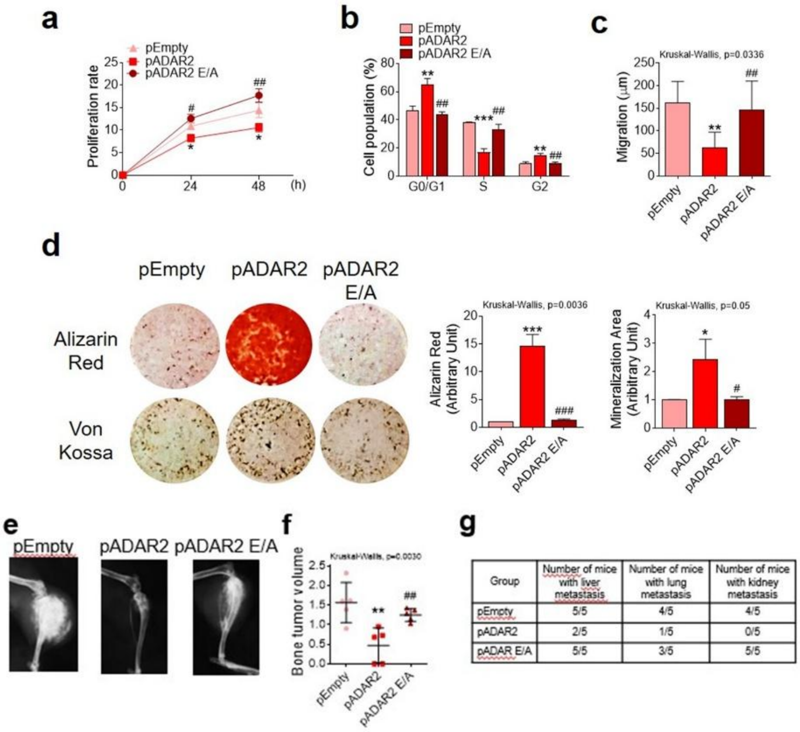

| Group | Number of mice with liver metastasis | Number of mice with lung metastasis | Number of mice with kidney metastasis |
|---|---|---|---|
| pEmpty | 5/5 | 4/5 | 4/5 |
| pADAR2 | 2/5 | 1/5 | 0/5 |
| pADAR E/A | 5/5 | 3/5 | 5/5 |

**P061**

### N-acetyl-L-cysteine impairs osteosarcoma cells aggressiveness: finding new therapeutic opportunities

*Argia Ucci*[1], *Luca Giacchi*[1], *Chiara Puri*[1], *Elisa Pucci*[1], *Kimberly Ferrante*[2], *Stefano Falone*[2], *Nadia Rucci*[1]
[1]*University of L'Aquila, Department of Biotechnological and Applied Clinical Sciences, L'Aquila, Italy*
[2]*University of L'Aquila, Department of Life- Health and Environmental Sciences, L'Aquila, Italy*

**Abstract Text**

Osteosarcoma (OS) is the most common primary bone tumour, predominantly affecting children and adolescents, and is prone to develop drug resistance and lung metastases. Currently, chemotherapy with high-dose methotrexate combined with cisplatin and doxorubicin is the gold standard treatment. The over-the-counter product N-acetyl-L-cysteine (NAC), a reactive oxygen species scavenger, has been demonstrated to exert controversial effects on cancer, however its effect on OS has been poorly elucidated and we aimed at investigating this aspect. Interestingly, treating the human OS cell line MNNG/HOS with NAC for 48h reduced, in a concentration-dependent manner, cell number ($p<0.05$) and metabolic activity ($p<0.05$) compared to untreated cells. We next evaluated any effect of 5 mM NAC on MNNG/HOS proliferation, finding a reduction at 48h ($p=0.0006$). However, apoptosis and cell viability, observed by flow cytometry and trypan blue, respectively, were not affected by NAC. Interestingly, MNNG/HOS pre-treated for 48h with 5 mM NAC also showed a significant lower ability to invade through Matrigel ($p=0.002$). Consistently, zymography performed on conditioned medium collected from MNNG/HOS treated with 5 mM NAC showed a significant reduction of MMP-2 activity ($p=0.008$), compared to untreated cells. Furthermore, an increased expression of the MMPs inhibitor TIMP-1 ($p=0.01$) was observed. Due to the mesenchymal/osteoblastic origin of MNNG/HOS, we also assessed their osteoblast-like profile after treatment with NAC, finding a reduction of the transcriptional expression of the osteoblastogenic markers *BGLAP* ($p=0.0009$) and *RUNX2* ($p=0.01$), the latter confirmed also at protein level ($p=0.0012$). Finally, we also found that 5 mM NAC reduced *PTHrP* expression ($p=0.0091$), while increasing *ANGPT2* ($p=0.0126$) in MNNG/HOS. In conclusion, our data indicate that treatment of OS cells with NAC significantly impairs their aggressiveness. Further experiments will allow to elucidate the mechanisms involved, with the proposal to understand whether NAC could be a valid antitumoral molecule to be used in OS therapy.

**P062**

### Osteoclasts modulate the immune bone metastatic microenvironment by dampening NK function

*Clarissa Orrico*[1], *Matteo Visca*[1], *Monica Parodi*[2], *Giulia Bertolini*[3], *Federico Mussano*[1], *Riccardo Ferracini*[4], *Ilaria Roato*[1]
[1]*CIR Dental School, Surgical Sciences, Turin, Italy*
[2]*IRCCS Ospedale Policlinico San Martino, Immunology Operative Unit, Genoa, Italy*
[3]*Fondazione IRCCS Istituto Nazionale dei Tumori, Unit of Epigenomics and Biomarkers of Solid Tumors, Turin, Italy*
[4]*University of Genoa, Integrated Surgical and Diagnostic Sciences, Genoa, Italy*

**Abstract Text**

**Introduction**: Non-small cell lung cancer (NSLSC) stem cells (CSCs) promote bone metastasis by interacting with osteoclasts (OCs), creating an immunosuppressive environment in the bone pre-metastatic niche.
**Purpose:** To figure out whether NK cells (NK) interfere with the pro-metastatic crosstalk between OCs and CSCs.
**Methods**: In vitro co-cultures of autologous OCs, IL-15-stimulated NK and CSCs from NSCLC A549 cell line (A549s) were set up on plastic and bone slices. OC activity was quantified by TRAP staining, dosage of TRAP enzymatic activity and quantification of resorption area through scanning electron microscope. The expression of NK receptors, ligands, NK degranulation activity and CSC phenotype were studied by flow cytometry.
**Results:** Multinucleated/TRAP+OCs number decreased in OCs+NK co-culture (297±43.5 vs 124±12.2, mean OC number ± SD) and with NK+A549s (297±43.5 vs 54±32.3). OC resorption activity on bone slices decreased with NK (45.6±24 vs 19.8±10.6 mean % resorption area±SD). The cytotoxic NK subset $CD56^{+}CD16^{+}$ increased with A549s (51.4±10.6 vs 69.7±6.3, mean % expression±SD), OCs (51.4±10.6 vs 69.9±6.4), or with OCs+A549s (51.4±10.6 vs 65.9±6.3). NK receptor DNAM-1 expression decreased with OCs (88.2±7.3 vs 66.2±18.7) and OCs+A549s (88.2±7.3 vs 59.5±15.7), while TIGIT expression increased with OCs (49.7±12.8 vs 71.4±12.2) and OCs+A549s (49.7±12.8 vs 74.5±13.7). NK degranulated with A549s (3.5±1.5 vs 17±7.6, mean % CD107a+ cells ± SD), while they lost A549s killing ability when previously co-cultured with OCs (3.5±1.5 vs 4.0±1.9). Anti-TIGIT treatment did not interfere with A549s killing by NK, but it increased the NK degranulation activity with OCs (9.2±4.3 vs 15.3±4.9, mean ± SD,) and OCs+A549s (9.2±4.3 vs 41.4±1.7). A549s expressed NK receptor ligands of DNAM1 and TIGIT without significant modulation in the different co-culture conditions. **Conclusions:** OCs negatively affect NK cytotoxic activity, allowing CSC growth, thus we reported an unrecognized role of OCs in modulating the immune microenvironment by inhibiting NK function.

**P063**

**Anti-tumoural effect of osteoblast-derived extracellular vesicles loaded with methotrexate, doxorubicin, and cisplatin on MNNG/HOS osteosarcoma cells**

*Elisa Pucci[1], Luca Giacchi[1], Argia Ucci[1], Chiara Puri[1], Letizia Clementi[1], Maria Maggi[2], Antonio Angelo D'Archivio[3], Adriano Angelucci[1], Marco Ponzetti[4], Nadia Rucci[1]*
*[1]University of L'Aquila, Department of Biotechnological and Applied Clinical Sciences, L'Aquila, Italy*
*[2]Hortus Novus, Department of Physical and Chemical Sciences, L'Aquila, Italy*
*[3]University of L'Aquila, Department of Physical and Chemical Sciences, L'Aquila, Italy*
*[4]Novo Nordisk A/S, Department of Rare Endocrine Disorders, Copenaghen, Denmark*

**Abstract Text**

Osteosarcoma remains a clinical challenge due to its aggressive nature, high metastatic potential, and poor outcomes in relapsed and refractory cases. Conventional chemotherapy has improved survival but is limited by systemic toxicity, low tumour selectivity, and the frequent development of chemoresistance. The advent of extracellular vesicles (EVs) as natural biological carriers offers new perspectives for drug delivery, combining nanoscale size, intrinsic biocompatibility, and the ability to mediate selective intercellular communication. In this study, we explored human fetal osteoblast-derived EVs (hFOB-EVs) as vehicle for targeted delivery of chemotherapeutics to osteosarcoma cells. We first demonstrated that hFOB-EVs are efficiently internalised by MNNG/HOS osteosarcoma cells; next, we successfully loaded these EVs with methotrexate (MTX), doxorubicin (DXR), or cisplatin (CPT) by employing optimised passive and indirect loading strategies, achieving encapsulated drug levels ranging from 0.7 µg/mL for MTX to 2.9 µg/mL for CPT and 20.4 µg/mL for DXR. Interestingly, treatment of MNNG/HOS with MTX, DXR or CPT – loaded hFOB-EVs reduced cell viability and proliferation, while enhancing apoptosis, *in vitro*. Conversely, treatment of normal cells (i.e. osteoblasts) with drug-loaded-EVs failed to affect their homeostasis *in vitro*, thus indicating the lack of any toxic effect. Finally, in an in vivo osteosarcoma model established through paratibial injection of MNNG/HOS cells in BALB/c nude mice, MTX, loaded hFOB-EVs seems to reduce tumour growth when compared to unloaded hFOB, as evaluated by bioluminescence analysis.In conclusion, these findings highlight hFOB-EVs as safe and functionally competent nanocarriers capable of enhancing the antitumour efficacy of standard chemotherapeutics, opening new avenues for the development of EVs-based therapeutic strategies against osteosarcoma.

**P065**

**Bone Metastasis Induces a ZIP1-Independent, SLC13A5-High Osteoblast Program in the Metastatic Bone Microenvironment**

*Eun Jung Lee*[1], *Serk In Park*[1]
[1]*Korea University College of Medicine, Department of Biochemistry and Molecular Biology, Seoul, Korea- South*

**Abstract Text**

The metastatic bone microenvironment is commonly accompanied by aberrant osteogenic responses, leading to proliferative osteoblasts and immature, woven bone formation. However, the molecular basis of this process remains poorly defined. Bone matrix is enriched with citrate, which is primarily supplied to osteoblasts via apical sodium-dependent citrate transporter SLC13A5, while ZIP1 supports compensatory endogenous citrate production via zinc-dependent rewiring of the Krebs' cycle. We examined the differential expression of SLC13A5 and ZIP1 in the metastatic bone microenvironment using *in vivo* tumor models and human bone metastasis samples.

SLC13A5 and ZIP1 expressions were measured by quantitative RT-PCR in osteoblasts and prostate and breast cancer cell lines. Spatial expression patterns were assessed by in situ hybridization (ISH) in osteoblastic, osteolytic, and mixed bone metastasis tissues from xenograft and syngeneic murine tumor models, including C4-2B (human prostate), MDA-MB-231 (human breast), and 4T1 (murine breast). ISH was additionally performed on human prostate cancer bone metastasis specimens.

SLC13A5 expression was detected exclusively in osteoblasts, with no detectable transcripts in any cancer cell line. In contrast, ZIP1 exhibited broad, non-cell-type-restricted expression. In vivo, C4-2B

and 4T1 models generated substantial metastasis-associated woven bone formation, whereas MDA-MB-231 produced minimal new bones. ISH analyses revealed marked and compartment-specific upregulation of SLC13A5 in osteoblasts lining tumor-induced woven bone, while adjacent trabecular and cortical bone remained low. Human prostate cancer bone metastasis specimens mirrored these findings, with strong SLC13A5 expression confined to osteoblasts at the tumor-induced woven bone interface and minimal expression in pre-existing bone. ZIP1 exhibited diffuse, non-compartment-specific expression (Figure).

Collectively, these findings demonstrate that bone metastasis induces a compartment-specific SLC13A5-high osteoblast program that is mechanistically independent of ZIP1 and uniquely associated with tumor-induced woven bone formation, thereby defining a previously unrecognized osteoblast population absent from normal bone and providing new insight into osteogenic remodeling in the metastatic bone microenvironment.

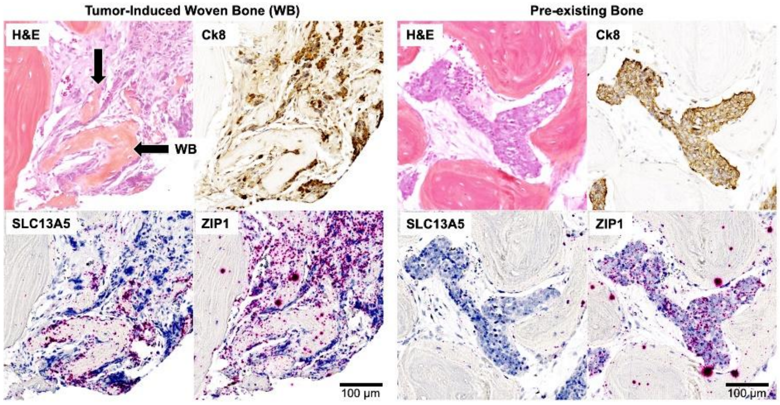

**P066**

**Anticancer effects of Lactobacilli-derived postbiotics on osteosarcoma cells**

*Aikaterini Zoi Lampraki[1], Nerea Alonso Lopez[2], Giovanny Rodriguez-Blanco[2], Antonia Sophocleous[1]*
*[1]European University Cyprus, Department of Life Sciences - School of Sciences, Nicosia, Cyprus*
*[2]Medical University of Graz, Clinical Institute of Medical and Chemical Laboratory Diagnostics, Graz, Austria*

**Abstract Text**

**Background:** Probiotics have shown promise in managing osteoporosis and alleviating pain and disease severity in osteoarthritis patients. Recent research has also highlighted the anti-tumorigenic potential of the gut microbiome, and osteosarcoma tumours have been shown to induce alterations in gut flora. However, the therapeutic potential of probiotics in osteosarcoma remains largely unexplored.
**Purpose:** This study examined the effects of *Lactobacilli*-derived factors on the progression and metastatic potential of K7M2wt osteosarcoma cells *in vitro*.
**Methods:** Sterile filtered cell-free conditioned media (CM) from *Lactobacilli* strains were prepared from overnight cultures and used alongside processed media controls. Metabolomic analysis of these was conducted using an omics-scale targeted metabolomics method employing liquid chromatography coupled with tandem mass spectrometry. Metabolites were separated on a high-pH HILIC HPLC column after 1:20 dilution in an 80% organic buffer. Cell viability, migration, and clonogenic capacity were evaluated using the MTT, wound-healing, and colony formation assays, respectively. K7M2wt osteosarcoma cells were cultured with 10–40% cell-free CM or control media for all experiments.
**Results:** *Lactobacilli*-derived cell-free CM did not alter K7M2wt osteosarcoma cell viability but significantly reduced their migratory capacity in a concentration-dependent manner. Particularly, exposure to 20% and 40% cell-free CM reduced wound closure to (mean±SD) 61.96% ± 45.48 ($p>0.05$) and 31.52% ± 29.97 ($p<0.05$), respectively. Colony formation was also moderately reduced (79.34% ± 12.00 and 83.49% ± 19.19 at 20% and 40% cell-free CM, respectively), although these changes did not reach statistical significance. Metabolomic analysis identified lactate, N-acetylglutamine, 4-aminobutyrate, acetyllysine, O-acetyl-L-serine, and acadesine as the top molecules enriched in the conditioned media. These metabolites participate in amino-acid metabolism, acetylation or stress-response pathways, and converge on central carbon metabolism and AMPK-related signalling.
**Conclusion:** *Lactobacilli*-derived postbiotics exhibit anti-metastatic effects on K7M2wt osteosarcoma cells, although further studies are needed, either alone or combining these metabolites, to elucidate the underlying mechanisms.

**P067**

**Spatial transcriptomics identifies multiple myeloma-intrinsic reprogramming and inflammatory myeloid expansion within the aged bone marrow microenvironment**

*<u>Karl Nyman</u>[1], Ryan Bishop[1], Tao Li[1], Haley du Bois[1], Jeremy Frieling[1], Conor Lynch[1]*
*[1]Moffitt Cancer Center, Tumor Microenvironment and Metastasis, Tampa, United States*

**Abstract Text**

**Background:** Multiple myeloma (MM) is an incurable hematological malignancy whose progression depends on both tumor-intrinsic alterations and remodeling of the bone marrow microenvironment (BMM). The BMM is a complex niche of stromal, immune, and precursor cells that dynamically shape tumor behavior. MM predominantly affects older adults (median diagnosis age ~70), yet most preclinical studies rely on young mice, limiting our understanding of how aging influences disease biology.
**Purpose:** To address this gap, we integrated scRNA-seq and high-definition spatial transcriptomics in age-appropriate MM mouse models to define key molecular and cellular differences in the aged BMM during multiple myeloma.
**Methods:** Young (3-mo; human <20 yrs equivalent) and aged (18-mo; >60 yrs equivalent) immunocompetent C57BL/6 KaLwRij mice were inoculated with luciferase-tagged syngeneic 5TGM1 MM cells. Tumor-naïve age-matched mice served as controls. We evaluated tumor burden, bone destruction, and BMM cellular/transcriptional changes using scRNA-seq and VisiumHD spatial transcriptomics.
**Results:** Although overall tumor growth was comparable between age groups, aged mice showed greater weight loss ($p=0.0039$), bone destruction (TBV: $p<0.0003$, CBV: $p=0.0004$), and prominent paramedullary disease due to cortical bone destruction (20%), confirmed by x-ray and µCT. Integrated single-cell and spatial analyses revealed striking MM-intrinsic transcriptional differences in aged mice, including upregulation of Milk Fat Globule EGF 8 (MFGE8), a secreted protein unstudied in MM but associated with poor patient prognosis ($p<0.0001$). In the aged BMM, we also observed expansion of immature inflammatory myeloid populations ($TNF^+$, $JUN^+$). Neighborhood enrichment and spatial proximity analyses demonstrated preferential localization of these inflammatory and exhausted immune cells near MM cells.

**Conclusion:** Age-appropriate modeling uncovers clinically relevant MM biology obscured in young mouse systems. Aged mice exhibit MM intrinsic transcriptional rewiring and an "inflammaging" niche dominated by immature myeloid cells. These findings highlight aging as a critical modifier of MM progression and a source of potential therapeutic targets.

**P068**

**Human prostate cancer cell dormancy and awakening in the bone microenvironment in vitro**

*Siyuan Ruan*[1], *Iris Van Den Berge*[1], *Jialun Li*[1], *Femke Verhaar*[1], *Johannes Van Leeuwen*[1], *Marjolein Van Driel*[1]
[1]*Erasmus MC, Department of Internal Medicine, Rotterdam, Netherlands*

**Abstract Text**

**Introduction:** Bone is a major metastatic site for prostate cancer (PCa), where metastasis can occur years after primary treatment. Only a minority of disseminated cells can survive in specialized bone metastatic niches, which may be prepared by extracellular vesicles (EVs) secreted by primary tumors. Cancer cells can enter a prolonged dormant state and later get reactivated near bone remodeling sites, which are regulated by osteoblasts and osteoclasts.

**Aim:** To investigate mechanisms driving the dormancy and awakening of PCa cells in the bone microenvironment.

**Methods:** An in vitro co-culture dormancy model was established using differentiating human SV-HFO osteoblasts and GFP-transduced human LNCaP prostate cancer cells for 4 weeks. Cell growth and motility were analyzed by time-lapse microscopy. EVs were isolated from LNCaP supernatant via ultracentrifugation (20,000 g and 100,000 g) and used to prime osteoblasts before co-culture. To study awakening, conditioned media (CM) from osteoblasts at different differentiation stages and from PBMC-derived osteoclasts were added to SV-HFO/LNCaP co-cultures during dormancy.

**Results:** After 4 weeks of co-culture, only a few LNCaP cells survived within the mineralized matrix, exhibiting small, round, and immobile dormant characteristics. EV-primed osteoblasts increased the number of dormant cells by 2.6-fold. EV-priming also caused a significant upregulation of SPARC and ALPL ($p < 0.01$ and $p < 0.05$, respectively), and an upward trend in COL1A1 expression in osteoblasts. Interestingly, a few dormant cells became active after weeks of quiescence when treated with CM from early-stage osteoblasts, but not from late-stage osteoblasts or osteoclast CM. Reactivated cells exhibited enlarged morphology with dendritic protrusions.

**Conclusions:** PCa-derived EVs modify the extracellular matrix of the bone micro-environment, stimulating dormant PCa survival in bone. During new bone formation, as part of the bone remodeling process, factors are released that may play a role in the awakening of dormant cells.

**P070**

**Talazoparib radio-sensitises bone metastases from ER+ve and triple negative breast cancer to Radium-223 increasing antitumour efficacy in bone.**

*Diane Lefley*[1], *Jiabao Zhou*[1], *Matthew Fisher*[1], *Janet Brown*[1], *Helen Bryant*[1], *Spencer Collis*[1], *Penelope Ottewell*[1]
[1]*University of Sheffield, Clinical Medicine, Sheffield, United Kingdom*

**Abstract Text**

**Background:** Breast cancer bone metastases (BCBMs) are incurable, therefore novel therapies are urgently needed. Radium-223 is a calcium mimetic, alpha emitting, radiation and is currently frontline therapy for prostate cancer bone metastasis (PCBM). Radium-223 does not benefit BCBM, possibly because breast cancer is osteolytic resulting reduced exposure of BCBC to bone bound Radium-223, compared with osteoblastic PCBM. DNA damage response inhibitors (DDRi's) are novel cancer treatments which exploit mutations in tumour cells and prevent DNA repair following DNA damage. **We hypothesise that DDRis will sensitise BCBM to Radium-223, increasing the anti-cancer effect of Radium-223 in lytic disease.**

**Methodology:** Optimal combinations of DDRi inhibitors AZD1390 (ATM), AZD6738 (ATR), AZD7648 (DNA-PK), Talazoparib (PARP) and COH34 (PARG), to combine with Radium 223 were tested on mouse triple negative cells E0771 and human ER+ MCF-7 breast cancer cell lines. *In vitro*, experiments were performed in 3% oxygen to mimic the bone environment. Effects on cell number and DNA damage response pathways were assessed by immunohistochemistry. Subsequently, effects of 25ug/kg Talazoparib and/or 27.5kBq/kg Radium-223 on E0771 or MCF7 induced bone metastases in C57BL/6 and BALB/c were assessed by IVIS imaging and uCT.

**Findings:** *In vitro*, combining ATMi, ATRi or PARPi with Radium-223 significantly reduced cell number compared with treatment with Radium-223 alone ($P=0.003$ and $P<0.001$ for ATMi; $P=0.003$ and $P=0.8317$ for ATRi; P-0.001 and $P=0.0006$ for PARPi for E0771 and MCF7 cells respectively). ATRi, DNAPKi and PARGi did not increase Radium-223 induced tumour cell killing. All DDRi's had expected effects on inhibition of DDR pathways.

*In vivo*, Combination of talazoparib and Radium-223 reduced E0771 and MCF-7 BCBM by 55.3% and 93% respectively and increased survival of C57BL/6 mice with E0771 BCBM by 166%.

**Conclusions:** Talazoparib radio-sensitises BCBM to Radium-223 increasing anti-tumour efficacy in bone.

**P071**

### Single-cell spatial characterization of the human bone marrow microenvironment in premalignant myeloma

*Stine Katrine  Rye Østergaard[1], Therese Standal[2], Alexander Rauch[3], Moustapha Kassem[3], Hanne Møller[4], Maja Hinge[5], Thomas Lund[6], Thomas Levin Andersen[7], Marta Diaz Del Castillo[1]*
[1]*University of Aarhus, Department of Forensic Medicine, Aarhus, Denmark*
[2]*Norwegian University of Science and Technology, Department of Clinical and Molecular Medicine, Trondhaim, Norway*
[3]*University of Southern Denmark, Department of Clinical Research, Odense, Denmark*
[4]*University of Southern Denmark, Clinical Institute, Odense, Denmark*
[5]*Lillebaelt Hospital- Department of Hematology-, Department of Hematology-, Vejle, Denmark*
[6]*Vejle Hospital, Department of Hematology, Vejle, Denmark*
[7]*Univesity of Southern Denmark, Department of Clinical Research, Odense, Denmark*

**Abstract Text**

**Introduction:** Multiple myeloma (MM) is an incurable bone marrow plasma cell malignancy always preceded by the premalignant stages monoclonal gammopathy of undetermined significance (MGUS) and smoldering multiple myeloma (SMM), with annual progression risks of ~1% and 10%, respectively. Risk stratification tools can identify high-risk patients, and recent findings suggest that some may benefit from preventive treatment. However, the mechanisms of progression remain unknown. As malignant and premalignant clonal plasma cells share genetic abnormalities, we hypothesize that disease progression is driven by alterations in the bone marrow microenvironment.

**Methods:** To define the cellular and molecular signature of the pro-tumorigenic niche, we applied Xenium High-plex Single cell spatial Profiling (HSSP, 10X Genomics) to formalin-fixed paraffin-embedded (FFPE) diagnostic iliac crest bone biopsies from patients with MGUS or SMM and known progression outcomes within 10 years (n = 10-22), along with freshly collected biopsies from age-matched healthy controls (n=8). Xenium HSSP gene expression profiling included an immune-oncology panel (380 targets) and a custom panel of 100 targets for bone cells, senescence, and metabolic alterations.

**Results:** We obtained superior median transcript per cell count compared with vendor bone datasets in healthy (7  vs 14–121 transcripts/cell) and diseased bone marrow (27 vs 30–217 transcripts/cell). We also achieved a higher number of decoded transcripts per 100µm² in healthy (8.4 vs 136.9–402.9 transcripts/100µm²) and diseased marrow (52.5 vs 245.5–533.6 transcripts/100µm²). Bone tissue was well preserved, and immunostaining enabled visualization of bone structural units based on their lamellar composition. Preliminary analysis identified 31 cellular clusters, which were visually consistent with the expected spatial distribution of bone cell types, including osteocytes, bone lining cells, and mature osteoblasts.

**Conclusion**: Overall, these results demonstrate high-quality single-cell spatial profiling of fresh and archival bone marrow biopsies. Ongoing analyses investigate changes in cell populations and theirs spatial context in the protumorigenic bone marrow.

**P072**

**Mature osteoblast-derived citrate induces a metabolic collapse in acute myeloid leukemia**

*Lotfi Laaouimir*[1], *Hernan A. Tirado*[1], *Nick van Gastel*[1]
[1]*UCLouvain, Cellular Metabolism and Microenvironment Laboratory- de Duve Institute, Brussels, Belgium*

**Abstract Text**

Acute myeloid leukemia (AML) is a malignancy of hematopoietic stem and progenitor cells. Chemoresistance in AML is believed to be attributable to many interacting factors, including metabolic abnormalities and the crosstalk between AML and the cells of the bone marrow microenvironment. Among the different reported bone marrow stromal cell-induced metabolic changes in AML, glycolytic and mitochondrial metabolism and their relation to chemoresistance has received growing interest in the field. However, which stromal cell subset is responsible for these metabolic changes and chemoprotection in AML remains unclear.

Co-culturing AML cells with variety of cell lines representing different stromal cell subsets revealed that osteoprogenitors are the most chemoprotective ($p<0.001$). Intriguingly, mature osteoblasts are not chemoprotective and induce apoptosis in AML cells ($p<0.0001$). Furthermore, RNA sequencing analysis revealed that both glycolytic genes and oxidative phosphorylation genes are significantly reduced in AML cells after coculture with mature osteoblasts ($p<0.01$).

Evaluating the secreted metabolome of mature osteoblasts using GC-MS revealed that they release citrate ($p<0.05$), a metabolite well-known as a glycolytic inhibitor. When exposing AML cells to citrate, we observed a significant decrease in the glycolytic capacity ($p<0.01$) and induction of apoptosis ($p<0.0001$). However, we unexpectedly found that citrate decreases mitochondrial activity in AML cells as well ($p<0.01$). Moreover, we found that citrate-treated AML cells upregulate the activity of cytosolic aconitase ($p<0.01$) by activating a mitochondrial protein called MitoNEET. Interestingly, blocking MitoNEET reverses the citrate-induced ($p<0.001$) and the mature osteoblast-induced cell death in AML cells ($p<0.01$). In addition, inducing AML in $\mathit{Slc13a5}^{fl/fl}\mathit{Ocn}^{Cre+}$ mice, which have more bone marrow citrate, or treating mice daily with citrate, decreased the leukemic burden ($p<0.001$).

These findings indicate that activating MitoNEET could have therapeutic potential in AML, while enhancing the levels of mature osteoblasts at the expense of osteoprogenitors could render chemotherapy more effective in AML.

**Poster Sessions: Osteoblasts and bone formation**

**P074**

**Identifying potential receptors for Wnt1 in ST2 cells**

*Kim M. Wagner[1], Leon Grahn[1], Wenbo Zhao[1], Julia Luther[1], Michael Amling[1], Thorsten Schinke[1], Timur Yorgan[1]*
*[1]University Medical Center Hamburg-Eppendorf, Department of Osteology and Biomechanics, Hamburg, Germany*

**Abstract Text**

Wnt1 is a key modulator of skeletal integrity as demonstrated by pathogenic *WNT1* variants that cause early-onset osteoporosis or osteogenesis imperfecta type XV and by a remarkable osteoanabolic effect of transgenic *Wnt1* expression in mice. Generally, Wnt ligands interact with receptors of the Frizzled (Fzd) family. However, the specific receptor for Wnt1 in the skeletal context remains to be established.

To identify putative receptors of Wnt1 in osteoblasts, we treated mesenchymal ST2 cells and osteoblastic MC3T3-E1 cells with recombinant Wnt1 (rWnt1). 15 days of treatment with rWnt1 during osteogenic differentiation significantly promoted mineralization of ST2 cells, but not of MC3T3-E1 cells (ST2: 0.02 vs 0.64*; MC3T3-E1: 0.67 vs 0.72). Additionally, short-term treatment (6h) with rWnt1 induced the expression of Wnt target genes in ST2 cells, but not in MC3T3-E1 cells, demonstrating that only ST2 cells have the molecular prerequisite to react to Wnt1. Next, we analyzed the expression of all known *Fzd* genes. *Fzd4* (0.002 vs 0.0001*), *Fzd6* (0.002 vs 0.001*) and *Fzd10* (0.014 vs 0.004*) were markedly stronger expressed in ST2 cells than in MC3T3-E1 cells. Furthermore, treatment with rWnt1 significantly repressed expression of *Fzd4* in ST2 cells, reminiscent of a negative feedback response. Subsequently, we set out to explore the relevance of Fzd4, Fzd6 and Fzd10 for the response of ST2 cells to Wnt1 by siRNA-mediated knock down of these receptors. Here, the decreased expression of each candidate receptor was able to modulate the response of the cells upon Wnt1 stimulation. However, we found that knock-down of Fzd4 diminished the transcriptional activation of the target genes *Apcdd1* (2.85 vs 6.46*) and *Ahr* (1.37 vs 3.79*) most strongly and consistently.

Our data suggest that Wnt1 acts via mesenchymal progenitor cells and that this effect is, at least in ST2 cells, predominantly, but not exclusively transmitted via the receptor Fzd4.

**P075**

**Development of a functional genomic screen for osteoarthritis associated genes in osteoblasts**

*Amy Simpson[1], Jessica Petts[1], Gareth Wayne[2], Lorena Benedetti[2], Paul Genever[1]*
*[1]University of York, Biology, York, United Kingdom*
*[2]GSK, Respiratory- immunology and inflammation, Stevenage, United Kingdom*

**Abstract Text**

**Background:** The specific role of osteoblasts in the development of osteoarthritis (OA) is mostly unknown. Our aim was to develop a robust screening tool to assess role of OA-associated genes in osteoblast function, to elucidate novel targets for OA treatment.

**Methods:** We engineered an immortalised clonal MSC line, Y201 which enables reproducible differentiation and further genetic modification. RNA-Seq analysis was conducted on Y201 differentiated into osteoblasts over 21 days. Y201 expressing Cas-9 protein (Cas9-Y201) were generated by lentiviral transduction and knockouts produced by lipofection with sgRNAs. Osteogenic differentiation was measured using ELF™-97 alkaline phosphatase (ALPL) and OsteoImage™ mineralisation assays.

**Results:** RNA-Seq analysis of Y201- osteogenesis demonstrated increased expression of key gene signatures and strong enrichment for primary MSC-derived osteoblast gene sets, validating the Y201 osteogenic model. Gene expression data was used to generate a list of 99 osteoblast expressed genes of interest as potential targets for osteoarthritis. We developed a dual staining protocol allowing assessment of early (ALPL activity) and late (hydroxyapatite deposition) osteogenesis in the same well which showed significant differences between Y201 differentiated in the presence and absence of OA-associated cytokines (IL-1b and OSM). Using Cas-9-Y201 we successfully created knockout populations using an unexpressed control gene (TRAC) and a lethal gene (PLK1) and optimised gene editing efficiency via sequencing and viability assessments respectively. Inhibitory effects on ALPL activity and hydroxyapatite deposition were confirmed using a biological control sgRNA, followed by pilot runs with 8 other sgRNAs. The full screen was run by simultaneously targeting the 99 OA-associated genes in the presence and absence of IL-1b and OSM.

**Conclusion:** We have developed a reproducible, high-throughput functional genomics screening tool in which the effect of OA-associated gene-knockout on osteoblast function can be assessed.

*Y201 cells were used in alignment with the informed consent and LREC approval under which they were collected*

**P076**

**Developing a high-throughput screen to identify druggable regulators of bone formation**

*Jessica Petts[1], Amy Simpson[1], Gareth Wayne[2], Katie Foster[2], Lorena Benedetti[2], Paul Genever[1]*
*[1]University of York, Biology, York, United Kingdom*
*[2]GSK, Respiratory- Immunology & Inflammation Research Unit, Stevenage, United Kingdom*

**Abstract Text**

**Background:** The mechanisms controlling osteogenic differentiation and mineralisation are complex and frequently fail in skeletal disorders. Our aim here was to establish a reproducible cell-based assay to enable simultaneous screening of multiple chemical modifiers of osteogenesis and mineralisation to provide mechanistic insight and identify new targets for drug discovery.

**Methods:** We developed an immortalised human mesenchymal stromal cell (MSC) line (Y201) capable of osteogenic differentiation with the reproducible features required for screening consistency. The Y201 cells were used in alignment with the informed consent and LREC approval under which they were collected. Two fluorescent readouts of early (ELF-97, alkaline phosphatase activity) and late (mineralisation, OsteoImage$^{TM}$) differentiation were adapted for dual-staining in a multi-well plate format over an extended time-course. RNA-Seq was used to validate the Y201 osteogenic model and identify target pathways to explore with pharmacological intervention.

**Results:** RNA-Seq analysis of Y201 differentiation over a 21-day time-course confirmed expression of key gene signatures associated with osteogenesis and bone formation, with most transcriptomic changes occurring between days 0 and 14. Integration of the transcriptomic datasets with publicly available data identified 51 candidate pathways that were targeted by a panel of 91 commercially available small molecules. Confluent Y201 cultures were exposed for 7 days to a blinded panel of the 91 small molecules using a 9-point dose curve with dual-staining performed at day 14. Z-scores were calculated to assess the robustness of the datasets and a four-parameter log fit was performed over the 9-point dose curves for the dual-readouts (Figure 1). From this analysis, top 20 hits were ranked by potency following unblinding. Of these, 9 compounds affected alkaline phosphatase activity, 3 affected mineralisation and 8 affected both.

**Conclusions:** We have developed a high-throughput small molecule plate-based screen that can identify potential druggable targets for the treatment of bone-related disorders.

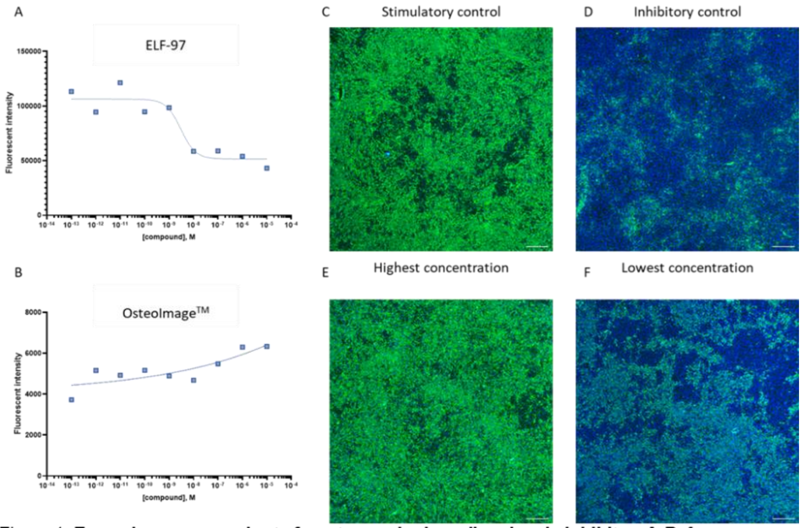

Figure 1. **Example screen readouts for a top-ranked small molecule inhibitor. A-B**: four-parameter log fit dose curves of raw fluorescence intensity for (**A**) alkaline phosphatase (ELF-97, top) and (**B**) hydroxyapatite (OsteoImage$^{TM}$, bottom) readouts. **C-F**: Representative images of hydroxyapatite formation for the inhibitor and relevant controls. Green; OsteoImage$^{TM}$. Blue; DAPI.

**P077**

**Effect of osteoblast-extracellular matrix-functionalized nanostructured titanium surface on osteoblastic and osteoclastic cell behavior**

*Georgia Kors Quiles[1], Paola Gomes Souza[1], Maria Paula Oliveira Gomes[1], Rayana Longo Bighetti-Trevisan[2], Leticia Faustino Adolpho[1], Helena Bacha Lopes[3], Adalberto Luiz Rosa[1], Marcio Mateus Beloti[1]*

[1]University of São Paulo, Bone Research Lab- Ribeirão Preto School of Dentistry, Ribeirão Preto, Brazil
[2]University of Ribeirão Preto, School of Dentistry- University of Ribeirão Preto- Ribeirão Preto, Ribeirão Preto, Brazil
[3]Federal University of Goiás, School of Dentistry, Goiânia, Brazil

**Abstract Text**

**Introduction:** Nanostructured titanium (Ti) drives osteoblast and osteoclast differentiation by modulating various signaling pathways and may enhance osseointegration, potentially through its influence on the formation and remodeling of the extracellular matrix (ECM). **Purpose:** This study aimed to investigate the impact of osteoblast-ECM-functionalized nanostructured Ti surface on osteoblastic and osteoclastic cell behavior. **Methods:** The expression of genes associated with ECM formation and remodeling in MC3T3-E1 osteoblastic cells grown on nanostructured Ti was compared to that on polished Ti surface. Then, MC3T3-E1 cells were cultured on nanotopography, and the cultures were decellularized to generate ECM-functionalized nanostructured Ti surface which was assayed through 4′,6-diamidino-2-phenylindole staining for DNA, fibronectin immunolabeling, scanning electron microscopy, and DNA quantification. To investigate the effect of ECM functionalization on bone cells, either calvaria-derived osteoblasts or RAW 264.7 osteoclastic cells were cultured on both non-functionalized and osteoblast-ECM-functionalized nanostructured Ti surfaces. **Results:** On day 5, the gene expression of ECM markers *Angpt1*, *Itgb1, Postn, Col1a2, Fn1, Timp2, Cdh1, Col10a1*, and *Mmp2* was higher ($p = 0.001$ for all evaluated genes) in MC3T3-E1 cells cultured on nanotopographical surface compared to those grown on polished Ti surface. Decellularization protocol successfully generated an osteoblast-ECM-functionalized nanostructured Ti surface. Functionalization did not affect cell morphology but reduced proliferation ($p = 0.001$), the gene expression of the osteoblastic markers *Runx2* ($p = 0.009$), *Sp7* ($p = 0.029$), *Alpl* ($p = 0.002$), *Bglap* ($p = 0.001$), and *Opn* ($p = 0.029$) while increased ALPL activity ($p = 0.001$) and ECM mineralization ($p = 0.016$). RAW 264.7 osteoclastic cells cultured on ECM functionalized surface exhibited higher TRAP activity than those cultured on non-functionalized surface. **Conclusion:** The functionalization of nanostructured Ti surface created a friendly microenvironment for bone cell activities and consequently bone remodeling, thereby contributing to the development of novel smart osseointegrated Ti implants.

**P078**

### Agrin ablation in osteoblasts compromises long bone structure and osteoblastic differentiation of mesenchymal stem cells

*Gabriela Camarneiro Siqueira[1], Maria Paula Oliveira Gomes[1], Letícia Faustino Adolpho[1], Aline Ying Ying Wang[1], Fabiola Singaretti Oliveira[1], Adriana Luisa Gonçalves Almeida[1], Roger Rodrigo Fernandes[1], Ana Paula Macedo[2], Ana Paula Ramos[3], Adalberto Luiz Rosa[1], Marcio Mateus Beloti[1]*
[1]University of São Paulo, Bone Research Lab- Ribeirão Preto School of Dentistry, Ribeirão Preto, Brazil
[2]University of São Paulo,
Bioengineering Lab- Orthopedics and Anesthesiology Department- Ribeirão Preto Medical School, Ribeirão Preto, Brazil

[3]University of São Paulo,
Chemistry Department- Faculty of Philosophy- Sciences and Letters at Ribeirão Preto, Ribeirão Preto, Brazil

**Abstract Text**

**Introduction:** Agrin participates in maintaining skeletal architecture but the role of osteoblast-expressed agrin in bone tissue remains unexplored. We hypothesized that agrin-deficient osteoblasts negatively affect bone tissue homeostasis by disrupting osteoblastic differentiation of mesenchymal stem cells (MSCs). **Purpose: T**o investigate this hypothesis, animals with agrin deletion in Runx2-expressing osteoblasts were generated using Runx2-Cre mice.
**Methods:** Microtomographic analyses of the femurs, fifth lumbar vertebrae, and calvaria were performed, and the femurs were further analyzed by histology, 3-point bending test, FTIR spectroscopy, and X-ray diffraction. Osteoblastic differentiation of adipose derived MSCs was evaluated on day 7 by gene expression of runt-related transcription factor 2 (*Runx2*), alkaline phosphatase (*Alpl*), collagen, type I, alpha 1 (*Col1a1*), osteocalcin (*Oc)*, and osteopontin (*Opn*), protein expression of RUNX2 and ALPL, and ALPL activity. The effect of osteoblast-specific agrin deletion on the expression of agrin and its receptors α-dystroglycan (*Dag1*), low-density lipoprotein receptor-related protein 4 (*Lrp4*), and muscle-associated tyrosine kinase receptor (*Musk*) was evaluated in adipose-derived MSCs.
**Results:** Microtomographic analyses revealed that agrin ablation in osteoblasts resulted in deleterious effects on the cortical bone of the femurs, resulting in a tendency towards reduced bone stiffness, as indicated by the three-point bending test. This negative impact of agrin-deficient osteoblasts on bone tissue may be partially attributed to the reduced osteoblastic differentiation of MSCs as evidenced by the lower expression of osteoblastic marker genes and reduced ALPL activity. This disruption in osteoblastic differentiation is directly linked to agrin-deficient osteoblasts as demonstrated by the diminished gene expression of agrin and its receptors in MSCs harvested from these animals.
**Conclusion:** Our results highlight the role of osteoblast-secreted agrin in maintaining long bone structure, mainly by promoting MSC differentiation into osteoblasts. These findings indicate that agrin could be an effective therapeutic target for treating bone loss-related conditions, such as osteoporosis and aging.

**P079**

**Non-canonical role of RANKL in osteoblast regulation through LGR4 siganling pathway**

*Kim Jung Ha[1], Kim Kabsun[1], Jun-Il Yoo[2], <u>Wonbong Lim</u>[3]*
*[1]Chosun University, Department of Premedical Science- School of Medicine, GwangJu, Korea- South*
*[2]Inha University Hospital, Department of Orthopaedic Surgery, Incheon, Korea- South*
*[3]Inha University Hospital, Department of Biomedical Research Institute, Incheon, Korea- South*

**Abstract Text**

The receptor activator of nuclear factor κB ligand (RANKL)–RANK axis plays a pivotal role in regulating bone turnover by modulating osteoclast differentiation and bone resorption. RANKL has also been implicated in promoting vascular calcification, a process analogous to bone mineralization. However, it remains unclear whether RANKL contributes directly to bone mineralization through its action on osteoblasts. Here, we demonstrate that RANKL enhances osteoblast differentiation and bone formation via the Wnt/β-catenin signaling pathway in osteoblasts that highly express the leucine-rich repeat-containing G-protein-coupled receptor 4 (LGR4), rather than RANK, the canonical receptor for RANKL. Notably, RANKL harboring mutations that abolish RANK binding retained its ability to promote osteoblast differentiation in vitro and bone formation in vivo, comparable to wild-type RANKL. Furthermore, the stimulatory effects of RANKL with mutated RANK binding sites on osteoblast activity were abolished in the absence of LGR4. These findings suggest that RANKL facilitates osteoblast differentiation and bone formation through LGR4, independently of RANK. Targeting the RANKL–RANK axis for inhibition while simultaneously enhancing the RANKL–LGR4 pathway may represent a promising therapeutic strategy for bone diseases characterized by excessive bone resorption.

**P080**

**Osteogenic differentiation of human induced pluripotent stem cells (hiPSCs) to study the impact of specific gene variants on skeletal integrity**

*Kim Wagner*[1]*, Marina Reinsch*[2]*, Ellen Orthey*[2]*, Timur A. Yorgan*[1]*, Arne Hansen*[2]*, Thorsten Schinke*[1]
[1]*University Medical Center Hamburg-Eppendorf, Institute of Osteology and Biomechanics, Hamburg, Germany*
[2]*University Medical Center Hamburg-Eppendorf,*
*Insitute of Experimental Pharmacology and Toxicology, Hamburg, Germany*

**Abstract Text**

**Introduction:** Human genetic research has identified a large number of monogenic disorders negatively affecting skeletal integrity. To better understand the underlying pathologies in order to establish and optimize bone-specific treatment, a molecular analysis of patient-derived hiPSCs may provide clinically relevant disease-specific information.

**Purpose:** The purpose of this study was to establish a valid osteogenic differentiation protocol for hiPSCs, which can be utilized to analyze the impact of specific genetic variants. Here, we focused on three monogenic disorders caused by pathogenic variants in different genes (*TMEM53, IFITM5,* and *RNU4-2*).

**Methods:** Skin fibroblasts were reprogrammed via Sendai virus-based vectors with four Yamanaka reprogramming factors to generate patient-specific hiPSC lines. Osteogenic differentiation of hiPSCs was induced by dexamethasone, β-glycerophosphate and ascorbic acid. Matrix mineralization (Alizarin Red staining) and gene expression (RNA sequencing and qRT-PCR) was assessed until day 28 of differentiation.

**Results:** We reprogrammed three different hiPSC lines from patients carrying pathogenic variants in *TMEM53*, *IFITM5* or *RNU4-2*. All cell lines maintained normal karyotype and cell morphology and were able to differentiate into all three germ layers. An osteogenic differentiation protocol was first set up with hiPSCs derived from a healthy donor. Using RNA sequencing we observed that all genes required for type I collagen synthesis and processing (e.g. *COL1A1*: 22.5-fold, p<0.0001) as well as SPARC (29.7-fold, p<0.0005) were strongly induced between day 3 and day 28 of differentiation. In contrast, other osteogenic differentiation markers (e.g. *BGLAP* or *IFITM5*) were barely expressed. In this regard it was a potentially relevant observation that the patient-derived hiPSC line (pathogenic variant of *TMEM53*) displayed increased expression of *BGLAP* (d3: 18.46 vs. d28: 4813.22, p<0.0001) or *IFITM5* (d3: 0.41 vs. d28: 10.07, p<0.001).

**Conclusion:** Our initial findings indicate that TMEM53 inactivation may accelerate osteogenic differentiation, which is consistent with the reported high bone mass phenotype of the affected patients.

**P081**

**Eosinophils aggravate iron-dependent development of ectopic bone formation**

*Sven Spangenberg*[1]*, Pauline Porschitz*[2]*, Jens Goronzy*[3]*, Darja Andreev*[2]*, Lorenz Hofbauer*[1]*,*
*Martina Rauner*[1]*, Ulrike Baschant*[1]

[1]*1Dresden University of Technology, Department of Medicine III and Center for Healthy Aging, Medical Faculty and University Hospital Carl Gustav Carus, Dresden, Germany*

[2]*Dresden University of Technology, Center for Regenerative Therapies Dresden CRTD, Dresden, Germany*

[3]*University Hospital Carl Gustav Carus, Orthopaedics- Trauma and Plastic Surgery, Dresden, Germany*

**Abstract Text**

**Background:** Heterotopic ossification (HO) is bone formation at abnormal anatomical sites, developing through the stages of inflammation, chondrogenesis and osteogenesis. Iron homeostasis plays an important role in inflammatory processes and bone formation.

**Purpose:** We aimed to investigate how iron metabolism influences HO development.

**Methods:** Wild type mice were fed with either low (<10 ppm Fe), normal (200 ppm Fe) or high (20,000 ppm Fe) iron diet (HID) for 8 weeks starting from weaning on. Afterwards, HO was induced by injecting recombinant BMP-2 into the M. tibialis anterior and HO sites were analyzed by flow cytometry (day 3), protein analysis (day 3) and µCT analysis (day 14). Iron contents of human HO samples were compared to femoral heads from patients undergoing total hip replacement.

**Results:** HID in WT mice increased HO by 2.6-fold (p<0.05), while low iron diet led to a 65%-decrease (p<0.05) compared to normal iron diet. Notably, iron levels were higher in murine HO tissues as well as in advanced human HO compared to normal bone (+21%; +24%, p<0.05). At day 3, oxidative stress was locally increased in HID mice (MDA concentration by 2.9-fold, p<0.001) as well as inflammatory cell recruitment in the muscle, including classical macrophages and eosinophils (2-fold higher, p<0.01). Along the line, we observed a significant upregulation of pro-inflammatory cytokines (IL-3, IL-4, IL-5 and IL-33 (1.7- to 2-fold, p<0.05)) and platelet-associated growth factors (e.g. PD-ECGF, 3.7-

fold, p<0.01). While macrophage depletion with clodronate had no impact on HO, absence of eosinophils in mice completely prevented the iron-induced increase of HO. Moreover, broad anti-inflammatory treatment with itaconate or dexamethasone effectively reduced iron-induced aggravation of HO (-50% and -70%, p<0.01).

**Conclusion:** Our work highlights the impact of iron homeostasis on HO and identified eosinophils as a potential cellular driver of iron-induced HO aggravation. This suggests new therapeutic options using anti-inflammatory or iron-chelating agents targeting eosinophils.

**P082**

**Characterising zebrafish osteocytes in heath and disease: a foundation for breakthrough discoveries in bone biology**

*Beatriz Laraz-Prieto[1], Jiun Kai Ng[1], Ximena Fernandez Hual[1], Faith Maes[2], Andrii Iakovliev[1], Martin Lee[1], Francesco Turci[3], Paul Eckhard Witten[4], Erika Kague[1]*
*[1]University of Edinburgh, Institute of Genetic and Cancer, Edinburgh, United Kingdom*
*[2]University of Gent, Biology Department, Gent, Belgium*
*[3]University of Bristol, School of Physics, Bristol, United Kingdom*
*[4]University of Gent, Biology Department, Gent, United Kingdom*

**Abstract Text**

Osteocytes, the 'superstars' of bone biology, are now understood to be critical orchestrators of skeletal remodelling. Their complex, neuron-like networks, embedded within the bone matrix, govern mechanotransduction and intercellular communication, processes vital for bone health. Dysregulation of these networks underlies debilitating conditions like osteoporosis and osteoarthritis, impacting millions globally. While zebrafish has emerged as a premier vertebrate model for biomedical research, it lacks a comprehensive characterisation of zebrafish osteocytes, hindering our ability to leverage the powerful genetic tools of zebrafish to investigate fundamental osteocyte biology and translate findings to human health. Here we report the first characterization of osteocytes during the lifespan of zebrafish, using advanced high-resolution imaging systems spanning from confocal microscopy, synchrotron micro-computed tomography, and customised Stimulated Raman Scattering (SRS) 3D confocal microscopy, and provide comparative gene expression profiling.

Our study reveals the presence of functional osteocytes in zebrafish as early as one-month post-fertilisation. Strikingly, in adults, osteocyte distribution mirrors predicted mechanical loading, with sparse populations in low-load regions. Osteocyte orientation aligns perpendicularly to loading direction, and regions of active remodelling exhibit larger, less cylindrical osteocytes amidst disorganized collagen fibres. Consistent with mammalian models, zebrafish osteocytes form a dense, neuron-like network, displaying three distinct populations based on dendritic process complexity. We observed significant variations in process length, including extended, non-branching processes up to 30µm. Furthermore, aged zebrafish display osteocyte morphology consistent with mammalian aging, including rounded lacunae and micropetrosis.

We demonstrate that Sp7, crucial for osteocyte dendritic process formation, functions similarly in zebrafish, as loss-of-function mutants exhibit rounded lacunae and abnormal processes. Notably, zebrafish bones express a broad suite of mammalian osteocyte signature genes. Our detailed characterization establishes zebrafish as a robust model for studying osteocyte biology, revealing conserved architecture and functional responses, particularly in dendritic process regulation, thereby offering new avenues for investigating skeletal adaptation and disease.

**P083**

**Integrated in vivo and in vitro transcriptomics reveal inflammatory drivers of GC-induced bone loss**

*Christoph Kölbl*[1], *Sooyeon Lee*[2], *Sandra Nungeß*[1], *Anita Ignatius*[1], *Jan Tuckermann*[2], *Mubashir Ahmad*[1]
[1]*Institute of Orthopaedic Research and Biomechanics, Ulm University Medical Centre, Ulm, Germany*
[2]*Institute of Comparative Molecular Endocrinology and Physiology, Ulm University, Ulm, Germany*

**Abstract Text**

**Introduction:** Glucocorticoids (GCs) are widely used anti-inflammatory drugs; however, high-dose or long-term administration is a major cause of secondary osteoporosis, known as glucocorticoid-induced osteoporosis (GIO). Despite its clinical relevance, the molecular mechanisms by which GCs suppress osteoblast differentiation and impair bone formation is not entirely understood.
**Purpose:** This study aimed to discover novel signaling pathways and key molecular factors responsible for the detrimental effects of high-dose GCs on osteoblast differentiation and bone formation.
**Methods:** We treated mice with prednisolone (12 mg/kg/day) for two weeks to induce GIO and analyzed primary osteoblasts. We performed quantitative and qualitative alkaline phosphatase (Alp) stainings, qPCR, micro-computed tomography (µCT), histomorphometry, ELISAs, RNAseq, and statistical analyses (unpaired Students *t*-test).
**Results:** *In vivo*, prednisolone treatment markedly reduced trabecular and cortical bone mass (Fig.1A). This bone loss was associated with impaired osteoblastogenesis (Ob.N/B.Pm: 5.02±1.10 vs 0.99±0.58) and enhanced osteoclastogenesis (Oc.N/B.Pm: 8.08±1.98 vs 11.93±2.94), consistent with decreased PINP (4.89±1.02 vs 2.48±0.67ng/mL) and increased CTX-I levels (34.52±5.94 vs 49.01±7.44ng/mL). Humeri cleared of bone marrow from these mice were subjected to RNAseq analysis. In parallel, primary osteoblasts treated with dexamethasone (1 µM) exhibited reduced Alp activity (Fig.1B) and downregulation of osteoblast marker genes (*Sp7, Alpl*). Integrated analysis of the *in vivo* and *in vitro* RNAseq datasets of these samples (Fig.1C), followed by Metascape enrichment analysis, revealed strong enrichment of the inflammatory response-related pathway (Fig.1D). Within this pathway, serum amyloid 3 (*Saa3*) emerged as one of the most highly upregulated genes, suggesting that high-dose GCs induce *Saa3* expression, indicating that this inhibits osteoblast differentiation *in vitro* and suppressing bone formation *in vivo*.
**Conclusion:** Our findings demonstrate that high-dose GCs activate the inflammatory response signaling pathway in both *in vitro* and *in vivo* models, leading to impaired osteoblast differentiation and reduced bone formation. Targeting this pathway may represent a promising therapeutic strategy to mitigate GC-induced bone loss.

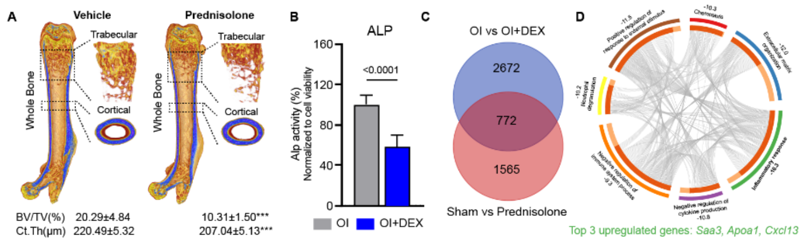

**Fig. 1: Glucocorticoid treatment decreases bone mass and negatively influences osteoblast differentiation through altered inflammatory response pathway.** (A) Representative μCT images of femurs from mice treated either with vehicle or prednisolone (12 mg/kg/day) for two weeks, highlighting trabecular and cortical bone parameters such as bone volume fraction - BV/TV (%), and cortical thickness - Ct.Th (μm) (n=6-7/group). (B) Cellular alkaline phosphatase activity after 6 days of dexamethasone (DEX) treatment during osteogenic differentiation (OI) (n=6/group). (C) Venn diagram illustrating the common differentially expressed genes (DEGs) between *in vivo* and *in vitro* glucocorticoid treatment. (D) Metascape analysis of significantly regulated genes identified seven highly enriched pathways. Corresponding Log*p* values are displayed in the circular plot. Overlapping genes in different pathways are indicated by interconnecting grey lines. Within the most enriched pathway, the top three regulated genes were identified regarding Log2FC. Statistical differences between the two groups were determined using unpaired Student's *t*-test.

### P084

### A humanised self-structuring bone model (SSBM) as a 3D in vitro bone formation platform

*Karthik Vaidya*[1,2]*, Melissa Finlay*[1]*, Joseph Weightman*[2,1]*, Ellie Northall*[1]*, Jonathan Lewis*[1]*,*
*Mohamed Patel*[3]*, Pola Goldberg Oppenheimer*[3]*, Liam M. Grover*[3,2]*, Amy J. Naylor*[1]

[1]*Department of Inflammation and Aging, University of Birmingham, United Kingdom*

[2]*Healthcare Technologies Institute, University of Birmingham, United Kingdom*

[3]*School of Chemical Engineering, University of Birmingham, United Kingdom*

### Abstract Text

Humanised in vitro bone models are increasingly sought as alternatives to animal testing, yet most fail to support osteocyte development, despite osteocytes being central regulators of bone remodelling through factors such as RANKL and sclerostin. We developed and characterised a humanised self-structuring bone model (SSBM) that enables long-term 3D culture and supports differentiation of osteoblasts into mature osteoblasts/osteocyte-like cells. Using this system, we assessed differentiation of the human osteoblastic cell line hFOB 1.19 over twelve weeks and compared outcomes with conventional 2D monolayer culture and human adipose-derived stem cells (ADSC).

Cell viability and metabolic activity remained stable throughout culture. Collagen fibril production (P1NP ELISA) was detected at 4, 8, and 12 weeks. Mineral deposition appeared from the culture midpoint, with an increased 1044 $cm^{-1}$ Raman peak confirming progressive mineral maturation. Osteoblast maturation markers rose markedly over time: podoplanin (PDPN) increased 25-fold, runt-related transcription factor 2 (RUNX2) 2.7-fold, and bone morphogenetic protein 2 (BMP2) 76.2-fold at the gene level. In contrast, 2D hFOB 1.19 cultures failed to express markers such as PDPN or dentine matrix acidic phosphoprotein 1 (DMP1) and detached by week 8, preventing further differentiation.

Primary ADSC cultured as SSBMs under osteogenic induction produced a more mature mineral phase than hFOB-derived SSBMs and additionally expressed late osteocyte markers such as sclerostin, which was absent from hFOB cultures. These findings further demonstrate the system's capacity to support advanced osteogenic maturation.

In summary, SSBMs sustain long-term osteoblast culture and promote differentiation toward osteocyte-like cells, enabling formation of a mature, mineralised matrix. This positions SSBMs as a robust, human-relevant platform for bone anabolic drug discovery and for studying bone formation, cancer metastasis, cancer-cell dormancy, and bone remodelling.

**P085**

**Molecular Phenotyping of bone-forming cells**

*Atenisa Caci*[1,2], *Nicholas Ditzel*[3], *Moustapha Kassem*[1,2], *Alexander Rauch*[1,2]
[1]*University of Southern Denmark, Department of Clinical Research, Odense M, Denmark*
[2]*Odense University Hospital, Molecular Endocrinology Department- KMEB, Odense M, Denmark*
[3]*The Faculty of Health Sciences, Department of Molecular Medicine, Odense M, Denmark*

**Abstract Text**

**Background**: The intrinsic ability of human bone marrow stromal cells (hBMSCs) to form bone is highly heterogeneous. The osteogenic capacity of individual clonal MSC lines varies significantly *in vivo*, even under identical culture circumstances.

**Purpose**: Characterize the transcriptional diversity present in high and low bone-forming MSC clones and investigate if paracrine signals can convey osteogenic capability between clones.

**Methods**: Assess the cellular diversity of clonal cell lines (high/low bone-forming: HBF/LBF) through scRNA-seq analysis and measure both *in vitro* and *in vivo* osteogenic capabilities.

**Results**: Cells in LBF demonstrated robust *in vitro* osteogenesis but weak *in vivo* bone formation, suggesting the absence of pro-osteogenic signals (A). scRNA-seq demonstrated differences in the cellular composition between HBF and LBF clones (B, bar plots). HBF clones showed enrichment for cells with Hippo signaling (~40%, 4-fold higher than LBF), while LBF clones showed an increase in cells with PI3K-mTOR pathway activity (~50%, 3-fold higher than HBF). Analysis of autocrine communication revealed that HBF clones have a more robust and prevailing signaling network (C). Bone formation assays in immunodeficient mice show that mixed-clone implants generated less bone than expected from their ratios, indicating that HBF cells do not promote bone formation by LBF cells via paracrine mechanisms (D). Mixed effects modeling validated notable clone variations (HBF: LBF 75:25 $p = 0.0084$; HBF: LBF 50:50 $p = 0.0058$, compared to the expected linear distribution). Interestingly, HBF but not LBF cells showed increased ALP activity in response to activators (Truli) of Hippo signaling *in vitro,* supporting the notion that the bone-forming capacity of HBF cells is linked to the cellular response rather than the expression of paracrine factors affecting Hippo signaling.

**Conclusion**: Collectively, these data suggest that intrinsic sensitivity to Hippo signaling, driven by uniquely transcribed HBF states, plays a crucial role in determining the functional osteogenic capability.

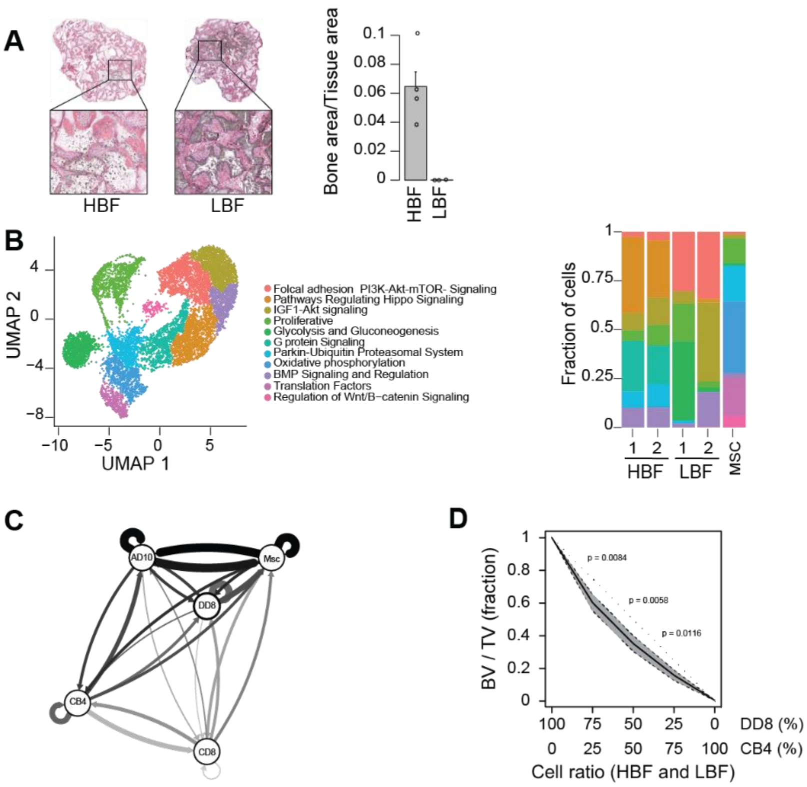

**P086**

**A Chromatin-Licensed GR–MZF1 Axis Links Physiological Differentiation to Glucocorticoid-Induced Osteoporosis**

*Dalia Ali[1,2], Martin Rønn Madsen[3], Mathilde Palmier[1,2], Atenisa Caci[1,3], Moustapha Kassem[1,2], Susanne  Mandrup[3], Alexander Rauch[1,2]*

*[1]University of Southern Denmark, Department of Clinical Research, Odense, Denmark*

*[2]Odense University Hospital,*
*Department of Endocrinology- Molecular Endocrinology & Stem Cell Research Unit KMEB, Odense, Denmark*

*[3]University of Southern Denmark,*
*Department of Biochemistry and Molecular Biology- Functional Genomics & Metabolism Research Uni t, Odense, Denmark*

**Abstract Text**

**Background:** Based on empirical knowledge, glucocorticoids are indispensable for *in vitro* differentiation towards human osteoblasts and adipocytes, yet their therapeutical use is a leading cause of secondary osteoporosis. How glucocorticoid receptor (GR) signaling and chromatin architecture couple physiological differentiation to pathological bone loss in human stromal cells remains poorly understood.

**Methods/Approach:** We combined GR, MED1 and H3K27ac ChIP-seq, DNase-seq, and RNA-seq with motif-activity modelling (IMAGE) to determine lineage-selective chromatin actions of GR and transcription factors that linked glucocorticoid response to osteoblast-selective genes. Candidates were prioritized through integrating genomic coordinates of motifs with eBMD SNPs and connected to glucocorticoid induced inhibition of osteoblast differentiation by loss of function studies *in vitro* and ectopic transplantation assays.

**Results:** GR binding licensed lineage-selective and glucocorticoid-dependent acetylation of H3K27 (fig. A) driving transcriptional programs for both osteoblast and adipocyte differentiation in a largely concentration-independent manner. It indicated a binary chromatin "on-switch" rather than a graded dose sensor. Motif-activity and GWAS enrichment ($p = 0.104$) nominated Myeloid Zinc Finger 1 (MZF1) as a GC-dependent regulator of osteoblast-selective genes (fig. B) and a member of a senescence signature during late stage of high-dose glucocorticoid exposure (fig. C). Genetic depletion of *MZF1* attenuated senescence (fig. D), and restored osteoblast differentiation and bone formation in transplantation assays (fig. E) under high-dose glucocorticoids.

**Conclusions:** Our data identify GR as a chromatin licensing factor for early human stromal cell specification and uncover MZF1 as a glucocorticoid-responsive, senescence-linked brake on osteoblast differentiation. This chromatin-anchored pathway provides a mechanistic framework and potential intervention point for glucocorticoid-induced osteoporosis in humans.

**ND-P008**

**Cereblon regulates osteogenic differentiation in mouse primary cultured cells**

*Yu-Mi Yang*[1], *Dong Min Shin*[1]
[1]*Yonsei University College Of Dentistry, Department of Oral Biology, Seoul, Korea- South*

**Abstract Text**

**Background:** Cereblon (CRBN) is a multifunctional protein located in the cytoplasm, nucleus, and peripheral membranes of the brain and other tissues. CRBN is closely related to the regulation of ion transport, modulation of AMP-activated protein kinase (AMPK) signaling pathways, and metabolism, especially cell proliferation and apoptosis, in cell and whole tissues or organs. It can lead to several diseases including cardiovascular disease, obesity, diabetes, and fatty liver by CRBN-mediated inactivation of AMPK. However, the role and the molecular mechanism of CRBN in differentiation of bone cells remain unknown.

**Purpose:** In this study, we investigated the role of CRBN in osteogenic differentiation using CRBN knock-out (KO) mice.

**Methods:** In vivo animal studies, mice of wild-type (WT) and CRBN KO (8-10 weeks old) were analyzed the morphology and mass levels of the bones using micro-CT and histopathological sections. In vitro, isolated bone marrow-derived monocytes/macrophages (BMM) and osteoblast precursor cells from male murine long bones. We investigated osteoclastogenesis via RANKL-mediated differentiation and functions. Osteoblast differentiation and function were assessed by alizarin red staining and gene expression analysis (qRT-PCR).

**Results:** The deletion of *CRBN* gene was markedly increased the bone density of the femur (~1.36-fold), especially in male CRBN KO mice (~1.52-fold). We found that CRBN KO cells enhanced potency of osteoblastic differentiation compared to WT mice using methods of alkaline phosphatase and alizarin red staining, but osteoclastogenesis was not affected. We also identified that the mRNA expression of osteogenic genes significantly regulated by treatments of blockers of $Ca^{2+}$ and $K^{+}$ channels during osteoblast differentiation of CRBN KO cells compared to WT cells.

**Conclusion:** These results suggest that CRBN plays a critical role for osteoblastogenesis in bone remodeling.

**Poster Sessions: Osteoclasts and bone resorption**

**P087**

**Establishment of a bone resorption assay using human CD14+ monocyte-derived osteoclasts**

*Mervi Ristola*[1], *Jenni HE Mäki-Jouppila*[1], *Jukka P Rissanen*[1], *Katja M Fagerlund*[1]
[1]*Pharmatest Services, Ltd, Turku, Finland*

**Abstract Text**

**Introduction.** Human osteoclasts can be derived from peripheral blood CD14+ monocytes and bone marrow-derived CD34+ osteoclast precursor cells in the presence of M-CSF and RANKL. Robust preclinical *in vitro* bone resorption assays utilizing human osteoclasts are essential to test novel therapeutic agents for osteoporosis and other bone-related disorders.
**Purpose.** This study describes the establishment of an *in vitro* human bone resorption assay based on CD14+ monocyte-derived osteoclasts cultured on natural bone matrix. The effects of odanacatib and zoledronic acid on bone resorption activity were studied in this culture system and the drug responses were compared with the corresponding data obtained in our previously established and validated bone resorption assay utilizing human CD34+ osteoclast precursors.
**Methods.** Human peripheral blood CD14+ monocytes were cultured on bovine bone slices in 96-well plates for 21 days in the presence of RANKL and M-CSF, allowing them to differentiate into mature osteoclasts. Odanacatib (0.001 – 1 µM) was added to the cultures twice a week from day 10, and zoledronic acid (0.001 – 500 µM) was added once on day 10. Tartrate-resistant acid phosphatase (TRACP) staining was performed to assess osteoclast differentiation, and C-terminal crosslinked telopeptides of type I collagen (CTX-I) was measured in the culture medium as an index of bone resorption activity.
**Results.** CD14+ monocyte culture differentiated into TRACP-positive multinucleated osteoclasts, and odanacatib and zoledronic acid showed concentration dependent inhibition of osteoclast resorption activity. EC50 values for odanacatib and zoledronic acid were 0.038 µM and 0.088 µM, respectively, and corresponded with the EC50 values obtained in human CD34+ osteoclast precursor-derived bone resorption assay (0.0433 µM for odanacatib, 0.192 µM for zoledronic acid).
**Conclusions.** The established human CD14+ monocyte-derived osteoclast culture system provides a reliable bone resorption assay, where odanacatib and zoledronic acid serve as validated reference compounds, enabling the identification of novel drugs with anti-resorptive activity.

**P088**

**Inflammatory Pathways underlie differential Responses to M-CSF, RANKL and OPG in osteoclast precursor cells**

*Joyce Kimenai*[1,2,3]*, Asma Mechakra*[1,3]*, Jess. G. Snedeker*[1,3]*, Sander Botter*[2]
[1]*ETH Zurich, Department of Health Science and Technology, Zurich, Switzerland*
[2]*Balgrist Campus AG, Swiss Center for Muskuloskeletal Biobanking, Zurich, Switzerland*
[3]*Balgrist University Hospital Zurich- University of Zurich, Department of Orthopedics, Zurich, Switzerland*

**Abstract Text**

**Background:** Donor-to-donor variation in osteoclastogenesis is a problem to overcome in terms of experimental planning and execution, especially when focusing on medication development.

**Aim:** This study focusses on osteoclast differentiation of $CD14^{++}/CD16^{-}$ classical monocytes under the influence of macrophage colony-stimulating factor (M-CSF), receptor activator of NF-kB ligand (RANKL) and osteoprotegerin (OPG) and explore the intrinsic differences between donors in varying conditions.

**Materials and methods:** $CD14^{++}/CD16^{-}$ classical monocytes were isolated and treated with Macrophage Colony-stimulating factor (M-CSF), M-CSF + Receptor Activator of Nuclear Factor-κB ligand (RANKL), and or M-CSF + RANKL + 30, 45 or 60 ng/ml of Osteoprotegerin (OPG) (OPG30, OPG45 and OPG60 respectively) for 11 days. Osteoclast differentiation potential of 22 donors was explored using morphological staining. An in-depth analysis of eight selected donors were performed with RNA sequencing. Specific measures were taken to reduce technical variability as much as possible.

**Results:** After cell morphology staining, two subsets of donors were identified. One subset responding well to RANKL and OPG as expected, while having a low osteoclastogenesis in the presence of M-CSF only. RNA sequencing data showed that inflammatory-related pathways (Figure 1A and Figure 1B), important in the non-canonical differentiation process of osteoclasts, were down-regulated in cells from a subset of the donors, which showed minimal differences between the conditions, showing relative high osteoclast formation in the presence of M-CSF only, while the effects of RANKL and OPG were limited (Figure 1C).

**Conclusion and discussion: I**ntrinsic differences were found between the two groups of donors in terms of activation of inflammatory pathways and morphological differences. Donor variation in osteoclastogenesis can potentially be explained by the differential expression in canonical and non-canonical inflammatory pathways. Awareness of these two sub-populations of donors, will aid to understand, as well as minimize variation in *in vitro* osteoclast differentiation.

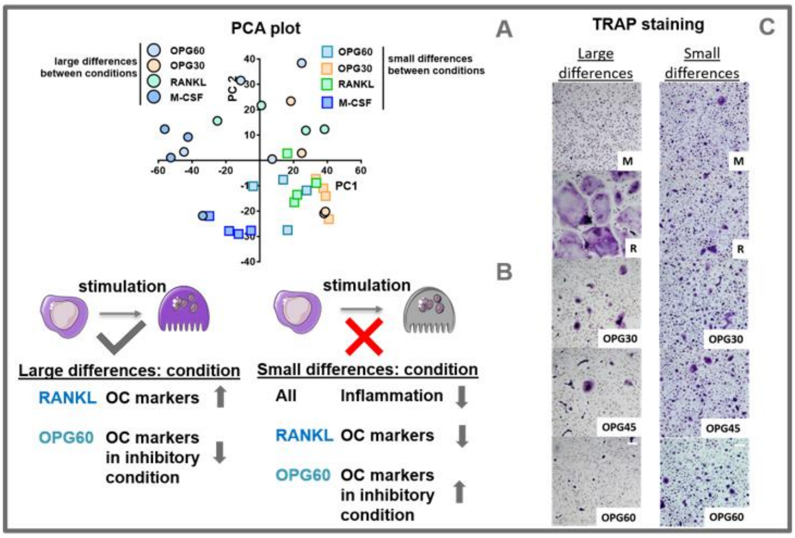

Figure 1. Donor sub-populations explained.
A: A PCA plot shows two distinct donor sub-populations: one with large differences between conditions and another with only small differences, allowing them to be clearly separated. B: Pathway analysis revealed lower inflammatory signaling in the subset of donors that displayed small differences between conditions. C: Visual comparison of two representative donors. One donor responds as expected to M-CSF (M), M-CSF + RANKL (R), and increasing concentrations of OPG (OPG30–60), while the other donor shows only minimal differences across these conditions.

**P089**

**Role of Purine Metabolism in Osteoclastogenesis and Inflammatory Bone loss.**

*Simrin Patrao*[1]*, Aline Bozec*[1]
[1]*Uniklinik Erlangen, Medicine 3, Erlangen, Germany*

**Abstract Text**

**Background/Introduction:** Chronic inflammatory diseases such as rheumatoid arthritis (RA) result in osteoclast-mediated bone loss and secondary osteoporosis. Although osteoclasts are highly metabolically active, their regulation under inflammatory conditions remains poorly understood. In our recent studies, increased levels of the purine metabolites inosine and hypoxanthine were detected during treatment with semi essential amino acid, L-arginine under inflammatory conditions, coinciding with reduced osteoclastogenesis. This led to the hypothesis that inosine could also potentially reduce osteoclast differentiation.

**Purpose:** This study investigates the mechanistic role of purine metabolites, particularly inosine, in regulating osteoclast differentiation within inflammatory arthritis induced bone loss models.

**Methods:** Differentiating mouse osteoclasts were treated with 20 mM inosine at early (day 0) or late (day 2) stages, including parallel cultures supplemented with TNFα and quantified using traditional TRAP staining. Untargeted mass spectrometry was performed to assess metabolic changes, and RNA-sequencing was used to identify differentially expressed genes. For *in vivo* validation, a collagen-induced arthritis (CIA) model was established in DBA/1J mice. They were immunized twice with type

II collagen and complete Freund's adjuvant, followed by treatment with 400 mg/kg inosine via oral gavage every other day from day 23 onward accompanied by recording inflammatory scores.

**Results:** *In vitro*, inosine significantly reduced osteoclast numbers at both early and late treatment time points, including under TNFα stimulation (Fig a-c). Untargeted mass spectrometry and RNA-sequencing revealed enrichment of cysteine and methionine metabolic pathways and the upregulation of related genes Arg1, Cxcl14, and Gstt4. *In vivo*, inosine treatment did not significantly alter inflammatory scores in CIA mice but resulted in a significant reduction in osteoclast numbers compared with untreated controls (Fig d-g).

**Conclusion(s):** These findings demonstrate that inosine impairs osteoclast differentiation, potentially through modulation of the cysteine–methionine pathway. Further investigation is required to clarify its importance and to assess the  molecular pathway and therapeutic potential of inosine supplementation in inflammatory bone loss.

**Figure**

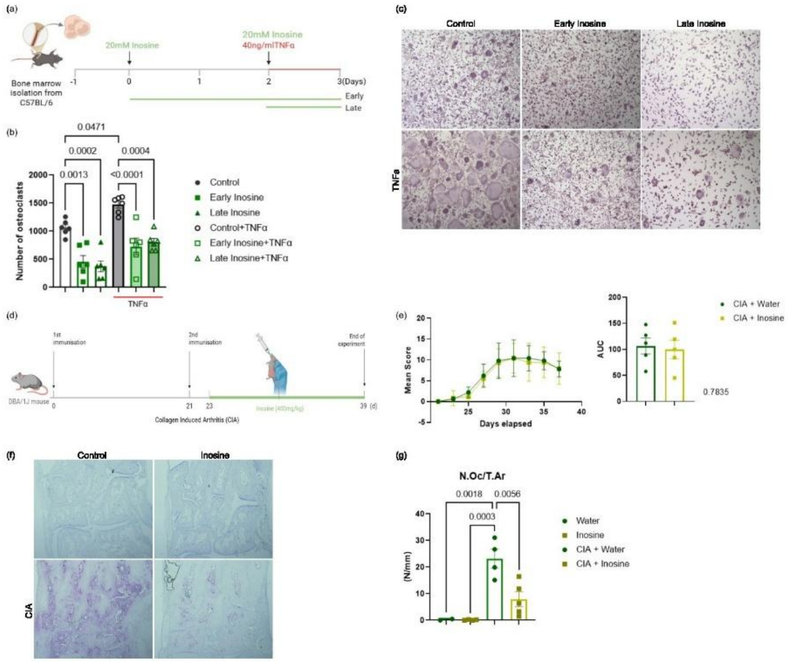

(a) Schematic diagram for *in vitro* osteoclastogenesis from bone marrow cells of C57BL/6 J mice(n=6); suplemented with Inosine (20mM) at day0 (Early) or day2 (Late) +/-TNFα. (b,c) Quantification of TRAP+ osteoclasts and representative TRAP staining images. (d) Schematic diagram for *in vivo* collagen induced arthritis (CIA) model in DBA/1J mice (n=5 per group) treated with 400mg/kg inosine via oral gavage every alternate day from day23. (e) Arthritis score and quantification of area under the curve (AUC) of arthritis. (f,g) Representative TRAP staining images of the paw and quantification of osteoclast number/tissue area (N.Oc/T.Ar).

**P090**

### Integrated transcriptomic and functional analysis revealed sexual dimorphism in mature osteoclasts

*<u>Camille Girolet</u>[1], Valeriia Rezapova[1], Julia Halper[1], Adrien  Mahler[1], Maria Materozzi[1], Abdelilah Wakkach[1], Claudine Blin-Wakkach[1]*
*[1]Université Côte d'Azur, Lp2m - UMR 7370, Nice, France*

**Abstract Text**

Sexual dimorphism in bone mass and osteoporosis susceptibility is well established and involves multiple factors that influence bone-cell differentiation and function. Among these cells, bone-resorbing osteoclasts (OCLs) are key regulators of bone homeostasis and belong to the myeloid lineage, which is well known for its diversity and sexual dimorphism. As such, OCLs are heterogeneous, comprising subsets with distinct roles in bone resorption, and immune and metabolic processes.

Studies of OCL sexual dimorphism have mainly focused on bone resorption, whereas potential differences in their immune function remain unexplored. Our aim was therefore to characterize murine OCLs from both sexes using complementary approaches to better define this dimorphism with a specific focus on immune processes.

OCLs were differentiated from bone marrow cells of 8-week-old male and female mice with RANK-L and M-CSF and characterized at the phenotypic and functional level.

Comparative scRNA-seq data on purified mature OCLs from males and females revealed greater differences in pathways related to immune processes than osteoclastogenesis (FDR=0.002 and 0.05, respectively). It also uncovered sex-specific differences in the representation of previously defined OCL clusters. Cluster C0, strongly associated with bone resorption (*Acp5, Ctsk*) was 40% more abundant in males. In contrast cluster C1, characterized by an inflammatory gene signature (*Fcgr2b, Fcgr3*) and C2, combining high resorption and inflammatory features, were 13% and 100% more abundant in females, respectively. Functional assays showed that inflammatory OCLs from females exhibit higher antigen-processing capacity (1.2-fold, p-val<0.05). They were also more efficient than male OCLs in stimulating CD4+ and CD8+ T-cell proliferation (1,8-fold and 1.3-fold, p-val<0,01 and <0,0001, respectively).

In conclusion, by revealing major sex-dependent differences in OCL composition and immune properties, our findings provide new insights into OCL sexual dimorphism and suggest that sex-specific therapeutic strategies may be beneficial in pathologies involving OCL dysfunction.

**P091**

**Role of I-BAR proteins in osteoclast formation and function.**

*Eleonora Ledaki Engonopoulou[1], Renaud Poincloux[1], Arnaud Labrousse[1], Christel Vérollet[1], Nathan Pavlos[2], Shiro Suetsugu[3], Fita Nilasari[3], Claudine Blin-Wakkach[4]*
*[1]IPBS, Pharmacology and Structural Biology, Toulouse, France*
*[2]Head- Bone Biology & Disease Laboratory, Head of Research Cluster-*
*Tissue Repair & Regenerative Biology, Perth, Australia*
*[3]Nara Institute of Science and Technology, Cell biology, Osaka, Japan*
*[4]CNRS Université de Cote d'Azur, Molecular Physiomedicine, Nice, France*

**Abstract Text**

Bone is a dynamic tissue that undergoes continuous remodeling through the coordinated actions of osteoblasts, which are responsible for bone formation, and osteoclasts, which mediate bone resorption. Osteoclasts are large, multinucleated cells that are derived from myeloid precursors and are specialized in degrading the mineralized bone matrix. Disruptions in osteoclast formation or function, due to aging, chronic inflammation, or bone tumors, can deregulate this balance and lead to skeletal disorders such as osteoporosis. Current treatments for osteoporosis, such as bisphosphonates, reduce osteoclast viability. However, because osteoblast and osteoclast functions are closely interconnected, these treatments can also impair bone formation, highlighting the need for more targeted therapeutic strategies.

Osteoclastogenesis involves the fusion of myeloid precursors through the formation of tunneling nanotubes and the acquisition of bone-resorbing capacity, both of which depend on dynamic actin cytoskeleton remodeling. My project focuses on I-BAR proteins, key regulators of membrane curvature and actin dynamics. In particular, IRSp53 has been described as a crucial linker between Rho-GTPase signaling and the actin cytoskeleton, regulating protrusion and migration in other cell types. However, their involvement in osteoclast differentiation and function remains poorly explored.

To investigate this, we use two osteoclast models, human osteoclasts derived from peripheral blood monocytes and murine osteoclasts derived from immortalized myeloid precursors. Using siRNA and CRISPR–Cas9 approaches, we individually knock out I-BAR proteins, including IRSp53, to study their roles in osteoclast fusion, cytoskeletal organization, and bone-resorbing capacity.

By identifying novel regulators of osteoclast function, this project aims to provide new mechanistic insights and should pave the way for the development of more precise therapies for bone diseases.

**ND-P009**

**Circulatory properties of osteoclast progenitor population in streptozotocin-induced mouse model of type 1 diabetes mellitus**

*<u>Marta Radošević</u>[1], Ozana Jaksić[1], Maša Filipović[1], Darja Flegar[1], Pavao Planinić[2], Tomislav Kelava[1], Edi Rođak[3], Sanja Novak[4], Nataša Kovačić[5], Ivo Kalajzić[6], Danka Grčević[1]*
*[1]University of Zagreb School of Medicine, Department of Physiology and Immunology, Zagreb, Croatia*
*[2]University of Mostar School of Medicine, Department of Physiology, Mostar, Croatia*
*[3]University of Osijek- Faculty of Medicine Osijek, Department of Histology and Embryology, Osijek, Croatia*
*[4]University of Arkansas for Medical Sciences, Department of Physiology and Cell Biology, Little Rock, United States*
*[5]University of Zagreb School of Medicine, Department of Anatomy, Zagreb, Croatia*
*[6]UConn Health, Center for Regenerative Medicine and Skeletal Development, Connecticut, United States*

**Abstract Text**

**Background:** Osteoclast progenitors (OCPs) of monocyte/macrophage lineage can be identified in bone-marrow, spleen, circulation and other hematopoietic tissues. Inflammation may drive recruitment of circulatory OCPs to bone surfaces and their maturation into bone-resorbing osteoclasts. Although chronic inflammatory state is associated with type 1 diabetes (T1D), with elevated cytokines and advanced glycation end-products, detailed characterization of OCP properties in T1D remain limited.

**Purpose**: To detect OCP subsets and assess their migratory potential in a mouse model of streptozotocin (STZ) induced T1D.

**Methods:** T1D was induced in male mice via STZ i.p. injections (50 mg/kg for 5 days). Bone-marrow and spleen CX3CR1+ OCPs were phenotyped and sorted by flow-cytometry to perform in vitro migration assays in a Traswell-system. In vivo migration of CX3CR1+ OCPs was evaluated using CX3CR1GFP reporter knock-in/knock-out mouse strain. Lineage tracing of CX3CR1+ progenitors was performed using inducible CX3CR1CreERT2/Ai9(TdTomato) mouse strain, after tamoxifen treatment.

**Results:** Flow-cytometry confirmed the presence of CX3CR1+ OCPs (CD45+CD3−B220−NK1.1−Ly6G−CD11blow/+CD115+) in bone-marrow and spleen of STZ-treated mice, with the additional expression of activation markers (RANK, CCR2, CD64, TLR4, CD27). T1D-derived OCPs exhibited enhanced migration in vitro, when cultured in media containing chemokines CCL2 or CX3CL1, with CCL2 having a more potent chemotactic effect. Bone sections of STZ-treated homozygous CX3CR1GFP mice (lacking CX3CR1 surface expression) had fewer GFP+ cells than heterozygous CX3CR1GFP littermates (having CX3CR1 surface expression). Parabiosis between heterozygous CX3CR1GFP control and wild-type T1D mouse confirmed the migration of GFP+ cells, detected on the bone sections of wild-type counterpart. Lineage tracing of CX3CR1+ TdTomato-labeled OCPs confirmed their migration to bone surfaces and fusion into multinucleated TRAP+ cells.

**Conclusions**: We found that CX3CR1+ OCPs increasingly express activation receptors in T1D and may be attracted from circulation to bone surfaces, suggesting their contribution to bone resorption in T1D. Funding: NPOO 10106-25-3013 (MEDIS), NPOO 10106-25-2987 (LIOS), HRZZ-IP-2022-10-2285, DOK-NPOO-2023-10-4220.

**Poster Sessions: Osteocytes and mechanobiology**

**P093**

**Age-Related Functional Changes in the Osteocyte Network During Healing of Critically Sized Bone Defects**

*Lea Gellee[1], Amel Manel Batouche[2], Claudine Boiziau[1], Sylvie Rey[1], Perrine Combet[1], Katharina Jähn-Rickert[2], Delphine B Maurel[1]*
*[1]Université de Bordeaux, Bioingénierie Tissulaire- INSERM U1026, Bordeaux, France*

[2]University Medical Center Hamburg-Eppendorf, Department of Osteology and Biomechanics, Hamburg, Germany

**Abstract Text**

The repair of critically sized bone defects remains a major clinical challenge. A deeper understanding of the mechanisms regulating bone regeneration is essential to identify new therapeutic targets. Aging delays bone regeneration, a phenomenon associated with structural and functional alterations in bone cells. Historically, functional analysis of osteocytes was limited due to their entrapment within the mineralized bone matrix. It is now well established that osteocytes play key roles in skeletal homeostasis through their lacuno-canalicular network, and recent studies have suggested their involvement in bone repair. Morphological alterations of the osteocyte network have also been reported with aging.

This study aimed to characterize the roles of osteocytes during the healing of a critically sized calvarial defect in mice and to assess the impact of aging on these processes. Bilateral 3.5-mm calvarial defects were created in 12- and 72-week-old male C57BL/6 mice. Animals were sacrificed at 3, 14, and 28 days post-surgery to analyze osteocyte morphology and communication during early, intermediate, and late phases of bone repair. Control mice without defects were included. Regions located close (0-600 µm) and far (1000-1600 µm) from the defect edges were examined using histology, immunostaining, and confocal microscopy.

We observed a significant reduction in the thickness of the newly formed bone at the defect edges in aged mice compared to adults (18.5 ± 12.7 µm vs. 40.8 ± 17.1 µm, $p < 0.0001$). At day 14, adult mice with defects displayed osteocytes with more dendritic processes in the far region than controls (27.2 ± 3.8 vs. 17.0 ± 2.7 dendrites/osteocyte, $p < 0.0001$), a difference not observed in aged mice. Confocal imaging also revealed location-dependent differences in the expression of molecules involved in cell communication.

These findings suggest that osteocyte network organization and communication are altered with aging, potentially impairing bone regeneration mechanisms.

**P094**

**3D polyurethane scaffolds as a platform for mechanical stimulation and osteogenic differentiation of mesenchymal stromal cells**

*Theresa-Maria Böhm[1], Sigrid Müller-Deubert[1], Melanie Krug[1], Camilla Mussoni[2], Philipp Stahlhut[2], Maximilian Rudert[3], Benedikt Kusan[1], Denitsa Docheva[1], Taufiq Ahmad[2], Regina Ebert[1]*

*[1]University of Würzburg, Department of Musculoskeletal Tissue Regeneration, Würzburg, Germany*

*[2]University of Würzburg, Department of Functional Materials in Medicine and Dentistry, Würzburg, Germany*

*[3]University of Würzburg, Department of Orthopedic Surgery, Würzburg, Germany*

**Abstract Text**

**Background:** Bone marrow-derived mesenchymal stromal cells (MSC) differentiate into osteogenic lineages and contribute to bone regeneration through biochemical and mechanical cues, such as fluid-flow and compression. These mechanoresponsive processes can be modeled in vitro using bioreactors combined with suitable 3D scaffolds.

**Purpose:** This study aimed to establish a 3D polyurethane (PU) scaffold as a platform to investigate mechanical stimulation and osteogenic differentiation of human MSC.

**Methods:** Primary human MSC (n=8) were cultured on 3D PU scaffolds and exposed to defined fluid-flow (2.5, 3.75, 5 ml/min) and 10% compression regimes. Mechanosensitivity was assessed by quantifying mechanoresponsive genes FOS and PTGS2. Cultures were maintained in expansion medium for up to 21 days or osteogenic medium for up to 14 days; controls included 2D PU and polystyrene (PS) substrates. Cell attachment and morphology were analyzed using SEM and F-actin staining. Osteogenic differentiation was evaluated by qPCR for early (RUNX2, ALPL), osteoblastic (SPP1, BGLAP), and osteocytic (PDPN, DMP1) markers. Relative expression values were calculated using the ΔΔCt method; statistical analyses used the Relative Expression Software Tool. Mineral deposition was confirmed by Alizarin Red staining and ICP-MS.

**Results:** Cells adhered and spread throughout 3D PU scaffolds, exhibiting pore-spanning morphologies. Gene expression showed material- and time-dependent upregulation of osteoblastic markers, with PU scaffolds promoting stronger responses than PS, even without osteogenic supplements. 3D PU additionally demonstrated mineralization confirmed by Alizarin Red and ICP-MS. Mechanical stimulation induced clear, time- and stimulus-dependent regulation of FOS (up to 60-fold, $p>0.01$) and PTGS2 (up to 22-fold, $p>0.001$), with distinct patterns for each loading mode.

**Conclusion:** 3D PU supported hMSC adhesion, osteogenic differentiation, and mechanical responsiveness, highlighting its suitability as a versatile platform for in vitro studies of osteogenic differentiation and mechanobiology.

### Poster Sessions: Chondrocytes and cartilage

### P095

### The Beta-2 Adrenergic Receptor Protects Against Cartilage Degeneration and Osteophyte Formation During Posttraumatic Osteoarthritis in Aged Mice

*Paul-Richard Knapstein*[1], *Anke Baranowsky*[1], *Johannes Keller*[1]
[1]*University Medical Center Hamburg-Eppendorf, Department of Trauma and Orthopedic Surgery, Hamburg, Germany*

**Abstract Text**

**Background/ Introduction:** Osteoarthritis (OA) is the most prevalent human joint disease. Early disease stages often involve tissue damage caused by mechanical instability or impaired cartilage metabolism. In recent years, the beta2-adrenoreceptor (Adrb2) and its primary ligand noradrenaline have been identified in playing a crucial role in bone and joint homeostasis, both of which are dysregulated during posttraumatic OA disease progression.

**Purpose:** To investigate the extent to which Adrb2-dependent signal transduction affects the progression of OA in both young (low sympathetic tone) and aged mice (elevated sympathetic tone).

**Methods:** OA was induced via anterior cruciate ligament (ACL) transection on the right knee in both *Adrb2*$^{+/+}$ and *Adrb2*$^{-/-}$ mice at the age of 14 and 30 weeks. Articular cartilage degeneration, synovitis, bone erosion, and osteophyte formation were scored histologically 4 weeks after surgery. Micro-computed tomography evaluation was conducted to measure the subchondral bone microstructure and osteophyte formation. Cartilage thickness and bone remodeling were assessed histomorphometrically.

**Results:** In 14-week-old mice with low sympathetic tone, Adrb2-deficiency did not affect cartilage degeneration, synovitis, bone erosion score, ratio of hyaline to calcified cartilage thickness, or osteophyte formation in posttraumatic OA. Radiological analysis showed no changes in subchondral bone loss or osteophyte number. In 30-week-old mice with increased sympathetic tone, however, *Adrb2*$^{-/-}$ mice showed an increase in cartilage degeneration, bone erosion score and osteophyte size and maturation. Radiological analysis showed an increase in osteophyte number and volume in mutant mice, while subchondral bone parameter remained unchanged.

**Conclusion(s):** Adrb2-deficiency exacerbated the post traumatic cartilage degeneration and increased osteophyte formation in aged mice. These findings suggest that Adrb2 may represent a novel therapeutic target for post-traumatic OA in the aging organism. Here, pharmacological agonization of Adrb2 could potentially aid in the prevention or treatment of disease progression, particularly following ACL injury or reconstruction.

**P096**

**Immunobased profiling of knee osteoarthritis patients to predict response to autologous platelet-rich plasma injection: 1-year follow-up**

*Antonio Tonutti[1], Valentina Granata[2], Veronica Marrella[3], Rita Ragusa[1], Chiara Camisaschi[4], Simone Puccio[3], Emanuela Morenghi[5], Nicola Rani[6], Berardo Di Matteo[7], Carlo Selmi[1], Cristina Sobacchi[3], Angela Ceribelli[1]*

*[1]Humanitas Research Hospital IRCCS, Dep Rheumatology and Clinical Immunology, Rozzano, Italy*

*[2]Humanitas Research Hospital IRCCS, Lab Cellular and Humoral Innate Immunity, Rozzano, Italy*

*[3]CNR, IRGB Milan Unit, Rozzano, Italy*

*[4]Humanitas Research Hospital IRCCS, Flow Cytometry Core, Rozzano, Italy*

*[5]Humanitas Research Hospital IRCCS, Biostatistics Unit, Rozzano, Italy*

*[6]Rizzoli Orthopedic Institute, Conservative Orthopedic Surgery and Innovative Techniques, Bologna, Italy*

*[7]Humanitas Research Hospital IRCCS, Division of Orthopedics, Rozzano, Italy*

**Abstract Text**

**Background**: Knee osteoarthritis (OA) is a degenerative musculoskeletal disease of multifactorial origin causing chronic disability. Different OA endotypes have been postulated but no biomarker can predict individual response to treatments. We recently reported the mid-term follow-up analysis in a small cohort of early knee OA patients receiving autologous platelet-rich plasma (PRP) injection[1].

**Purpose**: To define the peripheral blood profile predictive of response to PRP injection at 1-year follow-up in our cohort.

**Methods**: multicenter study; inclusion criteria: men/women, aged 40-75 years; knee OA grade 2-3, BMI<40. Analyses on PB samples prior to PRP injection (nSTRIDE): multiparametric FACS analysis of the adaptive and innate immune cell compartment followed by unsupervised cluster analysis (CRUSTY), as described[1]; comprehensive analysis of inflammatory and antioxidant pathways (qPCR, ELISA, enzymatic assays). Patient follow-up until 1 year after treatment. Response to treatment defined based on WOMAC questionnaires and established clinical criteria. Statistical analysis: Mann-Whitney test.

**Results**: Among 143 enrolled patients (56 males, 87 females), 64 were responders and 32 non-responders at both 6-month and 1-year follow-up. These two subgroups did not differ as for age, sex, and BMI, but responders had longer symptom duration and higher frequency of failed conservative therapy. In the serum, responders had higher total antioxidant capacity ($p<0.05$); higher CD40L, Groa, MIP3b ($p<0.01$), eotaxin, IL1RA, IP10 ($p<0.05$) and MCP1 ($p=0.052$); and lower TNFa, IL10 ($p<0.001$), IL6 ($p<0.01$), PTX3, VEGF, IFNg ($p<0.05$) and G-CSF ($p=0.054$). Moreover, responders had more circulating $CD14^+$ ($p<0.01$) and $CD4^+$ ($p<0.05$) cells, featuring predominantly a memory phenotype (higher CM $CD4^+$ and lower TE $CD4^+$ cells, both $p<0.05$), and lower Treg ($p<0.001$). CRUSTY analysis is ongoing.

**Conclusions**: Our study analysis delineated an immunophenotype associated with both early and sustained response to PRP treatment in our cohort. We intend to validate these results in an independent cohort.

1. 1. Tonutti A et al. Biomedicine & Pharmacotherapy 2025

**P097**

**Role of Apex1 in the molecular mechanism of chondrocyte hypertrophy**

*Miguel Echanove Gonzalez De Anleo[1], Megan Keech[2], Emma Muiños Lopez[1], Tania López Martinez[1], Purificación Ripalda Cemborain[1], Craig Duvall[2], Froilan Granero Moltó[1]*
*[1]Universidad de Navarra, Orthopedic Surgery and Traumatology, Pamplona, Spain*
*[2]Vanderbilt University, Vanderbilt Institute of Nanoscale Science and Engineering, Nashville, United States*

**Abstract Text**

**Background**: Osteoarthritis (OA) is a degenerative disease of the articular cartilage. Currently, pharmacological treatments are only palliative, joint replacement being the only available treatment option for this growing issue. Thus, a deeper understanding of the molecular mechanisms at play is key for identifying novel molecular targets. We identify *Apex1* as an essential player during endochondral ossification, controlling chondrocyte differentiation and hypertrophy.

**Purpose**: We hypothesize that inhibition of *Apex1* should delay articular cartilage degeneration and OA progression.

**Methods**: All animal procedures were approved by our institutional committee for animal use in research. To induce OA, a destabilization of the Medial Meniscus (DMM) were performed on 8-weeks-old C57BL6/J mice, where *Apex1* was silenced in chondrocytes using AcanCreER$^{t2}$ (AcanCre;Apex1$^{C/C}$), AcanCre$^{-}$ or Apex1 heterozygous littermates were used as WT controls. For siRNA inhibition studies, C57BL6/J mice were subjected to a mechanical loading protocol and received intra-articular (IA) injections of siRNA targeting Apex1 or a scramble control.

**Results:** Histological analysis of the knees, Toluidine blue staining, from the AcanCreER$^{t2}$ showed slowed advancement of OA, quantified through scoring of the destruction of the articular cartilage, $p<0.05$ (Figure 1A). There is also a more intense staining of the mutant mice knees than the WT counterparts. The knees of mechanically loaded mice, with intra-articular injections, were also analyzed through histology and results were comparable to the DMM KO mice (Figure 1B). A reduction in the damage to the articular cartilage was evident, as well as, a more intense staining in the mice that received the IA injection. Analysis of the cartilage through qPCR showed reduced expression of MMP13.

**Conclusion:** The inhibition of *Apex1* delays the advancement of OA as evidenced by the quantification of the articular destruction. Targeting of *Apex1* through siRNA exhibited similar results to those of the DMM KO mice.

**P098**

**IL-1β and OSM differentially modulate BMP4/ TGF-β3-induced chondrogenic differentiation of human FAPs involved in neurogenic heterotopic ossifications**

*<u>Juliette Fernandez</u>[1], Marion Grosbot[1], Sébastien Banzet[1], Dorothée Girard[1]*
*[1]IRBA, Hauts de Seine, Clamart, France*

**Abstract Text**

**Neurogenic heterotopic ossifications (NHO)** are intramuscular ectopic bone formations that develop after central nervous system (CNS) injury, such as spinal cord injury (SCI). Occurring near major joints, they cause pain, ankylosis, and neurovascular compressions, severely impairing patient recovery. CNS injury–induced inflammation profoundly alters muscle microenvironment and promotes chondro-osteogenic differentiation of fibro-adipogenic progenitors (FAPs) through the action of proinflammatory macrophages and cytokines such as IL-1β and oncostatin M (OSM). Evidence of circulating BMP4+ monocytes following SCI has been reported.

This study investigated the effect of BMP4 on TGF-β3–induced chondrogenic differentiation of human FAPs *in vitro*, as well as the impact of IL-1β and OSM on FAPs chondrogenic differentiation.

Human PDGFRα$^{+}$CD56$^{-}$ FAPs were isolated from muscle adjacent to NHO lesions and sorted by flow cytometry. $2.5 \times 10^5$ FAPs were cultured as spheroids in TGF-β3 (10 ng/mL) chondrogenic medium supplemented with BMP4 (50 ng/mL). The effects of IL-1β and OSM (0.1, 1, and 10 ng/mL) on BMP4/TGF-β3–induced chondrogenesis were assessed. After 28 days, spheroids were analyzed by Safranin O/Fast Green staining, RT-qPCR for chondrogenic gene expression, and immunohistochemistry for SOX9, aggrecan, and type II collagen. Statistical analyses were performed using Friedman tests.

BMP4 significantly enhanced Safranin O staining, indicating increased sulfated proteoglycan production. It also upregulated the expression of chondrogenic transcription factors (*SOX9*, *SOX5*, *SOX6*) and extracellular matrix genes (*COL2A1*, *COL10A1*, *ACAN*) compared with TGF-β3 alone (Friedman test, $p < 0.05$). Immunostaining confirmed increased SOX9, collagen II, and aggrecan protein expression. Higher IL-1β concentrations at 1 and 10 ng/mL significantly inhibited the expression of chondrogenic markers, whereas OSM had no significant effect.

These findings demonstrate that human FAPs involved in NHO exhibit *in vitro* chondrogenic potential and provides the first evidence that BMP4 strongly enhances their differentiation capacity, while inflammatory cytokines exert distinct modulatory effects on FAPs differentiation.

**P099**

### A transcriptional atlas of the pubertal human growth plate reveals two populations of cartilage stem cells.

*Nelson Chu[1], Ostap Dregval[1], Farasat Zaman[2], Claes Ohlsson[1], Lars Sävendahl[2], Igor Adameyko[3], Andrei Chagin[1]*
*[1]The University of Gothenburg, Medicine, Gothenburg, Sweden*
*[2]Karolinska Institute, Women's and Children's Health, Stockholm, Sweden*
*[3]Medical University of Vienna, Neuroimmunology, Vienna, Austria*

**Abstract Text**

The cartilaginous growth plate is a critical organ responsible for longitudinal bone growth. It closes after puberty in humans, but remains open throughout life in mice. Although cartilage stem cells have been identified in murine growth plates, their existence in humans remains unknown. Here, we characterized the cellular and molecular organization of early pubertal human growth plates using unique surgical specimens from growth-restricting procedures.

Single-cell and spatial analyses revealed two distinct stem-like populations in the resting zone, differing in proliferative activity, molecular identity, and regulatory cues. The root population consisted of PTHrP-negative cells residing in a Wnt- and TGFβ-low microenvironment and expressing multiple skeletal stem cell markers. A similar population was identified in transcriptionally profiled unsorted murine growth plates, and clonal lineage tracing demonstrated that PTHrP-negative root cells generate extensive chondrocyte clones and differentiate into stromal and osteoblastic lineages, confirming their stem cell properties.

These findings uncover a conserved dual stem cell organization in human and mouse growth plates, providing a framework for optimizing growth-promoting therapies.

**Poster Sessions: Endocrine factors**

**P101**

**Thyroid hormones modulate glycolysis in mouse primary osteoblasts and bone tissue**

*Franziska Brinkmann[1], Eleonore Kreß[1], Lorenz C. Hofbauer[1], Elena Tsourdi[1], Martina Rauner[1]*
*[1]Medical Faculty and University Hospital Carl Gustav Carus- Dresden University of Technology, Department of Medicine III & Center for Healthy Aging, Dresden, Germany*

**Abstract Text**

Thyroid hormones (TH) are key regulators of metabolism, however, their effects on bone cell metabolism are not well understood. Osteoblasts primarily rely on glucose as their main energy source, generating ATP predominantly through aerobic glycolysis. While TH stimulate osteoblast differentiation and function, the specific energy pathways that support these processes remain unclear.

Here, we analyzed long bone tissue from hyperthyroid and hypothyroid C57BL/6J mice to screen for glycolysis-related genes using real-time qPCR analysis. Additionally, primary murine osteoblasts were treated with 100 nM triiodothyronine (T3) to examine the effects of TH on glycolysis at both the transcriptional and protein levels using real-time qPCR, Western Blot analysis and Glucose-Glo™/Lactate-Glo™ assays. To further dissect the pathway, glycolysis was inhibited at different stages - initiation, intermediate, and final steps - using WZB117, Az-PFKFB3-67, and sodium oxamate (OXA), respectively.

In bone, hyperthyroidism led to an increased expression of glycolysis-related genes such as glucose transporter *Slc2a4* (12.4-fold; P=0.013), hexokinase 2 (*Hk2*: 4.0-fold; P=0.002), 6-phosphofructo-2-kinase/fructose-2,6-biphosphatase 3 (*Pfkfb3*: 3.1-fold, P=0.002), and lactate dehydrogenase a (*Ldha*: 4.7-fold; P=0.022) as compared to bone tissue from euthyroid controls. In contrast, *Slc2a4* (-13.9%; P=0.011) and *Ldha* (-55.2%; P=0.047) mRNA levels were decreased in hypothyroid bone tissue. *In vitro*, T3-stimulated mature osteoblasts showed an elevated expression of *Slc2a4* (3.0.fold; P=0.007), *Pfkfb3* (1.8-fold; P≤0.001), and *Ldha* (1.2-fold; P=0.010), and increased protein levels of GLUT4 (1.4-fold; P=0.064), HK2 (1.8-fold; P=0.017), PFKFB3 (1.5-fold; P=0.007) and LDHA (1.3-fold; P=0.264) together with enhanced glucose consumption (2.8-fold; P≤0.001) and lactate production (1.3-fold; P=0.002). While T3 increased the expression of osteoblast marker genes *Bglap* (8.7-fold;

P≤0.001) and *Sp7* (2.1-fold; P≤0.001) as well as the ALP activity (1.7-fold; P=0.008), additional treatment with each glycolysis inhibitor normalized TH effects on osteoblast activity.

In conclusion, our findings indicate that the effects of TH on osteoblast function may rely on upregulated glycolytic activity.

**P102**

### Metabolic dysfunction-associated steatotic liver disease and bone fragility: from liver fibrosis to trabecular bone score

*Cecilia Oliveri*[1], *Clelia Asero*[1], *Maria Stella Franzè*[1], *Adele Di Giovanni*[1], *Concetta Pitrone*[1], *Roberto Filomia*[1], *Gaia Caccamo*[1], *Carlo Saitta*[1], *Irene Cacciola*[1], *Giorgio Basile*[1], *Nunziata Morabito*[1], *Antonino Catalano*[1]

[1]*University of Messina, Clinical and Experimental Medicine, Messina, Italy*

**Abstract Text**

**Background and Aims:** Metabolic dysfunction–associated steatotic liver disease (MASLD) is increasingly recognized as a multisystem condition with possible skeletal involvement. Metabolic abnormalities such as visceral adiposity, insulin resistance, and chronic inflammation may impair bone quality, yet the mechanisms contributing to fracture risk in MASLD remain unclear.This study aimed to evaluate bone fracture risk in patients with MASLD using the trabecular bone score (TBS), an index of bone microarchitecture quality.

**Methods:** One-hundred-eight patients with MASLD were included in the study. Participants underwent liver stiffness measurement (LSM) and Controlled Attenuation Parameter (CAP) evaluation by FibroScan, as well as dual-energy X-ray absorptiometry (DEXA) with TBS assessment and bone fragility fracture risk assessment by FRAX.

**Results:** The study population showed median LSM and CAP values of 7.5 kPa (5.8–13.9) and 298 dB/m (255–324), respectively. Sixty-three patients (58.9%) had a pathological TBS (<1.350), and 55 (52%) presented subclinical vertebral fractures by vertebral fracture assessment (VFA) via DEXA. Patients with pathological TBS had higher BMI ($p < 0.0001$), LSM > 8 kPa ($p = 0.038$), higher CAP ($p = 0.007$), and higher LDL-c levels ($p = 0.035$). TBS values were inversely correlated with CAP and visceral adiposity index ($p = 0.006$; $p = 0.003$), and directly correlated with lumbar spine and femoral BMD ($p < 0.0001$; $p = 0.002$). Patients with vertebral fractures showed higher BMI ($p = 0.001$), LSM ($p = 0.027$), CAP ($p = 0.010$), and more frequently pathological TBS ($p < 0.001$). At univariate and multivariate logistic regression analysis, independent predictors of pathological TBS were BMI > 30 $kg/m^2$ ($p < 0.001$), CAP > 298 dB/m ($p = 0.020$), and higher total FRAX score ($p = 0.027$).

**Conclusions:** TBS and VFA assessment may be useful tools for evaluating bone fragility and enabling early detection of silent vertebral fractures in MASLD patients.

**Poster Sessions: Energy metabolism, fat, diabetes, nutrition**

**P103**

**Bile acid regulation of amino acid metabolism is associated with obesity-induced bone loss in rats**

*Bhargavi Gajendra Rao*[1], *Gunter Almer*[1], *Hans-Jürgen Gruber*[1], *Markus Herrmann*[1], *Giovanny Rodriguez-Blanco*[1], *Nerea Alonso*[1]
[1]*Medical University of Graz, Clinical Institute for Medical and Chemical Laboratory Diagnostics, Graz, Austria*

**Abstract Text**

High fat diet (HFD)-induced obesity in rats was associated with bone loss and dysregulation of bile acids (BAs). BAs can promote bone formation by activating RUNX2 and ALP via Farnesoid X receptor (FXR) in osteoblasts. We aim to evaluate further metabolic changes associated with bone loss in this model. Four-month-old female Sprague Dawley rats received HFD or chow diet for 10 months (n=23/group, ethics GZ: 66.010/0070-V/3b/2018). Bone quality was assessed by microCT scan (n=10/group) and controlled by weight. Metabolic changes were measured in serum using an omics-scale LC-MS method, and validated using quantitative methods. MC3T3 osteoblast-like cell line was treated with 10µM Guggulsterone (FXR inhibitor) or GW4064 (FXR activator) up to 24h for metabolite analysis. Student t-test and Spearman correlation analysis was performed in GraphPad Prism v10. Metabolomics identified changes in 51 metabolites in HFD animals compared to controls, including 20 amino acid-related metabolites ($FC_{range}$=0.34-1.88, $p_{range}$=$2.3\times10^{-13}$-0.003). Quantitative analysis (µM, HFD vs controls) validated the alterations in proline (304.8±84.99 vs 192.3±55.41, p=$4.0\times10^{-6}$), threonine (493.5±161.30 vs 311.7±73.47, p=$1.4\times10^{-5}$), and to a lesser extent in leucine/isoleucine (189.8 [102.3-768.7] vs 109.2 [101.5-134.9], p=0.008), methionine (62.37±16.34 vs 52.66±11.04, p=0.02), valine (260.2±69.56 vs 213.9±45.12, p=0.01) and citrulline (71.64±22.09 vs 87.97±20.91, p= 0.01). Proline and threonine levels negatively correlated with bone parameters (r=-0.44--0.68, p=$4.5\times10^{-4}$-0.04) and primary BAs (r=-0.48, p=0.03), especially with β-muricholic acid (r=-0.72, p=$3.6\times10^{-4}$). Citrulline showed a modest correlation with bone parameters (r=0.53, p=0.01) and glycine-conjugated primary BAs (r=0.63, p=0.003). FXR inhibition in osteoblasts reduced the levels of several amino acids (FC=0.76-0.89, p=$2.1\times10^{-5}$-0.01), whilst FXR activation increased them (FC=1.13-1.31, p=$2.3\times10^{-4}$-0.04), suggesting that BAs could regulate amino acid metabolism in osteoblasts. In conclusion, obesity-induced alterations in BA metabolism could lead to bone loss via FXR-induced amino acid dysregulation. Thus, dietary modulation of amino acids and/or bile acids could have a potential to prevent bone loss.

**P104**

### Secretome of bone marrow adipose tissue in osteoarthritis: Ex vivo insights from patient-derived bone marrow adipocytes

*Drenka Trivanovic*[1], *Marko Vujačić*[2,3], *Slavko Mojsilović*[1], *Milica Karahasan*[1], *Tamara Kukolj*[1], *Nikola Bogosavljević*[2,3], *Srđa Jankovć*[4], *Aleksandra Jauković*[1]
[1]*Institute for Medical Research University of Belgrade, Group for Hematology and Stem Cells, Belgrade, Serbia*
[2]*Institute for Orthopaedics Banjica, General orthopaedics department, Belgrade, Serbia*
[3]*University of Belgrade, Faculty of Medicine, Belgrade, Serbia*
[4]*University Children's Hospital, Department of Hematology and Oncology, Belgrade, Serbia*

**Abstract Text**

Osteoarthritis (OA) is one of the most common musculoskeletal diseases and might be associated with ageing, obesity and chronic metabolic inflammation (metaflammation). Bone marrow adipose tissue (BMAT) is a distinct adipose tissue depot, anatomically separate from visceral and subcutaneous fat. BMAT is considered an important regulator of bone remodelling and regeneration. However, there is a lack of studies of human bone marrow adipocytes (BMAds) to conclude on their roles in OA disease. To evaluate the inflammatory response, we analysed the secretome of BMAT from hip OA patients following exposure to bacterial endotoxin LPS and metabolic inhibitors.

Bone marrow stromal cells (BMSCs) and BMAds were isolated from the femoral metaphyseal spongiosa of OA patients undergoing hip replacement surgery (n=7, Ethical approval I-97/14.3) and cultured under 3% oxygen conditions. BMAds were exposed to LPS and metabolic inhibitors etomoxir (ETO, a CPT1 inhibitor impairing fatty acid oxidation) and FK866 (a NAD+ biosynthesis inhibitor). BMSCs were induced to undergo 14-day adipogenic differentiation before inhibitor exposure. Conditioned media were collected after 24 h, and the secretome was analysed using a multiplex flow cytometry assay. Statistical significance was determined by non-parametric tests.

Within adipokines, we found Adiponectin, Leptin and Adipsin were the most abundantly secreted by BMAT per se, and a similar pattern appeared in differentiated BMSCs. In addition, IL-1β, MCP-1, IL-6, IL-8, and IL-18 were found to be highly secreted by BMAds ex vivo as well as adipogenic BMSCs. LPS stimulated secretion of adipokines by BMAds, notably Adipsin, Leptin, IFN-α2 and IL-6. Conversely, ETO and FK866 significantly reduced adipokine secretion by BMAds.

These results indicate active inflammatory response in BMAds and BMSCs obtained from OA patients, identifying BMAds as important LPS-sensing compartments within the bone marrow. These findings could enhance understanding of BMAT's contribution to the inflammatory and metabolic landscape of OA.

**P105**

**Bone marrow adiposity is diminished in experimental colitis**

Eman Hagag[1], Maria G. Ledesma-Colunga[1], Caroline Schütz[1], Martina Rauner[1]
[1]Technische Universität Dresden, Department of Medicine III and Center for Healthy Aging, Dresden, Germany

**Abstract Text**

**Background:** Chronic inflammatory conditions, including inflammatory bowel disease (IBD), are associated with systemic bone loss and dysregulated bone remodeling. Our previous work using rodent models of arthritis and spondyloarthritis showed that chronic inflammation reduces bone mass, elevates bone marrow neutrophil, and decreases bone marrow adipose tissue (BMAT).

**Purpose:** Here, we aim to investigate whether BMAT is also affected in IBD , another chronic inflammatory disorder associated with bone loss, and explore how inflammation reshapes the metabolic landscape of the bone marrow.

**Methods:** Colitis was induced in 12-weeks-old C57BL/6 mice using 2.5% dextran sulfate sodium (DSS). Disease severity was scored daily through body-weight changes and a disease activity index (bleeding and stool consistency). At peak of inflammation, colon length, spleen weight, bones and bone marrow were collected for µCT, histology, qPCR and FACS analyses.

**Results:** DSS-treated mice developed pronounced colitis and showed reduced trabecular bone volume in the femur and L4-vertebra [31.3%, 14.8%; $p<0.05$]. Notably, despite the traditionally reported inverse correlation between BMAT and bone mass, DSS- treated mice showed marked reductions in the number and size of bone marrow adipocytes in both femur [5.6-, 3.4-fold; $p<0.05$] and tibia [2-, 2.6-fold; $p<0.05$], without changes in the adipocyte precursor abundance. Consistently, bone marrow expression of the adipogenic markers *Pparγ, Adipoq*, as well as the osteoblast markers *Bgalp and Runx2* was significantly downregulated [50%, 55%, 86%, 54%; $p<0.05$] in DSS-treated mice. Moreover, DSS-treated mice exhibited an increase proportion of immature neutrophils ($CD11b^+$, $GR1^+$, $CD117^-$, $CXCR2^+$, $CXCR4^{low}$, $CD101^-$) in the bone marrow [1.7-fold; $p<0.05$]. This shift in neutrophil subsets was accombined by an enhanced energetic metabolic phenotype, reflected by a 24.5% increase in mitochondrial respiration [$p<0.05$].

**Conclusion**: Together, these results indicate that systemic inflammation reduces BMAT in a mouse model of IBD and remodels the metabolic landscape of the bone marrow microenvironment.

**P106**

**De novo lipogenesis regulates skeletal stem/progenitor cell function**

Annabel Müller[1], Ingrid Stockmans[1], Karen Moermans[1], Geert Carmeliet[1], Steve Stegen[1]
[1]KU Leuven, Department of Chronic Diseases and Metabolism, Leuven, Belgium

**Abstract Text**

**Background/Introduction**: Metabolic diseases are often associated with skeletal cell dysfunction and bone loss. During obesity, the number of marrow adipocytes is increased, which likely occurs at the expense of bone-forming osteoblasts as both cell types arise from a common skeletal stem/progenitor cell (SSPC). However, whether disturbed SSPC lineage allocation and function is caused by metabolic rewiring remains unclear.

**Purpose and methodology:** Our single cell RNA-sequencing analysis on bones from high-fat diet-fed mice revealed that SSPCs express fatty acid synthase (FASN), a key enzyme in *de novo* lipogenesis, which is upregulated during their adipogenic differentiation. To define the role of FASN, we treated primary mouse-derived SSPCs with the small-molecule inhibitor TVB-3364 (TVB).

**Results**: TVB started to impair SSPC growth only after 5 days of culture (-18%, p=0.0785). During earlier timepoints, imaging and lipidomics analysis revealed that TVB reduced lipid droplet abundance (-43%, p<0.01) and intracellular triglyceride levels (-47%, p<0.05), suggesting SSPCs mobilize internal lipid stores to sustain proliferation. Consistently, blocking triglyceride breakdown using lysosomal acid lipase inhibitor Lalistat further suppressed growth of TVB-treated SSPCs relative to vehicle control (-27.2%, p<0.05). Released lipids were likely not used for energy production, as treatment with fatty acid oxidation inhibitor Etomoxir did not differentially affect the growth of TVB-treated SSPCs. Instead, reduced glycerophospholipid levels (phosphatidylcholine -27%, p<0.05; phosphatidylethanolamine -24.6%, p<0.01) suggest that *de novo* lipogenesis is rather necessary for membrane assembly during expansion.

During adipogenic differentiation, TVB markedly reduced accumulation of large lipid droplets without altering adipogenic gene expression, indicating a storage defect rather than impaired lineage allocation. Unexpectedly, blocking FASN in SSPCs impaired osteogenic differentiation, as evidenced by reduced collagen (Sirius Red staining) and mineral deposition (Von Kossa staining), although the underlying mechanism requires further study.

**Conclusion**: Together, our data indicate that FASN-mediated *de novo* lipogenesis regulates SSPC growth and differentiation, but likely through distinct mechanisms.

**P107**

**Molecular changes in zebrafish skeletal development upon hyperglycemia**

*Angelina Daube[1], Tessa Jacqueline Krappa[1], Niklas Hentschel[1], Ulrike Anne Friedrich[2], Nikolay Ninov[3], Franziska Knopf[1]*
*[1]TU Dresden,*
*CRTD - Center for Regenerative Therapies TU Dresden- Center for Healthy Aging- Faculty of Medicine- TU Dresden, Dresden, Germany*
*[2]TU Dresden, DRESDEN-*
*concept Genome Center- DFG NGS Competence Center- c/o Center for Molecular and Cellular Bioengin eering CMCB, Dresden, Germany*

[3]TU Dresden,
*CRTD - Center for Regenerative Therapies TU Dresden- Paul Langerhans Institute Dresden of the Helm holtz Center Munich at the University Hospital Carl Gustav Carus of TU Dresden- German Center for Di abetes Research DZD e.V., Dresden, Germany*

**Abstract Text**

Diabetes mellitus constitutes a growing global health concern due to increased life expectancy and lifestyle changes. The disease is characterized by high blood glucose levels (hyperglycemia) and has been linked to increased bone fragility, fracture risk, and reduced bone density - key features of osteoporosis. Understanding the causative mechanisms of diabetes-induced osteoporosis, including investigations of altered transcriptomics and disrupted cell homeostasis of bone forming osteoblasts, is crucial for developing new therapies.

Here, we examined osteoblasts in the opercle, an early-developing cranial bone, in hyperglycemic zebrafish larvae. High glucose levels were previously induced through larval glucose incubation and β-cell ablation, and here additionally through the usage of *insulin* mutant zebrafish. *Insulin* mutant zebrafish showed a reduced number of osteoblasts (*ins*$^{+/+}$: 38+/-1 osteoblasts, *ins*$^{+/-}$: 41+/-8 osteoblasts, *ins*$^{-/-}$: 29 +/- 4 osteoblasts, p=0.8644 *ins*$^{+/+}$ vs. *ins*$^{+/-}$, p=0.0014 *ins*$^{+/+}$ vs. *ins*$^{-/-}$, p=0.0395 *ins*$^{+/-}$ vs. *ins*$^{-/-}$) and reduced opercle size (*ins*$^{+/+}$: 5058 +/- 1059 µm$^2$, *ins*$^{+/-}$: 5423 +/- 1216 µm$^2$, *ins*$^{-/-}$: 3829 +/- 756 µm$^2$, p=0.8656 *ins*$^{+/+}$ vs. *ins*$^{+/-}$, p=0.2159 *ins*$^{+/+}$ vs. *ins*$^{-/-,}$ p=0.0337 *ins*$^{+/-}$ vs. *ins*$^{-/-}$), suggesting delayed bone development, similar to hyperglycemia after glucose incubation and β-cell ablation.

Transcriptome and differential gene expression analysis of euglycemic and hyperglycemic osteoblasts suggested alterations in genes involved in mitochondrial homeostasis (e.g., *hadhaa*, L2FC= 8.84, $p_{adj}$= 4.56E-09), the heat shock response (e.g., *hspa9*, L2FC= -1.4, $p_{adj}$= 1.43E-03) and DNA repair mechanisms (e.g., *brip1*, L2FC= -4.8, $p_{adj}$= 4.87E-02). Furthermore, alterations in Wnt-signaling activity were detected in hyperglycemic larvae (e.g., *pak4*, L2FC= -8,46, $p_{adj}$= 2.92E-04). With the help of transgenic reporter zebrafish, we are currently testing changes in Wnt-signaling and mitochondrial function. Furthermore, stainings for advanced glycation end products (AGEs), hallmarks of diabetes, are performed at different developmental stages.

(L2FC: log2 fold change)

**P109**

**Simulated hypoxia maintains physiological energy metabolism in osteogenic differentiated BMSCs: toward more realistic in vitro models**

*Yudong Zhao[1], Marijke Koedam[1], Johannes P.T.M. van Leeuwen[1], Bram C. J. van der Eerden[1]*
*[1]Erasmus University Medical Center, Internal Medicine, Rotterdam, Netherlands*

**Abstract Text**

**Background:** Physiological oxygen levels in the bone marrow are much lower than the ambient level used in cell culture. Studying BMSC-derived osteoblasts and their energy metabolism requires *in vitro* simulation of low oxygen levels to which these cells are naturally exposed. Physiological hypoxia can be created with special equipment such as hypoxia chambers, but hypoxia-mimicking compounds may offer a simple, stable, and cost-effective alternative.

**Purpose:** This study compared responses of BMSCs to 3% oxygen versus three hypoxia-mimicking compounds: dimethyloxalylglycine (DMOG), deferoxamine (DFO), and cobalt chloride (CoCl2).

**Methods:** Compound concentrations from 0.16 µM to 500 µM were compared to 20% ambient oxygen and 3% physiological oxygen concentration. BMSC viability and differentiation were evaluated by measuring total protein, alkaline phosphatase (ALP) activity, and mineralization. qPCR analysis assessed known hypoxia and osteogenic markers, while Flash-Seq studies yielded differentially expressed genes (DEG)-mediated identification of hypoxia-related cellular pathways. Seahorse mitochondrial stress tests were conducted to assess the metabolic effects.

**Results:** All compounds and 3% oxygen strongly inhibit osteogenic differentiation versus controls as shown by ALP activity and calcium assay; CoCl2 and DMOG at 20 µM match the physiological hypoxia effect most closely (Fig.1). Seahorse tests showed that compounds induce metabolic reprogramming by inhibiting mitochondrial respiration and enhancing glycolysis. Dose-response curves show distinct EC50 values between compounds, with DMOG being the most potent (Fig.2). Osteogenically differentiated BMSCs exhibit similar responses to compounds and 3% oxygen, demonstrated by a dose-dependent upregulation of hypoxia markers and downregulation of osteogenic markers compared to 20% oxygen (Fig.3). Ingenuity pathway analysis showed that the DEGs of all compounds strongly activated HIF1α signaling (z-score 2.38) and glycolysis (z-score 3.05) (FDR<0.05).

**Conclusions:** The optimal dose of CoCl2 and DMOG resembles 3% physiological oxygen on osteogenic BMSCs. They can serve as practical alternatives for creating more realistic *in vitro* models and help overcome the limits of physical hypoxia setups.

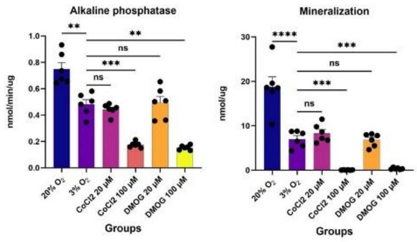

Fig 1. Hypoxia decreases alkaline phosphatase activity and calcium production of osteogenic BMSCs

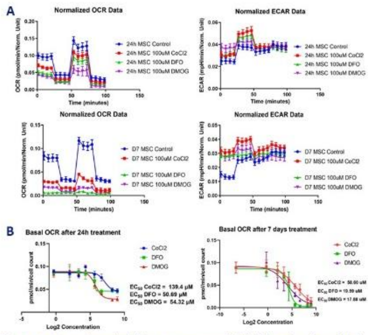

Fig 2. A: Hypoxia-mimicking compounds inhibit mitochondrial respiration and stimulate glycolysis;
B: Dose-response curves were plotted for the hypoxia-mimicking compounds to determine their EC50 values

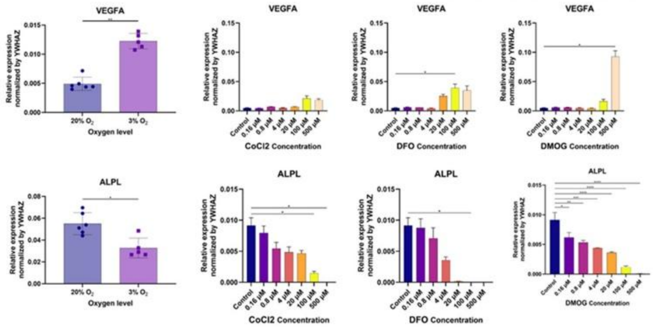

Fig 3. qPCR results suggest a similar response of VEGFA and ALPL between physical hypoxia and hypoxia-mimicking compounds

**P110**

**Correlative imaging of vertebral bone marrow adiposity using spectral computed tomography and undecalcified histology.**

*Johannes Krug[1,2], Fede Melzer[3], Graeme Campbell[4], Isabel Molwitz[3], Björn Busse[1,2]*
*[1]University Medical Center Hamburg-Eppendorf, Department of Osteology and Biomechanics, Hamburg, Germany*
*[2]University Medical Center Hamburg-Eppendorf,*
*Interdisciplinary Competence Center for Interface Research, Hamburg, Germany*
*[3]University Medical Center Hamburg-Eppendorf,*
*Diagnostic and Interventional Radiology and Nuclear Medicine, Hamburg, Germany*
*[4]Philips GmbH Market DACH, Clinical Scientist, Hamburg, Germany*

**Abstract Text**

**Background:** Adipose tissue inside the skeleton plays a crucial role for bone quality, with emerging research showing changes in marrow fat content connected to aging, bone loss and various pathologies. Spectral computed tomography (CT), which has been shown to allow for reliable assessment of body-fat distribution, also presents an opportunity to assess marrow fat-content without the necessity of biopsies but has not been validated for bone, yet.
**Purpose:** Validation of spectral CT based quantification of fat content in bone marrow of human vertebrae as a potential clinical diagnostic tool.

**Methods:** Human vertebrae were extracted from human individuals post mortem. Vertebrae underwent magnetic resonance imaging (MRI), followed by spectral CT imaging. Fat content inside of the vertebrae was derived from both mDIXONQuant and three-material decomposition from spectral imaging (both: (Philips, the Netherlands). Subsequently, vertebrae were fixed, resin-embedded, cut and ground into sections, stained with Toluidine-Blue and imaged via brightfield microscopy. Area fat-fraction was measured in histological sections by digital segmentation of adipocytes (Figure 1A). Further study groups with manifested bone marrow adiposity phenotypes were investigated (Djungarian Hamsters).
**Results:** Histological and MRI analysis showed a large variance in marrow fat-content between samples, highlighting the relevance of individual assessments of marrow adiposity in patient care. Linear regression analysis revealed a coefficient of determination of $R^2$=0.87 between MRI and spectral CT derived quantifications (Figure 1B).
**Conclusion:** Spectral CT was identified as a promising tool for marrow fat quantification, providing an additional tool for the assessment of bone health.

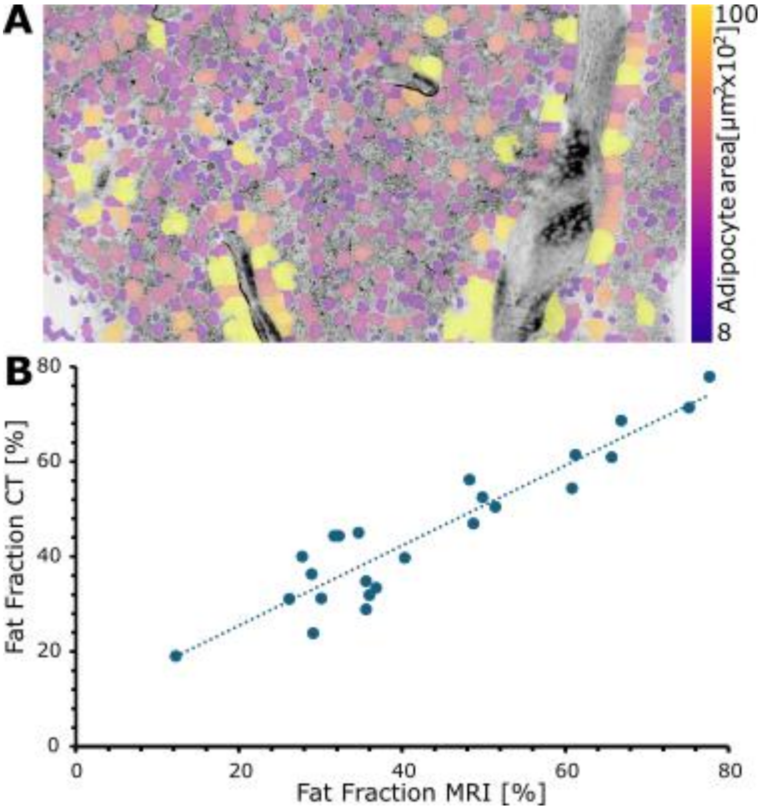

*Figure 1: A) Fat content in bone marrow assessed through adipocyte segmentation in histological sections. B) Correlation between fat fraction measurements in bone marrow from MRI and spectral CT.*

**P111**

**Type 2 diabetes alters Extracellular Vesicles from Bone Marrow Mesenchymal Stem Cells and Plasma**

*William Ndjidda Bakari*[1], *Ji Ding*[2], *Sarah Razafindrakoto*[3], *Kelly Aubertin*[4], *Amanda Brun*[5], *Graciela Pavon-Djavid*[6], *Fani Anagnostou*[1]
*[1]CNRS UMR7052- INSERM U1271- ENVA- Université Paris Cité,*
*Biology- Bioengineering and Osteoarticular Bioimaging, Paris, France*
*[2]CNRS UMR7052- INSERM U1271- ENVA- Université Paris Cité,*
*Biology- Bioengineering and Osteoarticular Bioimaging-, Paris, France*
*[3]CNRS umr 8175- INSERM U1334,*
*Nanomedicine Extracellular Biology- Integratome and health innovations, Paris, France*
*[4]CNRS umr8175 INSERM U1334,*
*Nanomedicine Extracellular Biology- Integratome and health innovations-, Paris, France*
*[5]CNRS umr8175 INSERM U1334,*
*Nanomedicine Extracellular Biology- Integratome and health innovations, Paris, France*
*[6]INSERM U1148 Université Sorbonne Paris Nord,*
*Laboratory for Vascular Translational Science Cardiovascular Bioengineering, Villetaneuse, France*

**Abstract Text**

**Introduction:** Type 2 Diabetes Mellitus (T2DM) is a metabolic disease associated with bone fragility, and impaired bone repair. T2DM-patients with bone complications are increasing while treatment remains challenging. T2DM microenvironment affects Bone Marrow (BM) and BM-mesenchymal stromal cells (MSC), key players of bone homeostasis and repair but its impact on MSC extracellular vesicles (MSC-EVs), crucial mediators of the paracrine effects and regenerative potential of MSC remains unclear.
The present study assessed the impact of T2DM microenvironment on BM and MSC-EVs.

**Methods:** BM, MSC and plasma were harvested from 24-week-old Zucker Diabetic Fatty (ZDF) and Zucker Lean (ZL) rats (controls). EVs from BM and MSC were isolated by filtration and MSC-EVs were purified from conditioned media (CM), using tangential flow filtration. EVs concentration and size were analyzed by nanoparticle tracking analysis. Their effects were studied on proliferation and migration. (Project:APAFIS #49625-2024030117359211).

**Results:** BM-EVs exhibited no size differences (151.4±12.5 vs 140.4±13.9 nm) and were less concentrated in ZDF (3.9-fold, p<0.01) than in ZL ($5.85x10^{10}$ ± $2.2x10^{10}$ vs $2,3x10^{11}$±$5,5x10^{10}$ particles/ml, respectively). ZDF-MSC-EVs were larger (p<0.01) than ZL-MSC-EVs (163,3±0,5 vs 145,9±2,9 nm respectively), with similar concentration. EVs from both CM enhanced diabetic and healthy MSC proliferation but ZDF-MSC-EVs exerted weaker effects on cell number increase than ZL-MSC-EVs (1.8- fold vs 2.2-fold, P<0.0001 respectively). The trend observed for both ZDF and ZL cultured MSC was similar. ZDF plasma-enriched EVs fraction (ZDF-PEF) exhibited no size differences (91.7 ± 6.3 vs 88.5 ± 6.2 nm) but contained 3.2-fold more EVs than ZL-PEF ($7.4x10^{11}$±$1.6x10^{11}$ Vs $2.4x10^{12}$ ± $9.1x10^{11}$particles/ml, p<0.01). Moreover, ZDF-PEF reduced migration (21.9%; p<0.0001) and inhibited both ZDF- and ZL-MSC proliferation. In contrast, ZL-PEF increased proliferation rate (by 1. 6 and 3.8-fold, p<0.001 for ZDF and ZL-MSC respectively).

**Conclusion** T2DM alters EVs profiles and exerts negative effects, which may contribute to impaired MSC function and compromised bone repair.

**P112**

### Bone marrow-derived mesenchymal stem cells from osteoporotic women exhibit deregulated energy metabolism

*Souad Daamouch*[1], *Emilie Grupe*[1], *Shakespeare Jeromdesella*[1], *Maria Bruun Jakobsson*[1], *Alexander Rauch*[1], *Moustapha Kassem*[1]
[1]*SDU-KMEB,*
*Molecular Endocrinology and Stem Cell Research Unit- Department of Endocrinology- Odense University Hospital, Odense, Denmark*

**Abstract Text**

**Background:** Intrinsic cellular defects in patients with osteoporosis remain poorly characterised. As energy metabolism regulates osteoblastic differentiation and function, metabolic alterations may contribute to osteoporotic pathophysiology.

**Purpose:** To investigate whether mesenchymal stem cells (MSCs) from osteoporotic women show defects in energy metabolism related to their intrinsic gene activity profiles.

**Methods:** Bone marrow-derived MSCs were isolated and cultured from young healthy (n = 10, age: 20-26), elderly healthy (n = 6, age: 66-75), and osteoporotic (n = 11, age: 56-87) women. Metabolic function was assessed using SeahorseÒ respirometry and ATP production quantification assays. Single-cell RNA-sequencing was performed on MSCs from young (n= 3) and osteoporotic (n = 5) donors with analyses of cell composition, differential gene expression, and metabolic activity using single-cell *Flux Estimation Analysis* (scFEA).

**Results:** We observed globally reduced total and glycolytic ATP levels in osteoporotic compared to young (-29%, p-value = 0.0209 and -45%, p-value = 0.0055) and elderly females (-21%, p-value = 0.1872 and -39%, p-value = 0.0689). Single-cell RNA-seq revealed metabolically divergent states ranging from glycolytic to nutrient-restricted phenotypes, supported by scFEA-predicted shifts in glycolytic and mitochondrial activity and the presence of metabolically divergent states, spanning glycolytic to nutrient-restricted transcriptional phenotypes. Although total ATP production was reduced, osteoporotic MSCs displayed a more divergent glycolytic to mitochondrial ATP production ratio. Two metabolic subgroups emerged: (1) an OXPHOS-low phenotype (n = 6; 36.4% ± 11.1% respiratory ATP production) comparable to young  (32.5% ± 6.5%) and elderly (34.7% ± 14.2%) MSCs; and (2) an OXPHOS-high phenotype (n = 5; 64.4% ± 6.8%), characterized by elevated oxygen consumption yet reduced ATP-linked respiration, suggesting mitochondrial inefficiency and increased oxidative stress response.

**Conclusion:** MSCs from osteoporotic women exhibit unrecognized metabolic dysfunction not attributable to aging *per se*. Targeting cellular energy metabolism may represent a useful therapeutic approach for osteoporosis in females.

**ND-P010**

### Peripheral osteoclast progenitor subsets and their migration potential in patients with type 1 diabetes mellitus

*Ozana Jakšić*[1], *Maša Filipović*[1], *Marta Radošević*[1], *Sara Aničić*[1], *Ivo Krešić*[2], *Alan Šućur*[1], *Nataša Kovačić*[3], *Marina Ikić Matijašević*[4], *Ivana Kraljević*[5], *Valerija Bralić Lang*[6], *Danka Grčević*[1]
[1]*University of Zagreb School of Medicine, Department of Physiology and Immunology, Zagreb, Croatia*
[2]*University of Mostar School of Medicine, Department of Physiology, Mostar, Bosnia - Herzegovina*
[3]*University of Zagreb School of Medicine, Department of Anatomy, Zagreb, Croatia*
[4]*University Hospital "Sveti Duh",*
*Department of Clinical Immunology- Rheumatology and Pulmonology, Zagreb, Croatia*
[5]*University Hospital Centre Zagreb, Department of Endocrinology, Zagreb, Croatia*
[6]*University of Zagreb School of Medicine, Department of Family Medicine, Zagreb, Croatia*

**Abstract Text**

**Background:** Type 1 diabetes (T1D), an immune-mediated inflammatory disease, is associated with increased fracture risk despite preserved or only modestly reduced bone mineral density, suggesting altered bone remodeling rather than bone mass alone. Chronic low-grade inflammation may promote osteoclastogenesis by activating circulating myeloid progenitors, but functional evidence in humans remains limited.

**Purpose:** We aimed to characterize the frequency, receptor expression profile, and migratory behavior of circulating osteoclast progenitor subsets in patients with T1D.

**Methods:** Human peripheral blood mononuclear cells were analyzed from healthy controls (n=39) and patients with T1D (n=32) after ethical approval and informed consent. Osteoclast progenitors were identified by flow cytometry as $CD45^{+}CD15^{-}CD3^{-}CD19^{-}CD56^{-}CD11b^{+}CD16^{-}CD14^{+}$ cells, belonging to the classical monocyte subset. Progenitor potency was confirmed by trilineage differentiation of sorted cells into osteoclasts, macrophages, and dendritic lineages, under lineage-specific conditions. Sorted progenitors were assessed in *Transwell* migration assays toward chemotactic ligands CCL2 or LPS, with pharmacological blockade of CCR2 or TLR4.

**Results:** Patients with T1D had a higher proportion of classical monocytes compared with controls (27.7% [IQR 22.6–35.8] vs 18.4% [IQR 12.3–26.2]; $p<0.05$). More than 80% of osteoclast progenitors expressed CCR2 and TLR4. In T1D, progenitors showed increased expression of CCR2, TLR4, and the M-CSF receptor CD115, indicating enhanced sensitivity to inflammatory and osteoclastogenic cues. CCL2 robustly induced progenitor migration, which was markedly reduced by CCR2 blockade, whereas LPS induced weaker migration with limited inhibition by TLR4 blockade.

**Conclusions:** T1D is associated with the expansion and functional priming of circulating osteoclast progenitors, characterized by enhanced CCR2-dependent migratory capacity and osteoclastogenic potential. These findings support a mechanistic link between systemic inflammation in T1D and skeletal fragility, specifically through the CCR2–CCL2 axis. Therefore, CCR2 may be further explored as a therapeutic target to limit diabetes-associated bone resorption. Funding was provided by the EU

through the National Recovery and Resilience Plan, grant agreement NPOO.C3.2.R3-I1.04.0093(CHIPART); HRZZ-IP-2022-10-2285; HRZZ-DOK-2025-02-2318.

**Poster Sessions: Genetics and epigenetics**

**P114**

**The role of the CYP1A1 gene in long-term glucocorticoid-related adverse effects.**

*Helena Florez Henrich[1], Eva Gonzalez[2], Katherine Cajiao[3], Josep Lluis Carrasco[4], José Hernández-Rodríguez[5], Sergio Prieto-González[5], Maria C. Cid[5], Ana Monegal[6], Núria Guañabens[7], Pilar Peris[7]*
*[1]Hospital Clinic- University of Barcelona., Metabolic Bone Diseases Unit- Department of Rheumatology., Barcelona, Spain*
*[2]Hospital Clinic- University of Barcelona, Department of Immunology, Barcelona, Spain*
*[3]Hospital Clinic- University of Barcelona., Metabolic Bone Diseases Unit. Department of Rheumatology, Barcelona, Spain*
*[4]University of Barcelona- Barcelona, Biostatistics- Department of Basic Clinical Practice, Barcelona, Spain*
*[5]Hospital Clínic- University of Barcelona- Institut d'Investigacions Biomèdiques August Pi i Sunyer IDIB APS- Barcelona, Vasculitis Research Unit- Department of Autoimmune Diseases, Barcelona, Spain*
*[6]Hospital Clinic- University of Barcelona, Metabolic Bone Diseases Unit. Department of Rheumatology, Barcelona, Spain*
*[7]Hospital Clinic- University of Barcelona., Metabolic Bone Diseases Unit. Department of Rheumatology., Barcelona, Spain*

**Abstract Text**

**Background**: Long-term glucocorticoid (GC) use can produce adverse effects (AE) including obesity, diabetes, osteoporosis and fractures. The variability in AE severity suggests a genetic influence. *CYP1A1*, a cytochrome P450 family enzyme involved in GC metabolism may play a role.

**Purpose:** This study aimed to genotype *CYP1A1* variants in patients on chronic GC treatment and analyze their relationship with long-term AE.

**Methods:** Seventy patients (56±16.6 years; 71.4% female; 47.1% postmenopausal) receiving chronic GC treatment (≥5 mg/day/>3 months) were included. Clinical data were collected at baseline and at 5 years of follow-up, including OP risk factors, previous and incidental fractures, GC-dose and duration, disease activity, anthropometric data, bone metabolism parameters, bone mineral density (BMD) and trabecular bone score (TBS) (by DXA), and vertebral fractures (X-ray). *CYP1A1* gene sequencing was performed by NGS using Amplicon Deep Sequencing on an Ion Torrent platform as described in Mensa-Vilaró et al.[1]

**Results:** Four *CYP1A1* polymorphisms were identified: rs41279188, rs1048943, rs1799814 and rs4646422. Nine patients had rs1048943, nine rs1799814, one rs4646422 and one rs12086634 polymorphisms, respectively. Patients with rs1799814 polymorphism (13%) had higher BMI

(29.3 *vs.* 25.5; p=0.019) and greater BMI change over 5 years (ΔBMI: 1.7 *vs.* -0.32;p=0.03; adjusted for cumulative GC dose). Additionally, they presented lower bone turnover markers (NTx: 21 vs. 40 nMol/mMol, p=0.003, CTx: 0.16 vs. 0.29 ng/mL, p=0.068, OC: 16.6 vs. 19.3 ng/mL, p=0.059). No associations were found between *CYP1A1* polymorphisms and the presence of osteoporosis and/or fractures, BMD or TBS values.

**Conclusion:** The *CYP1A1* rs1799814 polymorphism is associated with more severe GC-related AE, suggesting reduced GC metabolism.

**References**

[1] Mensa-Vilaró A, et al. Unexpected relevant role of gene mosaicism in patients with primary immunodeficiency diseases. J Allergy Clin Immunol. 2019;143:359-368. doi: 10.1016/j.jaci.2018.09.009.

**P115**

**Investigating the role of Variants of Uncertain Significance (VUS) on bone fragility and low bone mineral density (BMD): a multicenter study**

*Silvia Carrara[1], Agnese Allora[1], Silvia Vai[1], Alessandra Mihalich[2], Anna Di Blasio[2], Emanuela Ponti[2], Gregorio Guabello[3], Vito Guarnieri[4], Stella Pigni[5,6], Alessandro Fanti[5,6], Andrea Lania[5,6], Gherardo Mazziotti[5,6], Sabrina Corbetta[1,7]*
*[1]IRCCS Istituto Auxologico Italiano, Bone Metabolism Diseases and Diabetes Unit, Milan, Italy*
*[2]IRCCS Istituto Auxologico Italiano, Medical Genetic Laboratory, Cusano Milanino, Italy*
*[3]IRCCS Ospedale Galeazzi Sant'Ambrogio, Ambulatorio Metabolismo Osseo, Milan, Italy*
*[4]Fondazione IRCCS Casa Sollievo della Sofferenza, Division of Medical Genetics, San Giovanni Rotondo FG, Italy*
*[5]IRCCS Humanitas Research Hospital, Endocrinology- Diabetology and Medical Andrology Unit, Rozzano Milan, Italy*
*[6]Humanitas University of Milan, Department of Biomedical Sciences, Rozzano Milan, Italy*
*[7]University of Milan, Department of Biomedical- Surgical and Dental Sciences, Milan, Italy*

**Abstract Text**

Genetic predisposition is a key factor in determining fragility fractures' risk. The number of implicated genes is rising; however, the clinical relevance of Variants of Uncertain Significance (VUS) remains unclear. We examined the role of VUS in determining bone fragility or low BMD.

We retrospectively evaluated 122 adults (pre- or peri-menopausal women or men < 55 years), undergoing Next Generation Sequencing (NGS) analysis because of multiple fragility fractures and/or low BMD in the absence of secondary osteoporosis or a clear phenotype of monogenic bone disease. NGS analysis covered an extensive panel for osteogenesis imperfecta, low BMD and collagenopathies related genes, including *COL1A1, COL1A2, BMP1, WNT1, LRP5*. Genetic variants were classified according to American College of Medical Genetics and Genomics.

Gene variants were detected in 61/122 (50%) patients. Pathogenic or likely pathogenic mutations in *COL1A1, COL1A2, WNT1* or *ALPL* were found in 8 patients (6.6% of the cohort): 3 patients had densitometric osteoporosis, while heterozygous *ALPL* carriers had normal total and bone alkaline phosphatase (ALP, BALP) levels, all experienced at least one fracture. The remaining 53 patients harboured VUS, more frequently in *BMP1* (n=8), *LRP5* (n=8), *NOTCH1* (n=5), *COL5A1* and *WNT1* (n=4) gene; 20.7% harboured multiple VUS. Compared with wild-type patients, those with VUS did not differ in clinical variables (age, sex, age at first fracture, family history of fractures, fracture number and BMD).

In conclusion, in selected patients undergoing NGS analysis, genetic variants are frequent, often multiple and involving recurrent genes; however, pathogenic variants are rare, though not associated with classical phenotypes. Present data underscore the need for broader genetic testing and further investigation of the role of VUS.

**P116**

**Role of gonosomal mosaicism in Osteogenesis imperfecta**

*Eva González Roca*[1]*, Rosa Bou*[2]*, Alba Roset*[1]*, Mª Mar López*[1]*, Anna Mensa-Vilaró*[3]*, Juan Ignacio Arostegui*[3]*, Pilar Peris*[4]

[1]*Hospital Clinic De Barcelona, Molecular Biology CORE Laboratory, Barcelona, Spain*
[2]*Hospital Sant Joan de Déu, Pediatric Rheumatology, Esplugues, Spain*
[3]*Hospital Clinic de Barcelona, Immunology Department, Barcelona, Spain*
[4]*Hospital Clinic de Barcelona, Rheumatology Department, Barcelona, Spain*

**Abstract Text**

**Introduction:** Osteogenesis imperfecta (OI) is a genetic disease that affects collagen synthesis and increases bone fragility. Almost 90% of patients have pathogenic variants in genes codifying for type 1 collagen (*COL1A1* and *COL1A2*). Genetic mosaicism has been described in families with recurrent perinatal OI, that present with heterogenous phenotypes, and in some patients with overlapping OI/EDS (1).

**Purpose:** We wanted to study the presence of low allele pathogenic variants in parents of OI affected patients.

**Methods:** 18 families were included in the study as they fulfilled the inclusion criteria: 1) have 1 or more OI affected members with a pathogenic variant in *COL1A1*, *COL1A2* or *IFITM5* genes, 2) parents must not be carriers and, 3) patients had OI of any type except perinatal type 2. DNA was extracted for all family members and the regions were the pathogenic variants were located were amplified and sequenced by Amplicon Deep Sequencing (2) in an MiSeq (Illumina). A minimum coverage of 1000x was obtained in order to detect low allele frequency variants. Data analysis was done using the MiSeqReporter (Illumina) and IGV softwares.

**Results:** We identified a somatic variant in one father of the 18 families. The variant had an AF of 1.85% (425 sequences with the variant, 23000 wild-type), considering him as a probable carrier.

**Conclusions:** this study suggests a relevant role of somatic pathogenic variant in OI. The detection at low frequency in one father of the rest of 18 families can suggest a general role in the disease transmission not restricted to type 2 OI. In conclusion, we recommend to do genetic testing with the ability to detect low allele frequency variants to do a correct genetic counselling.

**References**

1.S. Symoens et al., (2017) Am.J.Med.Genet. Part A, vol. 173:1047–1050.

2.A. Mensa-Vilaró et al. (2019) JACI, vol. 143:359–368.

**P117**

**Teriparatide timing shapes mesenchymal stem cells transcriptomes: lncRNA and mRNA insights**

*Lucija Ana Vrščaj*[1], *Janja Marc*[1,2], *Barbara Ostanek*[1]
[1]*University of Ljubljana- Faculty of Pharmacy, Department of Clinical Biochemistry, Ljubljana, Slovenia*
[2]*University Clinical Centre- Ljubljana, Clinical Institute of Clinical Chemistry and Biochemistry, Ljubljana, Slovenia*

**Abstract Text**

**Background:** Teriparatide (TPTD) exhibits potent osteoanabolic effects; however, the transcriptomic mechanisms underlying its continuous and intermittent treatment effects are yet to be comprehensively elucidated. In this study, we present the **first lncRNA-centered transcriptomic analysis** of human bone marrow-derived mesenchymal stem cells (MSCs) undergoing osteogenic differentiation treated with TPTD using two distinct treatment protocols.

**Purpose:** To define how TPTD timing reprograms MSC transcriptional states and identify lncRNA-mRNA networks underlying the superior osteoanabolic response to intermittent treatment.

**Methods:** Commercially sourced human MSCs were differentiated osteogenically *in vitro* and treated with intermittent or continuous TPTD. RNA sequencing was followed by differential expression, pathway enrichment, and co-expression network analyses.

**Results:** RNA sequencing revealed **7,622 differentially expressed lncRNAs** across both regimens compared with untreated controls. Intermittent TPTD strongly activated **HIF-1, VEGF, and MAPK pathways**, key regulators of angiogenesis, hypoxia response, and osteogenesis. Network analysis identified **AC008622.2, MED17, and RNF213** as the most critical lncRNAs associated with intermittent stimulation. Continuous TPTD engaged a mechanistically distinct network centered on **XLOC_055533, SPG7, and HOOK3**.

**Table 1. Key differentially expressed transcripts under intermittent and continuous TPTD** (Each gene followed by its **p adj**)

| Treatment | Upregulated lncRNAs | Downregulated lncRNAs | Upregulated mRNAs | Downregulated mRNAs |
|---|---|---|---|---|

| | | | | |
|---|---|---|---|---|
| **Intermittent** | VCP (2.13E-43); EMP1 (4.48E-43); OXA1L (5.12E-42); LPP (1.11E-39) | KRTAP4-11 (3.62E-44); DUBR (6.64E-35); MEG3 (5.59E-33); DIABLO (1.01E-32) | DNAJC25-GNG10 (1.99E-46); ZBTB4 (2.09E-41); SLC2A6 (1.38E-37); TMEM189-UBE2V1 (3.15E-33) | PIP4K2B (5.79E-46); GFI1B (1.56E-43); ISY1-RAB43 (5.07E-34); AC010422.5 (3.04E-27) |
| **Continuous** | XLOC_055533 (6.13E-63); SCARB2 (1.38E-43); HNRNPC (2.82E-39); VCP (3.18E-39) | KRTAP4–11 (1.84E−42); XLOC_055164 (8.11E-42); SLC2A3 (7.47E-41); COPG1 (5.15E-39); SMTN (5.18E-36) | SLC2A6 (5.93E-39); ZBTB4 (5.93E-39); MX1 (6.97E-29); BDP1 (5.46E-27); RSAD2 (1.66E−20) | ISY1-RAB43 (4.02E−32); ZDBF2 (2.88E-31); KLKB1 (1.00E-30); RPS10-NUDT3 (1.74E-27); XLOC_055092 (2.22E-26) |

**Conclusion:** Collectively, these findings provide the **first high-resolution transcriptomic map** of MSC responses to clinically relevant TPTD regimens and nominate novel lncRNAs and mRNAs (Table 1) as potential biomarkers or therapeutic targets to optimize osteoanabolic treatment strategies.

**P118**

**Secretome-Induced Transcriptomic Remodeling in Mesenchymal Stem Cells: Insights into Key Regulators Driving Osteosarcopenia**

*Simona Jurkovic Mlakar[1], Žana Rus[2], Barbara Ostanek[3], Ana Lucija Vrscaj[3], Klemen Dolinar[4], Maja Lindic[5], Katarina Valek[5], Tomaž Kocjan[6], Radko Komadina[5], Sergej Pirkmajer[7], Janja Marc[3]*
*[1]University of Ljubljana- Faculty of pharmacy, Department for clinical biochemistry, Ljubljana, Slovenia*
*[2]Faculty bof Pharmacy- University of Ljubljana, Department for Clinical Biocehmistry, Ljubljana, Slovenia*
*[3]Faculty of pharmacy- University of Ljubljana, Department for Clinical Biochemistry, Ljubljana, Slovenia*
*[4]Medical Faculty Ljubljana- University of Ljubljana, Institute of Pathophysiology, Ljubljana, Slovenia*
*[5]General Hospital Celje, Traumatology department, Celje, Slovenia*
*[6]University Clinical Centre Ljubljana,*
*Clinical Department of Endocrinology- Diabetes and Metabolic Diseases, Ljubljana, Slovenia*
*[7]Medical Faculty Ljubljana, Institute of Pathophysiology, Ljubljana, Slovenia*

**Abstract Text**

**Background:** Osteosarcopenia, the coexistence of bone fragility and muscle wasting, is increasingly recognized as a systemic condition driven by pathological microenvironments. The sarcopenic secretome (MedS) profoundly impacts mesenchymal stem cells (MSCs), promoting inflammation, metabolic stress, and impaired osteogenesis, yet its molecular underpinnings remain poorly defined.

**Purpose:** This study aimed to delineate transcriptomic signatures and identify upstream regulators in MSCs exposed to MedS, providing mechanistic insights into osteosarcopenia.

**Methods:** RNA sequencing was performed on MSCs from sarcopenic (MSC_S) and non-sarcopenic (MSC_NS) donors under MedS conditions. Differential expression and pathway enrichment analyses were conducted using Ingenuity Pathway Analysis (IPA). Activation z-score, a predictive metric in IPA, estimates regulator activity based on directional gene expression changes, where positive values indicate activation and negative values indicate inhibition. Statistical significance was assessed via q-values for overlap with significantly differentially expressed gene targets (sigDEGs).

**Results:** MedS exposure profoundly reshaped the MSC transcriptome, revealing five pivotal regulators. PPARGC1A ($z = -0.757$, $p = 2.64\times10^{-9}$; 19 sigDEGs) was strongly inhibited, disrupting lipid metabolism and extracellular matrix integrity, linking energy homeostasis to tissue remodeling. AGT ($z = -0.139$, $q = 1.16\times10^{-9}$) suppression impaired IGF signaling and angiogenesis, compromising repair pathways. MYF6 ($z = +1.432$, $q = 3.15\times10^{-5}$) activation promoted muscle growth and anti-inflammatory effects, reinforcing bone–muscle crosstalk. S100 family members, including S100A9 ($z = 1.081$) and S100A8 ($z = 1.387$), triggered PI3K/AKT and NFκB cascades, accelerating inflammation and ECM degradation. CREB signaling, associated with BMP2 upregulation (fold change 1.121, $q = 4.93\times10^{-2}$), enhanced osteogenesis under stress, positioning CREB as a central transcriptional hub. These pathways converge on metabolic dysfunction, fibrosis,and impaired osteogenic differentiation.

**Conclusion:** PPARGC1A, AGT, MYF6, S100 proteins, and CREB emerge as novel molecular drivers of osteosarcopenia under sarcopenic secretome influence. Their integrative roles in metabolism, inflammation, and osteogenesis highlight actionable targets for biomarker development and precision therapies to counteract bone–muscle deterioration.

**ND-P011**

**Association between candidate genes and FRAX scores in postmenopausal women**

*<u>Jessica Pepe</u>[1], Arianna Viviani[2], Cristiana Cipriani[1], Bianca Maria Ciminelli[2], Carla Jodice[2], Luciano Colangelo[3], Marco Forti[4], Rachele Santori[1], Sara Terreri[5], Salvatore Minisola[1], Sara Latini[6], Marco Occhiuto[1], Patrizia Malaspina[2], Andrea Novelletto[2]*
*[1]Sapienza University, Department of medical and cardiovascular sciences, Rome, Italy*
*[2]University of Rome Tor Vergata, Department of Biology, Rome, Italy*
*[3]Azienda Ospedaliero Universitaria Pisana, Department of Emergency Medicine, Pisa, Italy*
*[4]Sapienza University, Department of Social Sciences and Economics, Rome, Italy*
*[5]Bambino Gesù Children's Hospital- IRCCS,*
*Bone Physiopathology Research Unit- Translational Pediatrics and Clinical Genetics Research Division, Rome, Italy*
*[6]Technical University of Denmark, Department of Biotechnology and Biomedicine, Kongens Lyngby, Denmark*

**Abstract Text**

**Background:** The risk of fracture increases with decreasing BMD, but about two thirds of individuals who suffer a fracture do not have low BMD. Recently it has been shown an improvement in the reclassification of major fractures risk in addition to FRAX adding osteoporosis-related genetic risk scores. Single nucleotide polymorphisms (SNPs) have been associated with BMD and bone fracture risk.

**Purpose:** The aim of this study was to assess the possible association between FRAX scores and specific SNPs in Italian osteopenic and osteoporotic postmenopausal women.

**Methods:** We enrolled 54 postmenopausal women with osteoporosis or osteopenia. FRAX (a free algorithm that provides the 10-year probability of fractures on the basis of classic risk factors) and BMD were obtained in every patient. We included 13 SNPs in the ESR1, OPG, RANK-L, VDR, PTH, CASR, COL1A1, CALCR, ACTN3 and TPRV6 candidate genes. We tested the association of principal component analysis scores based on individual multi-locus genotypes with FRAX.

**Results:** A FRAX_M >=20% was found in 20.3% of patients and a FRAX_H >=3 in 51.8%. Twenty- four patients had a fragility fracture. At the level of single-locus associations, a significant difference (p=0.046) was obtained for FRAX_H and the haplotype ESR1(GC), with mean values of 3.9 vs 7.7 among non-carriers vs carriers.In particular, patients characterized by high FRAX_M and FRAX_H values share haplotype (GT) at PTH and allele G at OPG. On the opposite, patients in the low-risk FRAX score are characterized by COL1A1 allele A, CASR allele T, VDR haplotypes (GA) and (AA)

**Conclusion:** In postmenopausal Italian women an appropriate combination of haplotypes/alleles at PTH and OPG genes is associated with high mean FRAX values. Long term studies would allow to assess if combining multi-locus genetic analyses with FRAX scores could empower physicians to tailor personalized preventive early strategies for osteoporosis.

**ND-P012**

**Epigenetic Aging Indicators are Associated with Bone Mineral Density in a Population-Based Setting**

*<u>Mengyuan Cai</u>[1], Evert van Velsen[1], Lieke Kuiper[1], Jinluan Chen[1], Katerina Trajanoska[1], Joyce van Meurs[1], Fernando Rivadeneira[1], Carola Zillikens[1]*
*[1]Erasmus MC, Internal Medicine, Rotterdam, Netherlands*

**Abstract Text**

**Background:** Epigenetic ageing markers capture biological ageing, but their link to bone mineral density (BMD) and trabecular bone score (TBS) remains unclear.

**Purpose:** To examine associations of epigenetic ageing indicators with BMD and TBS.

**Methods:** We examined four DNA methylation-based aging indicators: three epigenetic clocks (Horvath, GrimAge, and PhenoAge) that focus on relative age acceleration (RAA), and one that measures the pace of aging (DunedinPACE). RAA was defined as residuals from regressing DNAm age on chronological age (z-scored). Outcomes were femoral neck (FN), lumbar spine (LS), total body (TB) BMD, and TBS. Linear regression models were fitted with technical and blood cell composition covariates, with additional adjustment for lifestyle and clinical covariates. False discovery rate (FDR) was controlled across 16 primary tests (Benjamini-Hochberg).

**Results:** In the full sample (n=2,246), higher DunedinPACE was associated with lower TB BMD in the fully adjusted model (β = -0.08, 95% CI -0.12 to -0.04; FDR q < 0.05). Before multiple-testing correction, DunedinPACE also showed an inverse association with FN BMD (β = -0.06, 95% CI -0.11 to -0.01). In the basic model, DNAm clock-based RAAs were associated with TB BMD (Horvath: β = 0.006; GrimAge: β = -0.006), but these associations did not remain significant after FDR correction. No associations were observed for LS BMD or TBS.

**Conclusion:** A faster pace of ageing was associated with lower BMD, most consistently for TB BMD, whereas DNAm clock-based RAA measures showed no robust associations with BMD or TBS after multiple-testing correction.

**Poster Sessions: Inflammation, arthritis and other joint diseases**

**P119**

**Intermittent PTH treatment ameliorates acute sepsis through Serum Amyloid A-dependent mobilisation of bone marrow neutrophils**

*<u>Ruben Augustin</u>[1], Jan Sevecke[1], Anke Baranowsky[1], Johannes Keller[1]*
*[1]Universitätsklinikum Hamburg-Eppendorf, Experimental Trauma Surgery, Hamburg, Germany*

**Abstract Text**

**Background:** Sepsis remains a significant clinical challenge and is the leading cause of death in intensive care units. Intermittent parathyroid hormone (iPTH) treatment, which is used clinically to stimulate bone formation, has been shown to modulate immune pathways and haematopoiesis. While this suggests a potential modulatory role in systemic inflammation, the impact of iPTH on sepsis remains unknown.

**Objectives:** To test an immunomodulatory impact of iPTH on the outcome of experimental polymicrobial sepsis.

**Methods:** WT mice received daily PTH for 14 days prior to the induction of experimental sepsis through caecal ligation and puncture (CLP). All mice were examined at three-hour intervals, and injections were continued until the mice were euthanised. Immune cell populations were analysed via flow cytometry. To identify downstream mediators of iPTH, proteomic and transcriptomic analyses were performed. The validation of mechanistic findings was achieved through mice with antibody-mediated neutrophil depletion and global serum amyloid A 1-4 (SAA) deletion (*Saa*$^{-/-}$). All experiments have been approved by the local authorities.

**Results:** Pretreating wild-type mice with iPTH significantly improved their survival following CLP compared to vehicle controls (survival iPTH 7/12 vs vehicle 2/12; p<0.05). Mechanistically, immunophenotyping revealed that iPTH mobilised neutrophils from the bone marrow into the bloodstream (p<0.01), and neutrophil-depleted mice did not respond to the beneficial impact of iPTH on sepsis outcomes. As an explanation, proteomic and transcriptomic profiling demonstrated iPTH to potently induce the acute-phase protein Saa2 in the liver ($p<0.001$). Conversely, the survival benefit induced by iPTH was absent in $Saa^{-/-}$ mice, which failed to mobilise neutrophils from bone marrow in response to iPTH.

**Conclusions:** These findings imply that iPTH induces hepatic SAA, which mediates neutrophil mobilisation from the bone marrow and protects against sepsis. Therefore, iPTH may be a potential treatment strategy for patients at risk of developing acute sepsis, such as those with polytrauma or immunosuppression.

**P120**

### Wnt1 modulates subchondral bone remodeling and post-traumatic osteoarthritis progression in mice

*Weixin Xie[1], Donat Antonia[1], Jiang Shan[1], Pan Haoyan[1], Augustin Ruben[1], Gerriets Chiara[1], Sevecke Jan[1], Schinke Thorsten[2], Yorgan Timur[2], Baranowsky Anke[1], Keller Johannes[1]*
*[1]Universitätsklinikum Hamburg-Eppendorf, Department of Trauma and Orthopaedic Surgery, Hamburg, Germany*
*[2]Universitätsklinikum Hamburg-Eppendorf, Department of Osteology and Biomechanics, Hamburg, Germany*

**Abstract Text**

**Background:** Abnormal subchondral bone remodeling has been implicated in the progression of cartilage degeneration and osteoarthritis (OA). Wnt1 is a key Wnt ligand in bone metabolism with a potent osteoanabolic effect. However, the role of Wnt1 in subchondral bone remodeling and OA progression is still unclear.

**Purpose:** To investigate the effect of Wnt1 on the progression of post-traumatic knee OA (PTOA) in mice.

**Methods:** Twelve-week-old female C57BL/6 mice were subjected to anterior cruciate ligament transection (ACLT) of the right knee for 4 weeks to induce PTOA. An osteoblast-specific inducible *Wnt1* transgenic ($Col1a1^{tTA}/pTet^{Wnt1}$) mouse model was employed to induce Wnt1 overexpression in osteoblasts during week 0-2 (Early-Tg) or week 2-4 (Late-Tg) after ACLT. Mice carrying the homozygous *Wnt1-G177C* mutation, that causes osteogenesis imperfecta type XV in patients, were used to functionally inactivate Wnt1 signaling. Littermates were used as controls. Subchondral bone microstructure, cartilage degeneration, synovitis and osteophyte formation were assessed by micro-CT and histological evaluation. All experiments were approved by the local animal care committee.

**Results:** Microarray and immunohistochemistry analysis demonstrate that Wnt1 expression is increased in both human and murine OA-subchondral bone. Compared to littermate controls, Early-Tg mice do not show differences in cartilage degeneration after ACLT; however, they exhibit higher subchondral bone mass and attenuated subchondral bone loss. In contrast, Late-Tg mice display a more severe OA phenotype and aberrant sclerosis in subchondral bone when compared to controls.

Mechanistically, secreted frizzled-related protein 2 (Sfrp2) is found induced in OA-subchondral bone by inflammation and potentiates the harmful effects of Wnt1. Finally, *Wnt1-G177C* mutant mice exhibit alleviated ACLT-induced subchondral bone loss and resistance to OA progression.

**Conclusions:** These findings reveal the pathophysiological importance of Wnt1 in subchondral bone remodeling and OA progression, highlighting Wnt1 as a promising therapeutic target for OA.

**P121**

**Notch2 gain-of-function increases posttraumatic osteoarthritis in mice**

*Chiara Gerriets*[1], *Sarina Sczech*[1], *Timur Yorgan*[2], *Assil-Ramin Alimy*[1], *Tim Rolvien*[1], *Julian Kylies*[1], *Anke Baranowsky*[1], *Karl-Heinz Frosch*[1], *Johannes Keller*[1], *Tobias M. Ballhause*[1]
[1]*University Medical Center Hamburg-Eppendorf, Department of Trauma and Orthopedic Surgery, Hamburg, Germany*
[2]*University Medical Center Hamburg-Eppendorf, Department of Osteology and Biomechanics, Hamburg, Germany*

**Abstract Text**

**Background:** Osteoarthritis (OA) affects more than 500 million people worldwide. Even minor injuries to joint cartilage can lead to post-traumatic OA. The molecular mechanisms that lead to OA and subsequent joint degeneration are still not well understood. One promising signaling pathway in the pathophysiological setting of OA is Notch, as ubiquitous inhibition of Notch signaling has been shown to reduce the progression of experimental OA. While the global inactivation of Notch2 results in embryonic lethality, a gain-of-function mutation of Notch2 causes Hajdu-Cheney syndrome (HCS) in humans. HCS is characterized by high bone turnover and early-onset osteoporosis, which is mimicked in mice carrying a pathogenic Notch2 mutation.

**Purpose:** To explore the role of Notch2 in posttraumatic OA in mice.

**Methods:** OA was induced to the right knee via anterior cruciate ligament transection (ACLT) in 12-week-old male WT and Notch2 gain-of-function (HCS) mutant mice. Bilateral knees were harvested four and eight weeks after surgery and analyzed by micro-CT, decalcified histology and qRT-PCR. OA was evaluated with OARSI and inflammation by synovitis score. Experiments were approved by the ethical authorities.

**Results:** Four weeks after ACLT, the mean OARSI score was 2.8 (±1.3) in the operated knee of WT mice and 10.0 (±1.3) in HCS mice ($p=0.0043$). After eight weeks, OARSI scores increased in the WT 6.0 (±1.7) group, but were still lower than in HCS 16.8 (±4.6) mice ($p>0.0001$). Interestingly, contralateral non-operated control HCS knees also developed stronger spontaneous OA compared to WT mice. Inflammatory indices were not altered by Notch2 gain-of-function, however, histomorphometry demonstrated increased osteoclastic activity in HCS mice compared to WT littermates.

**Conclusion:** Notch2 gain-of-function leads to accelerated joint degeneration after ACLT. Cartilage degradation was increased in HCS mutants, the subchondral bone exhibited a parallel increase in osteoclast activity. This study shows the aggravating effect of Notch2 in a posttraumatic model of knee OA.

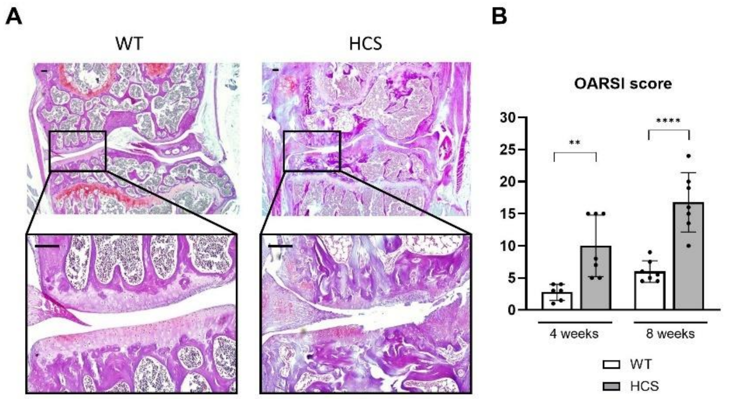

**Figure 1**: Notch2 gain-of-function in mice (HCS) leads to increased osteoarthritis after anterior cruciate ligament transection (ACLT) in comparison to wild type animals (WT). (**A**) Representative images of Bone-Inflammation-Cartilage staining sections of the medial compartments of knees 8 weeks after ACLT (scale bar = 100 µm). (**B**) Total knee joints were scored according to OARSI 4 and 8 weeks after ACLT. The data is expressed as means ± SD; n= 6- 7 per group as indicated (Student's t-tests were used for data analysis). **$p < 0.005$, ****$p < 0.0001$

**P122**

**Serum Amyloid A Deficiency Attenuates Cartilage Degeneration and Subchondral Bone Remodeling in Experimental Osteoarthritis**

*Haoyan Pan*[1], *Weixin Xie*[1], *Ruben Augustin*[1], *Shan Jiang*[1], *Jan Sevecke*[1], *Anke Baranowsky*[1], *Johannes Keller*[1]
*[1]Universitätsklinikum Hamburg-Eppendorf, Department of Trauma and Orthopaedic Surgery, Hamburg, Germany*

**Abstract Text**

**Background:** Osteoarthritis (OA) is the most prevalent degenerative joint disease and a leading cause of pain and disability worldwide. Although inflammation is increasingly recognized as a contributor to OA, its molecular drivers remain insufficiently defined. Serum amyloid A (SAA), a highly inducible acute-phase protein, promotes synovial inflammatory activation and induction of matrix-degrading mediators, however its role in OA pathogenesis remains unclear.

**Purpose:** To determine the impact of SAA deficiency on cartilage degeneration and joint structural alterations in experimental OA.

**Methods:** Osteoarthritis was induced by anterior cruciate ligament transection (ACLT) in SAA knockout (SAA-KO) and wild-type (WT) mice (n = 8 per group). Micro-CT was used to evaluate subchondral bone microarchitecture and osteophyte formation. Histological analyses, including Bone-Inflammation-Cartilage (BIC) staining, were performed to assess cartilage degeneration, subchondral bone alterations, and synovial inflammation. Experiments were approved by the local institutional animal care committee.

**Results:** At 4 weeks post-ACLT, the reduction of subchondral bone volume fraction (BV/TV) in OA knees relative to contralateral controls was significantly greater in WT mice than in SAA-KO mice (−0.302 ± 0.107 vs. −0.164 ± 0.146, $p = 0.043$). Compared to controls, SAA-KO mice showed lower tibial osteophyte volume (0.044 ± 0.028 vs. 0.105 ± 0.034, $p = 0.028$). Histological assessment revealed reduced total joint OARSI scores in SAA-KO mice compared with WT mice (5.88 ± 2.21 vs. 7.94 ± 2.21, $p = 0.013$), accompanied by decreased osteophyte scores (7.6 ± 2.2 vs. 10.5 ± 2.8, $p = 0.043$). However, at 8 weeks post-ACLT, structural and histological parameters did not differ significantly between genotypes.

**Conclusion:** SAA deficiency attenuates early cartilage degeneration and subchondral bone remodeling following ACLT but does not influence late-stage outcomes, indicating that SAA mainly contributes to the early phase of post-traumatic joint degeneration.

**P123**

### Comparative Analysis of Activin A in Chronic and Acute Arthritis Pathogenesis

*__Corinna Wehmeyer__[1], Vanessa Waltereit-Kracke[1], Berno Dankbar[1], Thomas Pap[1], Denise Beckmann[1]*
*[1]University of Muenster, Institute of Musculoskeletal Medicine, Muenster, Germany*

### Abstract Text

**Background:** Activin A, a TGF-β superfamily member, has diverse immunomodulatory functions in tissue homeostasis and inflammation. In rheumatoid arthritis (RA), its role remains unclear, with both pro- and anti-inflammatory activities reported depending on cell type and disease stage. We previously showed that chronic inflammation induces activin A in synovial tissues of hTNFtg mice, a chronic arthritis model closely resembling human RA, and that mesenchymal cell-specific activin A deletion reduces synovial inflammation, bone erosion, and osteoclast formation. Understanding its contribution across chronic and acute arthritis models is therefore essential.

**Purpose:** To determine the impact of mesenchymal cell–derived activin A in chronic and acute RA mouse models.

**Methods:** Activin A expression was assessed by immunohistochemistry. ColVI-Cre mice were crossed with $Act\beta A^{f/f}$ mice to generate mesenchymal cell-specific activin A-deficient mice. Acute arthritis was induced using the acute K/BxN serum-transfer model (K/BxN-STIA). Disease severity was evaluated by clinical scoring, histomorphometry of synovial infiltration and bone erosion, µCT imaging of structural damage, and TRAP staining for osteoclasts.

**Results:** Activin A was highly expressed in inflamed K/BxN-STIA joints. Unexpectedly, mesenchymal activin A deletion ($Act\beta A^{d/d}$; ColVI-Cre) aggravated acute arthritis, with increased ankle swelling

(+20.3%), reduced grip strength (-66%), and 2-fold higher joint scores by day 14 compared with controls. µCT and histomorphometry confirmed markedly enhanced joint destruction, including ~8.4-fold more inflamed tissue, ~4.4-fold more bone erosion, and ~8.2-fold more osteoclasts. No abnormalities occurred without serum transfer. In contrast, the same deletion reduced inflammation, bone erosion, and osteoclast numbers by 35-45% in hTNFtg mice, demonstrating opposing effects in chronic disease.

**Conclusion:** Mesenchymal cell-specific activin A deficiency strongly exacerbates inflammation and joint destruction in acute K/BxN-STIA, whereas it is protective in the chronic hTNFtg model. These findings highlight context-dependent roles of activin A in arthritis pathogenesis.

**P124**

**A case of intracapsular chondroma of the knee: The value of multimodal imaging with emphasis on ultrasound findings.**

*Nestoras Avgoustidis*[1], *Apostolos Karantanas*[2], *George Kakkos*[3], *Nikolaos Kougkas*[4]
[1]*School of Medicine - University of Crete,*
*Department of Rheumatology & Clinical Immunology - University Hospital of Heraklion - Crete - Greece, Heraklion, Greece*
[2]*School of Medicine- University of Crete,*
*Department of Medical Imaging- University Hospital of Heraklion- Heraklion- Crete- Greece, Heraklion, Greece*
[3]*School of Medicine - University of Crete,*
*Department of Medical Imaging- University Hospital of Heraklion- Crete- Greece, Heraklion, Greece*
[4]*Medical School- Aristotle University of Thessaloniki,*
*Fourth Department of Internal Medicine- Hippokration University Hospital, Thessaloniki, Greece*

**Abstract Text**

**Background:** Intracapsular chondroma is a rare benign lesion of the large joints mostly the knee. There are located anteriorly, in the infrapatellar region, usually on the medial aspect. Multimodal imaging can guide diagnosis avoiding invasive procedures.

**Case Discussion:** A 34-year-old woman had mild discomfort in the anterior knee for the last three months. The patient had no history of trauma or previous surgery. Laboratories were unremarkable including inflammatory markers. On physical examination there was a firm, movable, slightly tender mass in the infrapatellar area.  Multimodal imaging was performed and findings are summarized in a Figure. Based on these imaging features, the diagnosis of intracapsular chondroma was made.

Infrapatellar chondroma is a rare entity with specific imaging features, especially on MRI. The knowledge of these features is the key for the differential diagnosis. The ultrasound could also attribute to the initial evaluation of the lesion. The characteristic ultrasound findings of intracapsular chondroma include 1) a well delineated hypoechoic tissue corresponding to the cartilaginous component of the chondroma, 2) peripheral and central calcifications 3) in some cases focal ossifications, 4) no Power Doppler (PD) signal within lesion. It is important to be differentiated from

Pigmented Villonodular Synovitis (PVNS), synovial chondromatosis and chondrosarcoma.  In accordance to the above excision of the lesion and histological examination might be necessary only in doubtful cases or when the chondroma cause symptoms to the patient.  Malignant transformation of intracapsular chondroma has never been reported and local recurrence is rare

**Conclusion:** Using correct imaging modalities for diagnosis of lesions in infrapatellar area unnecessarily aggressive surgical treatment and diagnostic delays will be avoided

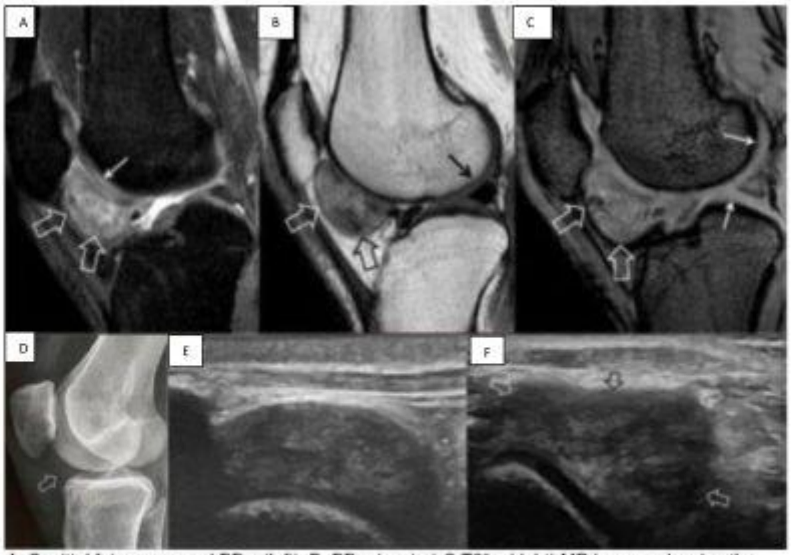

A. Sagittal fat suppressed PDw (left), B. PDw (center) C.T2*w (right) MR images showing the lesion (open arrows) with signal intensity which is similar to that of the articular cartilage (thin arrows) as shown in different pulse sequences. The findings are pathognomonic for intracapsular chondroma.
D. Lateral radiograph showing faint calcifications within the Hoffa's fat pad (open arrow).
E Ultrasonography-infrapatellar area. Longitudinal (left)  F.Transverse (right) view: showing a primarily hypoechoic lesion with well-defined borders. (open arrows) beneath of patellar tendon in the area of Hoffa's fat pad with some calcified areas.

**P125**

**The effect of exercise and physical activity on gait biomechanics and pain in people with knee osteoarthritis - A systematic review with meta-analysis**

*Nathan Bytheway[1], Shadi Amor[1], Paul Swinton[2], Kevin Cheah[3],*
*Andrew Morrison[4], Jasmine Samvelyan[1]*
*[1]Anglia Ruskin University, School of Medicine, Chelmsford, United Kingdom*
*[2]Robert Gordon University, School of Health, Aberdeen, United Kingdom*
*[3]Nuffield Health, Brentwood Hospital, Brentwood, United Kingdom*
*[4]Anglia Ruskin University, School of Psychology- Sport and Sensory Science, Cambridge, United Kingdom*

**Abstract Text**

**Introduction:** Osteoarthritis of the knee is the most prevalent type of osteoarthritis affecting 5.4million people in the UK leading to increasing healthcare costs. Exercise has been shown to help symptoms in people with knee osteoarthritis, however, there are unanswered questions about the effects of types of exercise/physical activity on mobility and pain.

**Purpose:** A systematic review of literature was carried out to provide an evaluation and review of current literature to determine the effects of physical activity and performance of specific exercises on gait characteristics and pain in people with knee osteoarthritis.

**Methods:** Clinical trials published in English up to 31 December 2024 were retrieved from 4 electronic databases for cross-referencing and expert review. The primary outcome measures were changes in gait biomechanics including kinematic and kinetic parameters. The secondary outcomes included changes in pain and mobility assessed through questionnaires such as Western Ontario and McMaster Universities Arthritis Index.

**Results:** A summary of the studies included in the review is shown in Figure 1. Meta-analysis indicated that exercise or physical activity intervention reduced pain (effect size 0.61; 95% credible interval [CrI] 0.29 to 0.92) with significant benefits on pain reduction observed in studies that used multi-component exercise/physical activities compared with control groups. Spatiotemporal parameters such as walking speed, step/stride length and width, cadence, swing, or stance phase duration improved following the intervention (effect size 0.67; 95% credible interval [CrI] 0.27 to 1.05) whereas three-dimensional patterns of the knee joint including kinematic (effect size 0.12; 95% credible interval [CrI] -0.18 to 0.41) and kinetic (effect size -0.03; 95% credible interval [CrI] -0.25 to 0.18) changes were least affected.

**Conclusions:** The findings of this research may form a basis for recommendations of targeted physical activity interventions for the benefits of patients with knee osteoarthritis including pain reduction, mobility improvement and gait modification.

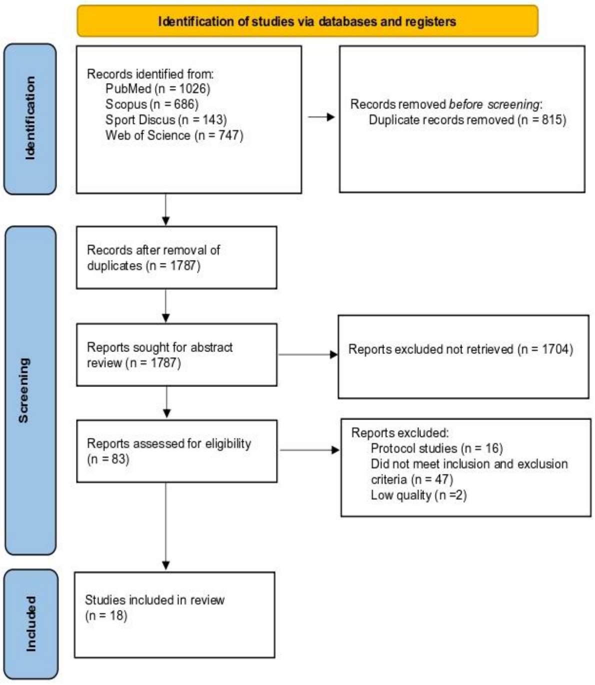

**Figure 1. Search flow diagram**

**P126**

**Regulatory Role of AVPR1A in Osteoarthritis Pathogenesis**

*Sujin Kim[1], Gyuseok Lee[2], Ka Hyon Park[2], Jeong-Tae Koh[2], Je-Hwang Ryu[2]*
*[1]chonnam national university, chonnam national university, Gwangju, Korea- South*
*[2]Chonnam National University,*
*Department of Pharmacology and Dental Therapeutics- School of Dentistry, Gwangju, Korea- South*

**Abstract Text**

**Purpose:** Osteoarthritis (OA) is a degenerative joint disease characterized by the progressive breakdown of joint tissues, primarily marked by persistent pain resulting from articular cartilage degradation. Despite its clinical significance, the molecular mechanisms underlying OA remain insufficiently understood. Arginine vasopressin (AVP), an antidiuretic hormone acting via three receptors (V1a, V1b, and V2), signals through AVPR1A, a G-protein-coupled receptor involved in bone remodeling and pain regulation. This study investigates the role of AVPR1A in OA pathogenesis and its potential as a therapeutic target.

**Methods:** The expression of AVPR1A in articular chondrocytes was analyzed using quantitative RT-PCR and Western blot. The role of AVPR1A in OA was examined through overexpression using adenovirus-mediated gene delivery and knockdown approaches with specific inhibitors or genetically deficient (Avpr1a$^{-/-}$) mice.

**Results:** During OA progression, we observed a specific upregulation of AVPR1A, a receptor for vasopressin (AVP), in cartilage. We aimed to elucidate the molecular link OA through the AVP-AVPR1A axis. Overexpression of AVPR1A in joints through Ad-*Avpr1a* injection did not affect joint degradation, whereas overexpression via the AVP-AVPR1A axis exacerbated the symptoms of DMM-induced post-traumatic OA. In contrast, inhibition of AVPR1A function or expression, either by the AVPR1A antagonist relcovaptan or through *Avpr1a*$^{-/-}$ mice, suppressed joint destruction induced by DMM. Specifically, we show that the AVP-AVPR1A pathway plays an important role in the pathogenesis of OA by regulating the expression of matrix-degrading enzymes in chondrocytes.

**Conclusion:** This study confirmed that the expression of AVPR1A is specifically upregulated in cartilage during the progression of OA, and genetic deficiency of AVPR1A in vivo ameliorated DMM-induced OA damage. These findings suggest that targeting the AVP-AVPR1A pathway could be a novel therapeutic strategy for OA.

**Keywords** Osteoarthritis, chondrocyte, AVPR1A

**P127**

### CRISPR-Cas9-Mediated Genome Editing for Epas1, and Ch25h in Knee Joints Reduces Cartilage Degradation and Knee Pain

*Gyuseok Lee[1], Ka-Hyon Park[1], Su-Jin Kim[1], Jeong-Tae Koh[1], Je-Hwang Ryu[1]*
*[1]Chonnam National University,*
*Department of Pharmacology and Dental Therapeutics- School of Dentistry, Gwangju, Korea- South*

### Abstract Text

**Purpose:** CRISPR-Cas9-mediated gene editing offers a promising approach for the silencing of pathogenic genes in osteoarthritis (OA). This study assesses the in vivo efficacy of two delivery platforms, Adeno-associated viruses (AAVs) and engineered virus-like particles (eVLPs), in targeting three OA-regulating genes: *Ch25h* (cytoplasmic enzyme for oxysterol synthesis), *Epas1* (nuclear transcription factor), and *Slc39a8* (membrane transporter for zinc influx). By comparing editing efficiency and therapeutic outcomes, we aimed to identify the most effective gene editing strategy and gene target across different cellular compartments for OA treatment.

**Methods:** AAVs encoding SaCas9 and screened single-guide RNA (sgRNA) expression cassettes, as well as eVLPs loaded with SpCas9 protein and selected sgRNAs, were intra-articularly injected into the knee joints of mice subjected to destabilization of the medial meniscus surgery. Genomic DNA was extracted from meniscus and frequencies of insertion/deletion were measured by next generation sequencing. The degree of OA progression was evaluated by safranin O staining and semi-quantitative scoring, and pain assessment.

**Results:** Three different AAV-SaCas9s administered three times achieved ~25% indel frequencies in mouse meniscus tissue, identifying *Epas1* and *Ch25h* as key therapeutic targets. eVLP-SpCas9

targeting *Epas1* and *Ch25h* enabled more efficient editing, with 35–40% indel frequencies in mouse meniscus with a single injection. In the DMM-induced OA mouse model, targeting *Epas1* and/or *Ch25h* via eVLP-SpCas9 significantly reduced joint pain and attenuated cartilage degradation.

**Conclusions:** These findings demonstrate that Cas9-mediated gene editing targeting transcriptional, and metabolic regulators effectively suppresses OA progression, while editing of the membrane transporter showed minimal benefit. Notably, eVLP-mediated SpCas9 delivery achieved higher indel efficiencies than AAV-SaCas9 and significantly reduced joint pain and cartilage damage in DMM-induced OA model. These findings highlight the therapeutic value of *Epas1* and *Ch25h* and validate eVLPs as an effective platform for in vivo gene editing in joint tissues.

**P128**

**Bone Properties and Biomechanics in Patients with Psoriatic Disease: A Cross-sectional Study with High-Resolution Peripheral Quantitative Computed Tomography (HRpQCT)**

*<u>Giovanni Adami</u>[1], Anna Piccinelli[1], Francesco Bellinato[2], Paolo Gisondi[2], Davide Gatti[1], Maurizio Rossini[1]*
*[1]University of Verona, Rheumatology Unit, Verona, Italy*
*[2]University of Verona, Dermatology Unit, Verona, Italy*

**Abstract Text**

**Background**: Psoriatic disease has a complex effect on bone metabolism, resulting in both pathological bone formation and bone resorption. However, microstructural changes in cortical and trabecular compartment remain poorly understood. The aim of this study was to investigate the prevalence and determinants of bone microarchitectural damage in patients with psoriatic disease.

**Methods**: We performed a cross-sectional study on patients with Psoriasis (PsO), Psoriatic arthritis (PsA) and age-matched healthy controls (HC) recruited into the department of Dermatology and Rheumatology of Verona medical center. We conducted High-Resolution peripheral Quantitative Computed Tomography (HRpQCT) of the radius and finger joints of the non-dominant hand. Bone microstructure parameters and Finite Element Analysis (uFEA) were calculated.

**Results**: Fifty-one patients with PsO and 39 patients with PsA were consecutively enrolled in the study. Twenty-four age-matched HC were enrolled. Distal radius total and cortical volumetric Bone Mineral Density (vBMD) levels were lower in patients with PsA and PsO compared with HC. On distal radius uFEA analysis we found a significant reduction of stiffness in PsA compared to both HC and PsO (figure and table). At the distal interphalangeal (DIP) joints, cortical vBMD and trabecular vBMD were lower in PsA and PsO compared to HC. Nail involvement in psoriatic disease was negatively associated with bone stiffness at the proximal and distal region of DIP.

**Conclusion**: Patients with psoriatic disease have lower bone density and microarchitectural alterations that impact biomechanical properties. Nail involvement was associated with decreased bone stiffness in psoriatic disease.

| Radius | | | | | | | |
|---|---|---|---|---|---|---|---|
| Microstructure parameter | HC | | PsO | | PsA | | p value |
| | Mean | SD | Mean | SD | Mean | SD | |
| Bone Volume $mm^3$ | 1150.9 | 181.2 | 1372.5 | 341.5 | 1373.4 | 269.5 | NS |
| BV/TV % | 46.7 | 3.1 | 46.3 | 2.7 | 45.4 | 2.7 | NS |
| Tb.BV/TV % | 24.0 | 2.1 | 25.6 | 3.4 | 25.2 | 2.8 | NS |
| BS $mm^2$ | 6171.0 | 1011.0 | 7825.9 | 2493.5 | 7821.3 | 1985.1 | NS |
| Trabecular number 1/mm | 1.011 | 0.037 | 1.049 | 0.075 | 1.032 | 0.053 | NS |
| Cortical Thickness mm | 0.949 | 0.076 | 0.877 | 0.128 | 0.878 | 0.100 | NS |
| Trabecular Thickness | 0.364 | 0.014 | 0.369 | 0.017 | 0.368 | 0.019 | NS |
| Trabecular Spacing | 0.621 | 0.038 | 0.604 | 0.056 | 0.621 | 0.060 | NS |
| Total Bone Mineral Density mgHA/$cm^3$ | 635 | 66 | 537 | 125 | 531 | 109 | ***, °°° |
| Cortical Bone Mineral Density mgHA/$cm^3$ | 929 | 77 | 806 | 135 | 810 | 125 | ***, °°° |
| Trabecular Bone Mineral Density mgHA/$cm^3$ | 320 | 64 | 285 | 76 | 280 | 92 | NS |
| Stiffness kN/mm | 53.4 | 13.5 | 36.4 | 17.5 | 24.7 | 14.3 | °°°, §§ |
| Cortical bricks failure load at 1500N % | 0.863 | 0.339 | 1.306 | 0.646 | 2.577 | 2.605 | °°°, § |
| Trabecular bricks failure load at 1500N % | 9.239 | 6.258 | 10.463 | 6.580 | 15.952 | 8.019 | § |
| Proximal region of the distal interphalangeal joints (II and III) | | | | | | | |
| Microstructure parameter | HC | | PsO | | PsA | | p value |
| | Mean | SD | Mean | SD | Mean | SD | |
| Bone Volume $mm^3$ | 218.8 | 31.2 | 229.1 | 46.6 | 205.8 | 41.1 | NS |
| BV/TV % | 64.2 | 3.8 | 65.5 | 5.0 | 62.1 | 5.1 | NS |
| Tb.BV/TV % | 11.94 | 2.8 | 13.7 | 2.6 | 13.4 | 3.0 | NS |
| BS $mm^2$ | 812.9 | 133.6 | 888.3 | 208.7 | 841.9 | 168.1 | NS |
| Trabecular number 1/mm | 1.154 | 0.073 | 1.143 | 0.108 | 1.126 | 0.141 | NS |
| Cortical Thickness mm | 0.921 | 0.108 | 0.876 | 0.118 | 0.836 | 0.119 | NS |
| Trabecular Thickness | 0.271 | 0.021 | 0.285 | 0.024 | 0,277 | 0.022 | NS |
| Trabecular Spacing | 0.599 | 0.050 | 0.597 | 0.071 | 0.629 | 0.151 | NS |
| Total Bone Mineral Density mgHA/$cm^3$ | 1181 | 99 | 960 | 195 | 995 | 125 | °, §§ |
| Cortical Bone Mineral Density mgHA/$cm^3$ | 1214 | 103 | 1029 | 115 | 988 | 118 | °°, §§ |
| Trabecular Bone Mineral Density mgHA/$cm^3$ | 926 | 110 | 773 | 81 | 753 | 102 | °, §§ |
| Stiffness kN/mm | 128.1 | 49.1 | 70.4 | 23.5 | 51.1 | 27.5 | *, °°, §§ |
| Cortical bricks failure load at 350N % | 1.521 | 1.185 | 2.009 | 1.527 | 2.368 | 1.855 | *, °, §§ |
| Trabecular bricks failure load at 350N % | 15.307 | 4.239 | 12.15 | 2.963 | 13.732 | 5.840 | NS |
| Distal region of the distal interphalangeal joints (II and III) | | | | | | | |
| Microstructure parameter | HC | | PsO | | PsA | | p value |
| | Mean | SD | Mean | SD | Mean | SD | |
| Bone Volume $mm^3$ | 137.3 | 24.8 | 155.7 | 36.1 | 131.9 | 34.1 | NS |
| BV/TV % | 60.9 | 2.9 | 59.7 | 3.4 | 60.9 | 2.59 | NS |
| Tb.BV/TV % | 14.6 | 2.3 | 15.4 | 2.4 | 13.4 | 2.2 | NS |
| BS $mm^2$ | 627.3 | 108.4 | 728.1 | 189.9 | 611.6 | 155.99 | NS |
| Trabecular number 1/mm | 1.147 | 0.092 | 1.161 | 0.082 | 1.173 | 0.090 | NS |
| Cortical Thickness mm | 0.736 | 0.062 | 0.697 | 0.048 | 0.701 | 0.05 | NS |
| Trabecular Thickness | 0.252 | 0.019 | 0.268 | 0.028 | 0.245 | 0.029 | NS |
| Trabecular Spacing | 0.625 | 0.480 | 0.597 | 0.056 | 0.612 | 0.071 | NS |
| Total Bone Mineral Density mgHA/$cm^3$ | 958 | 180 | 785 | 167 | 849 | 187 | NS |
| Cortical Bone Mineral Density mgHA/$cm^3$ | 999 | 170 | 826 | 173 | 883 | 189 | **, °° |
| Trabecular Bone Mineral Density mgHA/$cm^3$ | 630 | 129 | 498 | 140 | 551 | 176 | *, ° |
| Stiffness kN/mm | 36.3 | 14.6 | 18.24 | 9.0 | 24.7 | 14.6 | **, °°, §§ |
| Cortical bricks failure load at 350N % | 2.093 | 2.012 | 3.292 | 2.038 | 3.312 | 2.035 | *, ° |
| Trabecular bricks failure load at 350N % | 13.897 | 4.658 | 12.529 | 4.773 | 14.596 | 3.660 | NS |

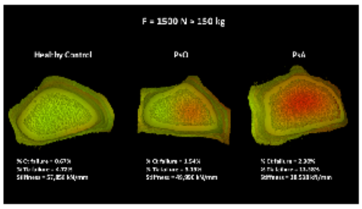

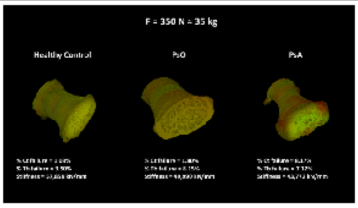

**p value between HC and PsO <0.05; **p value between HC and PsO <0.01; ***p value between HC and PsO <0.001*

*°p value between HC and PsA <0.05; °°p value between HC and PsA <0.01; °°°p value between HC and PsA <0.001*

*§p value between PsO and PsA <0.05; §§p value between PsO and PsA <0.01; §§§p value between PsO and PsA <0.001*

**P129**

**Sex and Hormonal Status Influence Early Post-Traumatic Osteoarthritis: Insights comparing Male, Female, and Ovariectomized Mouse Models**

*Wacharapol Saengsiwaritt[1], John J. Cole[2], Lynette Dunning[3], Kendal McCulloch[3],*

*Wanvisa Udomsinprasert[1], Rob van 't Hof[4], Carl Goodyear[5], John Lockhart[3], Carmen Huesa[5]*
*[1]Mahidol University, Faculty of Pharmacy, Bangkok, Thailand*
*[2]University of Glasgow, MVLS Research Facilities, Glasgow, United Kingdom*
*[3]University of the West of Scotland, Institute of Biomedical & Envirnmental Health Research, Paisley, United Kingdom*
*[4]Vanthof Scientific S.C., Vanthof Scientific S.C., Torun, Poland*
*[5]University of Glasgow, School of infection and immunity, Glasgow, United Kingdom*

**Abstract Text**

Osteoarthritis (OA) is a degenerative joint disorder in which multiple tissues within the joint interact to drive disease progression, with pain being a key clinical feature. The disease is more prevalent in women over the age of 50, coinciding with menopause, which profoundly affects joint health through hormonal changes that drive structural and metabolic alterations in bone, cartilage, and other tissues, making the joint particularly vulnerable to degeneration. This study investigated sex-related disparities and the impact of hormonal depletion on early-stage knee OA by comparing male, female, and ovariectomized (OVX) mice.

OA was induced in C57BL/6 mice (n=15 per group) using destabilization of the medial meniscus combined with cartilage scratch (DCS), an accelerated post-traumatic OA model characterized by early pain. Sham-operated controls (n=4–5) were included. Two weeks post-surgery, joint architecture was assessed by histology and micro-computed tomography, pain behavior by dynamic weight-bearing (DWB), and dorsal root ganglia (DRG) transcriptomes analyzed by bulk RNA sequencing. Serum estradiol and IL-6 were measured by ELISA.

Both male and female OA groups exhibited increased subchondral bone sclerosis ($P \leq 0.05$), whereas OVX mice did not. OVX mice showed significantly higher joint inflammation compared to males ($P \leq 0.001$) and females ($P \leq 0.01$). Estradiol levels in OA females were similar to OVX and markedly reduced compared to sham females ($P=0.0005$). Only males demonstrated unloading of the operated limb, indicative of pain, which correlated with DRG transcriptomic changes involving genes linked to joint architecture and pain sensitivity. OVX mice displayed distinct inflammatory gene profiles compared to intact females.

These findings suggest that sex and hormonal status influence murine OA pathology, with males showing greater pain sensitivity and morphological changes, while estrogen depletion exacerbates joint inflammation. These results should be interpreted cautiously regarding clinical relevance, as mouse models cannot capture the complex hormonal, metabolic, and biomechanical interactions present in human OA.

**P130**

**CD248/Endosialin regulates synovial fibroblast state to control coupling of inflammation and bone damage in murine models of immune-mediated inflammatory arthritis and ageing**

*Samuel Kemble[1], Corinna Wehmeyer[2], James Brown[3], Charlotte Smith[1], Holly Adams[1], Eva Crombie[1], Harris Perlman[4], Christopher Buckley[5], Adam Croft[1], Amy Naylor[1]*

[1]University of Birmingham, Inflammation and Ageing, Birmingham, United Kingdom
[2]Universitat Muenster, Musculoskeletal Medicine, Muenster, Germany
[3]University of Lincoln, Engineering and Physical Sciences, Lincoln, United Kingdom
[4]Northwestern University, Feinburg School of Medicine, Evanston, United States
[5]University of Oxford, Kennedy Institute of Rheumatology, Oxford, United Kingdom

**Abstract Text**

**Background:** Synovial fibroblasts directly contribute to inflammation and bone damage in immune-mediated inflammatory arthritis. CD248/Endosialin is expressed by fibroblasts in the synovium, is highly upregulated during inflammation and has been proposed as a potential therapeutic target for rheumatoid arthritis.

**Purpose:** This study aimed to map the expression of CD248 in disease-associated fibroblast states and to elucidate its role in pathology.

**Methods:** Molecular profiling of stromal cells from healthy and arthritic (K/BxN serum transfer-induced arthritis) wildtype (*Cd248*$^{+/+}$) and *Cd248*$^{-/-}$ mice was performed using 3' single cell RNA sequencing. Arthritis trajectory and severity were assessed in both genotypes by clinical scoring, microcomputed tomography (microCT), histology and flow cytometry of dissociated synovium. Additionally, healthy mice of each genotype were aged to 20 months and hind limbs assessed by microCT and histology.

**Results:** Cells expressing high levels of *Cd248* also express the '*Pi16*' fibroblast progenitor gene signature. Genetic deletion of *Cd248* in mice altered the differentiation trajectory of these progenitors, predisposing them to become destructive lining layer fibroblasts (lining layer fibroblasts comprised 8% of total synovial fibroblasts in *Cd248*$^{+/+}$ compared to 12% in *CD248*$^{-/-}$($p<0.05$)), and inducing osteoclast formation (see figure). Consequently, *Cd248*$^{-/-}$ arthritic mice showed a phenotype of rapid, extensive bone damage despite no significant difference in inflammation (measured by swelling and infiltrating Cd45+ cells). Unexpectedly, bone erosion and deformity was also observed in aged *Cd248*$^{-/-}$ mice in the absence of an inflammatory stimulus (see figure), but not in younger mice (aged <40 weeks).

**Conclusion:** Genetic deletion of *Cd248*$^{-/-}$ results in uncoupling of inflammation and damage with dysregulated fibroblast progenitor differentiation. Our data suggests that the high levels of CD248 expression seen on synovial fibroblasts provides a protective brake that attenuates bone damage by preventing the polarisation of emergent fibroblasts towards a destructive phenotype. Therapeutic targeting of this protein may exacerbate disease.

**P131**

**Developing a standard dataset for adult chronic non-bacterial osteitis (CNO) in the European registries for rare endocrine and bone conditions (EuRREB)**

*Gitanjali Sukhtankar[1], Mariya Cherenko[1], Anne T Leerling[2], Simone Appenzellers[3], Gunter Assmann[4], Judith Babbear[5], Roland Chapurlat[6], Gavin Clunie[7], Martine Cohen Solal[8], Salvatore D'Angelo[9],*

*Dmitirios Daoussis[10], Olaf M Dekkers[2], Olivier Fogel[11,12], Victoria Furer[13], Roba Ghossan[14], Tiara Gill[15], Hermann Girschick[16], Neveen A.T Hamdy[17], Barbara Hauser[18], Gilles Hayem[19], Philip Helliwell[20], Antonella Insalaco[21], Aruni Jayatilleke[22], Mitsumasa Kishimoto[23], Mikhail Kostik[24], Willem Lems[25], Paivi Miettunen[26], Natalia Palmou[27], Ahimalaipet V Ramanan[28], Marion Roderick[28], Murugan Sudhakar[29], James Teh[30], Charlotte Verroken[31], Yongdong Zhao[32], Hans-Georg Zmierczak[31], Natasha M Appelman-Dijkstra-[1], Elizabeth Winter[1]*

*[1]Leiden University Medical Center,*
*Department of Internal Medicine- Division of Endocrinology and Center for Bone Quality, Leiden, Netherlands*
*[2]Leiden University Medical Center, Department of Clinical Epidemiology, Leiden, Netherlands*
*[3]Rheumatology and Traumatology DORT- School of Medical Science- University of Campinas UNICAM P, Department of Orthopedics, Sao Paolo, Brazil*
*[4]Ruhr-university medical faculty, JWK Minden, Minden, Germany*
*[5]Centre for Metabolic Bone Disease, Royal National Orthopaedic Hospital, London, United Kingdom*
*[6]Hospices Civils de Lyon- Université Claude Bernard-Lyon 1, inserm umr 1033, Lyon, France*
*[7]Cambridge University Hospitals, Department of Rheumatology, Cambridge, United Kingdom*
*[8]Lariboisiere hospital, Rheumatology Department, Paris, France*
*[9]University of Basilicata, Department of Health Science, Potenza, Italy*
*[10]Patras University Hospital- Greece, Department of Internal Medicine- Division of Rheumatology, Rio, Greece*
*[11]Cochin Hospital- Université Paris Cité and Université Sorbonne Paris Nord-,*
*Department of Rheumatology, Paris, France*
*[12]Centre for Research in Epidemiology and Statistics CRESS-, Inserm- INRAE, Paris, France*
*[13]Tel Aviv University,*
*Rheumatology Department- Tel Aviv Sourasky Medical Center- School of Medicine- Faculty of Medical and Health Sciences, Tel Aviv, Israel*
*[14]Hospital Cochin, Department of Rheumatology, Paris, France*
*[15]Royal National Orthopaedic Hospital, Metabolic Bone Disease Unit, Stanmore, United Kingdom*
*[16]Vivantes Klinikum im Friedrichshain, Department of Pediatrics, Berlin, Germany*
*[17]Leiden University Medical Center, Department of Internal Medicine- Division of Endocrinology, Leiden, Netherlands*
*[18]Western General Hospital, Rheumatic Diseases Unit, Edinburgh, United Kingdom*
*[19]Paris-Saint Joseph Hospital Group, Department of Rheumatology, Paris, France*
*[20]University of Leeds, Leeds Institute of Rheumatic and Musculoskeletal Medicine, Leeds, United Kingdom*
*[21]IRCCS Bambino Gesù Children's Hospital- ERN- RITA center, Department of Rheumatology, Rome, Italy*
*[22]Lewis Katz School of Medicine at Temple University, Rheumatology- Department of Medicine-, Philadelphia, United States*
*[23]Kyorin University School of Medicine, Department of Nephrology and Rheumatology, Tokyo, Japan*
*[24]Saint-Petersburg State Pediatric Medical University, Saint-*
*Petersburg State Pediatric Medical University, Saint-Petersburg, Russia C.I.S.*
*[25]Amsterdam University Medical Center, Department of Rheumatology, Amsterdam, Netherlands*
*[26]University of Calgary McCaig Institute for Bone and Joint Health- Calgary,*
*Department of Paediatrics, Alberta, Canada*
*[27]Hospital Universitario Marques de Valdecilla- Santander,*

*Department of Rheumatology and Pediatric Rheumatology- Immunology Group, Cantabria, Spain*
[28]*Bristol Royal Hospital for Children BRHC, Department of Paediatric Immunology, Bristol, United Kingdom*
[29]*Christian Medical College, Department of Pediatrics, Vellore, India*
[30]*Nuffield Orthopaedic Centre Girdlestone Memorial Library, Department of Radiology, Oxford, United Kingdom*
[31]*Ghent University Hospital, Department of Endocrinology- Unit for Osteoporosis and Metabolic Bone Diseases, Ghent, Belgium*
[32]*Seattle Children's Hospital, Department of Rheumatology, Washington, United States*

**Abstract Text**

**Background:** Chronic non-bacterial osteitis (CNO) is a heterogenous, rare, sterile inflammatory bone disorder characterized by painful, sclerotic lesions most often involving the anterior chest wall, but may affect any bone in adults. Pathophysiology of CNO is poorly understood, lacking validated diagnostics and standardized treatments.

**Purpose & Methods:** The Core Registry within the European Registries for Rare Endocrine and Bone Conditions (EuRREB) collects mandatory Core Data Elements for ERN registries and allows a specific dataset within a condition-specific module (CSM), including clinical data and Patient Reported Outcome Measures (PROMS). Development of the CNO module occurred over 1.5 years through multi-stakeholder (ECTS, ERN-BOND, ECE) collaboration of clinicians, researchers, and a patient advocacy group (CNO-SCCH NL).

The aim was to create a framework supporting observational studies leading to future interventional projects elucidating the CNO phenotype, disease-course, pathophysiology, diagnosis, and treatment.

This module was derived from the 2024 consensus guidelines for CNO diagnosis and treatment and refined through a 9-point Likert scale survey (n=29) providing iterative feedback during testing. Variables were included if 80% scored >7 on the Likert scale.

**Results:** All 52 proposed variables (i.e., patient history, past/current medication, diagnostic tools, ASAS/CASPAR criteria, bone-pain, imaging, clinical follow-up, among others) were included. PROMS included EQ-5D, BPI-SF, and BFI-SF questionnaires. A beta version was launched August 2025 featuring CNO-unique variables. After incorporating revisions from the final feedback round, the completed module was launched on 01-12-2025.

**Conclusion:** This module is the first multicenter initiative to prospectively monitor and longitudinally characterize CNO patients. Addressing rare-disease research challenges via international data collection, this registry provides a powerful platform to meet patient needs and improve clinical care. As participation expands, data will enable studies of clinical practices, disease course, treatment effects, and geographical variability. Researchers and clinicians worldwide may request access to the module to support collaborative CNO research.

**P132**

### Articular, metabolic and epigenetic characterisation of the effects of a ketogenic diet on the progression of osteoarthritis in obese adult male mice

*Asmaa EL Maarbani[1], Thomas Solé[1], Jagadeesh K. Venkatesan[2], Magali Cucchiarini[3], Luciano Pirola[4], Maura Strigini[1]*
[1]*University Jean Monnet Saint-Etienne, Faculty of medicine- SAINBIOSE lab, Saint-Etienne, France*
[2]*Saarland University, University Hospital- Orthopedics surgery department, Homburg, Germany*
[3]*Saarland University, Orthopedic surgery department, Homburg, Germany*
[4]*Université Claude Bernard Lyon 1, CARMEN Laboratory- INSERM U1060- Faculty of medicine, Lyon, France*

### Abstract Text

Obesity, with its low-grade metabolic inflammation, unfavorable adipokine profile, and excessive mechanical loading on joints, exacerbates the progression of osteoarthritis (OA). A low carb/high fat ketogenic diet (KD) can promote weight loss, reduce metabolic inflammation and elevate circulating levels of the ketone body beta-hydroxybutyrate (BHB).

We fed mice a high-fat diet (HFD) for 10 weeks, and induced knee OA by medial meniscus destabilization at 16 weeks of age; we then fed mice one of three diets ad libitum: HFD, KD or standard diet (STD) for 8 weeks.

BHB levels increased ten-fold in KD group only. Glycemia remained high in HFD mice but decreased in STD and even more in KD ($p < 0,001$). HFD mice continued to gain weight, while STD and KD lost weight to a comparable extent.

OA progression was moderately limited by KD: Severe OA cases (OARSI score>2.5) were less frequent (HFD>STD>KD, $p=0.037$); osteophytes were smaller ($p < 0.001$). For OA marker genes in cartilage, MMP13 was 2-fold lower in KD compared to HFD ($p=0.005$) and to STD ($p=0.02$); OPN was lower in KD than in HFD ($p<0.05$). COL 10 was reduced in KD compared to STD ($p<0.05$). Leptin expression was lower in KD than in both HFD and STD ($p<0.05$), whereas p16 expression was lower in KD compared to HFD ($p<0.05$). Western blot analysis supported these findings.

Specific metabolic signatures were associated with the diets in serum (metabolomics), including changes in bile acid profiles, and liver, kidney and joint tissue, including distinct ketogenesis and ketolysis gene expression profiles in joint tissue.

KD significantly increased histone beta-hydroxybutyrylation in articular chondrocytes, with no effects on histone acetylation.

Mitochondrial downregulation may reflect a metabolic shift toward a more energy-conserving state in human chondrocytes.

KD demonstrates a moderate beneficial effect on disease progression in a post-traumatic OA murine model compounded by obesity.

**ND-P013**

### Frequencies of osteochondroprogenitor and inflammatory cells in the synovial compartment of mice with osteoarthritis

*Tomislav Balen*[1,2], *Marta Radošević*[2,3], *Sara Aničić*[2,3], *Sara Priselac*[1,2], *Ivo Krešić*[4], *Pavao Planinić*[4], *Darja Flegar*[2,3], *Alan Šućur*[2,3], *Tomislav Kelava*[2,3], *Danka Grčević*[2,3], *Nataša Kovačić*[1,2]
[1]*University of Zagreb School of Medicine, Department of Anatomy, Zagreb, Croatia*
[2]*Croatian Institute for Brain Research- University of Zagreb School of Medicine, Laboratory for Molecular Immunology, Zagreb, Croatia*
[3]*University of Zagreb School of Medicine, Department of Physiology and Immunology, Zagreb, Croatia*
[4]*University of Mostar School of Medicine, Department of Physiology, Mostar, Bosnia - Herzegovina*

### Abstract Text

**Background/purpose:** Osteoarthritis (OA) is a common joint disease induced by the complex interaction of inflammation, impaired regeneration and biomechanical stress. Our research seeks to assess the inflammatory and osteochondroprogenitor populations in murine models of OA accompanied by inflammation.

**Methods:** OA was induced in 12-16 -week-old C57BL/6 male mice by two intraarticular injections of 1U collagenase type VII in 10µL of 0.1M PBS (right OA knee) or 10µL of 0.1M PBS (left control knee) separated by 48 h. Mice were sacrificed on days 3, 7, 14 and 56. Systemic inflammation was induced by subcutaneous injection of 100µL complete Freund's adjuvant. Mice were sacrificed on days 10, 20, and 30 post-injection. Knees were analyzed by µCT or flow cytometry after labelling with antibodies to main hematopoietic (CD3, CD8, CD11b, Ly6G, F4/80) and osteochondroprogenitor (CD51, CD200, CD90, CD105) markers in both of the models.

**Results:** Accumulation of myeloid and osteochondroprogenitor populations ($CD11b^+$, $Ly6G^+$, $F480^+$ and $CD200^+$, $CD90.2^+$ respectively) was determined in days 3-7 of collagenase-induced OA (CIOA). By day 14, frequencies were similar to the control group except for $CD11b^+Ly6G^+$ cells, while by day 56 no significant group differences remained. The thinning of subchondral epiphyseal bone at day 10 of the systemic inflammation model was accompanied by an increased frequency of $CD200^+$ osteochondroprogenitors, while the sclerotic changes of subchondral bone (hallmark of OA development) at day 30 post induction was accompanied by accumulation of cells of myeloid lineage ($CD11b^+$, $Ly6G^+$, $F4/80^+$).

**Conclusions:** Changes of synovial osteochondroprogenitor and inflammatory cell populations in early CIOA do not persist in the later stages. Early phases of both systemic inflammation and CIOA are marked by the accumulation of $CD200^+$ early osteochondroprogenitor populations in the synovia.

**P133**

### Antimicrobial Peptide Pt5-1c promotes Bone Mineralisation and Formation in Zebrafish Larvae

*Margherita Muscat[1], Sherif Suleiman[2], Amy Marie Vella[1], Lucienne Vassallo Gatt[3], Márton Szoboszlay[2], Zhenhui Liu[4], Zhihui Hao[5], Pierre Schembri Wismayer[2,6], Melissa M Formosa[1,6]*
*[1]University of Malta, Department of Applied Biomedical Science, Msida, Malta*
*[2]University of Malta, Department of Anatomy, Msida, Malta*
*[3]University of Malta, International School of Foundation Studies, Msida, Malta*
*[4]University of China, Department of Marine Biology- Marine Life Science College Ocean, Qingdao, China*
*[5]Beijing University of Agriculture, Institute of Animal Medicine- College of Veterinary Medicine China, Beijing, China*
*[6]University of Malta, Centre for Molecular Medicine and Biobanking, Msida, Malta*

### Abstract Text

**Introduction:** Osteoporosis, a health condition characterised by frail bones, constitutes a major global health burden as it significantly increases the risk of falls, fractures, and institutionalisation. The development of novel, safe, and effective interventions is therefore a priority.

**Purpose:** The NASDAC project (SINO-MALTA-2022-08) investigated *in vivo* effects of the antimicrobial peptide Pt5-1c on bone mineralisation using a zebrafish larval model. The aim of the study was to assess any osteogenic effects of the peptide, while also determining its toxicity profile in early-stage zebrafish larvae.

**Methods:** A modified Fish Embryo Acute Toxicity (FET) test developed in-house was conducted on zebrafish larvae aged 3 to 7 days post-fertilisation (dpf) to accommodate bone mineralisation timelines. Toxicity screening involved exposing larvae to concentrations ranging from 100 µM down to 0.5 µM Pt5-1c to establish the lethal concentration 50 ($LC_{50}$). Neurotoxicity was assessed via behavioural analysis using the DanioVision™ tracker equipped with light and sound stimuli. The degree of mineralisation was assessed by Alizarin Red staining quantified using the operculum-to-head (O/H) ratio, whereas bone formation was investigated by measuring whole body Alkaline Phosphatase (ALP) levels quantified by spectrophotometry.

**Results:** The $LC_{50}$ was confirmed to be at approximately 1 µM. Behavioural analysis revealed that larvae exposed to 0.5–1.5 µM showed no significant signs of neurotoxicity, displaying acclimatisation patterns similar to untreated (E3) and vehicle (1% DMSO) controls. Larvae treated with 1 µM Pt5-1c demonstrated increased mineralisaton in the cranium compared to controls ($p$<0.05). Furthermore, the ALP assay confirmed a significant increase in osteoblast activity and bone formation at 1 µM concentration ($p$<0.01).

**Conclusion:** The study indicates that Pt5-1c has an osteoanabolic potential without inducing neurotoxicity. These results justify further investigation, including planned RNA-sequencing to identify the signalling cascades driving the increase in bone mineralisation and formation.

**P134**

### Modulating autophagy in a zebrafish model of osteogenesis imperfecta

Carla Aresi[1], Francesca Tonelli[1], Alessandro Palma[2], Chiara De Leonibus[3], Carmine Settembre[3], Antonella Forlino[1]
[1]University of Pavia, Molecular Medicine- Biochemistry Unit, Pavia, Italy
[2]Sapienza University- Rome, Department of Phycology- Division of Neuroscience, Rome, Italy
[3]Telethon Institute of Genetics and Medicine, Tigem, Naples, Italy

### Abstract Text

Osteogenesis imperfecta (OI) is a rare bone disease mainly associated to collagen I defects. The abnormal protein is partially intracellularly retained, causing ER stress. A relevant crosstalk exists between the ER quality control machinery and a form of autophagy, known as ER-phagy that selectively disposes certain misfolded clients from the ER exists. The modulation of this pathway could indeed alleviates ER stress in OI.

The aim of the study was to investigate, through a drug-repurposing approach, the therapeutic potential of molecules identified in a library screen for autophagy modulators, in the context of OI. To achieve this, we employed *Chi/+* zebrafish, a validated model of dominant OI.

Toxicity tests allowed to determine the dose of 5 µM for Gefitinib and 2 µM for Silmitasertib (n=12/group). 10 µM was chosen for Tetrandrine, based on literature. Following 6 days of treatment the mineralization of the cranial bones stained with alizarin red was analyzed for Gefitinib and Silmitasertib wild type (WT) and *Chi/+* larvae (n>50/group), whereas the operculum mineralized area was measured following Tetrandrine administration (n=30/group). Autophagy and ER-phagy were evaluated by western blotting and qPCR (n=4/group), respectively.

Bone mineralization was reduced in 6 dpf *Chi/+* larvae compared to WT ($p<0.05$). Treatment with Gefitinib increased notochord (NC) and branchiostegal ray 3 (BS3) mineralization in *Chi/+* compared to untreated mutants ($p<0.05$). Silmitasertib significantly increased mineralization in NC, hyomandibular bone and BS3 ($p<0.05$). LC3-II was upregulated in *Chi/+* larvae. Tetrandrine significantly increased the area of mineralized operculum in treated relative to untreated *Chi/+* larvae ($p<0.05$) and upregulated fam134b, a marker of ER-phagy ($p<0.01$).

The data suggest Silmitasertib and Tetrandrine as promising candidates to improve bone mineralization by targeting ER stress in OI.

Supported by the European Union's - Next Generation EU program through the Italian PRIN 2022 - M4C2, Investimento 1.1 - grant n. 20223C8C5B - CUP F53D23006870006

**P135**

### Altered Mineral Clusters in Bone Tissue of a Zebrafish Model of Osteogenesis Imperfecta Assessed by Synchrotron X-ray Scattering and Electron Microscopy

*Imke A. K. Fiedler*[1,2,3], *Johannes Krug*[1], *Christine Plumeyer*[1], *Nico Fischer*[1], *Lucas Puebla*[1], *Tilman Grünewald*[4], *Manfred Burghammer*[4], *Katharina Jähn-Rickert*[1], *Antonella Forlino*[5], *Björn Busse*[1,3]
[1]*University Medical Center Hamburg-Eppendorf, Department of Osteology and Biomechanics, Hamburg, Germany*
[2]*University Medical Center Hamburg-Eppendorf, Electron Microscopy Core Facility, Hamburg, Germany*
[3]*University Medical Center Hamburg-Eppendorf, Interdisciplinary Competence Center for Interface Research, Hamburg, Germany*
[4]*European Synchrotron Radiation Facility, Microfocus beamline ID13, Grenoble, France*
[5]*University of Pavia, Department of Molecular Medicine, Pavia, Italy*

**Abstract Text**

**Background:** Bone formation is a tightly regulated process in which the collagen scaffold templates the nucleation and growth of mineral particles. 3D imaging studies showed that these particles assemble into mesoscale ellipsoidal mineral clusters or tesselles whose organisation likely contributes to mechanical functionality. In classical dominant osteogenesis imperfecta (OI), a collagen type I disorder, skeletal fragility occurs despite often increased tissue mineralization. Current therapies primarily target bone turnover, yet clinical responses vary, and the nanoscale determinants of treatment efficacy remain poorly understood. For example, whether mineral cluster formation is altered in OI and how this may influence therapeutic responsiveness remains unclear.
**Purpose:** This study investigates how mineral particle geometry, cluster formation, and their organisation are affected in a collagen-defective bone matrix.
**Methods:** Vertebral bone of adult *Chihuahua* (Chi/+) zebrafish, a model of dominant OI characterized by excessive osteoid deposition, collagen defects, and hypermineralized yet mechanically fragile bone, was examined. Vertebrae from WT and *Chi/+* fish (N=3 per group) were analyzed using synchrotron small- and wide-angle X-ray scattering to quantify mineral particle geometry, and focused ion beam–scanning electron microscopy to visualize mineral organisation.
**Results:** *Chi/+* bone showed thinner mineral particles than WT (2.4±0.3nm vs. 3.1±0.4nm, p=0.04), while crystal length was similar (8.8±1.1nm vs. 8.8±1.0nm). Ellipsoidal mineral clusters/tesselles were found in bone-forming sites of both groups. Chi/+ appeared with higher ellipsoid packing density and fewer non-mineralized interspaces compared to WT.
**Conclusions:** These findings support that collagen defects in OI disrupt the hierarchical pathways through which mineral particles assemble into mechanically competent tissue. Thinner mineral particles within a hypermineralized and disorganized matrix may underlie the reduced tissue-level stiffness previously reported in *Chi/+* bone. This work highlights the zebrafish as a translational model for probing how candidate drugs could influence nano- and mesoscale mineral organisation and for identifying structural biomarkers that could predict treatment responsiveness in OI.

**P136**

**Protective effects of rFVIII prophylaxis: lower osteoclast activity and prevention of cortical changes in haemophilia A mice**

*Joyce Kimenai*[1,2,3], *Lara Monica*[4,5], *Olga Oleshko*[5], *Asma Mechakra*[2,3], *Sonja Werwitzke*[6], *Lina Aires*[7], *Silvio Wuschko*[8], *Helmut Paul*[8], *Paul Schauerte*[9], *Jonas Füner*[4], *Larisa Belyanskaya*[7], *Enrico Klotzsch*[10], *Jess. G. Snedeker*[2,3], *Andreas Tiede*[5,6], *Sander Botter*[1]

[1]*Balgrist Campus AG, Swiss Center for Musculoskeletal Biobanking, Zurich, Switzerland*

[2]*Balgrist University Hospital Zurich- University of Zurich, Department of Orthopedics, Zurich, Switzerland*

[3]*ETH Zurich, Department of Health Science and Technology, Zurich, Switzerland*

[4]*preclinics GmbH, none, Potsdam, Germany*

[5]*Hannover Medical School, Hematology- Hemostasis- Oncology- and Stem Cell Transplantation, Hannover, Germany*

[6]*Hannover Medical School, Institute of Clinical Chemistry and Central Laboratory, Hannover, Germany*

[7]*Octapharma AG, none, Lachen, Switzerland*

[8]*Octapharma Pharmazeutika Produktonsges m.B.H., none, Vienna, Austria*

[9]*Taconic Biosciences GmbH, none, Leverkusen, Germany*

[10]*Berlin Institute of Health BIH at Charité – Universitätsmedizin Berlin-,*
*BIH Center for Regenerative Therapies BCRT, Berlin, Germany*

**Abstract Text**

**Background:** Knee joint arthropathy is a frequent complication in patients with haemophilia A (HA), driven by recurrent intra-articular bleeding. Such bleeding can be prevented by recombinant FVIII (rFVIII) prophylaxis, but currently, no mouse model reproduces clinical prophylaxis or associated bone changes.

**Aim:** The goal of this study was to assess changes in the cortex of the proximal tibia, together with osteoclast presence with different treatment regimens of rFVIII in an HA mouse-model lacking inhibitor response.

**Materials and Methods:** FVIII$^{-/-}$ Ighm$^{-/-}$ knockout mice were injured in their left knee joint, using the contralateral leg as internal control. Mice were either non-treated, treated with a single dose of rFVIII prior to needle puncture, or a rFVIII secondary prophylaxis daily on day 1-4 and then 3 times per week. Mice were sacrificed on day 4, 11 or 21, and knee joints were isolated and scanned using µCT, measuring cortical thickness. Osteoclast presence was semi-quantified using tartrate-resistant phosphatase staining in histological sections (0 = no osteoclasts +, osteoclasts, ++, many osteoclasts were found). Statistical analyses included two-way ANOVA, logistic regression, and ordinal regression with Holm correction.

**Results:** On day 21, mice receiving rFVIII prophylaxis showed a stable cortical thickness ratio between the left and right tibia, while untreated mice showed an increased ratio on day 21 (Figure 1A). The rFVIII prophylaxis group shows lower osteoclast presence compared to the no-treatment group. This significant difference is mainly driven by day 4 (Figure 1B). A similar trend towards lower aberrant bone formation in the rFVIII prophylaxis group was found (Figure 1C), although not statistically significant.

**Discussion & Conclusion:** Altogether, these data show that rFVIII prophylaxis may help prevent cortical changes and increased bone turnover, as indicated by a stable cortical thickness, fewer osteoclasts and a trend towards limited formation of aberrant bone.

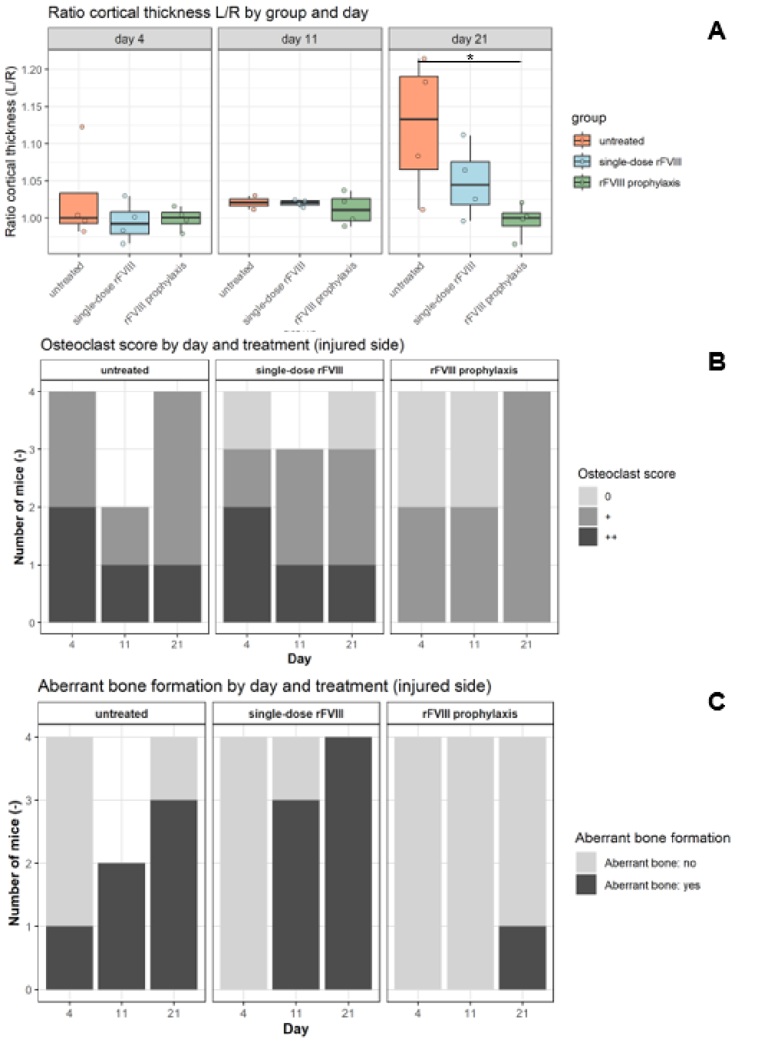

*Figure 1. Cortical thickness ratio, osteoclast formation, and aberrant bone formation after knee-injury in haemophilia A mice under different treatment regimens. A: Cortical thickness ratio. On day 21 the cortical thickness ratio is lower in the group with rFVIII prophylaxis compared to untreated mice. B: Osteoclast presence in the cortex in the different treatment groups. The rFVIII prophylaxis group has significantly lower osteoclasts compared to untreated mice. C: Incidence of aberrant bone formation in each treatment group.*

### Poster Sessions: Muscle, physical activity and bone

### P137

### Fall Risk, Cognitive Impairment, and All-Cause Mortality among Chinese Nursing Home Residents: A Whole-Population Retrospective Cohort Study

*Yuan Yuan[1], Ping-Yang Li[2], Wei Wang[1], Yan-Na Lu[3], Ling Wang[4], Shujing Lou[5], Zi Jie[6], Tian Wei[1]*

*[1]Beijing Jishuitan Hospital- Capital Medical University, Department of Geriatrics, Beijing, China*

*[2]Peking University Health Science Center, None, Beijing, China*

*[3]Beijing Jishuitan Hospital- Capital Medical University, Geriatrics, Beijing, China*

[4]Beijing Jishuitan Hospital- Capital Medical University, Department of Radiology, Beijing, China
[5]Longzeyuan Community Health Service Center, Department of General Practice, Beijing, China
[6]Beijing Taikang Yanyuan Rehabilitation Hospital, Medical Affairs Department, Beijing, China

**Abstract Text**

**Background:** Falls and cognitive impairment are major geriatric syndromes that often coexist and worsen functional decline and mortality in institutionalized older adults. However, data from Chinese nursing homes remain limited.

**Purpose:** To examine the associations among MMSE-defined cognitive status, fall incidence, and all-cause mortality in a whole-population cohort of Chinese nursing-home residents.

**Methods:** This retrospective cohort study included 1,935 residents (mean age 82.7 ± 7.7 years; 55.2% female) from an urban Beijing nursing home who completed the Mini-Mental State Examination (MMSE). Participants were classified as no cognitive impairment (NCI, 27–30), mild (MCI, 21–26), moderate (MOCI, 10–20), or severe cognitive impairment (SCI, 0–9). Fall events and mortality during follow-up were retrieved from electronic health records. Logistic regression identified independent predictors of falls and mortality.

**Results:** Overall, 314 residents (16.2%) experienced at least one fall, showing an inverted U-shaped pattern across cognitive strata—highest in MCI (24.7%), followed by MOCI (22.7%), NCI (14.0%), and SCI (11.9%). Common fall predictors included multimorbidity with polypharmacy, lower Barthel Index, higher PHQ-9/GAD-7 scores, and reduced social support. Stratum-specific factors were advanced age and osteoporosis (NCI), female sex and low prealbumin (MCI), cerebrovascular disease and high cystatin C (MOCI), and low BMI and elevated $HbA_{1}c$ (SCI). During follow-up, 206 deaths (10.6%) occurred; living alone and cognitive impairment increased mortality, while higher BMI and hemoglobin, female sex, and cardiovascular drug use were protective.

**Conclusions:** Fall risk peaked in mild cognitive impairment, forming an inverted U-shaped curve across cognitive strata. These findings support stratified, multidomain fall-prevention and mortality-reduction strategies that integrate cognitive, functional, psychosocial, and nutritional assessments in nursing-home residents.

**P138**

**Beyond body mass index: Appendicular lean mass index–defined sarcopenia and carpal tunnel syndrome — a cross-sectional analysis**

*Hyun Sik Gong*[1], *Sung Ha Chun*[1]
[1]*Seoul National University Bundang Hospital, Orthopedic Surgery, Seongnam, Korea- South*

**Abstract Text**

**Introduction/Aims:** Carpal tunnel syndrome (CTS) is the most common peripheral entrapment neuropathy. Despite consistent associations with obesity, the role of muscle loss remains unclear. Sarcopenia may increase nerve vulnerability to compression. We examined whether appendicular lean mass index (ALMI) and ALMI-defined sarcopenia are associated with CTS independent of body mass index (BMI).

**Methods:** We retrospectively analyzed 193 consecutive patients aged 50 years or older who underwent carpal tunnel release by a single surgeon at a tertiary center over four years. Controls were 5,665 age-matched participants from the 2022-2023 Korea National Health and Nutrition Examination Survey. BMI and sarcopenia defined by sex-specific ALMI thresholds were compared between groups. Multivariable logistic regression was applied to examine independent associations with CTS.

**Results:** CTS patients showed significantly higher BMI in both men ($P = 0.02$) and women ($P < 0.001$), and higher prevalence of sarcopenia in men ($P < 0.001$) and women ($P < 0.001$) compared with controls. Obesity was more evident in younger patients of both sexes, particularly those younger than 70 years, whereas sarcopenia predominated across all ages in women and in men older than 60 years. In multivariable models, BMI (OR 1.05, 95% CI 1.01–1.10) and sarcopenia (OR 2.41, 95% CI 1.79–3.22) were each independently associated with CTS.

**Conclusions:** Both obesity and ALMI-based sarcopenia were associated with CTS, with sarcopenia showing a stronger association. Our results support consideration of ALMI-defined sarcopenia as a complementary metric to BMI in CTS risk assessment; causality remains to be established.

**P139**

**Age- and Sex-Specific Patterns of Bone-Muscle Coupling: Relative Contributions of Muscle Volume versus Quality to Bone Mineral Density in UK Biobank**

*Hyeon Su Kim[1], Dong Won Byun[2], Deog-Yoon Kim[3], Seong Bin Hong[4], Jun-Il Yoo[5]*
*[1]Inha University,*
*Department of Biomedical Science- College of Medicine- Program in Biomedical Science & Engineering - Inha University- Department of Biomedical Research Institute- Inha University Hospital- Incheon- South Korea, Incheon, Korea- South*
*[2]Soonchunhyang University Hospital,*
*Division of Endocrinology and Metabolism- Department of Internal Medicine- Soonchunhyang University Hospital- Soonchunhyang University College of Medicine- Seoul- Republic of Korea, Seoul, Korea- South*
*[3]Kyung Hee University Hospital,*
*Department of Nuclear Medicine- Kyung Hee University Hospital- Kyung Hee University School of Medicine- Seoul- South Korea, Seoul, Korea- South*
*[4]Inha University Hospital,*
*Division of Endocrinology and Metabolism- Department of Internal Medicine- Inha University Hospital - Inha University College of Medicine- Incheon- Korea, Incheon, Korea- South*
*[5]Inha University Hospital, Orhopedics Surgery, Incheon, Korea- South*

**Abstract Text**

**Background/Introduction:** The bone–muscle unit operates as an integrated biomechanical system, with muscle forces a key determinant of bone mass. Although muscle quantity is consistently related to bone mineral density (BMD), the relative importance of muscle quality, particularly intramuscular fat, across age and sex remains unclear.

**Purpose:** To characterize age- and sex-specific patterns of bone–muscle coupling in a large UK Biobank cohort, directly comparing the contributions of thigh muscle volume versus muscle quality to hip BMD.

**Methods:** We analyzed 33,659 UK Biobank participants aged 40–82 years with paired hip DXA and thigh MRI. Total hip BMD ($g/cm^2$) was measured by DXA. Thigh muscle volume (fat-free muscle volume, FFMV) was derived from MRI, and muscle quality was proxied by DXA leg tissue fat percentage (higher values indicating poorer quality). Sex- and age-stratified multiple linear regression models with z score–standardized predictors estimated associations with BMD, adjusted for anthropometric and lifestyle factors. Relative dominance was defined as $\beta\_volume - |\beta\_quality|$.

**Results:** Adding muscle volume to the base model increased $R^2$ from 0.326 to 0.344, whereas adding muscle quality increased $R^2$ to 0.345; the combined model yielded $R^2 = 0.348$. In women, β_volume exceeded |β_quality| in all age groups (0.81 vs 0.55 in 40s; 0.32 vs 0.17 in ≥70). In men, β_volume and |β_quality| were similar (0.53 vs 0.50; 0.30 vs 0.29), with dominance values near zero. For both sexes, associations between BMD and both volume and quality weakened with age, without a shift toward quality dominance.

**Conclusion(s):** Bone–muscle coupling was sex-specific: thigh muscle volume was a stronger determinant of hip BMD than muscle quality in women, whereas both contributed similarly in men. The hypothesis that muscle quality becomes relatively more important with age was not supported, suggesting sex-specific priorities for intervention to preserve bone health.

**P140**

**Elevated Serum CXCL9 Levels Are Associated with Greater Frailty Risk in Older Women**

*<u>Seongbin Hong</u>[1], Da Hea Seo[2], Nancy Lane[3], Beom-Jun Kim[4]*
*[1]Inha University School of Medicine, Endocrinology, Incheon, Korea- South*
*[2]Inha University  School of Medicine, Endocrinology, Incheon, Korea- South*
*[3]University of California-, Reumatology, Sacramento, United States*
*[4]Asan Medical Center- University of Ulsan College of Medicine, Endocrinology, Seoul, Korea- South*

**Abstract Text**

**Background:** Frailty is a clinical syndrome that increases the vulnerability to adverse outcomes in older adults. CXC motif chemokine ligand 9 (CXCL9), a marker of chronic inflammation, has been implicated in musculoskeletal decline and mortality. However, its relationship with frailty in older individuals has not been well studied.

**Methods**: This study included 237 community-dwelling women, aged 65 years or older, who underwent a comprehensive geriatric assessment. Serum CXCL9 levels were measured by ELISA. Frailty was defined using the Fried phenotype and the Rockwood deficit accumulation index. Associations between serum levels CXCL9 and frailty outcomes were examined using a multivariate regression analysis.

**Results:** Serum CXCL9 increased progressively across the frailty categories (robust: 239.3 ± 110.1 pg/mL; frail: 347.5 ± 119.0 pg/mL; $P < 0.001$). Higher levels of CXCL9 were independently associated with a greater frailty index ($\beta = 0.0001$, $P = 0.004$), lower skeletal muscle index ($\beta = -0.001$, $P = 0.002$), weaker grip strength ($\beta = -0.010$, $P < 0.001$), and slower gait speed ($\beta = -0.0003$, $P = 0.027$). Women in the highest quartile had 4.53-fold greater odds of frailty [95% confidence interval (CI) 1.02–20.18] and 6.93-fold greater odds of lower muscle strength (95% CI 1.81–26.57) compared with the lowest quartile.

**Conclusions:** Elevated CXCL9 levels were independently associated with frailty and impaired musculoskeletal function in older women. These findings support CXCL9 as a promising biomarker of chronic inflammation and frailty, and warrant further investigation through longitudinal studies.

**P141**

### Molecular pathways linking muscle senescence to bone health: insights from an in vitro model

*Amir Mohammad Malvandi[1], Laura Gerosa[2], Maria Emanuela Orlando[3], Chiara Verdelli[1], Valerio Leoni[4], Giuseppe Banfi[5,6], <u>Giovanni Lombardi</u>[3,7]*

*[1]IRCCS Ospedale Galeazzi-Sant'Ambrogio, Division of Experimental Biochemistry and Advanced Diagnostics, Milano, Italy*

*[2]IRCCS Ospedale Galeazzi-Sant'Ambrogio, Division of Experimental Biochemistry & Advanced Diagnostics, Milano, Italy*

*[3]Poznan University of Physical Education, Department of Athletics- Strength and Conditioning, Poznan, Poland*

*[4]University of Milano-Bicocca- Azienda Socio Sanitaria Territoriale Della Brianza- ASST-Brianza- Desio Hospital, Department of Laboratory Medicine, Desio, Italy*

*[5]IRCCS Ospedale Galeazzi-Sant'Ambrogio, Scientific Director, Milano, Italy*

*[6]Vita-Salute San Raffaele University, Faculty of Medicine and Surgery, Milano, Italy*

*[7]IRCCS Ospedale Galeazzi-Sant'Ambrogio, Divisione of Experimental Biochemistry and Advanced Diagnostics, Milano, Italy*

### Abstract Text

Aging leads to the accumulation of senescent cells, which majorly contribute to tissue decline, including skeletal muscle. Muscle homeostasis relies on coordinated networks for regeneration, metabolism, and communication; disruptions can cause widespread dysfunction. Though interest in aging biology is growing, the exact molecular mechanisms linking senescence to muscle deterioration remain poorly understood. This study aimed to evaluate the functional molecular mechanisms underlying our *in vitro* murine model of muscle senescence, identifying key mechanisms involved in senescence induction and their consequent influence on muscle-osteoblast cross-talk. We performed global molecular profiling using high-resolution mass spectrometry (HRMS) to identify pivotal molecular trends in senescent myoblasts (SMs). Key findings include significant shifts in eicosapentaenoic acid (EPA) metabolism, mitochondrial functional dynamics, and different fatty acid metabolic pathways. Reduced mitochondrial fission 1 protein levels and altered mitochondrial morphology, as confirmed by western blotting and immunofluorescence analyses, support mitochondrial dynamics impairment in SMs. This was confirmed by a significant increase in Acetyl-CoA levels in SMs observed by LC-MS. Further quantitative target analysis using the GS-MS revealed significant changes in very long fatty acids (VLFAs) and cholesterol metabolism. A significant reduction in many VLFAs, with increased C24 and C26 fatty acids and oxysterol markers (7bOHC and 7KC), suggests peroxisome activation. Secreted pro-inflammatory chemokines such as CCL4, CCL5, and CCL19 were significantly increased from SMs. Long-term exposure to conditioned medium (CM) from SMs differentially altered the expression of Col1α1, Spp1, and Alp in MC3T3-E1 during differentiation. Interestingly, CM-treated mature osteoblasts showed increased phosphorylation of H2aX. This study shows muscle senescence involves changes in lipid metabolism, mitochondrial dynamics, and inflammation, altering metabolic balance to increase acetyl-CoA and pro-senescence signals in SMs. The secretome impacts osteoblast differentiation and genomic stability, revealing a new link between senescent muscle cells and bone function.

**P142**

**Sarcopenia in transfusion-dependent thalassemia: evidence of accelerated musculoskeletal aging.**

*Ekaterini Zigoura*[1], *Wanda Morganti*[1], *Emanuele Seminerio*[1], *Martina Vigo*[1], *Annarosa Floris*[1], *Manuela Balocco*[2]
[1]*E.O. Galliera Hospital, Geriatrics Unit, Genoa, Italy*
[2]*E.O. Galliera Hospital, Microcythemia- congenital anemias and iron dysmetabolism unit, Genoa, Italy*

**Abstract Text**

**Introduction:** Sarcopenia is a complex deterioration of muscle mass and function, typically associated with aging. However, its prevalence in younger populations, particularly those with chronic diseases, remains poorly documented. The effect of iron overload on osteoporosis is well-documented, and ferroptosis can impact muscle deleteriously. Thus, individuals with Transfusion-Dependent Thalassemia (TDT) may be at increased risk.

**Purpose:** Assessing the prevalence of sarcopenia in TDT patients referring to the Galliera Hospital in Genoa (Italy).

**Methods:** This retrospective observational study included patients with major/intermediate β-thalassemia. Demographic data and nutritional status (Mini-Nutritional Assessment, MNA) were collected. Participants underwent DEXA to measure femoral neck and lumbar spine (L1–L4) bone mineral density (BMD), and muscle mass with the Relative Skeletal Muscle Index (RSMI). Muscle strength was assessed with Hand Grip (HG). Dynapenia was defined as HG <16 kg in females and <27

kg in males; sarcopenia as RSMI <5.5 kg/m² in females and <7 kg/m² in males. Osteoporosis was classified by a BMD T-score <–2.5.

**Results:** The sample included 87 participants (mean age 46.5±12.72; 35.6% males). While age did not differ between genders, significant differences were found in HG (Females:13.2±2.85 Vs. Males:21.9±8.7; p<.001), RSMI (Females:5.77±0.95 Vs. Males:6.89±0.99; p<.001), and MNA scores (Females:25.1±2.9 Vs. Males:26.6±2.7; p=0.028). Dynapenia was found in 65/87 patients (74.7%), and sarcopenia in 30/87 (34.5%). Osteoporosis was identified in 49/87 patients (56.3%) based on either femoral neck or lumbar spine BMD. Eighteen patients (20.7%) had both sarcopenia and osteoporosis.

**Conclusions:** These findings indicate that patients with thalassemia represent a population at increased risk for early-onset muscle and bone impairment. The presence of sarcopenia and osteoporosis at substantially younger ages than expected in the general population suggests an accelerated aging process in individuals with TDT.

**P143**

### Effect of combined treatment of romosozumab and whole body vibration on muscle tissue in an ovariectomized rat model

*<u>Marina Komrakova</u>[1], Larissa J. Helm[1], Wolfgang Lehmann[1], Kai O. Böker[1], Arndt F. Schilling[1], Daniel B. Hoffmann[1]*
*[1]University Medical Center Goettingen, Trauma Surgery- Orthopaedics and Plastic Surgery, Goettingen, Germany*

**Abstract Text**

**Introduction:** Romosozumab (Romo), an anti-sclerostin antibody, is approved for postmenopausal osteoporosis. Besides its skeletal effects, sclerostin is also expressed in muscle, highlighting skeletal muscle as a new source of sclerostin. Whole body vibration (Vib) enhances musculoskeletal function and can reduce circulating sclerostin levels. However, the effect of scletostin inhibition on muscle tissue has not been studied so far and the interaction between Romo and Vib on muscle tissue remains unclear.

**Purpose:** This study aimed to investigate the effects of combined Romo and Vib treatments on muscle tissue in ovariectomized rats.

**Methods:** The animal study was approved prior to study. Six-month-old female rats were ovariectomized (Ovx) or sham operated (Sham). After 8 weeks, animals were assigned to groups (n=15/group): Sham, Ovx, Ovx+Romo (25 mg/kg/week, s.c.), Ovx+Vib (vertical, 30 Hz, 2×/day, 15 min, 7×/week), and Ovx+Romo+Vib. Physiological saline was injected to Sham, Ovx, and Vib groups. After 8 weeks, M. gastrocnemius, M. soleus, and M. longissimus were assessed using histological and gene expression analyses. Muscle sclerostin protein was quantified by ELISA.

**Results:** Ovx increased fiber area, while Romo reduced fiber size, and Vib and Romo+Vib treatments prevented Ovx-induced enlargement without altering overall muscle structure. Sclerostin gene expression was the lowest in the Romo+Vib group. IGF-1 and myostatin were upregulated with combination therapy; IGF-1 was also elevated in Ovx+Vib rats. Romo markedly increased sclerostin protein in serum, muscle, brain, and bone (200×, 160×, 16×, and 4×, respectively) without ELISA cross-reactivity.

**Conclusion:** Romo exerted local and systemic effects on muscle via sclerostin inhibition, while Vib confirmed the positive impact of physical activity on muscle morphology and growth factor expression. Combined Romo+Vib treatment showed no additive morphological benefit over single interventions, but induced distinct changes in sclerostin gene expression and growth-regulating factors. Further molecular and functional studies are warranted to elucidate underlying mechanisms of Romo action on muscle.

**P144**

**Myostatin as a Potential Biomarker for Sarcopenia and Age-Related Muscle Loss in Clinical Practice – Associations with Body Composition Data and Demographic Factors**

*Olivia Trummer[1], Felix Krasser[1], Ines Foessl[2], Barbara Obermayer-Pietsch[1]*
*[1]Medical University Graz, Internal Medicine- Division of Endocrinology and Diabetology, Graz, Austria*
*[2]Medical University Graz and JOANNEUM RESEARCH Forschungsgesellschaft mbH,*
*Dept. of Internal Medicine- Division of Endocrinology and Diabetology and COREMED-*
*Centre for Regenerative and Precision Medicine, Graz, Austria*

**Abstract Text**

**Background and Aim:** Myostatin, predominantly secreted by skeletal muscle, is a member of the TGF-beta superfamily. In huma cell culture studies it suppresses myogenesis while promoting adipogenesis and insulin resistance (Amor et al. 2018, Guo et al. 2008). Aim of the study was to investigate myostatin, a potential biomarker for sarcopenia and age-related muscle loss, in relation to demographic factors as well as body composition.

**Methods:** The BioPersMed longitudinal study recruited 1022 individuals aged ≥45 years with at least one cardiovascular risk factor between 2010 and 2016. In 215 participants (128 women [59.5%], 87 men [40.5%]), serum myostatin levels were measured using ELISA (R&D Systems Europe, Ltd.) and body composition was assessed using DXA (Lunar iDXA, GE HealthCare, USA). The study population was divided into groups based on sex, BMI, age, and appendicular skeletal muscle mass index (ASMMi). Statistical analysis was performed using t-test/Mann-Whitney U-test and ANOVA/Kruskal-Wallis test, respectively (R_Version_4.2.1 (2022 The R Foundation for Statistical Computing)).

**Results:** Men showed higher myostatin levels compared to women (875.0 pg/mL ± 407.7 vs. 756.8 pg/mL ± 346.3, $p<0.01$). BMI groups showed differences in myostatin levels (normal weight (747.3 pg/mL ± 343.2), overweight (821.8 pg/mL ± 381.95), obesity (887.1 pg/mL ± 361.3; $p=0.02$). Furthermore, there was a trend toward lower myostatin levels in participants with reduced ASMMi. Myostatin levels did not differ by age.

**Conclusion:** Men, individuals with obesity, and individuals with normal ASMMi showed higher myostatin levels. This may reflect greater muscle mass in these individuals. Further investigations, including analysis of groups using the ASMMi/myostatin-ratio instead of absolute myostatin values, are planned with an expanded study population.

**P146**

**Age-related sarcopenia regulates bone remodelling through mechanisms beyond mechanical stimulation: Evidence from PAK deficient mice**

*Florence Figeac*[1], *Lisbeth L.V. Møller*[2], *Atenisa Caci*[1], *Racha Yassine*[1], *Dina Awad Mohammad*[1], *Moemen Mohamed Hussein*[3,4], *Paula Fernandez Guerra*[1], *Hanna Taipaleenmäki*[5], *Abbas Jafari*[6], *Haralampos Hatzikirou*[7,8], *Alexander Rauch*[1], *Erik A. Richter*[9], *Moustapha Kassem*[1,10]
[1]*Odense University Hospital, Molecular Endocrinology Department- KMEB, Odense, Denmark*
[2]*University of Copenhagen, Department of Biomedical Sciences- Faculty of Health and Medical Sciences, Copenhagen, Denmark*
[3]*Khalifa University, Biomedical Engineering and Biotechnology Department, Abu Dhabi, United Arab Emirates*
[4]*Alexandria University, Department of Mathematical Engineering and Physics- Faculty of Engineering, Alexandria, Egypt*
[5]*LMU University Hospital, Institute of Musculoskeletal Medicine- Musculoskeletal University Center Munich, LMU Munich, Germany*
[6]*University of Copenhagen, Department of Cellular and Molecular Medicine- ICMM, Copenhagen, Denmark*
[7]*Khalifa University of Science and Technology, Department of Mathematics, Abu Dhabi, United Arab Emirates*
[8]*Technische Universitat Dresden, Center for Information Services and High Performance Computing, Dresden, Germany*
[9]*University of Copenhagen, Section of Human and Molecular Physiology- Department of Nutrition- Exercise and Sports- Faculty of Science, Copenhagen, Denmark*
[10]*Khalifa University, Faculty of Medicine and Health Sciences, Abu Dhabi, United Arab Emirates*

**Abstract Text**

Traditionally, muscle-bone interactions were conceived via mechanical stimulation as demonstrated by immobilization models. However, the role of muscle-related mechanisms on bone remodelling is not well defined.
Thus, we employed muscle-specific Group I p21-activated kinases (PAKs) deficient mice (mPAK$^{-/-}$) that exhibit age-related myopathy and thus a model for human age-related sarcopenia to investigate those mechanisms.
MicroCT analysis revealed significant bone loss in mPAK$^{-/-}$, with a 44.8% reduction in trabecular bone mass (p<0.01), as well as a 16.2% decrease in cortical thickness (p<0.01), compared to controls, while they did not exhibit significant changes in whole body energy metabolism. Bone histomorphometry showed a significant decrease in bone formation (with a 51% and 57% reduction in respectively mineral apposition rate and bone formation rate per bone surface p<0.05), with unchanged bone resorption. To determine underlying mechanisms, RNAseq analysis of mPAK$^{-/-}$ bones revealed a senescent profile accompanied by a significant downregulation of genes associated with osteoblast differentiation and functions, as well as energy metabolism pathways like glycogen metabolism, gluconeogenesis and TCA cycle associated with downregulation of mitochondria-related genes e.g. *Sdhb*, *Mpc2*, *Idh3b*. Interestingly, osteocytes gene signature did not change between the groups. Finally, integrative data analysis showed enrichment of genes related to bone biology and immune regulation, but also enrichment of genes related to several muscle development and differentiation pathways, highlighting the influence of those genes on the detrimental bone phenotypes observed in mPAK-/- mice.
Age-related myopathy in PAK-deficient mice leads to marked bone loss independent of mechanical signaling, suggesting that muscle-derived factors are key drivers of bone deterioration during aging.

**P147**

**A multicomponent intervention consisting of exercise, proteins and omega-3 supplementation to improve sarcopenia in community-dwelling older adults: lessons learned from the ENHANCe-trial.**

*Jolan Dupont*[1], *Nadjia Amini*[2], *Laurence Lapauw*[2], *Laura Vercauteren*[2], *Sabine Verschueren*[3], *Jos Tournoy*[1], *Evelien Gielen*[1]
[1]*UZ Leuven & KU Leuven- Belgium, Geriatrics & Gerontology, Leuven, Belgium*
[2]*KU Leuven, Geriatrics & Gerontology, Leuven, Belgium*
[3]*KU leuven, Rehabilitation Sciences, Leuven, Belgium*

**Abstract Text**

**Background:** Anabolic interventions, including physical exercise, proteins and omega-3 polyunsaturated fatty acids supplementation, have shown effectiveness in improving sarcopenia outcomes. However, data on their combined effects in older adults with sarcopenia remain limited.

**Purpose**: To assess feasibility, acceptability, and preliminary effects of a multicomponent intervention combining individualized home-based exercise, proteins, and/or omega-3 supplementation in community-dwelling older adults (≥65y) with sarcopenia.

**Methods**: A 5-armed randomized controlled feasibility trial was conducted : the Exercise and Nutrition for Healthy AgeiNg (ENHANce,NCT03649698) study. Participants were randomized into 5 groups: 1)Exercise only, 2)Protein only, 3)Exercise+Protein, 4)Exercise+Protein+Omega-3, and 5)Control group. Trial feasibility was evaluated by measuring eligibility, recruitment, retention and data completion rate. Acceptability was evaluated by participants' feedback. Potential intervention effects were evaluated by measuring change in sarcopenia outcomes after 12 weeks of intervention.

**Results**: Fifty-eight participants (76.2±6.6years,♀:65.5%) were included (Exercise,n=9;Protein,n=12; Exercise+Protein,n=13;Exercise+Protein+Omega-3;n=12;Control,n=12). Feasibility was low, with a recruitment rate of 2%. Acceptability was moderate, with most participants completing the planned assessments and reporting positive experiences such as feeling stronger and more aware of the importance of physical activity and nutrition. However, many found the study procedures demanding, and many experienced difficulties with the protein supplements. Adherence varied widely across interventions. Safety was high, with no significant adverse effects reported. The interventions showed potential to improve chair stand test (CST), Short Physical Performance Battery (SPPB), muscle mass and quadriceps strength.

**Conclusions**: A multicomponent intervention to treat sarcopenia showed low feasibility, moderate acceptability, and high safety. Preliminary efficacy results showed that exercise with protein supplementation may improve physical function. Adding omega-3 PUFA could offer further benefits for muscle strength and mass, however future large-scale trials are needed to confirm these findings. The insights and the practical challenges in the ENHANce study inform future sarcopenia intervention designs.

**ND-P016**

**Effect of the 6° head-down BED REST model on myokines and osteokines: focus on the interplay between irisin and sclerostin**

*Onofrio Daniele Terrevoli*[1], *Roberta Zerlotin*[1], *Clelia Suriano*[2], *Patrizia Pignataro*[2], *Luisa Pellicani*[2], *Michela Taurino*[1], *Manuela Dicarlo*[1], *Angela Oranger*[1], *Francesca Ferranti*[3], *Bostjan Simunic*[4], *Rado Pisot*[4], *Marco Narici*[5], *Silvia Colucci*[2], *Graziana Colaianni*[1], *Maria Grano*[1]
[1]*University of Bari, Department of Precision and Regenerative Medicine and Ionian Area, Bari, Italy*
[2]*University of Bari, Department of Translational Biomedicine and Neuroscience, Bari, Italy*
[3]*Italian Space Agency, Human Flight and Scientific Experiments Office, Roma, Italy*
[4]*Institute of Kinesiology Research, Science and Research Centre, Koper, Slovenia*
[5]*University of Padova, Department of Biomedical Sciences, Padova, Italy*

**Abstract Text**

**Background:** Irisin, a myokine produced by skeletal muscle during physical activity, is widely recognized for its pleiotropic effects and, in particular, for its anabolic action on the musculoskeletal system. In our previous human BED REST (BR) study, we showed that 10-day horizontal BR reduced irisin and increased sclerostin serum levels, suggesting an interaction between these two molecules as a coordinated mechanism by which muscle and bone respond to the absence of mechanical load.

**Purpose:** Based on these results, in the SCIENCE FOR BED REST campaign — organized in 2025 by the Italian Space Agency in partnership with the Science and Research Centre Koper and the General Hospital of Izola — we aimed to identify the FNDC5/irisin system as a biomarker of musculoskeletal changes in a 6° head-down BR model that simulates the shift of fluids to the upper body experienced by astronauts during space flights.

**Methods:** This study was based on a 21-day BR protocol, followed by a 21-day retraining period to assess recovery of the musculoskeletal system.

**Results:** Our results showed that serum irisin levels decreased significantly 2 days after the end of BR (R+2) ($p=0.025$), consistent with our findings in the previous 10-day horizontal BR study. Importantly, irisin returned to baseline (BDC) levels after 21 days of retraining (R+21), indicating a complete recovery of its synthesis. Concurrently, serum sclerostin levels increased at BR10 ($p=0.024$), BR15

(*p=0.004*), and BR21 (*p=0.012*) and 2 days after the end of BR (R+2) (*p=0.04*), compared with BDC. Interestingly, following the retraining period, sclerostin returned to baseline levels, indicating that its disuse-induced increase can be reversed by a 21-day reloading period.

**Conclusion:** These findings suggest that in the BR model, irisin and sclerostin interact in a negative feedback loop, as a pathophysiological mechanism to respond in a coordinated manner to the unloading/reloading state of the musculoskeletal system.

**Poster Sessions: Osteoporosis: evaluation and imaging**

**P149**

**Impact of Bariatric Surgery on Musculoskeletal Health After Age 60**

*Line Abdulghani[1], Julien Paccou[1], Cécile Philippoteaux[1], Robert Caiazzo[2], Hélène Verkindt[3], Laurine Cadart[4]*
*[1]Lille University Hospital, Rheumatology, Lille, France*
*[2]Lille University Hospital, Endocrine Surgery, Lille, France*
*[3]Lille University Hospital, Nutrition, Lille, France*
*[4]Lille University Hospital, Biostatistics Department, Lille, France*

**Abstract Text**

**Introduction:** After the age of 60, the risk of postoperative complications following bariatric surgery is high.However, specific data regarding the bone impact in this vulnerable population are lacking. The 2022 recommendations of the ECTS suggest initiating anti-osteoporotic medication (AOM)in case of a T-score ≤ -2 and/or in case of a fragility fracture within less than 2 years.Therefore, our study evaluated the prevalence of eligibility for AOM according to age(< 60 vs. ≥ 60 years)in a cohort of postmenopausal women, as well as other associated risk factors.
**Materials and Methods:** Observational, retrospective, monocentric study conducted at Lille University Hospital evaluated the prevalence of and risk factors for eligibility for AOM according to the 2022 ECTS criteria in postmenopausal women referred to rheumatology for bone health assessment before or after bariatric surgery.
**Results:** Among the 306 postmenopausal women included, 173 were aged < 60 years(group 1) and 133 were aged ≥ 60 years(group 2).Indications for AOM were found in 69 (22.5 %)patients.This prevalence was significantly higher in women in Group 2 (30.1% vs. 16.8%, p = 0.006). In multivariate analysis, risk factors independently associated with eligibility for AOM were age ≥ 60 years(OR= 2.34, 95% CI: 1.19–4.61),active smoking(OR = 2.98, 95% CI: 1.27–7.02),reduced appendicular lean mass/height$^2$ (< 5.5 kg/m$^2$)(OR = 3.72, 95% CI: 1.78–7.78), and elevated iPTH (OR = 1,15, 95% CI 1,05-1,36 for each increase of 10 ng/ml).
**Conclusion:** One in five postmenopausal women was eligible for AOM as part of their care pathway either before or after bariatric surgery.Beyond age ≥ 60 years, factors such as active smoking, secondary hyperparathyroidism,and decreased appendicular lean mass were identified as independent risk factors, highlighting the importance of lifestyle and dietary measures(smoking cessation, vitamin D and calcium supplementation, adequate protein intake, and physical activity)in the prevention and treatment of osteoporosis secondary to bariatric surgery in addition to AOM.

**P150**

**Validation of a personalised prediction model for hip fracture risk using data from the Study of Osteoporotic Fractures (SOF) cohort**

*Christina Wapp*[1], *Philippe Zysset*[1]
[1]*University of Bern, ARTORG Center for Biomedical Engineering Research, Bern, Switzerland*

**Abstract Text**

**Background:** The risk of hip fractures depends on the risk of falling, the impact force, and the femoral bone strength. We have developed a simple personalised prediction model based on biomechanical principles that integrates these factors in a stochastic process. In addition to age, height and weight, the required parameters can be extracted from QCT images; alternatively, they can be estimated with substitution equations using sex, height, weight and total hip aBMD from DXA. The aim of this study was to assess the model's predictive performance by confronting it with clinical data .

**Methods:** The publicly available data set of the SOF cohort was filtered for individuals with complete information regarding age, height, weight, prior falls, and aBMD at the first follow-up visit. Since the SOF data does not include QCT images, substitution equations were used to calculate all required model parameters. Subsequently, the model was calibrated with 5-year age-group-specific annual incidences, and 1-, 2-, 3- and 5-years fracture risks were calculated. Hip fractures were filtered for femoral neck and intertrochanteric fractures. Receiver operating characteristics and area under the curve (AUC) were evaluated for both the model and aBMD alone.

**Results:** In total, 7625 individuals were included in this analysis (mean age 73.4± 5.01 years ; mean aBMD 0.756 ± 0.13). During the 1, 2, 3, and 5 year follow-up periods, 30, 68, 113 and 198 hip fractures were registered, respectively. The model performed best for a 5-year prediction with an AUC of 0.779 compared to 0.756 for aBMD alone.

**Discussion:** The novel model demonstrated improved discriminative performance compared to aBMD alone. Thus, the use of biomechanical principles together with stochastic modelling to predict hip fracture risk has the potential to improve fracture risk prediction.

**P151**

**Trabecular Bone Score is Associated with Fragility Fractures Among Women with Osteopenia**

*Vivi Mäkinen*[1], *Amalie Lyngberg*[1], *Anne Sophie Sølling*[1], *Torben Harsløf*[1], *Bente Lomholt Langdahl*[1]
[1]*Aarhus University Hospital, Department of Endocrinology and Internal Medicine, Aarhus N, Denmark*

**Abstract Text**

**Introduction:** Most fragility fractures occur in women with osteopenia, defined by a T-score between -1.0 and -2.5. Although bone mineral density (BMD) is traditionally used to estimate fracture risk, bone strength is determined by both bone mass and bone quality. In the literature, a low trabecular bone score (TBS) may independently increase fracture risk. Notably, some individuals with osteopenia have degraded TBS despite only modest reductions in BMD. We investigated if TBS differed in individuals with osteopenia with or without fragility fractures.

**Methods:** We conducted a case-control study (1:1) of women with osteopenia. Cases were identified through their attendance in our Fracture Liaison Service between 01.05.2022-29.04.2024. Controls were matched on age. A review of the patient's electronic record was performed to obtain demographic information and DXA including TBS. Patients with a prior osteoporosis diagnosis, hip or vertebral fractures or receiving osteoporosis treatment were excluded.

**Results:** We included 371 cases (median age 68 (IQR 61;75)) and 381 control subjects (median age 69, IQR (61;75)) No difference was noted for BMI, total hip (p=0.13) or lumbar spine (p=0.57) BMD (Table 1). TBS, however, was significantly lower in cases (1.28 vs. 1.30, p<0.05).

**Conclusion:** In women with osteopenia, TBS was significantly lower in those with a fragility fracture compared to age-matched non-fractured controls, despite similar BMD. Therefore, TBS may be of use for fracture prediction in postmenopausal women with osteopenia.

Table 1*

| | Cases (N = 371) | Controls (N = 381) | p-value |
|---|---|---|---|
| Age, years | 68 (61:75) | 69 (61;75) | 0.35 |
| BMI kg/m$^2$ | 26 (24;30) | 25 (23;30) | 0.08 |
| TBS | 1.281 (1.204;1.354) | 1.300 (1.230;1.371) | 0.04 |
| TH BMD | 0.788 (0.748;0.842) | 0.797 (0.755;0.847) | 0.13 |
| LS BMD | 0.905 (0.844;0.978) | 0.911 (0.848;0.985) | 0.58 |
| FN BMD | 0.666 (0.627;0.700) | 0.670 (0.631;0.712) | 0.14 |

*Data presented as median (IQR) BMI *Body Mass Index* IQR *Interquartile Range* TBS Trabecular Bone Score TH Total Hip FN Femoral Neck LS Lumbar Spine

**P152**

**Risk factors associated with the incidence of fractures in glucocorticoid treated patients.**

*Helena Florez Henrich[1], Katherine Cajiao[2], Josep Lluis Carrasco[3], José Hernández-Rodríguez[4],*

*Africa Muxi[5], Sergio Prieto-González[6], Maria C. Cid[6], Ana Monegal[2], Núria Guañabens[2], Pilar Peris[2]*
*[1]Hospital Clinic. University of Barcelona.,*
*Metabolic Bone Diseases Unit- Department of Rheumatology., Barcelona, Spain*
*[2]Hospital Clinic- University of Barcelona.,*
*Metabolic Bone Diseases Unit. Department of Rheumatology., Barcelona, Spain*
*[3]University of Barcelona- Barcelona, Biostatistics- Department of Basic Clinical Practice, Barcelona, Spain*
*[4]Hospital Clínic- University of Barcelona- Institut d'Investigacions Biomèdiques August Pi i Sunyer IDIB APS- Barcelona, Vasculitis Research Unit- Department of Autoimmune Diseases- Hospital Clínic, Barcelona, Spain*
*[5]Hospital Clinic- University of Barcelona, Department of Nuclear Medicine, Barcelona, Spain*
*[6]Hospital Clínic- University of Barcelona- Institut d'Investigacions Biomèdiques August Pi i Sunyer IDIB APS- Barcelona, Vasculitis Research Unit- Department of Autoimmune Diseases, Barcelona, Spain*

**Abstract Text**

**Background:** Our group previously reported the prevalence and risk factors for osteoporosis (OP) and fractures (fxs) in 127 patients with autoimmune diseases receiving chronic glucocorticoid (GC) therapy[1]. Anti-osteoporotic treatment (AOT) was prescribed according to clinical guidelines.

**Purpose:** To analyze the 5-year incidence of new fragility fractures (FF), adherence to AOT, and identify risk factors associated with new FF in this cohort.

**Methods:** The original cohort was re-evaluated after 5 years. Data collected included: incidence and location of new FF (vertebral [VF] and peripheral), GC dose and duration, time since discontinuation, anthropometrics, AOT adherence, bone mineral density (BMD; DXA, OP: T-score ≤ -2.5), trabecular bone score (TBS; degraded microarchitecture: <1.230), dorsolumbar X-ray for VF, and baseline FRAX risk (GC-adjusted).

**Results:** Of 127 patients, 109 were reassessed (18 deaths); 66.1% were women, mean age 65.4±18.3 years. Median GC dose was 2.5mg/day (25.7% ≥5 mg/day) for 76 months. Forty-eight patients (44%) had discontinued GC 31 months before evaluation. AOT was initially prescribed to 81 patients; 38.27% (31/81) remained on treatment. During follow-up, 13 patients (11.9%) developed FF: 9 VF and 7 peripheral. Patients with new VF were older (88 vs. 67 years, p=0.006), had higher baseline FRAX risk (hip: 3.6 vs. 2.3, p=0.037; MOF: 12.7 vs. 7, p=0.017), previous VF (OR 4.83; 95%CI 1.04–21.3, p=0.039) and parental hip fracture (OR 9.63; 95%CI 1.79–56.7, p=0.003). For peripheral FF, similar results were observed, in addition to falls in the previous year (OR 2.03; 95%CI 0.99–4.18 p=0.034), especially ≥2 (OR 10.4; 95%CI 1.18–94.3 p=0.044). Neither BMD nor TBS were associated with new FF.

**Conclusions:** Age, previous VF, family history of hip Fx and falls were the main risk factors related to incident fxs during the follow-up. In addition, a higher baseline FRAX risk was also associated with fx development, indicating the importance of evaluating these factors in these patients.

[1] Florez H, *RMD Open*. 2020.

**P153**

### Utility of vertebral fracture assessment (VFA) for the identification of vertebral fractures in glucocorticoid-induced osteoporosis.

*Helena Florez Henrich*[1], *Africa Muxi*[2], *José Hernández-Rodríguez*[3], *Josep Lluis Carrasco*[4], *Sergio Prieto-González*[5], *Maria C. Cid*[3], *Ana Monegal*[6], *Núria Guañabens*[6], *Pilar Peris*[6]
*[1]Hospital Clinic. University of Barcelona., Metabolic Bone Diseases Unit- Department of Rheumatology., Barcelona, Spain*
*[2]Hospital Clinic- University of Barcelona, Department of Nuclear Medicine, Barcelona, Spain*
*[3]Hospital Clínic- University of Barcelona- Institut d'Investigacions Biomèdiques August Pi i Sunyer IDIB APS- Barcelona, Vasculitis Research Unit- Department of Autoimmune Diseases, Barcelona, Spain*
*[4]University of Barcelona- Barcelona, Biostatistics- Department of Basic Clinical Practice, Barcelona, Spain*
*[5]Hospital Clínic- University of Barcelona- Institut d'Investigacions Biomèdiques August Pi i Sunyer IDIB APS- BarcelonaHospital Clínic- University of Barcelona- Institut d'Investigacions Biomèdiques August P i i Sunyer IDIBAPS- Barcelona, Vasculitis Research Unit- Department of Autoimmune Diseases, Barcelona, Spain*
*[6]Hospital Clinic- University of Barcelona., Metabolic Bone Diseases Unit. Department of Rheumatology., Barcelona, Spain*

### Abstract Text

**Background:** Glucocorticoid-induced osteoporosis (GIOP) is a common form of secondary osteoporosis, and up to 50% of patients may develop fractures (Fx), with vertebral fractures (VF) being the most frequent. Radiography (X-ray) is the gold standard for diagnosis, although Vertebral Fracture Assessment (VFA) using dual-energy X-ray absorptiometry (DXA) can identify VF while measuring bone mass, which may be useful in GIOP assessment.

**Purpose:** To evaluate the usefulness of VFA in detecting VF in glucocorticoid (GC)-treated patients and compare it with X-ray.

**Methods:** A cross-sectional study included 127 patients on chronic GC therapy (≥5mg/day) (62±18years, 63% women). All underwent dorsolumbar X-ray (Genant criteria) and DXA (Lunar Prodigy) for vertebral morphometry (T4–L4). Concordance between techniques was assessed using the kappa index (≥0.41 moderate, ≥0.61 substantial, ≥0.81 near perfect). VFA sensitivity and specificity versus X-ray were calculated. Discordant cases were reviewed by two independent observers.

**Results:** 21 patients (16.6%) had at least one VF on X-ray (39 VF total).

| vertebra | True+ | False- | False+ | True- | Kappa |
|---|---|---|---|---|---|
| T4 | 0 | 1 | 2 | 124 | -0.011 |
| T5 | 1 | 3 | 3 | 120 | 0.226 |
| T6 | 1 | 0 | 1 | 125 | 0.663 |

| | | | | | |
|---|---|---|---|---|---|
| T7 | 1 | 1 | 2 | 123 | 0.388 |
| T8 | 0 | 2 | 2 | 123 | -0.016 |
| T9 | 0 | 1 | 2 | 124 | -0.011 |
| T10 | 3 | 0 | 2 | 122 | 0.742 |
| T11 | 5 | 4 | 1 | 117 | 0.647 |
| T12 | 2 | 4 | 2 | 119 | 0.376 |
| L1 | 5 | 1 | 0 | 121 | 0.905 |
| L2 | 0 | 2 | 0 | 125 | 0.000 |
| L3 | 0 | 1 | 3 | 123 | -0.012 |
| L4 | 0 | 1 | 1 | 125 | -0.008 |

Overall concordance was low (k=0.444), with sensitivity 46% and specificity 99%. Segmental concordance was moderate at T9–T12 and L1–L2 (k=0.536 and 0.513) and low at T4–T8 (k=0.245). Main discordance causes were degenerative changes, scoliosis, and poor definition in upper thoracic vertebrae.

**Conclusions:** VFA showed low sensitivity for VF detection in GC-treated patients, but its high specificity allows accurate identification of those requiring anti-osteoporotic treatment. Radiographic assessment should be performed concomitantly.

**P154**

**Ex vivo osteodensitometry and hip structural analysis in men with intravenous heroin addiction: preliminary data**

*Jelena Jadzic*[1], *Aleksa Lekovic*[2], *Slobodan Nikolic*[2], *Danijela Djonic*[1]
[1]*Faculty of Medicine- University of Belgrade, Center of Bone Biology, Belgrade, Serbia*
[2]*Faculty of Medicine- University of Belgrade, Institute of Forensic Medicine, Belgrade, Serbia*

**Abstract Text**

**Background:** Reports about skeletal alterations in individuals prone to heroin abuse are scarce in contemporary literature. Most of the previous data on heroin-induced low bone mineral density come from case reports or indirect findings from small-scale studies.

**Purpose:** We aimed to conduct *ex vivo* osteodensitometry and hip structure analysis of standardized regions of proximal femora collected from adult male cadavers with macroscopic stigmata of intravenous heroin addiction (IHA) and an age-matched control group.

**Methods:** With institutional ethic committee approval, a total of 73 proximal femora were obtained during autopsy and divided into the IHA group (n=32, age range: 26-70 years) and a control group (n=41, age range: 23-73 years). The Hologic Horizon Ci DXA device was used to perform *ex vivo* osteodensitometry of water-submerged proximal femora, and Hologic Apex software was used to perform hip structure analysis (HSA) on the same samples.

**Results:** Although our data implied a declining trend in bone mineral density and HSA parameters in the femoral neck and intertrochanteric region of the IHA group, the inter-group differences were not significant (Student t test for two independent samples, p>0.05).

**Conclusions:** The region-specific femoral bone mineral density and HSA parameters were not significantly altered in men with heroin addiction, implying that IHA *per se* does not have a substantial negative impact on bone health. Still, future studies are required to confirm these findings and to fully understand bone alterations in individuals prone to heroin abuse.

**P155**

**Bone Mineral Density assessment according to Italian Essential Assistance Levels: a positive predictive value analysis based on administrative data.**

*<u>Veronica Abate</u>[1], Anita Vergatti[2], Guido Cavati[3], Simone Magelli[1], Varriale Alfonso[2], Daniela Merlotti[3], Gaetano Piccinocchi[4], Lanfranco D'Elia[1], Luigi Gennari[3], Domenico Rendina[1]*
*[1]Federico II University, Department of Clinical Medicine and Surgery, Naples, Italy*
*[2]Federico II University, Department of Clinical Medicine and Surgery, Napoli, Italy*
*[3]University of Siena, Department of Medicine- Surgery- and Neurosciences, Siena, Italy*
*[4]COMEGEN, comegen, Napoli, Italy*

**Abstract Text**

**Background:** Osteoporosis (OP) is the gradual loss of bone mineral density (BMD), predisposing to fragility fractures. It can be the result of aging or menopause in primary OP, or other diseases and medications in secondary OP. The gold standard diagnostic tool for low BMD is represented by Dual-energy X-ray absorptiometry (DXA), which has been included among the Essential Assistance Levels (EALs) in Italy for adequate prescription. This observational study based on administrative data evaluates whether the EALs are designed to predict OP (designed to include OP screening), with subsequent BMD-DXA evaluation in Italian men and women. The SIMON database was used to selected women and men scheduled to undergo BMD-DXA according to EALs.

**Results:** BMD was evaluated in 18087 subjects [Male (M): Female (F) 889: 17198; mean age 71.3 ± 9.3 years; body mass index (BMI): 26.9 ± 4.5 Kg/m$^2$] for primary OP and in 6503 subjects (M: F 649: 5854; mean age 70.5 ± 9.4 years; BMI; 25.4 ± 4.8 Kg/m$^2$) for secondary OP, both suspected on the basis of EALs. Primary OP was confirmed by DXA in 13759 (80.0%; mean age71.9 ± 9.2 years; BMI: 26.5 ± 4.8 Kg/m$^2$) women and in 551 men (62.0%; mean age 69.3 ± 9.4 years; BMI: 26.5 ± 4.8 Kg/m$^2$). Secondary OP was confirmed by DXA in 4683 women (80.0%; mean age 70.7 ± 8.9 years; BMI; 25.3 ± 5.0 Kg/m$^2$) and in 514 men (79.2%; mean age 69.7 ± 9.4 years; BMI: 26.3 ± 5.3 Kg/m$^2$). The positive predictive values of EALs for OP was higher among women for primary Op than men, and similar in both genders without any significant difference in secondary OP.

**Conclusion:** Italian EAL are a useful diagnostic tool for primary of OP in both sexes, with significant gender dimorphism evident in secondary OP.

**P156**

**Feasibility of pQCT for people with Parkinson's**

*Katie Naylor*[1], *Emma Tenison*[1,2], *Katherine Lloyd*[1], *Nicola Giles*[1], *Leonardo Bruzze*[1], *Pimkhuan Kamnerdsupaphon*[1], *Mícheál Ó Breasail*[3], *Emily J Henderson*[1,2]
[1]*University of Bristol, Population Health Sciences, Bristol, United Kingdom*
[2]*Royal United Hospital, Older People's Unit, Bath, United Kingdom*
[3]*Monash University, Department of Medicine, Clayton, Australia*

**Abstract Text**

**Background:** People with Parkinson's have a greater risk of low bone mineral density (BMD), and of falls, both contributing to an increased risk of fractures [1]. Currently dual-energy X-Ray absorptiometry (DXA) scanners are used to measure BMD from 2-dimensional images. However, such areal measures are affected by bone size, and scans are not always practical for frail individuals. [2-3] Peripheral quantitative computed tomography (pQCT) scans assess bone density, composition, and geometry in 3-dimensional space, providing volumetric measures independent of bone size. [4]

**Purpose and Methods:** We assessed the feasibility of conducting pQCT scans of the lower-leg and forearm in a Parkinson's cohort using the Stratec-XCT2000L (REC reference 21/LO/0387). Three sites were imaged in the lower-leg, and two were imaged in the forearm. Image quality and positioning were graded.

**Results:** In a Parkinson's cohort (n=143) with mean age 74.7 years (SD=7.91), 29.4% clinical frailty scale 5 or more, 141 lower-leg and 163 forearm scans were completed including some triplicate repeats. Difficulty positioning participants, and hardware/software issues were the main reasons why leg and arm scans were not completed respectively. Of the completed scans 97% of slices obtained from the leg, and 90% of slices from the arm were of sufficient quality for analysis.

**Conclusion:** We have shown that pQCT scans are feasible in a Parkinson's cohort including older adults living with frailty. Tremor or dyskinesia rarely resulted in severe movement artefacts, and most scans were of sufficient quality for analysis. Using a scanner with a larger aperture would increase the number of scans at the leg.

**P157**

**Burn injuries locally affect the underlying bone**

*Maximilian Moshammer[1], Nikolaus Watzinger[1], Andrzej Hecker[2], Christian Smolle[3], Michael Kohlhauser[1], Barbara Obermayer-Pietsch[4], Petra Kotzbeck[5], Lars Kamolz[1], Ines Foessl[6]*
*[1]Medical University Graz,*
*Department of Surgery- Division of Plastic- Aesthetic and Reconstructive Surgery, Graz, Austria*
*[2]Medical University Graz and Hospital Ernst von Bergmann,*
*Department of Surgery- Division of Plastic- Aesthetic and Reconstructive Surgery and Department of P lastic- Aesthetic and Reconstructive Microsurgery/Hand Surgery, Graz and Potsdam, Austria*
*[3]Medical University Graz and JOANNEUM RESEARCH Forschungsgesellschaft mbH,*
*Department of Surgery- Division of Plastic- Aesthetic and Reconstructive Surgery, Graz, Austria*
*[4]Medical University Graz, Internal Medicine- Div. Endocrinology and Diabetology, Graz, Austria*
*[5]Medical University Graz and JOANNEUM RESEARCH Forschungsgesellschaft mbH,*
*Department of Surgery- Division of Plastic- Aesthetic and Reconstructive Surgery and COREMED-Centre for Regenerative and Precision Medicine, Graz, Austria*
*[6]Medical University Graz and JOANNEUM RESEARCH Forschungsgesellschaft mbH,*
*Dept. of Internal Medicine- Division of Endocrinology and Diabetology and COREMED-Centre for Regenerative and Precision Medicine, Graz, Austria*

**Abstract Text**

**Background:** High-resolution peripheral computed tomography (HRpQCT) can be used for local bone structure investigation at arms and legs. Burn injuries cause significant systemic bone loss due to hypermetabolic bone turnover and systemic inflammation - however, peripheral local reactions are not documented. The present study investigated local effects on peripheral bone after hand burn.

**Methods:** Patients with unilateral hand burns (degree 2b°–3°) were included. The site of the deepest burn and the radius of the affected hand were compared intraindividually with the corresponding site and radius of the control hand. Hand function, activities of daily life (ADL), and grip strength were evaluated using a score for Disabilities of the Arm, Shoulder, and Hand Questionnaire (Quick-DASH), the Michigan Hand Questionnaire (MHQ), and a hand grip dynamometer.

**Results:** The study included 9 patients. Total volumetric bone mineral density (vBMD) was significantly reduced at the site of the deepest burn compared to the control hand (p=0.035), with a strong effect size of -0.807 (95% CI: [-1.522, -0.056]). No difference was observed at the radius level (p=0.764). Further effects of burn injuries were found for trabecular vBMD (60% of Tb.Ar; 95% CI: [-1.209, 0.143]), trabecular area (Tb.Ar; 95% CI: [-0.066, 1.324]), and cortical vBMD (95% CI: [-1.315, 0.072]). Grip strength tended to be higher in the burned hands (42.6±10.48 vs. 40.7±9.73; p=0.139). Quick-DASH hand function was reduced (88.77±10.48 vs. 92.37±5.92; p=0.029), whereas ADL performance showed no significant differences (93.02±12.58 vs. 94.40±11.73; p=0.411).

**Conclusion:** Burns are widely affecting bone structure – we detected a local impact on the bone that extends beyond the systemic effects. To our knowledge, this is the first study to document local effects directly beneath the burn. Despite these localised changes, the overall hand function was only slightly reduced with preservation of activities of daily life and grip strength.

**P158**

**Impact of Bariatric Surgery on Musculoskeletal Health After Age 60**

*Line Abdulghani[1], Julien Paccou[1], Cécile Philippoteaux[1], Robert Caiazzo[2], Hélène Verkindt[3], Laurine Cadart[4]*
*[1]Lille University Hospital, Rheumatology, Lille, France*
*[2]Lille University Hospital, Endocrine Surgery, Lille, France*
*[3]Lille University Hospital, Nutrition, Lille, France*
*[4]Lille University Hospital, Biostatistics Department, Lille, France*

**Abstract Text**

**Introduction:** After the age of 60, the risk of postoperative complications following bariatric surgery is high.However, specific data regarding the bone impact in this vulnerable population are lacking. The 2022 recommendations of the ECTS suggest initiating anti-osteoporotic medication (AOM)in case of a T-score ≤ -2 and/or in case of a fragility fracture within less than 2 years.Therefore, our study evaluated the prevalence of eligibility for AOM according to age(< 60 vs. ≥ 60 years)in a cohort of postmenopausal women, as well as other associated risk factors.

**Materials and Methods:** Observational, retrospective, monocentric study conducted at Lille University Hospital evaluated the prevalence of and risk factors for eligibility for AOM according to the 2022 ECTS criteria in postmenopausal women referred to rheumatology for bone health assessment before or after bariatric surgery.

**Results:** Among the 306 postmenopausal women included, 173 were aged < 60 years(group 1) and 133 were aged ≥ 60 years(group 2).Indications for AOM were found in 69 (22.5 %)patients.This prevalence was significantly higher in women in Group 2 (30.1% vs. 16.8%, p = 0.006). In multivariate analysis, risk factors independently associated with eligibility for AOM were age ≥ 60 years(OR= 2.34, 95% CI: 1.19–4.61),active smoking(OR = 2.98, 95% CI: 1.27–7.02),reduced appendicular lean mass/height$^2$ (< 5.5 kg/m$^2$)(OR = 3.72, 95% CI: 1.78–7.78), and elevated iPTH (OR = 1,15, 95% CI 1,05-1,36 for each increase of 10 ng/ml).

**Conclusion:** One in five postmenopausal women was eligible for AOM as part of their care pathway either before or after bariatric surgery.Beyond age ≥ 60 years, factors such as active smoking, secondary hyperparathyroidism,and decreased appendicular lean mass were identified as independent risk factors, highlighting the importance of lifestyle and dietary measures(smoking cessation, vitamin D and calcium supplementation, adequate protein intake, and physical activity)in the prevention and treatment of osteoporosis secondary to bariatric surgery in addition to AOM.

**P159**

**Assessing osteoporosis using radiographs of the hands in rheumatoid arthritis patients- A diagnostic accuracy study**

Magdalena Gilg[1,2], Ulla Stumpf[3], Annsophie Arbogast[4], Olivia Helder[4], Thomas Kuenzer[5], Martin Arbogast[4], Diethard Kaufmann[6]
[1]Centre of Rheumatology Oberammergau- Germany, Department of Orthopaedics and Trauma, Oberammergau, Germany
[2]Medical University of Graz, Department of Orthopaedics and Trauma, Graz, Austria
[3]Musculoskeletal University Center Munich- Ludwig-Maximilians Universität Munich, Center of Osteology- Department of General- Trauma and Reconstructive Surgery, Munich, Germany
[4]Centre of Rheumatology Oberammergau, Department of Orthopaedics and Trauma, Oberammergau, Germany
[5]Medical University of Graz, Institute for Medical Informatics- Statistics and Documentation, Graz, Austria
[6]Department of Rheumatology, Centre of Rheumatology Oberammergau, Oberammergau, Germany

**Abstract Text**

**Background:** Generalized osteoporosis is a common finding in rheumatoid arthritis (RA) patients. Recently, the second metacarpal cortical percentage (2MCP), obtained from hand X-rays, was found to correlate with hip bone mineral density derived from dual-energy X-ray in postmenopausal women.

**Purpose:** Using the 2MCP in RA patients who undergo regular hand X-rays, would provide less resource-intensive access to osteoporosis assessment. This study aimed to examine the diagnostic performance of the 2MCP in identifying patients with osteopenia/osteoporosis in RA patients.

**Methods:** The 2MCP is calculated as the ratio of the cortical to the total diameter at the isthmus of the metacarpal bone. This retrospective study assessed the diagnostic performance of the 2MCP by analyzing inter-rater variability and the area under the curve (AUC), including ROC analyses to identify the best thresholds for diagnosing osteoporosis/osteopenia with the total hip t-score as the reference standard.

**Results:** We included 359 patients in this study. We found a strong correlation when two independent raters measured 2MCP (Spearman rho of 0.92 (95% CI 0.91-0.94)) and between the left and right hands (Spearman rho of 0.94 (95% CI 0.93 - 0.95)).

A 2MCP threshold of 50% yielded a sensitivity of 90.6% (95% CI 75.0 to 98.0) and a specificity of 55.4% (95% CI 49.8 to 60.8) in identifying patients with osteoporosis (t-score total hip <-2.5). A 2MCP threshold of 60% yielded a sensitivity of 91.7% (95% CI 86.2.0 to 95.5) and a specificity of 41.4% (95% CI 34.5 to 48.5) in identifying patients with osteopenia or worse (t-score total hip < − 1) .

**Conclusion:** The 2MCP shows good diagnostic performance and can be used as a simple screening tool to identify patients with RA who are at risk of osteoporosis and should undergo further diagnostic workup

**P160**

**Feasability of quantitative bone parameters measurement using Magnetic Resonance Imaging in a murine model**

*Nada Ibrahim-Nasser*[1,2], *Eric Lespessailles*[1], *Frederic Szeremeta*[2], *Rudy Clemencon*[2], *Sandra Meme*[2]
[1]*CHU Orleans, Rheumatology, Orleans, France*
[2]*CNRS, CBM UPR 4301 Mo2ving MRI platform, Orleans, France*

**Abstract Text**

**Purpose:** To evaluate the feasibility and variability of bone quantitative parameters assessment using Magnetic Resonance Imaging MRI in a mouse model.

**Methods:** Three 24-week-old CD1 mice were analyzed twice with repositioning to evaluate the coefficient of variation CV at each site studied and the least significant change LSC. Suitable coils were made for the spine and the lower limb, minimizing artifacts and mobility. Acquisitions were performed using a 7 Tesla imaging spectrometer, specifically designed for in-vivo imaging of small animals. Spin echo, gradient echo, and ultrashort echo sequences were used. 3 slices were positionned at each lower limb site - proximal femoral shaft, distal femur, proximal tibia- and one slice for each lumbar vertebra from L1 to L5. The parameters measured were : signal-to-noise ratio SNR, T1 and T2 values in milliseconds, the femoral cortical porosity index (PI), the fat fraction for bone marrow adiposity (FF) in the spine and bone texture analysis using radiomics.

**Results:** The measurement was non invasive, with an acquisition time of 1 hour and a resolution of 50 micrometers. The CV varied according to the site and the parameter evaluated. Thus, SNR at all sites, T1,T2 values of the slices closest to the femoro-tibial joint, L1 and L5, showed high CV, as well as the FF, ranging from 17% to 40%. In contrast, the midline slices at each lower limb site parameters had CV ranging from 2% to 10%. The most reproducible data was the femoral cortical PI, with a coefficient of 6.8% and a PPVS of 0.048. The results in texture analysis were homogeneous, with similar profiles after repositioning.

**Conclusion:** MRI is a reproducible, non-invasive measurement tool at the lower limb in mice, which could be used for the non-invasive longitudinal monitoring of bone quantitative parameters in further studies.

**ND-P017**

**Prevalence of cortical–trabecular bone density imbalance at the proximal femur in adults assessed by 3D-DXA**

*Enrique Casado*[1], *Silvana Di Gregorio*[2], *M. del Carmen Valero*[3], *Jesús González-Macías*[3], *Jose*

*Manuel Olmos[3], Rosa María Arboiro-Pinel[4], Manuel Diaz-Curiel[4], M. Mercedes Giner[5], M. José Montoya-García[6], María Cortés-Berdonces[7], Esteban Jodar[8], Mireia Barceló-Bru[9], José Luis Pérez-Castrillón[10], Beatriz García-Fontana[11], Manuel Muñoz-Torres[11], Pilar Aguado-Acín[12], Carolina Tornero[12], Federico Hawkins[13], Guillermo Martínez Diaz-Guerra[14], Javier Del Pino-Montes[15], Jorge Malouf[16], Ludovic Humbert[17], Marta Bracco[17], Luis Del Rio[2]*

*[1]Parc Taulí Hospital Universitari / Institut d'Investigació i Innovació Parc Taulí I3PT-, Unitat de Metabolisme Ossí- Servei de Reumatologia, Sabadell, Spain*

*[2]CETIR-ACIRES, Endocrinology / Bone Metabolism, Barcelona, Spain*

*[3]Hospital Universitario Marqués de Valdecilla- Universidad de Cantabria- IDIVAL, Endocrinology / Bone Metabolism, Santander, Spain*

*[4]Instituto de Investigación Sanitaria- Hospital Universitario Fundación Jiménez Díaz, Endocrinology / Bone Metabolism, Madrid, Spain*

*[5]Universidad de Sevilla, Departamento de Citología e Histología Normal y Patológica- Facultad de Medicina, Sevilla, Spain*

*[6]Universidad de Sevilla, Departamento de Medicina, Sevilla, Spain*

*[7]Hospital Universitario Ruber Juan Bravo, Endocrinology, Madrid, Spain*

*[8]Universitario Ruber Juan Bravo / Universidad Europea de Madrid, Departamento de Medicina, Madrid, Spain*

*[9]Hospital Universitario Vall d'Hebron, Endocrinology, Barcelona, Spain*

*[10]Hospital Río Hortega, Endocrinology, Valladolid, Spain*

*[11]Instituto de Investigación Biosanitaria de Granada ibs.GRANADA / Hospital Universitario Clínico San Cecilio / Universidad de Granada, UGC Endocrinología y Nutrición / Departamento de Medicina, Granada, Spain*

*[12]Hospital Universitario La Paz, Servicio de Reumatología, Madrid, Spain*

*[13]Instituto de Investigación Sanitaria i+12- Hospital Universitario 12 de Octubre / Universidad Complut ense, Endocrinology / Bone Metabolism, Madrid, Spain*

*[14]Instituto de Investigación Sanitaria i+12- Hospital Universitario 12 de Octubre / Universidad Complut ense, Endocrinología, Madrid, Spain*

*[15]Hospital Universitario de Salamanca, Endocrinology, Salamanca, Spain*

*[16]Hospital de la Santa Creu i Sant Pau, Metabolismo Mineral / Dolor, Barcelona, Spain*

*[17]3D-Shaper Medical, Research & Development, Barcelona, Spain*

**Abstract Text**

**Introduction:** Standard dual-energy X-ray absorptiometry (DXA) provides areal (a) bone mineral density (BMD) but does not distinguish between cortical and trabecular compartments, which may contribute differently to bone strength and fracture risk.

**Purpose:** To determine the prevalence of significant cortical–trabecular bone density imbalances at the proximal femur in an adult reference population using three-dimensional (3D) DXA.

**Methods:** Hip DXA scans from 1366 adults (1015 women, 351 men) were retrospectively analysed using a validated 3D-DXA modelling approach (3D-Shaper v2.14, 3D-Shaper Medical, Barcelona, Spain). Trabecular volumetric (v) BMD and cortical surface (s) BMD Z-scores were derived from sex- and age-specific reference curves. Compartmental imbalance was defined as an absolute difference between trabecular and cortical Z-scores exceeding the least significant change (LSC), calculated from precision error. Participants were stratified by sex and age. The study involved analysis of anonymised human imaging data obtained according to ethical standards.

**Results:** The LSC for the Z-score difference was 0.397. A significant compartmental imbalance was observed in 52.0% of women and 48.7% of men (Figure). In women, 26.3% showed trabecular-dominant patterns (positive Z-score difference), while 25.7% showed cortical-dominant patterns (negative Z-score difference). Similar proportions were observed in men (25.4% trabecular-dominant; 23.4% cortical-dominant). Both imbalance patterns were consistently present across all age groups, with no marked age dependency.

**Conclusion(s):** Nearly half of adults in a reference population exhibit significant cortical–trabecular bone density imbalances at the proximal femur that are not captured by standard DXA aBMD. Compartment-specific assessment using 3D-DXA may provide additional insights into bone strength and fracture risk beyond conventional measures. Further studies are warranted to evaluate the clinical implications of these imbalances.

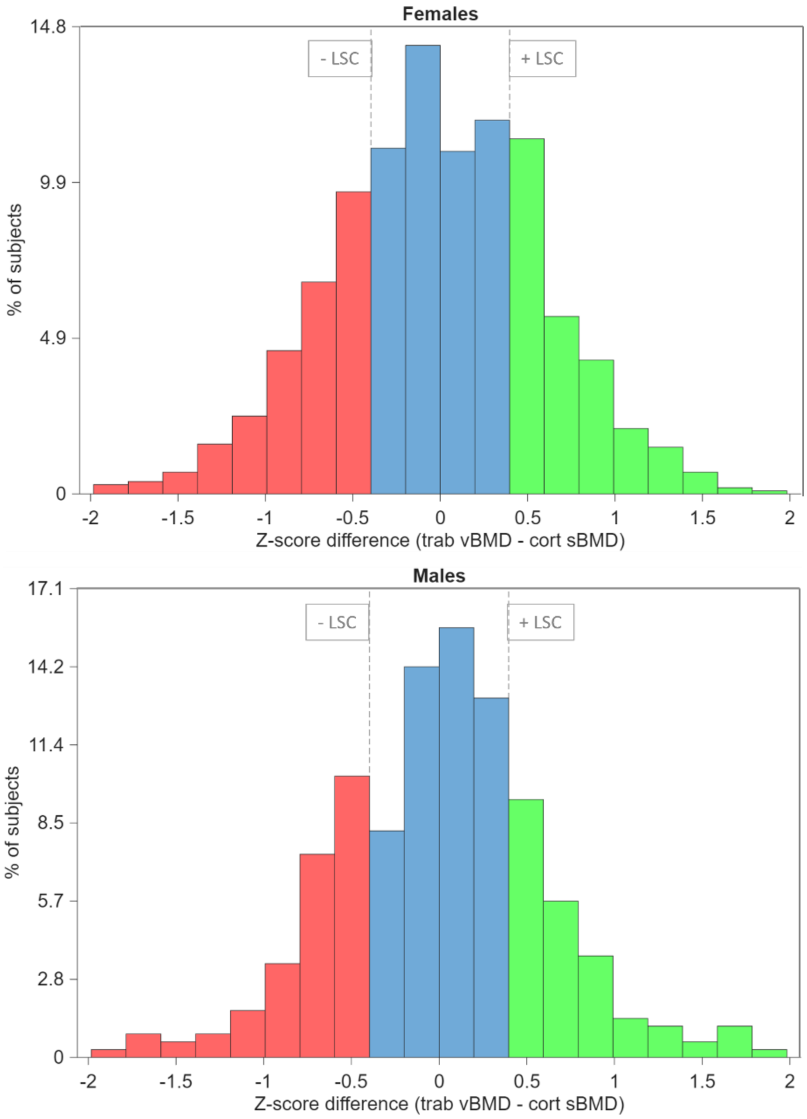

**ND-P018**

### Densitometric–microarchitectural discordance after romosozumab: real-world detectable-change thresholds and bone turnover markers

*Montserrat Robustillo Villarino*[1], *Andrés Zuñiga-Vera*[2], *Marile Sanabria-Hernández*[2], *Laia Gifre-Sala*[3], *Rodrigo Duran-Rodríguez*[4], *ELena Naveda*[5], *Soledad Ojeda*[5], *Antonio Naranjo-Hernández*[5]
[1]*Hospital Universitari de la Plana, Rheumatology, Vila-Real, Spain*
[2]*Hospital Universitario de la Plana, Rheumatology, Vila-Real, Spain*
[3]*Hospital Universitari Germans Trias i Pujol, Rheumatology, Badalona, Spain*
[4]*Hospital Universitari Trias i Pujol, Rheumatology, Badalona, Spain*
[5]*Hospital Universitario de Gran Canaria Dr Negrín, Rheumatology, Gran Canaria, Spain*

### Abstract Text

**Background:** DXA bone mineral density (BMD) is the main monitoring parameter in osteoporosis, while trabecular bone score (TBS) provides complementary microarchitectural information. In routine practice, it is unclear how closely TBS changes track densitometric response to romosozumab and whether BTMs or prior antiresorptive exposure influence this relationship.

**Purpose:** To quantify concordance/discordance between BMD and TBS changes after romosozumab, estimate proportions exceeding detectable-change thresholds, and explore associations with BTMs and prior antiresorptive therapy.

**Methods:** Retrospective multicenter study in women treated with romosozumab. Percent changes (Δ%) in lumbar spine (LS), femoral neck (FN) and total hip (TH) BMD and lumbar TBS (when available) were analyzed. Clinically relevant BMD response was defined as Δ%≥3%. TBS change was assessed by direction (Δ%>0) and least significant change (|Δ%|≥3.0% and ≥5.8%). Pearson correlations evaluated baseline TBS and β-CTX suppression versus Δ% LS BMD and Δ% TBS.

**Results:** 111 patients were assessed (paired TBS n=71). BMD increase ≥3% occurred in 89.0% for LS, 56.9% for FN and 62.0% for TH. TBS increased in 66.2%, was unchanged in 1.4% and decreased in 32.4%; 40.8% achieved |Δ%|≥3.0% and 35.2% achieved |Δ%|≥5.8%. In low LS BMD responders, TBS was often discordant: with Δ% LS BMD <3% (n=6), 4/6 increased TBS; with Δ% LS BMD <6% (n=20), 12/20 increased TBS and 14/20 met |Δ%|≥3.0%. Baseline TBS correlated with Δ% LS BMD (r=+0.234; p=0.039). Greater β-CTX suppression correlated with larger LS BMD gains (r=−0.304; p=0.019) and with Δ% TBS (r=−0.381; p=0.045; small n).

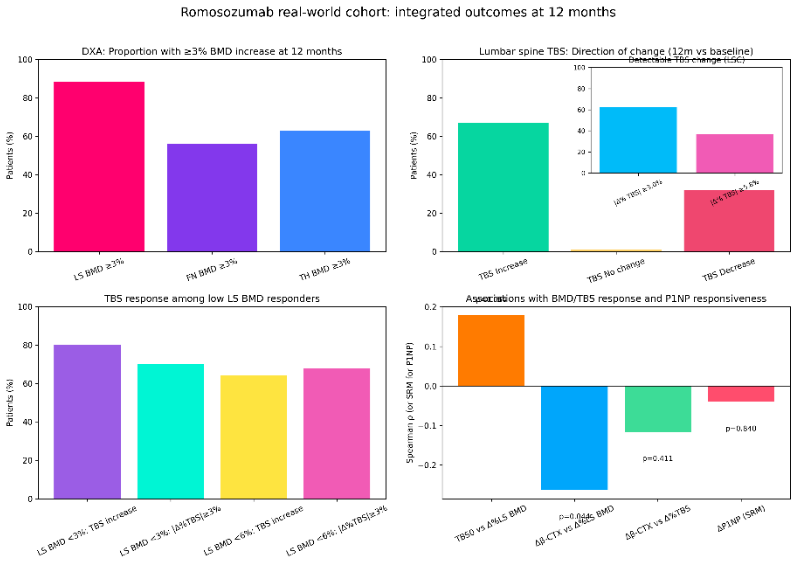

**Conclusions:** Romosozumab produced clinically relevant BMD gains in most patients, whereas TBS response was more heterogeneous and BMD–TBS discordance was common, including among low densitometric responders. β-CTX suppression related to greater BMD gains and, in small samples, to TBS change, suggesting that integrating BTMs and treatment history may improve interpretation of microarchitectural response.

**ND-P019**

**Intra and extracapsular hip fragility fractures differentiation based on cortical thickness and porosity obtained using bi-directional axial transmission**

*Jean-Gabriel Minonzio[1], Espinoza Cristina[2], Viviana Garcia[3], Eva Madrid[4], José Luis Dinamarca-Montecinos[5]*
*[1]Universidad de Valparaiso, Faculty of Engineering, Valparaiso, France*
*[2]Universidad de Valparaiso, Faculty of Engineering, Valparaiso, Chile*
*[3]Universidad de Valparaiso, Geropolis, Valparaiso, Chile*
*[4]Universidad de Valparaiso, Faculty of Medicine, Valparaiso, Chile*
*[5]Hospital Dr. Gustavo Fricke, Orthogeriatric Program- Adult Orthopedics and Traumatology Ward, Viña del Mar, Chile*

**Abstract Text**

**Background**: Intra- and extracapsular hip fragility fractures (ICF and ECF) are still mainly considered as one entity, despite evidences. Their differentiation should improve their prevention and treatments. Quantitative Ultrasound (QUS) has demonstrated a clear potential for fragility fracture assessment.

**Methods**: Bi-Directional Axial Transmission (BDAT) allows to estimate cortical thickness (Ct.Th) and porosity (Ct.Po) at the one-third distal radius. In this study, 130 participants (100 women, 30 men) were included, 60 without history of fragility fracture (NF group), and 70 with recent hip fragility fracture from Hospital Fricke.

**Results**: Differences in age (74±7 *vs* 80±8 years, $p<0.001$), weight (68±13 *vs* 60±13 kg, $p<0.001$) and BMI (27±4 *vs* 23±4 kg.m-2, $p<0.001$). BDAT parameters were found significantly different for Ct.Th (2.9±0.6 *vs* 2.6±0.8 mm, $p=0.02$), Ct.Po (10.2±3.8 vs 12.9±4.8 %, $p=0.001$) and the ratio Ct.Po/Ct.Th (3.6±1.5 *vs* 5.2±1.8, $p<0.001$). The highest discrimination was obtained considering the area under the ROC curve (AUC) and the odds ratio (OR) for the ratio Ct.Po/Ct.Th, adjusted with age and BMI: AUC=0.81 and OR=1.63 ($p=0.04$).

When comparing 31 ICF to NF, Ct.Po was found significantly different (12.4±4.5 %, $p=0.02$) while no significant difference were observed for Ct.Th. Differently, when comparing 39 ECF to NF, both parameters, Ct.Th (2.5±0.9 mm, $p=0.004$) and Ct.Po (13.4±5.1 %, $p=0.001$) were found different. Morever, the ICF group was found younger (78±8 vs 82±8, p=0.03) in agreement with previous studies. ICF and ECF were also differentiated by the ratio Ct.Po/ Ct.Th (4.6±1.6 *vs* 5.6±1.8, $p=0.02$) with the age, BMI-adjusted discrimination equal to AUC=0.73 and OR=1.92 ($p=0.04$).

**Conclusion**: This study confirms the benefit of using BDAT for fragility fracture risk assessment, in particular where DXA is not available. Moreover, cortical thickness and porosity, can be clinically interpreted in terms of bone quantity and quality, as shown here for ICF and ECF differentiation.

**ND-P020**

**Routine admission biomarkers discriminate intra- and extracapsular hip fragility fractures: a real-world orthogeriatric cohort study**

*Jean-Gabriel Minonzio*[1]*, Miguel Yáñez*[2]*, Diego Mellado*[3]*, Constanza Toloza Zamora*[4]*, Ana Aguilera*[5]*, José Luis Dinamarca-Montecinos*[6]

[1]*Universidad de Valparaiso, Faculty of Engineering, Valparaiso, France*
[2]*Universidad de Valparaiso, PhD program in Applied Informatics, Valparaiso, Chile*
[3]*Universidad de Valparaiso, PhD program in Science- Engineering for Health, Valparaiso, Chile*
[4]*Hospital Dr. Gustavo Fricke, Clinic Laboratory Unit, Viña del Mar, Chile*
[5]*Universidad de Valparaiso, Faculty of Engineering, Valparaiso, Chile*
[6]*Universidad de Valparaiso, Orthogeriatric Program- Adult Orthopedics and Traumatology Ward, Viña del Mar, Chile*

**Abstract Text**

**Background**: Hip fragility fractures are a significant geriatric syndrome due to their clinical, epidemiological, organisational, and economic impact. There are elements suggesting that they may represent at least two different entities, which would significantly impact our understanding of their prevention, treatment, and prognosis. The aim of the study is to explore the ability of 18 orthogeriatric hematological biomarkers to discriminate between intra- and extracapsular hip fragility fractures, denoted Chronic Risk Factors for Bone Damage (CRiF-BD).

**Methods**: A descriptive analysis was performed using data from an Orthogeriatric Program database, comprising 743 cases of hip fragility fractures in patients aged 60 years or older, hospitalised during 2023 and 2024. Statistical analyses included Student, Mann–Whitney and chi-square tests.

Multivariate analyses were also conducted, incorporating correlation matrices, hierarchical clustering, and logistic regression modelling.

**Results**: Albumin ($p < 0.001$), haemoglobin ($p < 0.001$), haematocrit ($p < 0.001$), free thyroxine ($p < 0.01$), potassium ($p < 0.05$) and glomerular filtration rate ($p < 0.05$) were identified as significant discriminators between intra- and extra-capsular hip fragility fractures. Among all models tested, the combination of haematocrit, albumin and urea in a logistic regression yielded the highest performance, with an area under the curve (AUC) of 0.71 ± 0.05.

**Conclusion**: The aetiology of bone damage associated with HFF is multi-factorial and depends on the interaction between different CRiF-BD, each one belonging to different and complementary physio-pathological ways. Interactions between haematocrit, albuminaemia and uraemia are useful to generate a model to explain etiological differences between ICF and ECF. These findings enhance our understanding of clinical and biochemical risk factors that may differentiate intracapsular and extracapsular hip fractures in older patients, potentially guiding targeted diagnostic, therapeutic and prognosis strategies.

**ND-P021**

**Diagnostic yield of vertebral fracture assessment (VFA) for the detection of "any", "unknown", or "treatment-decision–relevant" vertebral fractures: a study of 1,876 patients**

*Paul Husquinet[1], Marine Mina[1], Pauline Salpetier[1], Clio Ribbens[1], Olivier Malaise[1]*
*[1]CHU de Liège, Rheumatology, Liège, Belgium*

**Abstract Text**

**Background:** Vertebral fracture assessment (VFA), performed during dual-energy X-ray absorptiometry (DXA), is recommended by guidelines. Nevertheless, in an era of widespread medical imaging, data regarding the diagnostic yield and added clinical value of systematic VFA remain limited.

**Methods:** Patients referred for DXA at our center systematically underwent VFA. We retrospectively analyzed all VFA examinations performed between May 2022 and February 2023. Medical records were reviewed to determine whether identified fractures were previously known and whether their detection could influence therapeutic decisions.

**Results:** Among 1,876 included DXA examinations (70% women; median age 67), VFA identified at least one fracture in 365 patients (19.5%), corresponding to a number needed to screen (NNS) of 5.1. 696 fractures were detected (mean 1.9 per patient), most frequently at T12 and L1. According to Genant score, 34.6% were grade 1, 47.3% grade 2, and 18.1% grade 3; the NNS was 7.0 when considering only grade 2–3. Patients with vertebral fractures were older and had significantly lower BMD (all measured site: $p<0.05$).

After review of in-hospital medical records, fractures were considered previously unknown in only 8.0% of the 1,876 patients (NNS: 12.4), decreasing to 5.6% (NNS: 17.9) after extensive review including prior imaging and regional electronic datafiles. There was significantly less "unknown fracture" detection when age < 50 (NNS: 34.3 versus 11.5, p=0.0012), while difference was not significant with age 75 as a cut-off.

Identification of previously unknown fracture was associated with a potential therapeutic change in 91% of cases.

**Conclusions:** Systematic VFA shows a high apparent diagnostic yield. However, its true incremental yield for detecting previously unknown vertebral fractures is substantially lower after thorough medical record review. Nevertheless, identification of an unrecognized vertebral fracture has major clinical implications, strongly influencing treatment decisions. VFA may therefore rather serve as an important safety net to uncover clinically relevant fractures otherwise overlooked.

**Poster Sessions: Osteoporosis: pathophysiology and epidemiology**

**P161**

**Bone turnover markers and mortality in older men – the prospective STRAMBO study**

*Pawel Szulc*[1], *Roland Chapurlat*[2]
[1]*INSERM UMR 1033- University of Lyon- Hospices Civils de Lyon, Epidemiology of Osteoporosis, Lyon, France*
[2]*INSERM UMR 1033- University of Lyon- Hospices Civils de Lyon, Rhaumatology, Lyon, France*

**Abstract Text**

**Introduction:** Studies on the association between bone turnover markers (BTM) and mortality were focused on all-cause mortality and on specific populations (e.g., haemodialysis patients).

**Purpose:** To study the link between baseline BTM levels and cause-specific mortality in a cohort of prospectively followed home-dwelling older men.

**Methods:** We recruited 819 men aged 60–87 years. Serum PINP, osteocalcin, bone alkaline phosphatase, CTX-I and urinary total deoxypyridinoline (tDPD) were measured at baseline. Information on the death (date, cause) was confirmed by a health professional. Statistical models were adjusted for age, body composition, education level, co-morbidities, grip strength, glomerular filtration rate and abdominal aortic calcification.

**Results:** During the 8-year follow-up, 170 men died (median time to death: 58.5 months). Among them, 67 died from cardiovascular (CV) diseases, 70 from cancer and 40 from other causes (non-CV-non-cancer mortality). Higher levels of bone resorption markers were associated with higher all-cause mortality (CTX-I: HR=1.23 per SD, 95%CI: 1.06–1.43, p<0.01; tDPD: HR=1.37 per SD, 95%CI: 1.31–1.67, p<0.005). Using the log-likelihood chi-square statistics, tDPD was the third strongest predictor in the model ($\chi^2$=9.71, p<0.005). The association of bone formation markers with all-cause mortality was not statistically significant. Higher serum concentrations of all BTM were associated with higher non-

CV-non-cancer mortality (PINP: HR=1.60 per SD, 95%CI: 1.20–2.12, p<0.005; CTX-I: HR=1.62 per SD, 95%CI: 1.23–2.15, p<0.001). Higher urinary tDPD was also associated with increased non-CV-non-cancer mortality (HR=1.72 per SD, 95%CI: 1.17–2.52, p<0.005). CTX-I was the strongest predictor in the model compared with the confounders ($\chi^2$=12.10, p<0.001). BTMs were not associated with CV or cancer mortality.

**Conclusion:** Higher levels of bone resorption markers were associated with higher all-cause mortality in home-dwelling older men. All BTMs were predictive of higher non-CV-non-cancer mortality, but not of the CV or cancer mortality. Further studies are needed to elucidate the biological pathways explaining our observation.

**P162**

**Transforming Osteoporosis Care: measurable benefits of implementing a Fracture Liaison Service in a regional hospital**

*Montserrat Robustillo Villarino*[1], *Andrés Zúñiga Vera*[2], *Marile Sanabria Hernández*[2]
[1]*Hospital Universitari de la Plana, Rheumatology, Vila-Real, Spain*
[2]*Hospital de la Plana, Rheumatology, Vila-Real, Spain*

**Abstract Text**

**Background**: Fracture Liaison Services (FLS) have demonstrated effectiveness in reducing secondary fractures, improving treatment initiation, and optimizing follow-up of patients at high fracture risk. The FLS of Hospital Universitario de la Plana was implemented 18 months ago, providing an opportunity to evaluate early changes in fracture epidemiology and osteoporosis-related risk profiles.

**Objectives**: To describe temporal trends in patient characteristics, fracture types, and clinical risk factors before and after FLS implementation, using a stratified analysis by year in patients who presented and osteoporotic fracture.

**Methods:** A retrospective cohort was constructed from the hospital emergency registry covering **2018–2025**. **Only patients presenting with a clinical osteoporotic fracture** (hip, vertebral, humeral, Colles, or pelvic) were included. Patients <45 years were excluded. Annual strata were analyzed using descriptive statistics for continuous and dichotomous variables. Fracture incidence patterns were compared across years, with specific assessment of trends during the **18-month post-FLS period** (June 2024–December 2025).

**Results:** A total of **5,538 patients** were evaluated (4,602 pre-FLS; 936 post-FLS). Demographic and functional profiles remained stable across years. Documentation of classical osteoporosis risk factors improved after FLS implementation. Fracture distributions showed a relative reduction in hip and vertebral fractures post-FLS, alongside increased detection of peripheral fractures. Early indicators suggested lower-severity presentations and shorter hospital stays, consistent with strengthened secondary prevention pathways.

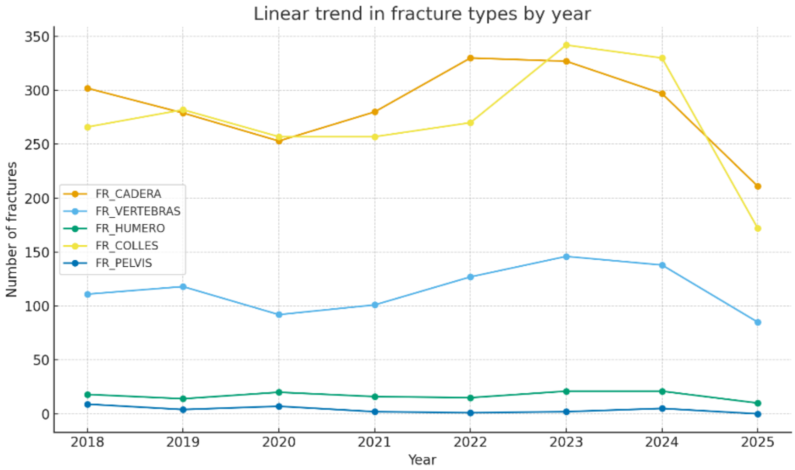

**Conclusions:** Introducing an FLS in June 2024 was associated with better risk-factor documentation, shifts in fracture patterns, and early signals of reduced major osteoporotic fractures. These findings support the positive early impact of the FLS and reinforce the need for continued long-term monitoring.

**P164**

**Influence of initial fracture site on subsequent fracture: a 5-year single centre Australian study**

*Lai-Ming Kathleen Pak*[1], *Michael Fogarty*[1], *Tung Pham*[2], *Tanita Botha*[1], *Hui Lyn Chin*[1], *Dhiraj Samarasinghe*[1], *Jacqueline Seiffert*[1], *Anna Kwok*[1], *Vivian Grill*[1], *Hanh H. Nguyen*[1]
[1]*Western Health, Department of Endocrinology and Diabetes, Melbourne, Australia*
[2]*Western Health, Performance Unit- Health Information Services, Melbourne, Australia*

**Abstract Text**

**Background:** An incident fragility fracture increases the risk of subsequent major osteoporotic fractures, with re-fracture risk varying by the site of the initial fracture.

**Purpose:** This study evaluates predictors of subsequent fracture risk in patients presenting with minimal-trauma fractures to an Australian tertiary hospital.

**Method:** Adults(age>50 years) presenting with fractures to Western Health between 2018-2022 were identified using emergency department and hospital ICD-10-AM codes. Skull/hand/foot, and trauma-related fractures were excluded. The re-fracture group comprised patients presenting ≥1 fracture(s) up to 2024. Demographics, fracture sites, and osteoporosis management data were extracted. Descriptive analyses were performed, and predictors of re-fracture were identified by comparing characteristics between the single-fracture and re-fracture groups.

**Results:** 8462 cases sustained 9386 fractures over the study period. Hip fractures were most common(n=1991), followed by rib fractures(n=1428). 860(10.2%) re-presented with ≥1 fracture(s); proximal hip(25.5%) and rib(17.8%) were the most frequent sites of re-fracture. Initial rib and hip fractures contributed the highest burden to the 1053 subsequent fractures(Figure 1). Initial vertebral, rib and pelvic fractures had the highest re-fracture rates(14.1%, 13.9%, and 12.6%, respectively). The re-fracture group was older [median(range)age: 79(69–86) vs. 74(63–84), p<0.0001], and had higher mortality rate (23.3% vs. 17.4%, p<0.0001) than the single-fracture group. European-born patients were more likely to re-fracture and Asian-born patients were less likely to re-fracture. Sex distribution and socio-economic status did not differ between groups. The median(range) duration between initial and subsequent fracture was 482.19(189.45–897.46)days [1.3(0.5–2.5)years]. At the time of re-fracture, 68% were not receiving osteoporosis therapy and 87% had not seen an osteoporosis specialist.

**Conclusion:** Significant treatment gaps were identified in the re-fracture cohort, underscoring the importance of osteoporosis interventions for all fracture types. In particular, rib fractures contributed one of the highest burdens of re-fracture presentations, with a re-fracture rate comparable to that of vertebral fractures in our study.

**Figure 1.**

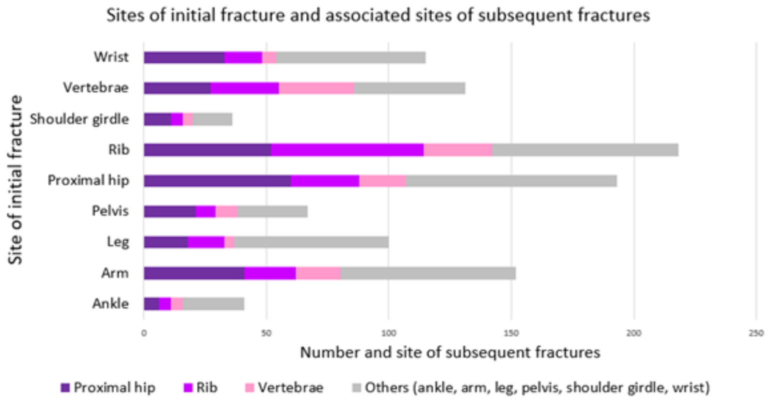

**P165**

**Association between low phosphate levels with fractures and cardiovascular events: a population based prospective study**

*Guido Cavati[1], Veronica Abate[2], Christian Mingiano[1], Anita Vergatti[2], Filippo Pirrotta[1], Marco Antonio Calabrese[1], Mattia Garofalo[1], Giuseppe Dipasquale[1], Mariangela Perazzo[1], Carla Caffarelli[1], Daniela Merlotti[1], Domenico Rendina[2], Luigi Gennari[1]*
*[1]University of Siena, Department of Medicine- Surgery and Neurosciences, Siena, Italy*
*[2]University of Naples Federico II, Department of Clinical Medicine and Surgery, Naples, Italy*

**Abstract Text**

Inorganic phosphate (P) is essential for several biological processes including bone mineralization and muscle function. Clinical and experimental evidences suggest that disturbances in P homeostasis may play a role in bone fragility, nephrolithiasis and cardiovascular disorders. However, the clinical consequences of low P levels (except phenotypes associated with hypophosphatemic rickets) on skeletal or cardiovascular health are often underestimated in clinical practice. To clarify this issue we assessed the relationship between serum P and incidence of fragility fractures and major cardiovascular events (MACE) in a population-based cohort of 1101 elderly men and women followed prospectively for up to 20 years (project code PNRR-POC-2023-12378009). All symptomatic fractures and MACE occurring between baseline (2004-2008) and December 2024 were recorded. P levels were measured at baseline and repeated for at least 2 times during the follow-up. At baseline 3.9% and 15.2% of subjects had P levels below 2.5 and 3.0mg/dl, respectively. Moreover 84% and 67% of subjects with P<2.5mg/dl and P<3.0mg/dl, respectively, maintained P levels below that thresholds at all time-points. Cox proportional regression models were used to examine the relationship of low P with incident fractures or MACE. Compared with reference group of subjects with normal P, subjects with baseline P<2.5mg/dl had a more than twofold increased risk to sustain a clinical fracture (HR 2.31, 95% CI:1.61-3.30, p<0.0001). Similar results were observed when cases with P below 2.5 at all time points, were only considered as well as after adjustment for age, body mass index, estimated GFR, serum levels of calcium, 25-hydroxyvitamin D, parathyroid hormone and hip-BMD. Moreover, subjects with P<2.5mg/dl at all time-points had a significantly increased prevalence of MACE (HR 2.49, 95% CI:1.05-5.90, p<0.05). In summary, this prospective study suggests that low serum P levels below 2.5 mg/dl might be predictive of fragility fractures and cardiovascular events in the general population.

**P166**

**Impact of COMT activity on bone homeostasis**

*<u>Malorie Cossez Guigne</u>[1], Isabelle Richard[2], gwenaëlle Jayat[1], Martine Cohen-Solal[1], Korng Ea[1], Catherine Baugé[3], Eric Haÿ[4]*
*[1]INSERM UMR1132/Université de Paris, bioscar, paris, France*
*[2]UR7451  Université de Caen Normandie, BioConnecT, Caen, France*
*[3]UR7451 Université de Caen Normandie, BioConnecT, Caen, France*
*[4]INSERM UMR1132/Université de Paris, bioscar, Paris 10ème arrondissement, France*

**Abstract Text**

Catecholamines are known to be mediators of stress-induced bone loss. Their degradation depends on the enzyme Catechol-O-Methyltransferase (COMT). A cohort studies have shown that a common variant Met158 of the COMT gene is associated with a 40% reduction in enzymatic activity compared to Val158. This variant is associated with an increased fracture risk in osteoporotic patients, although the mechanisms underlying this bone phenotype remain unclear.

The objectives of this study were to characterize the bone phenotype associated with the Val158 and Met158 variants and to dissect the underlying cellular mechanisms.

we used humanized mice carrying either the Met158 or Val158 variant. Bone microarchitecture was analyzed at 3, 6, 10, and 15 months by micro-CT, along with histological assessments. In vitro, osteoblastic (MC3T3) and osteoclastic (RAW) cell lines were differentiated in the presence or absence of Tolcapone, a COMT inhibitor.

*In vivo*, in the femur, trabecular bone mineral density and BV/TV were significantly lower in Met-variant compared with Val-variant between 3 and 10 months of age, (BV/TV 14% for Met VS 20% for Val P<0.05 at 6 months). TRAP staining revealed an increased number of osteoclasts in Met158 mice. However, cortical thickness increased (243,3µm for Met vs 232,2µm p<0.048). Immunofluorescent staining showed that the expression of COMT is similar between groups. *In vitro,* Tolcapone increased osteocalcin and alkaline phosphatase expression at day 7, at both the mRNA level and ALP activity. Furthermore, Tolcapone enhanced the osteoclastic differentiation of RAW cells compared with controls (2-fold increase in tolcapone-treated cells compared to control, P<0.05).

Together, these findings suggest that reduced COMT activity accelerates osteoblast differentiation while simultaneously increasing osteoclast differentiation, ultimately leading to a decrease in BV/TV. These data shed light in the mechanism of bone fragility observed in Met158 variant carriers.

**P167**

**Does sarcopenia aggravate the course of osteoporosis?**

*Daryna Kurylo[1], Nataliia Grygorieva[1], Nataliia Zaverukha[1]*
*[1]D.F. Chebotarev Institute of Gerontology of the National Academy of Medical Sciences of Ukraine, Department of Clinical Physiology and pathology of the musculoskeletal system, Kyiv, Ukraine*

**Abstract Text**

**Backgrounds:** Osteoporosis (OP) and sarcopenia (SP) are frequent age-related disorders. Their concurrence may contribute to increased fracture risk and substantial deterioration of functional status and quality of life.

**Objective:** To assess bone mineral density (BMD), fracture risk, and quality of life in patients with OP depending on the presence of SP.

**Material and Methods:** We examined 265 postmenopausal women (mean age 67.9±8.6 years) and divided them into two groups: 115 with OP (T-score ≤–2.5 SD, confirmed by DXA) and 45 with combined OP and SP (according to EWGSOP2). Anthropometric parameters were obtained using standard methods. The risk of major osteoporotic (MOF) and hip fractures (HF) was assessed with the Ukrainian FRAX®, quality of life – by the SarQol questionnaire, muscle strength – by handgrip dynamometry, and "sit-to-stand" test. BMD at the lumbar spine, total hip, and femoral neck, appendicular lean mass, and trabecular bone score (TBS) were measured by DXA (Hologic).

**Results:** Women with OP and SP exhibited significantly lower height, body weight, and BMI (p<0.0001); however, they did not differ in age and menopausal status compared to those with OP. They also showed reduced BMD at the lumbar spine (0.71±0.10 vs. 0.76±0.13 g/cm², p<0.05), total hip (0.67±0.11 vs. 0.73±0.10 g/cm², p<0.001), and femoral neck (0.54±0.08 vs. 0.59±0.08 g/cm², p<0.001) with no differences in distal radius BMD and TBS.

Patients with OP and SP exhibited significantly higher FRAX risk of MOF (FRAX BMI: p<0.05; FRAX BMD and FRAX BMD+TBS: p<0.00001 for both, respectively), and HF (FRAX BMI: p<0.05; FRAX BMD

and FRAX BMD+TBS: p<0.00001, respectively) compared with those with OP alone. However, fracture prevalence and SarQol scores did not significantly differ between the groups.

**Conclusion:** Lower BMD and higher 10-year probability of fracture risk in patients with OP and SP highlight the importance of detecting SP as a relevant component of comprehensive OP management.

**P168**

**Pathophysiology of osteoporosis (PATHOS Study): role of hidden cortisol excess (HidHyCo) and its predictors in bone fragility**

*Giorgia Grassi[1], Cristina Eller Vainicher[1], Elisa Cairoli[2], Romina Mirsepanj[3], Enrico Buccheri[4], Giuseppe Galesi[4], Flavia Pugliese[5], Guido Cavati[6], Alberto Ghielmetti[7], Anna Atlasova[3], Roberta Cosso[1], Carmen Aresta[8], Luca Giovanelli[9], Valentina Morelli[10], Vito Guarnieri[11], Giulia Casamassima[11], Francesco Sciacca[4], Orazio Palmieri[12], Francesca Tavano[12], Sabrina Corbetta[13], Alberto Falchetti[7], Luigi Gennari[6], Alfredo Scillitani[14], Agostino Gaudio[15], Iacopo Chiodini[16]*

*[1]Fondazione Cà Granda IRCCS Ospedale Maggiore Policlinico, Unit of Endocrinology, Milan, Italy*
*[2]ASST Lariana- Ospedale Sant'Anna, Unit of Endocrinology-Diabetology Dietetics and Clinical Nutrition, San Fermo della Battaglia, Italy*
*[3]University of Milan, Department of Clinical Sciences and Community Health, Milan, Italy*
*[4]Policlinico "G. Rodolico", Unit of Internal Medicine, Catania, Italy*
*[5]Fondazione IRCCS Ospedale Casa Sollievo della Sofferenza, Unit of Endocrinology, San Giovanni Rotondo, Italy*
*[6]University of Siena, Department of Medicine- Surgery and Neurosciences, Siena, Italy*
*[7]Ospedale Santa Maria della Misericordia, Unit of Endocrinology, Udine, Italy*
*[8]Unit of Endocrinology, ASST Grande Ospedale Metropolitano Niguarda, Milan, Italy*
*[9]Unit of Endocrinology and Diabetes, ASST Papa Giovanni XXIII, Bergamo, Italy*
*[10]IRCCS Istituto Auxologico Italiano, Bone Metabolic Diseases and Diabetes Unit- Department of Endocrine and Metabolic Diseases, Milan, Italy*
*[11]Fondazione IRCCS Casa Sollievo della Sofferenza, Division of Medical Genetics, San Giovanni Rotondo, Italy*
*[12]Fondazione IRCCS Casa Sollievo della Sofferenza, Unit of Gastroenterology, San Giovanni Rotondo, Italy*
*[13]University of Milan, Department of Biomedical- Surgical and Dental Sciences, Milan, Italy*
*[14]Fondazione IRCCS Casa Sollievo della Sofferenza, Unit of Endocrinology, San Giovanni Rotondo, Italy*
*[15]University of Catania, Department of Clinical and Experimental Medicine, Catania, Italy*
*[16]University of Milan, Department of Medical Biotechnology and Translational Medicine, Milan, Italy*

**Abstract Text**

**Purpose:** to assess the prevalence of hidden hypercortisolism (HidHyCo) in a sample of patients with osteoporosis or osteopenia plus the comorbidities possibly associated with cortisol excess and to evaluate the predictive characteristics.

**Methods:** in 5 Italian hospitals, we enrolled 1382 patients without clinically overt hypercortisolism or other secondary causes of osteoporosis, referred for evaluation of osteoporosis/fragility fractures (FX), or having osteopenia associated with ≥1 of: hypertension (HA) treated with≥2 drugs, diabetes mellitus (DM), history of cardiovascular events (CV). In all patients we collected data regarding FX, DM, HA and CV, the bone mineral density (BMD) measured with DXA at lumbar spine (LS) and femur, the presence of morphometric vertebral fractures (mVFX). All patients performed 1 mg overnight dexamethasone suppression test (DST), a cortisol cut-off ≥1.8 µg/dL was used to diagnose HidHyCo.

**Results:** HidHyCo was detected in 107 patients (7.7%). They were older (66.8±7.0 vs 63.2±7.6 years, p<0.001), had lower BMD (LS T-sc -3.0 ± 1.0 vs -2.8 ± 1.0, p=0.035; femural neck T-sc -2.5 ± 0.7 vs -2.3 ± 0.7, p=0.005; total hip T-sc -2.1 ± 0.7 vs -1.9 ± 0.8, p=0.003), higher prevalence of HA and HA treated with≥2 drugs (52.1 vs 33.6%, p<0.001 and 19.1 vs 9.7%, p=0.004, respectively), dyslipidemia (62.4 vs 46.8%, p=0.004), FX (73.6 vs 52.2%, p<0.001), major clinical FX (33.6 vs 18.9%, p<0.001), vertebral clinical FX (70.8 vs 51.5%, p<0.001) and mVFX (37.1 vs 27.7%, p=0.040) than non-HidHyCo group. At the logistic analyses, adjusted for age, sex, DM, dyslipidemia, the combination of FX and HA was associated with HidHyCo odds ratio, 2.4 [CI, 1.50 to 3.81], P <0.001).

**Conclusions:** HidHyCo may be more common than generally recognized in patients with bone fragility in whom other secondary causes have been excluded. The combination of FX and HA is associated with a higher risk of HidHyCo.

**P169**

**Validity of dual-energy x-ray absorptiometry for assessing visceral fat and its change after very low-calorie diet induced weight loss in obesity**

*<u>Ivan Raška</u>[1], Vit Zikan[1], Andrej Bocan[2], Monika Wágnerová[2], Martin Matoulek[1],*
*Radka Braunerova Taxova[3], Michaela Tencerova[4], Lukáš Lambert[5], Michaela Ferenčakova[4]*
*[1]General University Hospital- Faculty of Medicine 1- Charles University, Department of Internal Medicine 3, Prague, Czech Republic*
*[2]General University Hospital- Faculty of Medicine 1- Charles University, Department of Radiology, Prague, Czech Republic*
*[3]Institute of Endocrinology, Obesity Management Centre-, Prague, Czech Republic*
*[4]Institute of Physiology- Czech Academy of Sciences, Laboratory of Molecular Physiology of Bone, Prague, Czech Republic*
*[5]Motol University Hospital- Second Faculty of Medicine- Charles University, Dept. of Imaging Methods, Prague, Czech Republic*

**Abstract Text**

**Background:** Visceral adipose tissue (VAT) is a metabolically active organ whose excess accumulation increases the risk of metabolic syndrome, cardiovascular disease, cancer, and osteoporosis. Reliable methods to assess VAT and its longitudinal changes are therefore essential. This study aimed to evaluate: (1) the validity of dual-energy X-ray absorptiometry (DXA) compared with magnetic resonance imaging (MRI) for estimating VAT cross-sectionally and during very-low-calorie diet (VLCD)-

induced weight loss, and (2) the relationships between changes in bone mineral density (BMD), VAT, lipid profile, and insulin resistance.

**Methods:** Premenopausal women and men with and without obesity (n = 43; mean age 41.8 years; 88.4% women) underwent VAT assessment by DXA and MRI at baseline. A subset (n = 18) was reassessed after 6 months of dietary intervention consisting of 2 months of VLCD followed by 4 months of LCD. Intrahepatic lipids were measured by MRI, and lipid profile, HOMA-IR, and BMD were obtained by DXA. Associations between DXA and MRI VAT estimates were analysed using linear regression, and agreement was evaluated with Bland–Altman analysis.

**Results:** DXA-VAT correlated strongly with MRI-VAT at baseline (r = 0.88; p < 0.001) and for longitudinal changes (r = 0.79; p < 0.001). Bland–Altman analysis showed that DXA overestimated baseline VAT and underestimated VAT reduction. After 6 months, participants lost −13.1 % of initial body weight (p < 0.001), with VAT reductions of −25.2% by DXA and −30% by MRI (p < 0.001). HOMA-IR and lipid profiles improved significantly. Hip BMD declined significantly (p < 0.001), whereas lumbar spine BMD remained unchanged.

**Conclusions:** VLCD induced substantial reductions in body weight and VAT and improved metabolic parameters. Hip BMD decreased significantly during weight loss. Although DXA correlated strongly with MRI, it underestimated longitudinal VAT reductions.

**Keywords:** DXA, MRI, visceral adipose tissue, very-low-calorie diet **Funding:** MH CZ (NU23-01-00125)

**Poster Sessions: Osteoporosis: treatment**

**P170**

**Association between anti-fracture therapy prescription and one-year mortality after hip fracture: data from the Padua Fracture Liaison Service (FLS)**

*Gaetano Paride Arcidiacono[1], Marco Onofrio Torres[1], Mor Peleg Falb[1], Chiara Ceolin[2], Valentina Camozzi[3], Marina De Rui[2], Paolo Simioni[1], Giuseppe Sergi[2], Pietro Ruggieri[4], Sandro Giannini[1], Stefania Sella[1]*
*[1]University of Padua, Department of Medicine - Clinica Medica 1, Padua, Italy*
*[2]University of Padua, Geriatrics Division, Padua, Italy*
*[3]University Hospital of Padua, Endocrinology Unit, Padua, Italy*
*[4]University of Padua, Orthopedics and Orthopedic Oncology Unit, Padua, Italy*

**Abstract Text**

**Background:** Hip fractures (HF) are among the most severe—yet often undertreated—consequences of osteoporosis, carrying high risks of refracture, morbidity, and mortality. Fracture Liaison Service (FLS) models aim to improve case-finding of fragility-fracture patients and ensure systematic assessment with timely initiation of appropriate anti-fracture therapy. The aim of this study was to evaluate the association between anti-fracture therapy prescription and one-year refracture and allcause mortality following HF in an Italian real-world cohort, within a recently implemented FLS program.

**Methods:** We enrolled patients aged ≥50 years admitted with a fragility HF between March 2023 and March 2024 and assessed within the Padua University Hospital FLS. Clinical characteristics—including frailty as measured by the Multidimensional Prognostic Index (MPI)—and data on anti-fracture therapy prescriptions were collected during the index hospitalization. One-year follow-up after the index fracture was conducted by telephone calls or in-person visits.

**Results**: We included 554 patients (mean age 83±9 years; 71% women) with HF. Overall, 438 (79%) received or were prescribed anti-fracture therapy during hospitalization. Compared with untreated patients, treated patients had lower baseline frailty, with a median MPI of 0.38 (IQR 0.25–0.50) versus 0.56 (0.31–0.69); p<0.001. Treatment was associated with a reduced risk of one-year mortality in crude analyses and after inverse probability of treatment weighting (IPTW) adjustment for age, sex, and MPI (IPTW pseudo-population, n=1119; HR 0.62, 95% CI 0.41–0.95; p=0.028). No significant differences were observed for one-year refracture in crude or IPTW-adjusted analyses (overall one-year refracture rate 5%).

**Conclusions**: In one of the largest Italian FLS cohorts of HF patients, prescription of anti-fracture therapy was associated with lower one-year mortality. No difference in refracture risk was observed, which may be due to the short observation period. Longer follow-up is needed to determine the real-world impact of FLS-based treatment prescription on fracture-risk reduction.

**P171**

**Romosozumab and Microarchitecture: TBS Lumbar T-Score Detects Change Beyond BMD**

*Montserrat Robustillo Villarino*[1], *Andrés Zúñiga Vera*[1], *Marile Sanabria Hernández*[1]
[1]*Hospital Universitari de la Plana, Rheumatology, Vila-Real, Spain*

**Abstract Text**

**Background/Introduction:** Trabecular Bone Score (TBS) provides microarchitectural information complementary to bone mineral density (BMD). However, evidence on the responsiveness of TBS to romosozumab in real-world clinical settings remains limited.

Purpose: To evaluate and compare the sensitivity to change of TBS (index and lumbar T-score) and lumbar BMD after 12 months of romosozumab in a real-life cohort.

**Methods:** Postmenopausal women with osteoporosis who completed 12 months of romosozumab and underwent paired baseline and annual DXA scans were included. Outcomes were absolute and percentage changes in: (1) TBS index, (2) TBS lumbar T-score, and (3) lumbar BMD. Responsiveness was assessed using paired t-tests and Standardized Response Means (SRM). Correlations between change in TBS and change in BMD were evaluated using Pearson's r. Ethical approval and written informed consent were obtained.

**Results:** A total of 47 participants had paired TBS index values, 48 had paired TBS T-scores and 45 had paired lumbar BMD measurements. TBS index increased modestly (+0.016; +1.7%), without statistical significance (p=0.295) and with low responsiveness (SRM=0.15). TBS lumbar T-score showed a markedly greater improvement (+1.03), reaching high responsiveness (SRM=1.43, p<0.001). Lumbar BMD increased by +0.070 g/cm² (+10.5%), demonstrating strong responsiveness (SRM=1.34, p<0.001). Correlation between change in TBS index and change in lumbar BMD was minimal (r≈0.01), whereas change in TBS T-score correlated moderately with BMD improvement (r≈0.53). A summary table with descriptive changes are provided.

| Metric | Baseline | Annual | Change | % | p-value | SRM |
|---|---|---|---|---|---|---|
| TBS index | 1.136 | 1.154 | +0.016 | 1.7% | 0.295 | 0.15 |
| Lumbar TBS T-score | −4.41 | −3.37 | +1.04 | — | <0.001 | 1.43 |
| Lumbar BMD (g/cm²) | 0.699 | 0.771 | +0.071 | 10.2% | <0.001 | 1.34 |

**Conclusions:** In this real-world analysis, romosozumab produced robust increases in lumbar BMD and TBS lumbar T-score, while the TBS index showed limited sensitivity to change. TBS lumbar T-score exhibited strong responsiveness and appears to be a more reliable parameter for monitoring microarchitectural effects of romosozumab in clinical practice.

**P172**

**Romosozumab Super-Responders in Clinical Practice: Insights from a Large Italian Multicenter Cohort**

*Cristina Eller Vainicher[1], Alberto Ghielmetti[2,3], Giorgia Grassi[3], Silvia Carrara[4], Andrea Palermo[5,6], Anda Naciu[5,6], Eleonora Sargentini[5,6], Antonio Salcuni[2], Fabio Vescini[2], Marta Zampogna[3], Anna Atlasova[3,7], Sara Rondinella[7,8], Roberta Cosso[3], Iacopo Chiodini[9,10], Vittoria Favero[9,10], Massimiliano Mazza[7,10], Stefano Frara[4,11], Giovanna Mantovani[3,7], Sabrina Corbetta[4,12]*
*[1]Unit of Endocrinology, Fondazione Cà Granda IRCCS Ospedale Maggiore Policlinico, Milan, Italy*
*[2]Unit of Endocrinology and Metabolism, University Hospital Santa Maria della Misericordia, Udine, Italy*
*[3]Endocrine Unit, Fondazione IRCCS Ca' Granda Ospedale Maggiore Policlinico, Milan, Italy*
*[4]Unit of Bone Metabolism and Diabetes,*
*Department of Endocrinology and Metabolic Diseases- IRCCS Istituto Auxologico Italiano, Milan, Italy*
*[5]Unit of Bone Mineral and Thyroid Diseases, Fondazione Policlinico Universitario Campus Bio-Medico, Rome, Italy*
*[6]Unit of Endocrinology and Diabetes, Fondazione Policlinico Universitario Campus Bio-Medico, Rome, Italy*
*[7]Department of Clinical Sciences and Community Health – Dipartimento di Eccellenza 2023–2027, University of Milan, Milan, Italy*
*[8]Endocrine Unit, Fondazione Ca' Granda IRCCS Ospedale Maggiore Policlinico, Milan, Italy*
*[9]Department of Medical Biotechnology and Translational Medicine, University of Milan, Milan, Italy*
*[10]Endocrinology Unit, ASST Grande Ospedale Metropolitano Niguarda, Milan, Italy*
*[11]Department of Life Science, Health and health Professions- Università degli Studi Link, Rome, Italy*

[12]*Department of Biomedical- Surgical and Dental Sciences, University of Milan, Milan, Italy*

**Abstract Text**

**Background:** Real-world evidence on romosozumab is expanding, yet limited data describe high-magnitude response variability or the clinical determinants of "super-responder" phenotypes.

**Objective:** To evaluate the effectiveness of romosozumab in a large Italian multicentre cohort and identify predictors of high-magnitude densitometric response.

**Methods:** We retrospectively analysed postmenopausal women who completed 12 months of romosozumab across five Italian centres. DXA scans were performed at baseline and after treatment. Clinical history, morphometric and clinical fractures, and prior anti-osteoporotic therapies were collected. "Super responders" were defined as achieving ≥20% 20% BMD gain. Multivariate logistic regression identified independent predictors.

**Results:** A total of 149 women (mean age 69.4 ± 9.9 years) were included. Prevalent vertebral, femoral, and non-vertebral fractures were present in 85.2%, 30.9%, and 49.0% of participants, respectively; 82% had received previous therapy. Mean BMD gains were +13.7% at the lumbar spine (LS), +5.9% at the femoral neck (FN), and +4.4% at the total hip (TH). LS super-response (17.7%) was independently predicted by higher baseline CTX (OR 1.42; p=0.018), younger age (OR 1.1, p=0.036), and lower LS T-score (OR 0.29, p=0.003). FN super-response (5.9%) was strongly associated with prior femoral fracture (OR 14.6; p=0.020). A ≥10% TH increase was predicted by prior femoral fracture (OR 3.74; p=0.042) and lower TH T-score (OR 0.40, p=0.037).

**Conclusions:** Romosozumab was highly effective in this real-world cohort, with a substantial proportion of super-responders despite extensive prior treatment. Distinct predictors emerged at different skeletal sites, with turnover driving lumbar gains and prior femoral fracture identifying those most responsive at cortical-rich regions, supporting a differentiated biological basis for high-magnitude response.

**P173**

### Integrating Bone Quality and Systemic Health: Romosozumab-Driven Microarchitectural Gains and Their Relationship With Inflammation and Cardiovascular Risk

*Montserrat Robustillo Villarino*[1], *Andrés Zuñiga Vera*[1], *Marile Sanabria Hernández*[1]
[1]*Hospital Universitari de la Plana, Rheumatology, Vila-Real, Spain*

**Abstract Text**

**Background/Introduction:** Trabecular Bone Score (TBS) provides complementary information on trabecular microarchitecture, whereas bone mineral density (BMD) reflects bone quantity. Inflammatory ratios such as neutrophil-to-lymphocyte (NLR), platelet-to-lymphocyte (PLR), and monocyte-to-lymphocyte (MLR) are recognised biomarkers of subclinical inflammation and surrogate indicators of cardiovascular risk (CVR). Given ongoing debate about the cardiovascular safety of romosozumab, it is relevant to determine whether this anabolic agent modifies inflammatory markers linked to vascular risk. No previous real-world study has evaluated changes in these biomarkers under romosozumab.

**Purpose:** To assess changes in TBS, BMD, densitometric T-scores and inflammatory ratios after 12 months of romosozumab, and to determine whether treatment influences markers of subclinical inflammation associated with CVR.

**Methods:** A total of **60 postmenopausal women** with paired baseline and 12-month DXA scans were included. Mean age was **66.6 ± 9.5 years**. Comorbidities included **hypertension (43%)**, **dyslipidaemia (38%)**, **diabetes mellitus (3%)** and **inflammatory diseases (17%)**. Previous fractures were present in **42%**. Prior osteoporosis treatments included **bisphosphonates (52%)**, **denosumab (23%)**, and **teriparatide (8%)**. Outcomes were changes in TBS index, lumbar TBS T-score, lumbar and femoral BMD, and corresponding T-scores; NLR, PLR and MLR were assessed at baseline and at 12 months.

**Results:** Lumbar BMD increased from 0.699 to 0.771 g/cm² (**+10.2%**), lumbar TBS T-score improved by **+1.04**, and TBS index increased by **+1.7%**. Baseline inflammatory ratios did not showed associations with ΔT-score or ΔBMD but baselina NLR moderately correlated with ΔTBS (r≈−0.63). Importantly, inflammatory ratios remained **stable over 12 months**, showing no evidence of systemic inflammatory activation.

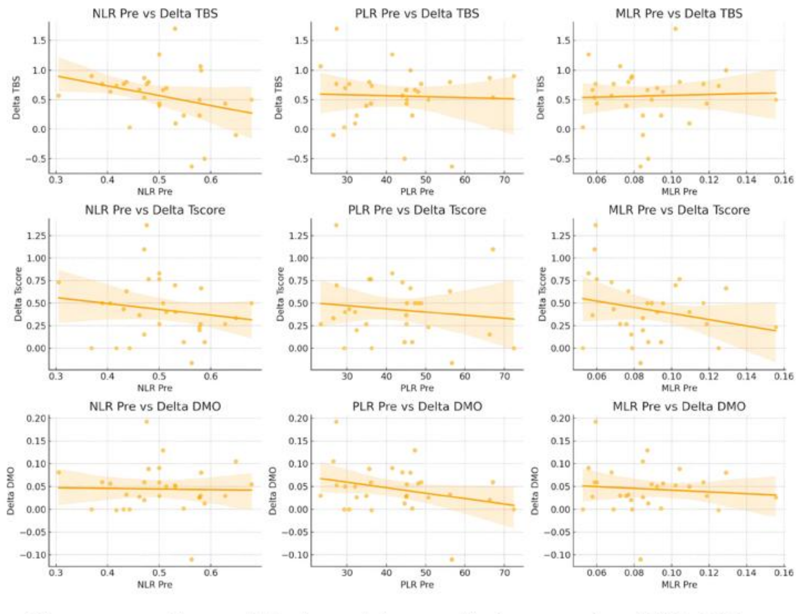

Linear regression models showed **low predictive capacity** of NLR, PLR, or MLR on changes in TBS, T-score, or BMD ($R^2 < 0.1$ in all models)

**Conclusions:** Romosozumab produced substantial anabolic effects on bone without altering inflammatory ratios linked to CVR. These findings support that romosozumab exerts a **purely anabolic mechanism**, maintains **stable subclinical inflammation**, and shows a **neutral cardiovascular profile** in real-world practice.

**P174**

**The association between low vitamin D and coronary calcifications**

_Sekib Sokolovic_[1]
[1]*Plava Medical Group, Rheumatology and Osteoporosis, Sarajevo, Bosnia - Herzegovina*

**Abstract Text**

**Background:** Vitamin D deficiency has been associated with adverse cardiovascular outcomes, including potential contributions to coronary artery calcification (CAC). However, clinical characterizations of patients presenting with both conditions remain limited. This case series describes the clinical features, laboratory profiles, and imaging findings of patients with vitamin D deficiency who were found to have coronary calcifications on imaging.

**Methods:** We conducted a retrospective case series of nine patients evaluated at a tertiary cardiorheumatology clinic between who had (1) serum 25-hydroxyvitamin D levels <20 ng/mL and (2) evidence of coronary calcification on non-contrast cardiac CT. Data collected included demographics, cardiovascular risk factors, vitamin D levels, hsCRP CAC scores, and clinical outcomes over follow-up. Descriptive statistics were used.

**Results:** Nine patients (mean age 62 ± 9 years; all postmenopausal females met inclusion criteria. All patients had vitamin D deficiency, with mean 25(OH)D levels of 12.4 ± 3.1 ng/mL. The median Agatston CAC score was 225 (range 64–585). The most common comorbidities were hypertension (75%), dyslipidemia (62%), and prediabetes or diabetes (50%). Five patients presented with exertional chest discomfort and had obstructive coronary LAD disease with ~70%, four were asymptomatic and identified incidentally. Vitamin D supplementation was initiated in all cases. HsCRP average was 4,5mg/l. The higher hsCRFP level, the more severe coronary calcium score.

**Conclusions:** This case series highlights a clinical pattern in which patients with vitamin D deficiency frequently exhibit moderate to severe CAC and share multiple cardiometabolic risk factors. While this descriptive analysis cannot establish causality, but demonstrate that inflammation plays significant role in ethiopatogenesis. This ephasize the potential interplay between vitamin D status and coronary calcification and supports the need for controlled studies to clarify their relationship and clinical significance.

**P175**

**Alendronate following denosumab discontinuation in postmenopausal women**

_Polyzois Makras_[1], *Athanasios Anastasilakis*[2], *Palermo Andrea*[3], *Anda Mihaela Naciu*[4], *Stergios Polyzos*[5], *Danai Georgakopoulou*[6], *Athanasios Papatheodorou*[7], *Mathaios Savvidis*[8], *Socrates Papapoulos*[9], *Maria Yavropoulou*[10]
[1]*251 Hellenic Air Force & VA General Hospital-,*

*Department of Endocrinology and Department of Medical Research, Athens, Greece*
*[2]424 General Military Hospital, Department of Endocrinology, Thessaloniki, Greece*
*[3]Fondazione Policlinico Universitario Campus Bio-Medico,*
*Unit of Metabolic bone and thyroid disorders, Rome, Italy*
*[4]FondazionPoliclinico Universitario Campus Bio-Medico, Unit of Metabolic bone and thyroid disorders, Rome, Italy*
*[5]School of Medicine- Aristotle University of Thessaloniki, First Laboratory of Pharmacology, Thessaloniki, Greece*
*[6]251 Hellenic Air Force & VA General Hospital, Department of Endocrinology and Diabetes, Athens, Greece*
*[7]251 Hellenic Air Force & VA General Hospital, Department of Medical Research, Athens, Greece*
*[8]424 General Military Hospital, 1st Department of Orthopedics, Thessaloniki, Greece*
*[9]Leiden University Medical Center, Center for Bone Quality, Leiden, Netherlands*
*[10]National and Kapodistrian University of Athens- Medical School,*
*Endocrinology Unit- First Department of Propaedeutic and Internal Medicine, Athens, Greece*

**Abstract Text**

**Purpose**: To prospectively assess the efficacy of alendronate (ALN) to prevent the overshoot of bone turnover markers (BTMs) and loss of BMD in postmenopausal osteoporotic (PMO) women stopping denosumab (Dmab) treatment.

**Methods**: One-year prospective, observational clinical trial of PMO patients previously treated with Dmab sc injections 60 mg 6-monthly who reached LS and FN BMD T-scores between −2.5 and −1.0. Patients received ALN 70 mg once weekly for 6 mo followed by 6 mo without medication (Group 1) or for 12 mo (Group 2). All received vitamin D and calcium supplements. The study's primary endpoint was the change of LS BMD from baseline to month 12.

**Results:** Eighty patients (39 Group 1, 41 Group 2) who had received 2 to 22 Dmab injections (mean 9.8) completed the study. Median age was 67 years, 22.7% had prevalent fractures and 59% had previously received antiosteoporotic treatments other than Dmab. Compared to baseline, median LS BMD decreased significantly in month 12 in the total cohort [-5.9% (8.2%), p<0.001] and this decrease was not different between the two groups [Group 1: -6.8% (8.0%) Vs Group 2: -5.0% (8.6%), p=0.50]. Similarly, FN BMD decreased significantly in month 12 by -3.5% (6.3%) (p<0.001) with no significant difference between the two groups. Serum P1NP, CTX and TRAcP5b increased significantly in month 12 with no differences in percentage changes between the two groups. However, values of serum P1NP were significantly higher in Group 1 compared to Group 2 both at 9 and 12 months. Two patients, one in each group sustained clinical fractures (hip and multiple vertebral) and one patient from Group 2 had a new asymptomatic vertebral fracture.

**Conclusions** Alendronate partially mitigates bone loss and bone turnover marker overshoot following denosumab discontinuation in PMO women. Six months were as effective as twelve months of alendronate treatment.

**P176**

### Inadequate response to Denosumab: differences between patients with incidental fractures and/or bone loss during treatment

*Clara Churtichaga Domenech[1], Judith Vidal-Ripoll[1], Francina Salabert-Carreras[1], Cristina Rocamora-Gisbert[1], Raquel Ugena-García[1], Cristina Calomarde-Gómez[1], Annika Nack[1], Águeda Prior-Español[1], Susana Holgado-Pérez[1], Clara Sangüesa[2], Lourdes Mateo-Soria[1], Laia Gifre[1]*
*[1]Hospital Universitari Germans trias i Pujol, Rheumatology, Badalona, Spain*
*[2]Hospital Severo Ochoa, Rheumatology, Madrid, Spain*

**Abstract Text**

**Introduction:** The definition of inadequate response (IR) to denosumab remains unclear, limiting clinical decision-making. This study aims to characterise patients presenting bone mineral density (BMD) loss and/or incidental fractures during treatment.

**Methods:** Retrospective study of 37 patients with IR, defined as lumbar or femoral BMD loss >3% and/or incidental fracture. Clinical variables (age, BMI, fracture history, prior bisphosphonates), CTx, and BMD during treatment were collected. Patients with poor adherence were excluded. Patients were classified as IR-fracture, IR-BMD, or IR-both.

**Results:** Table1 summarises the main characteristics. Incidental fractures occurred in 25 patients: vertebral(n=15), femur(n=5), other(n=5). 20% had multiple fractures. 25% had glucocorticoid-induced osteoporosis. IR-fracture patients showed a higher prevalence of previous vertebral fractures (p=0.011) and tended to be older, with lower femoral BMD and higher prevalence of glucocorticoid-induced osteoporosis. IR-BMD patients showed earlier IR and shorter prior bisphosphonate exposure. IR-both showed later IR, longer prior bisphosphonate exposure, and lower lumbar BMD. All patients showed higher-than-expected CTx values during denosumab.

**Conclusion:** Glucocorticoid treatment and elevated CTx may be associated with IR to denosumab. Baseline vertebral fractures, age, and BMD appear relevant, particularly in IR-fracture group.

Table1:

| **Variable** | **Total(n=37)** | **IR-fracture(n=19)** | **IR-BMD(n=12)** | **IR-both(n=6)** | **p-value*** |
|---|---|---|---|---|---|
| **Age(years)(SD)** | 72.72(10.10) | 76.29(9.23) | 68.31(10.39) | 70.27(9.40) | 0.078 |
| **Duration of denosumab (months)(SD)** | 37.02(28.30) | 39.47(34.15) | 29.43(16.46) | 44.46(27.25) | 0.504 |
| **BMI (kg/m²)(DE)** | 28.09(6.15) | 28.09(5.24) | 28.67(8.59) | 26.93(2.95) | 0.858 |
| Postmenopausal | 20(54.05%) | 11(57.89%) | 6(50%) | 3(50%) | 0.252 |
| Glucocorticoid-induced | 9(24.3%) | 6(31.58%) | 3(25%) | 0(0%) | 0.252 |

| Variable | Total(n=37) | IR-fracture(n=19) | IR-BMD(n=12) | IR-both(n=6) | p-value* |
|---|---|---|---|---|---|
| Other | 8(21.62%) | 2(10.53%) | 3(25%) | 3(50%) | 0.252 |
| **Number of previous fractures(SD)** | 2.50(2.06) | 2.33(1.59) | 2.86(2.61) | 2.50(2.74) | 0.867 |
| **Previous vertebral fracture, n(%)** | 25(67.57%) | 17(89.47%) | 6(50%) | 2(33.33%) | **0.011** |
| **Prior bisphosphonates, n(%)** | 22(59.46%) | 12(63.16%) | 6(50%) | 4(66.67%) | 0.711 |
| **Years on bisphosphonates(SD)** | 3.35(3.74) | 3.00(3.20) | 2.92(3.60) | 5.33(5.43) | 0.375 |
| **Femoral neck BMD(g/cm²)(SD)** | 0.723(0.097) | 0.691(0.083) | 0.766(0.071) | 0.726(0.153) | 0.126 |
| **Lumbar spine BMD(g/cm²)(SD)** | 0.841(0.202) | 0.853(0.166) | 0.877(0.126) | 0.737(0.371) | 0.374 |
| **Total femur BMD(g/cm²)(SD)** | 0.722(0.097) | 0.685(0.077) | 0.767(0.066) | 0.733(0.153) | 0.115 |
| **Baseline CTx(ng/mL)(SD)** | 0.159(0.137) | 0.147(0.141) | 0.172(0.129) | 0.174(0.190) | 0.941 |

*p-values obtained from chi-square or one-way ANOVA. Results expressed as mean(SD) or n(%).

**P177**

**Reduced efficacy of oral bisphosphonates is associated with concurrent proton pump inhibitor use**

*Cecilia Oliveri*[1], *Erika Nardo*[1], *Nunziata Morabito*[1], *Antonino Catalano*[1]
[1]*University of Messina, Clinical and Experimental Medicine, Messina, Italy*

**Abstract Text**

**Background:** Proton pump inhibitors (PPIs) are widely prescribed for acid-related disorders through inhibition of the gastric $H^+/K^+$-ATPase pump, leading to reduced gastric acid secretion. Growing evidence has raised concerns about the long-term skeletal effects of PPIs, with several studies reporting an increased risk of fragility fractures, particularly in older adults. Oral bisphosphonates (BPs) are first-line antiresorptive agents for osteoporosis and effectively reduce fracture risk, yet their therapeutic response may be influenced by factors affecting gastrointestinal absorption. The potential interaction between chronic PPI use and BP efficacy remains insufficiently characterized.

**Purpose:** To evaluate changes in bone mineral density (BMD) in postmenopausal women treated with oral BPs, stratified by concomitant PPI use.

**Methods:** We conducted a retrospective cohort study of postmenopausal women at high fracture risk who initiated oral BP therapy (alendronate, risedronate, or ibandronate). The cohort included both chronic PPI users—defined as continuous therapy with omeprazole, esomeprazole, lansoprazole, pantoprazole, or rabeprazole for ≥12 months—and non-users. All participants were bisphosphonate-naïve at baseline. BMD was assessed by dual-energy X-ray absorptiometry at baseline and after a median follow-up of 24 months.

**Results:** A total of 180 women (mean age 61.2 years) were enrolled, including 60 PPI users. At follow-up, women receiving concomitant PPIs and BPs exhibited significantly lower mean percentage change in lumbar spine BMD compared to non-users (−0.17 ± 7.4% vs. 2.8 ± 6.8%). In stepwise multiple regression analysis, PPI use emerged as an independent predictor of reduced lumbar spine BMD gain ($\beta = -2.87$, SE = 1.34, $p = 0.03$).

**Conclusion:** Long-term PPI therapy may attenuate the anabolic response to oral bisphosphonates in postmenopausal women with severe osteoporosis. When clinically feasible, reevaluating chronic PPI therapy may help optimize treatment outcomes in osteoporosis management.

**P178**

**Denosumab Exit Strategy in Glucocorticoid-Induced Osteoporosis: A Multi-site Randomized Clinical Trial**

*Giovanni Adami[1], Gary Cutter[2], Rahima Begum[2], Maurizio Rossini[1], Davide Gatti[1], Kenneth Saag[2], Maria Danila[2]*
*[1]University of Verona, Rheumatology Unit, Verona, Italy*
*[2]University of Alabama at Birmingham, Division of Clinical Immunology and Rheumatology, Birmingham, United States*

**Abstract Text**

**Background:** Prompt anti-resorptive treatment with bisphosphonates after denosumab discontinuation is generally recommended. Glucocorticoid users may not require longer-term denosumab and are at greater fracture risk. Herein, we test different exit strategies in patients on chronic glucocorticoids who discontinued DMAB.

**Methods:** We conducted an open-label, randomized, multicenter, clinical trial in which participants on DMAB 60 mg were assigned 1:1:1 to weekly oral alendronate (ALN) started 6 months after last DMAB dose, OR 1 dose of IV zoledronic acid 6 months after last DMAB (EARLY ZA) OR 1 dose of IV zoledronic acid 9 months after last DMAB (LATE ZA). Inclusion criteria: pts on prednisone ≥7.5 mg /day; baseline BMD T-score of ≤ -1.0. Exclusion criteria: no past BPs, >24 months of prior DMAB treatment.

**Results:** A total of 45 patients were randomized. Baseline characteristics are reported in **Table 1**. **Figure 1 adn figure 2** shows the BTMs and BMD data by group. At 6 months, we found a non-significant trend for change in CTX in the LATE ZA compared to ALN (p=0.07), but there was no difference in CTX between the LATE ZA and EARLY ZA groups (p=0.12). However, at 3 months there was a significant difference in the change in CTX between LATE ZA vs ALN (p=0.0182) but not between LATE ZA and EARLY ZA (p=0.757). For lumbar spine BMD, at 6 months, we observed a non-significant trend between LATE ZA and EARLY ZA (p=0.059). At 12 months, there was a statistically significant difference in BMD between LATE ZA and ALN (p=0.04), but not between LATE ZA and EARLY ZA (p=0.11).

**Conclusion:** Among those receiving delayed ZA, an escape in bone turnover at 3 months could create a window for fractures. However, it could be offset by greater BMD gain at 6 months, which, however, was not sustained at 12 months.

| Characteristics | Alendronate (70 mg. 6 months after the last denosumab dose) n=15 | Early ZA (5 mg. 6 months after the last denosumab dose) n=14 | Late ZA (5 mg. 9 months after last denosumab dose) n=16 |
|---|---|---|---|
| **Age in yrs., Mean (SD)** | 65.4 (10.56) | 66.8 (11.81) | 58.3 (14.91) |
| **Sex, Female, N (%)** | 11 (73.33) | 9 (64.29) | 11 (73.33) |
| **Enrollment site, N (%)** | | | |
| Birmingham (AL, USA) | 7 (46.67) | 6 (42.86) | 7 (43.75) |
| Verona (Italy) | 8 (53.33) | 8 (57.14) | 9 (56.25) |
| **Race, N (%)** | | | |
| African American/Black | 1 (6.67) | 1 (7.14) | 5 (31.25) |
| Asian | 1 (6.67) | 0 (0.00) | 1 (6.25) |
| White | 12 (80.00) | 13 (92.86) | 10 (62.50) |
| Other | 1 (6.67) | 0 (0.00) | 0 (0.00) |
| **Ethnicity, N (%)** | | | |
| Hispanic/Latino | 1 (6.67) | 1 (7.14) | 0 (0.00) |
| Not Hispanic/Latino | 14 (93.33) | 13 (92.86) | 18 (100.00) |
| Rheumatic Disease, N (%) | 15 (100) | 14(100) | 16 (100) |
| **BMD lumbar-spine T-score at Randomization, Mean (SD)** | -1.33 (1.51) | -1.17 (0.98) | -0.83 (0.94) |
| **BMD Total Hip T-score at Randomization, Mean (SD)** | -1.59 (0.91) | -1.19 (1.12) | -0.57 (0.85) |
| **BMD femoral neck T-score at Randomization, Mean (SD)** | -2.06 (0.87) | -1.71 (1.00) | -1.02 (1.03) |
| **CTX at baseline (ng/mL), Mean (SD)** | 0.07 (0.06) | 0.05 (0.05) | 0.11 (0.16) |
| **P1NP at baseline (ng/mL), Mean (SD)** | 31.04 (20.66) | 23.24 (11.64) | 45.35 (56.92) |

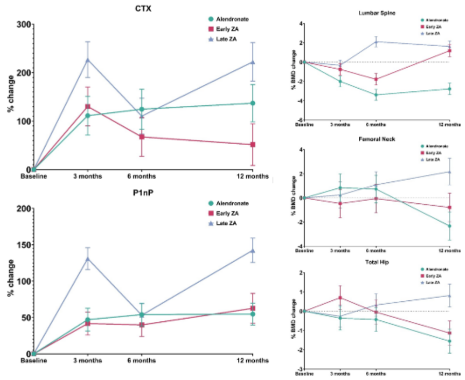

**P179**

**Romosozumab in Post-Menopausal Women with Multiple Myeloma, a Prospective 12-Month Study**

*Giovanni Adami*[1], *Mariana Diz Lopes*[1,2], *Martina Tinelli*[3], *Elena Marchetti*[3], *Davide Gatti*[1], *Maurizio Rossini*[1]
[1]*University of Verona, Rheumatology Unit, Verona, Italy*
[2]*Unidade Local de Saúde de São João, Rheumatology Unit, Porto, Portugal*
[3]*University of Verona, Hematology Unit, Verona, Italy*

**Abstract Text**

**Background:** Bone damage occurs early in the progression of plasma cell disorders. Romosozumab (ROMO), a sclerostin inhibitor with both anabolic and antiresorptive effects, may provide therapeutic benefit for this fracture-prone population.

**Methods:** We conducted a 12-month prospective observational study on postmenopausal women with osteoporosis and multiple myeloma (MM) treated with monthly ROMO. Bone mineral density (BMD) was assessed at baseline, 6, and 12 months with dual-energy X-ray absorptiometry (DXA) and high-resolution peripheral quantitative tomography (HR-pQCT), bone turnover makers (BTMs) and several MM markers were also monitored.

**Results:** Eight female patients with MM without evidence of CRAB completed 12 months of ROMO. A significant BMD increase was observed at the lumbar spine (+5.8%, p=0.048), femoral neck (+4.2%, p=0.020), and total hip (+3.3%, p=0.002). P1NP rose sharply at month 3 (+117.8%) and returned to baseline by month 12 (p=0.006). CTX decreased progressively, showing a trend towards significance (−53.6%, p=0.060). ALP and B-ALP significantly declined by month 12 (p=0.020 and p=0.022). No significant changes occurred in immunoglobulins, M-protein, or light chains. β2-microglobulin decreased at month 12 (from 2.35 to 2.1mg/L, p=0.042) but the overall trend was not significant (p=0.092). Failure load increased at month 6 (p=0.033) but not at month 12 while the other HR-pQCT parameters remained stable throughout the study.

**Conclusion:** In this exploratory study of postmenopausal women with MM and osteoporosis, ROMO significantly improved BMD and modulated BTMs, without any evidence of disease progression over the 12-month period. These findings support ROMO as a potential bone-targeted therapy in patients with osteoporosis and MM.

| | M0 | M6 | M12 | p-value |
|---|---|---|---|---|
| **Laboratory Parameters** | | | | |
| **Hb (g/dL), median (IQR)** | 12.5 (11.6-12.8) | 12.2 (11.6-12.5) | 12.2 (11.2-12.7) | 0.397 |
| **RBC count (10³/µl), median (IQR)** | 3.9 (3.6-4.1) | 3.9 (3.7-3.9) | 3.7 (3.5-4) | 0.293 |
| **WBC/µl, median (IQR)** | 5.8 (4.9-6.9) | 5.8 (5.1-8.1) | 5.8 (4.7-6.9) | 0.522 |
| **Platelets (10³/µl), median (IQR)** | 249.5 (223.8-331.8) | 248.5 (237.3-278.5) | 263.5 (241.5-304.0) | 0.320 |
| **25 (OH) vitamin D (ng/mL), median (IQR)** | 27.5 (26.0-45.9) | 43 (33.0-44.6) | 45.4 (35.9-56.1) | 0.090 |
| **PTH (pmol/L), median (IQR)** | 5.2 (4.1-5.5) | 4.7 (4.3-5.3) | 6.0 (4.1-6.4) | 0.468 |
| **ALP (U/L), median (IQR)** | 68.5 (61.3-73.3) | 67.5 (57.8-79.3) | 59 (52-66.5) | **0.020** |
| **B-ALP (ng/mL), median (IQR)** | 13.5 (9.3-17.0) | 13 (10.5-15.0) | 9.0 (6.0-12.0) | **0.037** |
| **Ca (mg/dL), median (IQR)** | 9.4 (9.3-9.7) | 9.4 (9.1-9.5) | 9.3 (9.2-9.6) | 0.495 |
| **Pi (mg/dL), median (IQR)** | 3.6 (3.4-3.7) | 3.3 (3.1-3.6) | 3.4 (3.1-3.9) | 0.282 |
| **Cr (mg/dL), median (IQR)** | 0.8 (0.6-0.9) | 0.9 (0.6-1.0) | 0.9 (0.6-1.0) | 0.122 |
| **GFR (mL/min/1.73 m²), median (IQR)** | 84.5 (65-93) | 79.5 (62-94) | 72.5 (59.3-92.8) | 0.324 |
| **IgG (g/L), median (IQR)** | 27.1 (23-31.8) | 29.8 (24.1-33.3) | 30.2 (26.2-33.2) | 0.460 |
| **IgA (g/L), median (IQR)** | 0.3 (0.3-0.5) | 0.3 (0.3-0.5) | 0.4 (0.3-0.6) | 0.307 |
| **IgM (g/L), median (IQR)** | 0.4 (0.3-0.4) | 0.4 (0.3-0.5) | 0.4 (0.3-0.5) | 0.518 |
| **M-protein (g/L), median (IQR)** | 22.8 (18-27.3) | 22.5 (18.8-25.8) | 23.3 (20.0-27.3) | 0.060 |
| **Serum FLC: λ (mg/L), median (IQR)** | 17.3 (7.6-43.3) | 20.8 (9.1-75.4) | 15.9 (10.1-47.5) | 0.895 |
| **Serum FLC: K (mg/L), median (IQR)** | 90.9 (10.9-239.4) | 98.2 (10.5-249.6) | 97.9 (9.8-411.8) | 0.181 |
| **Serum FLC ratio (K/λ), median (IQR)** | 6.9 (0.3-40.8) | 8.1 (0.4-50.0) | 8.1 (0.4-65.4) | 0.586 |
| **Urine FLC λ /Cr (mg/mmol), median (IQR)** | 1.5 (0-6.8) | 3.0 (0-6.3) | 3.0 (0-0.3) | 0.442 |
| **Urine FLC K/Cr (mg/mmol), median (IQR)** | 8.7 (0.1-30.5) | 8.7 (0.1-21.5) | 8.3 (0.1-19.8) | 0.256 |
| **Total proteins (g/L), median (IQR)** | 77.5 (76.3-87.2) | 82 (78.8-84) | 83.5 (79-86.5) | 0.337 |
| **β2-microglobulin (mg/L), median (IQR)** | 2.35 (2.1-3.0) | 2.1 (1.8-2.8) | 2.1 (1.8-2.5) | 0.092 |
| **DXA Parameters** | | | | |
| **Lumbar Spine BMD (g/cm2), median (IQR)** | 0.822 (0.750-0.908) | 0.850 (0.7.61-0.953) | 0.832 (0.0.752-0.908) | **0.046** |
| **Femoral Neck BMD (g/cm2), median (IQR)** | 0.749 (0.694-0.805) | 0.759 (0.698-0.826) | 0.784 (0.728-0.836) | **0.015** |
| **Total Hip BMD (g/cm2), median (IQR)** | 0.821 (0.804-0.841) | 0.835 (0.799-0.851) | 0.848 (0.260-0.884) | **0.003** |

| | M0 | M6 | M12 | p-value |
|---|---|---|---|---|
| **Tot.vBMD (mgHA/cm3)** | 303.82 (285.59-363.79) | 308.83 (295.00-365.39) | 301.33 (282.78-325.48) | 0.987 |
| **Ct.vBMD (mgHA/cm3)** | 740.12 (738.29-840.76) | 745.57 (740.86-854.81) | 771.05 (720.30-800.41) | 0.610 |
| **Tb.vBMD (mgHA/cm3)** | 162.34 (148.86-204.24) | 170.30 (165.59-183.59) | 177.91 (152.37-196.99) | 0.821 |
| **Ct.Th (mm)** | 0.73 (0.71-0.76) | 0.76 (0.71-077) | 0.73 (0.69-0.75) | 0.399 |
| **Tb.BV/TV (%)** | 12.07 (11.65-12.64) | 12.53 (11.88-13.01) | 12.65 (11.24-13.74) | 0.832 |
| **Tb.Th (mm)** | 0.28 (0.27-0.28) | 0.27 (0.26-0.28) | 0.27 (0.27-0.27) | 0.603 |
| **Tb.Sp (mm)** | 0.82 (0.82-0.84) | 0.81 (0.81-0.83) | 0.83 (0.78-088) | 0.910 |
| **Stiffness (kN/mm)** | 36.88 (36.70-37.46) | 37.19 (36.62-37.25) | 36.62 (35.16-36.94) | 0.215 |
| **Failure Load (kN)** | 1.06 (0.99-1.16) | 1.35 (1.27-1.51) | 1.16 (1.06-1.27) | 0.079 |

**P180**

### Is there a distinct patient profile for each osteoanabolic drug: teriparatide, romosozumab, and abaloparatide?

*Judith Vidal[1], Clara Churtichaga-Domenech[1], Agueda Prior-Español[1], Francina Salabert[1], Cristina Rocamora[1], Annika Nack[1], Susana Holgado[1], Lourdes Mateo[1], Laia Gifre[1]*
*[1]Hospital Universitari Germans Trias i Pujol, Rheumatology, Barcelona, Spain*

**Abstract Text**

**Introduction**: Clinical practice guidelines identify patients who are candidates for initiating osteoanabolic treatment; however, they do not differentiate which specific drug should be started.

**Purpose:** Our objective was to assess whether differences exist among patients who start teriparatide, abaloparatide, or romosozumab in real-world clinical practice.

**Methods**: We conducted a retrospective observational study including 47 patients who initiated osteoanabolic therapy (teriparatide, romosozumab, or abaloparatide) in a tertiary hospital between 2022–2025. Clinical variables (risk factors, fractures, treatments, comorbidities), laboratory parameters (bone turnover markers), radiological findings, and baseline bone mineral density were collected at treatment initiation.

**Results**: The main variables are shown in Table 1. Among patients initiating teriparatide (n=20), 20% were male, with a higher prevalence of osteoporosis risk factors (smoking, early menopause, corticosteroid use) and greater comorbidity (hypertension, diabetes mellitus, dyslipidemia, and cardiovascular disease). Although 90% had prior fractures, the prevalence of osteoporosis was lower. Combination therapy was used in 20%.

Patients initiating abaloparatide (n=12) were 91% female, had lower hip BMD (compared with teriparatide), a high prevalence of fractures (83%), and more recurrent falls. Twenty-five percent had previously received teriparatide.

All patients starting romosozumab (n=15) were women and presented higher bone turnover markers and lower BMD. All had previous fractures, predominantly vertebral. Thirty-three percent had previously been treated with teriparatide. Seventy-three percent had sustained fractures during prior anti-osteoporotic therapy. No patients had cardiovascular or oncologic disease.

**Conclusions**: In clinical practice, beyond BMD and fracture data, patients starting teriparatide include men and individuals with greater comorbidity and cardiovascular risk. One-third of patients starting abaloparatide or romosozumab had previously received teriparatide and remained at very high fracture risk. Abaloparatide use was common in patients with recurrent falls, whereas romosozumab was more frequent in women with very low bone mass and recent fractures.

Table 1.

| | ALL 47 | TERIPARATIDE 20 | ABALOPARATIDE 12 | ROMOSOZUMAB 15 |
|---|---|---|---|---|
| **Age (years)** | 73.4 ± 1.5 | 75.4 ± 10.4 | 72.8 ± 8.4 | 71.9 ± 7.6 |
| **Women (%)** | 89.36 | 80 | 91.67 | 100 |
| **Postmenopausal osteoporosis (%)** | 82.97 | 75 | 83.33 | 93.33 |
| **Active smoking (%)** | 14.89 | 20 | 16.67 | 6.67 |
| **Early menopause (%)** | 12.76 | 20 | 8.33 | 6.67 |
| **Corticotherapy (%)** | 8.5 | 10 | 8.33 | 6.67 |
| **Falls in last year** | 17.02 | 5 | 41.67 | 20 |
| **Previous oral bisphosphonates (%)** | 53.13 | 45 | 50 | 66.7 |
| **Previous IV bisphosphonates (%)** | 21.28 | 10 | 8.33 | 46.67 |
| **Previous osteoformer (%)** | 17 | 0 | 25 | 33.33 |
| **Previous fractures (%)** | 91.48 | 90 | 83.33 | 100 |
| **Number of vertebral fractures** | 2 ± 1.9 | 2.2 ± 1.9 | 1.5 ± 1.7 | 2.2 ± 2 |
| **Number of non-vertebral fractures** | 0.6 ± 0.9 | 0.4 ± 0.6 | 0.6 ± 0.6 | 1.1 ± 1.3 |
| **Hip fracture (%)** | 12.8 | 20 | 8.33 | 6.67 |
| **Hypertension (%)** | 44.1 | 55 | 41.67 | 33.33 |
| **Diabetes Mellitus (%)** | 12.8 | 20 | 16.67 | 0 |
| **Dyslipidemia (%)** | 36.17 | 50 | 41.67 | 13.33 |
| **Cardiovascular disease (%)** | 6.3 | 10 | 8.33 | 0 |
| **Lumbar BMD (g/cm$^2$)** | 0.791 ± 0.127 | 0.846 ± 0.142 | 0.784 ± 0.110 | 0.736 ± 0.091 |
| **Femoral neck BMD (g/cm$^2$)** | 0.711 ± 0.086 | 0.759 ± 0.094 | 0.678 ± 0.060 | 0.676 ± 0.057 |
| **Total femur BMD (g/cm$^2$)** | 0.736 ± 0.117 | 0.788 ± 0.127 | 0.711 ± 0.115 | 0.692 ± 0.072 |

OP = Osteoporosis; VF = Vertebral Fractures; NVF = Non-vertebral Fractures; Hip Fx = Hip fracture.

Results are shown as percentage or mean ± SD

**P181**

### Effect of caffeine on the musculoskeletal system in an experimental model of sporadic Alzheimer's disease in estrogen-deficient rats

*Piotr Londzin[1], Maria Zych[2], Joanna Folwarczna[1]*
*[1]Medical University of Silesia- Katowice, Department of Pharmacology, Sosnowiec, Poland*
*[2]Medical University of Silesia- Katowice, Department of Pharmacognosy and Phytochemistry, Sosnowiec, Poland*

### Abstract Text

Alzheimer's disease (AD) and osteoporosis are diseases of old age. Both diseases occur more frequently in women. There is increasing interest in dietary components that may affect the development of the diseases. Such a compound may be caffeine, which has been reported to exert some beneficial effects in experimental AD models. We have previously observed that administration of caffeine at a moderate dose favorably affected the skeletal system in female rats. The aim of the present study was to investigate the effects of caffeine on the musculoskeletal system in estrogen-deficient rats with streptozotocin (STZ)-induced sporadic AD.

The experiments were carried out on mature female Wistar rats, divided into 4 groups (*n*=10-12): sham-operated control rats, ovariectomized (OVX) control rats, OVX rats with experimental AD, and OVX rats with experimental AD treated with caffeine. Experimental AD was induced by a single *i.c.v.* injection of STZ one week after the bilateral ovariectomy surgery. Administration of caffeine (20 mg/kg *p.o.*, once daily) started four weeks after the STZ injection and lasted four weeks. Serum bone turnover markers, bone mass, density, composition, as well as histomorphometric parameters and mechanical properties of cancellous and compact bone, and skeletal muscle mass and strength were determined.

Estrogen deficiency worsened cancellous bone histomorphometric parameters and mechanical properties in the OVX control rats. There was no significant effect of *i.c.v.* STZ on the musculoskeletal system compared to the OVX control rats. Administration of caffeine to the OVX rats with experimental sporadic AD counteracted the effects of the model on cancellous bone histomorphometric parameters, increasing BV/TV and decreasing Tb.Sp values.

In conclusion, results of the present study support the notion that moderate dose caffeine may favorably affect the skeletal system.

**P182**

**Lack of effect of diosmin on changes in bone mechanical properties induced by estrogen deficiency in rats**

*Maria Zych[1], Weronika Borymska[1], Radosław Wolan[2], Kacper Grzywnowicz[2], Sylwia Klasik-Ciszewska[2], Joanna Folwarczna[2], Ilona Kaczmarczyk-Żebrowska[1]*
*[1]Medical University of Silesia- Katowice, Department of Pharmacognosy and Phytochemistry, Sosnowiec, Poland*
*[2]Medical University of Silesia- Katowice, Department of Pharmacology, Sosnowiec, Poland*

**Abstract Text**

There is increasing interest in substances of plant origin, which may exert health-promoting activities. Diosmin, a representative of flavonoids, has been reported to exert antioxidative and anti-inflammatory effects, and to favorably affect rat skeletal system. Diosmin is widely used in the treatment of venous chronic insufficiency; it seems that possible improvement in blood microcirculation exerted by diosmin may contribute to its antiosteoporotic potential. The aim of the study was to investigate the effects of diosmin, administered at a dose corresponding to that used in clinical practice, on bone mechanical properties in estrogen-deficient (bilaterally ovariectomized) rats.

The experiments were carried out on 3-month-old male Wistar rats, divided into four groups (n=7): I – healthy control rats, II – ovariectomized control rats, III – ovariectomized rats receiving estradiol (0.2 mg/kg), IV – ovariectomized rats receiving diosmin (100 mg/kg). Estradiol or diosmin administration, once daily by oral gavage, started one week after the bilateral ovariectomy surgery and lasted four weeks. The body mass gain and uterine mass, serum calcium and phosphorus concentrations, bone mass and mineralization (tibia, femur, L4 vertebra), and mechanical properties of cancellous (the proximal tibial metaphysis) and compact (the femoral diaphysis), as well as the femoral neck were examined.

Estrogen deficiency caused an increase in the body mass gain and a decrease in uterine mass, disorders in bone mineralization, strong worsening of cancellous bone mechanical properties, and slight unfavorable effect on mechanical parameters of compact bone. Estradiol reduced body mass gain and increased uterine mass in ovariectomized rats. Diosmin administration further increased the body mass gain in relation to the ovariectomized controls. No effect of diosmin on bone mass, mineralization, and mechanical properties was demonstrated in ovariectomized rats.

In conclusion, the results indicate that diosmin should not be treated as a potential drug for the prevention and treatment of osteoporosis.

**P186**

**Denosumab versus Zoledronate in Postmenopausal Women with Primary Hyperparathyroidism and Osteoporosis: A Randomized Pilot Study**

*Katarina Mlekus Kozamernik*[1], *Tomaž Kocjan*[1]
[1]*UMC Ljubljana, Dept. of Endocrinology- Diabetes and Metabolic Diseases, Ljubljana, Slovenia*

**Abstract Text**

**Introduction:** Primary hyperparathyroidism (PHPT) is frequently associated with reduced bone mineral density (BMD). In patients managed conservatively, antiresorptive therapy aims to prevent skeletal deterioration and mitigate hypercalcemia, but data directly comparing available agents are limited.

**Objective:** To compare the effects of denosumab and zoledronate on serum calcium and BMD in postmenopausal women with PHPT and osteoporosis.

**Methods:** In this randomized, open-label pilot study, 40 postmenopausal women with PHPT and osteoporosis (mean age 73.0±7.8 years) were assigned to zoledronate 5 mg intravenously once yearly (ZOL) or denosumab 60 mg subcutaneously every six months (DMAB) (ClinicalTrials.gov NCT04085419). Serum total calcium was assessed at 3, 6, 12, 18, and 24 months. BMD at the lumbar spine (LS), total hip (TH), femoral neck (FN), and one-third radius (1/3R) was measured at baseline, 12 months, and 24 months.

**Results:** Serum calcium decreased significantly at 3 months ($F (1,37) = 15.9$; $p<0.001$) and remained below baseline at 6 months ($F (1,37) = 5.1$; $p=0.03$) in both treatment groups, with no between-group differences observed. From 12 to 24 months, serum calcium no longer differed from baseline. No significant differences in BMD were detected between groups at any skeletal site or time point. Within-group analysis showed that in the DMAB group, BMD increased significantly during the first year at LS (+4.9%) and TH (+5.0%), with no additional gains in the second year. In the ZOL group, a significant first-year increase in BMD was observed only at the LS (+3.5%). BMD at the FN and 1/3R remained unchanged over two years in both groups.

**Conclusion:** In postmenopausal women with PHPT and osteoporosis, two years of denosumab and zoledronate treatment resulted in comparable, transient reductions in serum calcium. BMD gains did not differ between treatments, were site-specific, and largely confined to the first treatment year.

**P187**

**Sequential Hepatotoxicity and Polyarthritis Following Zolendronate Infusion: A Rare Dual Adverse Reaction**

*Maria Evangelia Koloutsou[1], Maria Mateniadou[1], Polyzois Makras[2], Efthymia Giannitsioti[3], Eva Kassi[1], Katerina Laskari[3], Maria Yavropoulou[1]*
*[1]General Hospital of Athens Laiko- Athens- Greece.,*
*Endocrinology Unit- First Department of Propaedeutic and Internal Medicine- National and Kapodistri an University of Athens General Hospital of Athens Laiko- Athens- Greece., Athens, Greece*
*[2]251 Hellenic Air Force and VA General Hospital- Athens- Greece.,*
*Department of Endocrinology and Diabetes and Department of Medical Research- 251 Hellenic Air For ce and VA General Hospital- Athens- Greece., Athens, Greece*
*[3]General Hospital of Athens Laiko- Athens- Greece.,*
*First Department of Propaedeutic and Internal Medicine- National and Kapodistrian University of Athe ns General Hospital of Athens Laiko- Athens- Greece., Athens, Greece*

**Abstract Text**

**Background:** Zoledronate is widely used for osteoporosis management and the prevention of fragility fractures. Acute phase response (APR) is common, immune-mediated, self-limiting and typically presenting within 1–3 days. Hepatotoxicity and arthritis are extremely rare and sparsely documented.

**Case:** A 65-year-old postmenopausal woman with osteoporosis, previously treated with denosumab for 11 consecutive years, received a 5 mg infusion of zoledronate 6 months after the last denosumab injection in the context of denosumab discontinuation management with a lowest T-score in the femoral neck of −2. She experienced an acute-phase response with fever, chills, and myalgias on days 1–2, resolving by day 3. Ten days later she developed fatigue and diffuse abdominal pain. Laboratory evaluation showed marked hepatocellular injury (AST 804 U/L, ALT 618 U/L, γ-GT 642 U/L) with normal bilirubin and significantly elevated inflammatory markers (CRP 47 mg/L, ESR 85 mm/h).Viral hepatitis panels, autoimmune markers, and abdominal ultrasound were unremarkable. Supportive management led to complete biochemical resolution within 10 days. Thirty-two days after the infusion she developed acute symmetrical polyarthritis (shoulders, elbows, and small joints of the hands) with elevated inflammatory markers but normal radiographs and liver enzymes. Autoimmune testing was negative. Prednisolone 10 mg/day for 10 days resulted in rapid improvement and full normalization without recurrence.

**Discussion:** Both hepatotoxicity and arthritis have been exclusively described following zolendronate administration in few cases; however, their sequential occurrence in the same patient represents an extremely rare event. The temporal association, the presence of systemic inflammation, and the absence of alternative diagnoses suggest a late a delayed immune response potentially triggered by the zoledronate-induced acute phase response.

**Conclusion:** We report the first documented case of two rare adverse events—acute hepatocellular injury and delayed immune-mediated polyarthritis—occurring sequentially after zoledronate treatment. Appropriate monitoring and timely recognition are crucial while supportive treatment and short term prednisolone may lead to complete recovery without further long-term management.

**P188**

**T2T in osteoporosis: Experience in clínical practice**

*Daniel Roig[1], Dacia Cerdà Gabaroi[1], Paula Valentina Estrada Alarcón[1], Vanesa Andrea Navarro Ángeles[1], Sergi Heredia Martín[1], Oscar Armando Camacho Alcázar[1], Marta López Gómez[1], Silvia García Díaz[1], Delia Reina Sanz[1]*
*[1]Complex Hospitalari Universitari Moisès Broggi, Rheumatology, Sant Joan Despí, Spain*

**Abstract Text**

**Background:** Osteoporosis (OP) management has evolved to a treat-to-target strategy based on baseline fracture risk.

**Objective:** To analyse characteristics of densitometric osteoporotic patients who improved to osteopenia or normal BMD after treatment.

**Methods:** Patients diagnosed with OP by dual-energy X-ray absorptiometry (DXA) at LS and/or FN were included. Other inclusion criteria were: (1) no prior OP treatment before baseline DXA (except HRT), (2) therapy begun after the first DXA, and (3) at least one follow-up DXA. The final DXA was the latest performed during continuous treatment or before treatment discontinuation or goal achievement. Treatment goal was defined as improvement to osteopenia in both LS and FN. Variables: Demographics, BMI, lifestyle factors, comorbidities, glucocorticoid (GC) use, fractures before and during follow-up, parental fracture history, baseline and final BMD and T-scores, follow-up duration, and treatment regimen. Statistical analysis included the description of the sample and the comparison of variables between the group that achieved the treatment target and the group that did not.

**Results:** 897 patients met inclusion criteria (tables 1 and 2). 403 improved to osteopenia (44.4%) or normal BMD (0.6%)

Men achieved the goal faster (3.58±2.34 vs. 4.91±4.04 years, P=0.004), as did older patients (R=-0.120, P=0.016), those with better baseline BMD in LS (R=-0.321, P=0.000), patients with bone-affecting conditions or treatments (3.71±2.97 vs. 5.04±4.49, P=0.002), including GC (3.86±3.03 vs. 5.06±4.49, P=0.018), and those without fractures during follow-up (P<0.001 for osteoporotic, non-osteoporotic, and any fractures). The number of treatments correlated with treatment duration

(R=0.671, P=0.000), while the treatment initiation year correlated negatively with treatment duration (R=-0.209, P=0.000).

**Conclusions:** Patients achieving the target were younger, with better initial BMD, higher weight and height, and fewer new fractures. Factors influencing treatment duration included gender, age, baseline LS BMD, GC exposure, fractures during follow-up, and number of drugs used. More recent treatment initiation was associated with shorter duration.

**P189**

**Efficacy and saturation of sclerostin antibody therapy in a mouse model of osteoporosis**

*Neashan Mathavan[1], Gisela A Kuhn[1], Esther Rosenwald[1], Friederike A Schulte[1], Ralph Müller[1]*
*[1]ETH Zurich, Institute for Biomechanics, Zurich, Switzerland*

**Abstract Text**

**Background/Introduction:** Sclerostin antibody (SclAB), a potent anabolic therapy, increases bone mass by inhibiting sclerostin-mediated suppression of Wnt signalling. Peak efficacy is observed early in treatment, with the anabolic response eventually reaching saturation, limiting further gains in bone mass. Understanding the mechanisms underlying saturation is critical for extending the anabolic window and optimizing therapeutic efficacy.

**Purpose:** To investigate the dynamics of SclAB efficacy and saturation in a mouse model of osteoporosis using time-lapsed *in vivo* micro-CT.

**Methods:** 16-week-old female C57BL/6 mice (n=30) underwent bilateral ovariectomy (license: ZH011_2025). Five weeks after surgery, mice were subdivided into two treatment groups: Vehicle (VEH, n=11) and SclAB (n=19) (25 mg/kg administered subcutaneously twice/week, UCB Biopharma SRL). *In vivo* micro-CT imaging of the $6^{th}$ caudal vertebra was performed fortnightly (treatment weeks 0-16). Trabecular bone parameters were quantified using the full trabecular mask and included bone volume fraction (BV/TV), trabecular thickness (Tb.Th) and bone formation/resorption rates (BFR/BRR).

**Results:** At the onset of treatment, mice had lost -20.4 ±1.3% (mean ± SEM) of BV/TV five weeks following ovariectomy. SclAB efficacy was evident in the potent bone formation response induced, resulting in a greater than twofold increase in BV/TV by treatment week 16 (+126.6 ±4.8% vs. +13.5 ±1.9% in VEH mice; p<0.0001) (Fig.1A,1E). This effect was attributable to a pronounced increase in Tb.Th (+94.2 ±2.4% vs. +9.1 ±1.3%; p<0.0001) (Fig.1B). In SclAB-treated mice, BFR was significantly higher compared to vehicle-treated mice in only the first 4 weeks (p<0.001, p<0.0001) (Fig.1C), whereas BRR declined steeply and remained suppressed (p<0.01, p<0.0001) (Fig.1D). Consequently, dynamic parameters saturated by week 4, while static parameters continued to increase due to the sustained imbalance between BFR and BRR.

**Conclusion(s):** Collectively, these findings reflect the potent anabolic efficacy of SclAB and highlight the saturation of dynamic parameters at 4 weeks of treatment.

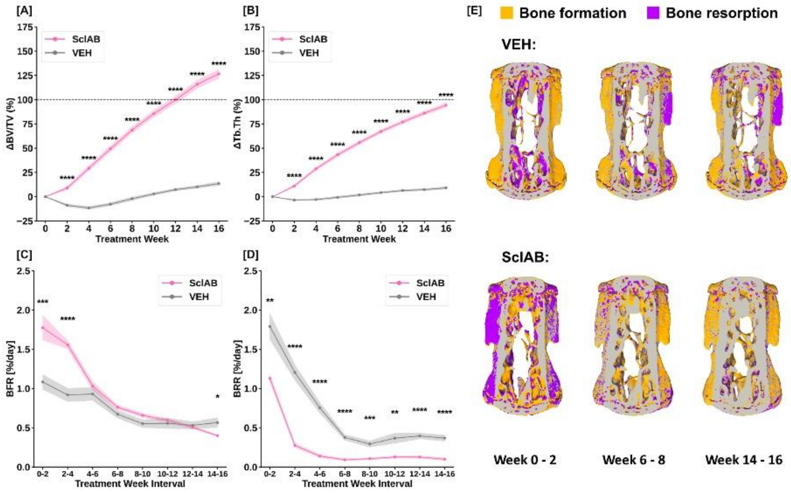

**Fig 1:** Evaluated micro-CT derived bone parameters presented as mean ±SEM: **[A]** ΔBV/TV, **[B]** ΔTb.Th (trabecular thickness), **[C]** BFR (bone formation rate per bone volume) and **[D]** BRR (bone resorption rate per bone volume). **[E]** Volume renderings of representative samples from each treatment group. Statistics: Comparisons between treatment groups at each timepoint were performed using two-way repeated-measures ANOVA followed by Tukey's post-hoc test (* $p < 0.05$, ** $p < 0.01$, *** $p < 0.001$, **** $p < 0.0001$).

### P190

### Multiple fragility fractures after completion of Romosozumab therapy despite preserved Bone Mineral Density: A challenging case of therapeutic failure.

*Maria Evangelia Koloutsou[1], Polyzois Makras[2], Athanasios Anastasilakis[3],*
*Maria Mateniadou[1], Maria Yavropoulou[1]*
*[1]General Hospital of Athens Laiko- Athens- Greece.,*
*Endocrinology Unit- First Department of Propaedeutic and Internal Medicine- National and Kapodistrian University of Athens General Hospital of Athens Laiko- Athens- Greece., Athens, Greece*
*[2]251 Hellenic Air Force and VA General Hospital- Athens- Greece.,*
*Department of Endocrinology and Diabetes and Department of Medical Research- 251 Hellenic Air Force and VA General Hospital- Athens- Greece., Athens, Greece*
*[3]424 General Military Hospital- Thessaloniki- Greece.,*
*Department of Endocrinology- 424 General Military Hospital- Thessaloniki- Greece., Thessaloniki, Greece*

### Abstract Text

**Background**: Romosozumab is an anabolic agent that stimulates bone formation and reduces resorption, improving bone mineral density (BMD) and lowering fracture risk in severe osteoporosis. With its increasing use, cases of variable response and fractures despite therapy are being reported, highlighting the need for careful monitoring.

**Purpose:** This case report aims to illustrate a possible unfavorable response to romosozumab in a patient with severe osteoporosis, to explore reasons for treatment failure—including secondary causes of osteoporosis—and to discuss subsequent therapy.

**Methods:** BMD was assessed using DXA at the spine and hip. X-rays of the right forearm and thoracolumbar spine were obtained to identify vertebral fractures. Bone turnover markers were monitored, and investigations for secondary causes of osteoporosis were conducted before and after romosozumab therapy.

**Results:** A 66-year-old postmenopausal woman, previously treated with ibandronate, was evaluated after an L3 vertebral fracture. DXA scan showed severe osteoporosis (T-scores: spine −3.7; left femoral neck −3.0; right femoral neck −2.6). Evaluation for secondary causes was negative. She received romosozumab 210 mcg monthly with calcium and vitamin D. After one year, despite adherence and monitored bone markers, she sustained a distal radius fracture and multiple thoracic and lumbar vertebral fractures. Follow-up BMD showed T-scores of −3.4 at the spine and −2.2 at the left femoral neck. Subsequent therapy with denosumab was initiated.

**Conclusion:** As romosozumab use expands, clinicians may encounter new fractures or unfavorable BMD responses in high-risk patients. Careful monitoring of BMD, fractures, and bone turnover markers is essential. Non-adherence, secondary causes, and prior antiresorptive therapy should be considered as potential contributors. Transitioning to antiresorptive therapy, such as denosumab, may be warranted in cases of inadequate response or persistent fracture risk.

**P192**

**Short- and mid-term persistence to osteoporosis medications among patients with type 2 diabetes mellitus: a population-based cohort study**

*Cristina CARBONELL ABELLA[1,2], Laura Gómez Rodriguez[1,2], Carlen Reyes[2], Maria Antonia Pou Gimenez[1,2], César Díaz Torné[2,3], Xavier Nogues Solan[4], Daniel Prieto Alhambra[5], Daniel Martinez Laguna[1,2]*

*[1]ICS, Family Medicine, Barcelona, Spain*
*[2]GREMPAL research group of IDIAP Jordi Gol, Universitat Autònoma, Barcelona, Spain*
*[3]Hospital de Sant Pau, Rheumatology, Barcelona, Spain*
*[4]Hospitaldel Mar, URFOA Internal Medicine, Barcelona, Spain*
*[5]Centre for Statistics in Medicine- NDORMS-, University of Oxford, Oxford, United Kingdom*

**Abstract Text**

**Introduction:** Individuals with type 2 Diabetes Mellitus (T2DM) present a higher risk of fragility fractures. Nevertheless, this risk is frequently underestimated, leading to a reduced use of osteoporosis drugs (OPD)

**Objective:** To evaluate potential differences in persistence to OPD between patients with T2DM and non-diabetic individuals.

**Methods:** A population-based cohort study was performed in the primary care context. All patients who began OPD therapy between 2012-2016 were identified using the SIDIAP database, which includes anonymized clinical data from >5.5 million individuals in Catalonia. Patients who had used OPD in the previous 12 months were excluded. Follow-up continued until death, discontinuation or change to another OPD, or study completion (31/12/2018). Persistence was defined as the ratio between the number of defined daily doses (NDDD) and the duration of treatment, calculated from the initial to the final billing with a permissible gap not exceeding 3 months, as appropriate. Non-parametric methods were applied to identify differences in drug persistence between T2DM and non-diabetic patients and according to OPD category.

**Results:** A total of 45,020 individuals initiating OPD were identified (6,072 with T2DM and 38,948 non-diabetics). Oral bisphosphonates were prescribed to 80.6%, denosumab (DMAB) to 9.7%, raloxifene/bazedoxifene (SERM) to 6.3% and teriparatide (TPD) to 3.4%. Using a 3-month gap for all OPD, persistence at 1-year was 47.6%, at 2-years 31.2% and at 3-years 21.9%. (no statistically significant differences between T2DM and non-diabetics). When analysing specific therapies, denosumab showed the highest persistence. Among patients with T2DM, persistence was significantly lower (p<0.05) compared with non-diabetics for DMAB at all time points, and for SERM and TPD at 2 years (Table 1).

**Conclusions:** In general, persistence to OPD among patients with T2DM is low and comparable to that observed in non-diabetic individuals. However, for those receiving DMAB, SERM and TDP, persistence is reduced in the T2DM2group.

**ND-P022**

**A real-world study on the efficacy and adverse events of romosozumab in Chinese patients with osteoporosis**

*Wu Haoyu[1], Ouyang Zhuji[1], Li Chunhai[1], Yue Ding[1]*
*[1]Sun Yat-sen Memorial Hospital- Sun Yat-sen University, Department of Orthopaedics, Guangzhou, China*

**Abstract Text**

**Background:** Currently, romosozumab is not yet commercially available in mainland China, and data on its real-world clinical application remain relatively scarce. Therefore, there is an urgent need to accumulate and analyze clinical data on romosozumab among Chinese osteoporosis patients.

**Purpose:** To analyze the therapeutic and adverse effects of romosozumab among female osteoporosis patients at very high fracture risk in China.

**Methods:** A retrospective study initiated in October 2024 until December 2025, involving postmenopausal women with severe osteoporosis who are treated with romosozumab. BMD at the lumbar spine (L1-L4), femoral neck, and total hip were measured by DXA at baseline, 6 months and 12 months. Serum bone turnover markers and calcium-phosphate metabolism parameters were also monitored.

**Results:** Among the 55 female patients, 56.3% were treatment-naive. The mean age ± standard deviation for treatment-naive patients and previously treated patients was 70.2 ± 9.3 years and 73.0 ± 8.6 years, respectively.The previous osteoporosis treatments were denosumab (36.8%), zoledronic acid (31.6%), and teriparatide (15.8%), with 15.8% of patients having received two or more types of medication. At 6 and 12 months of romosozumab treatment, the mean percentage increases in BMD from baseline were 7.4% and 16.6% at the lumbar spine, 2.8% and 5.9% at the total hip, and 3.0% and 7.5% at the femoral neck, respectively. All changes were statistically significant compared with baseline.When analyzed by prior treatment history, treatment-naive patients showed a 11.2% increase in lumbar spine BMD at 6 months, whereas previously treated patients showed a 2.8% increase. For the femoral neck, the increases were 3.1% and 3.3%, and for the total hip, 2.3% and 2.8%, respectively.No significant complications related to cardiac or other organ diseases were observed during treatment.

**Conclusion:** Significant improvements in lumbar spine, total hip, and femoral neck BMD were observed following 6 and 12 months of romosozumab treatment, according to real-world evidence.

**ND-P023**

**The Relation between Changes in Bone turnover Markers and BMD differs significantly between Estrogen and Alendronate: Evidence of positive remodeling Balance during Menopausal Hormone Therapy**

*Erik Fink Eriksen[1], Sindre Lee-Ødegård[2], Barbara Fink Eriksen[3], Tove Tveitan Borgen[4]*
*[1]University of Oslo, Faculty of Odontology, Oslo, Norway*
*[2]Oslo University Hospital- Oslo- Norway,*
*Department of Endocrinology- Morbid Obesity and Preventive Medicine, Oslo, Norway*
*[3]Spesialistsenteret Pilestredet Park, Osteoporose, Oslo, Norway*
*[4]Vestre Viken Hospital Trust- Drammen Hospital, Department of Rheumatology, Drammen, Norway*

**Abstract Text**

The main bisphosphonate effect on bone is mediated through anti-activation, i.e. lowering of bone turnover, reduction of the remodeling space, increased matrix mineralization and bone mineral density (BMD). This is reflected in a negative correlation between BMD gains and bone turnover marker levels. Histomorphometrically, no positive remodeling balance has been demonstrated in bisphosphonates, and BMD gains levels off after 4-5 years of therapy. However, studies on menopausal hormone therapy (MHT) report improvement in remodeling balance after 2 years, increased wall thickness and continuous BMD increases following 6-9 years of treatment. If BMD gains of MHT are caused by positive remodeling balance, you would expect BMD gains to occur at higher bone turnover rates, offsetting potential decreases in BMD due to increased remodeling space. To test this hypothesis, we investigated the relation between bone turnover and changes in BMD in patients treated up to 8 years with MHT or alendronate (ALN).

We assessed BMD at lumbar spine and hip, S-P1NP and S-CTX levels at baseline and up to 8 years of treatment in 75 patients on MHT and 100 patients on ALN (mean age 55 and 72 years, respectively).

In individuals on MHT, BMD increases occurred at higher bone turnover rates than seen in individuals on ALN ($p<0.001$)(Figure). Mixed modeling analysis of changes in P1NP vs changes in TH-BMD over 8 years revealed differences in slope between ALN and MHT ($p<0.001$). While ALN group displayed negative correlation between PINP and increases in BMD, MHT displayed a negative trend early on, shifting to a slightly positive slope later, suggesting the development of a positive remodeling balance over time. Similar differences were demonstrated for CTX vs. BMD.

These data suggest that MHT apart from anti-activation effects early on creates a long-term positive remodeling balance, which is not demonstrable in patients treated with ALN.

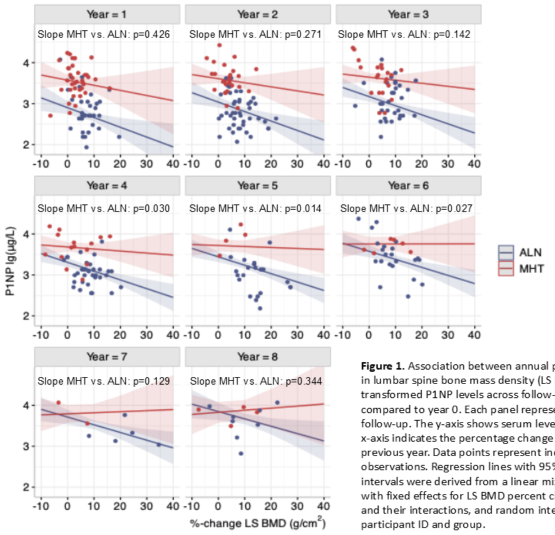

**Figure 1.** Association between annual percentage change in lumbar spine bone mass density (LS BMD) and log-transformed P1NP levels across follow-up years 1 to 8 compared to year 0. Each panel represents one year of follow-up. The y-axis shows serum levels of P1NP and the x-axis indicates the percentage change in LS BMD from the previous year. Data points represent individual observations. Regression lines with 95% confidence intervals were derived from a linear mixed-effects model with fixed effects for LS BMD percent change, year, group, and their interactions, and random intercepts for participant ID and group.

**ND-P026**

**Osteoclasts and Denosumab Discontinuation: ZOLARMAB2 - A Danish RCT with Osteoporotic Women - Preliminary investigations of Baseline osteoclast activity.**

*<u>Louise Hjorth Lind</u>[1], Natasha Laursen[2], Simone R.S. Nielsen[1], Jacob B. Olesen[3], Anne Sophie Sølling[2], Torben Harsløf[2], Bente Langdahl[2,4], Kent Søe[1,3]*
*[1]University Of Southern Denmark, Department of Clinical Research, Odense M, Denmark*
*[2]Aarhus University Hospital, Department of Endocrinology and Internal Medicine, Aarhus, Denmark*
*[3]Odense University Hospital, Department of Pathology, Odense, Denmark*
*[4]Aarhus University, Department of Clinical Medicine, Aarhus, Denmark*

**Abstract Text**

**Introduction:** ZOLARMAB2 is a nation-wide Danish RCT (n=200) investigating an optimized algorithm for preventing bone loss after discontinuing denosumab treatment with zoledronate in postmenopausal women with osteoporosis. Osteoclast activity was assessed *in vitro* in a subset of participants. Here, we present preliminary baseline data from this exploratory sub-cohort.

**Purpose:** Characterise the bone resorption activity of osteoclasts *in vitro* after denosumab discontinuation.

**Methods:** Blood samples were collected at baseline, six months after the last denosumab dose.

Osteoclasts were generated and their resorptive activity were tested on bone slices. Resorption activity was quantified as trench surface/bone surface (TS/BS), pit surface/bone surface (PS/BS), and the activity levels of tartrate-resistant acid phosphatase (TRAcP) were evaluated. Data was analysed using multiple linear regression analysis (MLR).

**Results:** Sixty postmenopausal women (mean age 66.8 ± SD 6.57 years) previously treated with denosumab for 11 ± 4.95 years were included. BMD T-scores at the lumbar spine, total hip, and femoral neck were -1.41 ± 0.83, -1.12 ± 0.69, and -1.51 ± 0.67, receptively. Osteoclastic bone resorptive activity *in vitro* was TS/BS; 1.39% ± 1.50, PS/BS; 0.28% ± 0.45 (n=30 - to date), and TRAcP activity; 0.362 ± 0.44 (n = 60).

Treatment duration with denosumab did not correlate with the resorptive activity of osteoclasts ($R^2$ = 0.58, TS/BS: p = 0.45; PS/BS: p = 0.34; TRAcP: p = 0.28). Interestingly, TRAcP activity during osteoclastic bone resorption($R^2$ = 0.52) was found to negatively correlate with total hip BMD (p = 0.033) and show a trend towards a negative correlation with PS/BS (p = 0.096).

**Conclusion:** Treatment duration with denosumab did not correlate with osteoclast activity *in vitro*. However, the TRAcP-activity of bone resorbing osteoclasts was found to correlate with baseline BMD. Hence, better BMD correlate with lower osteoclast TRAcP-activity. Analyses are ongoing, including epigenetic, various biomarker, and prospective analyses.

**Poster Sessions: Other diseases of bone and mineral metabolism**

**P193**

**The Two Different Faces of Hyperparathyroidism**

*Chee Kian Chew*[1]
[1]*Tan Tock Seng Hospital, Endocrinology, Singapore, Singapore*

**Abstract Text**

**Introduction:** Primary hyperparathyroidism (PHPT) is curable surgically. However, low calcium intake post-operatively is commonly overlooked leading to the development of secondary hyperparathyroidism.

**Case Report:** A 78 year-old female was referred for hypercalcemia found during medical check-up. She was asymptomatic of hypercalcemia. There were no symptoms to suggest any malignancy and no history of prolonged immobilization. She denied family history of hypercalcemia or taking any drug that can cause hypercalcemia. On examination, there was no pallor, lymphadenopathy and bony tenderness. Breast examination was normal. Abdominal, respiratory, cardiovascular and neurological examinations were unremarkable.

**Biochemical tests:** serum calcium 2.63 mmol/L (2.15-2.58), PTH 15.3 nmol/L (0.8-6.8), phosphate 0.9 mmol/L (0.8-1.6), 25-hydroxyvitamin D 33.0 ug/L (>20), creatinine 66 umol/L (60-105), ALP 110 U/L (38-126). Her calcium/creatinine clearance ratio of 0.03 (>0.02) excluded the diagnosis of familial hypocalciuric hypercalcemia. She was diagnosed with PHPT and planned for surgery. Preoperative sestamibi scan localized a hyperfunctioning right inferior parathyroid adenoma. Her intra-operative PTH dropped from 30.2 to 5.5 nmol/L confirmed the excision of the hyperfunctioning parathyroid adenoma. Post-operatively, her serum calcium and PTH levels normalized. However, at 3 month-follow up, her biochemical tests showed serum calcium 2.25 mmol/L, PTH 18.3 nmol/L, phosphate 0.8 mmol/L and 25-hydroxyvitamin D 37.0 ug/L, prompting a further investigation to rule out persistent PHPT and other causes of hyperparathyroidism. Her low ionized calcium of 1.06 mmol/L (1.16-1.32) and low 24 hours urinary calcium of 1.9 mmol/day (2.5-10) confirmed the diagnosis of secondary hyperparathyroidism from low calcium intake. Further history revealed that she had low calcium diet after surgery due to fear of hypercalcemia. She was started calcium supplement and encouraged to increase her dietary calcium intake. Her adjusted calcium, ionized calcium and PTH levels remained normal since then.

**Conclusion:** Clinician should ensure adequate calcium intake after surgical cure of PHPT to avoid secondary hyperparathyroidism.

**P194**

### Cyclic mechanical stretch enhances osteogenic differentiation of human fibro-adipogenic progenitors implicated in neurogenic heterotopic ossifications

*Stéphane Héraud[1], Juliette Fernandez[2,3], François Etienne[4], François Rannou[4], Sébastien Banzet[3,5], Dorothée Girard[1,3]*
*[1]Institut de Recherche Biomédicale des Armées, Unité Traumatologie de Guerre, Brétigny-sur-Orge, France*
*[2]Institut de Recherche Biomédicale des Armées, Unité Thérapie Cellulaire et Tissulaire, Clamart, France*
*[3]INSERM, UMR MD 1197 Storm, Clamart, France*
*[4]Université Paris Cité,*
*Mechanobiology Platform - BioMedTech Facilities - INSERM US36 - CNRS UAR2009, Paris, France*
*[5]Centre de Transfusion Sanguine des Armées, Recherche et Développement, Clamart, France*

### Abstract Text

Neurogenic heterotopic ossifications (NHO) are pathological extra skeletal bones that develop in periarticular muscles after severe injuries of the central nervous system (CNS). The combination of a severe CNS lesion and macrophages-mediated inflammation deeply alters muscle microenvironnement and stimulates the chondro-osteogenic differentiation of fibro-adipogenic progenitors (FAPs). Profound changes in muscle mechanical properties are also observed and could be involved in NHO pathophysiology. In this pilot study, we aimed to investigate the effect of cyclic mechanical stretch on the osteogenic differentiation of human FAPs.

Human FAPs were isolated from muscle surrounding NHO. Using the Tissue Train® Flexcell® Tension System, $2.10^6$ FAPs were seeded in Type 1 collagen TeloCol® matrices and cultured in control medium or osteogenic differentiation medium (OB) supplemented or not with LPS. From day 3 to day 7 and day 10 to day 14, matrices were submitted to 5% uniaxial cyclic mechanical stretch with a sinusoidal waveform at 1 Hz for 6 hours or in static condition as control. Samples were collected for histological analysis and RT-qPCR analysis of chondrogenic and osteogenic genes expression.

Our data show that under static conditions, Alizarin Red staining of matrices seeded with FAPs displayed mineralization in only 1 out of 6 donors in the OB medium, whereas 5 out of 6 donors in the OB + LPS condition exhibited mineralization, indicative of osteogenic differentiation. Under mechanical stretch, calcium deposition was induced in 1 of the 6 donors cultured in OB medium alone and, interestingly, was triggered or exacerbated in all donors cultured in OB + LPS. Furthermore, mechanical stretch significantly upregulated *BMP2* and *SOX9* expression in FAPs under both OB and OB + LPS medium, while *RUNX2* expression remained unchanged.

This study provides first evidences that the concomitant presence of inflammation and mechanical stretch accentuates the osteogenic differentiation potential of FAPs involved in NHO.

**P195**

**Histomorphometry predicts denosumab-induced HRpQCT parameters improvements in dialysis patients**

*<u>Benjamin Batteux</u>[1], Ana Pimentel[2], Agnes Ostertag[3], Tilman B Drüeke[4], Martine Cohen-Solal[3]*
*[1]Amiens-Picardie University Medical Center, Pharmacology, Amiens, France*
*[2]AURA Paris, Nephrology, Saint-Ouen, France*
*[3]Université Paris-Cité, Inserm U1132, Paris, France*
*[4]Université Paris Sud, Inserm U1018, Paris, France*

**Abstract Text**

**Introduction:** Denosumab has been shown to increase bone mineral density (BMD) in patients on dialysis, but its effects on trabecular and cortical bone microarchitecture using high-resolution peripheral quantitative computed tomography (HRpQCT) assessment have not been reported.

**Methods:** In this longitudinal study, 32 consecutive patients receiving hemodialysis at a single center between 2014 and 2024 had at least one HRpQCT examination at the distal tibia and radius at baseline. Subsequently, 19 patients had repeated HRpQCTs. After excluding the presence of osteomalacia and ensuring well-controlled parathyroid hormone levels, 9 patients with recent fractures were treated with denosumab. Changes in HRpQCT parameters observed over time were compared between denosumab-treated and non-treated (control) patients, using linear mixed models for repeated measures, and baseline histomorphometric parameters were analyzed as predictors of changes in HRpQCT parameters over time.

**Results:** During a median [minimum – maximum] follow-up period of 1.8 [0.7 – 4.9] years, 19 patients on hemodialysis had repeated HRpQCTs over time, including the nine patients treated with denosumab. Compared to control patients, denosumab-treated patients had a significant preservation of (i) the ratio of bone volume to total volume at distal tibia (p=0.006) and radius (p=0.002), (ii) trabecular volumetric BMD at distal tibia (p=0.006) and radius (p=0.002), (iii) trabecular number at distal radius (p<0.001), and (iv) trabecular separation at distal tibia (p=0.025) and radius (p<0.001). Changes in cortical parameters did not significantly differ between groups. At most, changes in trabecular volumetric BMD at the radius tended to be negatively associated with osteoid volume (p=0.057).

**Conclusion:** The present real-life study shows that denosumab improves trabecular bone parameters over time in patients on dialysis therapy, in contrast to the progressive trabecular bone loss observed in the absence of denosumab treatment. Furthermore, our results suggest that the optimal effect of denosumab could be achieved in the absence of mineralization defect.

**P196**

**Feasibility and Preliminary Efficacy of a Musculoskeletal Health Package for Women Undergoing Pelvic Radiotherapy: Results from the RadBone Study.**

*<u>Victoria Chatzimavridou Grigoriadou</u>[1], Lisa Barraclough[2], Kate Haslett[2], Nooreen Alam[2], Robert Bristow[3], Rohit Kochhar[4], Thomas Westwood[4], Louise James[5], Zoe Merchant[6], John Moore[7], Karen Johnson[2], Sarah O'Connell[4], Sally Taylor[8], Melissa Perry[9], Glen Martin[10], Joy Thorpe[11], Janelle Yorke[12], Claire Higham[13]*

*[1]Christie NHS Foundation Trust- University of Manchester, Department of Endocrinology- School of Medical Sciences, Manchester, United Kingdom*

*[2]Christie NHS Foundation Trust, Department of Clinical Oncology, Manchester, United Kingdom*

*[3]University of Manchester, Division of Cancer Sciences- Faculty of Biology- Medicine and Health- School of Medical Sciences, Manchester, United Kingdom*

*[4]Christie NHS Foundation Trust, Department of Radiology, Manchester, United Kingdom*

*[5]Christie NHS Foundation Trust, National Institute for Health and Care Research NIHR Manchester Biomedical Research Centre BRC Liv ing with and Beyond Cancer Theme., Manchester, United Kingdom*

*[6]Manchester University Hospitals NHS Foundation Trust, North West Lung Centre, Manchester, United Kingdom*

*[7]Manchester University Hospitals NHS Foundation Trust, Department of Anaesthesia, Manchester, United Kingdom*

*[8]Christie NHS Foundation Trust- University of Manchester, Christie Patient Centred Research- Division of Nursing Midwifery and Social Work- School of Health Sci ences- Faculty of Biology- Medicine & Health, Manchester, United Kingdom*

*[9]Christie NHS Foundation Trust, Christie Patient Centred Research, Manchester, United Kingdom*

*[10]University of Manchester, Division of Informatics- Imaging and Data Science- Faculty of Biology- Medicine and Health, Manchester, United Kingdom*

*[11]Vertec Scientific Limited, Senior Applications Specialist, Manchester, United Kingdom*

[12]Christie NHS Foundation Trust- University of Manchester, Christie Patient-
Centred Research- Division of Nursing- Midwifery & Social Work- School of Health Sciences, Manchester, United Kingdom
[13]Christie NHS Foundation Trust- University of Manchester,
Department of Endocrinology- Division of Cancer Sciences, Manchester, United Kingdom

**Abstract Text**

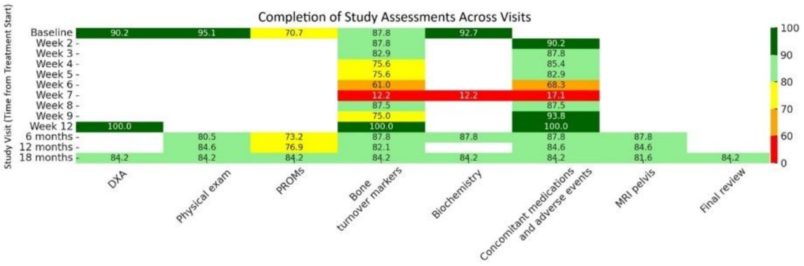

**Background**: Pelvic radiotherapy increases the risk of radiotherapy-related insufficiency fractures (RRIFs), increasing morbidity, healthcare burden, and reducing quality of life. There are currently no evidence-based interventions for RRIFs. Preventive strategies are needed.

**Methods**: The RadBone RCT evaluated the feasibility of a musculoskeletal health package [MHP: musculoskeletal assessment, 3-month prehabilitation exercise (prehab4Cancer), fracture risk evaluation, bone treatments (calcium/vitamin D, bisphosphonates) as indicated] for women receiving pelvic radiotherapy (gynaecological/anorectal cancers) and explored preliminary efficacy. Patients were randomised 1:1 to MHP or observational arm (Obs). Duration of participation was 18 months. DXA scan at baseline/18 months. MHP patients were allocated to High Risk (HR-osteoporosis and/or FRAX above threshold), Medium Risk (MR-osteopenia+FRAX below threshold), or Low Risk (LR-normal BMD) groups. RRIF assessment (MRI scan) at 6,12,18 months. Primary outcome feasibility; secondary outcomes change in BMD, bone turnover markers (CTx,P1NP). Target recruitment was 40 patients/arm (Ethics20/NW/0410).

**Results**: Forty-one patients were enrolled (constrained by COVID-19 pandemic). Cancers included endometrial (n=21), cervical(n=14), anorectal(n=6); 29 were postmenopausal. Baseline DXA-BMD normal (n=16), low BMD/osteopenia (n=18), osteoporosis (n=3). Risk stratification identified 15LR, 15MR, and 7HR; three HR-MHP arm participants received bisphosphonates. Feasibility outcomes: eligibility/screening 13.3% (102/767), recruitment/allocation 40.2% (41/102), attrition 14.6% (6/41). Four patients (1 MHP) with RIFFs identified by 18 months, all from MR/HR groups. No significant difference in fracture risk between arms (p=0.25). Baseline BMD/T-scores lower in MHP arm. MHP intervention appeared to attenuate BMD loss, in lumbar spine (p=0.04) and total hip (p=0.05). Exploratory analysis: P1NP was reduced in MHP compared to control from 6 months onwards (p=0.01); no change in CTx.

**Conclusion**: A multicentre RCT of MHP in patients undergoing pelvic radiotherapy is feasible. Preliminary data suggest RRIF risk highest in those with higher osteoporotic fracture risk and a potential impact from MHP intervention on BMD and bone turnover. Results will support the development of a UK multicentre RCT.

**P197**

### Impact on phospho-calcic and bone metabolism of iron infusions in Hemorrhagic Hereditary Telangiectasia patients. A retrospective study.

*Rose-Marie Javier*[1], *Murielle Rondeau-Lutz*[2], *philippe Mertz*[3], *Olivier Vollmer*[4], *Marc Laplace*[5]
[1]*University Hospital Strasbourg, Rheumatology Unit 6501, Strasbourg, France*
[2]*University Hospital Strasbourg, Internal medicine, Strasbourg, France*
[3]*Hôpital Tenon AP-HP, Department of internal Medicine-, - Paris, France*
[4]*University Hospital, Internal medicine, Strasbourg, France*
[5]*University Hospîtal, Internal medicine - Rheumatology, Strasbourg, France*

**Abstract Text**

**Introduction:** Hereditary hemorrhagic telangiectasia (HHT) is a rare autosomal dominant vascular disorder frequently leading to chronic iron deficiency, often managed with intravenous iron therapy (IIT). Usually well tolerated, IIT, especially ferric carboxymaltose (FCM), can cause hypophosphatemia (HP), potentially leading to osteomalacia. This study explores phosphate metabolism and skeletal complications linked to IIT in HHT patients.

**Method:** This French multicenter retrospective study included HHT patients aged ≥18 years who received at least one IIT (FCM and/or ferric hydroxide-sucrose, FHS) between 01/01/2018 and 31/08/2022.

**Results:** Among 148 patients (M/F ratio 1:2, mean age 63), 70 had phosphate levels measured after infusion; 24 (35%) developed HP. All cases of moderate HP occurred with FCM. Only 1 HP was observed among 14 patients exclusively treated with FHS. Vitamin D levels were not correlated with phosphatemia. Fourteen patients (10 F, 4 M) experienced 18 skeletal events (1 pain, 3 fissures, 14 fractures), all received FCM. HP was significantly associated with bone events (p=0.02; OR 4.1 [CI: 1.16–14.4]). No association was found with smoking, alcohol, corticosteroids, PPIs, or hormonal status. Among patients with HP, bone events were more likely in women (OR 5.71 [1.21–28.9]), those with skeletal fragility risk factors (OR 5.3 [1.3–21.0]), and those treated with FCM (OR 4.1 [1.17–14.42]).

**Conclusion:** In HHT patients receiving IIT, HP was frequent and associated with skeletal events. Among those with HP, increased risk of skeletal events was observed in women, individuals with factors for skeletal fragility, and FCM recipients. Systematic phosphate monitoring is recommended during IIT.

**P198**

### Diagnostic value of the calcium load test for the diagnosis of normocalcemic hyperparathyroidism

*Marta Zampogna*[1], *Marco Demarchi*[2], *Fréderic Triponez*[2], *François Herrmann*[3], *Andrea Trombetti*[4]
[1]*Endocrine Unit- Fondazione IRCCS Ca' Granda Ospedale Maggiore Policlinico,*

*Department of Clinical Sciences and Community Health – Dipartimento di Eccellenza 2023–2027- University of Milan, Milan, Italy*
*[2]Geneva University Hospitals, Division of Thoracic and Endocrine surgery- Department of Surgery, Geneva, Switzerland*
*[3]Geneva University Hospitals, Department of Rehabilitation and Geriatrics, Geneva, Switzerland*
*[4]Geneva University Hospitals, Division of Bone Diseases- Department of Medicine, Geneva, Switzerland*

**Abstract Text**

**Background:** Normocalcemic primary hyperparathyroidism (nPHPT) is defined by elevated parathyroid hormone (PTH) levels with persistently normal calcium levels. The calcium load test (CLTest) has been proposed to differentiate nPHPT from secondary hyperparathyroidism by assessing PTH suppression after calcium administration. This study aimed to evaluate the diagnostic value of CLTest in patients with nPHPT treated at the University Hospitals of Geneva (HUG).

**Methods:** We retrospectively reviewed patients operated for nPHPT (n=91) and a matched control group of hypercalcemic PHPT patients (n=91) treated between 2012-2025. Nineteen nPHPT patients underwent a CLTest before surgery (group A), while 72 were referred for surgery by general practitioners without CLtest (group B). The CLTest consisted of 1 g of oral calcium followed by intravenous calcium gluconate at 2 mg/kg and was considered positive if at least one published PTH suppression criterion was met. All patients underwent preoperative localization studies (neck ultrasound, 18F-fluorocholine PET and/or 99mTc-sestamibi scintigraphy).

**Results:** Bilateral cervical exploration was performed, based on preoperative imaging, in 68% of group A, compared with 44% of group B and 22% of controls ($p<0.001$). Multiglandular disease was more frequent in group A (47%) than in group B (29%) and controls (13%) ($p=0.003$). Histopathological confirmation of parathyroid disease was obtained in all patients in group A and in all controls, whereas 5% (4/72) of group B had no pathological confirmation ($p=0.044$). At 6-month follow-up, persistent PTH elevation occurred in 5% (1/19) of group A, 21% (15/72) of group B, and 15% (14/91) of controls, though persistent hypercalcemia was rare (2/91) ($p=0.296$).

**Conclusions:** The CLTest appears useful in supporting the diagnosis of nPHPT when combined with localization studies. All patients with a positive CLTest had histologically confirmed disease. Given the high prevalence of multiglandular involvement, bilateral exploration should be considered in nPHPT. Multidisciplinary evaluation remains essential for optimal patient care.

**P199**

**Effectiveness of burosumab in fracture healing in 2 patients with XLH**

*Efthymia Karlafti[1], Dimitris Evangelopoulos[1], Anna Papadopoulou[2], Konstantinos Makris[3], Julia Trifonidou[3], Efstathios Chronopoulos[1], Symeon Tournis[1]*
*[1]Medical School- National & Kapodistrian University of Athens,*
*Laboratory for Research of the Musculoskeletal System "Th. Garofalidis"- General Hospital of Athens K AT, Athens, Greece*

*[2]Medical School- National & Kapodistrian University of Athens,*
*Department of Clinical Biochemistry- General Hospital 'Attikon', Athens, Greece*
*[3]KAT General Hospital, Clinical Biochemistry Department, Athens, Greece*

**Abstract Text**

**Background/Introduction:** X-Linked Hypophosphatemia (XLH) is an X-linked dominant form of rickets, characterized by hypophosphatemia and increased fracture risk. Conventional therapy, with phosphate and vitamin D metabolites, does not always ensure adequate fracture healing. Burosumab is a new regimen, a monoclonal antibody against FGF23, with promising effects on the disease's biochemical and clinical manifestations, including fracture healing.

**Purpose:** We present the course of fracture healing in two adults with XLH on burosumab.

**Methods:** We describe two cases of XLH, under conventional therapy, presenting with delayed fracture healing and persistent bone pain. The first patient had difficulties with gait and balance, while the second patient required crutch-assisted ambulation due to bone material impaction in the acetabulum after osteosynthesis in the right femur. Also, the second patient suffered from a non-healing fracture in both left femur and the humerus, with pseudarthrosis formation. Burosumab (70 mg and 80 mg, subcutaneous injection) was prescribed in each patient, respectively. The fractures' healing progress was observed six months after burosumab initiation.

**Results:** Six months after burosumab initiation, phosphorus levels increased and marked healing of the fractures was observed. Radiographs showed the formation of a bridging callus across the interline of the fracture with near resolution of the fracture line, which was accompanied with clinical improvement in pain and improved walking tolerance, assessed by 6-minute walk test, especially in case 1.

**Conclusion(s):** Burosumab is a promising treatment in adults with X-linked hypophosphatemia, especially for patients who do not respond to conventional treatment.

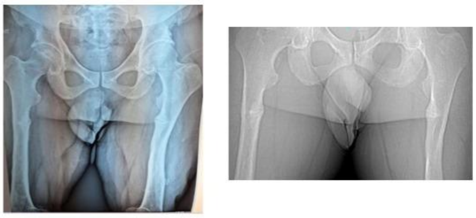

Case 1: baseline (left), after 6 months (right)

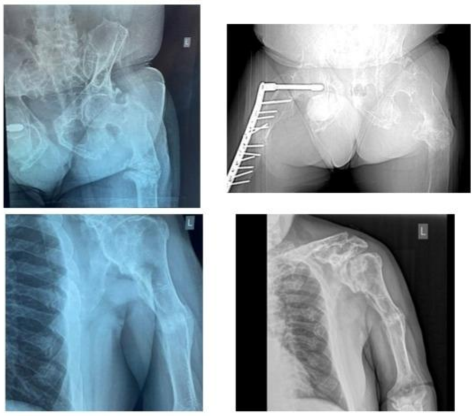

Case 2: baseline (left), after 6 months (right)

### P200

### Determinants of Vertebral Fracture Risk in Systemic Mastocytosis: The Central Role of TBS

Anna Atlasova[1,2], Marta Zampogna[1,2], Alberto Ghielmetti[3], Sara Rondinella[1,2], Giorgia Grassi[1], Mariarita Sciumè[4], Giovanna Mantovani[2,4], Francesco Passamonti[4,5], Cristina Eller Vainicher[1,2]
[1]Fondazione IRCCS Ca' Granda Ospedale Maggiore Policlinico, Endocrinology, Milan, Italy
[2]University of Milan,
Department of Clinical Sciences and Community Health – Dipartimento di Eccellenza 2023–2027, Milan, Italy
[3]University Hospital Santa Maria della Misericordia, Endocrinology and Metabolism, Udine, Italy
[4]Fondazione IRCCS Ca' Granda Ospedale Maggiore Policlinico, Ematology, Milan, Italy
[5]University of Milan, Department of Oncology and Hemato-Oncology, Milan, Italy

### Abstract Text

**Background:** Bone fragility is a relevant complication in systemic mastocytosis (SM), and bone mineral density (BMD) often underestimates fracture risk. We aimed to identify clinical and skeletal determinants of vertebral fractures (VF), with a focus on trabecular bone score (TBS).

**Methods:** We retrospectively analysed 113 SM patients. VF were assessed morphometrically. Clinical, biochemical, densitometric and microarchitectural variables were examined using univariate statistics and multivariate logistic regression. Age was dichotomised at 50 years based on ROC analysis.

**Results:** VF prevalence was 30%. Fractured patients were older (65.5 ± 12.0 vs 50.1 ± 14.3 years; $p < 0.001$) and had lower TBS (1.142 ± 0.137 vs 1.283 ± 0.120; $p < 0.001$). Although lumbar and femoral T-scores were reduced in fractured patients, BMD showed no independent association with VF. Sex distribution did not differ between groups. In multivariate analysis, degraded TBS and age ≥50 years independently predicted VF (OR 3.18, p = 0.016; OR 6.41, p = 0.026), while diagnostic subtype was not associated with fractures. The model achieved 77.3% accuracy (specificity 81.8%, sensitivity 65.0%). A combined variable integrating age ≥50 with degraded TBS further strengthened prediction (OR 11.6, $p < 0.001$) and reached 80.5% accuracy with 93% specificity.

**Conclusions:** TBS and age were the only independent predictors of vertebral fractures in SM, whereas BMD was not informative. Fracture prevalence was high, particularly after midlife, and individuals with both older age and degraded TBS showed a markedly increased risk. These findings support incorporating TBS into routine skeletal assessment in SM.

### P201

### Possible Implication of Antispastic Treatment in Heterotopic Ossification: Baclofen Alters the Osteoblast-Adipocyte Balance

Ana Alonso-Pérez[1], Lorena Zas-Veiga[1], María Crugeiras-Sampedro[1], Verónica López[1], Andrés Pazos-Pérez[1], Djedjiga Ait Eldjoudi[1], María Guillán-Fresco[1], Alberto Jorge-Mora[1], María Piñeiro-Ramil[1],

*Rodolfo Gómez[1]*
*[1]Health Research Institute of Santiago de Compostela IDIS, Musculoskeletal Pathology Group, Santiago de Compostela, Spain*

**Abstract Text**

**Background:** Heterotopic ossification (HO) is a complication that frequently arises after neurological injuries. A large cohort study of patients with traumatic brain injury reported a higher incidence of HO in those receiving antispastics, although it remains unclear whether this reflects the severity of spasticity or a drug-specific effect. Osteoblastogenesis and adipogenesis are antagonistic processes, and an imbalance in this axis may favour HO. Clarifying how antispastic drugs influence this balance may help elucidate mechanisms contributing to their association with HO.

**Purpose:** This study aimed to determine whether baclofen, a commonly used antispastic, modulates lineage commitment of mesenchymal progenitors.

**Methods:** Adipogenic differentiation of C3H10T1/2 cells was induced for 7 days employing specific differentiation and maintenance mediums. During differentiation, cells were treated with 100 µM baclofen alone or in combination with the hypoxia-mimetic agent cobalt chloride ($CoCl_2$) or inflammatory stimuli (IL1β and LPS). Gene expression of adipogenic and osteochondrogenic markers was quantified using by real-time PCR.

**Results:** Baclofen significantly reduced the expression of all four adipogenic markers (FABP4, PLIN2, ADIPOQ, and PPARG) during adipogenic differentiation. In parallel, the expression of the osteochondrogenic markers COL2A, SOX9, COLX, BGLAP, and PTHR was increased. Hypoxia-mimetic treatment with $CoCl_2$ led to a reduction in two of the four adipogenic markers, whereas the combination of $CoCl_2$ and baclofen further decreased the expression of all four adipogenic markers and increased the expression of COL2, COLX, and BGLAP compared with $CoCl_2$ alone. Additionally, under inflammatory conditions, baclofen again resulted in a reduction in the expression of all adipogenic markers.

**Conclusions:** Baclofen consistently suppresses adipogenic differentiation and enhances osteochondrogenic gene expression in a pro-adipogenic environment. Importantly, the anti-adipogenic effect persists under hypoxic and inflammatory conditions, which are key microenvironmental cues in ectopic bone formation. These findings suggest that antispastic agents may exert direct lineage-modifying actions that contribute to the increased clinical risk of HO.

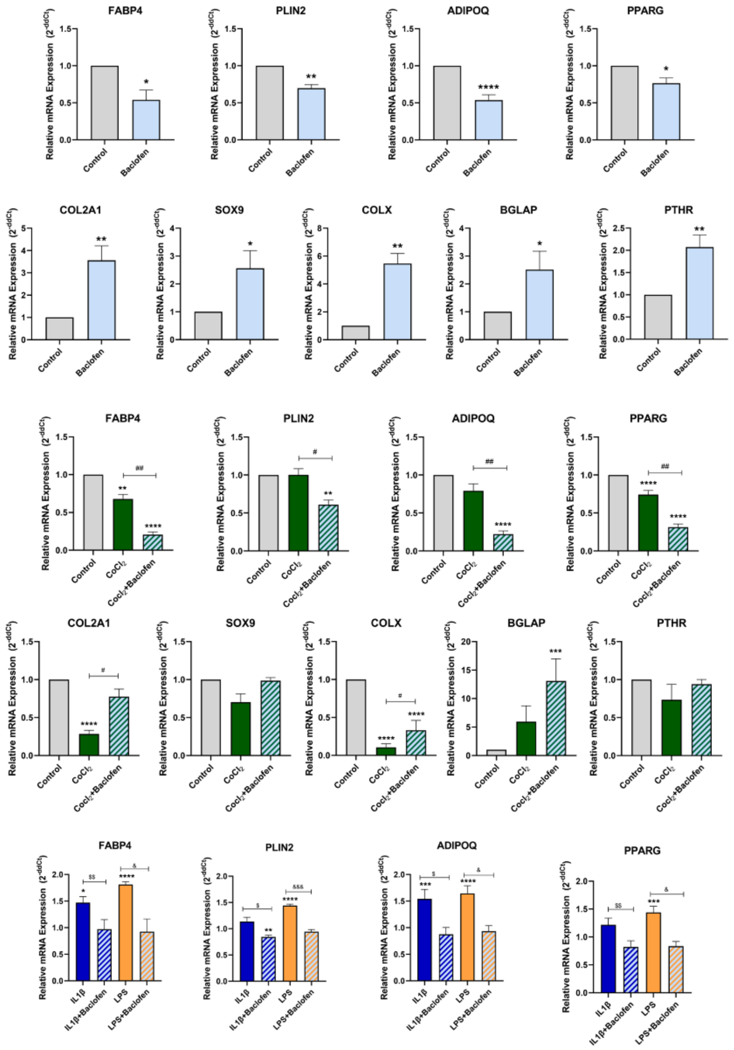

### P202

### Rates of electrolyte derangements associated with Ferric Derisomaltose amongst an adult inpatient cohort in a metropolitan Australia Health Service

*Huy Do[1], Aditya Mattu[1], Vivian Grill[1,2], Hanh H. Nguyen[1,2,3]*

*[1]Western Health, Department of Endocrinology & Diabetes, St. Albans, Australia*

*[2]The University of Melbourne, Department of Medicine, Parkville, Australia*

*[3]Monash University, Medicine- School of Clinical Sciences, Clayton, Australia*

### Abstract Text

**Background:** Significant hypocalcaemia and hypophosphataemia are increasingly recognised complications of iron preparations and antiresorptive therapies, particularly when co-administered[1]. Ferric derisomaltose(FDM) has a lower reported risk of electrolyte derangement than other formulations, but there is limited published real world data[2]. We aimed to evaluate rates of electrolyte disturbances following FDM, denosumab(Dmab), or both(FDM+Dmab), at our institution.

**Methods:** Inpatients>18 years who received FDM or Dmab between May 2024-January 2025 were identified through pharmacy records at a single centre. Demographic characteristics, comorbidities, and biochemistry at baseline and post-treatment were retrospectively collected from records. Analysis included descriptive statistics and chi-square testing.

**Results:** 292 cases were included(34 FDM, 225 Dmab, 33 FDM+Dmab). Vitamin-D deficiency and CKD stage=<3a affected 3.8% and 40.1%, respectively. Hypophosphataemia rate in FDM+Dmab group was double that of DMab(p0.044)(Table 1), while hypocalcaemia rates were similar. Electrolyte disturbances were low in the FDM alone group. 9.2% of abnormalities occurred in CKD stage3a or less. Nadir calcium and phosphate occurred up to 40 days post-treatment(median 9-12days).

**Conclusion:** Although rates of electrolyte disturbance with FDM alone is low, we identified a significantly higher rate with concurrent Dmab, with disturbances persisting up to 40 days. Further research is necessary to establish the optimal timing, surveillance interval, and management, when concurrent treatment is required.

| | **FDM(n=34)** | **FDM+Dmab(n=33)** | **Dmab(n=225)** |
|---|---|---|---|
| **Surveillance period, days** | 4(0-51) | 6(0-48) | 5(0-106) |
| **Hypophosphataemia(Total)** | 1(2.9%) | 8(24.2%) | 26(11.6%) |
| -Mild(0.65-0.75) | 1(2.9%) | 5(15.2%) | 13(5.8%) |
| -Mod(0.32-0.65) | 0 | 3(9.1%) | 12(5.3%) |
| -Severe(<0.32) | 0 | 0 | 1(0.4%) |
| **Nadir phosphate, mmol/L** | 0.70[0.70-0.70] | 0.66(0.37-0.74) | 0.67(0.30-0.74) |
| **Days to nadir phosphate** | 21(21-21) | 10(1-37) | 9(3-36) |
| **Hypocalcaemia(Total)** | 0 | 4(12.1%) | 29(12.8%) |
| mild(1.9-2.15) | 0 | 2(6.1%) | 23(10.2%) |
| severe(<1.9) | 0 | 2(6.1%) | 6(2.6%) |
| **Nadir calcium, mmol/L** | - | 2.00 | 2.04 |
| **Days to nadir calcium** | - | 12(4-40) | 11(6-36) |

Data presented as n(%) or median(min-max)

**P203**

**Citrate transport deficiency in osteolineage cells modulates osteocyte lacunar morphology without affecting the degree of bone matrix mineralization**

*Stéphane Blouin*[1], *Markus A. Hartmann*[1], *Nadja Fratzl-Zelman*[1], *Sonja Lueger*[1], *Thomas L. Clemens*[2,3], *Naomi Dirckx*[4]
[1]*Ludwig Boltzmann Institute of Osteology at the Hanusch Hospital of OEGK and AUVA Trauma Centre Meidling, 1st Medical Department Hanusch Hospital, Vienna, Austria*

[2]University of Maryland School of Medicine, Department of Orthopaedics, Baltimore, United States
[3]The Baltimore Veterans Administration Medical Center, Research and Development Service, Baltimore, United States
[4]Washington University in St. Louis, Department of Orthopaedic Surgery, St. Louis, United States

**Abstract Text**

**Background/Introduction:** Citrate constitutes roughly 5% of the bone's organic matrix and supports the intracellular energy production. Mice lacking *Slc13a5*, a membrane sodium citrate co- transporter required for extracellular citrate uptake, exhibit increased serum and mineral citrate level alongside reduced bone mass, bone mineral density and strength. However, the precise role of citrate in bone material characteristics remains unclear

**Purpose:** To determine the influence of citrate on bone matrix mineralization at the tissue level.

**Methods:** We investigated right femurs of 2-month-old male mice (n=13 WT, n=12 *Slc13a5*$^{-/-}$) by quantitative backscattered electron imaging (qBEI) using a scanning electron microscope to obtain calcium concentration maps with 1.1 µm spatial resolution. We analyzed bone mineralization density distribution (BMDD) in cortical mid-diaphysis (Ct.BMDD), epiphyseal spongiosa (Es.BMDD) and metaphyseal spongiosa (Ms.BMDD). Osteocyte lacunae sections (OLS) were characterized from binary images (thresholded at 5.2 weight% calcium). We measured OLS-density, OLS-porosity, OLS-area, OLS-perimeter, OLS-aspect ratio of the long and short axes of the lacunae.

**Results:** qBEI analysis revealed a BMDD shift towards lower mineralization density from cortical diaphyseal to epiphyseal and to metaphyseal bone in both WT and *Slc13a5*$^{-/-}$ mice due to a decrease in local tissue age. However, no differences in Ct.BMDD, Es.BMDD and Ms.BMDD were observed between WT and *Slc13a5*$^{-/-}$ animals. Similarly, OLS-porosity, -density, -area and -perimeter did not differ between genotypes. In contrast, *Slc13a5*$^{-/-}$ animals showed a markedly increased OLS aspect ratio (+10.5%, p=0.002) indicating a more elongated morphology.

**Conclusion(s):** The elevated mineral citrate levels in the *Slc13a5*$^{-/-}$ mouse model do not alter the local calcium content of the bone tissue; however, altered osteocyte lacunar morphology suggests modified collagen matrix organization similar to differences between primary and secondary lamellar bone or to the disorganized collagen lamellation in osteogenesis imperfecta. Follow-up spectroscopic analyses will determine potential effects on matrix characteristics.

**P204**

**CTX and PINP plasma levels have prognostic value in metastatic castration-resistant prostate cancer patients concerning mortality and progression – hereditary variation may play a role**

*<u>Simone Snitgård Rosendal Nielsen</u>[1], Ahmed Zedan Raid[2], Pernille Møller Bøttger[3], Budour Jedie[1], Palle JR Osther[4], Jonna S Madsen[3], Karin Nørgaard[2], Lone M Volmer[2], Torben F Hansen[2], Kent Søe[1]*
[1]University of Southern Denmark, Pathology Research Unit- Dept. of Clinical Research, Odense M, Denmark
[2]Vejle Hospital- University Hospital of Southern Denmark, Department of Oncology, Vejle, Denmark

[3]Vejle Hospital- University Hospital of Southern Denmark,
Department of Biochemistry and Immunology, Vejle, Denmark
[4]Vejle Hospital- University Hospital of Southern Denmark, Department of Urology, Vejle, Denmark

**Abstract Text**

**Introduction:** Bone is the primary site of metastasis in prostate cancer (PC), which through a vicious cycle induces bone disease. When patients become castration-resistant (mCRPC) they may receive anti-resorptive treatment. However, this is not to equally effective in all patients.

**Purpose:** We wished to investigate the prognostic value of CTX and PINP in mCRPC and the possible role of hereditary single-nucleotide polymorphisms (SNPs).

**Methods:** 48 mCRPC patients were followed for 2 years (approval S-20170011). All received first-line treatment for mCRPC and monthly zoledronic acid or denosumab. Blood samples for biomarker analyses were collected every 3 to 4 weeks, clinical follow-up every 8 to 12 weeks, and radiological follow-up every 9 to 16 weeks. 29 of the patients were analyzed for hereditary variation in 6 SNPs potentially related to severity of bone disease or response to anti-resorptive treatment. Non-responders were defined as ≥median for CTX (3 months) or PINP (5 months). Kaplan-Meier plots for overall survival, radiological-, and biochemical progression-free survival.

**Results:** Kaplan-Meier plots showed: 1) accelerated mortality for CTX- (HR 2.7, p=0.004) and PINP non-responders (HR 2.7, p=0.005) and 2) accelerated radiological progression for CTX non-responders (HR 3.4, p=0.001). SNP rs1038304 (*ESR1*) was coupled to accelerated mortality (HR 3.8, p=0.007) and accelerated radiological progression (HR 3.5, p=0.0099) for genotype AA vs GA (log-rank test). SNP rs1054016 (*TNFSF11*) showed near significance for accelerated mortality (HR 2.4, p=0.056) for genotype GG vs GT (Gehan-Breslow-Wilcoxon test). Multiple logistic regression (MLR) for death against baseline risk factors and responder/non-responder: CTX response p=0.051 & basic phosphatase p=0.0008; PINP p=0.016 & basic phosphatase p=0.007. MLR for radiological progression against baseline risk factors and responder/non-responder: CTX p=0.035; PINP p=0.0065.

**Conclusion(s):** CTX and PINP plasma levels have prognostic value in mCRPC concerning mortality and progression. Hereditary variation in *ESR1* and *TNFSF11* are also risk factors for accelerated disease.

**P205**

**Inter-Rater Agreement on Radiologic Features of Methotrexate-Associated Osteopathy: a blinded expert evaluation study**

*Judith Haschka[1], Martina Behanova[2], Tim F. Harr[3], Anton Sokhan[2], Svetlana Slavic[4], Barbara Hauser[5], Janina M. Patsch[6], Albrecht W. Popp[7], Bernhard Rintelen[8], Lothar Seefried[9], Heide Siggelkow[10,11], Jochen Zwerina[4], Roland Kocijan[3,12]*
*[1]Ludwig Boltzmann Institute Of Osteology,*
*Hanusch Hospital- 1st Medical Department- Vienna Bone and Growth Center, Vienna, Austria*
*[2]Ludwig Boltzmann Institute of Osteology, Hanusch Hospital of OEGK, Vienna, Austria*
*[3]Sigmund Freud University Vienna, Metabolic Bone Diseases Unit- School of Medicine, Vienna, Austria*

*[4]Ludwig Boltzmann Institute of Osteology, Hanusch Hospital of OEGK- 1st Medical Department, Vienna, Austria*
*[5]Western General Hospital, Rheumatic Diseases Unit- NHS Lothian, Edinburgh, United Kingdom*
*[6]Medical University of Vienna,*
*Division of General and Pediatric Radiology Department of Biomedical Imaging and Image-Guided Therapy, Vienna, Austria*
*[7]Bern University Hospital,*
*Division of Diabetes- Endocrinology- Nutritional Medicine & Metabolism UDEM Inselspital, Bern, Switzerland*
*[8]Lower Austrian State Hospital Stockerau,*
*2nd Medical Department- Karl Landsteiner Institute for Clinical Rheumatology, Stockerau, Austria*
*[9]University of Würzburg, Orthopedic Department, Vienna, Germany*
*[10]University Medical Center Göttingen,*
*Clinic of Gastroenterology- Gastrointestinal Oncology and Endocrinology, Göttingen, Germany*
*[11]Medical Care Center, Endokrinologikum, Göttingen, Germany*
*[12]Ludwig Boltzmann Institute of Osteology,*
*Hanusch Hospital of OEGK- 1st Medical Department- Vienna Bone and Growth Center, Vienna, Austria*

**Abstract Text**

**Introduction:** Methotrexate (MTX) is the most commonly used disease-modifying antirheumatic drug for autoimmune inflammatory diseases. A rare adverse effect, MTX osteopathy, causes insufficiency fractures of weight-bearing lower-extremity bones with characteristic radiologic features. This study evaluated inter-rater agreement on radiological signs of MTX-related osteopathy in patients with stress fractures or bone marrow edema (BME), comparing those with and without MTX treatment.

**Methods:** In this cross-sectional expert rating study, MRI scans of 14 de-identified patients were evaluated by eight specialists in orthopedics/trauma surgery, radiology, and internal medicine with osteology/rheumatology focus. All raters were blinded to the patients' medical history, medication, and imaging of other anatomical sites, and only one anatomical region per patient was assessed. Experts independently assessed (i) presence of IF, (ii) predefined radiological signs (BME, fracture line, sclerosis, periosteal reaction), (iii) suspected association with MTX, and (iv) certainty of judgement. Inter-rater agreement for primary outcomes was quantified using Fleiss' kappa(κ). Secondary outcomes included majority agreement proportions and descriptive analysis of certainty ratings.

**Results:** The 14 patients (median age 56 years; 71% female) included five on MTX (20–25 mg/week, median 8.5 years). Overall agreement for IF presence was moderate (κ=0.56). Agreement for specific radiologic signs varied: BME (κ=0.39), fracture line (κ=0.56), sclerosis (κ=0.06), and periosteal reaction (κ=0.05). Agreement on suspected MTX association was fair (κ=0.22), improving to moderate (κ=0.40) after excluding one expert who omitted responses. Per-patient agreement ranged widely (IF:21–100%; MTX association:25–88%). High agreement on "no association" corresponded exclusively to non-MTX cases, while among cases with "suspected association," 3/5 patients were indeed on MTX. Two MTX-treated patients showed evenly split assessments.

**Conclusion:** Because experts were fully blinded to patients' MTX status, the observed variability indicates that MTX-associated osteopathy cannot be reliably identified based on radiologic features alone. This underscores that, imaging findings have to be interpreted in clinical context, for diagnosing MTX-related osteopathy.

**P206**

### Methotrexate osteopathy among patients with stress fractures and bone marrow edema: a three year retrospective analysis from two specialist centers

*Svetlana Slavic*[1,2,3], *Tim Fabian Harr*[1], *Martina Behanova*[3], *Anton Sokhan*[3], *Jochen Zwerina*[1,3], *Judith Haschka*[1,3,4], *Roland Kocijan*[1,2,3,4]
[1]*Hanusch Hospital, 1st Medical Department, Vienna, Austria*
[2]*School of Medicine- Sigmund Freud University Vienna, Metabolic Bone Diseases Unit, Vienna, Austria*
[3]*Hanusch Hospital, Ludwig Boltzmann Institute of Osteology, Vienna, Austria*
[4]*Hanusch Hospital, Vienna Bone and Growth Center, Vienna, Austria*

### Abstract Text

**Introduction:** Methotrexate-associated osteopathy (MTXO), a rare adverse effect of methotrexate (MTX) therapy, is likely under-recognized. We assessed the frequency of MTXO among patients presenting with stress fractures or bone marrow edema (BME) and described clinical characteristics of affected patients.

**Methods:** We conducted a retrospective analysis of records from two specialist rheumatology/osteology centres in Vienna, Austria. Medical records were screened for ICD diagnosis stress fracture and MTX exposure over three years (October 2020–November 2023). Patients with ongoing or prior MTX who developed stress fracture/BME were included.

**Results:** Among 69 patients with stress fracture/BME, 5 (7.2%) had MTX exposure - four at diagnosis and one stopped approximately 1 year earlier. All MTXO patients were female; median age was 65 years (IQR 56–77.5) and median body mass index 27.4 kg/m² (IQR 19–37). Underlying conditions were rheumatoid arthritis (n=3), systemic sclerosis (n=1), and Crohn's disease (n=1). Vitamin D, parathyroid hormone, and calcium were within reference ranges in all except the patient with Crohn's disease, who was hypocalcaemic due to malabsorption. MTXO patients had median T-scores within the osteopenic range at both the lumbar spine and femoral neck. None received glucocorticoids at diagnosis or within the preceding two years. Median MTX dose was 20 mg/week (IQR 20–22.5), and median time to first fracture was 11 years (IQR 7.5–15.5). Tibial fractures occurred in all MTXO patients (100%); three (60%) had multiple bilateral fractures and two had unilateral tibial fractures. The foot was the second most common fracture site.

**Conclusions:** In this specialist cohort, MTXO accounted for 7.2% of stress fracture presentations, with a predilection for the tibia and frequent multifocal involvement. MTXO should be considered in long-term MTX users, particularly older women, who present with tibial or foot pain/stress fractures, even in the absence of glucocorticoid therapy or metabolic abnormalities.

**P207**

### Impact of Renal Dysfunction and Obesity on Symptom Burden in Moderate-to-Severe Carpal Tunnel Syndrome: Crossectional Analysis of 124 Patients from the Institute for Orthopaedics Banjica

*Maja Velinovic*[1], *Violeta Bascarevic*[2], *Predrag Nedeljkovic*[2], *Milos Vucetic*[2], *Milan Stajic*[2], *Zeljko Curcic*[2], *Andjelka Milic*[3], *Marko Stojanovic*[4]
[1]*Institute for Orthopaedics Banjica, Internal Medicine Department-Bone Metabolism Unit, Belgrade, Serbia*
[2]*Institute for Orthopaedics Banjica, Department of Plastic and Reconstructive Surgery, Belgrade, Serbia*
[3]*Institute for Orthopaedics Banjica, Department of Laboratory Medicine and Pathobiology, Belgrade, Serbia*
[4]*Clinic for Endocrinology- Diabetes- and Metabolic Diseases- University Clinical Center of Serbia- Medi cal Faculty University of Belgrade, Department of Neuroendocrinology, Belgrade, Serbia*

**Abstract Text**

**Introduction:** Carpal tunnel syndrome (CTS) is influenced by mechanical, metabolic, and neurovascular factors. Obesity and impaired renal function may contribute to median nerve vulnerability. It remains unclear whether these systemic conditions affect symptom severity and functional disability in patients with established CTS.

**Purpose:** This study aimed to examine the impact of obesity and renal function on symptom severity and functional status in moderate-to-severe CTS.

**Methods:** This cross-sectional study included 124 adults with electrophysiologically confirmed moderate-to-severe CTS. BMI, history of diabetes, serum creatinine, eGFR, Functional Status Score (FSS), and total Boston Carpal Tunnel Questionnaire (BCTQ) score were analyzed in all patients. Symptoms (night pain, daytime pain, numbness, weakness, tingling) were dichotomized (1–2 = mild; 3–5 = moderate/severe). BMI was graded as normal BMI (18.5–24.9 kg/m²) vs obese (≥30 kg/m²) and CKD was established by eGFR <60ml/min.

**Results:** Symptom intensity did not differ significantly between obese and normal-weight patients for nocturnal pain (p=1.00), daytime pain (p=0.44), numbness (p=0.56), weakness (p=0.87) or tingling (p=0.64). Similarly, CKD was not associated with higher symptom burden across any domain (all p>0.25). However, patients with CKD demonstrated significantly worse functional status (FSS 3.29 vs 2.74; p=0.0097) and a trend toward a higher total BCTQ score (63.5 vs 57.0; p=0.075). No functional differences were observed between normal-weight and obese patients (FSS 2.90 vs 2.69; p=0.57).

**Conclusion:** In patients with established moderate-to-severe CTS, neither obesity nor CKD significantly influences individual symptom intensity. However, CKD is independently associated with worse functional disability. Renal impairment may therefore affect global hand performance rather than isolated symptom domains, underscoring the importance of systemic evaluation in CTS management.

**P208**

### Suspected Hypophosphatasia without (likely) pathogenic ALPL variants: Results from genome sequencing in 35 patients and comparison with genetically and clinically confirmed Hypophosphatasia

*Oskar Windels*[1], *Florian Dudde*[1], *Pierre Huber*[1], *Mikolaj Bartosik*[1], *Alexander Simon*[1], *Timur Yorgan*[1], *Uwe Kornak*[2], *Florian Barvencik*[1]
[1]*University Medical Center Hamburg Eppendorf, Department of Osteology and Biomechanics, Hamburg, Germany*
[2]*University Medical Center Göttingen, Institute for Human Genetics, Hamburg, Germany*

**Abstract Text**

**Background:** Hypophosphatasia (HPP) is a rare disorder with multisystemic manifestations. In patients with suspected HPP, the detection of a (likely) pathogenic *ALPL* variant can help confirm the diagnosis when further diagnostic criteria are met. However, in a group of patients with persistent signs and symptoms of HPP, no variant was found (NVF) by Sanger sequencing, nor exome sequencing (ES).

**Purpose and Methods:** To better characterize this group of patients with no definite diagnosis and suspected but not genetically confirmed HPP, we conducted genome sequencing (GS) in 35 patients. These patients were compared with a control group of 70 age, sex, and BMI-matched individuals with clinically and genetically confirmed HPP. The retrospective comparison included laboratory parameters, HR pQCT, DXA, muscle function, and quality of life.

**Results:** GS identified ACMG class IV-V variants in 7 genes, class III variants in 17 genes, and 11 CNVs, with some patients having multiple variants. Notably, zinc levels, a cofactor of the metalloprotease ALP, were lower within the NVF group, which may partially explain the reduced ALP activity observed in the NVF group. Furthermore, NVF patients consistently showed ALP values within or below the lower reference range and PLP values within or above the upper reference range. Nevertheless, ALP levels in the NVF group were significantly higher, and PLP levels were significantly lower, than those observed in the HPP cohort. No differences we found regarding DXA and microarchitectural outcomes, with both groups being close to the reference median.

**Conclusion:** In summary, the cause of the HPP-like constellation of signs and symptoms in this group remains unclear, as no definitive genetic variants were identified. However, zinc levels, a cofactor of the metalloprotease ALP, were lower in the group with NVF, which might lead to reduced ALP activity in patients without detectable *ALPL* variants.

**P209**

### Bone-Muscle Alterations in a Triple Transgenic Mouse Model of Alzheimer's Disease: Early Manifestations of AD

*Giuseppina Storlino*[1], *Francesca Posa*[1], *Stefania Dell'Endice*[1], *Graziana Colaianni*[2], *Federica Piccolo*[1], *Tommaso Cassano*[3], *Maria Grano*[2], *Giorgio Mori*[1]
*[1]University of Foggia,*
*Department of Clinical and Experimental Medicine- University of Foggia- Viale Pinto 1- 71122 Foggia- Italy, Foggia, Italy*
*[2]University of Bari, Department of Precision and Regenerative Medicine and Ionian Area DiMePRe-J- University of Bari Aldo Moro- Piazza Giulio Cesare 11- 70124 Bari- Italy, Bari, Italy*
*[3]University of Foggia,*
*Department of Medical and Surgical Sciences- University of Foggia- Viale Pinto 1- 71122 Foggia- Italy, Foggia, Italy*

**Abstract Text**

Alzheimer's disease (AD), osteoporosis, and muscle atrophy are interconnected and frequently coexist in the elderly. However, the link between these conditions has been barely studied. Previously, we characterized the skeletal phenotype of the triple transgenic mouse model of AD (3xTg-AD) harboring three mutant genes: β-amyloid precursor protein (βAPPSwe), presenilin-1 (PS1M146V), and tauP301L. This model, characterized by the onset of the first neuropathological signs at six months, already showed cortical bone loss and reduced bone geometric properties at two months, suggesting that skeletal fragility is an early event in the pathogenesis of AD. At this stage of our investigation, we aimed to identify the causes of cortical bone reduction and to describe the muscular phenotype of these mice. Histological analysis of bone segments of 2-month-old AD male mice revealed that cortical bone impairment was related to an increase in the percentage of Empty Osteocyte Lacunae (+45%, *p=0.0234) and a simultaneous reduction in the number of TRAP+ Osteocytes (-29%, *p=0.0361), suggesting an impaired osteocytic osteolysis. Moreover, AD mice exhibited a significant enhancement in the percentage of Osteoclast Number (+93%, *p=0.0474) and in the percentage of bone surface covered by osteoclasts (+2.3-fold, *p=0.0187) in cortical bone, but not in trabecular bone (p=0.4351 and p=0.9386, respectively). Interestingly, we also observed a reduction in Muscle Fiber cross-sectional area (CSA) (-54%, **p=0.0014) without changes in Fiber Number (p=0.8447) in the quadriceps of AD mice compared with non-transgenic littermates. In conclusion, our study demonstrates that in two-month-old 3xTg-AD mice, although plaque development is still absent, cortical bone loss arises prior to the accumulation of these distinctive pathological lesions and is caused by osteocyte dysfunction and increase in osteoclast number, accompanied by early-onset muscle atrophy.

**ND-P027**

**Deferasirox-induced Fanconi Syndrome in an 80-year-old patient with chronic lymphocytic leukaemia**

*Daniel Grigorie*[1], *Alina Sucaliuc*[1]
*[1]National Institute of Endocrinology, Endocrinology, Bucharest, Romania*

**Abstract Text**

Deferasirox is an oral iron chelator widely used to treat iron overload in transfusion dependent patients. The common side effects of deferasirox therapy include rises in serum creatinine but proximal tubular dysfunction, Fanconi syndrome (FS), is less known.

**Case presentation:** The patient was admitted to our bone department in great pain, mostly at the thoraco-lumbar junction, with difficult and assisted ambulation. He was in remission for chronic lymphocytic leukemia, treated with chronic cyclosporin and deferasirox 0.72 g/day in the last 6 months, having more than 20 transfusions in the past years for severe anemia. He lost 5 kg in the last months, almost anorectic and accused severe fatigue. He had other comorbidities: type 2 diabetes on insulin (HbA1c= 5.63%), previous total thyroidectomy for papillary carcinoma (TSH 1.22 uUI/ml, thyroglobulin 0.326 ng/mL) osteoporosis with recent multiple vertebral fractures. An MRI revealed a new complete fracture at T12; the biochemistries: Hg 10.8 g/dl, creatinine 1.14 mg/dL eGFR 61ml/min, moderate hypophosphatemia (1.6-1.8 mg/dl), Alkaline phosphatase 168 U/L (38-129 U/L), C-telopeptide 1.26 ng/ml and normal calcium, 25OHD, PTH. Iv zoledronate was administrated and insulin and deferasirox were stopped because of recurrent hypoglycemia and 24-hour urine showed glucose 17 g, proteins 1 g, phosphate 0.96 g, TmP/GFR= 0.89 mg/dl; FGF23 8.31 pg/ml (23.2 - 95.4 / pg/mL). Our presumptive diagnosis was FS due possibly to deferasirox and alfacalcidol 2 mcg/day and milk were recommended. One month later serum P 4.3 mg/dl (2.5 - 4.5 / mg/dL) without treatment and urine protein 285 mg/24 h, no glucose, eGFR 88 ml/min. There was progressive improvement of bone pain, posture and gait.

**Conclusion:** We presented a case of FS induced by deferasirox which went into remission after stopping the treatment. We suggest that monitoring patients on chronic treatment with deferasirox should include serum phosphate and urine analysis.

### Poster Sessions: Paediatric bone diseases

### P211

### Primary hyperparathyroidism in an adolescent presenting with predominant bone manifestations: a case report

*Svetlana Bacetic*[1], *Aleksandar Stojanovic*[2], *Maja Velinovic*[2], *Vesna Jovanovic*[2]
[1]*Institute of Orthopedics Banjica, Pediatric orthopedics, Belgrade, Serbia*
[2]*Institute of orthopedics Banjica, General orthopedics, Belgrade, Serbia*

**Abstract Text**

**Background:** Primary hyperparathyroidism (PHPT) is rare in the pediatric population but can manifest with significant bone and metabolic complications. Adolescents undergoing rapid skeletal growth demand higher calcium (Ca) and phosphate (P) for bone mineralization, predisposing them to more pronounced bone lesions when PHPT occurs.

**Methods and Results:** We present a 17-year-old girl with prolonged subfebrile fever, abdominal pain, refractory hypertension, mood disturbances, and multiple cystic bone lesions. Initial workups focused on infectious, hepatobiliary, and endocrine causes without unifying diagnosis. Markedly elevated serum calcium (up to 3.97 mmol/L), PTH (1,170 pg/mL), and ALP (1,436 IU/L), alongside imaging evidence of a hyperfunctioning parathyroid adenoma and osteolytic bone cysts, confirmed PHPT. She underwent successful parathyroidectomy with rapid normalization of biochemical parameters and gradual regression of bone lesions. At six-month follow-up, the patient reported normalized appetite, weight gain, resolution of bone pain, and stabilized blood pressure. Radiographic monitoring showed gradual filling of prior bone cysts. No further orthopedic surgery was required.

**Conclusion:** Early consideration of PHPT in adolescents presenting with nonspecific symptoms such as subfebrile temperatures, abdominal pain, psychiatric disturbances, and especially bone lesions is vital. A multidisciplinary approach combining clinical assessment, biochemical screening (serum Ca, P, PTH, ALP), imaging (neck ultrasound, sestamibi scan), and timely surgical intervention can restore metabolic balance, allow bone healing, and prevent unnecessary morbidity.

**P212**

**Early-onset Hypophosphatasia**

*Anna Pujol Manresa[1], Mariya Sigatullina Bondarenko[1], Marta Ocaña Rico[1], Marta Murillo Valles[1], Juan Navarro Moron[1], Joan Calzada Hernández[2], Laia Gifre[3]*
*[1]Hospital Universitari Germans Trias i Pujol, Pediatrics, Badalona, Spain*
*[2]Hospital Sant Joan de Déu, Pediatrics, Barcelona, Spain*
*[3]Hospital Universitari Germans Trias i Pujol, Rheumatology, Badalona, Spain*

**Abstract Text**

**Background:** Hypophosphatasia (HPP) is an ultra-rare metabolic bone disease caused by deficient tissue-nonspecific alkaline phosphatase (TNSALP) activity due to *ALPL* mutations. The neonatal and infantile forms may present with life-threatening complications, including respiratory distress, skeletal hypomineralization, cranial abnormalities, and pyridoxine-responsive seizures.

**Case Presentation:** We report a 7-day-old male neonate, born to consanguineous parents, who presented with a 2–3-minute paroxysmal episode characterized by perioral cyanosis, downward gaze deviation, environmental unresponsiveness, and generalized hypertonia. He was afebrile and previously asymptomatic. On arrival to the emergency department, vital signs and physical examination were normal. During observation, he developed apneic episodes with desaturation and two similar paroxysmal events.

The patient was admitted to the neonatal intensive care unit and was started on phenobarbital. Laboratory studies of blood, urine and CSF revealed no infectious or metabolic disturbances. However, markedly low alkaline phosphatase (7 U/L) prompted initiation of pyridoxine, allowing discontinuation of phenobarbital without recurrence of events. Elevated plasma pyridoxal-5-phosphate and urinary phosphoethanolamine supported the suspicion of HPP.

Cranial ultrasound, brain MRI, EEG, and ophthalmologic assessment were normal. Skeletal survey initially showed no abnormalities. Genetic testing confirmed *ALPL* homozygosity for the pathogenic variant p.(E291Q). Both parents were heterozygous carriers.

During follow-up, the patient developed progressive craniosinostosis with macro- and dolichocephaly, occipital bone spurs compromising the posterior fossa and downward displacement of cerebellar structures, for which he has undergone corrective surgery. Moreover, he developed a bell-shaped thorax with resting tachypnea, and a premature tooth loss with minimum trauma at 9 months.

**Conclusions:** Low alkaline phosphatase levels in neonates with paroxysmal events should raise suspicion for HPP, a clinically heterogeneous disorder with potentially severe outcomes. Early recognition is crucial given the availability of enzyme replacement therapy (asfotase alfa), for which this patient is currently awaiting approval.

**P213**

**Parathyroid adenoma in Ollier disease caused by a somatic IDH1 variant**

*Tanja Fritz*[1], *Rodrigo Montero-Lopez*[1], *Markus A. Hartmann*[2], *Stephane Blouin*[2], *Gerald Webersinke*[3], *Mario Scala*[4], *Lena Rossetti*[5], *Julian Schirl*[1], *Marcell Tamas*[1], *Nadja Fratzl-Zelman*[2], *Wolfgang Högler*[1]
[1]*Johannes Kepler University Linz,*
*Centre for Growth and Osteology- Department of Paediatrics and Adolescent Medicine, Linz, Austria*
[2]*Ludwig Boltzmann Institute of Osteology at Hanusch Hospital of OEGK and AUVA Trauma Centre Mei dling, 1st Med. Dept. Hanusch Hospital, Vienna, Austria*
[3]*Ordensklinikum Linz, Laboratory for Molecular Genetic Diagnostics, Linz, Austria*
[4]*Johannes Kepler University Linz, Central Radiology Institute, Linz, Austria*
[5]*Johannes Kepler University Linz, Department of General Surgery, Linz, Austria*

**Abstract Text**

**Introduction:** Ollier disease (OD; OMIM 166000) is a rare skeletal disorder characterized by multiple enchondromas, most commonly caused by somatic heterozygous *IDH1* variants that increase production of the oncometabolite D-2-hydroxyglutarate. This metabolite drives epigenetic dysregulation, abnormal mesenchymal proliferation, and enhances chondrogenesis.
Somatic *IDH1* variants are well documented in chondrosarcomas, gliomas, and myeloid neoplasms, whereas endocrine tumors with *IDH1* variants are extremely rare, with only isolated pituitary adenomas or juvenile ovarian granulosa cell tumors reported. Histologically, enchondromas show chondroid matrix, persistent cartilage islands, and reduced mineralization. Cartilage components may also appear in radiologically unaffected bone, reflecting the mosaic nature of OD. We report the first case of a parathyroid adenoma with *IDH1* mutation in OD, providing new insights into disease pathophysiology.

**Case presentation:** A Caucasian girl, diagnosed with OD at 8 years of age due to multiple limb enchondromas, presented with persistent hyperparathyroidism. A parathyroid adenoma was identified at age 12 years. Surgical excision was performed together with biopsy of an enchondroma and radiologically unaffected bone. Genetic testing revealed an *IDH1* c.394C>T (R132C) variant in both the enchondroma and the parathyroid adenoma. A transiliac bone biopsy sample was obtained and even though the region appeared enchondroma free on radiography, bone histology revealed the presence of multiple highly mineralized cartilage inclusions scattered throughout the trabecular and cortical bone matrix.

**Discussion:** Our report adds to the pathophysiological understanding of OD as a mosaic disorder with malignant potential. We first report that, similar to other mosaic conditions such as Pseudohypoparathyroidism (*GNAS*-mutation), *IDH1*-mutated parathyroid tissue can give rise to adenomas. The presence of hypermineralized cartilage in radiologically unaffected bone likely reflects early embryonic *IDH1* mutations persisting in mesenchymal precursors, impairing osteogenic differentiation via D-2-hydroxyglutarate and enabling lifelong enchondroma formation. These observations highlight the need for interdisciplinary care in OD, including regular screening for endocrine tumors.

**P214**

**Bone Mineral Density and Vitamin D Status in Non-Ambulatory Adolescents with Duchenne Muscular Dystrophy**

*Yaryna Stepaniuk[1], Nataliya Balatska[2], Nataliia Grygorieva[3], Alla Antoshkina[2], Musiienko Anna[3], Tetiana Shkliarska[4], Maryna Kozelkova[5], Daryna Kurylo[3]*

*[1]National Specialized Children's Hospital «Okhmatdyt» of the Ministry of the Health of Ukraine, Pediatric Department, Kyiv, Ukraine*

*[2]Bogomolets National Medical University, Pediatrics Department No 1, Kyiv, Ukraine*

*[3]D.F. Chebotarev Institute of Gerontology of the National Academy of Medical Sciences of Ukraine, Department of Clinical Physiology and pathology of the musculoskeletal system, Kyiv, Ukraine*

*[4]National Specialized Children's Hospital «Okhmatdyt» of the Ministry of theHealth of Ukraine, Center of Orphan Diseases and Gene Therapy, Kyiv, Ukraine*

*[5]National Children's Specialized Hospital "Okhmatdyt" of the Ministry of Health of Ukraine, Medical Statistics Department, Kyiv, Ukraine*

**Abstract Text**

**Background:** Duchenne muscular dystrophy (DMD) is a severe neuromuscular disorder characterized by progressive muscle degeneration, ultimately resulting in complete loss of mobility and, eventually, premature death. The disease's pathological mechanisms, combined with prolonged glucocorticoid use and advancing immobility, contribute to the deterioration of bone tissue, leading to secondary osteoporosis, which markedly increases the risk of pathological fractures.

The **objective** was to evaluate bone mineral density (BMD) and vitamin D status, and to assess the prevalence of osteoporosis in non-ambulatory adolescents with DMD.

**Materials and methods:** The study involved 13 patients aged 11-18 years diagnosed with DMD from various regions of Ukraine. Anthropometric measurements were done using standard clinical techniques. BMD was assessed at the lumbar spine and total body using dual-energy X-ray absorptiometry (DXA) on a Hologic Discovery Wi. Lateral thoracolumbar spine radiography was performed to verify vertebral fractures. Vitamin D status was assessed by serum dihydroxyvitamin D (25(OH)D) level.

**Results:** According to anthropometric results, 38.5% had normal height, 30.8% were within the range of -1 to -2 SD, and growth retardation was noted in 30.8%. Body mass index (BMI) within the normal range was in 30.8%, 53.9% were classified as overweight, 46.2% had obesity, and 15.5% were underweight. DXA results showed that 84.6% of the patients had low BMD. Secondary osteoporosis was diagnosed in 38.5%. Four patients (30.8%) had multiple vertebral fractures during glucocorticoid therapy at a dose of 20-30 mg/day. One patient had low bone BMD and a history of low-energy fractures of both femurs. Only two patients (15,5%) had normal BMD. Low 25(OH)D levels were found in 61.5% of adolescents, and 15.3% had vitamin D deficiency.

**Conclusions:** The high frequency of low BMD among patients with DMD underscores the importance of routine DXA scans, vertebral morphometry, and monitoring of serum 25(OH)D levels, especially for individuals receiving glucocorticoid treatment.

**P215**

### Biochemical and Ultrasound Markers of Bone Health in Children with Cerebral Palsy

*<u>Cristina Dettori</u>[1], Agnese Giamborino[1], Valentina Vitelli[1], Leonardo Galfo[1], Sabina Frascarelli[1], Vittoria Carnicelli[1], Pasquale Comberiati[2], Giuseppina Sgandurra[3], Alessandro Saba[1], Federica Saponaro[1]*
*[1]University of Pisa, Department of Surgical- Medical and Molecular Pathology and Critical Care Medicine- University of Pisa- Pisa Italy, Pisa, Italy*
*[2]University of Pisa, Department of Clinical and Experimental Medicine, Pisa, Italy*
*[3]University of Pisa - IRCCS Stella Maris Foundation, IRCCS - Stella Maris Foundation, Pisa, Italy*

### Abstract Text

**Background:** Cerebral Palsy (CP) is the most common chronic disability in childhood, associated with severely compromised bone/muscle health due to brain lesions. These lesions result in motor, sensory, cognitive, feeding, and communication impairments, significantly affecting quality of life. Patients present low bone mineral density, vitamin D deficiency, increased risk of fragility fractures and sarcopenia.

Motor impairment is classified into five levels using the Gross Motor Function Classification System (GMFCS) and the Manual Ability Classification System (MACS).

Patients with CP underwent biochemical and bone/muscle health assessments. The aim of this prospective study is to investigate correlations between brain lesions, vitamin D levels, and biochemical markers of bone/muscle health.

**Materials and Methods:** Clinical data were collected, including weight, height, BMI, pubertal stage, diagnosis type, GMFCS and MACS levels. Serum calcium, parathyroid hormone (PTH), and 25(OH)D levels were measured to assess calcium metabolism. Vitamin D metabolites were analyzed using a novel HPLC-MS/MS method capable of simultaneously quantifying four metabolites in plasma: cholecalciferol, 25(OH)$D_3$, 1,25$(OH)_2D_3$, and 24,25$(OH)_2D_3$.

Bone and mineral health were evaluated using Quantitative Ultrasound (QUS), that measures the speed of sound (QUS-AD-SoS, m/s) and bone transmission time (QUS-BTT, µs) in the phalanges.

**Results:** A total of 51 patients aged 3-18 years were included: 21 females (mean age=9.7±4.6years, height=128.1±27.3cm, weight=26.7±12.8kg, BMI=15.3±2.1, Ca=9.64±0.483mg/dL, PTH=22.22±16.3ng/L)

and 30 males (mean age=10.3±3.6years, height=130.8±21.5cm, weight=26.7±11.5kg, BMI=15.2±2.5, Ca=9.83±0.338mg/dL, PTH=22.27±6.894ng/L). Overall, 80% of patients had a low BMI.

Regarding 25(OH)D levels, 61.9% of patients were below the clinical cut-off (20=ng/mL), 47.6% were below the median (14=ng/mL), and 19% had levels below 10=ng/mL. QUS parameters expressed as Z-scores showed values < -1 in 26.7% of patients.

**Conclusion:** The presence of hypovitaminosis D and reduced QUS parameters suggests bone fragility in patients with CP, underscoring the importance of regular monitoring of metabolic and bone health status.

**Poster Sessions: Rare bone diseases**

**P216**

**Progression of spinal fibrous dysplasia lesions in adults**

*Arnaud Vanjak[1], Amandine Laporte[2], Valérie Bousson[2], Roland Chapurlat[3], Thomas Funck-Brentano[4]*
*[1]APHP Lariboisière Hospital, Rheumatolgy, Paris, France*
*[2]APHP Lariboisière Hospital, Radiology, Paris, France*
*[3]Hospices Civils de Lyon, Rheumatology, Lyon, France*
*[4]APHP Lariboisière Hospital, Rheumatology, Paris, France*

**Abstract Text**

**Introduction:** Fibrous dysplasia (FD) is a rare genetic bone disorder caused by a post-zygotic activating mutation of *GNAS*, leading to replacement of normal bone tissue by a proliferative fibrous component. Spinal involvement is rare and may result in pain, scoliosis, fracture, or neurological complications. Most lesions stop growing after the age of 15, except in rare hormonal or mitotic contexts. However, we observed progression in several adult patients from our center, outside of these situations. We therefore decided to systematically assess the evolution of spinal fibrous dysplasia lesions in adulthood.

**Methods:** This is a prospective observational study conducted between 01/2023 and 01/2025 within our FD reference center. Patients >18 years old with a diagnosis of spinal FD, and with CT scans performed at least 3 years apart, were included. We collected clinical characteristics of the patients and their lesions (particularly spinal involvement), treatments used, as well as laboratory findings and imaging follow-up data.

**Results:** One hundred twenty-eight patients with FD from our center were included between 01/2023 and 01/2025. Among them, 16 presented with spinal involvement (12.5%). Two patients were excluded due to the absence of follow-up CT imaging. Among the remaining 14 patients, 9 were women. Dysplastic involvement was polyostotic in 12/14 patients (86%). Lesions were predominantly purely lytic (9/14, 64%) or mixed with sclerotic components (4/14, 29%). Spinal lesions were evenly distributed across the cervical, thoracic, lumbar spine and sacrum. With a median interval of 90 months (7.5 years) between two imaging studies, progression was observed in 10/14 patients (71%) according to RECIST 1.1 criteria.

**Conclusion:** In our cohort, when systematically assessed in adult patients with spinal fibrous dysplasia, lesion progression was frequent. A nationwide study across the entire FRANCEDYS registry (n = 1100) will help confirm and refine these findings, and, if necessary, modify patient surveillance strategies.

**P217**

**"Not Just More Porous: Vascular Channels and Osteocyte Lacunae in osteogenesis imperfecta mouse Bones"**

*Grégoire André*[1], *Antoine Chretien*[2], *Pierre-Louis Docquier*[2], *Greet Kerckhofs*[1], *Catherine Behets*[1]
[1]*UCLouvain, Morf, Brussels, Belgium*
[2]*UCLouvain, Irec, Brussels, Belgium*

**Abstract Text**

**Introduction:** Osteogenesis imperfecta (OI) is an uncommon congenital bone disorder often resulting from a mutation in one of the type I collagen genes. It is characterized by frequent fractures and bone deformities.

**Purpose:** Cortical porosity of the humerus of 23-week-old wildtype (wt, n=7) and osteogenesis imperfecta mice (oim, n=7) was assessed in order to explore its contribution to bone fragility.

**Methods**: Fracture incidence was documented with *in vivo* scanning at weeks 5, 11,17 and 23. After euthanasia at week 23, diaphyses of humerus from both groups underwent *ex vivo* Nano-CT analyses (1.5µm³) and cortical porosity was assessed by taking into account both the vascular channels and the lacuno-canalicular network of osteocytes.

**Results**: In oim, the total number of fractures increased by 5.1±2.5 fractures per mouse from age 5 to 23 weeks. The cortical porosity of oim humerus (2.6±0.4%) was significantly higher (p<0.001) than the wt one (1.3±0.3%). Furthermore, the distribution of this porosity was significantly different between both groups. Indeed, lacuno-canalicular network of osteocytes represented 66.6±8.1 % of total porosity in the wt group versus 47.7±7.4 % (p<0.001) in the oim. Porosity of the vascular channels was 33.3±8.1% and 50.35±9.4 % of total cortical porosity in the wt and oim groups, respectively (p<0.01).

**Conclusion**: Bone fragility, attested by the number of fractures in oim, can be explained not only by increased cortical porosity but also by an altered distribution of this porosity. Specifically, the higher vascular proportion than in wt may contribute to this fragility. Furthermore, the decreased osteocytic component suggest an impaired cellular communication within the bone matrix and, consequently, inefficient bone remodeling.

**P218**

**Analysis of the role of spermine synthase in bone metabolism**

*Yihao Zhu*[1], *Florian Barvencik*[1], *Ralf Oheim*[1], *Michael Amling*[1], *Thorsten Schinke*[1], *Timur Yorgan*[1]
[1]*University Medical Center Hamburg-Eppendorf, Department of Osteology and Biomechanics, Hamburg, Germany*

**Abstract Text**

**Introduction:** Spermine synthase (SMS) catalyzes the synthesis of spermine from spermidine in the polyamine metabolic pathway. Pathogenic variants of SMS cause the Snyder-Robinson-Syndrome (SRS), characterized by intellectual disability, muscular hypotonia, and skeletal abnormalities including osteoporosis. We have previously shown that male mice carrying the SRS-causing variant G56S display reduced bone mass due to decreased osteoblast activity. However, knowledge about the role of the X-linked *SMS* gene in females and the cellular mechanism of the skeletal effect is still limited.

**Material and Methods:** The musculoskeletal phenotype of heterozygous female *Sms*$^{+/G56S}$ mice was analyzed by µCT, histomorphometry. Functional studies in primary murine osteoblasts and transfected MC3T3-E1 cells were performed to obtain novel insights into the molecular pathomechanism.

**Results:** Compared to wildtype littermates, male 12-week-old female Sms$^{+/G56S}$ mice showed mild osteopenia in vertebral trabecular bone (BV/TV -24%, $p<0.05$) while cellular indices were not significantly altered. Transcriptomic microarray analysis of primary osteoblasts at day 10 of osteogenic differentiation identified glucose metabolism and oxidative stress response as strongly altered molecular pathways in addition to enchondral ossification. Functional glucose uptake and oxidative stress assays confirmed key aspects of these findings *in vitro*. Interestingly, MC3T3-E1 cells transfected with a G56S SMS expression plasmid displayed a significantly reduced expression of key osteoblast marker genes compared to cells transfected with WT SMS (*ALP*L -44%, *SP7* -48%, both $p<0.05$), while the top regulated genes identified in primary cells were not altered.

**Conclusion:** Our data indicate that a cell autonomous impairment of osteoblast differentiation and activity due to impaired glucose metabolism and increased oxidative stress contributes to the skeletal phenotype of SRS. Furthermore, our results highlight that also female carriers of pathogenic SMS variants may be at risk to develop low bone mass disorders.

**P219**

### Unravelling the osteocytes involvement in Cystic Fibrosis-related Bone Disease

*Léa Lagny[1], Christine Guillaume[1], Laurine Hamon[1], Guénaëlle Bouet-Chalon[2], Arnaud Vanden-Bossche[3], David Marchat[2], Frédéric Velard[1], Léa Thoraval[1]*
*[1]University of Reims Champagne-Ardenne, UR BIOS "Biomatériaux et inflammation en site osseux", Reims, France*
*[2]Mines Saint-Etienne- Univ Jean Monnet- Etablissement Français du Sang, INSERM- U 1059 Sainbiose, Saint-Etienne, France*
*[3]Université Jean Monnet Saint-Étienne- INSERM- Mines Saint Etienne, Sainbiose  U1059, Saint-Etienne, France*

**Abstract Text**

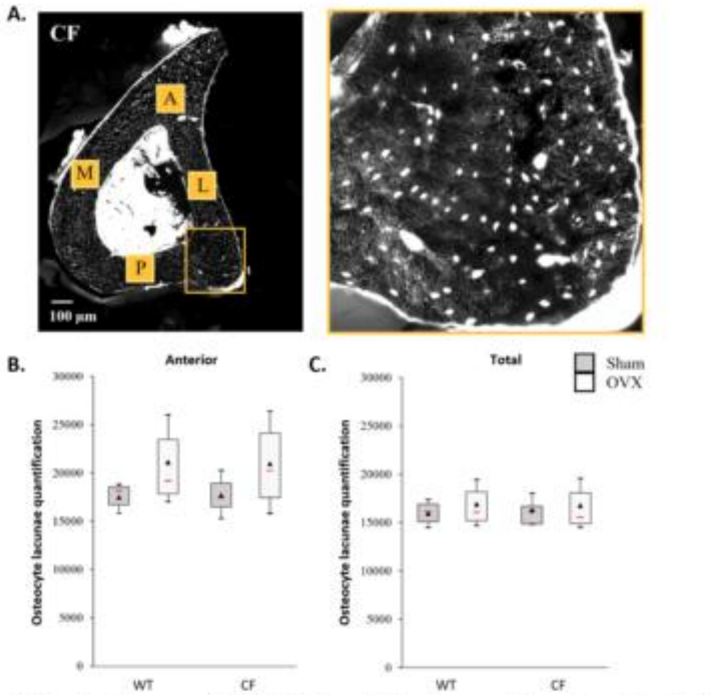

***Figure :*** **(A)** Confocal microscopy (Zeiss LSM800) acquisition of mouse tibia sections stained with Rhodamine 6G then tissue-cleared (×40 objective, ×0.5 zoom). Quantification of osteocyte lacunae in **(B)** the anterior tibial region as well as in **(C)** the four regions analyzed using IMARIS software (n=3-5).

**Background:** The life expectancy of people with cystic fibrosis (CF) continues to increase. Cystic fibrosis-related bone disease (CFBD) affects nearly 50% of patients, but the impact of aging on its development has not been described. While the effects of CFTR (whose mutation is responsible for the disease) dysfunction on osteoblasts [Delion, 2016; Dumortier, 2025] and osteoclasts [Jourdain, 2021] are documented, its impact on osteocytes, the sentinels of bone integrity and mineral homeostasis, remains unknown. The aims of this work are, i) to use an innovative *in vitro* model to study the impact of CFTR dysfunction on osteocytes, ii) to analyze osteocytes *ex vivo*, in CF(F508delCFTR) and non-CF mice, in an ovariectomy (OVX)-induced osteoporosis model.

**Methods:** Human primary mesenchymal stem cells (n=6 independent donors) were cultured on 3D bioceramics for 28 days [Juignet, 2017], without or with CFTR pharmacological inhibitors (10 µM, $Inh_{172}$, BPO-27). *In vitro*-formed tissue was investigated by Phalloidin-AlexaFluor®488-DAPI staining.

Osteoblast-to-osteocyte transition was analyzed by RT-qPCR and ELISA. Eight-week-old CF (n=9) or non-CF (n=6) mice were subjected to OVX (to mimic post-menopausal osteoporosis) or sham surgery. Tibiae were collected, stained with rhodamine 6G, cut and tissue-cleared for confocal microscopy to qualify and quantify the osteocyte lacuno-canalicular network.

**Results:** Phalloidin-DAPI staining shows dense actin networks in bioceramics grooves. Gene expression pattern analysis coupled with SOST secretion suggested concomitant mature osteoblasts and osteoid osteocytes inside our cultures. The effects of CFTR inhibitors require further clarifications. OVX surgery evidenced blunted bone microarchitecture in CF conditions as compared to wild type and sham counterparts. Confocal imaging of bone samples showed different osteocytes lacunae distribution in mineral matrix, in OVX *versus* sham samples across phenotypes, though total lacunae count remains similar (**see Figure**).

**Conclusion:** This work would shed light on osteocytes involvement in the deregulation of bone homeostasis in CF patients.

**P220**

**Adult Diagnosis of ARHR2: A Rare Case of Hereditary Hypophosphatemic Rickets**

*<u>Marco Onofrio Torres</u>[1], Gaetano Paride Arcidiacono[1], Andrea Rubin[1], Mor Peleg Falb[1], Martin Diogo[1], Francesca Guidolin[1], Paolo Simioni[1], Stefania Sella[1], Sandro Giannini[1]*
*[1]University of Padua, Clinica Medica 1- Department of Medicine, Padua, Italy*

**Abstract Text**

**Background**: Hypophosphatemic rickets comprises a heterogeneous group of disorders characterized by low serum phosphate and impaired bone mineralization. Clinical manifestations include skeletal developmental abnormalities, fragility fractures, and bone and muscle pain. Among the genetic forms, autosomal recessive hypophosphatemic rickets type 2 (ARHR2) is an exceptionally rare subtype caused by mutations in the ectonucleotide pyrophosphatase/phosphodiesterase 1 (ENPP1) gene, leading to overproduction of the phosphaturic hormone fibroblast growth factor 23 (FGF23). ENPP1 mutations may also be associated with generalized infantile arterial calcification (GACI), a condition often characterized by perinatal lethality.

**Case presentation**: We report the case of a 36-year-old woman presenting with chronic musculoskeletal pain since age 18. Her medical history included short stature and early-onset hip osteoarthritis requiring joint replacement. Family history revealed a sister who died in childhood and a maternal history of spontaneous miscarriage. Laboratory testing showed hypophosphatemia (1.9 mg/dL), reduced TMP/GFR (1.6 mg/dL), secondary hyperparathyroidism (PTH 65.7 ng/L), and increased bone turnover (bALP 37.9 µg/L), with normal 1,25-dihydroxyvitamin D levels (104 pmol/L). Bone densitometry revealed osteoporosis. Given the suspicion of a hypophosphatemic syndrome, serum intact FGF23 levels were measured (107 pg/mL) and found to be elevated. Due to the early onset of symptoms, next-generation sequencing was performed, revealing a homozygous ENPP1 mutation (Cys241Arg), confirming ARHR2; both parents were heterozygous carriers.

**Discussion and Conclusions**: This case illustrates an exceptionally rare form of hereditary hypophosphatemic rickets diagnosed in adulthood despite long-standing clinical manifestations. The differential diagnosis of hypophosphatemic disorders is challenging and requires FGF23 measurement to guide appropriate evaluation. Emerging therapeutic approaches, particularly ENPP1 replacement strategies, appear promising. INZ-701, a recombinant fusion protein consisting of the active ENPP1 domain linked to a human Fc fragment, is currently under regulatory evaluation and may offer future therapeutic opportunities for affected patients.

**P221**

**Interleukin-6 receptor inhibition with sarilumab in a bisphosphonate-resistant patient with polyostotic fibrous dysplasia**

*Olivier Malaise[1], Florence Rogister[2], Fanny Gester[3], Gilles Reuter[4], Bertrand Delvoie[5], Marie-Antoinette Ferrara[5], Clio Ribbens[1]*
*[1]CHU de Liège, Rheumatology department, Liège, Belgium*
*[2]CHU de Liège, Ear- Nose and Throat department, Liège, Belgium*
*[3]CHU de Liège, Pulmonary department, Liège, Belgium*
*[4]CHU de Liège, Neurosurgery department, Liège, Belgium*
*[5]CHU de Liège, Radiology department, Liège, Belgium*

**Abstract Text**

**Background:** Fibrous dysplasia is a rare skeletal disorder in which normal bone is replaced by fibro-osseous tissue, leading to pain, deformity and fragility fractures. Antiresorptive agents (bisphosphonates or denosumab) are the most used treatments. IL-6 receptor blockade has also been explored, based on IL-6 expression within lesions and its role in increased bone turnover. Tocilizumab has shown mixed results, with the TOCIDYS trial (Chapurlat, Bone 2022) reporting no overall change in bone pain, although a subset of patients experienced marked improvement.

**Case presentation:** We report the use of sarilumab, a fully human IL-6 receptor antagonist with higher binding affinity than tocilizumab, in a 35-year-old woman with polyostotic fibrous dysplasia. She presented vertebral, bilateral costal (causing lung compression and restrictive pulmonary syndrome), and cranial lesions involving the right temporal and sphenoid bones with narrowing of the optic nerve canal and mixed hearing loss. She initially received ibandronate from age 19. Due to worsening pain, treatment was switched to yearly zoledronate (5 mg), with initial benefit for 3 years (visual analog scale - VAS – decreased from 8 to 4) before pain relapse. An additional dose of zoledronate after 6 months provided no improvement.

**Results:** Sarilumab (200 mg subcutaneously every 2 weeks) was initiated and resulted in rapid and substantial improvement in costal bone pain (VAS decreased from 8 to 3), with bone alkaline phosphatase reduction from 130.6 to 92.6 U/L. After 10 months, clinical improvement was sustained, with stabilization of bone turnover markers and trends to reduction of some costal bone lesions volume. Treatment was well tolerated with no severe infectious complications.

**Conclusion:** This case suggests that IL-6 receptor inhibition may be an effective therapeutic option in selected patients with fibrous dysplasia refractory to bisphosphonates. To our knowledge, this is the first reported case of sarilumab use in fibrous dysplasia.

**P222**

**Modelling aberrant BMP signaling in Heterotopic ossification through human induced pluripotent stem cells (iPSC)**

*Judit Bestilleiro-Márquez*[1], *Daniel Rubiera*[1], *Lucia Llera*[1], *Marius Wits*[2], *Francesc Ventura*[3], *Gonzalo Sanchez-Duffhues*[1]
[1]*CSIC-CINN/ISPA, Tissue-specific BMP signalling group, Oviedo, Spain*
[2]*Leiden University Medical Center,*
*Department of Cell and Chemical Biology- Cardiovascular Cell Biology, Leiden, Netherlands*
[3]*Universitat de Barcelona, Department de Ciències Fisiològiques, Barcelona, Spain*

**Abstract Text**

**Background / Introduction:** Patients with Fibrodysplasia Ossificans Progressiva (FOP) experience episodic heterotopic ossification (HO) of connective soft tissues in response to inflammatory events or "flare-ups," ultimately leading to severe immobility and cardiorespiratory failure. HO lesions in FOP arise through dynamic interactions among multiple cell populations, including mesenchymal stem cell (MSC) bone progenitors, capillary-forming endothelial cells (ECs), and infiltrating immune cells. FOP is caused by ubiquitous mutations in *ACVR1*, which encodes the Bone Morphogenetic Protein (BMP) type I receptor ALK2. The predominant heterozygous single-point mutation, c.617G>A (p.R206H), confers neomorphic signaling properties: unlike the wild-type receptor, mutant ALK2 responds to circulating Activins by activating canonical BMP/SMAD signaling. To identify potential treatments for this currently incurable disease, robust humanized disease models are critically needed.

**Purpose:** To develop a humanized research platform that recapitulates the key cell types involved in FOP lesion formation.

**Methods:** Generation, maintenance, and differentiation of induced pluripotent stem cells (iPSCs) into mesenchymal stem/progenitor cells (MSC), endothelial cells (EC), and monocyte-like cells. Characterization was performed using brightfield imaging, western blotting, qPCR, and immunofluorescent staining.

**Results:** Using CRISPR/Cas9, we generated isogenic control and FOP iPSC colonies and confirmed their pluripotency. iPSCs were successfully differentiated into MSCs using a commercially available protocol, and into ECs and monocyte-like cells using adapted published methods. Cell morphology and expression of lineage-specific markers were validated by qPCR. All differentiated cell types exhibited aberrant responses to Activin A, consistent with known FOP mechanisms.

**Conclusion:** Refinement of cell culture and differentiation protocols enables the use of patient-derived iPSCs as a viable alternative for studying the cellular and molecular mechanisms underlying genetic HO. Further work is required to validate iPSC-derived MSCs, ECs, and monocytes as in vitro models for target identification, drug discovery, and toxicity assessment.

**P223**

**Novel methods to resemble biomechanical environmental cues in vitro applied to Fibrodysplasia ossificans progressiva.**

*Daniel Rubiera López*[1], *Gonzalo Sanchez-Duffhues*[1,2], *Judit Bestilleiro-Marquez*[1], *Lucía Llera*[1],
*Lucía Suárez*[1], *Francesc Ventura*[3]
[1]*Consejo Superior de Investigaciones Científicas CSIC,*
*Centro de Investigación en Nanomateriales y Nanotecnologías CINN, Oviedo, Spain*
[2]*Leiden University Medical Center LUMC,*
*Department of Cell and Chemical Biology- Cardiovascular Cell Biology, Leiden, Netherlands*
[3]*Universitat de Barcelona, Department de Ciències Fisiològiques, Barcelona, Spain*

**Abstract Text**

**Background/Introduction**: Fibrodysplasia Ossificans Progressiva (FOP) is an ultra-rare disorder characterized by heterotopic ossification (HO) of connective soft tissues in response to inflammatory trauma. The genetic basis of FOP is a germline gain-of-function mutation (c.617G>A; p.R206H) in the Bone Morphogenetic Protein (BMP) type I receptor ALK2, which is ubiquitously expressed. ALK2 mutants enable Activin cytokines to aberrantly activate the SMAD1/5/8 signaling pathway, thereby eliciting BMP-like cellular responses, including osteogenesis.
Despite the widespread expression of $ALK2^{R206H}$, FOP-related HO lesions arise preferentially in tissues subjected to high mechanical loading where dynamic interactions occur among mesenchymal stem cell (MSC) bone progenitors, capillary-forming endothelial cells (ECs), and infiltrating immune cells. We hypothesize that the biomechanical properties of the tissue microenvironment sensitize bone progenitor cells, fostering HO lesions in FOP.

**Purpose**: To develop effective and functional methods to investigate the role of biomechanical cues in FOP development.

**Methods**: Human MSC and EC lines stably overexpressing the wild type and mutant R206H forms of ALK2 were used to evaluate the impact of hypoxia (1% O2) using a Don Whitley H25 workstation. Exposure to shear stress (20 dyn/cm$^2$) was achieved through a microfluidic unidirectional flow pump system from Ibidi. ALK2 activation was evaluated through western blotting and qPCR analysis of ALK2 downstream targets. Results were confirmed using isogenic control and FOP ECs derived from induced pluripotent stem cells (iPSCs).

**Results**: MSC and EC lines stably overexpressing $ALK2^{R206H}$ resemble the Activin aberrant response from primary patient-derived cells. Activin-induced ALK2 signaling in mutant ECs was modulated by high shear stress (20 dyn/cm$^2$), unlike in control ECs. Exposure of human MSCs expressing $ALK2^{R206H}$ to hypoxia (1%) resulted in a significant enhancement of ALK2 downstream signaling.

**Conclusion**: Results suggest that the in vitro study of FOP development may benefit of advanced cell culture models resembling the complex interactions between cell types and biomechanical cues in FOP lesions.

**P224**

**Pregnancy and lactation associated osteoporosis (PLAO): what happens in the subsequent pregnancy?**

*Marta Zampogna[1], Giorgia Grassi[2], Giorgia Casiraro[3], Alberto Ghielmetti[4], Anna Atlasova[1], Rondinella Sara[1], Giovanna Mantovani[1], Laura Trespidi[5], Cristina Eller Vainicher[2]*
*[1]Endocrine Unit- Fondazione IRCCS Ca' Granda Ospedale Maggiore Policlinico, Department of Clinical Sciences and Community Health – Dipartimento di Eccellenza 2023–2027- University of Milan, Milan, Italy*
*[2]Fondazione IRCCS Ca' Granda Ospedale Maggiore Policlinico, Endocrine Unit, Milan, Italy*
*[3]Department of Obstetrics and Gynecology- Fondazione IRCCS Ca' Granda Ospedale Maggiore Policlinico, Department of Clinical Sciences and Community Health – Dipartimento di Eccellenza 2023–2027- University of Milan, Milan, Italy*
*[4]University Hospital Santa Maria della Misericordia, Unit of Endocrinology and Metabolism, Udine, Italy*
*[5]Fondazione IRCCS Ca' Granda Ospedale Maggiore Policlinico, Department of Obstetrics and Gynecology, Milan, Italy*

**Abstract Text**

**Background:** Risk factors for pregnancy and lactation associated osteoporosis (PLAO) are not yet fully understood. In a previous national survey, we identified several pre-pregnancy and pregnancy-related factors (e.g., fragility fractures, low calcium intake, insufficient gestational weight gain, heparin use) that may be associated with a higher risk of PLAO and its recurrence.

**Methods:** Via the Italian association of women with PLAO (MAMog association), an anonymous questionnaire was administered to women diagnosed with PLAO who had at least one subsequent pregnancy, to assess the prevalence of recurrence and associated risk factors.

**Results:** Twenty-three responses were collected. Three patients (13%) reported new vertebral fractures (rePLAO): one case occurred immediately after delivery, and two within 3 months postpartum. Another patient experienced a rib fracture, although this could not be confidently attributed to PLAO. Compared with patients without recurrence, women with rePLAO more frequently reported a family history of fragility fractures (100% vs 25%; p=0.03) and all had used heparin during their second pregnancy (100% vs 57%; p=0.005). Moreover, 66.7% had not discontinued breastfeeding after the first episode, compared with 20.0% (p=0.08). One woman had a diagnosis of systemic mastocytosis after rePLAO. No significant differences were found between groups regarding vaginal delivery rates (33% rePLAO vs 15%), previous pharmacological treatment (66% rePLAO vs 75%), breastfeeding during the second pregnancy (33% rePLAO vs 35%), or insufficient weight gain during pregnancy (33% rePLAO vs 50%). All women took vitamin D during their second pregnancy; however, insufficient calcium intake remained highly prevalent (100% rePLAO vs 70%).

**Conclusions:** PLAO can recur in subsequent pregnancies; family history of fragility fractures and heparin use emerge as potential associated factors. The lack of awareness regarding breastfeeding and low calcium intake as major risk factors is concerning. Confirmation of these findings in a larger cohort and the development of clinical practice guidelines are needed.

**P226**

**The Erlenmeyer flask sign as a key element in the diagnosis of Pyle's disease: the importance of clinical experience**

*Sara Rondinella*[1,2]*, Maria Francesca Bedeschi*[3]*, Giorgia Grassi*[1]*, Alberto Ghielmetti*[4]*,*
*Marta Zampogna*[1]*, Anna Atlasova*[1,2]*, Alice Moroni*[5]*, Francesca Gurioli*[5]*, Luca Sangiorgi*[5]*,*
*Enrico Schileo*[6]*, Fulvia Taddei*[6]*, Marco Miceli*[7]*, Giovanna Mantovani*[1,2]*, Cristina Eller-Vainicher*[1]
[1]*Fondazione IRCCS Ca' Granda Ospedale Maggiore Policlinico, Endocrinology Unit, Milan, Italy*
[2]*University of Milan,*
*Department of Clinical and Community Sciences- Dipartimento di eccellenza 2023-2027, Milan, Italy*
[3]*Fondazione IRCCS Ca' Granda Ospedale Maggiore Policlinico, Medical Genetics Unit, Milan, Italy*
[4]*University Hospital Santa Maria della Misericordia, Unit of Endocrinology and Metabolism, Udine, Italy*
[5]*IRCCS Istituto Ortopedico Rizzoli, Unit of Rare Skeletal Diseases, Bologna, Italy*
[6]*IRCCS Istituto Ortopedico Rizzoli, Computational Bioengineering Laboratory, Bologna, Italy*
[7]*IRCCS Istituto Ortopedico Rizzoli, Diagnostic and interventional radiology, Bologna, Italy*

**Abstract Text**

**Background:** Pyle's disease is an extremely rare, autosomal recessive skeletal dysplasia caused by biallelic loss-of-function mutations in the SFRP4 gene that codes for secreted frizzled-related protein 4 (SFRP4), a Wnt pathway antagonist that plays a key role in bone remodeling. The loss of SFRP4 function disrupts the balance between cortical apposition and metaphyseal modeling, leading to metaphyseal trabecular expansion, cortical thinning, and skeletal fragility. The clinical presentation is characterized by genu valgum, dental anomalies, and the typical Erlenmeyer flask metaphyseal deformities.

**Clinical cases**
Case 1. A 30-year-old Turkish male initially diagnosed with osteogenesis imperfecta (OI). His history included vertebral and non-vertebral fractures treated for years with intravenous bisphosphonates without benefit. NGS analysis for skeletal fragility was negative. Exome sequencing revealed a homozygous mutation in exon 2 of SFRP4. Limb radiographs and orthopantomography documented the typical alterations consistent with Pyle's disease. Romosozumab therapy was initiated *off-label*, resulting in a significant increase in bone mass (LS +8.3%) over 12 months, without new clinical or morphometric fractures.

Case 2. A 64-year-old Italian woman with early-onset osteoporosis and fragility fractures, previously diagnosed as OI despite the absence of typical clinical signs. Retrospective re-evaluation of radiographs taken in her youth revealed Erlenmeyer flask metaphyseal deformities and arachnodactyly. The patient also presented with retained deciduous teeth. NGS analysis identified a likely pathogenic homozygous mutation in SFRP4. HR-pQCT documented thinned cortical bone and reduced tibial trabecular density. Romosozumab therapy was initiated in this case too.

**Conclusions:** Pyle's disease, though rare, is likely underdiagnosed due to limited clinical and radiological recognition. In the second case, retrospective evaluation of typical signs allowed for the correct diagnosis. The use of advanced techniques like HR-pQCT can improve bone characterization. Treatment with Romosozumab, a drug that enhances the Wnt pathway, may represent a potential new therapeutic option.

**P227**

**Adult patients with X-linked hypophosphatemia exhibit metabolic impairments beyond expected from body composition analysis – signals from a single-centre pilot cohort**

*Marko Stojanovic[1], Jelena Bajic[2], Bojana Radosevic-Caric[3], Valentina Soldat-Stankovic[4], Mirjana Doknic[2], Sandra Pekic[2], Zvezdana Jemuovic[2], Ivana Cekic[2], Ivan Jevtic[2], Boris Kurcubic[2], Marina Nikolic-Djurovic[2]*
*[1]Clinic for Endocrinology- Diabetes and Metabolic Diseases, Department of Neuroendocrinology, Belgrade, Serbia*
*[2]Clinic for Endocrinology- Diabetes and Metabolic Diseases- University Clinical Center of Serbia, Department of Neuroendocrinology, Belgrade, Serbia*
*[3]University Clinical Center of Republika Srpska- Banja Luka- Faculty of Medicine- University of Banja Lu ka-, Department of Endocrinology-, Banja Luka, Bosnia - Herzegovina*
*[4]University Clinical Center of Republika Srpska- Banja Luka- Faculty of Medicine- University of Banja Lu ka, Department of Endocrinology, Banja Luka, Bosnia - Herzegovina*

**Abstract Text**

**Background**: X-linked hypophosphatemia (XLH) is a rare genetic disease of autonomous FGF-23 over-secretion, hallmarked by hypophosphatemia and osteomalacia with progressive skeletal deformities. Obesity is frequently observed in XLH, attributed to impaired mobility, but possibly also linked to direct negative metabolic impact of FGF23. Other authors suggest that adult XLH patients are obese with excess fat mass, but without metabolic dysfunction.

**Methods**: Cross-sectional pilot study included a single-centre cohort of 11 adult XLH patients (9 female), 37.9±4.2years old, treated with active vitamin D and phosphate supplements. Anthropometric analysis included weight, height, BMI, and waist to hip circumference ratio (WHR). Body composition was analysed by DXA whole body scan - WBS (Hologic Discovery QDR). HOMA-IR was calculated from fasting blood glucose and fasting insulin. Matsuda index of insulin sensitivity (M-ISI) was calculated based on glucose and insulin (0min to 120min) during OGTT (75g).

**Results**: Average BMI (28.4 ±1.5kg/m$^2$) was on overweight to obesity borderline. Four patients were obese (36.4%) and 4 (36.4%) overweight. Average WHR (0.77±0.02) was below threshold for high metabolic risk. DXA-WBS revealed average total body fat mass (FM) of 21.2±1.9kg, average total lean-body mass (LMB) of 34.5±1.8kg, translating to Fat% 36.4±2.1%. According to age and gender reference centiles Fat% was above 75$^{th}$ centile in 7 (63.6%) subjects. Average HOMA-IR was 3.3±0.4 and M-ISI was 3.97±0.8, both indicating impaired insulin sensitivity. M-ISI did not correlate significantly with WBS-Fat%.

**Conclusion**: Adult XLH patients in our pilot single-centre cohort were overweight and obese with average waist to hip ratio not indicating high metabolic risk, but with average total body fat percent exceeding 75$^{th}$ age and gender centile in two-thirds. Average insulin resistance was higher than previously reported, possibly linked to factors beyond obesity or body composition. Comprehensive multidisciplinary follow-up of XLH patients is mandatory and further research should elucidate disease-specific metabolic risk factors.

**P229**

**Disease modeling of early-onset osteoarthritis in spondyloepiphyseal dysplasia using patient-derived human induced pluripotent stem cells**

*Maximilian Möhlmann[1], Assil-Ramin Alimy[1], Marina Reinsch[2], Arne Hansen[2], Ralf Oheim[3], Thorsten Schinke[3], Frank Timo Beil[1], Tim Rolvien[1]*
*[1]University Medical Center Hamburg-Eppendorf, Department of Trauma and Orthopaedic Surgery, Hamburg, Germany*
*[2]University Medical Center Hamburg-Eppendorf, Department of Experimental Pharmacology and Toxicology, Hamburg, Germany*
*[3]University Medical Center Hamburg-Eppendorf, Department of Osteology and Biomechanics, Hamburg, Germany*

**Abstract Text**

**Introduction:** Spondyloepiphyseal dysplasia congenita (SEDC) is an inherited skeletal disorder caused by pathogenic variants in the *COL2A1* gene. SEDC is characterized by low bone mass and early progressive joint damage, which leads to early-onset osteoarthritis (EOOA). Despite the significant impairment of the patients' quality of life and mobility, the underlying molecular mechanisms of EOOA remain poorly understood, and apart from total joint replacement, there is no effective treatment.

**Purpose:** The aim of this study was to decipher the protein signature in the synovial fluid of a SEDC patient, investigate the osteochondral hip phenotype and establish an *in vitro* system in which endochondral ossification can be simulated.

**Methods:** Synovial fluid samples from OA control patients (n=5) and one SEDC patient (n=2) were examined for targeted proteome analysis. To assess the osteochondral phenotype in SEDC, we used multiscale histology-based imaging. Fibroblasts from a skin biopsy were isolated and reprogrammed into human induced pluripotent stem cells (hiPSC). Isogenic controls were generated using CRISPR/Cas9.

**Results:** Compared to OA, the synovial fluid of the SEDC patient showed an overall unaltered to lower inflammatory state including downregulation of IL-1β ($p=0.048$) (Fig.1 A). The subchondral bone analysis in *COL2A1*-related SEDC revealed a lower calcium content ($p=0.029$) and decreased hardness ($p<0.001$) (Fig.1 B). A patient-derived *COL2A1*-variant hiPSC line and its isogenic control were generated, and chondrogenic and osteogenic differentiation protocols were established (Fig.1 C). Osteogenically differentiated patient-derived cells showed reduced mineralization compared with healthy controls, as indicated by lower Alizarin Red absorbance.

**Conclusion:** Our data suggests that inflammation is not a driver of EOOA in *COL2A1*-related SEDC. Biopsy data indicated structurally compromised bone with reduced resistance to deformation. The establishment of the dual hiPSC/isogenic control model indicates impaired mineralization as an explanation for the co-occurrence of EOOA and low bone mass and provides a robust foundation for future mechanistic studies of *COL2A1*-related SEDC.

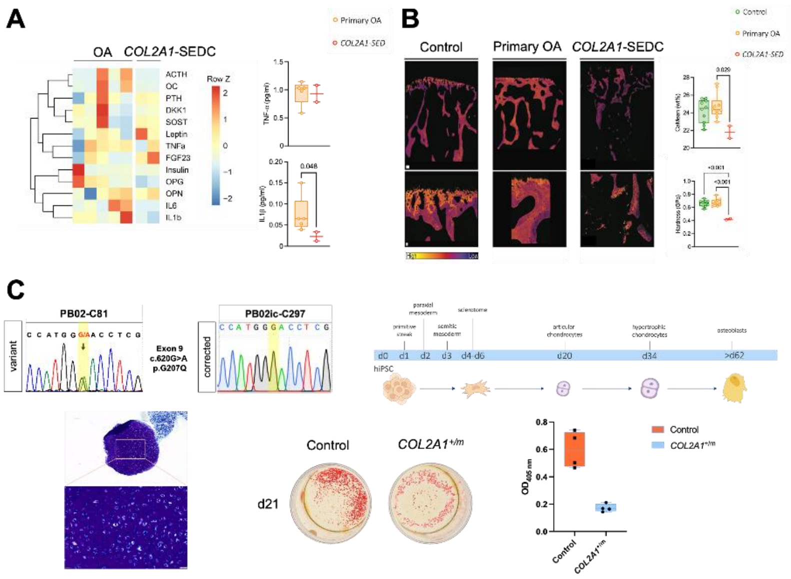

**Fig. 1.** A structural and mineralization impairment in the osteochondral unit of a patient with SEDC, along with *in vitro* hypomineralization in patient-derived cells. **A** Proteome analysis of the synovial fluid of a patient with SEDC and primary OA patients. **B** Subchondral bone calcium content (wt%) and hardness (GPa) were quantified in a patient with SEDC, patients with primary OA and controls. **C** Generation of *COL2A1*-variant and isogenic control hiPSC lines, establishment of chondrogenic and osteogenic differentiation protocols, and quantification of Alizarin Red staining in osteogenically differentiated cells.

### P230

### Investigating the role of misfolded collagen I in skeletal and cardiorespiratory aging

*Wendy Pérez Franco*[1], *Roberta Besio*[1], *Antonella Forlino*[1], *Marta Filibian*[2], *Roberta Gioia*[2]
[1]*University of Pavia, Department of Molecular Medicine, Pavia, Italy*
[2]*University of Pavia, Centro Grandi Strumenti, Pavia, Italy*

**Abstract Text**

Mutations in collagen I genes are the most common cause of osteogenesis imperfecta (OI), a hereditary pathology characterized by bone fragility. In adulthood, patients with OI also exhibit premature aging–related frailty, including impairments in cardiovascular and respiratory function, although the underlying molecular mechanisms remain poorly understood. To this aim we investigated by micro-computed tomography and magnetic resonance imaging long bone, lung and heart of male and female 6-, 12- and 18- months old (m) Brittle (Brtl/+) mice, a well characterized model of dominant OI. The presence of abnormal collagen I caused detrimental effects over time in male bone parameters at the tibia mid-diaphysis with a 10.11 % reduction in cortical thickness ($p < 0.05$) and 9.14 % decrease in cortical area fraction ($p < 0.01$) in old mutant mice (18 m) with respect to mutant adults (6 m). On the contrary, age did not compromise cortical bone parameters in Brtl/+ females. Ex vivo high-resolution scanning confirmed that cortical bone in Brtl/+ is hypermineralized at 12 m and more porous with age compared to wild-types (WT) bone ($p < 0.01$). Compared with age-matched WT mice, male Brtl/+ mice at 12 m showed impaired systolic function, evidenced by reduced ejection fraction and cardiac output ($p < 0.05$). Diastolic function also appeared compromised, with a decreased peak filling rate ($p < 0.05$). Gene expression analysis showed an increased mRNA expression of the senescence marker Cdkn1a in bone ($p < 0.05$), while Trp53 mRNA was increased in all tissues ($p < 0.05$), suggesting a differential activation of cellular pathways in a tissue-specific manner in the Brtl/+ mice. Our findings support an alteration of the properties of bone and heart's extracellular matrix, revealing a functional association between misfolded collagen I and progression of aging-related impairment.
Funding Horizon Europe MSCA-DN programme CHANGE No 101072766.

**P231**

**Pharmacological inhibition of CDK8 kinase by KY-065 rescues abnormal chondrogenesis in an achondroplasia mouse model**

*<u>Koki Sadamori</u>[1], Kubo Takuya[1], Megumi Yamamoto[2], Tatsuya Kitao[2], Hiroaki Shirahase[2], Eiichi Hinoi[1]*
*[1]Gifu Pharmaceutical University, Department of Bioactive Molecules- Pharmacology, Gifu, Japan*
*[2]Kyoto Pharmaceutical Industries- Ltd., Drug Discovery Research Department, Kyoto, Japan*

**Abstract Text**

**Background:** Achondroplasia (ACH) is a rare skeletal disease characterized by abnormal cartilage formation. It is widely known that ACH is caused by a gain-of-function mutation in fibroblast growth factor receptor 3 (FGFR3), a receptor tyrosine kinase, leading to overactivation of its downstream mitogen-activated protein kinase (MAPK) and signal transducer and activator of transcription (STAT) signaling pathways. Vosoritide has been approved for ACH treatment as the first precision drug in children, while novel therapeutic options with different mechanisms are still required. Cyclin-dependent kinase 8 (CDK8) belongs to the transcription-related CDK family member and plays an important role in bone homeostasis. However, the functional role and underlying mechanisms of CDK8 on progression of ACH remain largely unknown.

**Purpose:** The present study investigated the potential efficacy of CDK8 inhibitor KY-065 in reversing the ACH phenotypes *in vitro* and *in vivo*.

**Method:** We used *Fgfr3*$^{Ach}$ transgenic mice (hereafter referred to as ACH mice), which express mouse *Fgfr3* harboring a G380R mutation under the *Col2a1* promoter. Primary chondrocytes were prepared from costal cartilages of 3 day-old wild-type (WT) and ACH mice and subsequent western blotting and cell staining. WT and ACH mice were administered KY-065 daily from 3 to 30 day-old, followed by µCT and histological analyses.

**Results:** CDK8 expression and the phosphorylation of STAT1$^{ser727}$ were elevated in ACH mouse chondrocytes. KY-065 reversed the suppression of chondrogenesis induced by the *Fgfr3* mutation. KY-065 selectively decreased the phosphorylation of STAT1$^{Ser727}$without affecting MAPK activation. KY-065 increased the femur length, the thickness of growth plate, and hypertrophic chondrocyte area in ACH mice.

**Conclusion:** The present study suggests that CDK8 in chondrocytes may be a viable therapeutic target and that KY-065 has the potential to serve as a promising drug for the treatment of ACH.

**P232**

**Circulating extracellular vesicles in Tumor-Induced Osteomalacia: analysis in a patient before and after radical excision of a sino-nasal PMT.**

*<u>Luciano Colangelo</u>[1], Laura Di Giuseppe[2], Michela Rossi[2], Davide Diacinti[3], Alessandro Corsi[4], Salvatore Minisola[1], Andrea Del Fattore[2]*
*[1]Sapienza University of Rome, Department of Medical and Cardiovascular Sciences, Rome, Italy*
*[2]Bambino Gesù Children's Hospital- IRCCS, Bone Physiopathology Research Unit-*
*Translational Pediatrics e Clinical Genetics Research Division, Rome, Italy*
*[3]Sapienza University of Rome, Department of Radiological Sciences- Oncology and Pathology, Rome, Italy*
*[4]Sapienza University of Rome, Department of Molecular Medicine, Rome, Italy*

**Abstract Text**

**Background**: Extracellular vesicles (EVs) play a pivotal role in cell-to-cell communication and may act as mediators of pathological conditions. Even though their role in bone homeostasis is well established, their impact on bone disease remains to be clarified.

**Purpose**: This study aimed to characterize the circulating EVs in a patient with Tumor-Induced Osteomalacia (TIO) caused by a sino-nasal Phosphaturic Mesenchymal Tumor (PMT) before (T0) and 2 months (T1) and 2 years (T2) after surgery. Clinical, biochemical, and densitometric assessments were also performed at T0, T1 and T2.

**Results**: Nanosight analysis revealed an increase in circulating extracellular vesicles in the patient before surgery compared with sex- and age-matched healthy donors (HDs). Patient-derived EVs were also smaller than HD-derived EVs. Interestingly, FACS analysis of isolated EVs showed a higher percentage of EVs positive for the exosomal marker CD81 and for bone-remodelling markers, including CD115 (M-CSF), CD265 (RANK), and RANKL. After surgery, we observed a time-dependent reduction in circulating EVs, that reached levels similar to those of HD at T2. Moreover, we detected an increase in EV size at T1, and this change persisted at T2. FACS analysis also showed a reduction in the percentage of CD81/CD115/CD265/RANKL-positive EVs in post-surgery samples, reaching levels comparable to those observed in HDs. After surgery, the patient also showed progressive improvement in BMD, increase of serum phosphate, decrease of alkaline phosphatase in the absence of changes in serum calcium, which collectively support the normalization of bone mineralization.
**Conclusion**: This study suggests that the striking increase in BMD does not reflect only a passive process driven by the enhanced mineralization following the normalization of the serum levels FGF23 but also an active biological mechanism through the restoration of the proper balance of bone formation and resorption that EVs derived from a TIO-causing PMT impair.

**P233**

**Induced pluripotent stem cells validate the hypothesis of IL1B up-regulation in cystinosis osteoblasts and open new therapeutic perspectives**

*<u>Claire Dumortier</u>[1], Candide Alioli[1], Chloé Groyeux[1], Corinne Antignac[2], Irma Machuca-Gayet[1], Justine Bacchetta[1]*
*[1]INSERM, Unité U1033 LYOS, Lyon, France*
*[2]Inserm UMR 1163, Institut Imagine, Paris, France*

**Abstract Text**

Cystinosis is a rare autosomal recessive lysosomal storage disease caused by mutations in cystinosin (CTNS), a cystine transporter, leading to hypophosphatemic rickets, chronic kidney disease, growth retardation and skeletal abnormalities. We previously showed intrinsic defects in bone cells with a higher number of osteoclasts generated from patients' PBMCs. In the Ctns -/- murine model, osteoblasts displayed increased interleukin 1B, independently of RANKL/OPG, that may explain, at least partly, increased osteoclastogenesis. Last, patients' PBMCs have peculiar inflammatory profile with an over-expression of IL-1 receptor in differentiated osteoclasts, suggesting that cystinotic osteoclasts are more sensitive to IL1 signaling.

As cystinosis is a rare and severe disease, bone biopsies in patients cannot be performed. To validate murine results, we need human data, and especially new human cell models, such as induced pluripotent stem cells (IPSC) to have unlimited access to osteoblasts carrying different CTNS mutations.

IPSC have been generated from three cystinosis patients (whole gene KO or point mutations of CTNS). The three lines and a CRISPR-corrected one have been differentiated into MSCs. Osteoblast commitment was then compared between the lines. We also assessed IL18, the surrogate for Il1 pathway, in patients with cystinosis under conservative therapy.

In all 8 patients, IL18 concentrations were slightly increased, on average 1.9 times higher than the upper normal limit. Under MSC basal stage and osteoblast induction medium with addition of 10nM of 1.25VD3, the corrected line displayed less IL1B than the three others ($p<0.05$). The point mutation line presents a 10-100 fold increased IL1B expression compared to the other lines, suggesting the importance of the presence of the misfolded protein for IL1B expression.

The iPSC tool confirms murine results of IL1B pathway up-regulation in cystinotic osteoblasts, with a clinical relevance in patients displasying high Il18 levels: these results open potential therapeutic perspectives for cystinotic patients with severe bone impairment.

**P234**

**A heartbreaking case of hypocalcaemia**

*Lai-Ming Kathleen Pak*[1]*, Vivian Grill*[1]*, Hanh H. Nguyen*[1]
[1]*Western Health, Department of Endocrinology and Diabetes, Melbourne, Australia*

**Abstract Text**

**Introduction:** Hypocalcaemia-induced heart failure is a rare but reversible cause of cardiomyopathy. We report a case of a young man with an activating mutation of the calcium-sensing receptor (CaSR) who presented with cardiomyopathy in the setting of severe hypocalcaemia.

**Case summary:** A 35-year-old man with obesity presented with decompensated heart failure [left ventricular ejection fraction (LVEF) 31%]. Coronary angiography excluded an ischemic cause. Biochemistry demonstrated hypocalcaemia (corrected calcium 1.8mmol/L, ionized calcium 0.86mmol/L), hyperphosphataemia (2.25mmol/L), low 25-hydroxyvitamin D (19nmol/L), inappropriately-normal parathyroid hormone (PTH 6.8pmol/L), and normal renal function. Marked hypercalciuria (11.7mmol/day) and a family history of hypocalcaemia raised suspicion for autosomal dominant hypocalcaemia (ADH), which was confirmed on genetic testing. After correction of hypocalcemia, vitamin D supplementation, and initiation of heart failure therapy, his LVEF improved rapidly to 53%. Ongoing titration of calcium and calcitriol supplementation proved challenging due to persistent hyperphosphataemia, hypercalciuria, and the consequent risk of renal complications.

**Discussion:** Evaluation of non-iatrogenic hypoparathyroidism includes consideration of autoimmune, genetic, and infiltrative conditions, with family history often providing crucial diagnostic clues. ADH type 1 is caused by a gain-of-function mutation in the CaSR gene, increasing the receptor's sensitivity to calcium. PTH becomes suppressed at lower levels of extracellular calcium. Conventional therapy for hypoparathyroidism is often limited by hyperphosphataemia, hypercalciuria, and a high pill burden. Ultimately, it does not address the underlying pathophysiology of ADH. Novel agents—including the oral calcilytic Encaleret and PTH-based treatments—offer options that target the mechanisms driving this disorder.

**Conclusion:** This case highlights a rare genetic cause of hypocalcaemia and contributes to the limited literature on hypocalcaemic heart failure. It also illustrates the limitations of conventional therapy. Novel agents that target the underlying pathophysiology of ADH should improve the treatment of patients with this disorder.

**P235**

**In vitro micromass model for enchondral ossification using periodontal ligament cells from patients with Fibrodysplasia Ossificans Progressiva**

*Nathalie Bravenboer*[1], *Esmee van der Velden*[1], *Huib van Essen*[1], *Ivan Fernandez Cano*[1,2], *Joey Blonk*[1], *Jaika Rutgrink*[1], *Ton Schoenmaker*[2]
[1]*Amsterdam UMC, Laboratory Science, Amsterdam, Netherlands*
[2]*Academic Center Dentistry Amsterdam, Periodontology, Amsterdam, Netherlands*

**Abstract Text**

Fibrodysplasia Ossificans Progressiva (FOP) is a rare genetic disorder caused by mutations in the ACVR1 gene, leading to progressive heterotopic ossification through endochondral pathways. To establish a noninvasive in vitro model, we utilized periodontal ligament (PDL) cells, multipotent stem cells obtainable from extracted teeth, and cultured them as high-density micromasses to mimic endochondral ossification. PDL cells from two FOP patients and two healthy controls were expanded and seeded at 40,000 cells per agarose-filled well in 96-well plates, then maintained for up to 4 weeks under proliferative, chondrogenic, osteogenic, or subsequent chondrogenic and osteogenic differentiation conditions. Weekly Alcian blue and Alizarin red staining revealed stable micromass formation, with progressive increases in chondrogenic and osteogenic markers (Figure 1). FOP-derived micromasses showed elevated expression of ColX and RUNX2 compared to controls, while Activin A treatment did not alter differentiation outcomes.

**Conclusion:** High-density micromasses of PDL cells provide a robust model to recapitulate endochondral ossification in vitro. This system offers a valuable platform to investigate mechanisms underlying heterotopic ossification in FOP and to explore potential therapeutic interventions.

**Figure 1:**Chondro-osteogenic differentiation of high-density micromasses, cultured in chondrogenic medium for the first two weeks and on osteogenic medium for the last two weeks. Weekly time points show increased staining intensity for alcian blue (A-D) and alizarin red (E-H) staining.

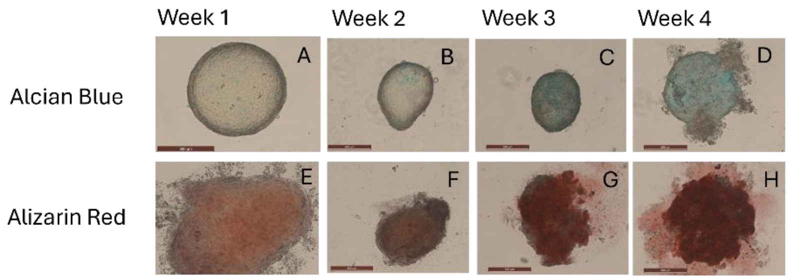

**P236**

### Specific effects on cortical bone in PLS3 associated early onset osteoporosis: findings in humans and mice, including response to treatment

*Oskar Windels[1], Alexander Simon[1], Julian Delsmann[2], Uwe Kornak[3], Michael Amling[1], Thorsten Schinke[1], Timur Alexander Yorgan[1], Ralf Oheim[1]*
*[1]University Medical Center Hamburg Eppendorf, Department of Osteology and Biomechanics, Hamburg, Germany*
*[2]University Medical Center Hamburg Eppendorf, Department of Trauma and Orthopaedic Surgery- Division of Orthopaedics, Hamburg, Germany*
*[3]University Medical Center Göttingen, Institute for Human Genetics, Hamburg, Germany*

**Abstract Text**

The importance of the actin-bundling protein plastin-3 for skeletal integrity has been demonstrated by the identification of pathogenic *PLS3* variants in individuals with X-linked osteoporosis (XLO). Prior studies, including our own, have shown that *Pls3* deficiency in mice results in reduced cortical thickness.

In order to better characterize the impact of *PLS3* inactivation in humans, we retrospectively compared the skeletal parameters of male XLO patients with those of males with a genetically confirmed diagnosis of osteogenesis imperfecta type I (OI-I).

No significant differences were observed between XLO and OI-I patients regarding fracture prevalence, laboratory parameters, or bone mineral density measurements obtained using dual-energy X-ray absorptiometry (DXA). However, high-resolution peripheral quantitative computed tomography (HR-pQCT) revealed a remarkable difference between the two groups. More specifically, XLO patients showed significantly lower cortical bone parameters than both the reference range and OI-I, particularly at the distal radius. We also analyzed female heterozygous carriers of (likely) pathogenic *PLS3* alleles. These females were less affected than male XLO patients. However, it is notable that two female carriers experienced osteoporotic fractures during the postmenopausal period. Hence, treatment with the sclerostin antibody romosozumab was initiated and resulted in a favorable response. As another sclerostin-neutralizing antibody is currently undergoing clinical trials to evaluate its effectiveness in treating OI, we crossed *Pls3*-deficient mice with those carrying a high bone mass allele of *Lrp5* ($Lrp5^{A231V}$). This allele serves as a molecular mimic of anti-sclerostin therapy. We found that the presence of the $Lrp5^{A231V}$ allele resulted in a significant increase in cortical and trabecular bone mass not only in wildtype but also, importantly, in *Pls3*-deficient mice.

In summary, these results demonstrate that inactivation of *PLS3* leads to a specific skeletal phenotype in the cortical bone and suggest that anti-sclerostin therapy could be an effective treatment for XLO.

**P237**

**McCune-Albright syndrome in the young woman: challenges in diagnosis and treatment**

*Nataliia Grygorieva*[1], *Daryna Kurylo*[1]
[1]*D.F. Chebotarev Institute of Gerontology of the National Academy of Medical Sciences of Ukraine, Department of Clinical Physiology and pathology of the musculoskeletal system, Kyiv, Ukraine*

**Abstract Text**

**Background:** McCune-Albright syndrome (MAS) is a rare genetic disorder caused by activating mutations in the GNAS gene at R201 position, with an estimated prevalence of 1/100,000 to 1/1,000,000. It is classically characterized by the triad of fibrous bone dysplasia (FD), precocious puberty (PP), and café-au-lait skin pigmentation (CSP). Typically, FD is the most prominent component, and it may present in mono- or polyostotic forms.

The **aim** was to describe the case of a young woman with MAS and to analyze the clinical challenges in her diagnosis and treatment, based on current literature knowledge.

**Case Summary:** A 21-year-old female visited our center with complaints of knee pain aggravated by physical activity. She was referred for bone mineral density (BMD) assessment after more than seven years of bisphosphonate therapy. She had also been receiving an active vitamin D analogue for long-standing hypophosphatemia.

MAS was first suspected at 3 months of age due to PP and CSP, and later confirmed radiographically (FD). She received hormone therapy until menarche. During childhood, she developed progressive scoliosis requiring surgical correction with metal osteosynthesis. She also experienced several low-trauma rib fractures.

Endocrine laboratory tests (FSH, LH, progesterone, estradiol, androgens, cortisol, prolactin) were within normal limits. Parameters of calcium-phosphate metabolism (calcium, phosphorus, PTH, ALP), as well as 25(OH)D and $1,25(OH)_2D$ concentrations, were normal. Despite bisphosphonate treatment, the bone turnover marker P1NP remained elevated, and scintigraphy showed increased uptake at FD sites. The patient looks for recommendations regarding the safety of planned pregnancy, the appropriate duration of ongoing treatments, and potential updates to her long-term management plan.

**Conclusion:** A review of the literature revealed significant gaps in the management of MAS features, particularly bone and joint symptoms, and uncertainties regarding conservative treatment strategies for FD. Establishing registries for rare bone diseases could improve clinician collaboration and enhance collective expertise in managing MAS.

**P238**

**A comparison between postmenopausal women with classic OI and women with severe osteoporosis receiving romosozumab**

Mikolaj Bartosik[1], Oskar Windels[1], Felix N. Von Brackel[1], Michael Amling[1], Ralf Oheim[1]
[1]University Medical Center Hamburg-Eppendorf, Department of Osteology and Biomechanics, Hamburg, Germany

**Abstract Text**

**Background:** Osteogenesis imperfecta (OI) is the most common hereditary bone disease, characterized by increased bone fragility and impaired bone quality. But pharmacological treatment is still limited. Ongoing clinical trials investigating monoclonal anti-sclerostin antibodies for OI patients, offer new hope for reducing bone fragility by improving bone quality.

**Methods**: Postmenopausal women with either Osteogenesis imperfecta (n = 5) or severe osteoporosis (OPO, n = 10) receiving monthly Romosozumab treatment (210 mg s.c.) for 12 months were analyzed retrospectively. Clinical assessments were performed at baseline, after 6 months, and after 12 months. Areal bone mineral density (DXA) and bone microstructure (HR-pQCT) were evaluated at baseline and after 12 months of treatment. In addition, serum and urine markers of bone turnover were analyzed at each time point.

**Results:** The mean age of patients was 53.6 ± 8.9 years for OI patients and 57.2 ± 5.8 years for OPO patients (p = 0.444). After 12 months of Romosozumab treatment, spinal aBMD and bone formation markers (osteocalcin, P1NP) increased significantly in OI patients, indicating an anabolic response, while resorption markers remained unchanged. HR-pQCT analysis revealed no statistically significant microstructural changes in patients with OI, although trends with moderate to strong effect sizes suggested potential improvements in trabecular and cortical parameters, particularly at the tibia. In patients with OPO, we observed a more pronounced response of bone turnover markers with greater aBMD gains at both the spine and femur, as well as significant improvements in bone microstructure.

**Conclusions:** Romosozumab treatment in adults with OI resulted in a promising increase in spinal aBMD, though the effect on hip aBMD and peripheral bone microstructure was limited in contrast to postmenopausal osteoporosis.

### P239

### Incidental Fractures in Patients with Osteogenesis Imperfecta in Adulthood: A Descriptive Study of a Cohort

*Cristina Rocamora Gisbert[1], Francina Salabert-Carreras[1], Raquel Ugena-Garcia[1], Cristina Calomarde-Gomez[1], Clara Churtichaga[1], Judit Vidal-Ripoll[1], Agueda Prior-Español[1], Laia Gifre[1]*
[1]Hospital Germans Trias i Pujol, Rheumatology, Badalona, Spain

**Abstract Text**

**Background:** Osteogenesis Imperfecta (OI) is a condition caused by collagen mutations. It is associated with fractures, bone deformities, osteoporosis, and systemic manifestations.

**Purpose:** To describe the characteristics of adults with OI, including their phenotype, fracture, densitometric data and the treatments received.

**Methods:** Descriptive observational study involving OI patients at a tertiary hospital. Data collected were clinical (age at diagnosis, dentinogenesis imperfecta, hearing loss, skeletal deformities, and fracture history), genetics, densitometric data (Lunar DPX), radiology results, and antiosteoporotic treatment (antiOP). In patients under 50 years, a Z score < -2 SD was used for OP diagnosis. Trabecular structure was assessed using TBS (TBS Insight®). Statistical analyses were conducted using SPSS.

**Results:** 13 patients (9 women and 4 men) with a mean age of 54±19 [28-84] years diagnosed with OI were included. 6 patients were the index case for OI. Patient characteristics are summarized in *Table1.* The mean age at diagnosis was 20±19.57 [0-63] years. Mutations were in COL1A1 (n=8) and COL1A2 (n=2), and not available in 3 patients. By densitometry, 9 patients met criteria for osteoporosis and only 4 patients had a degraded TBS.

11 patients had ever received antiOP, including oral and intravenous bisphosphonates (n=9; n=10), denosumab (n=4), and teriparatide (n=4). Nine patients remained on antiOP at their last follow-up visit.

The 13 patients had a total of 167 fractures, with 25 being traumatic, with a mean of 12.84 [1-27] fractures per patient. 7 patients had vertebral fractures. Over the last two years, 7 patients sustained 15 incidental fractures (10 fragility and 5 traumatic) while treated with bisphosphonates (n=4), denosumab (n =2), and without treatment (n =1).

**Conclusion:** More than half of the OI patients, even with active antiOP, experienced incidental fractures in the last two years. TBS does not seem to effectively predict the risk of fractures in OI patients.

**P240**

**Denosumab as a therapeutic option in McCune-Albright Syndrome and Fibrous Dysplasia: a case report**

*Daniel Queremel Milani[1], Myriam Partida Muñoz[1], Guillermo Martinez Diaz-Guerra[1]*
*[1]Hospital Universitario 12 de Octubre, Endocrinología y Nutrición, Madrid, Spain*

**Abstract Text**

**Introduction**: McCune-Albright syndrome (MAS) is a rare genetic disorder with challenging diagnostic and therapeutic approaches. We present a clinical case that illustrates the long-term management complexity.

**Description**: A 51-year-old woman diagnosed with MAS, characterized by polyostotic fibrous dysplasia (PFD) with extensive involvement of the axial and appendicular skeleton (skull, costal arches, vertebral bodies, sternum, scapula, femur, tibia, right metatarsals, and metatarsophalangeal joints), resumed medical care three years prior after being lost to follow-up. Her medical history also includes precocious puberty, cutaneous hyperpigmentation ("café-au-lait" spots), a right forearm fracture after a fall, and a prophylactic intramedullary nailing of the right femur. At presentation, she reported chronic generalized bone pain (VAS 7/10), predominantly the right lower limb and costal region, with substantial impairment of quality of life. Imaging studies revealed well-defined expansive lytic lesions in multiple posterior and lateral costal arches, a 50% compression fracture of the L3 vertebral body, and a markedly reduced femoral bone mineral density (BMD) (t-score -2.5SD). Following three doses of zoledronic acid administered biannually with minimal improvement, therapeutic change to denosumab 60 mg provided mild femoral pain relieve and considerable reduction in serum biomarkers (see Table 1).

**Conclusion**: Denosumab may represent a safe and potentially effective alternative in PFD refractory to bisphosphonate therapy.

**Table 1**. Serum biomarker progression

| | **Baseline** | **5 mo after 1st ZA** | **Before 2nd ZA** | **1 mo after 2nd ZA** | **Before 3rd ZA** | **5 mo after 3rd ZA** | **1 mo after denosumab** | **3 mo after denosumab** |
|---|---|---|---|---|---|---|---|---|
| Alkaline phosphatase* | - | 14.1 | 26.7 | 26.7 | 20.7 | 16.7 | 11.0 | 12.3 |
| Beta-crosslaps* | 0.45 | 0.20 | 0.29 | 0.08 | 0.21 | 0.19 | 0.04 | 0.10 |
| Osteocalcin* | - | 27.9 | 37.6 | 27.7 | 26.2 | 21.2 | 15.3 | 14.1 |
| Parathyroidhormone# | 47.2 | 36.9 | 57.3 | 69.1 | 31.4 | 26.4 | 112.0 | 87.9 |
| Vitamin D* | 10.9 | 33.4 | 38.7 | 51.4 | 48.4 | 40.8 | 37.9 | 34.1 |

* Units in ng/ml; # Units in pg/ml.
Abbreviations: mo: month; ZA: Zoledronic acid

**P241**

### The spectrum of bone disease in distal renal tubular acidosis

*Pankaj Singhania*[1], *Ramamoorthy Ponnusamy*[2], *Gouranga Santra*[2]
[1]*Arogyam Health Care, Endocrinology and metabolism, Purulia, India*
[2]*Deben Mahatha government Medical College, Medicine, Purulia, India*

**Abstract Text**

**Introduction:** Renal tubular acidosis is associated with a wide range of metabolic bone disorders. While rickets and osteomalacia are common presentations in children and adults, sclerotic bone disease is a rare manifestation of renal tubular acidosis.

**Case presentation:**

**Case 1**

A 13 yrs old child presented with recurrent proximal muscle weakness, protuberant abdomen, widened wrists and ankle joints. She had severe hypokalemia (1.5 mmol/L) with normal anion gap metabolic acidosis and urine pH 6.0 suggestive of distal renal tubular acidosis. She had hypercalciuria medullary nephrocalcinosis. Patient was initiated on oral alkali and potassium replacement. Dramatic improvement in myopathy, bone pain, height was noted over 6 months. Hypokalemia and metabolic acidosis improved and X-ray of wrist joints showed provisional zone of calcification.

**Case 2**

A 42 years gentleman presented with severe pain in hip and quadriparesis. There was severe hypokalemia (2.5 mmol/ L), elevated bone alkaline phosphatase (216 IU/L, N- 26-117), hypophosphatemia(1.36 mg/dl) and normal anion gap metabolic acidosis. Urine pH was 6.5. X ray of hip and spine showed hyperdense bones which was further confirmed as increased BMD on DXA scan. Patient was started on oral alkali and potassium replacement therapy showing rapid improvement in musculoskeletal symptoms.

**Discussion:** People presenting with recurrent hypokalemia with musculoskeletal symptoms should be evaluated for renal tubular acidosis. Renal tubular acidosis can present with rickets and stunted growth in children and osteomalacia, proximal muscle weakness, nephrocalcinosis in adults and very rarely sclerotic bones. Widely available and inexpensive treatment with oral alkali and potassium can provide long term relief, improvement in bone pain and prevent renal deterioration

**Conclusion:** Renal tubular acidosis, especially distal type can present with wide spectrum of metabolic bone disorders and with hypokalemia and metabolic acidosis. Meticulous history, structured investigations will clinch the diagnosis and treatment responses are excellent and prevent long term musculoskeletal deformity, height loss and preserve renal function.

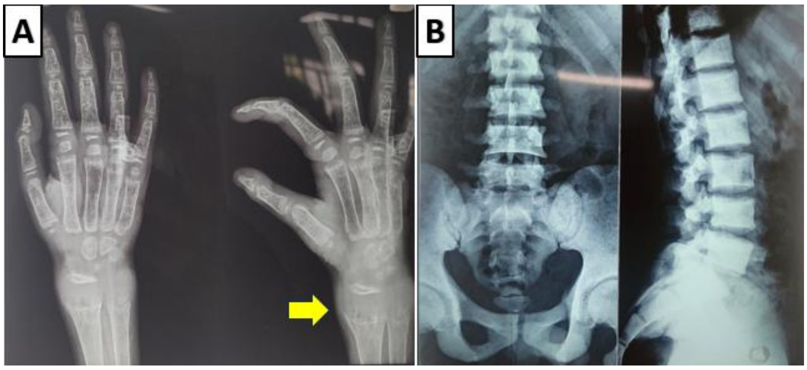

**ND-P028**

### Beyond Plaques: A RARE CASE OF CALCINOSIS CUTIS IN A PATIENT WITH PSORIASIS VULGARIS

_Sreya Yarlagadda_[1], *Rajeswari Sankaralingam*[2], *Balaji Chilukuri*[2]
[1]*SRI RAMA CHANDRA INSTITUTE OF HIGHER EDUCATION AND RESEARCH, CLINICAL IMMUNOLOGY AND RHEUMATOLOGY, CHENNAI, India*
[2]*SRIHER, Clinical Immunology and Rheumatology, Chennai, India*

### Abstract Text

**BEYOND PLAQUES: A RARE CASE OF CALCINOSIS CUTIS IN A PATIENT WITH PSORIASIS VULGARIS**

A lady in her early fifties previously diagnosed to have palmoplantar psoriasis vulgaris about five years prior to visit, presented to the outpatient department with the history of swelling over tips of fingers of both hands since two years. Initially painless, the swelling present over fingertips became algetic over the past one year. The patient also complained of pruritus over the lesions.

Patient had no history of muscle weakness, cardiorespiratory complaints, photosensitivity, Raynaud's phenomenon, oral ulcers, skin thickening or sclerodactyly. Patient also did not report worsening of psoriatic plaques or skin lesions over palms and soles.

On cutaneous examination, she was noted to have- firm to hard, well-defined papular and nodular lesions. The lesions were chalky white and were non-compressible and non-fluctuant on palpation, with some lesions showing surface irregularity. The patient also had exfoliative plaques of plantar psoriasis over both her feet. A consideration of calcinosis cutis was made. Her systemic and vitals examination were otherwise normal.

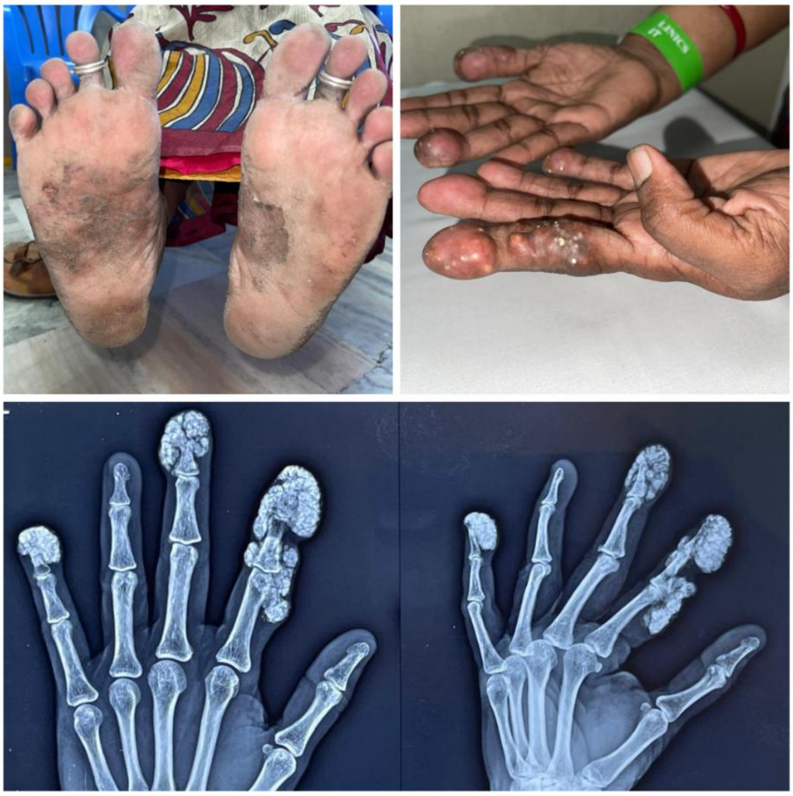

Roentgenograms of both her hands were taken which revealed radio-opaque, prominent and calcified bulbous lesions over fingertips.

A suspicion of autoimmune rheumatological etiology was ruled out, with a negative antinuclear antibody profile. A positron emission with computed tomography scan was undertaken to rule out malignancy which was normal. All hematological, renal and urinary examinations that were done were also normal.

Patient was diagnosed to have dystrophic calcification and calcinosis cutis by means of radiological correlation in the computed tomography scanning.

Patient was treated with diltiazem, calcium, vitamin D supplementation and bisphosphonates. Patient improved symptomatically with the above treatment, with no complaints of pain, no worsening of the lesions and continues to be on follow up with the department.

**ND-P029**

**Generation and characterization of a P4hb-Y395C mouse model of Cole–Carpenter syndrome and therapeutic proof-of-concept**

*Elisa Pucci*[1]*, Luciana Silvestri*[1]*, Mattia Capulli*[1]*, Nadia Rucci*[1]*, Antonio Maurizi*[1]
[1]*University of L'Aquila, Department of Biotechnological and Applied Clinical Sciences, L'Aquila, Italy*

**Abstract Text**

The Cole Carpenter Syndrome (CCS) is a neglected autosomal dominant disease affecting bone, which becomes brittle. CCS is caused by the heterozygous p.Y393C mutation in the *P4HB* gene encoding for the protein disulfide isomerase A1 (PDIA1). To date, there are no studies showing how the mutation affects the skeletal and the disease has no cure. Based on this, we generated a mouse model carrying the mouse homolog *P4hb* Y395C mutation. Skeletal phenotyping revealed pronounced osteopenia and increased bone fragility in mutant mice across multiple ages, associated with an impaired bone quality ($p<0.05$). Consistently, CCS mice exhibited reduced serum procollagen type I N-terminal propeptide (PINP) levels (-60%; $p<0.01$), indicating decreased type I collagen biosynthesis. Primary osteoblasts isolated from CCS mice showed lower expression of osteoblast-associated markers (*Col1α1* -26%, p=0.003; *Ocn* -37%, p=0.04), whereas osteoclast differentiation was increased (+2.5fold, p=0.005). At the subcellular level, Western blot and immunofluorescence analyses conducted on cellular models carrying the $P4HB^{Y393C}$ revealed a reduction in type I collagen expression (-50%;p=0.009) and secretion (-70%;p=0.01) along with a higher PDIA1 cytosolic stabilization and Bip1 and p62 expression (+3 fold and +1.8fold, respectively; $p\leq0.05$) compared to WT cells suggesting the presence of ER stress and an impaired autophagic flux. Similar results were obtained in primary CCS mouse osteoblasts. Alteration in the type I collagen path and cellular trafficking was further confirmed by unbiased proteomic analysis performed in CCS femurs ($p<0.05$). Finally, we explored mutation-specific therapeutic strategies. Specifically, we developed an allele-specific siRNA approach identifying sequences capable to selectively silencing the mutant P4HB allele in vitro (-50%, $p<0.05$) and ex vivo in CCS femurs (-53%, $p=0.007$) without affecting the WT allele. Collectively, our study establishes a robust preclinical model for P4HB-related CCS, defines defective collagen I biosynthesis as a central pathogenic mechanism, and identifies mutation-specific therapeutic strategies.

**ND-P030**

**Clinical Characterization of X-Linked Hypophosphatemia and Acquired Renal Phosphate Wasting in Adults: Preliminary Results from a Registry-Based Analysis**

*<u>Andrea Rebeca Hernandez Hernandez</u>[1], M. Carola Zillikens[1], Ariadne Bosman[1], Natasha M Appelman-Dijkstra[2], Annemieke M. Boot[3], Martin de Borst[4], Annenienke van de Ven[5], Renate T. de Jongh[6], Arend Bökenkamp[7], J.P. Joop van den Bergh[8], B.C.J. Bram van der Eerden[1], Evert F.S. van Velsen[1]*
*[1]Erasmus University Medical Center, Department of Internal Medicine- Erasmus MC Bone Center, Rotterdam, Netherlands*
*[2]Leiden University Medical Center, Department of Internal Medicine- Center for Bone Quality Leiden, Leiden, Netherlands*
*[3]University Medical Center Groningen- University of Groningen, Department of Pediatrics, Groningen, Netherlands*
*[4]University Medical Center Groningen, Department of Internal Medicine, Groningen, Netherlands*
*[5]Radboud University Medical Center, Department of Internal Medicine, Nijmegen, Netherlands*
*[6]Amsterdam University Medical Center- Vrije Universiteit, Department of Internal Medicine, Amsterdam, Netherlands*
*[7]Amsterdam University Medical Center- Emma Children's Hospital, Department of Pediatric Nephrology, Amsterdam, Netherlands*
*[8]VieCuri Medical Center, Department of Internal Medicine, Venlo, Netherlands*

**Abstract Text**

**Background:** Chronic hypophosphatemia with renal phosphate wasting can be due to rare genetic forms, most often X-linked hypophosphatemia (XLH) or can be acquired, most often through tumor-induced osteomalacia (TIO) or hyperparathyroidism/vitamin D deficiency Not seldomly no underlying cause is identified and patients are classified as unexplained hypophosphatemia, making etiological differentiation challenging, and difficult clinical decision-making.

**Purpose:** To compare clinical manifestations in adults with chronic renal phosphate–wasting hypophosphatemia due to genetic (XLH) and (suspected) acquired causes to determine whether these manifestations can inform initial evaluation.

**Methods:** We analyzed baseline data from the ORPHOS-NED Registry, an ongoing observational study of chronic hypophosphatemia. Cases were classified as XLH when confirmatory diagnosis was available (genetic testing and/or family history, and clinical diagnosis). TIO and unexplained cases were grouped as (suspected) acquired hypophosphatemia Clinical manifestations were compared using Fisher's exact and Chi-square tests

**Results:** 92 adult patients were analyzed, 58 females (63%, median age 46 years [IQR 32-57.5]). Groups consisted of 53 XLH and 39 acquired patients (14 TIO, 25 unexplained). XLH showed higher prevalence of skeletal deformities (64.2% vs 7.7%; p<0.05), dental problems (22.6% vs 7.7%), history of rickets (32.1% vs 5.1%; p<0.05). The acquired group showed more muscle weakness (59% vs13.2%; p<0.05), fatigue (51.3%vs17% p<0.05), and bone/joint pain (56.4% vs 28.3%; p<0.05) and stiffness (30.8%vs11.3%; p<0.05). Fractures were more prevalent in the acquired group (48.7% vs 32.1%), with vertebral predominance (16.5%) versus femoral in XLH (28.3%).

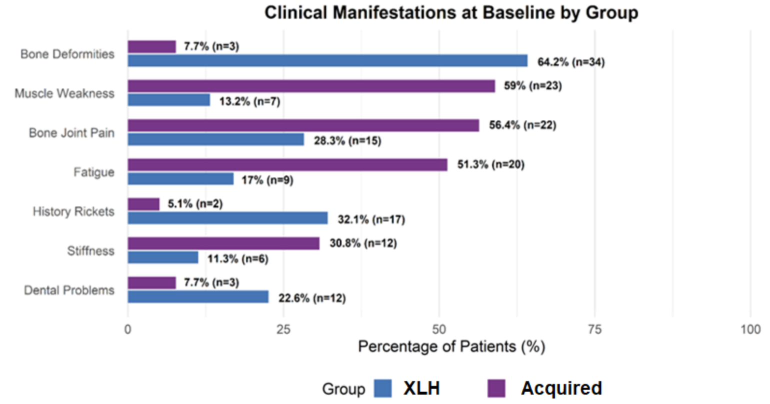

**Conclusions:** This preliminary analysis shows that clinical characteristics like a history of rickets, dental problems, and bone deformities are more frequent in XLH while acquired forms present more often with muscle weakness, pain, fractures and fatigue. While XLH features are well-described, their characterization remains relevant given overlap with acquired causes. Recognition of these patterns may help distinguish between genetic and acquired forms of hypophosphatemia.

**ND-P031**

**Ollier Disease and Maffucci Syndrome in Adults: A Retrospective Cohort Study**

*Astrid Dauchez[1], Frédéric Sailhan[2], Mevyn Nizard[3], Justine Luciano[1], Frédérique Larousserie[4], David Biau[2], Pascaline Boudou-Rouquette[5], Camille Tlemsani[5], Sophie Monnot[3], Alexandra Villard[2], Philippe Anract[2], Valérie Cormier-Daire[3], Eugénie Koumakis[1]*
*[1]Cochin Hospital, Rheumatology, Paris, France*
*[2]Cochin Hospital, Orthopedic, Paris, France*
*[3]Necker-Enfants Malades Hospital, Genomic Medicine for Rare Diseases, Paris, France*
*[4]Cochin Hospital, Pathology, Paris, France*
*[5]Cochin Hospital, Medical Oncology, Paris, France*

**Abstract Text**

**Background/Introduction:** Ollier disease (OD) is a rare skeletal disorder (prevalence ~1/100,000) caused by somatic *IDH1*/*IDH2* mutations, characterized by childhood-onset, typically asymmetrical enchondromas affecting mainly the metaphysis of long bones, hands, and feet. Maffucci syndrome (MS) is a related disorder combining multiple enchondromas with soft tissue hemangiomas. Both conditions are associated with an increased risk of malignancy, including chondrosarcoma and non-skeletal cancers.

**Purpose:** To describe the characteristics of adult patients with OD/MS, with and without chondrosarcoma.

**Methods:** We retrospectively studied adult patients with clinically and radiologically confirmed OD/MS at our referral center. Demographic, enchondroma-related, malignant transformation, imaging, laboratory, and genetic data were collected. Patients with and without malignant transformation were compared.

**Results:** Fifty-eight patients were included (34 women, 24 men): 48 with OD and 10 with MS. Mean age was 39 years, mean age at diagnosis was 18±19 years, mean diagnostic delay 4±9 years. Diagnosis was prompted by clinical skeletal abnormalities (48%), fracture (20%), pain (28%), or was incidental (4%). When tested, *IDH1* mutations were detected in 70% of patients, mostly in tissue samples (bone or other).

Eighteen patients (31%) had a history of chondrosarcoma, including five with ≥2 lesions; mean age at diagnosis was 35 years. Tumors involved lower limb long bones (36%), trunk (27%), fingers (14%), skull (14%), and upper limb long bones (9%). Patients with chondrosarcoma were older (46 vs 35 years, p = 0.01), but did not differ in terms of disease subtype, enchondroma distribution or complication. Six patients had other malignancies and nine had other benign tumors. Four patients had or were receiving anti-IDH therapy.

**Conclusion:** In this adult OD/MS cohort, chondrosarcoma occurred in one-third of patients, most often in long bones. Older age was associated with malignancy, while other clinical features did not differ, highlighting the need for long-term monitoring for malignant transformation.

**ND-P032**

**Pain Patterns in adults with Fibrous Dysplasia.**

*Melanie Legrand*[1,2], *Franz Clemeno*[1], *Kassim Javaid*[1]
[1]*University of Oxford, Botnar institute for Musculoskeletal Sciences- NDORMS, Oxford, United Kingdom*
[2]*Geneva University Hospitals, Division of Bone Diseases, Geneva, Switzerland*

**Abstract Text**

**Introduction:** Pain is a common and disabling symptom in adults with fibrous dysplasia (FD), although its temporal patterns, triggering factors, and underlying pain mechanisms remain poorly understood. In particular, the contribution of nociplastic pain has not been investigated in this population.

**Purpose:** This study aimed to characterize pain patterns in adults with FD and to explore the presence of nociplastic pain using the UK RUDY registry.

**Methods:** A disease-specific FD-pain questionnaire was developed in collaboration with FDSSUK patient association to assess pain duration, flare-ups, and flare triggers. In parallel, the 2016 ACR

fibromyalgia diagnostic criteria were applied to evaluate nociplastic pain. Both questionnaires were implemented in the online UK RUDY registry for adult FD patients in July 2025 and data were extracted in January 2026. Descriptive analyses were conducted using R.

**Results**: Among the 21 patients who completed the FD-specific pain questionnaire (71% women, median age 54 years), 81% reported chronic pain (>3 months) and 76% reported a course characterized by recurrent flares. The most frequently reported triggers were Improper or sudden movements (63%) and excessive physical activity (56%) and hormonal changes (e.g., menstruation - 42%). Fibromyalgia diagnostic criteria were met by 5 of the 19 patients (26%) who completed the questionnaire, all of whom were women, including 60% with monostotic FD.

**Conclusion:** Pain in FD is predominantly chronic and fluctuating, with identifiable triggers. A substantial proportion of adult FD patients exhibit nociplastic pain features, suggesting that central pain mechanisms may contribute to FD-related pain. These findings highlight the importance of multidimensional pain assessment and provide insight to guide pain management in FD, including pharmacological and rehabilitative strategies.

**ND-P033**

**Burosumab in Tumor Induced Osteomalacia: a local clinical survey and a Systematic Review with Individual Patient Data Analysis**

*Veronica Abate[1], Anita Vergatti[2], Aquilino Flavio Zarrella[2], Gianpaolo De Filippo[3], Ciro Menale[2], Lanfranco D'Elia[2], Daniela Merlotti[4], Luigi Gennari[4], Domenico Rendina[2]*
*[1]Federico II University, Department of Clinical Medicine and Surgery, Naples, Italy*
*[2]Federico II University, Department of Clinical Medicine and Surgery, Napoli, Italy*
*[3]Hôpital National d'Instruction des Armées Percy, Service de médecine interne et rhumatologie, Clamart, France*
*[4]Azienda Ospedaliera Universitaria Senese, Department of Medical Sciences, Siena, Italy*

**Abstract Text**

**Introduction:** Burosumab, a recombinant antibody against fibroblast growth factor 23 (FGF23), represent a new therapeutical option for Tumor induced osteomalacia (TIO). To estimate the clinical need and evaluate the efficacy and safety of Burosumab, we conducted a hybrid study composed by a clinical survey and a systematic review

**Methods:** Data were extracted on 12/31/2025. All patients referring for TIO to the Federico II University were enrolled in our clinical survey. A comprehensive literature search on a Medline, Google Scholar, Google Books, and the Cochrane Library was conducted for the Systematic Review. Data

**Results:** For the local survey, we collected 10 patients affected by TIO [M: F= 5 (50.0%): 5 (50.0%); mean age at symptom onset 57.6 ± 14.3 years]. Two (20.0%) patients received Burosumab, obtaining clinical and biochemical improvement. Two who didn't receive the treatment died. For the systematic review, we collected 42 studies and 49 cases [M: F= 19 (44.2%): 24 (55.8%); mean age at onset 36.8 ±

21.3 years; mean age at diagnosis 44.5 ± 22.9 years]. In all cases, Burosumab reduces clinical symptoms and ameliorates biochemical investigations by normalizing phosphate serum levels. It is also safe and well tolerated.

**Conclusion:** Burosumab represents a safe and efficient treatment in TIO patients, to be administered in cases of tumor identification while waiting for surgery, and in case of surgical inoperability to manage hormonal FGF23 effect.

**ND-P034**

**Proof-of-concept for a non-viral gene therapy for autosomal recessive osteopetrosis**

*Uwe Kornak[1], Dario Gajewski[1]*
*[1]University Medical Center Göttingen, Institute of Human Genetics, Göttingen, Germany*

**Abstract Text**

**Background:** Autosomal recessive osteopetrosis (ARO) is a rare hereditary bone disease caused by osteoclast (OC) dysfunction. ARO is characterized by abnormal accumulation of mineralized cartilage and bone leading to lethal bone marrow failure if untreated. The most common cause are mutations in *TCIRG1* and *CLCN7*, which encode crucial proteins for the acidification machinery of OCs. ARO can be treated by allogeneic hematopoietic stem cell (HSC) transplantation but relies on a suitable donor, rapid treatment and comes at risk for serious adverse events.

**Purpose:** Development and proof-of-concept of a gene therapy for *CLCN7*-dependent ARO

**Methods:** We developed a non-viral gene therapy using minicircle (MC) technology combined with Sleeping Beauty transposase (SB) for *CLCN7*-dependent ARO. SB integrates the expression cassette contained in the MC into the genome, which then drives a strong expression of *CLCN7*. The strategy was tested by transfection of patient-derived induced pluripotent stem cells (iPSCs). iPSCs were then differentiated into osteoclasts for functional analysis.

**Results:** Transfection of ARO-iPSCs led to 55% stable expressing cells in batch cultures and an average vector copy number of 0.87 vectors/cell. After differentiation we observed a 47-fold overexpression of *CLCN7* by qPCR. In contrast to resorption-deficient control cells (MC), transgene expressing cells (MC+SB) showed bone resorption comparable to wildtype iPSC-derived cells (Figure A). Next, we determined the necessary number of transgene expressing cells by diluting them with ARO-iPSC-derived cells. These mixed cultures all showed a resorption comparable to wildtype cells even with only 12.5% of transgenic cells (1 to 3, Figure B).

**Conclusion:** Here we provide proof-of-concept for a gene therapy for *CLCN7*-dependent ARO in our iPSC-model. We show that a full rescue is achieved with low percentages of transgenic cells. In the future, applying our approach to autologous HSCs may allow a quicker and safer treatment of ARO patients.

**ND-P035**

**IDH-mutant inhibition induce structural and clinical regression of enchondromas in Ollier disease and Maffucci syndrome: a first case series**

*Thomas Funck-Brentano*[1], *Mehdi Touat*[2], *Annouk Bisdorff Bresson*[3], *Valérie Bousson*[4], *Emmanuel Mandonnet*[5]
[1]*Universite Paris Cite, Rheumatology, Paris, France*
[2]*APHP Pitié Salpétriere, Neuro-oncology, Paris, France*
[3]*Lariboisière Hospital, Neuroradiology, Paris, France*
[4]*Universite Paris Cite Lariboisière Hospital, Radiology, Paris, France*
[5]*Universite Paris Cite Lariboisière Hospital, Neurosurgery, Paris, France*

**Abstract Text**

**Background and Objective:** Ollier disease and Maffucci syndrome are ultra-rare skeletal mosaic disorders caused by post-zygotic somatic mutations in *IDH1* or *IDH2*. They are characterized by multiple enchondromas, often associated with vascular malformations and a high lifetime risk of malignant transformation (e.g., glioma, chondrosarcoma). There is currently no targeted therapy for skeletal lesions, which can cause pain, deformities, and significant functional disability. We aimed to evaluate the clinical, biological and radiological impact of IDH inhibitor therapy on enchondromas in these patients.

**Methods:** We report five patients (3 Maffucci, 2 Ollier; all with *IDH1*-mutated low-grade gliomas) treated with ivosidenib (500 mg) or vorasidenib (40 mg) for 12–40 months. Clinical assessments included pain evaluation and physical examination. Imaging (MRI, X-ray) and biomarkers of bone formation (ALP, P1NP) were monitored longitudinally.

**Results:** All patients achieved glioma stability. Four of five reported marked improvement or complete resolution of chondroma-related pain. MRI showed regression of STIR signal and increased T1 fat signal in enchondromas in 3/3 evaluable cases. In 2 patients, X-rays revealed re-ossification of previously lytic enchondromas. Transient elevation of ALP was observed in 4 patients; P1NP increased at 12 months in one patient. No relevant adverse events were reported.

**Conclusion:** This first longitudinal case series provides clinical proof of concept that systemic IDH inhibition may induce structural remodeling and symptom relief in enchondromas associated with IDH-driven skeletal mosaicism. These results support further investigation of IDH inhibitors as a potential disease-modifying approach in Ollier and Maffucci syndromes.

**ND-P036**

**Delayed diagnosis of juvenile hypophosphatasia due to dental care neglect**

*Jerzy Konstantynowicz*[1], *Karolina Kierus*[2], *Pawel Abramowicz*[3], *Śliwko Pawel*[4], *Przemyslaw Wilinski*[5], *Tomasz Guszczyn*[6]
[1]*Medical University of Bialystok,*

*Department of Pediatrics- Rheumatology- Immunology- and Metabolic Bone Diseases, Bialystok, Poland*
[2]*University Children's Clinical Hospital,*
*Department of Pediatrics- Rheumatology- Immunology and Metabolic Bone Diseases, Bialystok, Poland*
[3]*Medical University of Bialystok,*
*Department of Pediatrics- Rheumatology- Immunology and Metabolic Bone Diseases, Bialystok, Poland*
[4]*University Children's Clinical Hospital, Department of Pediatric Radiology, Bialystok, Poland*
[5]*University Children's Clinical Hospital, Department of Pediatric Orthopedics and Traumatology, Bialystok, Poland*
[6]*Medical University of Bialystok, Department of Pediatric Orthopedics and Traumatology, Bialystok, Poland*

**Abstract Text**

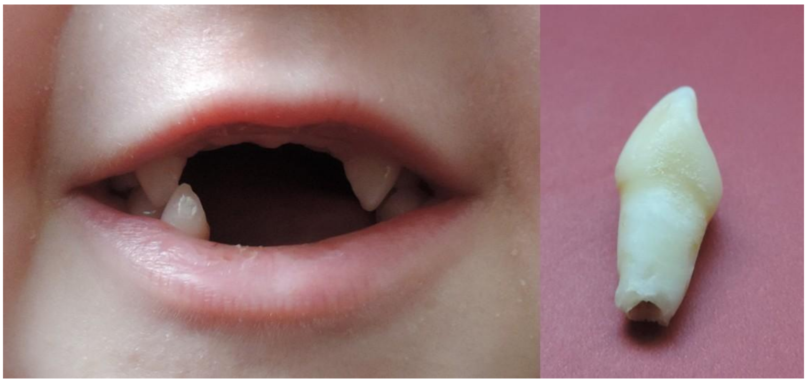

**Background**: Hypophosphatasia (HPP) is one of the underdiagnosed diseases with skeletal manifestations, especially since it can imitate other types of rickets and be masked by conditions presenting with fragility fractures and/or bone deformities.

**Case presentation**: A 3.5-year-old boy was referred due to clinical signs of rickets, premature loss of primary teeth, and severe chronic pain in the lower limbs. Psychomotor development was normal, teething was not disturbed, and his first tooth erupted at the age of 6 months. At the age of 1.5 years, he lost the first upper tooth with the roots intact; thereafter, from the age of 2, he lost all his incisors and canines (Fig.). He sustained one low-impact fracture of the tibia. He was receiving regular dental care, however, his dentition was treated only for caries. On admission, he presented the absence of incisors and lower canines, widened knee and wrist joints, knock-knees, a disturbed walking pattern, and fatigue. A persistently low-for-age concentration of tissue non-specific alkaline phosphatase (TNS-ALP) was found (minimum 51 IU/mL), whereas the patient was never screened before for ALP activity. An X-ray revealed delayed bone age, copper-beaten skull, radiolucency/deformity in metaphyses, and signs of severe rickets (Rickets Severity Score 3.5). Lumbar Spine bone mineral density using DXA was normal (Z-score = -1.4). Based on the comprehensive approach and differential studies, the boy was diagnosed with hypophosphatasia. The material was subjected to genetic testing for the *ALPL* gene mutation. Eligibility for bone-targeted treatment using human recombinant enzyme asfotase alfa was immediately decided.

**Conclusion**: Over two years of regular dental care appeared insufficient to detect a typical clinical picture of HPP. This case report underscores the need to raise awareness and knowledge of HPP within the medical community. This, in turn, may facilitate early implementation of effective etiological therapy for this rare multiorgan disease.

**Poster Sessions: Innovative concepts, new technologies, case reports**

**P242**

**RANKL cytokine is a novel supportive signal in the bone marrow hematopoietic niche**

*Laura Crisafulli[1], Maria Lucia Schiavone[2], Dario Strina[1], Federico Colombo[3], Gabriele De Simone[3], Chiara Camisaschi[3], Matteo Giovanni Della Porta[4], Cristina Sobacchi[1], Francesca Ficara[1]*
*[1]CNR, IRGB Milan Unit, Rozzano, Italy*
*[2]Humanitas Research Hospital IRCCS, Bone Metabolism Unit, Rozzano, Italy*
*[3]Humanitas Research Hospital IRCCS, Flow Cytometry Core, Rozzano, Italy*
*[4]Humanitas University, Department of Biomedical Sciences, Pieve Emanuele, Italy*

**Abstract Text**

**Introduction:** In the bone, RANKL is the essential pro-osteoclastogenic signal and has a pro-clonogenic and pro-osteogenic role in BMSCs[1], but whether it also affects the HSC compartment is not known.

**Purpose:** To assess whether RANKL is important for the BMSC support to the HSC niche. This has clinical relevance since RANKL is target of therapy in common disorders with excessive bone loss in patients with likely HSC dysfunctions. Due to the chronic nature of osteoporosis and the aging population, already prone to clonal hematopoiesis and BM microenvironment alterations, the consequences of prolonged RANKL inhibition deserve investigation.

**Methods:** Phenotypic (FACS analysis, gene expression) and functional (*in vitro* cultures, *in vivo* transplantation) characterization of HSCs and progenitors (HSPCs) from C57BL/6J *Rankl*$^{-/-}$ and WT mice (both sexes; experimental procedures as described[1,2]). Statistical analysis: Mann-Whitney test; n≥7.

**Results:** *Rankl*$^{-/-}$ mice displayed:

- Higher frequency of BM LSK cells ($p<0.01$ vs WT at 5 weeks of age), with a 1.5-fold relative expansion of the MPP1 myeloid-prone fraction at the expenses of HSCs ($p<0.05$);

- premature loss of HSC markers (**Fig.1a**);

- 1.5-fold lower proportion of CLP ($p<0.05$ vs WT at 5 weeks of age) and 3.6-fold lower CLP clonogenicity; accordingly, lower ability of MPP3 cells and HSCs to differentiate into CD19$^+$ B cells (**Fig.1b-c**);

- preserved multilineage reconstitution capacity of *Rankl*$^{-/-}$ donor cells in BM long-term reconstitution assays, in WT recipients of competitive BM transplants;

- no hematopoietic defects in the adult spleen and in the fetal liver;

- reduced expression of genes related to HSC support in SSCs (**Fig.1d**).

Finally, Denosumab-treated human CD34$^{+}$ cells preserved clonogenic ability and LTC-IC output, while Denosumab-treated BMSCs displayed a 3-fold reduction in the expression of the niche-supporting factor *ANGPT1* compared to untreated ($p<0.05$).

**Conclusion:** HSPCs in *Rankl*$^{-/-}$ mice have non-cell autonomous defects due to defective microenvironment. Also in humans RANKL expression is crucial to maintain the HSC-supporting ability of BMSC.

### References

1. PMID:38971808

2. PMID:23660001

**Figure 1**

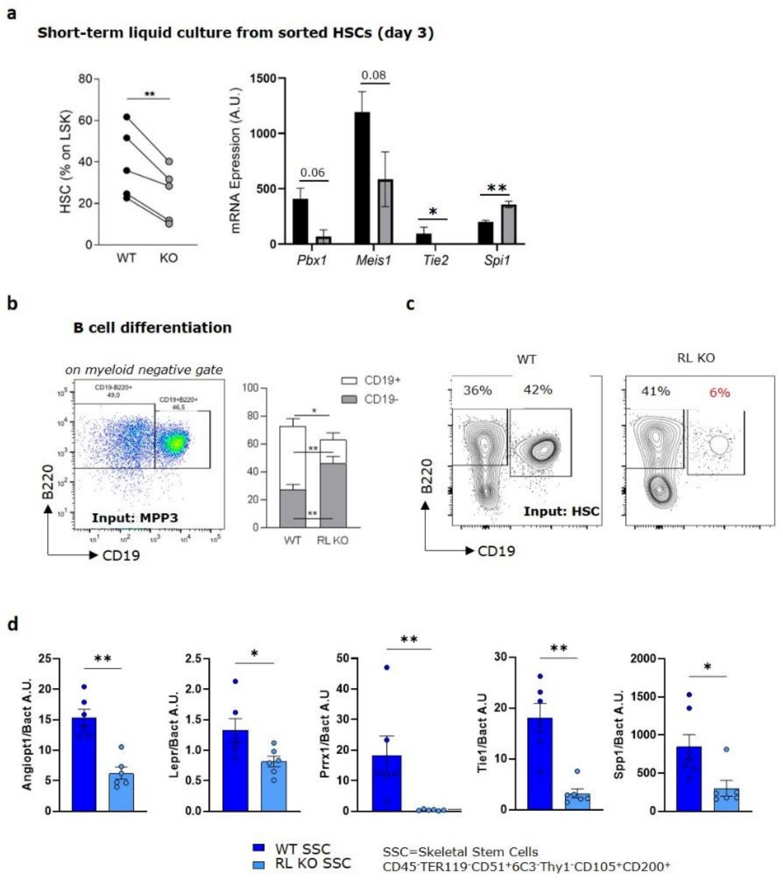

All data in the figure are presented as mean ± SEM; *$p$<0.05, **$p$<0.01

**P243**

### Accuracy and clinical utility of technology-derived assessments in older adults with cognitive impairment and sarcopenia: preliminary findings from the NRRP RAISE study.

*Ekaterini Zigoura[1], Emanuele Seminerio[1], Wanda Morganti[1], Giulia Bodo[2], Barbara Senesi[1], Carolina Massone[1], Giacomo Servidei[1], Alberto Pilotto[3], Romina Custureri[1], Matteo Laffranchi[2]*
*[1]E. O. Galliera Hospital, Geriatrics Unit, Genoa, Italy*
*[2]Italian Institute of Technology, Rehab Technologies Lab, Genoa, Italy*
*[3]E. O. Galliera Hospital, Scientific Coordination Unit, Genoa, Italy*

**Abstract Text**

**Background:** Technology-assisted assessment offers objective, continuous, and non-invasive monitoring of functional and clinical parameters in older adults. However, concordance between technology-derived and traditional measurements remains unclear, especially in neurocognitive disorders (NCD) or sarcopenia.

**Purpose:** To (i) validate the technological assessment by investigating its concordance with clinical evaluation, and (ii) identify differences in the multidimensional profile of older adults with NCD, sarcopenia, and healthy controls.

**Methods:** Thirty adults ≥65 years admitted to the intra-hospital RAISE Living Lab underwent a comprehensive geriatric assessment including Hand Grip (HG), Bioimpedance Analysis (BIA), sit-to-stand, and Timed Up and Go (TUG). Cognition (SPMSQ), nutrition (MNA), and frailty were assessed through the Multidimensional Prognostic Index (MPI). Technology-based monitoring was performed using Withings Body Cardio and BPM Core. Preliminary analyses included 10 controls, 8 NCD, and 7 sarcopenic participants.

**Results:** Clinical and technology-derived values for blood pressure, heart rate, muscle mass, fat mass, and total body water were aligned, no statistically significant difference was found. One-way ANOVA showed significant group differences in HG, Skeletal Muscle Index (SMI), BMI, sit-to-stand time, cognitive status, and frailty. Post-hoc comparisons revealed:

- **Control vs. NCD:** HG (46.3 vs 26.2, $p=0.009$), ADL (6 vs 5.5, $p=0.016$), IADL (8 vs 5, $p=0.004$), SPMSQ (0 vs 4, $p=0.001$), MPI (0.14 vs 0.28, $p=0.013$), sit-to-stand (11.6 vs 14.5, $p=0.023$), TUG (7.6 vs 11.6 s, $p=0.037$).
- **Control vs. Sarcopenia:** HG (49.2 vs 8.9, $p<.001$), BMI (26.5 vs 20.1, $p=0.010$), SMI (8.9 vs 5.8, $p<.001$), SPMSQ (0 vs 1, $p=0.044$), MNA (14 vs 10, $p=0.007$).

An inverse correlation was observed between TUG time and HG ($\rho=-0.505$, $p=0.010$).

**Conclusions:** Technology-derived assessments and clinical measures demonstrated good concordance. Significant differences emerged across groups, with both sarcopenic and cognitively impaired showing less muscle strength, associated with worse mobility, compared to the controls. These findings support the feasibility and usefulness of integrating sensor-based assessments into geriatric workflows.

**P244**

### Lineage topology and replication kinetics reveal regulated growth dynamics in human bone marrow stromal cell colonies

*Biagio Palmisano[1], Alessandro Allegrezza[2], Riccardo Beschi[2], Domenico Caudo[2], Alessandro Corsi[3], Antonio Culla[1], Samantha Donsante[4], Giuseppe Giannicola[5], Irene Giardina[2], Giorgio Gosti[6], Tomàs S. Grigera[7], Stefania Melillo[1], Leonardo Parisi[1], Lorena Postiglione[8], Mara Riminucci[3], Francesco Saverio Rotondi[2], Andrea Cavagna[1]*

[1]*National Research Council, Institute for Complex Systems, Rome, Italy*
[2]*Sapienza University of Rome, Physics, Rome, Italy*
[3]*Sapienza University of Rome, Molecular Medicine, Rome, Italy*
[4]*Fondazione IRCCS San Gerardo dei Tintori, Tettamanti Center, Monza, Italy*
[5]*Sapienza University of Rome, Anatomical- Histological- Medico Legal and Orthopaedic Sciences, Rome, Italy*
[6]*Italian Institute of Technology, Center for Life Nano & Neuro Science, Rome, Italy*
[7]*Universidad Nacional de La Plata, Instituto de Física de Líquidos y Sistemas Biológicos IFLYSIB, La Plata, Argentina*
[8]*University of Naples Federico II,*
*Department of Chemical- Materials and Industrial Production Engineering-, Naples, Italy*

**Abstract Text**

Bone marrow stromal cells (BMSC) – which include skeletal stem cells – are a promising tool in regenerative medicine. However, their *in vitro* heterogeneity and the unpredictable differentiation potential *in vivo*, remain critical barriers preventing the development of standardized therapeutic approaches for skeletal tissue regeneration. Several studies have attempted to identify *in vitro* features that could correlate with the *in vivo* differentiation properties, yet the fundamental mechanisms ruling BMSC heterogeneity remain poorly understood. Through the use of biophysical and statistical tools, we started investigating the basic *in vitro* growth dynamics of human clonogenic BMSCs. We conducted 2-week time-lapse imaging experiments of 32 single-cell-derived BMSC colonies (Fig.1a) and deconvoluted their lineage trees until the 7th generation of progeny cells (Fig.1b). By looking at the lineage topology determined solely by the number of senescent/apoptotic (N.inactive) cells, and replication kinetics (expressed as division time, $\tau$ and proliferation rate, $\gamma$) of each colony, we observed a significant inter-colony heterogeneity (Mean±SD: N.inactive=10.38±8.55; $\tau$=20.55±4.10; $\gamma$=0.19±0.12). Moreover, a significant intra-colony heterogeneity was also observed, in terms of division times, and occurrence of senescent/apoptotic events, localized at some branches of the lineage trees and not in others (Fig. 1b). Interestingly, lineage topology and replication kinetics of each colony resulted strongly correlated (Spearman correlation $\rho$=0.83, P-value<$10^{-6}$), suggesting the existence of regulatory factors linking the non-dividing/apoptotic subpopulations with proliferating cells. Interestingly, we found that the variability observed among the colonies from the same donor and passage was as large as the overall heterogeneity across all donors, age and passages. Indeed, except for a very weak correlation observed between $\tau$ and passage (Spearman correlation $\rho$=0.30, P-value= 0.046), no clearcut correlations among the other parameters were observed. Our findings reveal fundamental mechanisms governing BMSC growth dynamics that may inform strategies to control their heterogeneity and regenerative potential.

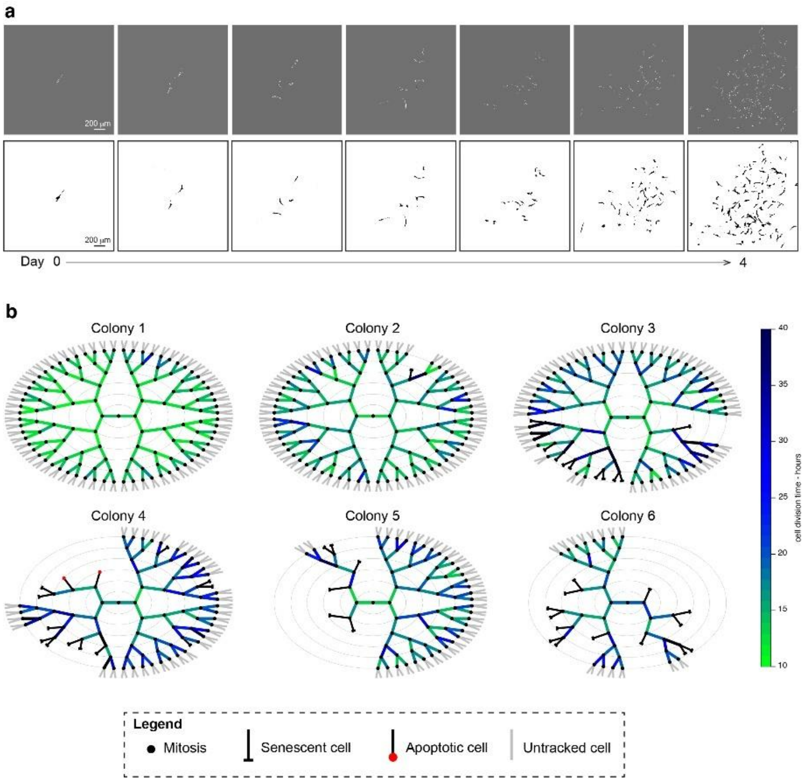

**P245**

**Primary trabecular bone architecture formed by the OmGFP66 osteogenic cell line: symmetry breaking across scales, from macro to nano**

*Marc Mckee[1], Benazir Khurshid[2], Aisha Mousa[3], Sarah L Dallas[4], Joseph Deering[5], Natalie Reznikov[2]*
*[1]McGill University, Anatomy and Cell Biology, Montreal, Canada*
*[2]McGill University, Bioengineering, Montreal, Canada*
*[3]McGill University, Faculty of Dental Medicine and Oral Health Sciences, Montreal, Canada*
*[4]University of Missouri, Oral and Craniofacial Sciences, Kansas City, United States*
*[5]McMaster University, Faculty of Dental Medicine and Oral Health Sciences, Montreal, Canada*

**Abstract Text**

**Introduction:** Osteocytes are abundant bone cells that serve as central regulators of skeletal homeostasis. Within mineralized bone tissue, osteocytes and their cell processes/dendrites maintain cell connectivity through a lacunocanalicular network morphologically positioned for executing myriad cell signaling pathways, including those related to mechanosensation, mineral ion homeostasis, and extracellular matrix mineralization. **Purpose**. Given the complexity of osteocyte morphological transitions within mineralized bone, there are few robust *in vitro* models that reproduce the mineralized bone microenvironment. A recently developed mouse calvarial osteocyte cell line, OmGFP66, overcomes many of these limitations. Here we provide a comprehensive 3D

multiscale characterization of mineralized, primary bone-like trabeculae formed by OmGFP66 cells *in vitro*, with a comparison to mouse calvariae from which they derived.

**Methods/Results:** Submicron X-ray microcomputed tomography (µCT) was used to image and reveal quantitative features of thousands of discrete, variably shaped and branching trabeculae formed by OmGFP66 cells with open and closed osteocyte lacunae having features quantitatively similar to those of neonatal mouse calvarial primary woven bone. Moreover, FIB-SEM and TEM electron microscopy analyses revealed the 3D ultrastructure of an extended lacunocanalicular network formed by the OmGFP66 cells within mineralized extracellular matrix and extending through an osteoid layer to osteoblasts at the surface, comparable to bone *in vivo*. At the nanoscale-to-microscale, again like bone, OmGFP66 trabeculae exhibit a 3D crossfibrillar mineral tessellation pattern. We also fit OmGFP66 trabecular morphology patterning and mineralization to the Gray-Scott model of oscillating reaction-diffusion patterns to describe symmetry breaking that initiates and facilitates mineralization through the combined dynamics of diffusing mineral ions and inhibitors.

**Conclusion:** These findings establish OmGFP66 cell cultures as a powerful *in vitro* bone model for studying osteocyte differentiation, lacunocanalicular network formation, matrix mineralization, and pattern formation, collectively allowing for early-pipeline genetic manipulations and assessments, and pre-screening of therapeutic compounds, prior to pre-clinical animal studies.

**P246**

**Engineering patient-derived organotypic bone models to understand osteoanabolic therapy in skeletal disease using fracture score and transcriptome analysis**

*<u>Gian Nutal Schädli</u>[1], Anke De Leeuw[1], Chris Steffi[1], Amit Singh[1], Pei Jin Lim[2], Marianne Rohrbach[2], Cecilia Giunta[2], Matthias Rüger[3], Ralph Müller[1]*
*[1]Institute for Biomechanics- ETH Zurich, Health Sciences and Technology, Zurich, Switzerland*
*[2]Connective Tissue Unit- University Children's Hospital Zurich,*
*Division of Metabolism and Children's Research Center, Zurich, Switzerland*
*[3]University Children's Hospital Zurich, Department of Pediatric Orthopaedics and Traumatology, Zurich, Switzerland*

**Abstract Text**

**Background:** Osteoanabolic therapies often fail in genetic skeletal disorders, highlighting gaps in mechanistic understanding and treatment evaluation. Patient-derived *in vitro* models offer opportunities to decode treatment response beyond empirical evaluation and toward mechanistically informed precision medicine.

**Purpose:** We engineered 3D-bioprinted organotypic bone models using primary cells from a patient with *FKBP10*-related osteogenesis imperfecta (OI) and from a metabolically healthy control obtained via femoral osteotomy (FO). These bone models, which can display a fracture phenotype, were used to evaluate mechanical integrity, extracellular matrix maturation, and transcriptional effects of Dickkopf-1 antibody (DKK1Ab) treatment.

**Methods:** Primary osteoblasts from surgical waste material were isolated and used to generate 3D-bioprinted constructs cultured under cyclic mechanical loading for eight weeks. Treatment with 1 µg/mL DKK1Ab or IgG-control began after four weeks. Time-lapsed micro-CT quantified mineralization and fracture score. Collagen deposition and RNA sequencing assessed secretome and transcriptome responses.

**Results:** OI constructs showed elevated interferon-stimulated gene (ISG) expression, increased early mineral formation, and hypermineralization accompanied by progressive loss of structural fidelity. Fracture scores increased to a median of 30.0% (maximum 79.2%) under IgG-control conditions, whereas DKK1Ab reduced deterioration, lowering fracture scores to median 6.7%, approaching FO values (2.2–2.7%). Mineral density remained significantly higher in OI than FO ($p<0.05$). RNA-sequencing revealed 2,522 differentially expressed genes(DEGs) between untreated OI and FO models ($|log2FC|>1$, FDR<0.01). DKK1Ab treatment induced 746 DEGs in FO, including classical Wnt-associated osteogenic markers. In contrast, OI constructs exhibited only 20 DEGs, of which 19 ISGs were downregulated and MKI67 was upregulated, indicating interferon-associated repression and increased proliferative activity in response to DKK1Ab.

**Conclusion(s):** Unlike in FO, treatment in OI resulted in a limited transcriptional response marked by ISG downregulation. Nevertheless, these treated samples exhibited improved matrix secretion, enhanced construct stability, and reduced fracture scores in *FKBP10*-related OI. These findings demonstrate that patient-derived organotypic bone models can reveal multiscale treatment responses.

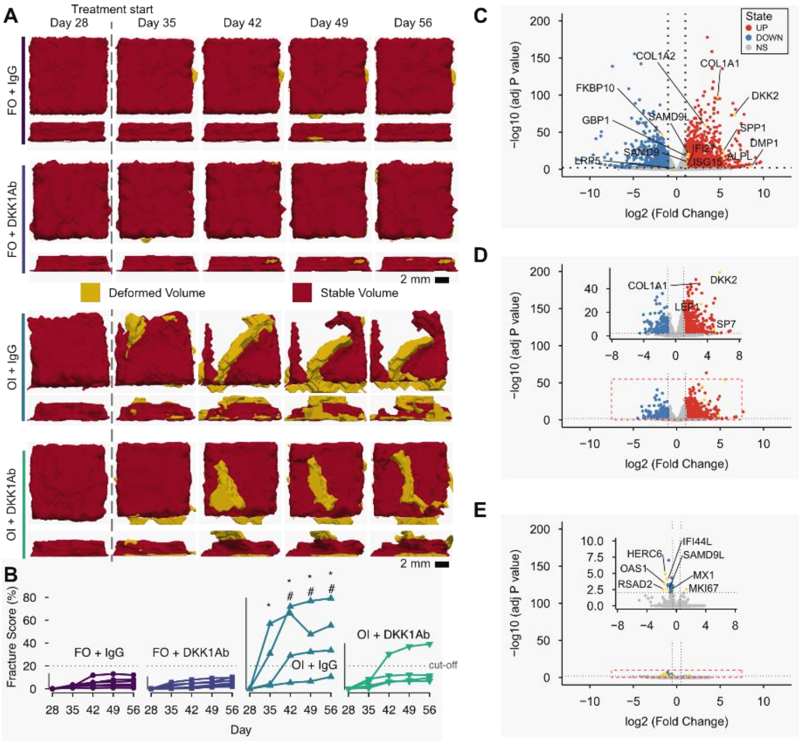

Figure 1: Fracture score and transcriptome analysis in FO and OI organotypic bone models treated with IgG or DKK1Ab. (A) Registered time-lapsed micro-CT images from day 28-56 for the four experimental conditions showing Deformed Volume (yellow), Stable Volume (red) relative to the day 28 reference. (B) Quantitative fracture score analysis for the four experimental conditions. FO + DKK1Ab and FO + IgG, n = 6 samples; OI + IgG and OI + DKK1Ab, n = 4 samples. Data represent individual samples. Statistical significance was assessed using nparLD with Benjamini-Hochberg post-hoc tests. Letters indicate significant differences ($p < 0.05$) as follows: *, different from FO + IgG; #, different from FO + DKK1Ab. C) Volcano plot showing differentially expressed genes between OI+IgG and FO+IgG control models. (D) Volcano plot of differentially expressed genes of FO models following DKK1Ab treatment (FO+DKK1Ab) compared to OI+IgGand (E) of OI+DKK1Ab compared to OI+IgG. FDR-adjusted P value < 0.01.

**P248**

**A case of progestogen-induced bone loss: importance of the knowledge of contraceptive therapies and their side effects for patients and all healthcare providers**

*Ewout Verly*[1]
[1]*VITAZ, Endocrinology, Sint-Niklaas, Belgium*

**Abstract Text**

**Clinical case:** a 47 year old female patient with auto-immune hypothyroidism presented to the emergency department with thoracic pain after an episode of heavy coughing. Chest X-ray revealed a rib fracture but no pneumonia. An incidental finding of an (asymptomatic) impaction fracture of vertebra 8 was noted in the radiological protocol.
The case was accidentally picked up by the treating endocrinologist, after which a bone densitometry was performed.
DXA revealed a lowest T-score of -2,8 at lumbar spine with a Z-score of -3,5. VFA confirmed a fracture of Th8 (43% - 9mm) with a suspected grade 1 fracture of Th9. An elaborate work-up with screening of secondary factors revealed active smoking, SSRI use (Sertraline 50mg daily since 1,5 years) and use of an intramuscular progestogen (contraceptive, Depo-Provera 150mg every 12 weeks for over 10 years). Biochemistry showed suppressed estradiol levels but no other secondary causes of osteoporosis. After stopping the IM progestogen, estradiol levels returned to normal without any arguments for menopause.Calcium- and vitamin D supplements as well as Alendronate 70mg once weekly were started as additional treatment.

**Discussion:** we discuss a case of iatrogenic, progestogen-induced bone loss in a premenopausal woman which led to (multiple) fragility fractures.
The main learning points is that this problem occur more frequent than expected, and that it is often diagnosed 'by accident' (or even underestimated).
Everyone starting on high dose IM progestogens should be counselled. Healthcare providers should be more aware of this potentially severe side effect. Follow-up of estrogen levels or bone densitometry should be considered on long-standing progestogen therapy as the problem could be reversed if stopped or switched.
The impact of frequently used contraceptive strategies in young women could be clinically significant and might have devastating effects: it might put patients at risk for osteoporosis and fragility fractures, necessitating treatment.
An overview of current literature is provided.

**P249**

**A bispecific small molecule drives armed antibodies to the bone mineral matrix**

*Alberto Dal Corso*[1], *Cassandra Spinosa*[1], *Mattia Mason*[1], *Cristina Visentin*[2], *Stefano Ricagno*[2], *Beatrice Rocutto*[3], *Marco Gobbi*[3], *Massimo Zucchetti*[4], *Roberta Frapolli*[4], *Claudia Nastasi*[4]

[1]Università degli Studi di Milano, Department of Chemistry, Milan, Italy
[2]Università degli Studi di Milano, Department of Biosciences, Milan, Italy
[3]Istituto di Ricerche Farmacologiche Mario Negri IRCCS,
Department of Molecular Biochemistry and Pharmacology, Milan, Italy
[4]Istituto di Ricerche Farmacologiche Mario Negri IRCCS, Department of Experimental Oncology, Milan, Italy

**Abstract Text**

**Introduction:** Mineral targets like bone hydroxyapatite (HA) remain inaccessible to therapeutics exploiting monoclonal antibodies (mAbs), limiting the use of advanced targeting therapies.

**Aim:** We designed and synthetized an innovative pre-targeting strategy in which a small molecule (SM), made of alendronate and an artificial antigen FITC, drives mAbs to the HA of the bones.

**Methods and Results:** The SM ALN-FITC was obtained in one step, showing high aqueous solubility, binding stably synthetic HA. The anti-FITC mAb was formatted as a fragment antigen-binding (Fab) with an accessible C-terminal disulfide for conjugation to cysteine-reactive payloads; initial studies used Cy5-maleimide.

*In vitro* confocal microscopy showed ALN-FITC binding to synthetic HA and Fab-Cy5 recognition of the FITC moiety. Surface plasmon resonance (SPR), direct and competitive assays, confirmed high Fab-Cy5 affinity for FITC (KD 622 pM).

To study the SM biodistribution *in vivo*, healthy balb/c mice (authorization no. 9F5F5.198, Italian Ministry of Health) were intravenously injected with saline, FITC, or ALN-FITC; organs were collected at 6, 24, and 72h and analyzed by IVIS imaging. ALN-FITC selectively accumulated to bones, excluding other organs, generating strong and persisting signals up to 72h.

The Fab was subsequently conjugated to monomethyl auristatin E (MMAE), a microtubule disrupting drug. SDS-PAGE and size-exclusion chromatography (SEC) were used to characterize it. Functional *in vitro* assays evaluated the activity of the antibody–drug conjugate (ADC) containing MMAE on the 4T1.2 bone-tropic cancer cell line. After 72h, cells were stained and analyzed by flow cytometry, showing that only ALN-FITC-treated HA enabled ADC accumulation and induced apoptosis in neighboring cells.

**Conclusion:** We developed a flexible platform in which an SM enables mAb recruitment and payload delivery. The payload of the mAb can be independently optimized, allowing delivery of diverse therapeutics, including cytotoxic agents to primary or secondary bone tumors, and may extend to additional bone diseases after further studies.

**P250**

**Micro-multiphysics agent-based modeling of trabecular bone remodeling after estrogen depletion - towards animal-free in silico preclinical trials**

*Friederike Schulte[1], Sorour Khadivi[1], Jack J. Kendall[1], Gisela A Kuhn[1], Ralph Müller[1]*

[1]*Institute for Biomechanics, ETH Zurich, Zurich, Switzerland*

**Abstract Text**

**Introduction:** Estrogen depletion during menopause disrupts bone remodeling and accelerates bone loss, driven by molecular-level changes within bone cells. Although *in vivo* micro-computed tomography in preclinical animal models can identify formation and resorption sites, it provides no access to the molecular mechanisms driving these spatial patterns. Micro-multiphysics agent-based (micro-MPA) models can simulate these cellular and molecular interactions but they require rigorous validation against experimental data. Closing this gap is key to building reliable animal-free *in silico* preclinical trials for osteoporosis therapy development.

**Purpose:** To validate the ability of a micro-MPA model of simulating remodeling patterns after estrogen depletion in an *in silico* model of osteoporosis.

**Methods:** We combined *in vivo* micro-computed tomography imaging of ovariectomized mice with an *in silico* mechanoregulated micro-MPA model to examine how estrogen-dependent cellular effects can produce observed bone loss and remodeling patterns. The model presented osteoblasts, osteoclasts and osteocytes, pre-osteoclasts and lining cells as discrete cytokine-producing agents interacting with their micro-environment to drive remodeling. Scans from four 15-week-old ovariectomized mice served as baseline data, with week 20 scans used for calibration, and weeks 22 and 24 for validation.

**Results:** Bone volume fraction (BV/TV) over the 9-week simulation closely matched the experimental data. The model also generated mechanoregulated sites of bone formation and resorption that visually corresponded to those observed experimentally (Figure 1). Estrogen depletion triggered increased osteoclast recruitment and activity, resulting in decreased osteoprotegerin and increased sclerostin and RANKL levels, which together sustained elevated osteoclast activity.

**Conclusion:** Our micro-MPA model reproduced both the remodeling patterns and the molecular responses to estrogen depletion, helping bridge the gap between *in vivo* observations and their underlying mechanistic drivers. Such validation strengthens the reliability of *in silico* models that support 3R-aligned research and lay the foundation for animal-free *in silico* preclinical trials of osteoporosis therapy.

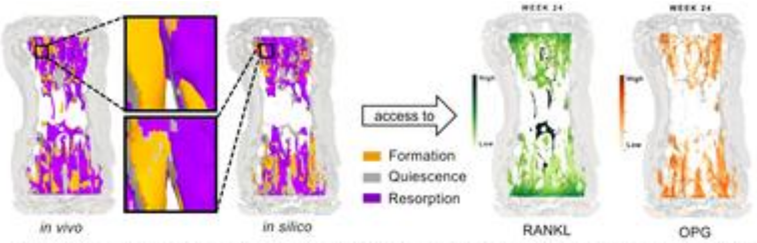

**Figure 1:** Realistic prediction of bone formation and resorption sites resulting from the mechanoregulating *in silico* model from week 15 to 24. In contrast to the *in vivo* data, the *in silico* model furthermore allows three-dimensional access to key molecules such as Receptor Activator of Nuclear Factor-κB Ligand (RANKL) and osteoprotegerin (OPG).

**P251**

### Discovery of plant-derived therapeutic candidates for prostate cancer bone metastases using high-throughput screening of Dutch horticultural extracts

*Iris Robbesom - van den Berge[1], Martin van Royen[2], Pim French[3], Hans van Leeuwen[1],*
*Barbara Schrammeijer[4], Marjolein van Driel[1]*
*[1]Erasmus MC, Internal medicine, Rotterdam, Netherlands*
*[2]Erasmus MC, Pathology, Rotterdam, Netherlands*
*[3]Erasmus MC, Neurology, Rotterdam, Netherlands*
*[4]Rotterdam University of Applied Sciences, Biology and Medical laboratory Research, Rotterdam, Netherlands*

**Abstract Text**

Bone is a preferential site for metastatic outgrowth of prostate cancer. Given the absence of curative treatment for this terminal disease, there is a need for novel therapeutic options. Plant-derived agents provide a promising source, since they have multi-targeted anti-cancer potential and limited adverse effects. Many currently approved drugs are derived from natural sources, still most plant species have not been investigated yet. Moreover, in the Netherlands, there is a huge residual flow from horticulture of which the biomass could be (re)used for medicine.

The aim of this study was to find plant-derived drug candidates for bone metastases.

SV-HFO human fetal osteoblasts were co-cultured with GFP-transduced bone-metastatic prostate cancer cells (PC-3) in 384-well plates for 4 weeks. Cells were continuously treated with 6 µM of 2,240 plant extracts from Dutch horticulture (Dutch Extract Library). High-throughput screening was performed with the Opera Phenix high content screening system at weeks 1 and 4, analyzing GFP fluorescence (cancer cells) and Xylenol Orange (bone mineralization) using Harmony software.

Growth of PC-3 cells in co-culture with osteoblasts prevented their mineralization. Interestingly, 10 out of 2,240 plant extracts restored mineralization within a 4-week period while simultaneously decreasing the number of prostate cancer cells by a factor of 2 to 30. These extracts were derived from various plant species and different parts of the plants. Overall, 65% of the extracts inhibited cancer cell growth but did not promote bone formation, whereas 34% were found to be toxic to both cancer and bone cells. Intriguingly, 1% of extracts stimulated prostate cancer cell growth 2 to 6 times.

In conclusion: high throughput screening of the Dutch plant extract library in a human bone metastatic disease model revealed 10 hits that will be taken forward for validation and compound identification as possible therapeutic agents in bone metastasis.
