# Correction to: Abstracts of the ECTS 2026 Congress

**DOI:** 10.1093/jbmrpl/ziag181

**Published:** 2026-11-10

This is a correction to: Abstracts of the ECTS 2026 Congress, *JBMR Plus*, Volume 10, Issue Supplement_1, April 2026, Pages i1–i319, https://doi.org/10.1093/jbmrpl/ziag066

The originally published version of Abstract P019, ‘Anti-miR-195-Engineered Mesenchymal Stem Cells Promote Migration, Vascularization, and ECM Protein Expression In Vivo’ contained errors in affiliations, an erroneous figure, and missing funding information.

The affiliations have been corrected to read:

“Sara Moura^1^, Mafalda Montenegro Cortez^1^, Isabel Brandão^1,2^, Inês Alencastre^3^, Susana Gomes Santos^1^, Carla Cunha^1^, Maria Inês Almeida^1,5^

^1^i3s - Instituto de Investigação e Inovação em Saúde, INEB - Instituto de Engenharia Biomédica, Universidade do Porto, Porto, Portugal

^2^ICBAS - Instituto de Ciências Biomédicas Abel Salazar, Universidade do Porto, Porto, Portugal

^3^RISE - Health, Departamento de Nefrologia R&D, Faculdade de Medicina, Universidade do Porto, Porto, Portugal

^5^FFUP - UCIBIO, LaboratÓrio de Bioquímica, Faculdade de Farmácia da Universidade de Porto, Porto Portugal”

Instead of:

“Sara Moura^1^, Mafalda Montenegro Cortez^2^, Isabel Brandão^3^, Inês Alencastre^4^, Susana Gomes Santos^5^, Carla Cunha^6^, Maria Inês Almeida^7^

^1^i3s - Instituto de Investigação e Inovação em Saúde, Tumour and Microenvironment Interactions, Porto, Portugal

^2^i3S - Instituto de Investigação e Inovação em Saúde- Universidade do Porto- Porto- Portugal, Microenvironments for New Therapies, Porto, Portugal

^3^i3S - Instituto de Investigação e Inovação em Saúde- Universidade do Porto- Porto- Portugal / ICBAS - Instituto de Ciências Biomédicas Abel Salazar- Universidade do Porto- Porto- Portugal, Advanced Graphene Biomaterials, Porto, Portugal

^4^RISE - Health- Departamento de Nefrologia R&D- Faculdade de Medicina- Universidade do Porto- Porto- Portugal, Nefrologia, Porto, Portugal

^5^i3S - Instituto de Investigação e Inovação em Saúde- Universidade do Porto- Porto- Portugal / INEB - Instituto de Engenharia Biomédica- Universidade do Porto- Portugal, Stem Cells in Regenerative Biology and Repair, Porto, Portugal

^6^i3S - Instituto de Investigação e Inovação em Saúde- Universidade do Porto- Porto- Portugal / INEB - Instituto de Engenharia Biomédica- Universidade do Porto- Portugal, Molecular Biomaterials, Porto, Portugal

^7^i3S - Instituto de Investigação e Inovação em Saúde- Universidade do Porto- Porto- Portugal / INEB - Instituto de Engenharia Biomédica- Universidade do Porto- Portugal, Tumour and Microenvironment Interactions, Porto, Portugal”

The following funding statement has been added:

“This work was funded by COMPETE2030-FEDER-00727500 (Operation no. 16123; FCT)”.

The corrected image has been provided below:


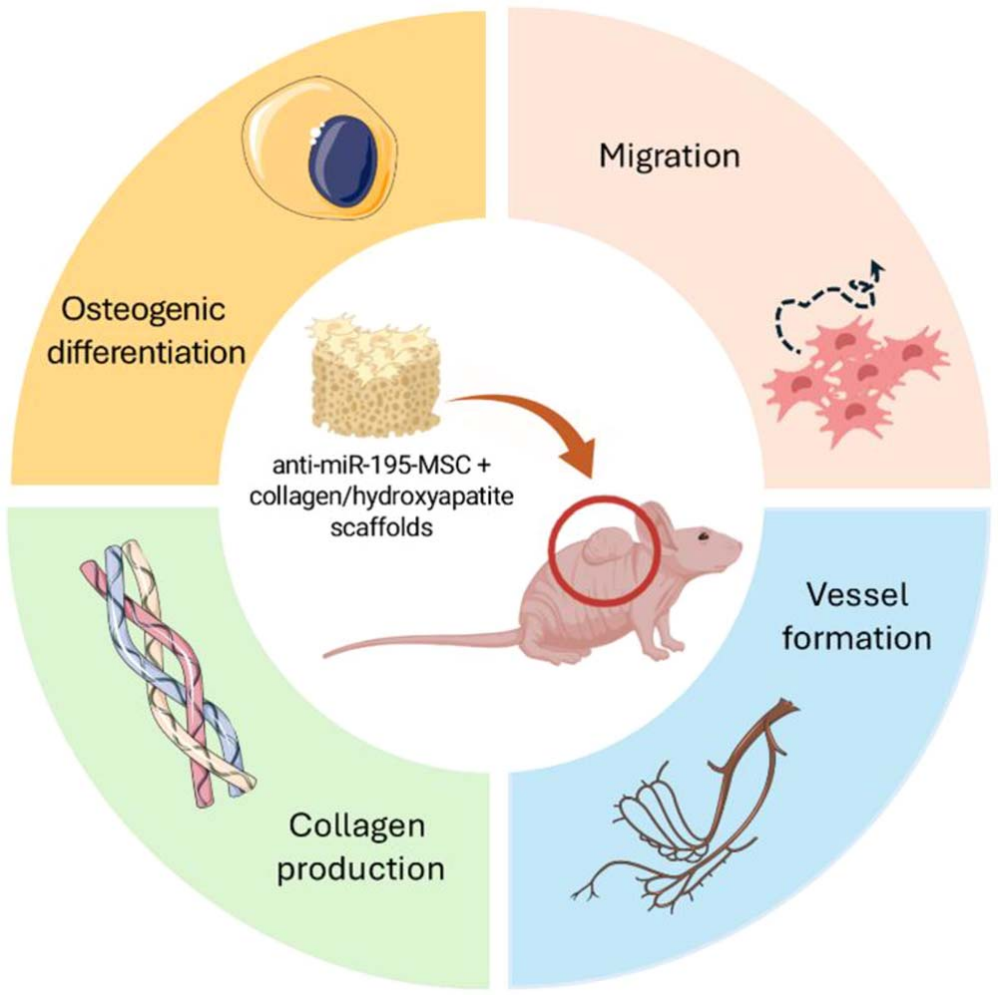


These changes have been made to the published supplement.

